# A Strong Lewis Acidic Diethylsilylium Catalyst for Direct Sulfonamidation of Challenging Ketones

**DOI:** 10.1002/advs.75783

**Published:** 2026-05-26

**Authors:** Woo Hee Kim, Muhammad Israr, You Kyoung Chung, Shinwon Ham, Joonsuk Huh, Han Yong Bae

**Affiliations:** ^1^ Department of Chemistry Sungkyunkwan University Suwon Republic of Korea; ^2^ Department of Chemistry Yonsei University Seoul Republic of Korea; ^3^ Department of Quantum Information Yonsei University Incheon Republic of Korea

**Keywords:** amino esters, ion pairs, lewis acid catalysis, organocatalysis, organosilicon, silylium ion, sitagliptin, sulfonamide

## Abstract

Low‐substitution‐order silylium ions (R_2_HSi^+^), as less sterically congested analogs of carbenium ions, exhibit exceptionally high Lewis acidity and distinctive reactivity compared with tertiary counterparts. Herein, we present a strong Lewis acidic diethylsilylium catalysis that enables efficient reductive sulfonamidation of functionalized ketones, including β‐ketoesters, to access alkyl β‐amino esters. The active diethylsilylium ion pair ([Et_2_HSi]^+^[B(C_6_F_5_)_4_]^–^), in situ generated via hydride abstraction from diethylsilane by trityl tetrakis(pentafluorophenyl)borate, displays a remarkably enhanced catalytic performance. Diethylsilane functions dually as the reductant and precursor of the silylium species. Operating under solvent‐, metal‐, hydrogen‐, and additive‐free conditions, the protocol offers broad substrate scope and excellent scalability, exemplified by a concise three‐step, multi‐gram synthesis of the antidiabetic drug sitagliptin (> 4 g). Combined computational, NMR, and HR‐MS analyses reveal that the remarkable Lewis acidity of the secondary silylium ion enables activation of challenging substrates and affords exceptional selectivity. A mechanistic understanding of this process provides the foundation for a practical and sustainable catalytic system that expands the frontier of silylium‐mediated C─N bond formation.

## Graphical abstract

Low‐substitution‐order silylium ions (R_2_HSi^+^) exhibit exceptional Lewis acidic activity. We report diethylsilylium‐catalyzed reductive sulfonamidation of functionalized ketones to access β‐amino esters. The in situ generated ion pair shows enhanced reactivity, with diethylsilane serving as both reductant and precatalyst. The developed protocol proceeds under sustainable conditions with a broad scope and excellent scalability, highlighted by multi‐gram scale preparation of antidiabetic sitagliptin.

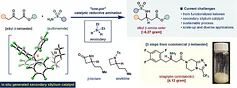

## 1 Introduction

**FIGURE 1:**
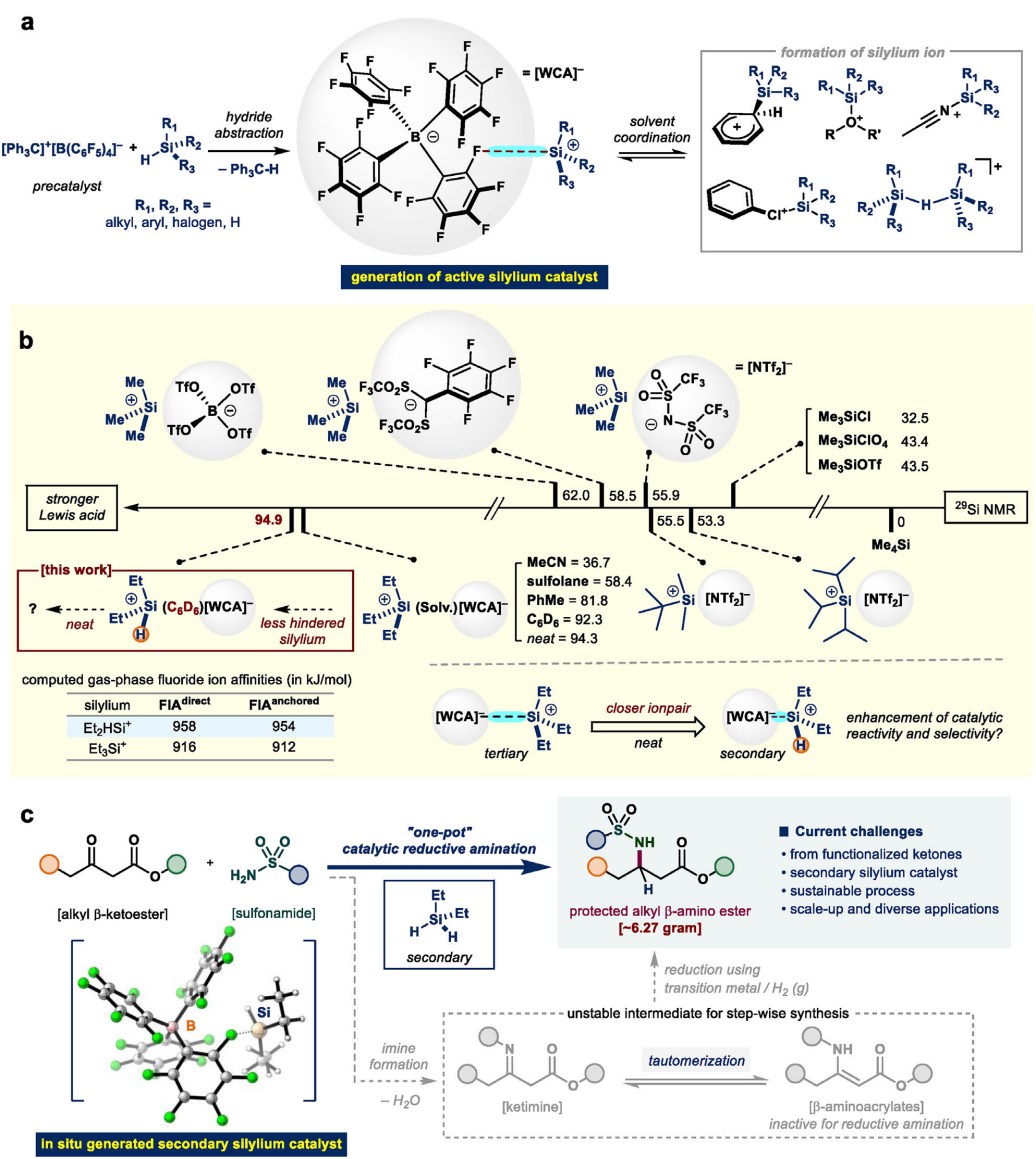
Catalytic direct sulfonamidation of challenging ketone to access alkyl β‐amino esters. (a) Generation of active silylium‐[WCA]^–^ Lewis acid. (b) Structure‐Lewis acidity relationship of various silylium catalysts. (c) This work: Super Lewis acidic diethylsilylium catalyst for direct sulfonamidation.

Silylium ions (R_3_Si^+^), as heavier analogs of carbenium ions, represent fundamentally important yet comparatively underdeveloped reactive intermediates whose structural and reactivity features remain to be fully elucidated [1, 2, 3, 4]. Hydrogen‐substituted silylium ions (R_2_HSi^+^, RH_2_Si^+^, and H_3_Si^+^), regarded as too reactive to isolate, have recently been made accessible by Oestreich and co‐workers through a protolysis approach of carborane acid [5, 6]. Their structural elucidation revealed pronounced Lewis acidity and unique stabilization modes, representing a milestone in silylium chemistry. In general, silylium ions paired with weakly coordinating anions (WCAs) [7, 8, 9, 10, 11]. function as exceptionally powerful Lewis acids. Such species have drawn considerable attention as superacid catalysts for diverse transformations, including (i) hydrosilylation of olefins [12, 13, 14, 15, 16, 17], carbonyls [18], and imines [19], (ii) carbon–fluorine bond activation [20, 21, 22, 23], and (iii) a variety of carbon–carbon [24, 25, 26, 27, 28, 29, 30], and carbon–heteroatom [31, 32] bond‐forming reactions. The most straightforward approach for silylium chemistry involves using a silane (R_3_SiH) as a precatalyst and reductant. In situ generation of the active silylium species can be achieved by Bartlett‐Condon‐Schneider protocol (hydride abstraction) [33] using neutral acceptor such as trityl tetrakis(pentafluorophenyl)borate ([Ph_3_C]^+^[B(C_6_F_5_)_4_]^–^) [34, 35], or protolysis using super Brønsted acids (e.g., trifluoromethanesulfonic acid, bistriflimide, and C─H acid). Generated silylium–counteranion pairs are stabilized by donors (e.g., solvents) of varying polarity and structural diversity, and such coordination can affect both the reactivity and selectivity of the catalytic process [9, 36, 37]. Under solvent‐free conditions with excess hydrosilane, it is feasible that the silylium ion can be stabilized through η^1^ Si–H coordination with another hydrosilane, generating hydride‐bridged silylium ion‐like species. (Figure 1a) [38, 39, 40].

Importantly, even subtle variations in the ion‐pair structure can lead to markedly different outcomes. For instance, trimethylsilylium ions (Me_3_Si^+^) paired with relatively less electron delocalized, compact counteranions (e.g., chloride, perchlorate, and triflate), displaying attenuated Lewis acidity, as indicated by ^29^Si NMR chemical shifts in the range of δ = 32.5–43.5 ppm [41]. When the cationic framework is held constant as trimethylsilyl, replacement of the counteranion can induce pronounced changes in acidity (NTf_2_^–^, anion of C─H acid, and B(OTf)_4_^–^, δ = 55.9–62.0) [42, 43, 44]. Conversely, fine structural modifications at the silylium center (e.g., trimethylsilyl, *tert*‐butyldimethylsilyl, and triisopropylsilyl) [41, 45, 46], with a fixed counterion (NTf_2–_), also translate into significant variations in Lewis acidity (δ = 53.3–55.9). Solvent coordination further complicates this landscape (e.g., acetonitrile, sulfolane, toluene, C_6_D_6_, and neat condition), underscoring the delicate interaction of steric and electronic factors in controlling activity of triethylsilyl tetrakis(pentafluorophenyl)borate ([Et_3_Si]^+^[B(C_6_F_5_)_4_]^–^, ^29^Si NMR δ = 36.7–94.3), a representative ion pair commonly utilized in silylium chemistry [35]. Motivated by these facts, we turned our attention to the effect of reducing the substitution order of silylium ions from tertiary to secondary on their catalytic properties. Secondary silylium ions exhibit higher fluoride ion affinity (FIA, computed gas‐phase fluoride ion affinities in kJ/mol) values (diethylsilylium = 958 (direct) and 954 (anchored)) than their tertiary analogue (triethylsilylium = 916 (direct) and 912 (anchored)) [47], indicating stronger intrinsic Lewis acidity and more effective activation of electrophiles with WCAs due to perhaps lower steric hindrance. We anticipated that such structural features could lead to significant improvements in catalytic transformations (Figure 1b).

Herein, we report a strong Lewis acidic diethylsilylium catalysis, where secondary silylium exhibited remarkable enhancements in both reactivity and selectivity, enabling the efficient reductive sulfonamidation of challenging and functionalized ketones such as β‐ketoesters. A seminal report by Oestreich and co‐workers demonstrated the catalytic hydrosilylation of preformed *N*‐tosyl imines derived from aldehydes and acetophenone using tertiary silylium–[WCA]– pairs, in situ generated in 1,2‐difluorobenzene or 1,2‐dichlorobenzene as a solvent [19]. Although previous studies exhibited high reactivity toward aldehyde‐derived imines, the acetophenone‐derived imine afforded only moderate conversion (76%), indicating room for further optimization. In contrast, this solvent‐, metal‐, and additive‐free protocol demonstrated excellent scalability, enabling multi‐gram synthesis exemplified by a concise three‐step preparation of the antidiabetic blockbuster drug sitagliptin (> 4 g). Furthermore, mechanistic investigations combining density functional theory (DFT), ^29^Si NMR spectroscopy, and high‐resolution mass spectrometry (HR‐MS) established the unique catalytic utility of secondary silylium systems (Figure 1c).

## 2 Results and Discussion

### 2.1 Optimization of the Reaction Conditions

To develop the catalytic reductive sulfonamidation reaction, ethyl 3‐oxohexanoate was employed as the model substrate. *p*‐toluenesulfonamide (TsNH_2_) and diethylsilane were selected as the aminating and organosilicon reductant, respectively (Table 1). Initial attempts under solvent‐ and catalyst‐free conditions at 80°C resulted in no detectable product formation. Various metal Lewis acid salts (Fe(OTf)_3_, Cu(OTf)_2_, Ag(OTf), Sc(OTf)_3_, Bi(OTf)_3_, and Bi(OAc)_3_) were ineffective (entries 1 and 2) [48].

**TABLE 1:** Optimization of the catalyst^a^.

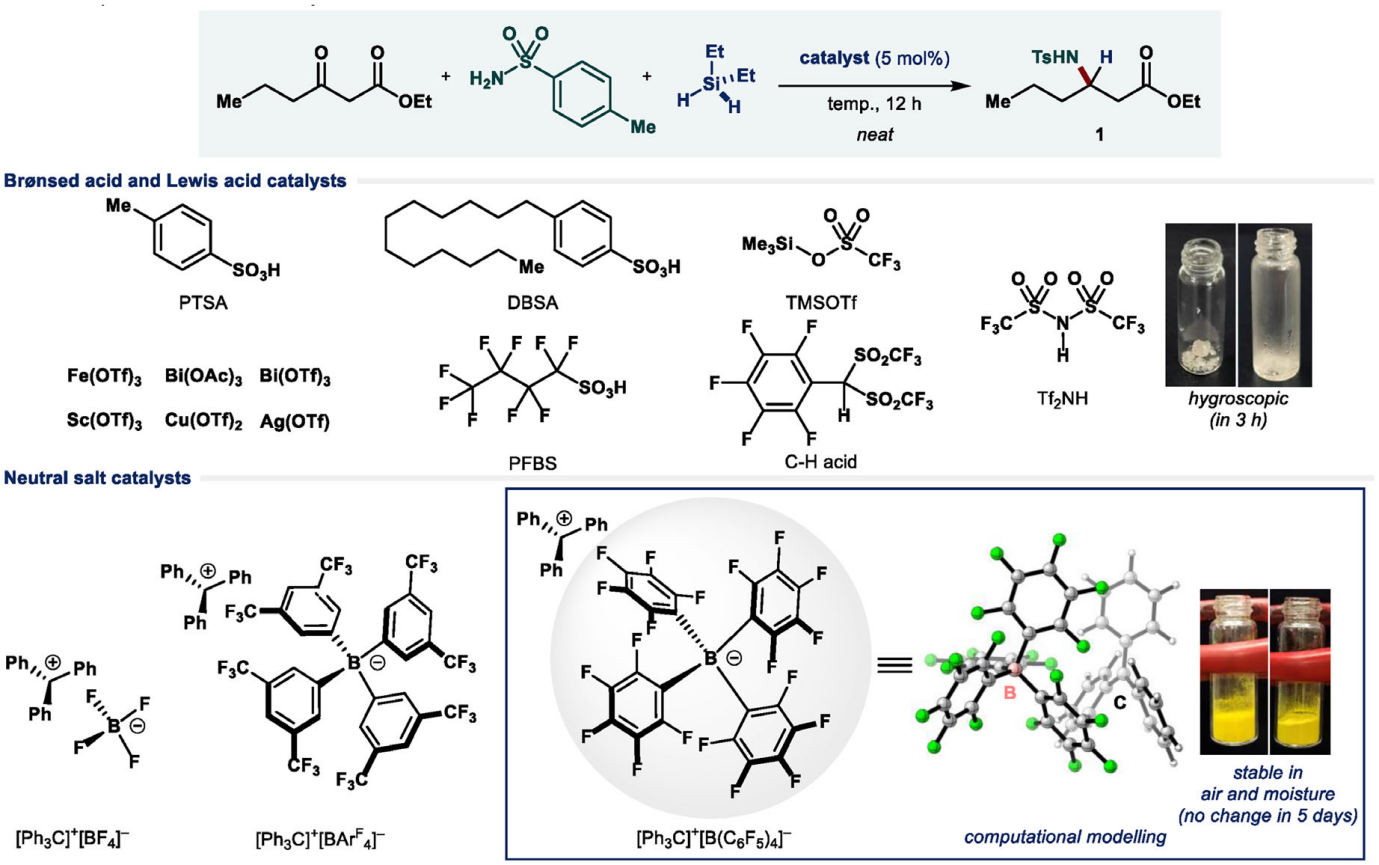			

Entry^a^	Catalyst	Temp. (°C)	Yield (%)^b^
1	*no catalyst*	80	0
2	*various metal Lewis acids*	80	0
3	PTSA	60	< 3
4	DBSA	60	< 3
5	Tf_2_NH	60	30
6	PFBS	60	42
7	TfOH	60	48
8	C─H acid	60	51
9	TMSOTf	60	52
10	[Ph_3_C]^+^B(C_6_F_5_)_4_]^–^	60	73
11	[Ph_3_C]^+^[BF_4_]^–^	80	0
12	[Ph_3_C]^+^[BAr^F^_4_]^–^	80	25
13^c^	[Ph_3_C]^+^B(C_6_F_5_)_4_]^–^	80	68
14^d^	[Ph_3_C]^+^B(C_6_F_5_)_4_]^–^	80	85
15^e^	[Ph_3_C]^+^B(C_6_F_5_)_4_]^–^	80	84
16^f^, ^g^	[Ph_3_C]^+^B(C_6_F_5_)_4_]^–^	80	89
17^f^, ^h^	[Ph_3_C]^+^B(C_6_F_5_)_4_]^–^	80	90 (85)

^a^ Reaction was performed using ethyl 3‐oxohexanoate (0.2 mmol, 1 equiv.), TsNH_2_ (0.3 mmol, 1.5 equiv.), silane (0.3 mmol, 1.5 equiv.), and the catalyst (0.01 mmol, 5 mol%) without solvent.

^b^ Yield (%) was determined using ^1^H NMR analysis.

^c^ 3 mol% catalyst was used.

^d^ TsNH_2_ (0.3 mmol, 1.5 equiv.) and Et_2_SiH_2_ (0.4 mmol, 2 equiv.) were used.

^e^ TsNH_2_ (0.4 mmol, 2 equiv.) and Et_2_SiH_2_ (0.4 mmol, 2 equiv.) were used.

^f^ TsNH_2_ (0.4 mmol, 2 equiv.) and Et_2_SiH_2_ (0.5 mmol, 2.5 equiv.) were used.

^g^ [Ph_3_C]^+^B(C_6_F_5_)_4_]– that was exposed to air for 5 days was used.

^h^ Isolated yield. Ts = *p*‐toluenesulfonyl.

Strong Brønsted acids such as *p*‐toluenesulfonic acid (PTSA) and 4‐dodecylbenzenesulfonic acid (DBSA), previously utilized in our previous “on‐water” allylation studies [49, 50], exhibited almost no reactivity (entries 3 and 4). However, triflimide (Tf_2_NH) [25, 51], which demonstrated high catalytic efficiency in hydroboration‐based reductive amination studies [52], afforded the desired product in moderate yield (30%, entry 5). Similarly, perfluorobutanesulfonic acid (PFBS), triflic acid (TfOH), and pentafluorophenylbis(triflyl)methane (C─H acid) [53] exhibited relatively high catalytic activities, with yields ranging from 42% to 51% (entries 6–8). Furthermore, trimethylsilyl trifluoromethanesulfonate (TMSOTf) afforded the desired product in moderate yield (entry 9).

Given our previous findings on the silylium‐catalyzed cyanosilylation of ketones, we employed trityl tetrakis(pentafluorophenyl)borate as the precatalyst [30, 34]. This catalytic strategy generates an ion pair of a highly reactive silylium ion and a WCA [11] in situ, thereby efficiently enhancing the Lewis acidity. Remarkably, this approach significantly improved the yield (73%, entry 10). According to previous studies, the counteranion effect is critical for silylium catalysis. Consistent with previous reports, the use of BF_4_^–^ [9, 36] or 3,5‐bis(trifluoromethyl)phenylborate [B(ArF)_4_]– [8] resulted in no activity or very low activity, providing yields of 0% and 25%, respectively (entries 11 and 12).

Further optimization showed that the optimal reactant quantities were TsNH_2_ (0.4 mmol, 2 equiv.) and Et_2_SiH_2_ (0.5 mmol, 2.5 equiv.), affording the desired product in 90% yield (85% isolated yield, entries 13–17). Notably, unlike the hygroscopic nature of Tf_2_NH, trityl tetrakis(pentafluorophenyl)borate exhibited excellent air and moisture stability, maintaining consistent performance even when the catalyst was exposed to air for 5 days in an uncapped vial (entry 16).

### 2.2 Effects of the Reductant and Reaction Medium, and Studies (^1^H, ^13^C, ^29^Si NMR and DFT) for Silylium Ion Pairs

**FIGURE 2:**
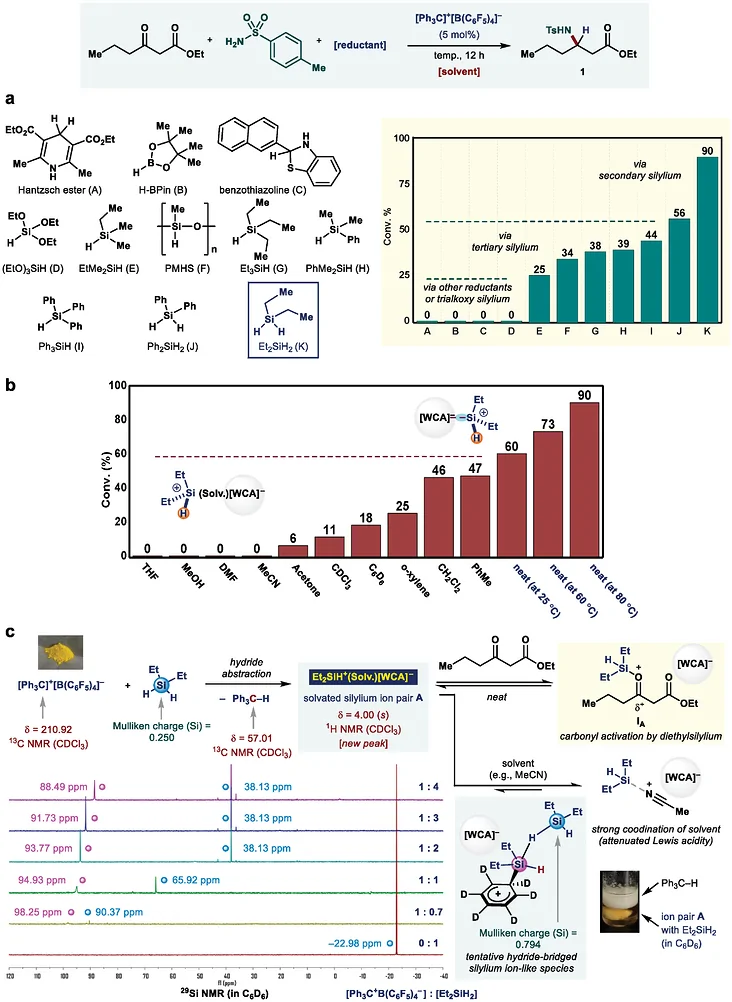
Mechanistic investigations. (a) Effects of the reductant. (b) Effects of the reaction medium. (c) NMR titration study for the generation of silylium ion pair **A** [a] Reaction was performed using ethyl 3‐oxohexanoate (0.2 mmol, 1 equiv.), TsNH_2_ (0.4 mmol, 2 equiv.), the reductant (0.5 mmol, 2.5 equiv.), and [Ph_3_C]^+^[B(C_6_F_5_)_4_]– (0.01 mmol, 5 mol%) in solvent at 80°C. Yield (%) was determined using ^1^H NMR analysis.

The selection of an appropriate reductant is essential for reductive sulfonamidation reactions (Figure 2a). Common reductants frequently employed in catalytic reduction chemistry, such as Hantzsch esters [54], pinacolborane, and benzothiazoline [55] did not show reactivity (entries A–C). Therefore, various silane reductants were investigated. The structure of the silane significantly influenced the reactivity. For example, (EtO)_3_SiH exhibited no activity (entry D). Silanes bearing three substituents, such as EtMe_2_SiH, polymethylhydrosiloxane (PMHS), Et_3_SiH, PhMe_2_SiH, and Ph_3_SiH demonstrated moderate reactivity, yielding products in the range of 25%–44% (entries E–I). Interestingly, silanes with two substituents exhibited enhanced performance. For instance, Ph_2_SiH_2_ afforded the product in 56% yield (entry J), whereas Et_2_SiH_2_ delivered the highest yield of 90% (entry K).

The reaction medium also played a critical role in determining the outcomes (Figure 2b). Polar solvents, such as tetrahydrofuran (THF), MeOH, dimethylformamide (DMF), and acetonitrile (MeCN), exhibited no reactivity, whereas acetone resulted in only a 6% yield. Deuterated solvents, including CDCl_3_ and C_6_D_6_, provided low yields of 11% and 18%, respectively. Nonpolar solvents such as *o*‐xylene, CH_2_Cl_2_, and toluene afforded moderate yields ranging from 25% to 47%. Remarkably, solvent‐free conditions provided improved results, particularly when the reaction temperature was optimized. While moderate yields were observed at 25°C–60°C (60% and 73%, respectively), heating the reaction to 80°C resulted in a maximum yield of 90%.

The silane‐mediated reductive sulfonamidation reaction was presumed to be catalyzed by the active silylium ion pair **A**. We conducted NMR studies to investigate the nature of the reactive species based on a previous study that used trityl tetrakis(pentafluorophenyl)borate and TMS‐CN (Figure 2c) [30]. Using CDCl_3_ as the solvent at room temperature, we mixed trityl tetrakis(pentafluorophenyl)borate and Et_2_SiH_2_ in a 1:1 molar ratio. Upon hydride abstraction, a new singlet species appeared in the ^1^H NMR spectrum at δ = 4.00, which we attributed to the Si─H signal of EtHSi^+^. (see the Supporting Information for details). Simultaneously, the ^13^C NMR spectrum indicated the disappearance of the carbocation center of triphenylcarbenium (δ = 210.92) and the formation of triphenylmethane (δ = 57.01, tertiary carbon).

To further investigate the nature of the reactive species, we performed titration experiments using C_6_D_6_ as the solvent and monitored the reaction by ^29^Si NMR at ambient temperature. When Et_2_SiH_2_ (δ = −22.98) was introduced under catalyst‐free conditions, it rapidly disappeared and generated two new species. By varying the molar ratio of Et_2_SiH_2_ to trityl tetrakis(pentafluorophenyl)borate from 1:0.7 to 1:4, we observed that the presumed active silylium ion pair **A** emerged at δ = 98.25, which progressively shifted to δ = 94.93, 93.77, 91.73, and 88.49 with increasing silane concentration. By contrast, another silicon species dynamically shifted from δ = 90.37 to 38.13 (1:0.7 to 1:2) and remained unchanged thereafter (1:2 to 1:4). These observations suggest that the solvent‐coordinated silylium ion pair (pink‐dotted Si) facilitates hydride activation, resulting in weakly activated Si species that are not fully converted to the silylium form (blue‐dotted Si). It is hypothesized that under solvent‐free conditions, silylium ion pair **A** acts as a Lewis acid to activate ethyl 3‐oxohexanoate, leading to the formation of intermediate **I_A_**. By contrast, polar and Lewis basic solvents (such as MeCN) are presumed to interact with silylium, thereby relatively weakening its Lewis acid activity and hindering the catalytic process. DFT calculations of Mulliken charges on the silicon atom in diethylsilane further support the generation of a Lewis acidic silylium ion. The Mulliken charge of the silicon atom in diethylsilane alone was calculated to be 0.250, which increased to 0.794 upon hydride activation by ion pair **A** (see the Supporting Information for details).

### 2.3 Control Experiments and DFT‐Based Computations for Relative Catalytic Activity

**FIGURE 3:**
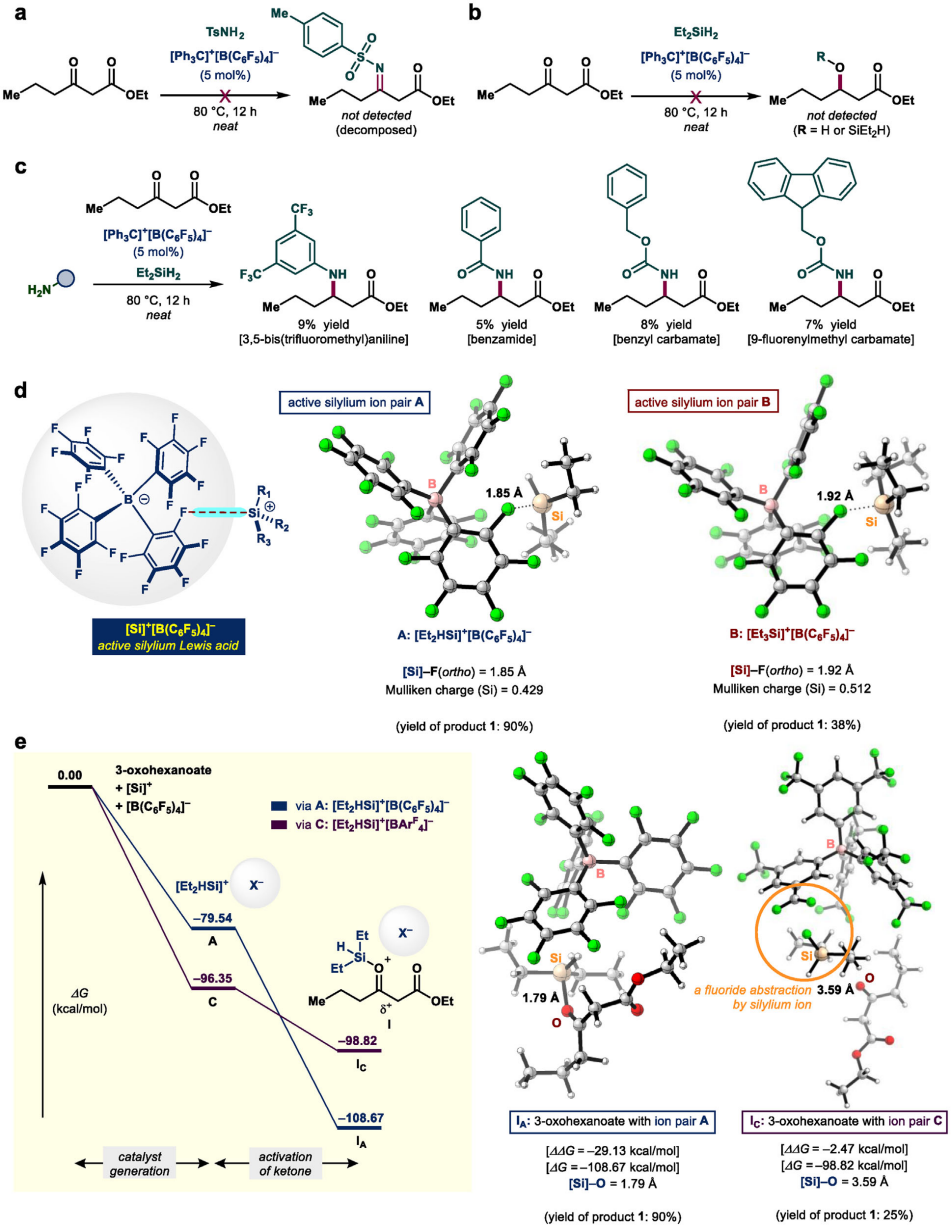
Control experiments and DFT‐based computational studies conducted to compare the catalyst structures. (a) Reaction without Et_2_SiH_2_. (b) Reaction without TsNH_2_. (c) Reactions using alternative amines. (d) DFT computation: Comparison of the active silylium Lewis acid with different silanes [Et_2_HSi^+^ vs. Et_3_Si^+^] (e) Energy profile comparison: WCA effect on active silylium Lewis acid [B(C_6_F_5_)_4–_ vs. BAr^F–^].

We conducted a series of control experiments to elucidate the roles of the various reaction components. In the absence of Et_2_SiH_2_ under standard conditions, imine formation was not observed (Figure 3a). Similarly, the omission of TsNH_2_ led to no ketone reduction (Figure 3b). When TsNH_2_ was replaced with other nitrogen nucleophiles such as 3,5‐bis(trifluoromethyl)aniline, benzamide, benzyl carbamate, and 9‐fluorenylmethyl carbamate, the reactivity was drastically diminished, providing only 5%–9% yields (Figure 3c). These results indicate that all the reaction components are essential for catalytic reactions.

Computational studies were conducted to further investigate the details of the silylium catalyst and provide theoretical insights (using def2‐TZVP basis set). First, we examined how the structure of the silylium ion influenced its relative Lewis acidity when the counteranion concentration was held constant (Figure 3d). The structures of the active silylium ion pairs **A** and **B** are predicted to be in situ generated through the association of [Ph_3_C]^+^[B(C_6_F_5_)_4_]^–^ with Et_3_SiH or Et_2_SiH_2_. The computational approach revealed a weak interaction between the pentafluorophenyl group's ortho‐fluorine and the silyl cation upon ion pair formation. In the case of ion pair **A** ([Et_2_HSi]^+^[B(C_6_F_5_)_4_]–), the bond length between [Si] and F(ortho) was calculated to be 1.85 Å (with a Mulliken charge of 0.429 on the Si atom). Conversely, ion pair **B** ([Et_3_Si]^+^[B(C_6_F_5_)_4_]^–^) exhibited a longer bond length of 1.92 Å (with a Mulliken charge of 0.512 on the Si atom). This difference is likely attributable to the increased steric hindrance imposed by the additional ethyl group, which interferes with effective ion pair formation; consequently, ion pair **A** is expected to form a stronger Lewis acid‐base interaction than ion pair **B**.

Next, we investigated the relative Lewis acidity of the same silylium species (Et_2_HSi^+^) with different counteranions (Figure 3e). Comparative analysis between ion pairs **A** and **C** (a salt featuring the counteranion tetrakis(3,5‐bis(trifluoromethyl)phenyl)borate: [Et_2_HSi]^+^[B(ArF)_4_]^−^) revealed that **C** was more thermodynamically stable (**A**: ∆G = −79.54 kcal/mol; **C**: ∆G = −96.35 kcal/mol). However, upon coordination with ethyl 3‐oxohexanoate to form complex **I**, the structure of **I_A_** was found to be 9.85 kcal/mol more stable than that of **I_C_** (**I_A_**: ∆G = −108.67 kcal/mol; **I_C_**: ∆G = −98.82 kcal/mol). The Si─O bond lengths of **I_A_** and **I_C_** were calculated to be 1.79 Å and 3.59 Å, respectively, indicating that ion pair **A** effectively activates the ketone substrate. Interestingly, analysis of the [B(ArF)_4_]^–^ counteranion in **I_C_** revealed that fluoride abstraction occurs via the silylium ion, consistent with Sakurai's observation [8], and is likely responsible for the diminished overall catalytic activity. Notably, these computational results align well with the experimental trends observed in Table 1.

### 2.4 DFT‐Based Computations for Relative Energies of Intermediates

**FIGURE 4:**
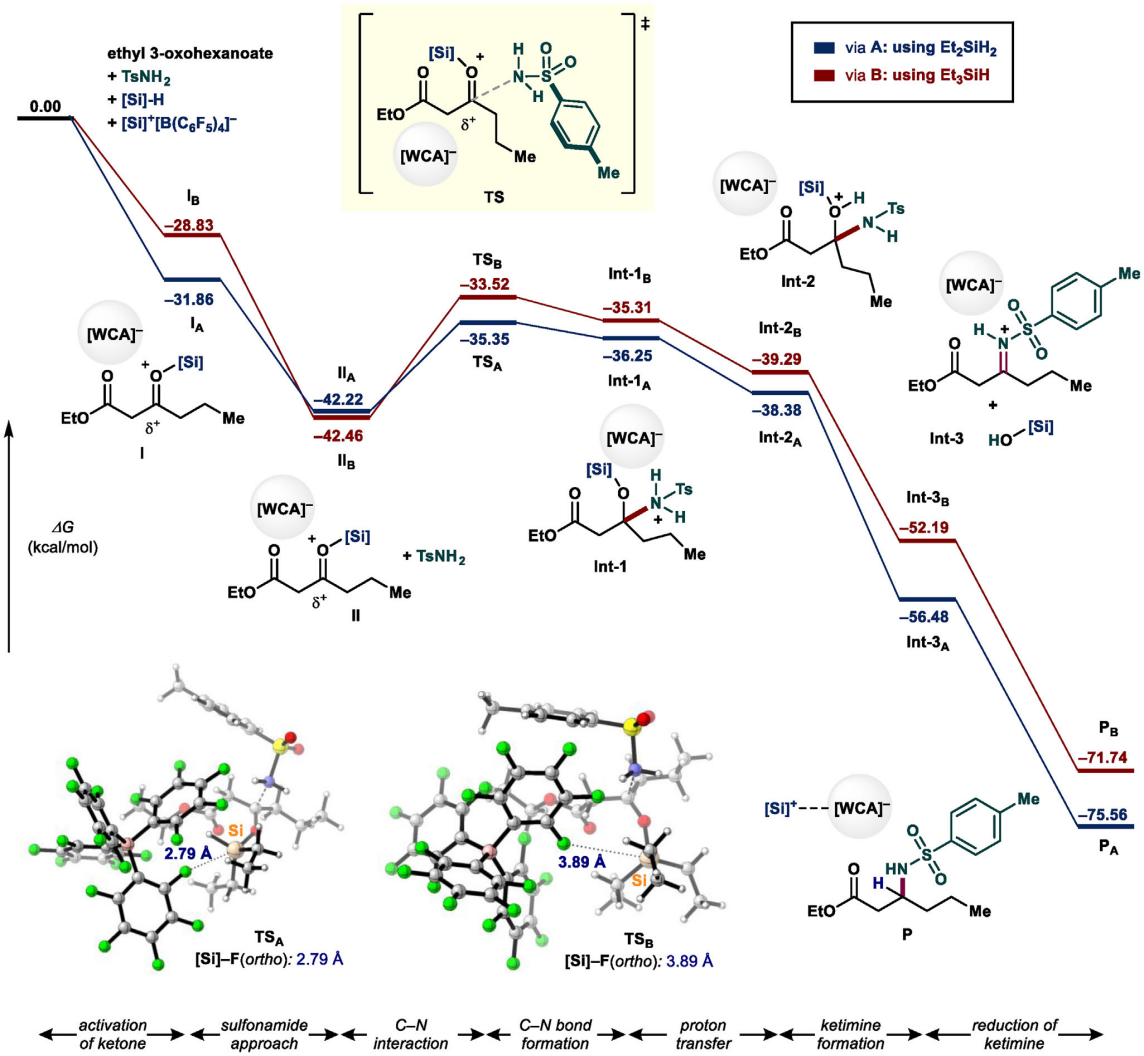
Relative energy profiles of silylium‐catalyzed reductive sulfonamidation.

The complete quantum mechanically calculated catalytic cycle for accessing *N*‐Ts‐β‐amino ester **1** using ion pair **A** ([Et_2_HSi]^+^[B(C_6_F_5_)_4_]^–^) and ion pair **B** ([Et_3_Si]^+^[B(C_6_F_5_)_4_]^–^) is summarized in Figure 4 (using def2‐SVP basis set). The catalytic cycle begins with the activation of ethyl 3‐oxohexanoate by the reactive silylium Lewis acid complex, leading to the formation of complex **I** (**I_A_**, ∆G = −31.86 kcal/mol; **I_B_**, ∆G = −28.83 kcal/mol).

When TsNH_2_ approaches the electrophilic carbonyl group, the electrostatic potential at the carbon center in ion pair **A** is slightly lower than that in ion pair **B**, explaining the observed energy difference of 0.24 kcal/mol in complex **II** (**II_A_**, ∆G = −42.22 kcal/mol; **II_B_**, ∆G = −42.46 kcal/mol).

Subsequently, the system undergoes a carbon–nitrogen σ‐bond formation, resulting in the formation of an imine intermediate (**Int‐1_A_**, ∆G = −36.25 kcal/mol; **Int‐1_B_**, ∆G = −35.31 kcal/mol) via the transition state (**TS_A_**, ∆G = −35.35 kcal/mol; **TS_B_**, ∆G = −33.52 kcal/mol). The transition state involving C─N bond formation is the key activated structure facilitated by ion pair **A**, providing a more favorable pathway with a relatively lower energy barrier (**TS_A_**, ∆∆G = 6.87 kcal/mol; **TS_B_**, ∆∆G = 8.94 kcal/mol). This enhanced activity is attributed to the steric hindrance induced by the additional ethyl group present in [Et_3_Si]^+^[B(C_6_F_5_)_4_]^–^, which reduces the Lewis acidity of the silylium ion. Following C─N bond formation, intramolecular proton transfer of the resulting silylated hemiaminal occurs, forming **Int‐2** (**Int‐2_A_**, ∆G = −38.38 kcal/mol; **Int‐2_B_**, ∆G = −39.29 kcal/mol). Subsequently, diethylsilanol is spontaneously released, leading to the generation of the protonated ketimine intermediate **Int‐3** (**Int‐3_A_**, ∆G = −56.48 kcal/mol; **Int‐3_B_**, ∆G = −52.19 kcal/mol).

Finally, the addition of hydrosilane facilitates the formation of the desired *N*‐Ts‐β‐amino ester **1** via hydrosilylation of the ketimine, producing the corresponding ion pair (**A** or **B**) (**P_A_**, ∆G = −75.56 kcal/mol; **P_B_**, ∆G = −71.74 kcal/mol).

### 2.5 HR‐MS Approaches to Elucidate Key Intermediates and Proposed Catalytic Cycle

**FIGURE 5:**
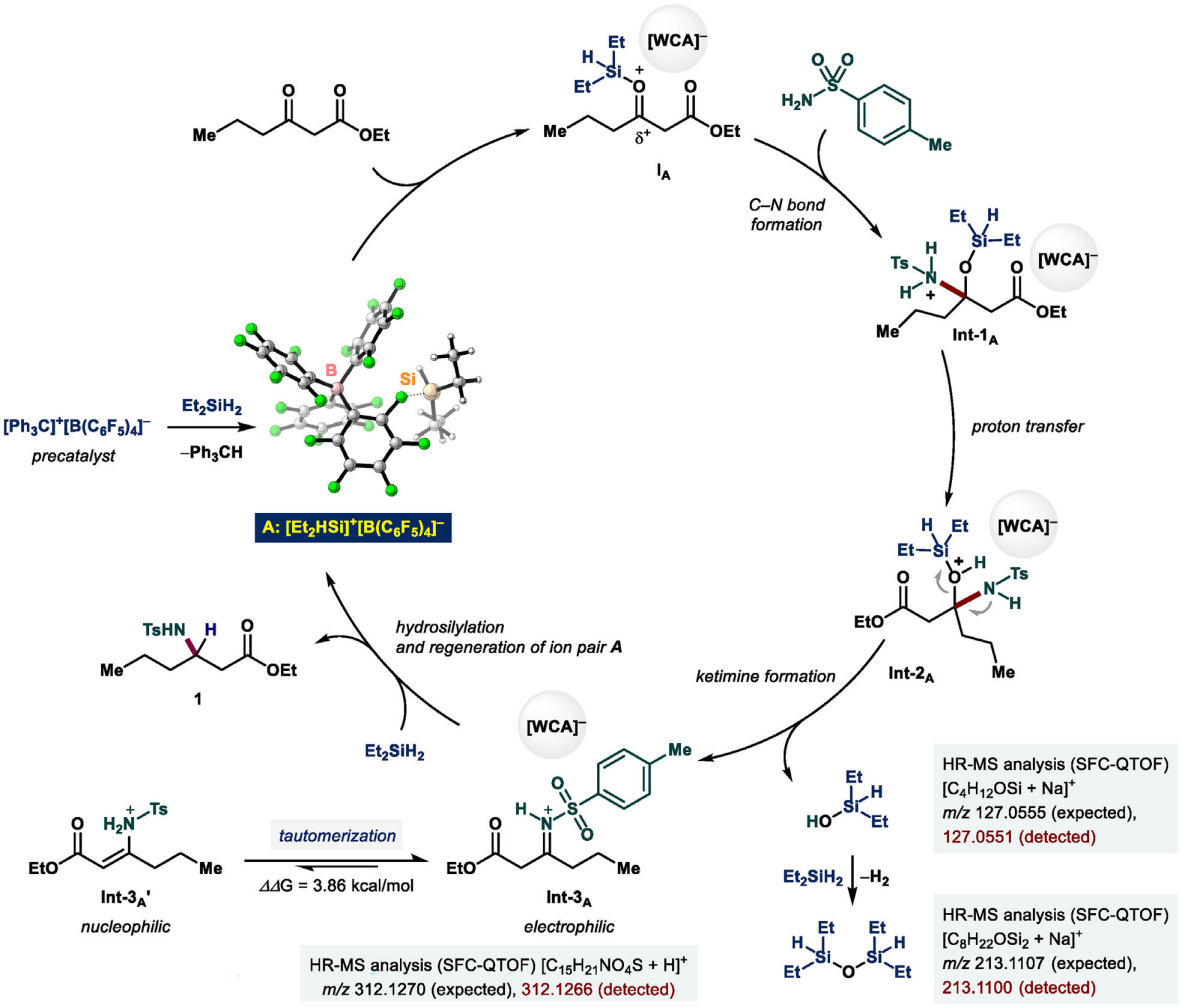
Proposed mechanism of silylium‐catalyzed reductive sulfonamidation.

Based on experimental, analytical, and quantum chemical results, a plausible catalytic cycle is proposed, as illustrated in Figure 5. Upon hydride abstraction from Et_2_SiH_2_, the precatalyst [Ph_3_C]^+^[B(C_6_F_5_)_4_]^–^ is converted into the catalytically active silylium ion pair **A** ([Et_2_HSi]^+^[B(C_6_F_5_)_4_]^–^), accompanied by the formation of Ph_3_CH. It is conceivable that the silylium ion pair **A** can further engage in a reversible equilibrium with a hydride‐bridged silylium ion‐like species in the presence of excess diethylsilane. The resulting free silylium Lewis acid ion pair **A** activates the electrophilic substrate ethyl 3‐oxohexanoate, generating complex **I_A_**, which subsequently undergoes C─N bond formation with TsNH_2_, yielding intermediates **Int‐1_A_** and **Int‐2_A_**.

To gain insight into the transient intermediates, the crude reaction mixture was subjected to in situ supercritical fluid chromatography‐high‐resolution quadrupole time‐of‐flight mass spectrometry (SFC‐HR‐QTOF‐MS). Notably, the possible protonated ketimine intermediate **Int‐3_A_** was successfully detected with high accuracy ([C_15_H_21_NO_4_S + H]^+^, *m/z* 312.1270 (calculated) and 312.1266 (observed)). Our computational studies indicate that the tosyl sulfonamide–derived ketimine (**Int‐3_A_**) is more favored than the corresponding enamine form (**Int‐3_A_′**) by 3.86 kcal/mol, thereby affording the formation of the reactive electrophilic species. In parallel, both diethylsilanol (Et_2_HSi–OH) and 1,1,3,3‐tetraethyldisiloxane (Et_2_HSi–O–SiHEt_2_) were observed as by‐products, providing evidence for the proposed reaction pathway ([C_4_H_12_OSi + Na]^+^, *m/z* 127.0555 (calculated) and 127.0551 (observed); [C_8_H_22_OSi_2_ + Na]^+^, *m/z* 213.1107 (calculated) and 213.1100 (observed)). The release of these side products serves as a thermodynamic driving force for ketimine formation.

The catalytic cycle is completed by the subsequent hydrosilylation of the ketimine intermediate by another equivalent of Et_2_SiH_2_, ultimately affording desired product **1**. Finally, the silylium ion pair catalyst **A** was regenerated, thus participating in the subsequent catalytic cycles.

### 2.6 Substrate Scope and Late‐Stage Modification

**FIGURE 6:**
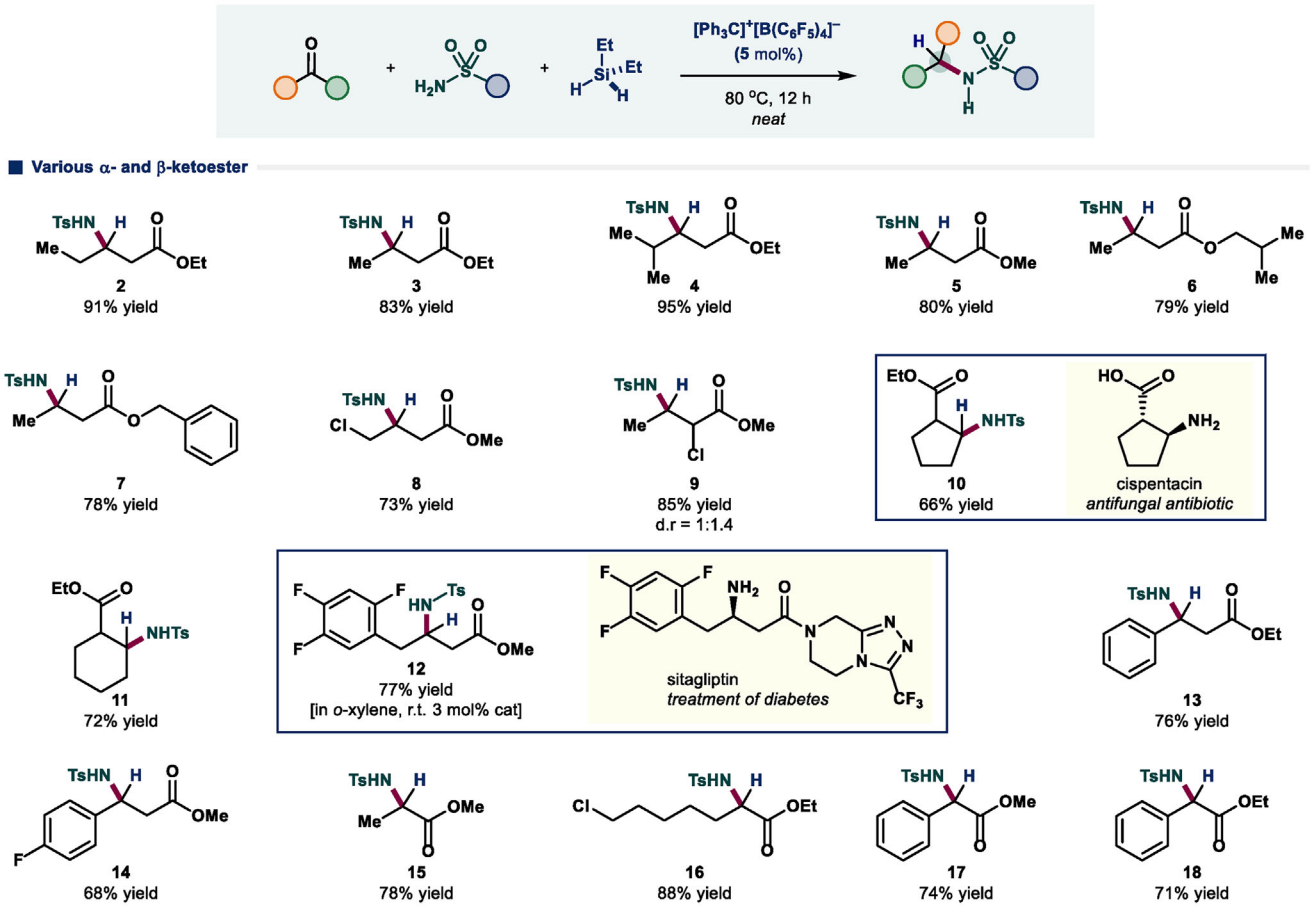
Substrate scope‐1. Reaction was performed using ketone (0.2 mmol, 1 equiv.), TsNH_2_ (0.4 mmol, 2 equiv.), Et_2_SiH_2_ (0.5 mmol, 2.5 equiv.), and [Ph_3_C]^+^[B(C_6_F_5_)_4_]– (0.01 mmol, 5 mol%) at 80°C. Yield was determined after purification by column chromatography.

The optimized conditions were applied to a broad range of ketone substrates for the catalytic reductive sulfonamidation (Figure 6). Various β‐ketoesters bearing linear, branched, and cyclic alkyl substituents were successfully transformed into the desired *N*‐Ts‐β‐amino esters with excellent yields (up to 95%, compounds **1–12**). Notably, a structural analog of the antifungal antibiotic cispentacin was synthesized in 66% yield (compound **10**). Moreover, a precursor of sitagliptin, an antidiabetic drug [56], was conveniently prepared from a commercially available ketone in a single step with a yield of 77% (compound **12**). β‐ketoesters substituted with aromatic groups also participated effectively in the reaction, affording the desired products in 76% and 68% yields (compounds **13** and **14**, respectively). Furthermore, various alkyl and aryl α‐ketoesters were successfully converted into the corresponding *N*‐Ts α‐amino esters with yields ranging from 71% to 88% (compounds **15–18**).

**FIGURE 7:**
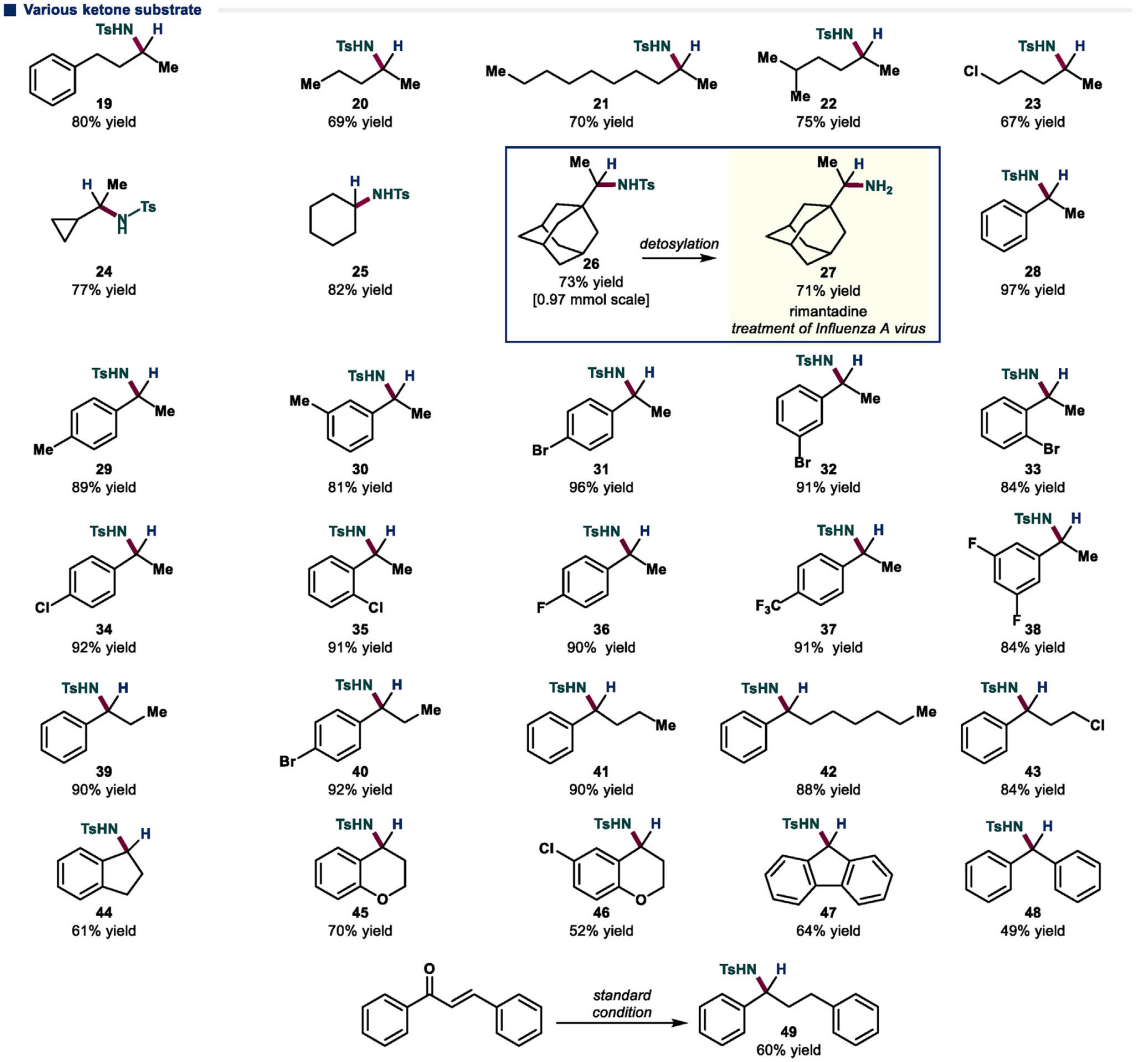
Substrate scope‐2.

The substrate scope was extended to various ketone classes, such as alkyl‐alkyl, alkyl‐aryl, aryl‐aryl, and cyclic ketones, which were smoothly transformed into the desired sulfonamide products (Figure 7, compounds **19–49**). This method exhibited excellent tolerance toward long‐chain, terminally branched, chloroalkyl, cyclopropyl, and cycloalkane‐substituted ketones. Specifically, compound **26** was successfully converted into compound **27** via detosylation, which serves as a precursor to rimantadine, a pharmaceutical agent used for the treatment of influenza A. For the conjugated ketone (chalcone), the reaction conditions led to a reduction of the alkenyl moiety, producing compound **49**.

**FIGURE 8:**
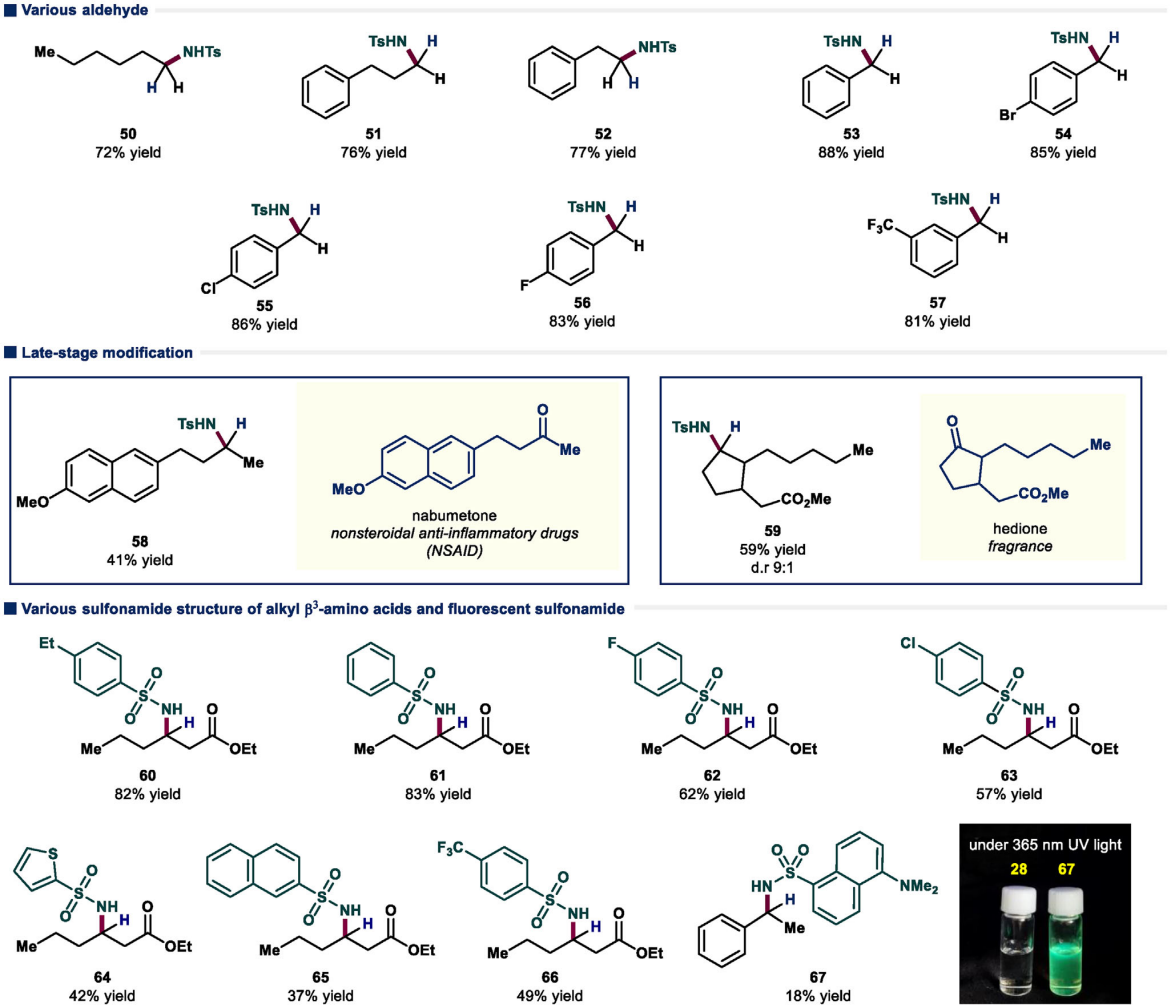
Substrate scope‐3.

This methodology also proved effective for aldehydes containing both alkyl chains and aryl groups, affording the corresponding *N*‐Ts sulfonamides in yields ranging from 72% to 88% (Figure 8, compounds **50–57**). Moreover, functional ketones, such as nabumetone (a nonsteroidal anti‐inflammatory drug) and hedione (a fragrance compound), were readily converted into *N*‐Ts sulfonamides at the late stage, with yields of 41% and 59% (d.r. = 9:1), respectively (compounds **58** and **59**). Additionally, a diverse array of substituted sulfonamides was successfully obtained in yields ranging from 37% to 83% (compounds **60–66**). Remarkably, the use of dansyl amide as a substrate led to the formation of a fluorescent sulfonamide [57], which was isolated in 18% yield (compound **67**).

### 2.7 Scale‐up Synthesis and Synthetic Applications: Multi‐Gram‐Scale, Three‐Step Production of Sitagliptin

**FIGURE 9:**
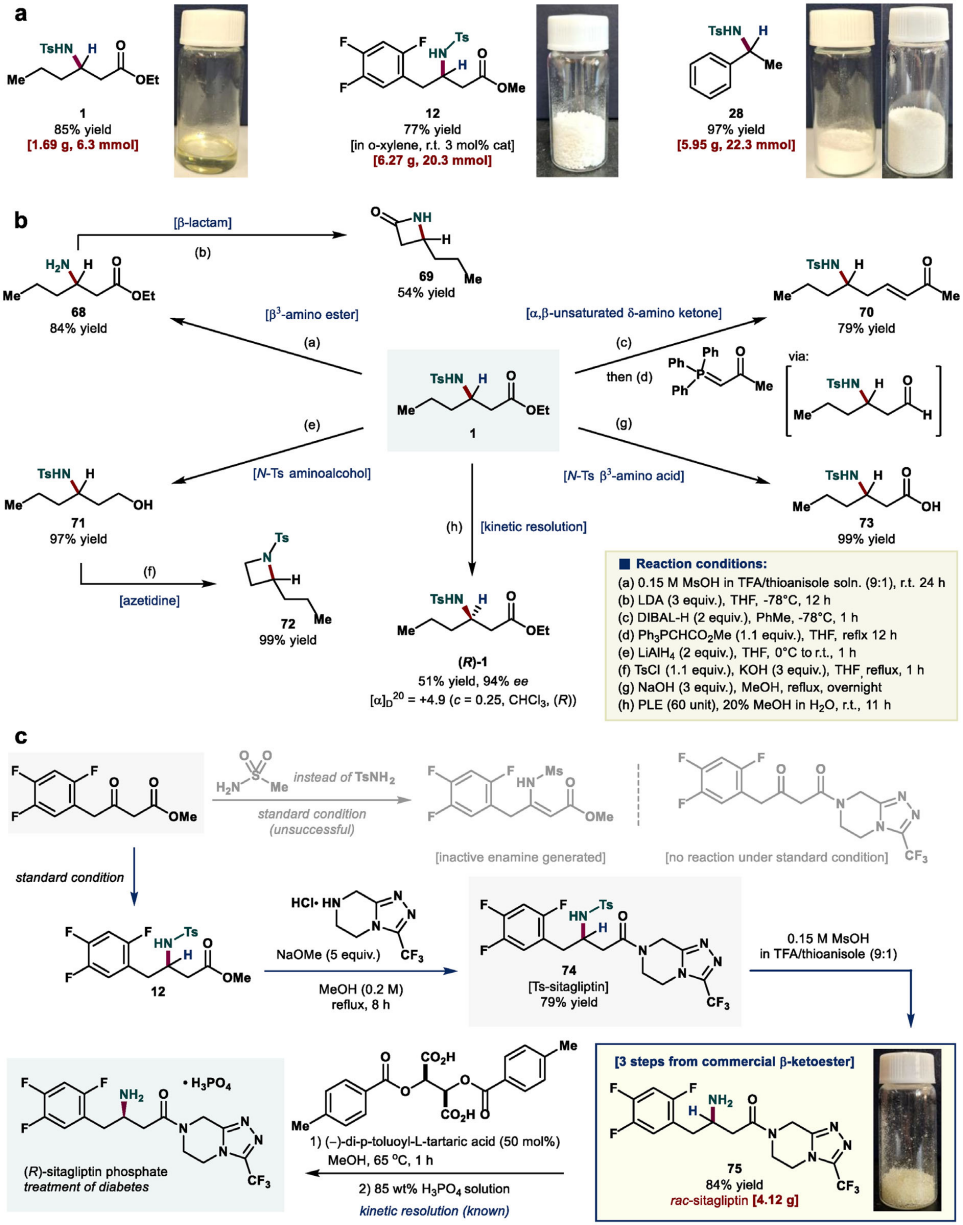
Practical utilities. (a) Scale‐up preparation of products. (b) Synthetic applications. (c) Multi‐gram scale production of sitagliptin.

To demonstrate the practical potential of the catalytic reductive sulfonamidation, multi‐gram scale syntheses were performed (Figure 9a). The model compound *N*‐Ts‐β‐amino ester **1** (1.69 g, 85% yield), sitagliptin precursor **12** (6.27 g, 77% yield), and alkyl sulfonamide product **28** (5.95 g, 97% yield) were successfully isolated.

The synthetic utility of the obtained alkyl *N*‐Ts‐β‐amino ester **1** is highlighted in Figure 9b. Tosyl deprotection using methanesulfonic acid (MsOH) in a TFA/thioanisole solution afforded the β‐amino ester **68** in 84% yield. Subsequent intramolecular amidation using lithium diisopropylamide (LDA) was performed to furnish β‐lactam, a key structural motif widely used in various antibiotic frameworks [58], in 54% yield. Reduction of the ester moiety to the corresponding aldehyde was achieved using diisobutylaluminium hydride (DIBAL‐H), which was directly subjected to a Horner‐Wadsworth‐Emmons reaction with (acetylmethylene)triphenylphosphorane, providing the α,β‐unsaturated‐δ‐amino ketone **70** in 79% yield. Furthermore, the reduction of compound **1** with the strong reductant lithium aluminum hydride (LiAlH_4_) produced the 1,3‐amino alcohol **71** with high efficiency (97% yield). Following tosyl protection of alcohol to introduce leaving group ability, azetidine **72**, an important motif in medicinal chemistry [59], was synthesized quantitatively through an S_N_2 reaction. Saponification of compound **1** yielded *N*‐Ts‐β‐amino acid **73** with quantitative efficiency. Notably, enzymatic kinetic resolution of the racemic compound **1** using Pig liver esterase (PLE) [60] afforded optically active *N*‐Ts‐β‐amino ester **(*R*)**‐**1** in 94% *ee* ([α]_D_^20^ = +4.9, c = 0.25, CHCl_3_, (*R*)).

The developed catalytic sulfonamidation enabled the streamlined synthesis of the blockbuster antidiabetic drug sitagliptin (Figure 9c). Careful selection of reaction conditions was critical; for instance, employing methanesulfonamide (MsNH_2_) under our standard conditions resulted in the formation of an en‐sulfonamide instead of the desired product, while utilizing the known intermediate β‐ketoamide as the starting material led to no reaction. Sitagliptin was successfully synthesized by the simple amidation of compound **12** with commercially available 3‐(trifluoromethyl)‐5,6,7,8‐tetrahydro‐[1,2,4]triazolo[4,3‐a]pyrazine hydrochloride, providing the desired *N*‐Ts‐β‐amino amide in 79% yield. Subsequent tosyl group deprotection afforded sitagliptin **75** in 84% yield on a preparative scale (4.12 g). Enantioenriched (*R*)‐sitagliptin phosphate can be readily obtained from a racemic mixture via kinetic resolution using (–)‐di‐p‐toluoyl‐L‐tartaric acid [61].

## 3 Conclusion

In summary, mechanistic investigations have led to the development of an efficient and practical approach for the synthesis of alkyl β‐amino esters, enabled by a lower‐substitution‐order silylium ion‐catalyzed reductive sulfonamidation. In this system, diethylsilane serves dually as the reductant and as the precursor for generating a highly active silylium Lewis acid catalyst in combination with trityl tetrakis(pentafluorophenyl)borate. This operationally simple process, which avoids the use of pressurized H_2_ and transition metals, exhibits a broad substrate scope and excellent scalability, as demonstrated by the three‐step multi‐gram production of sitagliptin. Comprehensive mechanistic studies using control experiments, DFT calculations, NMR, and HR‐MS confirmed the critical role of silylium ion pairs in substrate activation and catalytic efficiency. The robust nature of the diethylsilylium catalyst, combined with its operational simplicity, represents a substantial advancement in the synthesis of alkyl β‐amino esters and related derivatives. We believe that this approach will find broad applications in pharmaceutical synthesis and holds significant potential for extension to agrochemical and materials science.

## Conflicts of Interest

The authors declare no conflicts of interest.

## Supporting information




**Supporting File**: advs75783‐sup‐0001‐SuppMat.pdf.

## Data Availability

The data that supports the findings of this study are available in the supplementary material of this article.
