# Supplementary material for: A Strong Lewis Acidic Diethylsilylium Catalyst for Direct Sulfonamidation of Challenging Ketones
Source: Adv Sci (Weinh). 2026 May 26;13(46):e75783. doi: 10.1002/advs.75783 (PMC13336042; doi:10.1002/advs.75783)
Supplement: Supplementary file 1 [file ADVS-13-e75783-s001.pdf]

Supplementary Information

A Strong Lewis Acidic Diethylsilylium Catalyst for Direct Sulfonamidation of Challenging Ketones

Woo Hee Kim,[a†] Muhammad Israr,[a†‡] You Kyoung Chung,[b†] Shinwon Ham,[a] Joonsuk Huh,[a,b,c] Han Yong Bae*[a]*

[a]Department of Chemistry, Sungkyunkwan University 2066, Seobu-ro, Jangan-gu, Suwon, 16419 Republic of Korea

[b]Department of Chemistry, Yonsei University 50, Yonsei-ro, Seodaemun-gu, Seoul, 03722 Republic of Korea

[c]Department of Quantum Information, Yonsei University, Incheon, 21983, Republic of Korea.

‡ Present address: Center for Integrative Petroleum Research, College of Petroleum Engineering and Geosciences, King Fahd University of Petroleum and Minerals, Dhahran, 31261 Saudi Arabia

† These authors contributed equally: Woo Hee Kim, Muhammad Israr, You Kyoung Chung

*Corresponding author. Email: hybae@skku.edu (HYB); joonsukhuh@yonsei.ac.kr (JH)

Table of Contents

**1. General Information**	....	**S3**
**2. Reaction Optimization**	....	**S4**
2.1. Solvent study	....	**S4**
2.2. Silane study	....	**S5**
2.3. Equivalent and temperature study	....	**S5**
2.4. Equivalent and catalyst loading study	....	**S6**
2.5. Amine source study	....	**S7**
**3. Synthetic Protocols**	....	**S8**
3.1. General procedure for silylium-ion initiated catalytic reductive amination	....	**S8**
3.2. Procedures for the synthetic transformations	....	**S8**
3.3. Preparation of Trityl tetrakis(3,5-is(trifluoromethyl)phenyl)borate	....	**S12**
**4. Unsuccessful Substrates**	....	**S13**
**5. Time-dependant reaction progress based on catalyst loading**	....	**S14**
**6. Analytical Data of the Products**	....	**S15**
**7. NMR Spectra of the Products**	....	**S38**
**8. NMR experiment**	....	**S133**
**9. Computational Studies on Mechanistic Investigation**	....	**S134**
9.1. Cartesian coordinate of the Figure 4	....	**S134**
9.2. Cartesian coordinate of the Figure 3D and 3E	....	**S194**
9.3. DFT calculations of Mulliken charge on silicon atom	....	**S214**
9.4 Computational study of ketimine-enamine type tautomerization	....	**S214**
**10. HR-MS analytical data for products**	....	**S215**
**11. HPLC spectra of product**	....	**S254**
**12. Calculation of E-factors**	....	**S255**
**13. Supplementary References**	....	**S257**

1. General information

■ Chemical

Chemicals were purchased from commercial suppliers (e.g., Aldrich, Alfa Aesar, TCI) and used without further purification unless otherwise stated. Anhydrous solvents, NMR solvents, and additional organic solvents were purchased from commercial vendors (e.g., Aldrich, Alfa Aesar, CIL Inc., Merck, and Wako) and used without further distillation or purification.

■ Thin-Layer Chromatography (TLC)

TLC results were monitored using silica gel plates (Merck, Kieselgel 60 F254 0.25 mm). Visualization by staining methods (*e.g.*, Ninhydrin and phosphomolybdic acid upon heating) was performed when if it was needed.

■ Column chromatographic purification

Purification was carried out using silica gel (Merck, 60 Å, 230–400 mesh, 0.040–0.063 mm).

■ Nuclear magnetic resonance (NMR) spectroscopy

^{1}H NMR (500 MHz, 700 MHz), ^{13}C NMR (126 MHz, 175 MHz), and ^{19}F NMR (470 MHz) spectra were analyzed and obtained by using Bruker Ascend™ 500 and Avance™ III 700 spectrometer at 25 °C. ^{1}H (600 MHz), ^{13}C (151 MHz), ^{19}F (565 MHz), and ^{31}P (243 MHz) NMR spectra were recorded using Bruker Advance Neo 600 spectrometer (NFEC-2026-02-312992) at the Chiral Material Core Facility Center of Sungkyunkwan University. The chemical shifts (δ) were internally referenced to tetramethylsilane (TMS δ = 0.00, ^{1}H NMR) and Chloroform-*d* ($CDCl_3$ δ = 77.160, ^{13}C NMR) for ^{1}H and ^{13}C NMR spectroscopy. Integration data are represented as follows: coupling constant (J = Hz) and multiplicity (s = singlet, bs = broad singlet, d = doublet, dd = double of doublets, ddd = doublet of doublets of doublets, t = triplet, q = quartet, m = multiplet).

■ Mass spectroscopy (MS)

High-resolution mass spectra were analyzed by using a Supercritical Fluid Chromatograph combined with Xevo G2-XS QTOF Mass Spectrometer (Waters, Milford, MA, USA) at the Chiral Material Core Facility Center of Sungkyunkwan University. Analysis was conducted by SFC-MS/MS using a Shimadzu Nexera UC/LCMS-8060RX triple quadrupole system (NFEC-2026-01-311930) at the Chiral Material Core Facility Center of Sungkyunkwan University.

■ Melting point determination (mp)

Melting point of products was determined using Büchi® M-560.

2. Reaction optimization

2.1. Solvent study

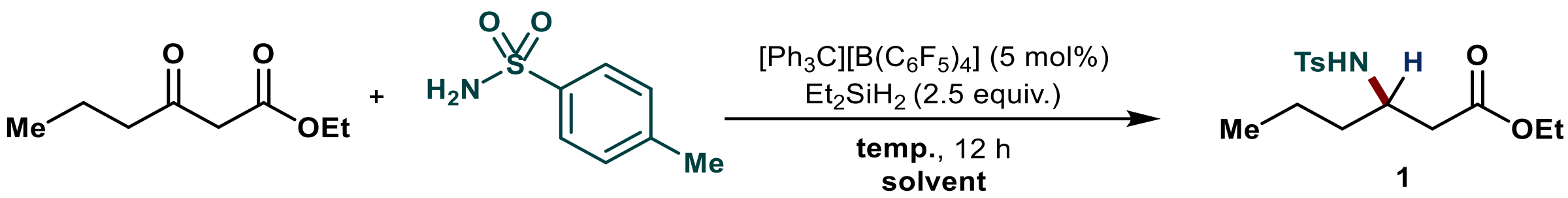

entry[a]	solvent	temp. (°C)	conv. (%)[b][c]
1	$MeNO_2$	60	37
2	CH_2Cl_2	60	46
3	$CHCl_3$	60	trace
4	MeOH	60	n.d.[e]
5	THF	60	n.d.
6	DMF	60	n.d.
7	MeCN	60	n.d.
8	PhMe	60	47
9	PhCl	60	45
10	1,2-Chlorobenzene	60	35
11	*o*-Xylene	60	25
12	Acetone	60	6
13	1,4-Dioxane	60	trace
14	HFIP	60	trace
15	C_6D_6	80	18
16	$CDCl_3$	80	11
17	neat	r.t.	60
18	neat	60	78
19	neat	80	90 (85)

[a] Reactions were performed with Ethyl 3-oxohexanoate (0.2 mmol, 1.0 equiv.), *p*-toluenesulfonamide (0.4 mmol 2 equiv.), Et_2SiH_2 (0.5 mmol, 2.5 equiv.), and catalyst (0.01 mmol, 5 mol%) in solvent (0.2 M, 1 mL). [b] Yield (%) were determined by ^1H NMR analysis using 1,3,5-trimethoxybenzene as an internal standard. [c] Isolated yield was indicated in parenthesis. [e] n.d. = not detected.

2.2. Silane screening

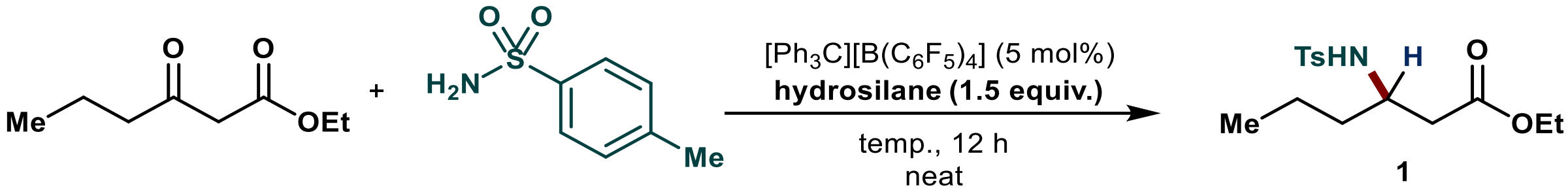

entry[a]	silane	temp. (°C)	conv. (%)[b]
1	Polymethylhydrosiloxane	80	34
2	$PhMe_2SiH$	80	39
3	$EtMe_2SiH$	80	25
4	$(EtO)_3SiH$	80	trace
5	Ph_3SiH	80	44
6	Ph_2SiH_2	80	56
7	Et_3SiH	80	38
8	Et_2SiH_2	80	90 (85)

[a] Reactions were performed with Ethyl 3-oxohexanoate (0.2 mmol, 1.0 equiv.), *p*-toluenesulfonamide (0.4 mmol 2 equiv.), silane (0.5 mmol, 2.5 equiv.), and catalyst (0.01 mmol, 5 mol%) in solvent (0.2 M, 1 mL). [b] Yield (%) were determined by ^{1}H NMR analysis using 1,3,5-trimethoxybenzene as an internal standard.

2.3. Equivalent and temperature study

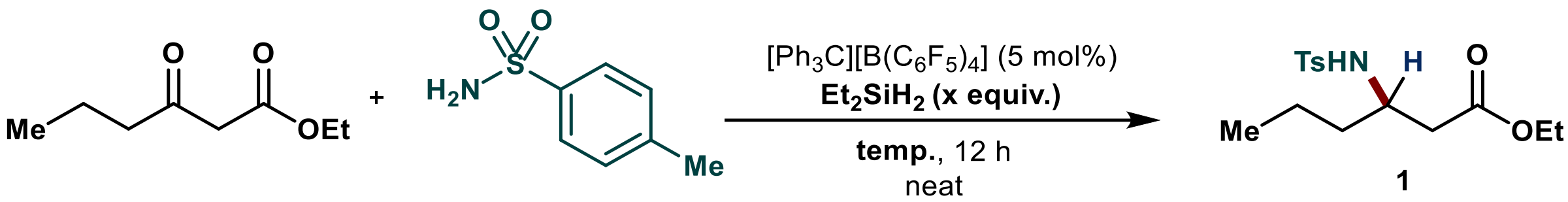

entry[a]	silane (x equiv.)	amide (y equiv.)	temp. (°C)	conv. (%)[b][c]
1	1.5	1.5	r.t.	60
2	1.5	1.5	60	73
3	2	1.5	60	84
4	2	1.5	80	85
5	2	2	60	84
6	2	2	80	86
7	2.5	1.5	80	84
8	2.5	2	60	83
9	2.5	2	80	90 (85)
10	3	2	60	83
11	3	2	80	89

[a] Reactions were performed with Ethyl 3-oxohexanoate (0.2 mmol, 1.0 equiv.), *p*-toluenesulfonamide (y mmol y equiv.), Et_2SiH_2 (x mmol, x equiv.), and catalyst (0.01 mmol, 5 mol%) in solvent (0.2 M, 1 mL). [b] Yield (%) were determined by ^{1}H NMR analysis using 1,3,5-trimethoxy benzene as an internal standard. [c] Isolated yield was indicated in parenthesis.

2.4. Equivalent and catalyst loading study

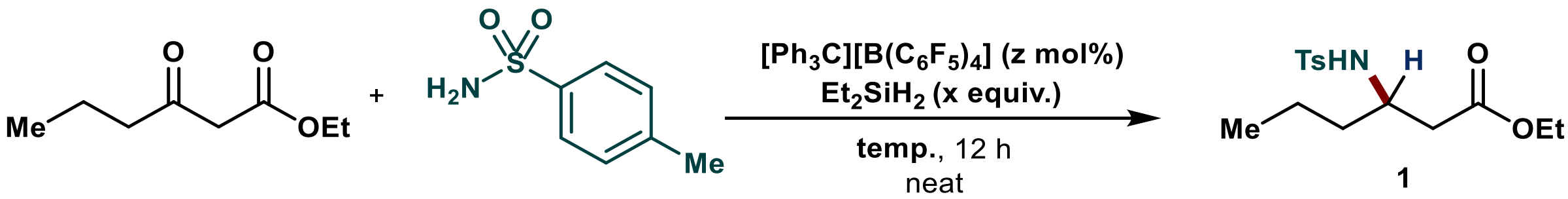

entry[a]	silane (x equiv.)	amide (y equiv.)	catalyst (z mol%)	temp. (°C)	conv. (%)[b][c]
1	1.5	1.5	5	r.t.	60
2	1.5	1.5	5	60	73
3	2	1.5	5	60	84
4	2	1.5	7	60	84
5	2	1.5	5	80	85
6	2	1.5	7	80	86
7	2	2	5	60	84
8	2.5	2	5	80	90 (85)
9	2.5	2	3	80	68
10	2.5	2	4	80	82
11	2.5	2	7	80	89
12	2.5	2	10	80	90

[a] Reactions were performed with Ethyl 3-oxohexanoate (0.2 mmol, 1.0 equiv.), *p*-toluenesulfonamide (y mmol y equiv.), Et_2SiH_2 (x mmol, x equiv.), and catalyst (0.01 mmol, 5 mol%) in solvent (0.2 M, 1 mL). [b] Yields were determined by ^1H NMR analysis using 1,3,5-trimethoxy benzene as an internal standard. [c] Isolated yield was indicated in parenthesis.

2.5. Amine source study

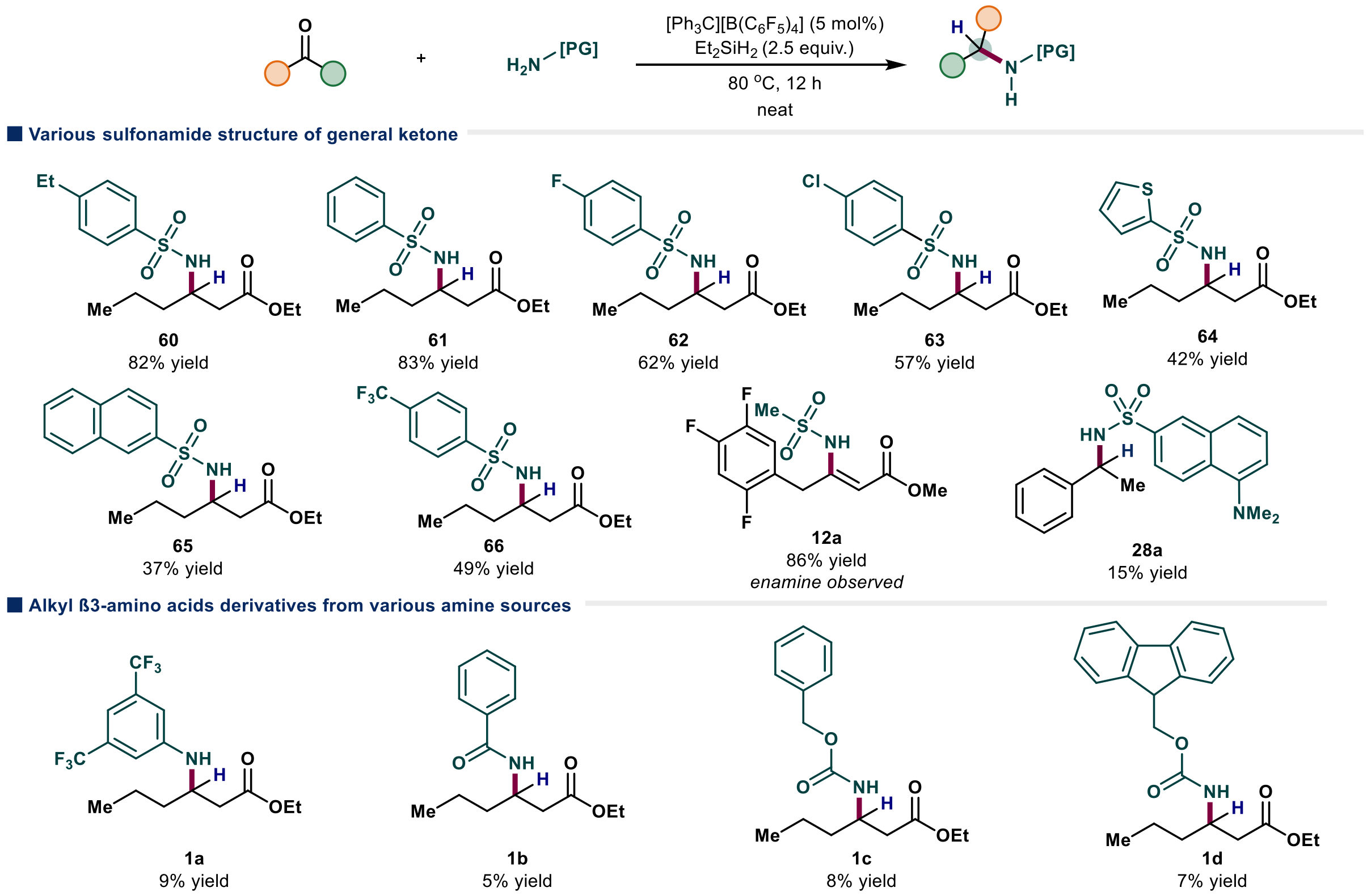

Reaction was performed with ketone (0.2 mmol, 1 equiv.), *p*-toluenesulfonamide (0.4 mmol, 2 equiv.), Et_2SiH_2 (0.5 mmol, 2.5 equiv.), and $[Ph_3C]^+[B(C_6F_5)_4]^-$ (0.01 mmol, 5 mol%) at 80°C. The yield was determined after purification by column chromatography.

3. Synthetic protocols

3.1. General procedure for silylium-ion initiated catalytic reductive amination

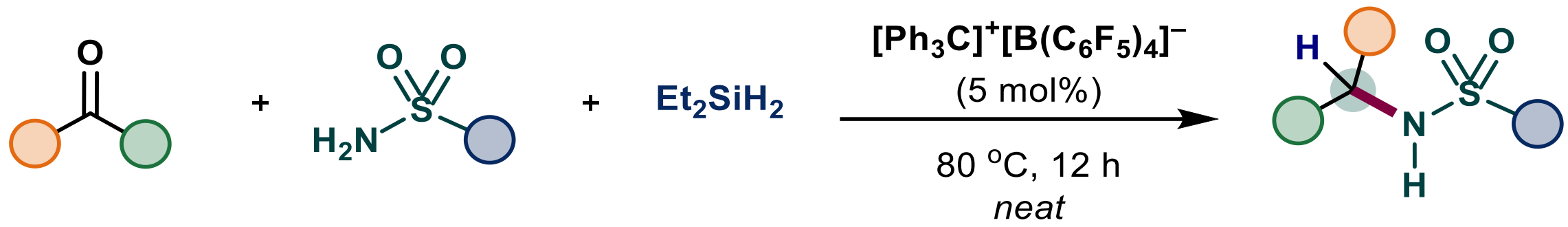

A flame dried 5.0 mL vial was charged, under Ar atmosphere, ketone (0.2 mmol, 1.0 equiv.), *p* -toluenesulfonamide (68.4 mg, 0.4 mmol, 2 equiv.) and $[Ph_3C]^+[B(C_6F_5)_4]^-$ (9.22 mg 0.01 mmol, 5 mol%) were added. Subsequently, Et_2SiH_2 (65 µL, 0.5 mmol, 2.5 equiv.) was added. The reaction mixture was then stirred at 80 °C for 12 h. After the completion of reaction, the residue was purified by column chromatography to afford the desired product.

*** Scale-up experiment (gram scale)**

To a flame-dried round bottom flask, equipped with a magnetic stirring bar, back filled with Ar gas, ketone (1.0 equiv.), *p*-toluenesulfonamide (2 equiv.) and $[Ph_3C]^+[B(C_6F_5)_4]^-$ (5 mol%) were added respectively. Then, Et_2SiH_2 (2.5 equiv.) was added dropwise via syringe. The reaction mixture was then stirred at 80 °C for 12 h in oil bath. After the completion of reaction, the residue was purified by column chromatography to afford the desired product.

3.2. Procedures for the synthetic transformations

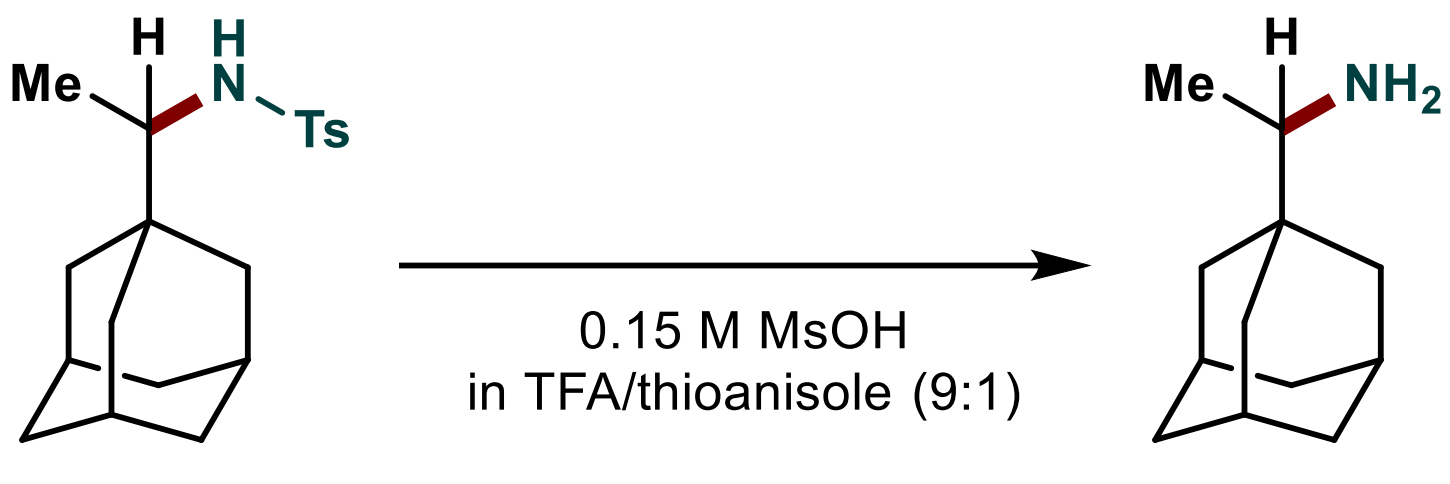

Reaction was carried out according to a literature procedure:[1] In a flame-dried valved Schlenk tube, equipped with a magnetic stirring bar and filled with Ar gas, **26** (64.4 mg, 0.19 mmol, 1 equiv.) was treated with a 0.15 M solution of methanesulfonic acid in TFA/thioanisole (9:1, 2.5 mL) at r.t., and the solution was stirred for 24 h. After removal volatiles under reduced pressure, the resulting mixture was diluted and extracted with CH_2Cl_2/brine, and the combined organic layer was dried over anhydrous Na_2SO_4 and filtered. The resulting mixture was concentrated *in vacuo*, and the residue was purified by column chromatography on silica gel (MeOH:CH_2Cl_2 = 1:9 v/v) to afford desired product (24.1 mg, 71% yield) as a yellowish oil.

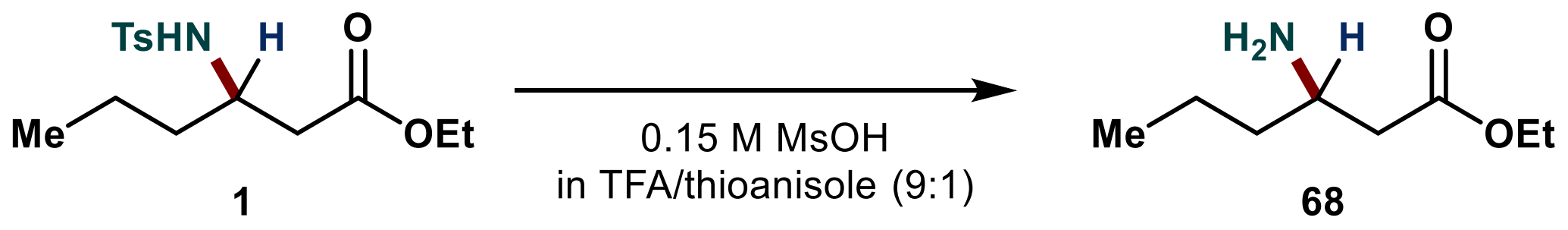

Reaction was carried out according to a literature procedure:[1] In a flame-dried valved Schlenk tube, equipped with a magnetic stirring bar and filled with Ar gas, **1** (98 mg, 0.31 mmol, 1 equiv.) was treated with a 0.15 M solution of methanesulfonic acid in TFA/thioanisole (9:1, 4 mL) at r.t., and the solution was stirred for 24 h. After removal volatiles under reduced pressure, the resulting mixture was diluted and extracted with CH_2Cl_2/brine, and the combined organic layer was dried over anhydrous Na_2SO_4 and filtered. The resulting mixture was concentrated *in vacuo*, and the residue was purified by column chromatography on silica gel (MeOH:CH_2Cl_2 = 1:9 v/v) to afford β-amino ester (50 mg, 84% yield) as a yellowish oil.

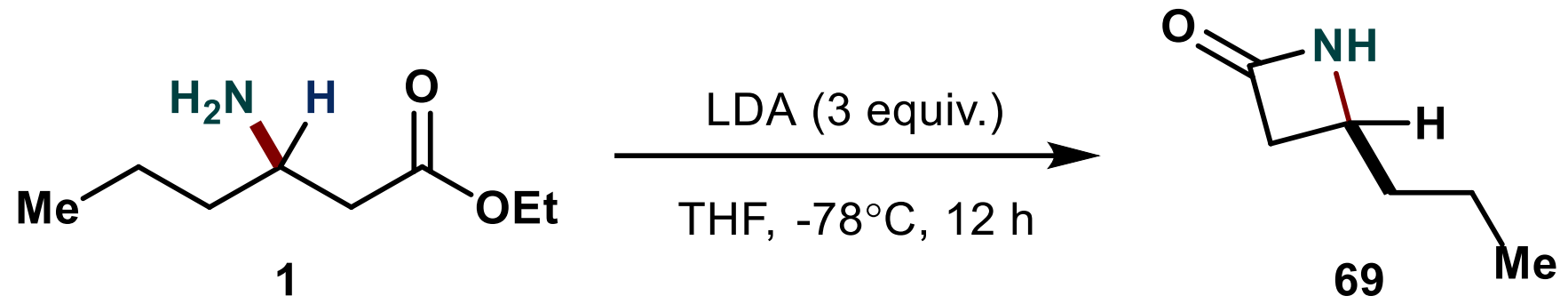

Reaction was carried out according to a literature procedure:[2] In a flame-dried valved Schlenk tube, equipped with a magnetic stirring bar and filled with Ar gas, *n*-BuLi (1.6 M solution in hexane, 1.85 mL, 3 mmol, 3 equiv.) was added at 0°C to a THF solution (10 mL) of diisopropylamine (303 mg, 3 mmol, 3 equiv.). After stirred for 10 min, the mixture was cooled to -78°C. **1** was added and the mixture was stirred for 12 h at the same temperature. H_2O was added to quench the reaction, and the reaction mixture was diluted and extracted with ethyl acetate/brine, and the combined organic layer was dried over anhydrous Na_2SO_4 and filtered. The resulting mixture was concentrated *in vacuo*, and the residue was purified by column chromatography on silica gel (MeOH:CH_2Cl_2 = 1:9 v/v) to afford β-lactam (50 mg, 54% yield) as a yellowish oil.

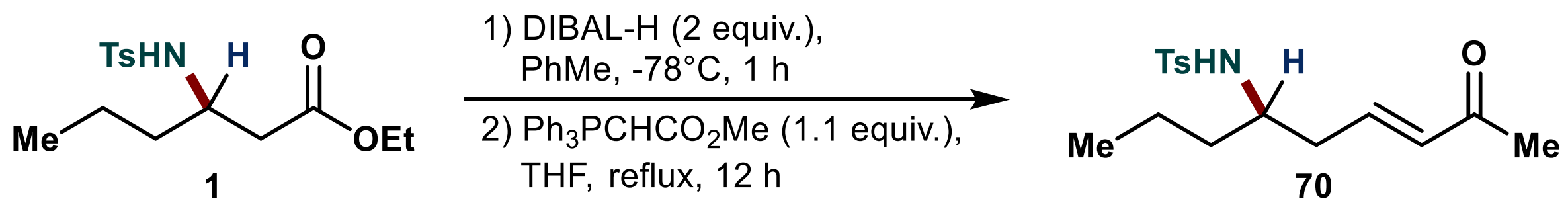

Reaction was carried out according to a literature procedure:[3] In a flame-dried valved Schlenk tube, equipped with a magnetic stirring bar and filled with Ar gas, DIBAL-H (1 M solution in THF, 1 mL, 1 mmol, 2 equiv.) was added dropwise to a solution of **1** (156 mg, 0.5 mmol, 1 equiv.) in PhMe (5 mL) at -78°C and reaction mixture was stirred at -78°C for 1h. Subsequently, the crude mixture was quenched with MeOH (5 mL) and allowed to warm to room temperature. The reaction mixture was concentrated *in vacuo*, which was used in the next step without further purification. To a solution of prepared crude mixture in THF (5 mL), Ph_3PCHCO_2Me (175 mg, 0.55 mmol, 1.1 equiv.) was added and stirred at reflux for 12 h. The resulting mixture was concentrated *in vacuo*, and the residue was purified by column chromatography on silica gel (EtOAc:hexanes = 1:49 to 1:9 v/v) to afford α,β-unsaturated-γ-amino ketone (122 mg, 79% yield) as a yellowish oil.

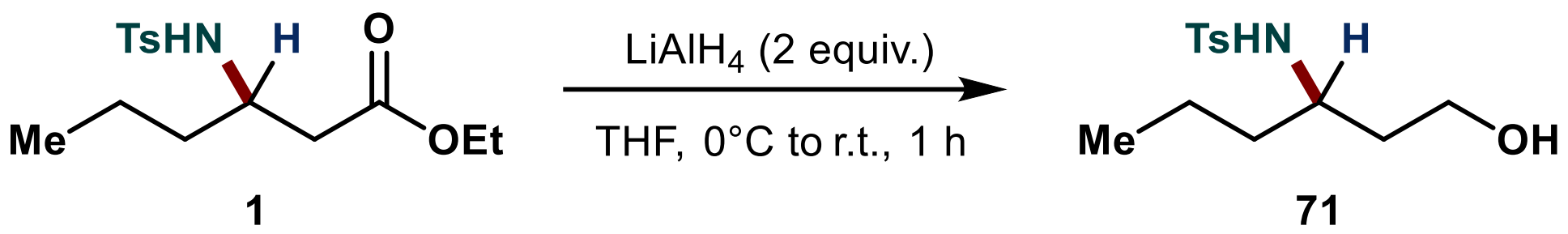

Reaction was carried out according to a literature procedure:[3] In a flame-dried valved Schlenk tube, equipped with a magnetic stirring bar and filled with Ar gas, a suspension of lithium aluminium hydride (37.9 mg, 1 mmol, 2 equiv.) in dry THF (1 mL) was added a solution of **1** (156 mg, 0.5 mmol, 1 equiv.) in dry THF (2 mL) at 0 °C and the mixture was stirred at r.t. for 2 h. Then it was quenched with ethyl acetate (1 mL) at 0 °C. The resulting mixture was diluted and extracted with ethyl acetate/brine, and the combined organic layer was dried over anhydrous Na_2SO_4 and filtered. The filtrate was concentrated *in vacuo*, and the residue was purified by column chromatography on silica gel (EtOAc:hexanes = 1:49 to 1:9 v/v) to afford γ-amino alcohol (131.6 mg, 97% yield) as a yellowish oil.

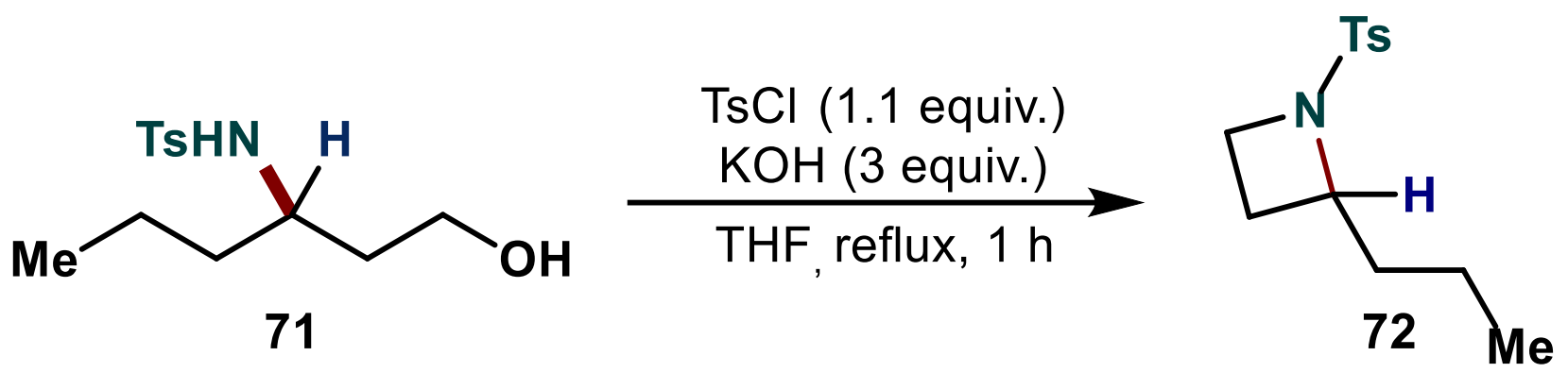

Reaction was carried out according to a literature procedure:[4] To a solution of powdered KOH (37 mg, 0.66 mmol, 3 equiv.) and **71** (131.6 mg, 0.22 mmol, 1 equiv.) in dry THF (1.1 mL), TsCl (46.1 mg, 0.242 mmol, 1.1 equiv.) was added dropwise at r.t. and the reaction mixture was refluxed for 1 h. After completion of the reaction, cold water was added and the reaction mixture was diluted and extracted with ethyl acetate/brine, and the combined organic layer was dried over anhydrous Na_2SO_4 and filtered. The filtrate was concentrated *in vacuo*, and the residue was purified by column chromatography on silica gel (EtOAc:hexanes = 1:49 to 1:9 v/v) to afford azetidine (55 mg, 99% yield) as a yellowish oil.

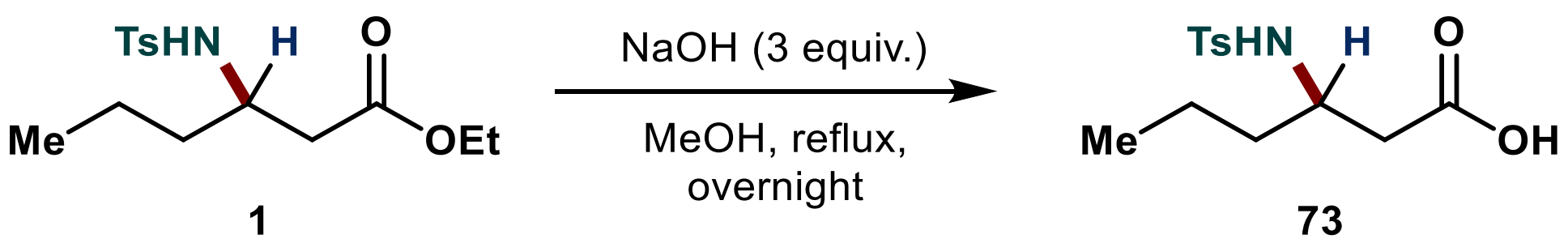

Reaction was carried out according to a literature procedure:[5] In a flame-dried valved Schlenk tube, equipped with a magnetic stirring bar and filled with Ar gas, compound **1** (313 mg, 1 mmol, 1 equiv.) and NaOH (120 mg, 3 mmol, 3 equiv.) were added in MeOH (10 mL). The mixture was stirred at reflux for overnight. After the completion of reaction, the solvent was concentrated *in vacuo*, and CH_2Cl_2 and water were added. The resulting solution was then acidified with 1 M HCl solution and extracted with CH_2Cl_2. The combined organic layer was washed with brine, dried with sodium sulfate, and concentrated *in vacuo*. The residue was purified by column chromatography (EtOAc:hexanes = 1:1 v/v) to afford the hydrolysis product (285 mg, 99% yield) as a white solid.

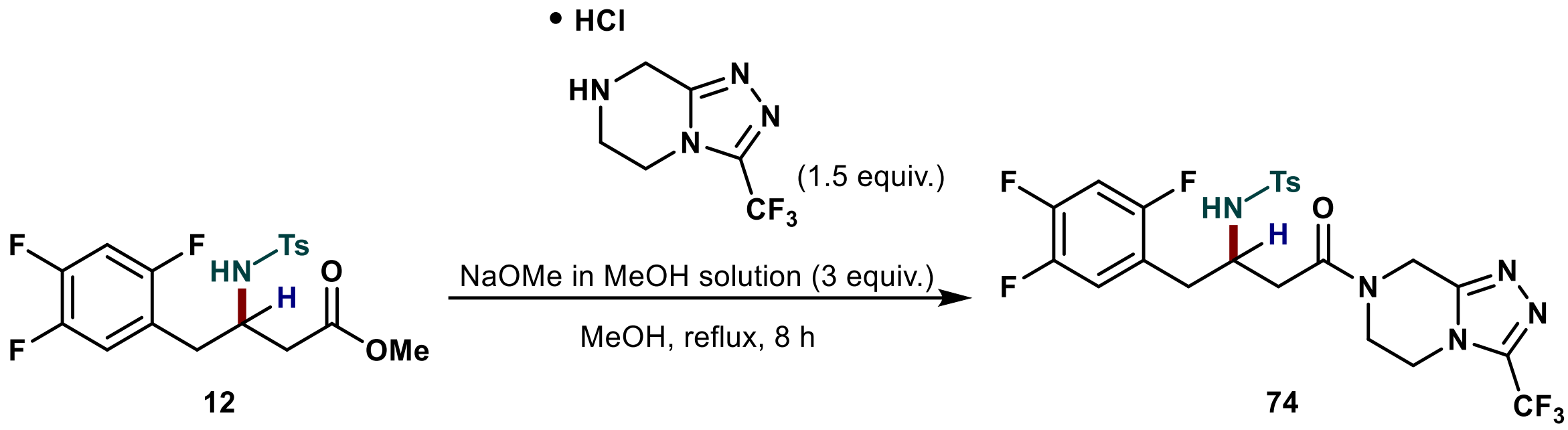

Reaction was carried out according to a literature procedure:[6] To a flame-dried round bottom flask, equipped with a magnetic stirring bar, back filled with Ar gas, **12** (401 mg, 1 mmol, 1 equiv.) and 3-(trifluoromethyl)-5,6,7,8-tetrahydro-[1,2,4]triazolo[4,3-a]pyrazine hydrochloride (274 mg, 1.2 mmol, 1.2 equiv.) were added in anhydrous MeOH (10 mL). Then a 25 wt % solution of NaOMe in MeOH (0.686 mL, 1.2 mmol, 3 equiv.) was added dropwise at r.t. and the reaction mixture was refluxed for 8 h. After completion of the reaction, the reaction mixture was allowed to cool to r.t. and concentrated in vacuo to remove the volatile, then acidified to pH 2 using a 1 M HCl solution. The aqueous phase was extracted twice with EtOAc, and the combined organic layers were washed with brine, dried over anhydrous Na_2SO_4. The resulting mixture was concentrated *in vacuo*, and the residue was purified by column chromatography on silica gel (EtOAc:hexanes = 1:1 to 9:1 v/v) to afford β-amino ester (443 mg, 79% yield) as a pale gray solid.

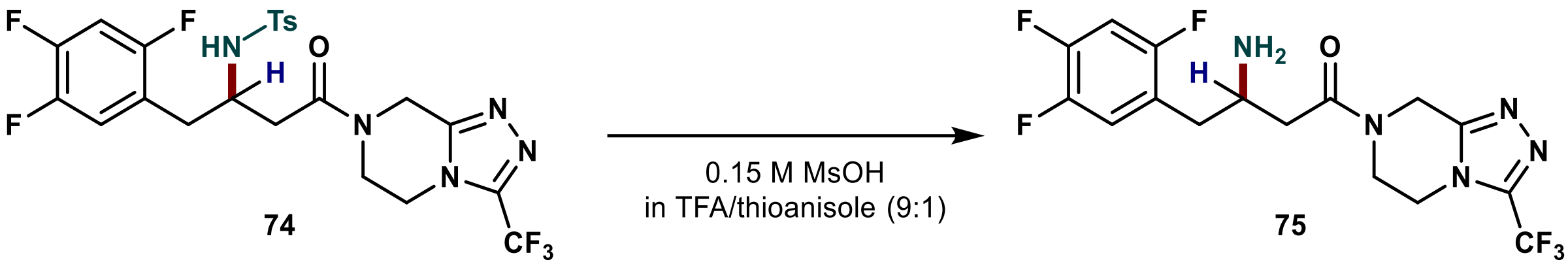

Reaction was carried out according to a literature procedure: [1] In a flame-dried valved Schlenk tube, equipped with a magnetic stirring bar and filled with Ar gas, **74** (56 mg, 0.1 mmol, 1 equiv.) was treated with a 0.15 M solution of methanesulfonic acid in TFA/thioanisole (9:1, 2.6 mL) at r.t., and the solution was stirred for 24 h. After removal volatiles under reduced pressure, the resulting mixture was diluted and extracted with CH_2Cl_2/brine, and the combined organic layer was dried over anhydrous Na_2SO_4 and filtered. The resulting mixture was concentrated *in vacuo*, and the residue was purified by column chromatography on silica gel (MeOH:CH_2Cl_2 = 1:9 v/v) to afford desired product (34.2 mg, 84% yield) as a colorless gummy solid.
※ Please note that H_2 evolution may occur during catalytic reductive sulfonamidation in scale up synthesis.

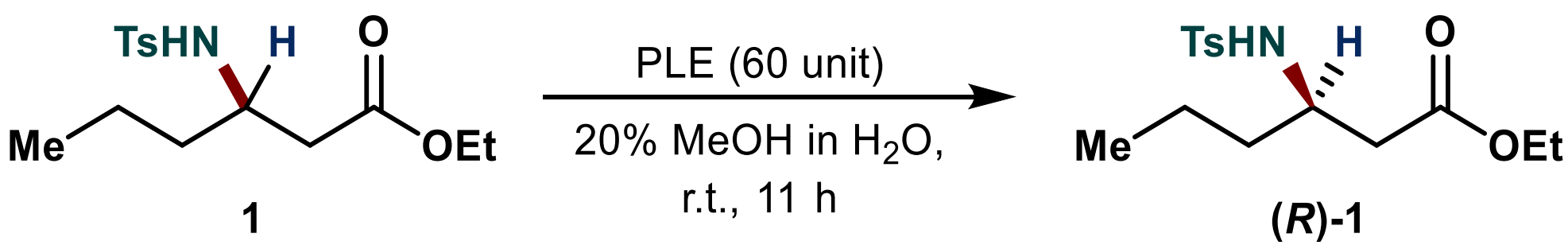

1 was dissolved in methanol (0.6 mL) followed by the addition of DI water (2.4 mL). To this mixture 4 mg (60 units, 1 mg = 15 units) of Pig liver Esterase (PLE) was added and kept stirring at room

temperature for 10 hours. After the reaction was completed, 1 M HCl was added to the reaction mixture to adjust the solution to pH 3. The aqueous layer was extracted with EtOAc, dried over Na_2SO_4, filtered and evaporated. The residue was purified by column chromatography (EtOAc:hexanes = 1:4 v/v) to afford ***(R)*-1**. $[\alpha]^{20}_D$ +4.9 (*c* = 0.25, $CHCl_3$)

3.3. Preparation of Trityl tetrakis(3,5-bis(trifluoromethyl)phenyl)borate

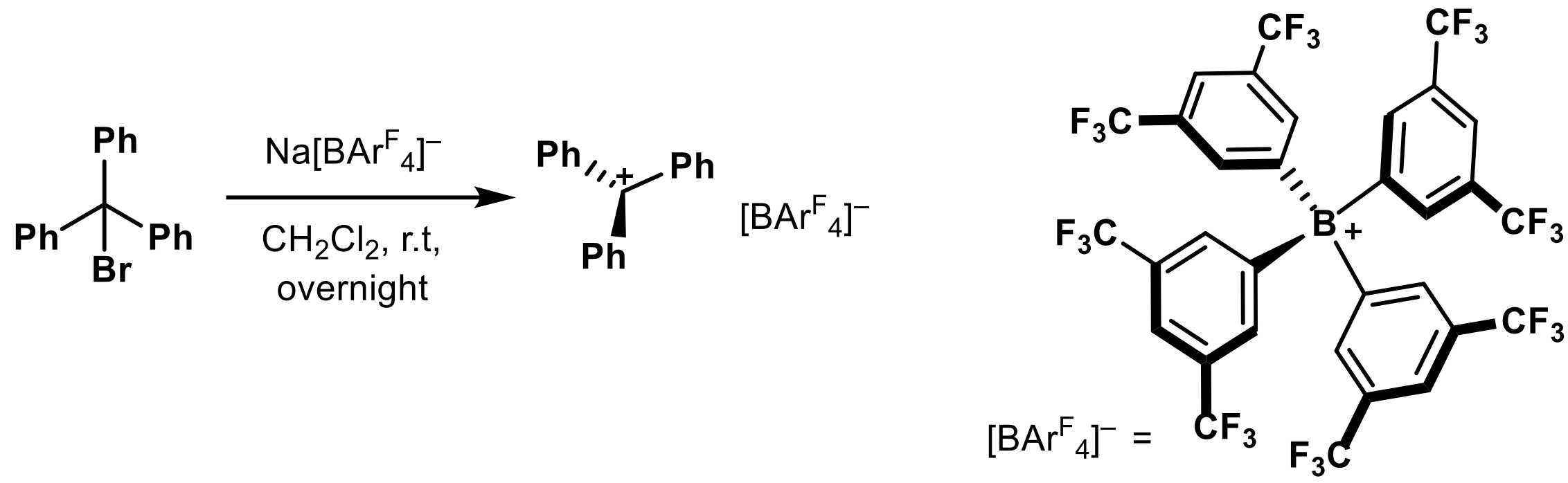

Reaction was carried out according to a literature procedure:[7] To a flame-dried round bottom flask, equipped with a magnetic stirring bar, back filled with Ar gas, trityl bromide (886 mg, 1 mmol, 1 equiv.) and sodium tetrakis(3,5- bis(trifluoromethyl)phenyl)borate (278 mg, 1 mmol, 1 equiv.) were added in anhydrous CH_2Cl_2 (14 mL) and stirred overnight at room temperature. Then, reaction mixture was filtered through Celite, and the filtrate was evaporated under reduced pressure. The resulting solid was washed with *n*-pentane and then purified by recrystallization with CH_2Cl_2/*n*-pentane to afford bright yellow solid (973 mg, 88% yield).

^{1}H NMR (500 MHz, $CDCl_3$): δ 8.14 (t, *J* = 7.5 Hz, 3H), 7.75 (t, *J* = 7.8 Hz, 6H), 7.68 (s, 8H), 7.57 (d, *J* = 7.5 Hz, 6H), 7.48 (s, 4H).

^{13}C NMR (126 MHz, $CDCl_3$): δ 210.9, 161.8 (dd, $J_{C\text{-}F}$ = 99.9 Hz, 49.6 Hz), 143.9, 142.5, 139.9, 134.9, 130.8, 129.1 (d, $J_{C\text{-}F}$ = 34.1 Hz), 124.6 (q, $J_{C\text{-}F}$ = 272.5 Hz), 117.6.

^{19}F NMR (471 MHz, $CDCl_3$): δ -62.41.

4. Unsuccessful Substrates

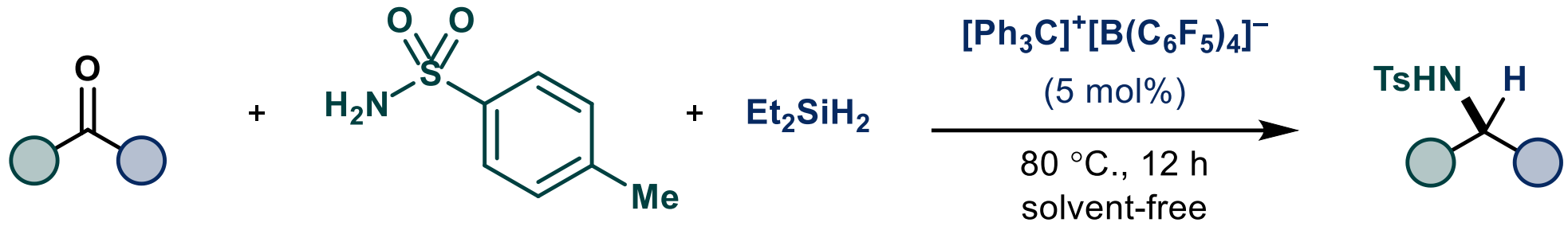

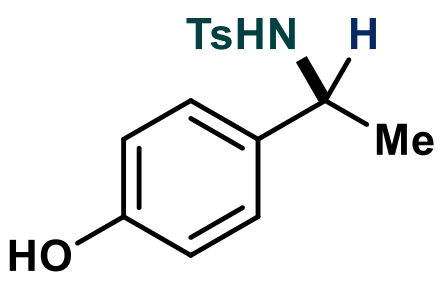

n.r

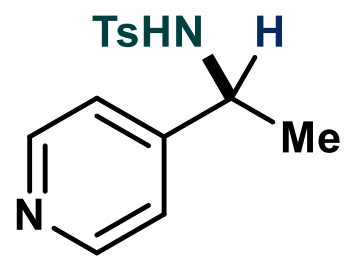

n.r

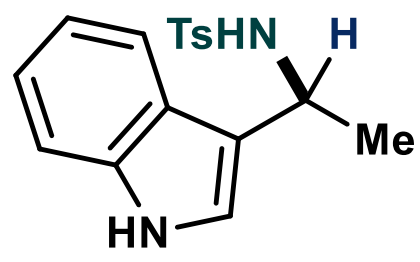

n.r

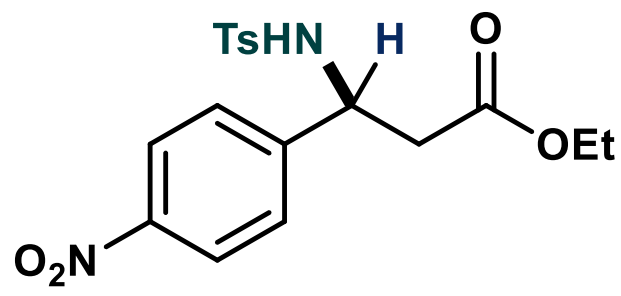

decomposed
(1 mol% cat.)

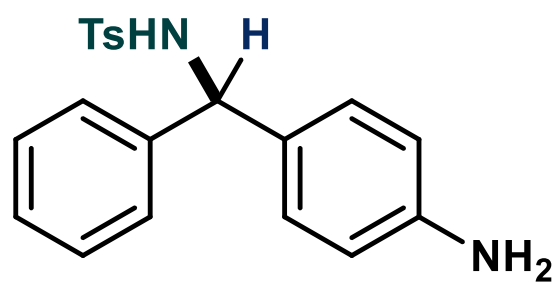

n.r

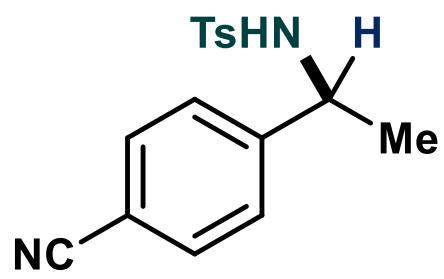

n.r

5. Time-dependant reaction progress based on catalyst loading

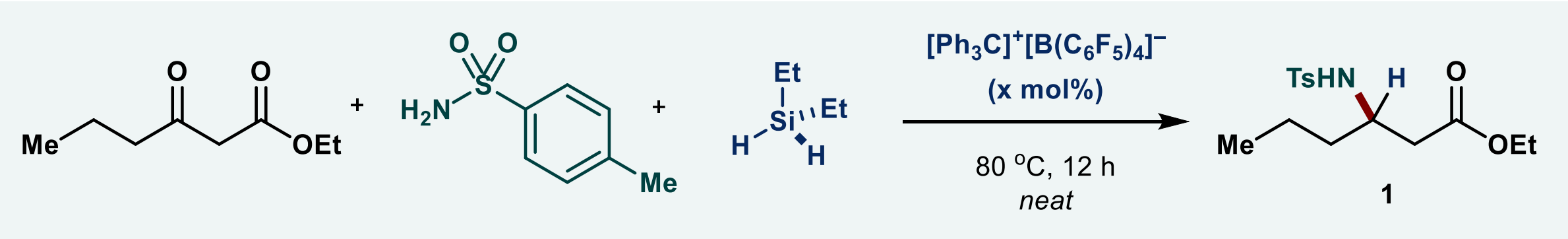

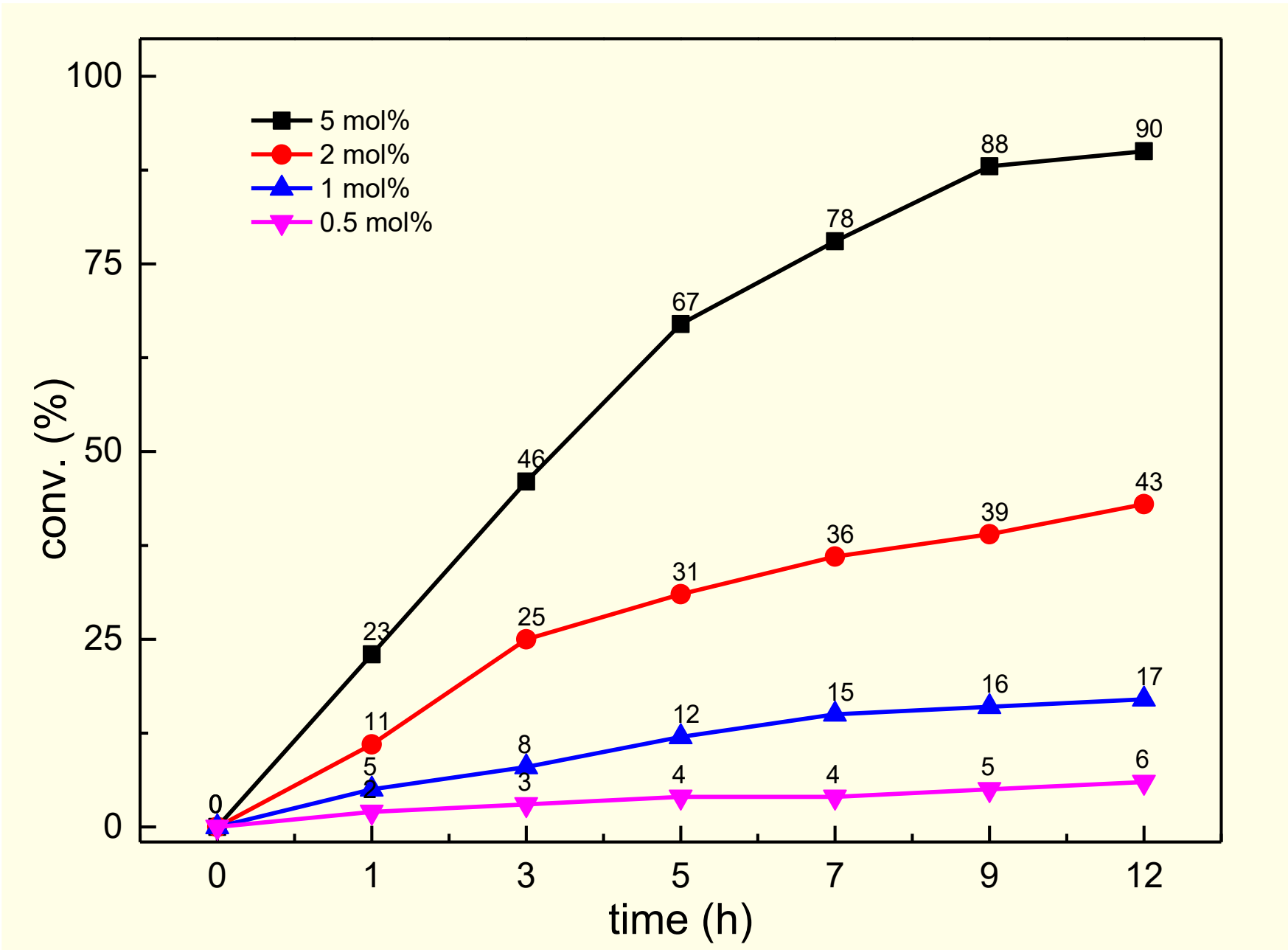

6. Analytical Data of the Products

Ethyl 3-((4-methylphenyl)sulfonamido)hexanoate (1)

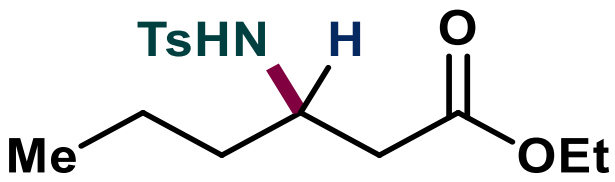

New compound, colorless oil, 53.3 mg, 85% yield.
Purification conditions for column chromatography: EtOAc:hexanes 1:49 to 1:9
R_f = 0.35 (EtOAc:hexanes = 1:4 v/v).
^{1}H NMR (500 MHz, $CDCl_3$): δ 7.75 (d, *J* = 8.1 Hz, 2H), 7.29 (t, *J* = 7.8 Hz, 2H), 5.25 (d, *J* = 9.0 Hz, 1H), 4.11 – 4.01 (m, 2H), 3.52 (qd, *J* = 10.9, 5.6 Hz, 1H), 2.41 (s, 3H), 2.38 – 2.31 (m, 2H), 1.47 – 1.37 (m, 2H), 1.32 – 1.26 (m, 2H), 1.21 (t, *J* = 7.1 Hz, 3H), 0.78 (t, *J* = 7.3 Hz, 3H).
^{13}C NMR (126 MHz, $CDCl_3$): δ 171.5, 143.4, 138.3, 129.8, 127.2, 60.8, 50.6, 38.8, 36.9, 21.6, 19.1, 14.2, 13.7.
HR-MS: m/z calcd. $[C_{15}H_{23}NO_4SNa]^+$: 336.1245; found (ESI): 336.1242.

Ethyl 3-((4-methylphenyl)sulfonamido)pentanoate (2)

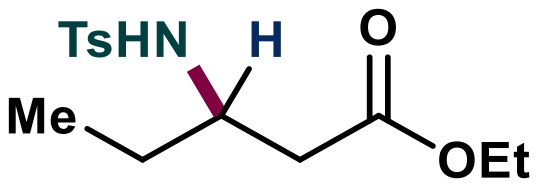

New compound, colorless oil, 54.5 mg, 91% yield.
Purification conditions for column chromatography: EtOAc:hexanes 1:49 to 1:9
R_f = 0.53 (EtOAc:hexanes = 1:4 v/v)
^{1}H NMR (500 MHz, $CDCl_3$): δ 7.75 (d, *J* = 8.3 Hz, 2H), 7.27 (d, *J* = 8.3 Hz, 2H), 5.31 (d, *J* = 8.9 Hz, 1H), 4.08 – 4.01 (m, 2H), 3.48 – 3.41 (m, 1H), 2.41 (s, 3H), 2.40 (dd, *J* = 15.9 Hz, 4.9 Hz, 1H), 2.33 (dd, *J* = 16.0 Hz, 5.8 Hz, 1H), 1.48 (p, *J* = 7.3 Hz, 2H), 1.20 (t, *J* = 7.1 Hz, 2H), 0.79 (d, *J* = 7.4 Hz, 2H).
^{13}C NMR (126 MHz, $CDCl_3$): δ 171.5, 143.4, 138.3, 129.7, 127.1, 60.8, 52.3, 38.5, 27.8, 21.6, 14.2, 10.4.
HR-MS: m/z calcd. $[C_{14}H_{21}NO_4SNa]^+$: 322.1089; found (ESI): 322.1090.

Ethyl 3-((4-methylphenyl)sulfonamido)butanoate (3)

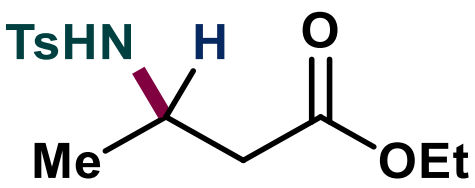

Known compound, see Ref. 8. 47.4 mg, 83% yield.
Purification conditions for column chromatography: EtOAc:hexanes 1:49 to 1:9
^{1}H NMR (500 MHz, $CDCl_3$): δ 7.75 (d, *J* = 8.2 Hz, 2H), 7.28 (d, *J* = 8.1 Hz, 2H), 5.31 (d, *J* = 8.4 Hz, 1H), 4.11 – 4.01 (m, 2H), 3.71 –3.63 (m, 1H), 2.44 – 2.36 (m, 2H), 2.41 (s, 3H), 1.20 (t, *J* = 7.1 Hz, 3H), 1.12 (d, *J* = 6.7 Hz, 3H).
^{13}C NMR (126 MHz, $CDCl_3$): δ 171.3, 143.4, 138.1, 129.8, 127.1, 60.8, 46.7, 40.9, 21.6, 21.1, 14.2.

Ethyl 4-methyl-3-((4-methylphenyl)sulfonamido)pentanoate (4)

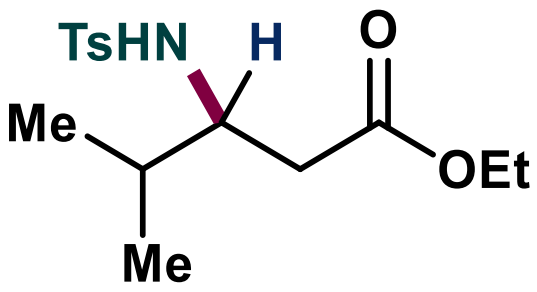

New compound, colorless oil, 59.5 mg, 95% yield.
Purification conditions for column chromatography: EtOAc:hexanes 1:49 to 1:9
R_f = 0.53 (EtOAc:hexanes = 1:4 v/v)
^{1}H NMR (500 MHz, $CDCl_3$): δ 7.75 (d, *J* = 8.3 Hz, 2H), 7.28 (d, *J* = 8.2 Hz, 2H), 5.26 – 5.23 (m, 1H), 4.08 – 3.97 (m, 2H), 3.35 – 3.30 (m, 1H), 2.41 (s, 3H), 2.37 (d, *J* = 5.1 Hz, 1H), 2.28 (dd, *J* = 15.8 Hz, 5.7 Hz, 1H), 1.80 (dq, *J* = 13.5 Hz, 6.7 Hz, 1H), 1.20 (t, *J* = 7.1 Hz, 3H), 0.84 (d, *J* = 6.8 Hz, 3H), 0.81 (d, *J* = 6.8 Hz, 3H).
^{13}C NMR (126 MHz, $CDCl_3$): δ 171.7, 143.3, 138.2, 129.7, 127.2, 60.8, 56.4, 36.4, 31.8, 21.6, 19.0, 18.6, 14.2.
HR-MS: m/z calcd. $[C_{15}H_{23}NO_4SNa]^+$: 336.1245; found (ESI): 336.1244.

Methyl 3-((4-methylphenyl)sulfonamido)butanoate (5)

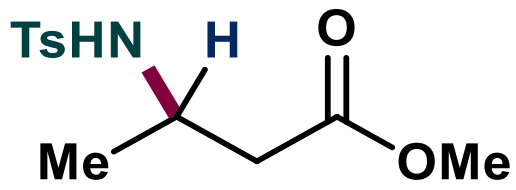

Known compound, see Ref. 8. 43.4 mg, 80% yield.
Purification conditions for column chromatography: EtOAc:hexanes 1:49 to 1:9
^{1}H NMR (500 MHz, $CDCl_3$): δ 7.77 (d, *J* = 8.2 Hz, 2H), 7.30 (d, *J* = 8.0 Hz, 2H), 5.42 (d, *J* = 8.4 Hz, 1H), 3.72 – 3.67 (m, 1H), 3.61 (s, 3H), 2.50 – 2.39 (m, 2H), 2.42 (s, 3H), 1.13 (d, *J* = 6.7 Hz, 3H).
^{13}C NMR (126 MHz, $CDCl_3$): δ 171.6, 143.4, 138.0, 129.8, 127.1, 51.8, 46.7, 40.9, 21.6, 21.1.

Isobutyl 3-((4-methylphenyl)sulfonamido)butanoate (6)

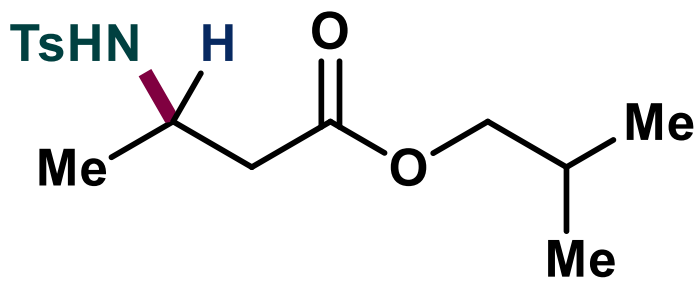

New compound, colorless oil, 49.5 mg, 79% yield.
Purification conditions for column chromatography: EtOAc:hexanes 1:49 to 1:9
R_f = 0.40 (EtOAc:hexanes = 1:4 v/v)
^{1}H NMR (500 MHz, $CDCl_3$): δ 7.75 (d, *J* = 8.2 Hz, 2H), 7.28 (d, *J* = 8.0 Hz, 2H), 5.30 (d, *J* = 8.4 Hz, 1H), 3.79 (qd, *J* = 10.6 Hz, 6.7 Hz, 1H), 3.71 – 3.64 (m, 1H), 2.46 – 2.41 (m, 1H), 2.40 (s, 3H), 2.40 – 2.37 (m, 1H), 1.90 – 1.82 (m, 1H), 1.13 (d, *J* = 6.7 Hz, 3H), 0.89 (d, *J* = 6.7 Hz, 6H).
^{13}C NMR (126 MHz, $CDCl_3$): δ 171.4, 143.4, 138.1, 129.8, 127.2, 70.9, 46.7, 40.8, 27.7, 21.6, 21.2, 19.1.
HR-MS: m/z calcd. $[C_{15}H_{23}NO_4SNa]^+$: 336.1245; found (ESI): 336.1240.

Benzyl 3-((4-methylphenyl)sulfonamido)butanoate (7)

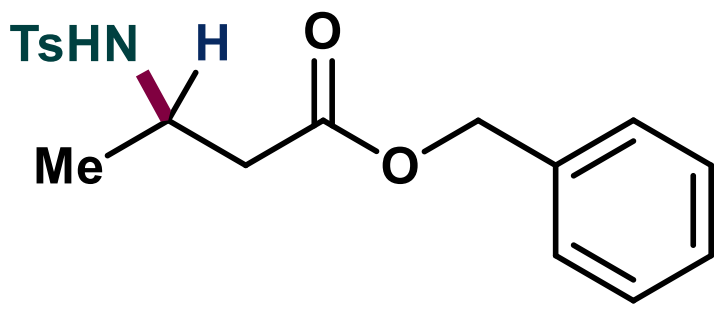

New compound, colorless oil, 54.2 mg, 78% yield.
Purification conditions for column chromatography: EtOAc:hexanes 1:49 to 1:9
R_f = 0.24 (EtOAc:hexanes = 1:4 v/v)
^{1}H NMR (500 MHz, $CDCl_3$): δ 7.67 (d, *J* = 8.2 Hz, 2H), 7.29 – 7.21 (m, 5H), 7.17 (d, *J* = 8.0 Hz, 2H), 5.37 (d, *J* = 8.4 Hz, 1H), 4.97 (q, *J* = 12.3 Hz, 2H), 3.67 – 3.59 (m, 1H), 2.39 (ddd, *J* = 22.2 Hz, 16.0 Hz, 5.7 Hz, 1H), 2.31 (s, 3H), 1.03 (d, *J* = 6.7 Hz, 3H).
^{13}C NMR (126 MHz, $CDCl_3$): δ 171.0, 143.4, 138.0, 135.5, 129.8, 128.6, 128.4, 128.3, 127.1, 66.5, 46.7, 41.0, 21.5, 21.1
HR-MS: m/z calcd. $[C_{18}H_{21}NO_4SNa]^+$: 370.1089; found (ESI): 370.1085.

Methyl 4-chloro-3-((4-methylphenyl)sulfonamido)butanoate (8)

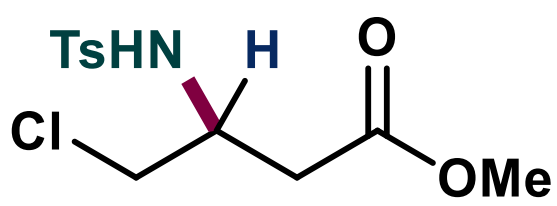

New compound, white powder, 44.6 mg, 73% yield.
Purification conditions for column chromatography: EtOAc:hexanes 1:49 to 1:9
R_f = 0.36 (EtOAc:hexanes = 1:4 v/v)
^{1}H NMR (500 MHz, $CDCl_3$): δ 7.75 (d, *J* = 8.2 Hz, 2H), 7.30 (d, *J* = 8.3 Hz, 2H), 5.47 (d, *J* = 8.8 Hz, 1H), 3.91 – 3.85 (m, 1H), 3.62 (dd, *J* = 11.3 Hz, 3.9 Hz, 1H), 3.60 (s, 3H), 3.52 (dd, *J* = 11.3 Hz, 6.4 Hz, 1H), 2.62 (qd, *J* = 16.7 Hz, 5.9 Hz, 2H), 2.42 (s, 3H).
^{13}C NMR (126 MHz, $CDCl_3$): δ 171.0, 143.9, 137.5, 129.9, 127.1, 52.1, 51.0, 46.6, 36.1, 21.6.
HR-MS: m/z calcd. $[C_{12}H_{16}ClNO_4SNa]^+$: 328.0386; found (ESI): 328.0385

Methyl 2-chloro-3-((4-methylphenyl)sulfonamido)butanoate (9)

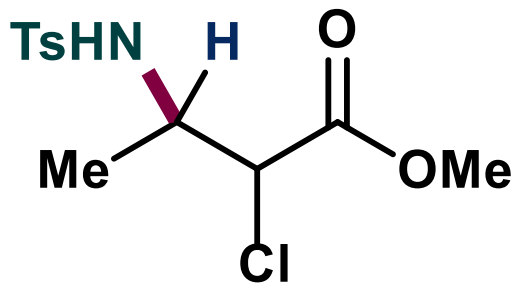

New compound, colorless oil, 52 mg, 85% yield.
Purification conditions for column chromatography: EtOAc:hexanes 1:49 to 1:9
R$_f$ = 0.53 (EtOAc:hexanes = 1:4 v/v)
^{1}H NMR (500 MHz, $CDCl_3$): δ 7.76 (dd, *J* = 13.1, 8.3 Hz, 4H), 7.31 – 7.30 (m, 3.83H), 5.14 – 5.12 (m, 2H), 4.42 (d, *J* = 4.0 Hz, 0.73H), 4.02 – 3.94 (m, 2H), 3.74 (s, 2H), 3.65 (s, 3H) , 2.42 (s, 5.84H), 1.20 (d, *J* = 6.7 Hz, 3H), 1.10 (d, *J* = 6.7 Hz, 2.55H).
^{13}C NMR (126 MHz, $CDCl_3$): δ 167.9, 167.8, 143.9, 143.8, 137.8, 137.7, 130.0, 129.9, 127.2, 127.1, 61.8, 61.4, 53.4, 53.3, 52.2, 51.6, 29.8, 29.4, 21.7, 21.6, 18.5, 17.0
HR-MS: m/z calcd. $[C_{12}H_{16}ClNO_4SNa]^+$: 328.0386; found (ESI): 328.0385.

Ethyl 2-((4-methylphenyl)sulfonamido)cyclopentane-1-carboxylate (10)

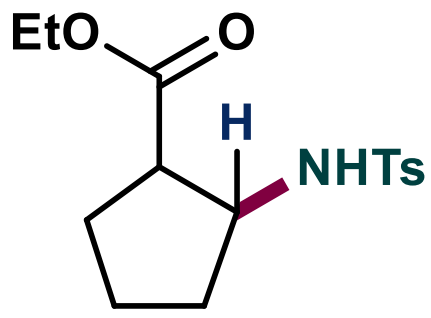

Known compound, see Ref. 9. 41.1 mg, 66% yield.
Purification conditions for column chromatography: EtOAc:hexanes 1:49 to 1:9
^{1}H NMR (500 MHz, $CDCl_3$): δ 7.75 (d, *J* = 8.2 Hz, 2H), 7.29 (d, *J* = 8.0 Hz, 2H), 4.96 (d, *J* = 6.0 Hz, 1H), 4.03 (q, *J* = 7.1 Hz, 2H), 3.74 – 3.69 (m, 1H), 2.63 (dd, *J* = 16.5 Hz, 7.7Hz, 1H), 2.42 (s, 3H), 2.10 – 1.93 (m, 2H), 1.79 – 1.70 (m, 1H), 1.65 – 1.61 (m, 2H), 1.50 –1.43 (m, 1H), 1.19 (t, *J* = 7.1 Hz, 3H)
^{13}C NMR (126 MHz, $CDCl_3$): δ 174.3, 143.5, 137.3, 129.8, 127.4, 60.9, 57.9, 50.9, 33.7, 28.1, 23.0, 21.6, 14.2.

Ethyl 2-((4-methylphenyl)sulfonamido)cyclohexane-1-carboxylate (11)

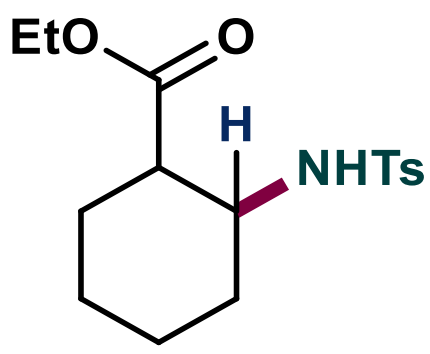

New compound, white powder, 46.9 mg, 72% yield.
Purification conditions for column chromatography: EtOAc:hexanes 1:49 to 1:9
R$_f$ = 0.32 (EtOAc:hexanes = 1:4 v/v)
^{1}H NMR (500 MHz, $CDCl_3$): δ 7.73 (d, *J* = 8.0 Hz, 2H), 7.28 (d, *J* = 8.1 Hz, 2H), 4.78 (d, *J* = 7.8Hz, 1H), 3.98 – 3.91 (m, 1H), 3.87 – 3.81 (m, 1H), 3.39 – 3.33 (m, 1H), 2.41 (s, 3H), 2.22 (td, *J* = 11.0 Hz, 3.7Hz, 1H), 2.01 – 1.99 (m, 1H), 1.88 – 1.86 (m, 1H), 1.68 – 1.63 (m, 3H), 1.54 – 1.46 (m, 1H), 1.30 – 1.22 (m, 2H), 1.15 (t, *J* = 7.0 Hz, 3H).
^{13}C NMR (126 MHz, $CDCl_3$): δ 173.6, 143.3, 138.4, 129.7, 127.2, 60.8, 54.4, 50.0, 33.9, 28.6, 24.5, 24.2, 21.7, 14.2.
HR-MS: m/z calcd. $[C_{16}H_{23}NO_4SNa]^+$: 348.1245; found (ESI): 348.1237

Methyl 3-((4-methylphenyl)sulfonamido)-4-(2,4,5-trifluorophenyl)butanoate (12)

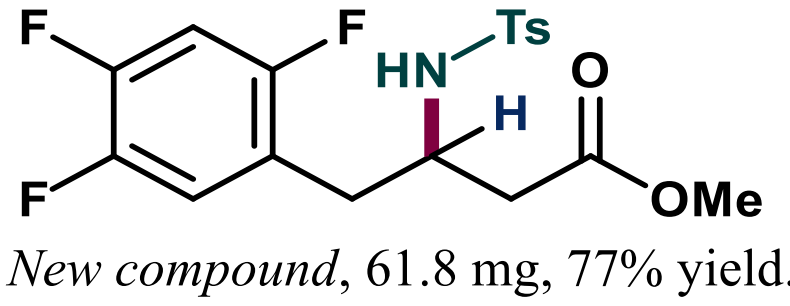

New compound, 61.8 mg, 77% yield.
Purification conditions for column chromatography: EtOAc:hexanes 1:49 to 1:9
^{1}H NMR (500 MHz, $CDCl_3$): δ 7.54 (d, *J* = 8.1 Hz, 2H), 7.17 (d, *J* = 8.0 Hz, 2H), 6.79 (dd, *J* = 16.9 Hz, 9.1 Hz, 1H), 6.69 (td, *J* = 9.6 Hz, 6.8 Hz, 1H), 5.42 (d, *J* = 7.5 Hz, 1H), 3.75 – 3.69 (m, 1H), 3.66 (s, 3H), 2.78 – 2.71 (m, 2H), 2.59 (qd, *J* = 16.6 Hz, 5.2 Hz, 2H), 2.40 (s, 3H).

^{13}**C NMR** (126 MHz, $CDCl_3$): δ 171.6, 156.0 (ddd, J_{C-F} = 244.1 Hz, 9.6 Hz, 2.5 Hz), 150.2 – 148.0 (m), 146.6 (ddd, J_{C-F} = 245.0 Hz, 12.3 Hz, 3.5 Hz), 143.6, 137.3, 129.6, 126.9, 120.9 – 120.4 (m), 119.0 (dd, J_{C-F} = 19.3 Hz, 5.5 Hz), 105.4 (dd, J_{C-F} = 28.5 Hz, 20.8 Hz), 52.0, 51.2, 39.0, 33.4, 21.5.
^{19}**F NMR** (471 MHz, $CDCl_3$): δ -119.4 (dd, J = 15.7 Hz, 4.0 Hz, 1F), -135.3 (dd, J = 26.1 Hz, 3.8 Hz, 1F), -142.5 (dd, J = 25.7 Hz, 15.5 Hz, 1F).
HR-MS: m/z calcd. $[C_{18}H_{18}F_3NO_4SNa]^+$: 424.0806; found (ESI): 424.0801.

Ethyl 3-((4-methylphenyl)sulfonamido)-3-phenylpropanoate (13)

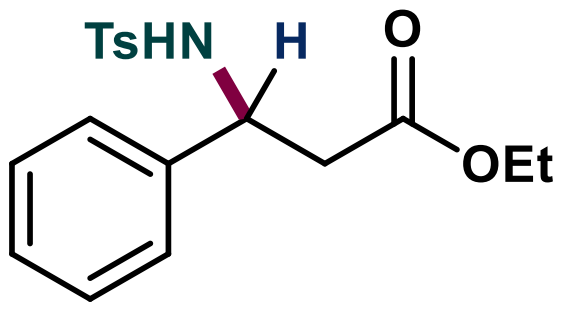

Known compound, see Ref. 10. 52.8 mg, 76% yield.
Purification conditions for column chromatography: EtOAc:hexanes 1:49 to 1:9
^{1}**H NMR** (500 MHz, $CDCl_3$): δ 7.59 (d, J = 8.1 Hz, 2H), 7.17 – 7.14 (m, 5H), 7.11 – 7.10 (m, 2H), 5.88 (d, J = 7.8 Hz, 1H) 4.74 (dd, J = 13.8 Hz, 6.6Hz, 1H), 4.00 (q, J = 7.1 Hz, 2H), 2.78 (ddd, J = 49.6 Hz, 15.8 Hz, 6.3 Hz, 2H), 2.36 (s, 3H), 1.12 (t, J = 7.1 Hz, 3H).
^{13}**C NMR** (126 MHz, $CDCl_3$): δ 170.7, 143.3, 139.5, 137.6, 129.5, 128.6, 127.8, 127.2, 126.6, 61.0, 54.5, 41.5, 21.6, 14.1.

Methyl 3-(4-fluorophenyl)-3-((4-methylphenyl)sulfonamido)propanoate (14)

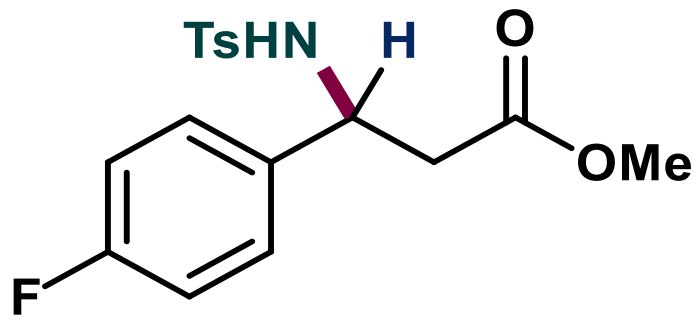

New compound, white solid, 47.8 mg, 68% yield.
Purification conditions for column chromatography: EtOAc:hexanes 1:49 to 1:9
$\mathbf{R_f}$ = 0.53 (EtOAc:hexanes = 1:4 v/v)
^{1}**H NMR** (500 MHz, $CDCl_3$): δ 7.58 (d, J = 8.3 Hz, 2H), 7.17 (d, J = 8.1 Hz, 2H), 7.08 (dd, J = 8.7 Hz, 5.2 Hz, 2H), 6.86 (t, J = 8.6 Hz, 2H), 5.82 (d, J = 7.6 Hz, 1H), 4.72 (dd, J = 13.7 Hz, 6.4 Hz, 1H), 3.56 (s, 3H), 2.77 (ddd, J = 44.3 Hz, 16.0 Hz, 6.2 Hz, 2H), 2.38 (s, 3H).
^{13}**C NMR** (126 MHz, $CDCl_3$): δ 171.1, 162.3 (d, J_{C-F} = 246.7 Hz) 143.6, 137.5, 135.3, 129.6, 128.4 (d, J_{C-F} = 8.2 Hz), 127.2, 115.6 (d, J_{C-F} = 21.7 Hz), 53.8, 52.1, 41.2, 21.6.
^{19}**F NMR** (471 MHz, $CDCl_3$): δ -114.4 (s, 1F).
HR-MS: m/z calcd. $[C_{17}H_{18}FNO_4SNa]^+$: 374.0838; found (ESI): 374.0835.

Methyl tosylalaninate (15)

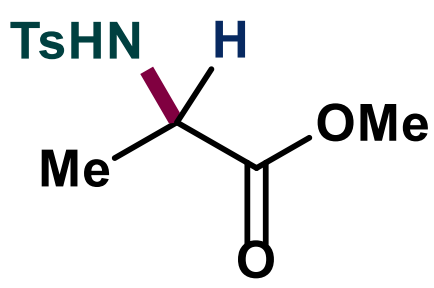

Known compound, see Ref. 11. 40.1 mg, 78% yield.
Purification conditions for column chromatography: EtOAc:hexanes 1:49 to 1:9
^{1}**H NMR** (500 MHz, $CDCl_3$): δ 7.74 (d, J = 8.2 Hz, 2H), 7.29 (d, J = 8.1 Hz, 2H), 5.44 (d, J = 8.3 Hz, 1H), 4.02 – 3.96 (m, 1H), 3.54 (s, 3H), 2.42 (s, 3H), 1.37 (d, J = 7.2 Hz, 3H).
^{13}**C NMR** (126 MHz, $CDCl_3$): δ 172.7, 143.7, 136.9, 129.7, 127.3, 52.6, 51.5, 21.6, 19.8.

Ethyl 7-chloro-2-((4-methylphenyl)sulfonamido)heptanoate (16)

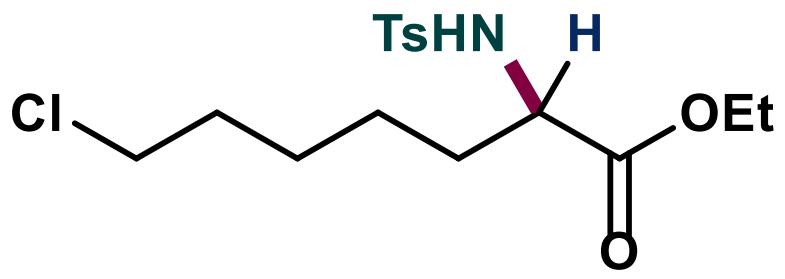

New compound, colorless oil, 63.7 mg, 88% yield.
Purification conditions for column chromatography: EtOAc:hexanes 1:49 to 1:9
R_f = 0.53 (EtOAc:hexanes = 1:4 v/v)
^{1}H NMR (500 MHz, $CDCl_3$): δ 7.70 (dd, *J* = 17.0, 8.1 Hz, 2H), 7.26 – 7.23 (m, 2H), 5.45 (d, *J* = 9.1 Hz, 1H), 3.88 (q, *J* = 7.1 Hz, 2H), 3.83 (td, *J* = 8.5, 5.3 Hz, 1H), 3.43 (q, *J* = 7.0 Hz, 2H), 2.36 (s, 3H), 1.70 – 1.63 (m, 2H), 1.60 – 1.53 (m, 1H), 1.33 (d, *J* = 3.7 Hz, 4H), 1.05 (t, *J* = 7.1 Hz, 3H).
^{13}C NMR (126 MHz, $CDCl_3$): δ 171.69, 143.65, 136.80, 129.60, 127.25, 61.62, 55.62, 44.81, 33.07, 32.22, 26.15, 24.20, 21.48, 13.90.
HR-MS: m/z calcd. $[C_{16}H_{24}ClNO_4SNa]^+$: 384.1012; found (ESI): 384.1008.

Methyl 2-((4-methylphenyl)sulfonamido)-2-phenylacetate (17)

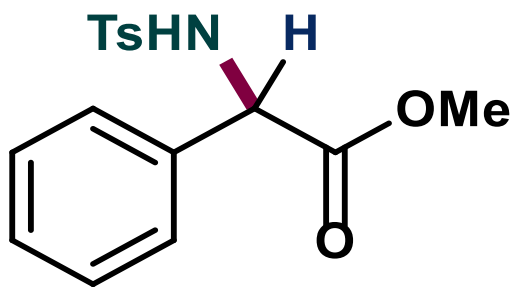

Known compound, see Ref. 12. 47.3 mg, 74% yield.
Purification conditions for column chromatography: EtOAc:hexanes 1:49 to 1:9
^{1}H NMR (500 MHz, $CDCl_3$): δ 7.62 (d, *J* = 8.1 Hz, 2H), 7.26 – 7.18 (m, 7H), 5.79 (d, *J* = 7.9 Hz, 1H), 5.06 (d, *J* = 8.0 Hz, 1H), 3.56 (s, 3H), 2.38 (s, 3H).
^{13}C NMR (126 MHz, $CDCl_3$): δ 170.7, 143.6, 137.0, 135.3, 129.6, 128.9, 128.7, 127.3, 127.2, 59.4, 53.1, 21.6.

Ethyl 2-((4-methylphenyl)sulfonamido)-2-phenylacetate (18)

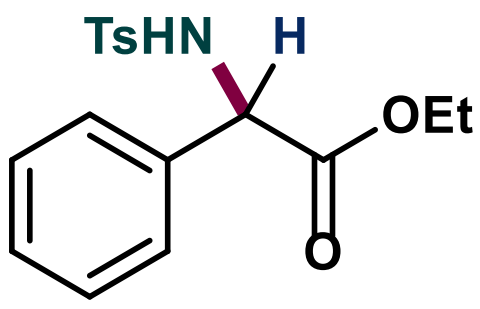

Known compound, see Ref. 13. 47.3 mg, 71% yield.
Purification conditions for column chromatography: EtOAc:hexanes 1:49 to 1:9
^{1}H NMR (500 MHz, $CDCl_3$): δ 7.63 (d, *J* = 8.2 Hz, 2H), 7.26 – 7.19 (m, 7H), 5.73 (d, *J* = 8.1 Hz, 1H), 5.04 (d, *J* = 8.1 Hz, 1H), 4.08 – 3.94 (m, 2H), 2.38 (s, 3H), 1.09 (t, *J* = 7.1 Hz, 3H).
^{13}C NMR (126 MHz, $CDCl_3$): δ 170.2, 143.6, 137.1, 135.5, 129.6, 128.9, 128.6, 127.3, 127.2, 62.3, 59.5, 21.6, 14.0.

4-Methyl-*N*-(4-phenylbutan-2-yl)benzenesulfonamide (19)

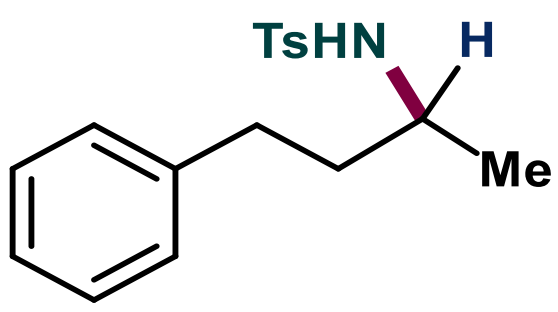

Known compound, see Ref. 14. 48.5 mg, 80% yield.
Purification conditions for column chromatography: EtOAc:hexanes 1:49 to 1:9
^{1}H NMR (500 MHz, $CDCl_3$): δ 7.76 (d, *J* = 8.2 Hz, 2H), 7.29 (d, *J* = 8.1 Hz, 2H), 7.24 (t, *J* = 7.4 Hz, 2H), 7.17 (t, *J* = 7.3 Hz, 1H), 7.05 (d, *J* = 7.2 Hz, 2H), 4.69 (d, *J* = 8.2 Hz, 1H), 3.39 – 3.31 (m, 1H), 2.63 – 2.57 (m, 1H), 2.55 – 2.49 (m, 1H), 2.43 (s, 3H), 1.71 – 1.66 (m, 2H), 1.06 (d, *J* = 6.6 Hz, 3H).
^{13}C NMR (126 MHz, $CDCl_3$): δ 143.4, 141.5, 138.3, 129.8, 128.5, 128.4, 127.2, 126.0, 49.8, 39.3, 31.9, 21.8, 21.6.

4-Methyl-*N*-(pentan-2-yl)benzenesulfonamide (20)

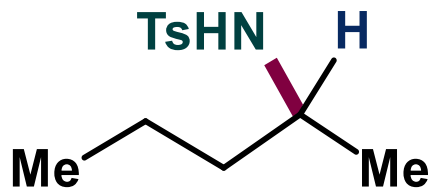

Known compound, see Ref. 15. 33.3 mg, 69% yield.
Purification conditions for column chromatography: EtOAc:hexanes 1:49 to 1:9
^{1}H NMR (500 MHz, $CDCl_3$): δ 7.76 (d, *J* = 8.2 Hz, 2H), 7.29 (d, *J* = 8.1 Hz, 2H), 7.24 (t, *J* = 7.4 Hz, 2H), 7.17 (t, *J* = 7.3 Hz, 1H), 7.05 (d, *J* = 7.2 Hz, 2H), 4.69 (d, *J* = 8.2 Hz, 1H), 3.39 – 3.31 (m, 1H), 2.63 – 2.57 (m, 1H), 2.55 – 2.49 (m, 1H), 2.43 (s, 3H), 1.71 – 1.66 (m, 2H), 1.06 (d, *J* = 6.6 Hz, 3H).
^{13}C NMR (126 MHz, $CDCl_3$): δ 143.4, 141.5, 138.3, 129.8, 128.5, 128.4, 127.2, 126.0, 49.8, 39.3, 31.9, 21.8, 21.6.

***N*-(decan-2-yl)-4-methylbenzenesulfonamide (21)**

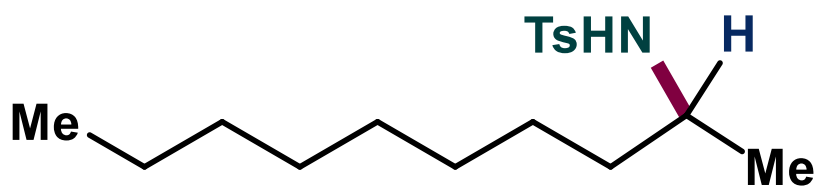

New compound, colorless oil, 43.6 mg, 70% yield.
Purification conditions for column chromatography: EtOAc:hexanes 1:49 to 1:9
R_f = 0.53 (EtOAc:hexanes = 1:4 v/v)
^{1}H NMR (500 MHz, $CDCl_3$): δ 7.76 (d, *J* = 8.2 Hz, 2H), 7.28 (d, *J* = 8.1 Hz, 2H), 4.51 (d, *J* = 8.0 Hz, 1H), 3.27 (dp, *J* = 13.2 Hz, 6.6 Hz, 1H), 2.41 (s, 3H), 1.35 – 1.30 (m, 2H), 1.29 – 1.25 (m, 2H), 1.23 – 1.18 (m, 5H), 1.13 – 1.12 (m, 5H), 1.02 (d, *J* = 6.5 Hz, 3H), 0.87 (t, *J* = 7.1 Hz, 3H).
^{13}C NMR (126 MHz, $CDCl_3$): δ 143.2, 138.4, 129.7, 127.2, 50.1, 37.6, 32.0, 29.5, 29.4, 29.3, 25.6, 22.8, 21.9, 21.6, 14.2
HR-MS: m/z calcd. $[C_{17}H_{29}NO_2SNa]^+$: 334.1817; found (ESI): 334.1812.

4-Methyl-*N*-(5-methylhexan-2-yl)benzenesulfonamide (22)

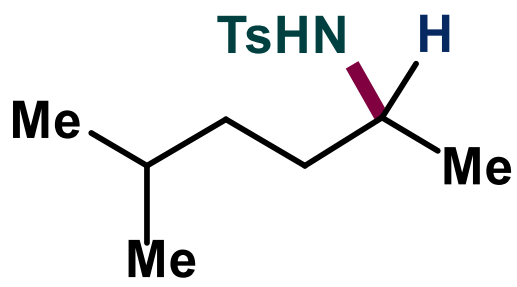

New compound, white solid, 40.4 mg, 75% yield.
Purification conditions for column chromatography: EtOAc:hexanes 1:49 to 1:9
R_f = 0.33 (EtOAc:hexanes = 1:4 v/v)
^{1}H NMR (500 MHz, $CDCl_3$): δ 7.76 (d, *J* = 8.3 Hz, 2H), 7.29 (d, *J* = 8.0 Hz, 2H), 4.50 (d, *J* = 8.1 Hz, 1H), 3.30 – 3.22 (m, 1H), 2.41 (s, 3H), 1.40 – 1.30 (m, 3H), 1.12 – 1.03 (m, 2H), 1.01 (d, *J* = 6.6 Hz, 3H), 0.78 – 0.75 (m, 6H).
^{13}C NMR (126 MHz, $CDCl_3$): δ 143.3, 138.5, 129.7, 127.2, 50.4, 35.4, 34.7, 27.8, 22.6, 22.5, 21.9, 21.6.
HR-MS: m/z calcd. $[C_{14}H_{23}NO_2SNa]^+$: 292.1347; found (ESI): 292.1346.

***N*-(5-chloropentan-2-yl)-4-methylbenzenesulfonamide (23)**

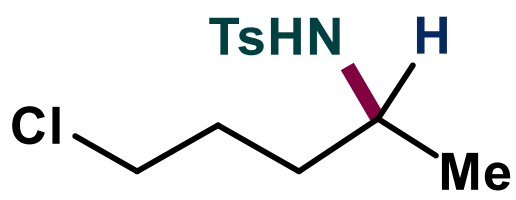

New compound, colorless oil, 37 mg, 67% yield.
Purification conditions for column chromatography: EtOAc:hexanes 1:49 to 1:9
R_f = 0.53 (EtOAc:hexanes = 1:4 v/v)
^{1}H NMR (500 MHz, $CDCl_3$): δ 7.76 (d, *J* = 8.3 Hz, 2H), 7.30 (d, *J* = 8.1 Hz, 2H), 4.50 (d, *J* = 8.3 Hz, 1H), 3.49 – 3.42 (m, 2H), 3.37 – 3.29 (m, 1H), 2.43 (s, 3H), 1.83 – 1.67 (m, 2H), 1.60 – 1.53 (m, 1H), 1.51 – 1.43 (m, 1H), 1.00 (d, *J* = 6.6 Hz, 3H).
^{13}C NMR (126 MHz, $CDCl_3$): δ 143.5, 138.2, 129.8, 127.1, 49.5, 44.7, 34.8, 28.7, 21.9, 21.6.
HR-MS: m/z calcd. $[C_{12}H_{18}ClNO_2SNa]^+$: 298.0645; found (ESI): 298.0645.

N-(1-cyclopropylethyl)-4-methylbenzenesulfonamide (24)

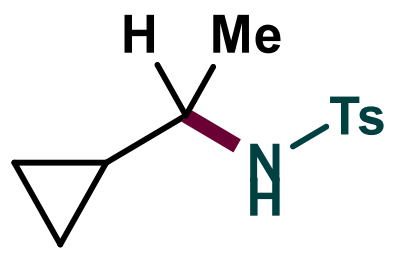

Known compound, see Ref. 16. 239.3 mg, 77% yield.
Purification conditions for column chromatography: EtOAc:hexanes 1:49 to 1:9
^{1}H NMR (500 MHz, $CDCl_3$): δ 7.76 (d, *J* = 8.2 Hz, 2H), 7.28 (d, *J* = 8.0 Hz, 2H), 4.89 (d, *J* = 6.4 Hz, 1H), 2.67 – 2.60 (m, 1H), 2.41 (s, 3H), 1.12 (d, *J* = 6.6 Hz, 3H), 0.78 – 0.71 (m, 1H), 0.44 – 0.39 (m, 1H), 0.33 – 0.27 (m, 1H), 0.14 – 0.09 (m, 1H), 0.01 – -0.04 (m, 1H).
^{13}C NMR (126 MHz, $CDCl_3$): δ 143.2, 138.3, 129.7, 127.2, 54.8, 21.6, 21.3, 18.0, 3.8, 3.3.

N-cyclohexyl-4-methylbenzenesulfonamide (25)

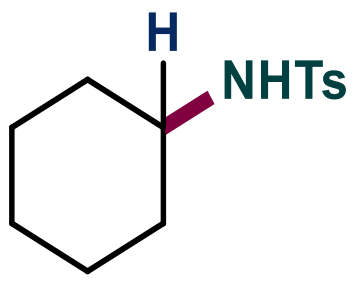

Known compound, see Ref. 17. 41.6 mg, 82% yield.
Purification conditions for column chromatography: EtOAc:hexanes 1:49 to 1:9
^{1}H NMR (500 MHz, $CDCl_3$): δ 7.79 (d, *J* = 8.2 Hz, 2H), 7.29 (d, *J* = 7.9 Hz, 2H), 5.13 – 5.09 (m, 1H), 3.11 – 3.10 (m, 1H), 2.42 (s, 3H), 1.74 – 1.72 (m, 2H), 1.63 – 1.60 (m, 2H), 1.50 – 1.47 (m, 1H), 1.22 – 1.08 (d, 5H).
^{13}C NMR (126 MHz, $CDCl_3$): δ 143.1, 138.6, 129.7, 127.0, 52.6, 33.8, 25.2, 24.7, 21.6.

N-(1-((3r,5r,7r)-adamantan-1-yl)ethyl)-4-methylbenzenesulfonamide (26)

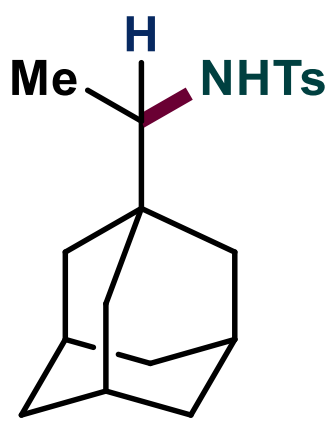

Known compound, see Ref. 18. 236 mg (0.97 mmol scale), 73% yield.
Purification conditions for column chromatography: EtOAc:hexanes 1:49 to 1:9
^{1}H NMR (500 MHz, $CDCl_3$): δ 7.76 (d, *J* = 8.3 Hz, 2H), 7.29 (d, *J* = 8.0 Hz, 2H), 4.31 (d, *J* = 9.5 Hz, 1H), 2.87 (dq, *J* = 9.6 Hz, 6.8 Hz, 1H), 2.42 (s, 3H), 1.95 (s, 3H), 1.68 – 1.65 (m, 3H), 1.58 – 1.56 (m, 3H), 1.55 – 1.54 (m, 1H) 1.523 – 1.520 (m, 2H), 1.35 (dd, *J* = 12.1 Hz, 1.9 Hz, 3H), 0.82 (d, *J* = 6.8 Hz, 3H).
^{13}C NMR (126 MHz, $CDCl_3$): δ 143.2, 138.5, 129.7, 127.2, 58.8, 38.4, 37.0, 36.1, 28.4, 21.7, 15.1.

1-((3r,5r,7r)-adamantan-1-yl)ethan-1-amine (27)

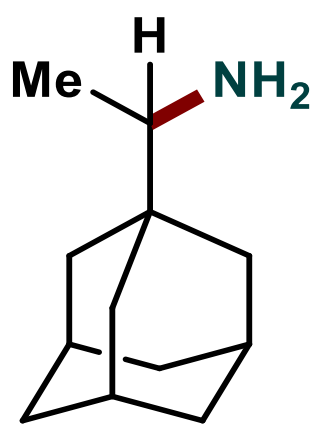

Known compound, see Ref. 19. 24.1 mg (0.19 mmol scale), 71% yield.
Purification conditions for column chromatography: MeOH:CH_2Cl_2 1:49 to 1:9
^{1}H NMR (500 MHz, $CDCl_3$): δ 2.85 (dd, *J* = 12.7, 6.1 Hz, 1H), 2.03 (s, 3H), 1.72 – 1.68 (m, 3H), 1.64 – 1.61 (m, 3H), 1.54 (dd, *J* = 23.8, 11.7 Hz, 6H), 1.21 (d, *J* = 6.7 Hz, 3H).
^{13}C NMR (126 MHz, $CDCl_3$): δ 57.4, 37.4, 36.6, 34.5, 28.0, 12.7.

4-Methyl-*N*-(1-phenylethyl)benzenesulfonamide (28)

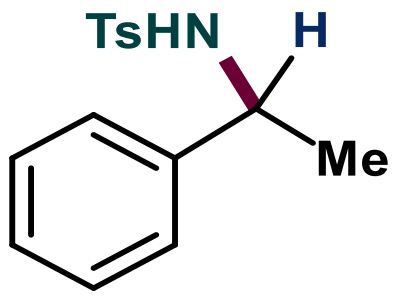

Known compound, see Ref. 20. 53.4 mg, 97% yield.
Purification conditions for column chromatography: EtOAc:hexanes 1:49 to 1:9
^{1}H NMR (500 MHz, $CDCl_3$): δ 7.65 (d, *J* = 8.2 Hz, 2H), 7.19 – 7.13 (m, 7H), 5.82 (d, *J* = 7.4 Hz, 1H), 4.47 (p, *J* = 7.0 Hz, 1H), 2.36 (s, 3H), 1.40 (d, *J* = 6.9 Hz, 3H).
^{13}C NMR (126 MHz, $CDCl_3$): δ 142.9, 142.3, 137.7, 129.4, 128.4, 127.2, 127.1, 126.1, 53.7, 23.6, 21.4.

4-Methyl-*N*-(1-(p-tolyl)ethyl)benzenesulfonamide (29)

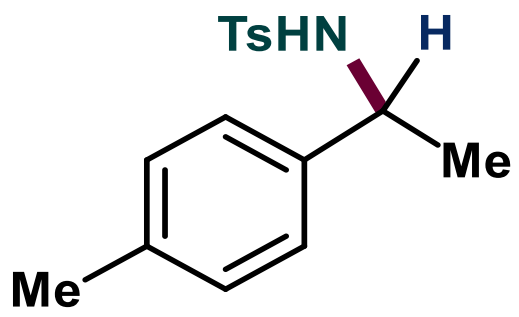

Known compound, see Ref. 20. 51.5 mg, 89% yield.
Purification conditions for column chromatography: EtOAc:hexanes 1:49 to 1:9
^{1}H NMR (500 MHz, $CDCl_3$): δ 7.63 (d, *J* = 8.2 Hz, 2H), 7.19 (d, *J* = 8.1 Hz, 2H), 7.02 – 6.97 (m, 4H), 4.89 (d, *J* = 6.8 Hz, 1H), 4.41 (p, *J* = 6.8Hz, 1H), 2.39 (s, 3H), 2.28 (s, 3H), 1.41 (d, *J* = 6.8 Hz, 3H).
^{13}C NMR (126 MHz, $CDCl_3$): δ 143.2, 139.2, 137.8, 137.3, 129.5, 129.3, 127.3, 126.2, 53.5, 23.6, 21.6, 21.1.

4-Methyl-*N*-(1-(*m*-tolyl)ethyl)benzenesulfonamide (30)

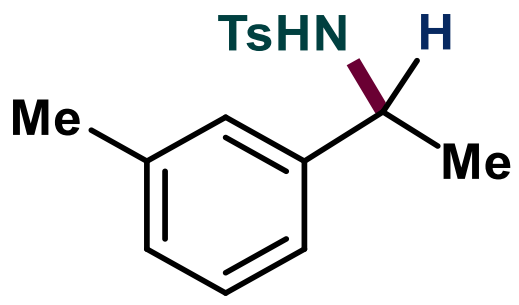

Known compound, see Ref. 20. 46.9 mg, 81% yield.
Purification conditions for column chromatography: EtOAc:hexanes 1:49 to 1:9
^{1}H NMR (500 MHz, $CDCl_3$): δ 7.61 (d, *J* = 8.2 Hz, 2H), 7.16 (d, *J* = 8.1 Hz, 2H), 7.08 (t, *J* = 7.6 Hz, 1H) , 6.96 (d, *J* = 7.5 Hz, 1H), 6.91 (d, *J* = 7.6 Hz, 1H), 6.83 (s, 1H), 5.27 – 5.24 (m, 7H), 4.42 (p, *J* = 6.9 Hz, 1H), 2.38 (s, 3H), 2.20 (s, 3H), 1.40 (d, *J* = 6.9 Hz, 3H).
^{13}C NMR (126 MHz, $CDCl_3$): δ 143.1, 142.0, 138.1, 137.8, 129.4, 128.5, 128.2, 127.2, 127.0, 123.3, 53.8, 23.7, 21.5, 21.3.

***N*-(1-(4-bromophenyl)ethyl)-4-methylbenzenesulfonamide (31)**

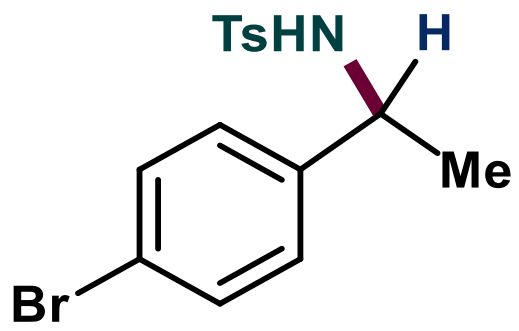

Known compound, see Ref. 21. 68 mg, 96% yield.
Purification conditions for column chromatography: EtOAc:hexanes 1:49 to 1:9
^{1}H NMR (500 MHz, $CDCl_3$): δ 7.58 (d, *J* = 8.1 Hz, 2H), 7.24 (d, *J* = 8.3 Hz, 2H), 7.15 (d, *J* = 8.0 Hz, 2H), 6.97 (d, *J* = 8.3 Hz, 2H), 5.72 (d, *J* = 7.3 Hz, 1H), 4.40 (p, *J* = 6.9 Hz, 1H), 2.39 (s, 3H), 1.35 (d, *J* = 6.9 Hz, 3H).
^{13}C NMR (126 MHz, $CDCl_3$): δ 143.4, 141.2, 137.5, 131.5, 129.5, 128.1, 127.1, 121.2, 53.2, 23.5, 21.6.

***N*-(1-(3-bromophenyl)ethyl)-4-methylbenzenesulfonamide (32)**

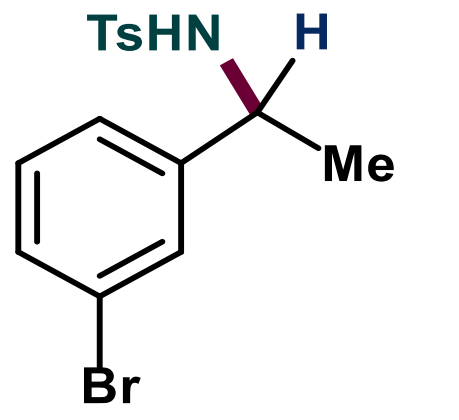

Known compound, see Ref. 22. 64.5 mg, 91% yield.
Purification conditions for column chromatography: EtOAc:hexanes 1:49 to 1:9
^{1}H NMR (500 MHz, $CDCl_3$): δ 7.58 (d, *J* = 8.2 Hz, 2H), 7.24 (d, *J* = 7.7 Hz, 1H), 7.15 (d, *J* = 8.1 Hz, 2H), 7.12 (s, 1H), 7.08 – 7.01 (m, 2H), 5.64 (d, *J* = 7.2 Hz, 1H), 4.42 (p, *J* = 7.0 Hz, 1H), 2.37 (s, 3H), 1.37 (d, *J* = 6.9 Hz, 3H).
^{13}C NMR (126 MHz, $CDCl_3$): δ 144.4, 143.4, 137.4, 130.4, 130.1, 129.54, 129.50, 127.1, 125.0, 122.5, 53.3, 23.6, 21.6.

***N*-(1-(2-bromophenyl)ethyl)-4-methylbenzenesulfonamide (33)**

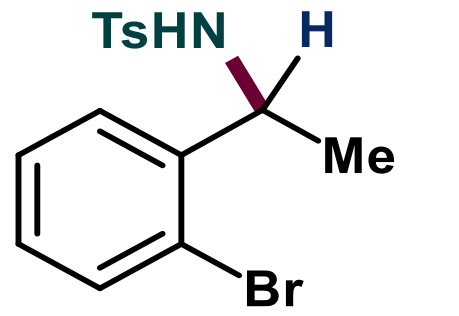

Known compound, see Ref. 22. 59.5 mg, 84% yield.
Purification conditions for column chromatography: EtOAc:hexanes 1:49 to 1:9
^{1}H NMR (500 MHz, $CDCl_3$): δ 7.65 (d, *J* = 8.2 Hz, 2H), 7.36 (d, *J* = 7.9 Hz, 1H), 7.25 (dd, *J* = 7.8 Hz, 1.2 Hz, 1H), 7.13 (d, *J* = 8.1 Hz, 2H), 7.10 (t, *J* = 7.7 Hz, 1H), 6.97 (td, *J* = 7.9 Hz, 1.4 Hz, 1H), 5.91 (d, *J* = 7.4 Hz, 1H), 4.89 (p, *J* = 7.0 Hz, 1H), 2.34 (s, 3H), 1.37 (d, *J* = 6.9 Hz, 3H).
^{13}C NMR (126 MHz, $CDCl_3$): δ 143.2, 141.4, 137.1, 132.8, 129.4, 128.6, 127.82, 127.80, 127.2, 122.0, 53.1, 23.1, 21.5.

***N*-(1-(4-chlorophenyl)ethyl)-4-methylbenzenesulfonamide (34)**

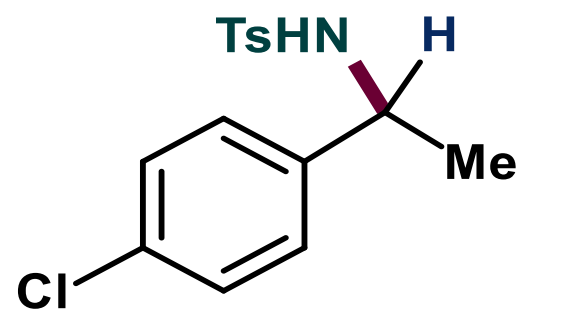

Known compound, see Ref. 23. 57 mg, 92% yield.
Purification conditions for column chromatography: EtOAc:hexanes 1:49 to 1:9
^{1}H NMR (500 MHz, $CDCl_3$): δ 7.58 (d, *J* = 8.1 Hz, 2H), 7.17 (d, *J* = 8.0 Hz, 2H), 7.12 (d, *J* = 8.3 Hz, 2H), 7.03 (d, *J* = 8.3 Hz, 2H), 5.26 (d, *J* = 7.0 Hz, 1H), 4.43 (p, *J* = 6.9 Hz, 1H), 2.39 (s, 3H), 1.37 (d, *J* = 6.9 Hz, 3H).
^{13}C NMR (126 MHz, $CDCl_3$): δ 143.5, 140.7, 137.6, 133.3, 129.6, 128.7, 127.7, 127.2, 53.2, 23.6, 21.6.

***N*-(1-(2-chlorophenyl)ethyl)-4-methylbenzenesulfonamide (5)**

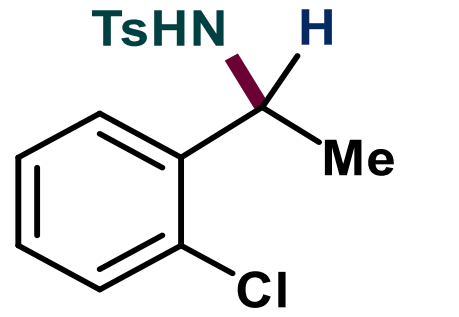

New compound, white crystal, 56.4 mg, 91% yield.
Purification conditions for column chromatography: EtOAc:hexanes 1:49 to 1:9
R_f = 0.51 (EtOAc:hexanes = 1:4 v/v)
^{1}H NMR (500 MHz, $CDCl_3$): δ 7.63 (d, *J* = 8.2 Hz, 2H), 7.26 – 7.22 (m, 1H), 7.18 – 7.15 (m, 1H), 7.13 (d, *J* = 8.0 Hz, 2H), 7.09 – 7.05 (m, 2H), 5.63 (d, *J* = 7.6 Hz, 1H), 4.89 (p, *J* = 7.0 Hz, 1H), 2.34 (s, 3H), 1.40 (d, *J* = 6.9 Hz, 3H).
^{13}C NMR (126 MHz, $CDCl_3$): δ 143.3, 139.7, 137.2, 131.9, 129.7, 129.5, 128.4, 127.8, 127.2, 51.1, 22.8, 21.5.

HR-MS: m/z calcd. $[C_{15}H_{16}ClNO_2SNa]^+$: 332.0488; found (ESI): 332.0480.

***N*-(1-(4-fluorophenyl)ethyl)-4-methylbenzenesulfonamide (36)**

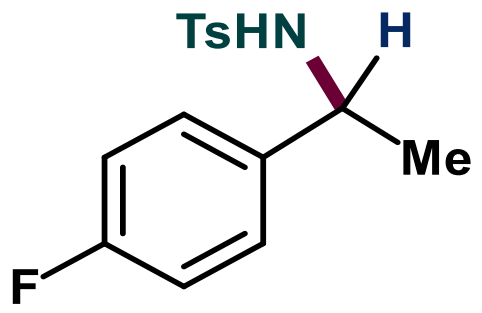

Known compound, see Ref. 20. 52.8 mg, 90% yield.
Purification conditions for column chromatography: EtOAc:hexanes 1:49 to 1:9
^{1}H NMR (500 MHz, $CDCl_3$): δ 7.60 (d, *J* = 8.3 Hz, 2H), 7.17 (d, *J* = 8.1 Hz, 2H), 7.08 – 7.05 (m, 2H) 6.86 – 6.82 (m, 2H), 5.36 (d, *J* = 7.0 Hz, 1H), 4.44 (p, *J* = 6.9 Hz, 1H), 2.38 (s, 3H), 1.37 (d, *J* = 6.9 Hz, 3H).
^{13}C NMR (126 MHz, $CDCl_3$): δ 162.1 (d, J_{C-F} = 245.8 Hz), 143.4, 138.1 (d, J_{C-F} = 3.2 Hz), 137.7, 129.6, 128.0 (d, J_{C-F} = 8.1 Hz), 127.2, 115.3 (d, J_{C-F} = 21.5 Hz), 53.1, 23.7, 21.6.
^{19}F NMR (471 MHz, $CDCl_3$): δ -115.0 (s, 1F).

4-Methyl-*N*-(1-(4-(trifluoromethyl)phenyl)ethyl)benzenesulfonamide (37)

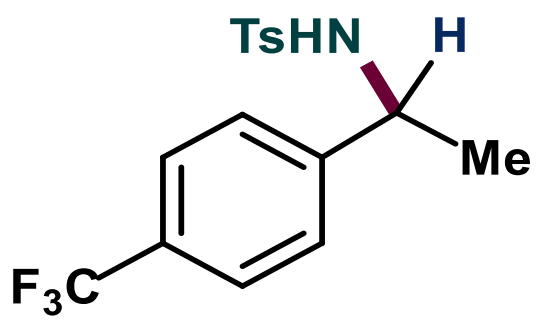

Known compound, see Ref. 23. 62.5 mg, 91% yield.
Purification conditions for column chromatography: EtOAc:hexanes 1:49 to 1:9
^{1}H NMR (500 MHz, $CDCl_3$): δ 7.54 (d, *J* = 8.1 Hz, 2H), 7.37 (d, *J* = 8.0 Hz, 2H), 7.20 (d, *J* = 8.0 Hz, 2H), 7.11 (d, *J* = 8.0 Hz, 2H), 5.56 (d, *J* = 7.2 Hz, 1H), 4.53 (p, *J* = 6.9 Hz, 1H), 2.35 (s, 3H), 1.41 (d, *J* = 6.9 Hz, 3H).
^{13}C NMR (126 MHz, $CDCl_3$): δ 146.1, 143.5, 137.4, 129.8, 129.5, 127.2, 126.8, 125.5 (q, J_{C-F} = 3.8 Hz), 124.1 (dd, J_{C-F} = 544.1 Hz, 272.1 Hz), 53.5, 23.6, 21.4.
^{19}F NMR (471 MHz, $CDCl_3$): δ -62.6 (s, 3F).

***N*-(1-(3,5-difluorophenyl)ethyl)-4-methylbenzenesulfonamide (38)**

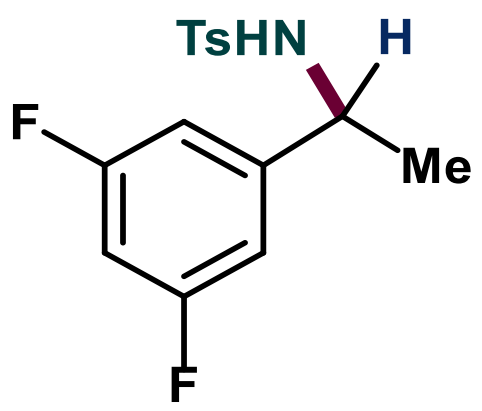

New compound, white solid, 52.3 mg, 84% yield.
Purification conditions for column chromatography: EtOAc:hexanes 1:49 to 1:9
R_f = 0.53 (EtOAc:hexanes = 1:4 v/v)
^{1}H NMR (500 MHz, $CDCl_3$): δ 7.63 (d, *J* = 8.1 Hz, 2H), 7.19 (d, *J* = 8.0 Hz, 2H), 6.64 (d, *J* = 6.4 Hz, 2H), 6.56 (t, *J* = 8.8 Hz, 1H), 5.73 (d, *J* = 7.2 Hz, 1H), 4.41 (p, *J* = 6.9 Hz, 1H), 2.38 (s, 3H), 1.35 (d, *J* = 6.9 Hz, 3H).
^{13}C NMR (126 MHz, $CDCl_3$): δ 163.0 (dd, J_{C-F} = 248.9 Hz, 12.6 Hz), 146.5 (t, J_{C-F} = 8.3 Hz), 143.7, 137.4, 129.6, 127.2, 109.3 (dd, J_{C-F} = 19.7 Hz, 6.0 Hz), 102.6 (t, J_{C-F} = 25.3 Hz), 53.1, 23.4, 21.5.
^{19}F NMR (471 MHz, $CDCl_3$): δ -109.4 (s, 2F).
HR-MS: m/z calcd. $[C_{15}H_{15}F_2NO_2SNa]^+$: 334.0689; found (ESI): 334.0688.

4-Methyl-*N*-(1-phenylpropyl)benzenesulfonamide (39)

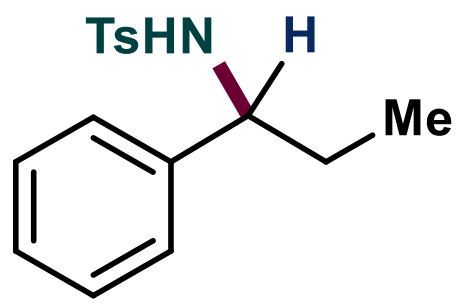

Known compound, see Ref. 20. 52.1 mg, 90% yield.
Purification conditions for column chromatography: EtOAc:hexanes 1:49 to 1:9
^{1}H NMR (500 MHz, $CDCl_3$): δ 7.56 (d, *J* = 8.2 Hz, 2H), 7.13 – 7.11 (m, 3H), 7.09 (d, *J* = 8.2 Hz, 2H), 7.03 – 7.01 (m, 2H), 5.48 (d, *J* = 7.6 Hz, 1H), 4.19 (q, *J* = 7.3 Hz, 1H), 2.34 (s, 3H), 1.84 – 1.76 (m, 1H), 1.74 – 1.65 (m, 1H), 0.78 (t, *J* = 7.4 Hz, 3H).
^{13}C NMR (126 MHz, $CDCl_3$): δ 142.9, 140.9, 137.9, 129.3, 128.4, 127.3, 127.1, 126.7, 60.0, 30.7, 21.5, 10.6.

***N*-(1-(4-bromophenyl)propyl)-4-methylbenzenesulfonamide (40)**

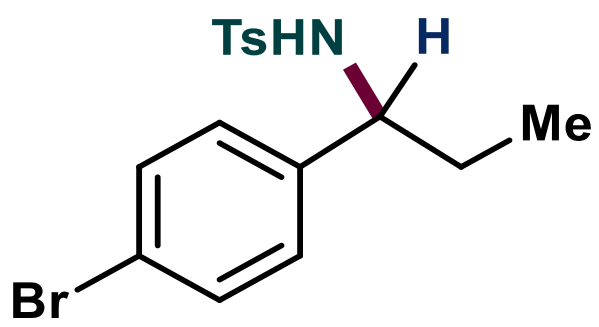

Known compound, see Ref. 24. 67.7 mg, 92% yield.
Purification conditions for column chromatography: EtOAc:hexanes 1:49 to 1:9
^{1}H NMR (500 MHz, $CDCl_3$): δ 7.50 (d, *J* = 8.2 Hz, 2H), 7.24 (d, *J* = 8.4 Hz, 2H), 7.12 (d, *J* = 8.2 Hz, 2H), 6.88 (d, *J* = 8.4 Hz, 2H), 5.23 (d, *J* = 7.2 Hz, 1H), 4.15 (q, *J* = 7.2 Hz, 1H), 2.38 (s, 3H), 1.81 – 1.72 (m, 1H), 1.69 – 1.62f (m, 1H), 0.78 (t, *J* = 7.4 Hz, 3H).
^{13}C NMR (126 MHz, $CDCl_3$): δ 143.4, 139.8, 137.6, 131.5, 129.5, 128.6, 127.2, 121.3, 59.4, 30.5, 21.6, 10.5.

4-Methyl-*N*-(1-phenylbutyl)benzenesulfonamide (41)

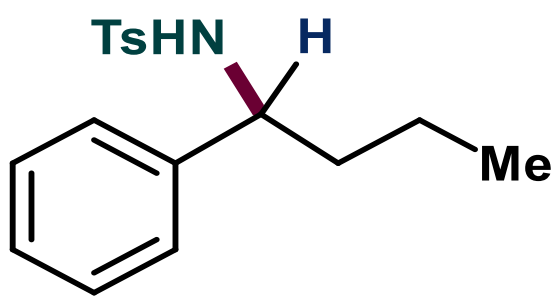

Known compound, see Ref. 25. 54.6 mg, 90% yield.
Purification conditions for column chromatography: EtOAc:hexanes 1:49 to 1:9
^{1}H NMR (500 MHz, $CDCl_3$): δ 7.53 (d, *J* = 8.2 Hz, 2H), 7.13 – 7.12 (m, 3H), 7.09 (d, *J* = 8.1 Hz, 2H), 7.02 – 7.00 (m, 2H), 5.24 (d, *J* = 7.4 Hz, 1H), 4.27 (q, *J* = 7.4 Hz, 1H), 2.34 (s, 3H), 1.78 – 1.71 (m, 1H), 1.68 – 1.60 (m, 1H), 1.30 – 1.21 (m, 1H), 1.18 – 1.09 (d, 1H), 0.82 (d, *J* = 7.4 Hz, 3H).
^{13}C NMR (126 MHz, $CDCl_3$): δ 143.0, 141.2, 137.9, 129.3, 128.5, 127.3, 127.2, 126.6, 58.2, 39.9, 21.5, 19.2, 13.7.

4-Methyl-*N*-(1-phenylheptyl)benzenesulfonamide (42)

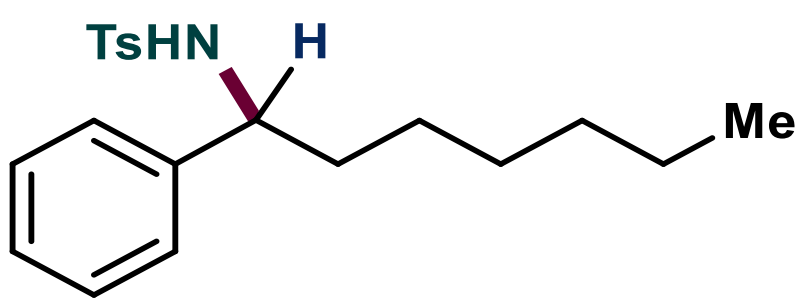

Known compound, see Ref. 26. 60.8 mg, 88% yield.
Purification conditions for column chromatography: EtOAc:hexanes 1:49 to 1:9
^{1}H NMR (500 MHz, $CDCl_3$): δ 7.56 (d, *J* = 8.2 Hz, 2H), 7.12 – 7.08 (m, 5H), 7.04 – 7.02 (m, 2H), 5.62 (d, *J* = 7.7 Hz, 1H), 4.25 (q, *J* = 7.4 Hz, 1H), 2.34 (s, 3H), 1.79 – 1.71 (m, 1H), 1.68 – 1.61 (m, 1H), 1.23 – 1.05 (d, 8H), 0.82 (t, *J* = 7.1 Hz, 3H).
^{13}C NMR (126 MHz, $CDCl_3$): δ 142.9, 141.3, 137.9, 129.3, 128.4, 127.2, 127.1, 126.6, 58.4, 37.7, 31.6, 28.8, 25.9, 22.6, 21.5, 14.1.

***N*-(3-chloro-1-phenylpropyl)-4-methylbenzenesulfonamide (43)**

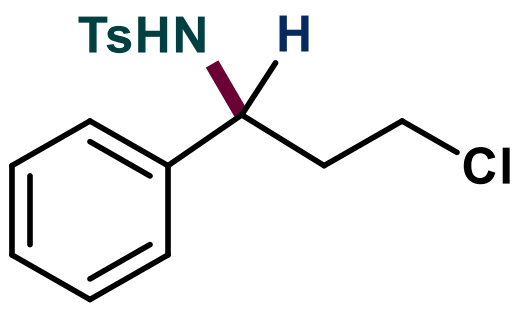

Known compound, see Ref. 27. 54.4 mg, 84% yield.
Purification conditions for column chromatography: EtOAc:hexanes 1:49 to 1:9
^{1}H NMR (500 MHz, $CDCl_3$): δ 7.58 (d, *J* = 8.2 Hz, 2H), 7.17 – 7.15 (m, 3H), 7.12 (d, *J* = 8.1 Hz, 2H), 7.04 – 7.02 (d, 2H), 5.61 (d, *J* = 8.1 Hz, 1H), 4.52 (q, *J* = 7.5 Hz, 1H), 3.50 – 3.46 (m, 1H), 3.29 (ddd, *J* = 11.2 Hz, 7.3 Hz, 5.8 Hz, 1H), 2.36 (s, 3H), 2.29 – 2.22 (m, 1H), 2.12 – 2.05 (m, 1H).
^{13}C NMR (126 MHz, $CDCl_3$): δ 143.2, 139.6, 137.3, 129.4, 128.7, 127.8, 127.1, 126.5, 55.7, 41.1, 40.0, 21.5.

***N*-(2,3-dihydro-1*H*-inden-1-yl)-4-methylbenzenesulfonamide (44)**

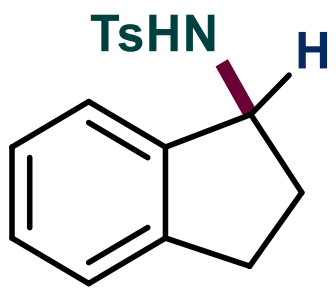

Known compound, see Ref. 20. 35.1 mg, 61% yield.
Purification conditions for column chromatography: EtOAc:hexanes 1:49 to 1:9
^{1}H NMR (500 MHz, $CDCl_3$): δ 7.84 (d, *J* = 8.2 Hz, 2H), 7.34 (d, *J* = 8.0 Hz, 2H), 7.22 – 7.08 (m, 4H), 4.82 (dd, *J* = 15.7 Hz, 7.6 Hz, 1H), 4.75 (d, *J* = 8.9 Hz, 1H), 2.90 (ddd, *J* = 15.9 Hz, 8.7 Hz, 3.5 Hz, 1H), 2.77 – 2.71 (m, 1H), 2.46 (s, 3H), 2.36 – 2.30 (m, 1H), 1.79 – 1.71 (m, 1H).
^{13}C NMR (126 MHz, $CDCl_3$): δ 143.6, 143.0, 142.1, 138.4, 129.9, 128.4, 127.3, 127.0, 124.9, 124.2, 58.9, 34.9, 30.1, 21.7.

***N*-(chroman-4-yl)-4-methylbenzenesulfonamide (45)**

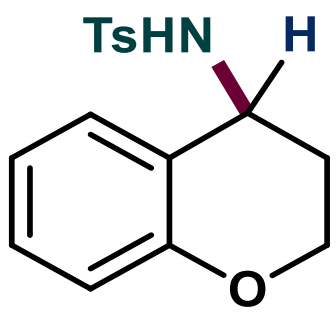

Known compound, see Ref. 28. 42.5 mg, 70% yield.
Purification conditions for column chromatography: EtOAc:hexanes 1:49 to 1:9
^{1}H NMR (500 MHz, $CDCl_3$): δ 7.82 (d, *J* = 8.2 Hz, 2H), 7.35 (d, *J* = 8.0 Hz, 2H), 7.12 – 7.08 (m, 1H), 6.77 – 6.71 (m, 3H), 4.91 (d, *J* = 6.5 Hz, 1H), 4.37 (dd, *J* = 10.8 Hz, 4.8 Hz 1H), 4.20 – 4.10 (m, 2H), 2.47 (s, 3H), 2.11 – 1.98 (m, 2H).
^{13}C NMR (126 MHz, $CDCl_3$): δ 155.1, 143.9, 137.7, 130.0, 129.8, 129.4, 127.3, 120.9, 120.8, 117.4, 62.5, 47.6, 29.8, 21.7.

***N*-(6-chlorochroman-4-yl)-4-methylbenzenesulfonamide (46)**

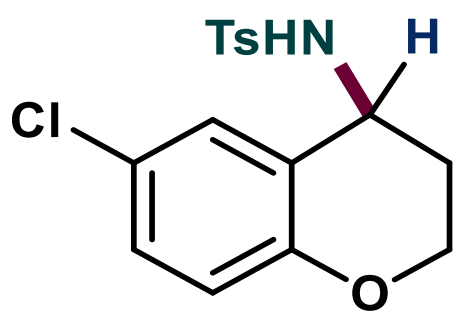

New compound, colorless oil, 35.1 mg, 52% yield.
Purification conditions for column chromatography: EtOAc:hexanes 1:49 to 1:9
R_f = 0.53 (EtOAc:hexanes = 1:4 v/v)
mp: 92 °C.
^{1}H NMR (500 MHz, $CDCl_3$): δ 7.82 (d, *J* = 8.1 Hz, 2H), 7.39 (d, *J* = 8.0 Hz, 2H), 7.05 (dd, *J* = 8.8 Hz, 2.3 Hz, 1H), 6.69 (d, *J* = 8.8 Hz, 1H), 6.52 (d, *J* = 2.2 Hz, 1H), 4.80 (d, *J* = 7.0 Hz, 1H), 4.35 (dd, *J* = 11.4 Hz, 4.9 Hz 1H), 4.36 – 4.10 (m, 2H), 2.48 (s, 3H), 2.09 – 1.99 (m, 2H).

^{13}C NMR (126 MHz, $CDCl_3$): δ 153.6, 144.3, 137.6, 130.2, 129.9, 128.9, 127.3, 125.5, 122.3, 118.9, 62.8, 47.5, 29.8, 21.7.
HR-MS: m/z calcd. $[C_{16}H_{16}ClNO_3SNa]^+$: 360.0431; found (ESI): 360.0432.

***N*-(9*H*-fluoren-9-yl)-4-methylbenzenesulfonamide (47)**

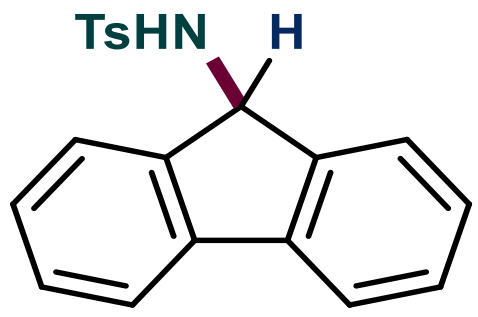

Known compound, see Ref. 29. 42.9 mg, 64% yield.
Purification conditions for column chromatography: EtOAc:hexanes 1:49 to 1:9
^{1}H NMR (500 MHz, $CDCl_3$): δ 7.94 (d, *J* = 8.2 Hz, 2H), 7.60 (d, *J* = 7.5 Hz, 2H), 7.41 (d, *J* = 8.1 Hz, 2H), 7.34 (dp, *J* = 8.0 Hz, 3.9 Hz, 2H), 7.23 – 7.19 (m, 4H), 5.38 (d, *J* = 9.5 Hz, 1H), 4.77 (d, *J* = 9.5 Hz, 1H), 2.51 (s, 3H).
^{13}C NMR (126 MHz, $CDCl_3$): δ 143.9, 143.5, 140.2, 138.6, 130.1, 129.1, 128.0, 127.5, 125.3, 120.1, 58.5, 21.8.

***N*-benzhydryl-4-methylbenzenesulfonamide (48)**

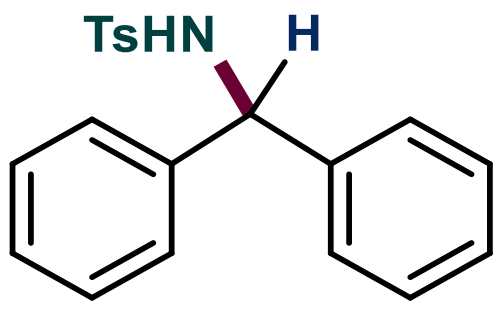

Known compound, see Ref. 30. 33.1 mg, 49% yield.
Purification conditions for column chromatography: EtOAc:hexanes 1:49 to 1:9
^{1}H NMR (500 MHz, $CDCl_3$): δ 7.56 (d, *J* = 8.2 Hz, 2H), 7.23 – 7.20 (m, 6H), 7.13 – 7.10 (m, 6H), 5.57 (d, *J* = 7.2 Hz, 1H), 5.27 (d, *J* = 7.2 Hz, 1H), 2.37 (s, 3H).
^{13}C NMR (126 MHz, $CDCl_3$): δ 143.3, 140.7, 137.5, 129.5, 128.7, 127.7, 127.5, 127.3, 61.5, 21.6.

***N*-(1,3-diphenylpropyl)-4-methylbenzenesulfonamide (49)**

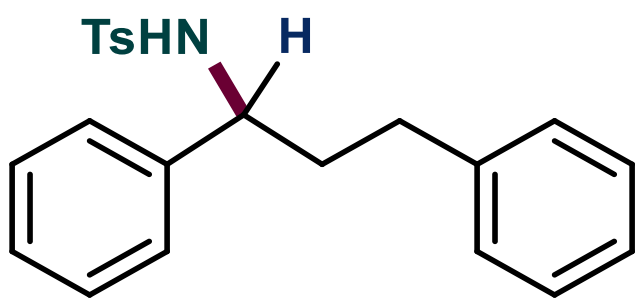

Known compound, see Ref. 31. 43.9 mg, 60% yield.
Purification conditions for column chromatography: EtOAc:hexanes 1:49 to 1:9
^{1}H NMR (500 MHz, $CDCl_3$): δ 7.44 (d, *J* = 8.2 Hz, 2H), 7.18 – 7.14 (m, 2H), 7.11 – 7.06 (m, 4H), 7.01 (d, *J* = 8.0 Hz, 2H), 6.96 (d, *J* = 7.2 Hz, 2H), 6.94 – 6.92 (d, 2H), 5.12 (d, *J* = 7.6 Hz, 1H), 4.20 (q, *J* = 7.3 Hz, 1H), 2.50 – 2.38 (m, 2H), 2.27 (s, 3H), 2.08 – 2.00 (m, 1H), 1.97 – 1.89 (m, 1H).
^{13}C NMR (126 MHz, $CDCl_3$): δ 143.1, 141.0, 140.7, 137.7, 129.4, 128.7, 128.53, 128.50, 127.6, 127.2, 126.7, 126.1, 58.0, 39.1, 32.2, 21.6.

***N*-hexyl-4-methylbenzenesulfonamide (50)**

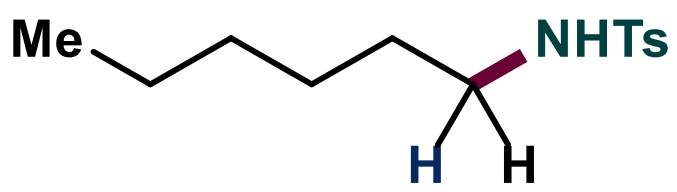

Known compound, see Ref. 32. 36.8 mg, 72% yield.
Purification conditions for column chromatography: EtOAc:hexanes 1:49 to 1:9
^{1}H NMR (500 MHz, $CDCl_3$): δ 7.75 (d, *J* = 8.2 Hz, 2H), 7.29 (d, *J* = 8.1 Hz, 2H), 4.90 (t, *J* = 6.0 Hz, 1H), 2.89 (dd, *J* = 13.5 Hz, 6.9 Hz, 2H), 2.41 (s, 3H), 1.45 – 1.39 (m, 2H), 1.25 – 1.15 (m, 6H), 0.82 (t, *J* = 7.0, 3H).
^{13}C NMR (126 MHz, $CDCl_3$): δ 143.3, 137.1, 129.7, 127.2, 43.3, 31.3, 29.5, 26.3, 22.5, 21.6, 14.0.

4-Methyl-*N*-(3-phenylpropyl)benzenesulfonamide (51)

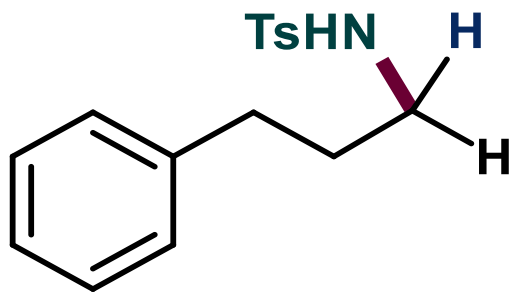

Known compound, see Ref. 33. 44 mg, 76% yield.
Purification conditions for column chromatography: EtOAc:hexanes 1:49 to 1:9
^{1}H NMR (500 MHz, $CDCl_3$): δ 7.74 (d, *J* = 8.2 Hz, 2H), 7.28 (d, *J* = 8.1 Hz, 2H), 7.23 (t, *J* = 7.4 Hz, 2H), 7.16 (t, *J* = 7.3 Hz, 1H), 7.06 (d, *J* = 7.2 Hz, 2H), 4.93 (t, *J* = 6.1 Hz, 1H), 2.94 (dd, *J* = 13.3 Hz, 6.7 Hz, 2H), 2.59 – 2.56 (m, 2H), 2.41 (s, 3H), 1.79 – 1.73 (m, 2H).
^{13}C NMR (126 MHz, $CDCl_3$): δ 143.5, 141.0, 137.0, 129.8, 128.5, 128.4, 127.2, 126.1, 42.7, 32.8, 31.2, 21.6.

4-Methyl-*N*-phenethylbenzenesulfonamide (52)

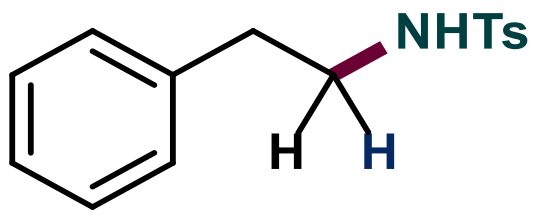

Known compound, see Ref. 32. 42.4 mg, 77% yield.
Purification conditions for column chromatography: EtOAc:hexanes 1:49 to 1:9
^{1}H NMR (500 MHz, $CDCl_3$): δ 7.69 (d, *J* = 8.3 Hz, 2H), 7.29 – 7.19 (m, 5H), 7.07 (d, *J* = 7.0 Hz, 2H), 4.55 (t, *J* = 6.1 Hz, 1H), 3.20 (dd, *J* = 13.4 Hz, 6.9 Hz, 2H), 2.75 (t, *J* = 7.0 Hz, 1H), 2.42 (s, 3H).
^{13}C NMR (126 MHz, $CDCl_3$): δ 143.5, 137.8, 137.0, 129.8, 128.9, 127.2, 126.9, 44.3, 35.9, 21.6.

***N*-benzyl-4-methylbenzenesulfonamide (53)**

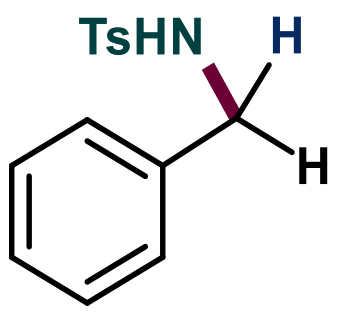

Known compound, see Ref. 32. 46 mg, 88% yield.
Purification conditions for column chromatography: EtOAc:hexanes 1:49 to 1:9
^{1}H NMR (500 MHz, $CDCl_3$): δ 7.74 (d, *J* = 8.2 Hz, 2H), 7.28 – 7.22 (m, 5H), 7.21 – 7.17 (m, 2H), 4.99 (t, *J* = 6.0 Hz, 1H), 4.09 (d, *J* = 6.2 Hz, 2H), 2.42 (s, 3H).
^{13}C NMR (126 MHz, $CDCl_3$): δ 143.6, 136.9, 136.4, 129.8, 128.7, 128.0, 127.9, 127.3, 47.3, 21.6.

***N*-(4-bromobenzyl)-4-methylbenzenesulfonamide (54)**

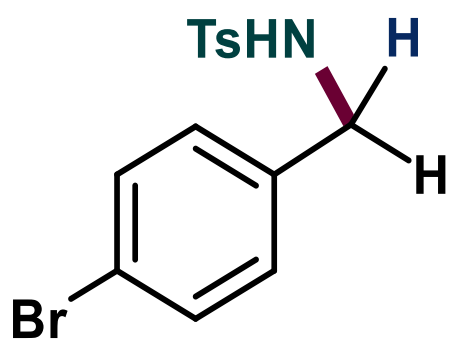

Known compound, see Ref. 34. 57.8 mg, 85% yield.
Purification conditions for column chromatography: EtOAc:hexanes 1:49 to 1:9
^{1}H NMR (500 MHz, $CDCl_3$): δ 7.70 (d, *J* = 8.2 Hz, 2H), 7.35 (d, *J* = 8.4 Hz, 2H), 7.27 (d, *J* = 7.7 Hz, 2H), 7.05 (d, *J* = 8.3 Hz, 2H), 5.19 (t, *J* = 6.3 Hz, 1H), 4.05 (d, *J* = 6.4 Hz, 2H), 2.43 (s, 3H).
^{13}C NMR (126 MHz, $CDCl_3$): δ 143.8, 136.9, 135.6, 131.8, 129.9, 129.7, 127.2, 121.8, 46.6, 21.7.

***N*-(4-chlorobenzyl)-4-methylbenzenesulfonamide (55)**

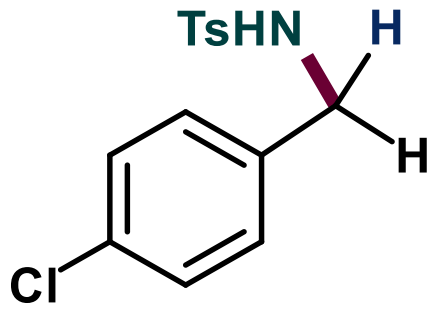

Known compound, see Ref. 32. 50.8 mg, 86% yield.
Purification conditions for column chromatography: EtOAc:hexanes 1:49 to 1:9
^{1}H NMR (500 MHz, $CDCl_3$): δ 7.69 (d, *J* = 8.3 Hz, 2H), 7.26 (d, *J* = 8.1 Hz, 2H), 7.18 (d, *J* = 8.4 Hz, 2H), 7.11 (d, *J* = 8.4 Hz, 2H), 5.31 (t, *J* = 6.3 Hz, 1H), 4.05 (d, *J* = 6.4 Hz, 2H), 2.42 (s, 3H).
^{13}C NMR (126 MHz, $CDCl_3$): δ 143.7, 136.8, 135.1, 133.6, 129.8, 129.3, 128.8, 127.2, 46.5, 21.6.

***N*-(4-fluorobenzyl)-4-methylbenzenesulfonamide (56)**

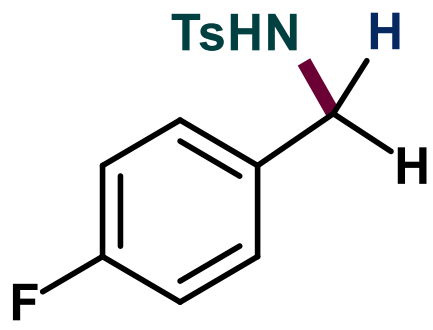

Known compound, see Ref. 32. 46.4 mg, 83% yield.
Purification conditions for column chromatography: EtOAc:hexanes 1:49 to 1:9
^{1}H NMR (500 MHz, $CDCl_3$): δ 7.72 (d, *J* = 7.9 Hz, 2H), 7.28 (d, *J* = 7.8 Hz, 2H), 7.17 – 7.14 (m, 2H), 6.92 (t, *J* = 8.1 Hz, 2H), 5.13 (t, *J* = 5.6 Hz, 1H), 4.07 (d, *J* = 6.1 Hz, 2H), 2.42 (s, 3H).
^{13}C NMR (126 MHz, $CDCl_3$): δ 162.4 (d, $J_{C\text{-}F}$ = 246.3 Hz), 143.7, 136.9, 132.3 (d, $J_{C\text{-}F}$ = 3.2 Hz), 129.8, 129.7 (d, $J_{C\text{-}F}$ = 8.2 Hz), 127.2, 115.6 (d, $J_{C\text{-}F}$ = 21.5 Hz), 46.6, 21.6.
^{19}F NMR (471 MHz, $CDCl_3$): δ -114.3 (s, 1F).

4-Methyl-*N*-(3-(trifluoromethyl)benzyl)benzenesulfonamide (57)

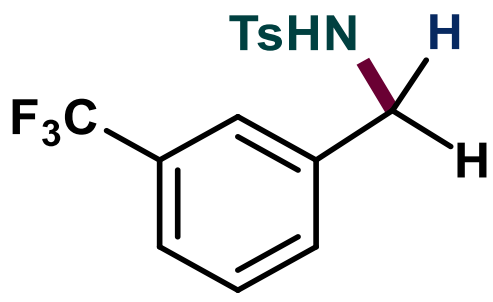

Known compound, see Ref. 32. 53.4 mg, 81% yield.
Purification conditions for column chromatography: EtOAc:hexanes 1:49 to 1:9
^{1}H NMR (500 MHz, $CDCl_3$): δ 7.71 (d, *J* = 8.1 Hz, 2H), 7.47 (d, *J* = 7.5 Hz, 1H), 7.43 – 7.41 (m, 1H), 7.39 – 7.37 (d, 2H) 7.26 (d, *J* = 8.0 Hz, 2H), 5.22 (t, *J* = 6.2 Hz, 1H), 4.19 (d, *J* = 6.4 Hz, 2H), 2.41 (s, 3H).
^{13}C NMR (126 MHz, $CDCl_3$): δ 143.9, 137.6, 136.9, 131.3, 131.0 (d, $J_{C\text{-}F}$ = 32.5 Hz), 129.9, 129.3, 127.2, 124.7 (dq, $J_{C\text{-}F}$ = 12.8 Hz, 3.7 Hz), 124.0 (d, $J_{C\text{-}F}$ = 272.4 Hz), 46.8, 21.6.
^{19}F NMR (471 MHz, $CDCl_3$): δ -62.8 (s, 3F).

***N*-(4-(6-methoxynaphthalen-2-yl)butan-2-yl)-4-methylbenzenesulfonamide (58)**

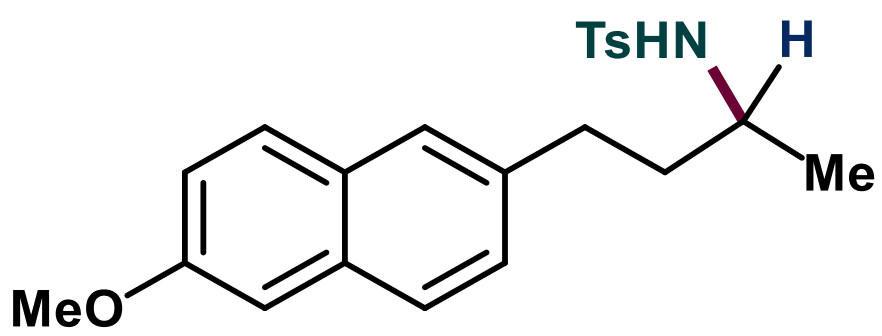

New compound, white solid, 31.4mg, 41% yield.
Purification conditions for column chromatography: EtOAc:hexanes 1:49 to 1:9
R_f = 0.53 (EtOAc:hexanes = 1:4 v/v)
^{1}H NMR (500 MHz, $CDCl_3$): δ 7.73 (d, *J* = 8.2 Hz, 2H), 7.62 (d, *J* = 8.5 Hz, 2H), 7.41 (s, 1H), 7.26 – 7.24 (m, 2H), 7.16 – 7.10 (m, 3H), 4.47 (d, *J* = 8.1 Hz, 1H), 3.91 (s, 3H), 3.42 – 3.33 (m, 1H), 2.75 – 2.64 (m, 2H), 2.40 (s, 3H), 1.75 (dd, *J* = 14.4 Hz, 7.4 Hz, 2H), 1.08 (d, *J* = 6.5 Hz, 3H).
^{13}C NMR (126 MHz, $CDCl_3$): δ 157.4, 143.4, 138.3, 136.6, 133.1, 129.8, 129.2, 129.0, 127.7, 127.2, 127.0, 126.4, 118.9, 105.8, 55.4, 49.8, 39.3, 31.9, 21.9, 21.7.

HR-MS: m/z calcd. $[C_{22}H_{25}NO_3SNa]^+$: 406.1453; found (ESI): 406.1445.

Methyl 2-(3-((4-methylphenyl)sulfonamido)-2-pentylcyclopentyl)acetate (59)

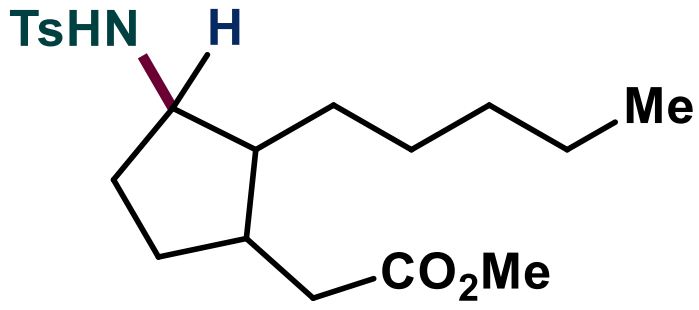

New compound, colorless oil, 45 mg, 59% yield.
Purification conditions for column chromatography: EtOAc:hexanes 1:49 to 1:9
R_f = 0.53 (EtOAc:hexanes = 1:4 v/v)
^{1}H NMR (500 MHz, $CDCl_3$): δ 7.75 (d, J = 7.8 Hz, 2H), 7.28 (d, J = 8.0 Hz, 2H), 4.87 (d, J = 7.8 Hz, 1H), 3.64 (s, 3H), 3.28 – 3.22 (m, 1H), 2.41 (s. 3H), 2.20 (dd, J = 15.4 Hz, 8.7 Hz, 1H), 1.85 – 1.81 (m, 1H), 1.77 – 1.68 (m, 2H), 1.45 – 1.41 (m, 1H), 1.37 – 1.30 (m, 2H), 1.24 – 1.16 (m, 5H), 1.12 – 1.02 (m, 4H), 0.83 (t, J = 7.2 Hz, 3H).
^{13}C NMR (126 MHz, $CDCl_3$): δ 173.4, 143.4, 138.1, 129.7, 127.3, 59.7, 51.7, 51.6, 39.7, 39.3, 32.6, 32.4, 32.2, 29.8, 29.5, 26.6, 22.6, 21.6, 14.2.
^{19}F NMR (471 MHz, $CDCl_3$): δ 50.5 (s, 1F).
HR-MS: m/z calcd. $[C_{20}H_{31}NO_4SNa]^+$: 404.1871; found (ESI): 404.1868.

Ethyl 3-((4-ethylphenyl)sulfonamido)hexanoate (60)

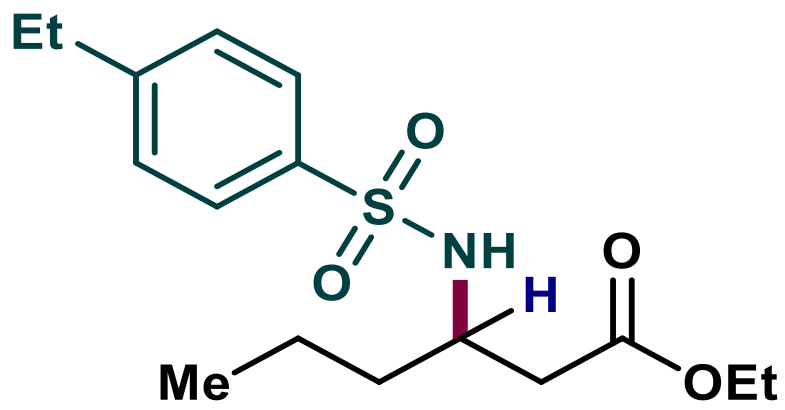

New compound, colorless oil, 54.3 mg, 83% yield.
Purification conditions for column chromatography: EtOAc:hexanes 1:49 to 1:9
R_f = 0.53 (EtOAc:hexanes = 1:4 v/v)
^{1}H NMR (500 MHz, $CDCl_3$): δ 7.78 (d, J = 8.3 Hz, 2H), 7.31 (d, J = 8.2 Hz, 2H), 5.23 (d, J = 9.0 Hz, 1H), 4.10 – 4.00 (m, 2H), 3.53 (qd, J = 10.8, 5.6 Hz, 1H), 2.71 (q, J = 7.6 Hz, 2H), 2.37 (qd, J = 16.1 Hz, 5.1 Hz, 2H), 1.48 – 1.38 (m, 2H), 1.29 – 1.18 Hz (m, 8H), 0.77 (t, J = 7.3 Hz, 3H).
^{13}C NMR (126 MHz, $CDCl_3$): δ 171.5, 149.6, 138.4, 128.6, 127.3, 60.8, 50.6, 38.8, 37.0, 28.9, 19.1, 15.3, 14.2, 13.7.
HR-MS: m/z calcd. $[C_{16}H_{25}NO_4SNa]^+$: 350.1402; found (ESI): 350.1399.

Ethyl 3-(phenylsulfonamido)hexanoate (61)

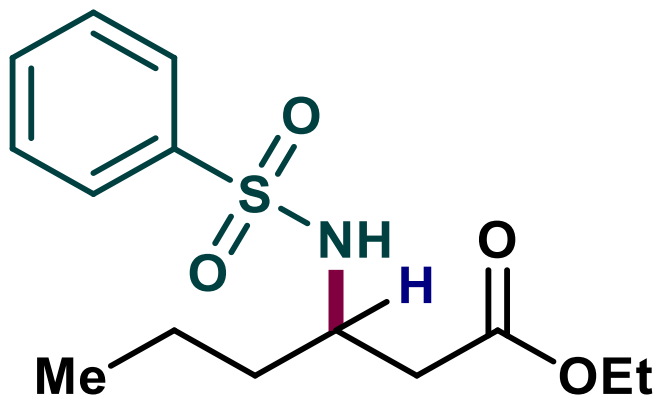

New compound, colorless oil, 122.7 mg (0.5 mmol scale), 82% yield.
Purification conditions for column chromatography: EtOAc:hexanes 1:49 to 1:9
R_f = 0.53 (EtOAc:hexanes = 1:4 v/v)
^{1}H NMR (500 MHz, $CDCl_3$): δ 7.88 (d, J = 7.4 Hz, 2H), 7.53 (dt, J = 32.0 Hz, 7.4 Hz, 3H), 5.29 (d, J = 9.0 Hz, 1H), 4.10 – 4.02 (m, 2H), 3.58 – 3.51 (m, 1H), 2.37 (qd, J = 16.1 Hz, 5.1 Hz, 2H), 1.50 – 1.37 (m, 2H), 1.31 – 1.14 (m, 5H), 0.78 (t, J = 7.3 Hz, 3H).
^{13}C NMR (126 MHz, $CDCl_3$): δ 171.5, 141.2, 132.7, 129.2, 127.1, 60.8, 50.7, 38.8, 37.0, 19.1, 14.2, 13.6.
HR-MS: m/z calcd. $[C_{14}H_{21}NO_4SNa]^+$: 322.1089; found (ESI): 322.1083.

Ethyl 3-((4-fluorophenyl)sulfonamido)hexanoate (62)

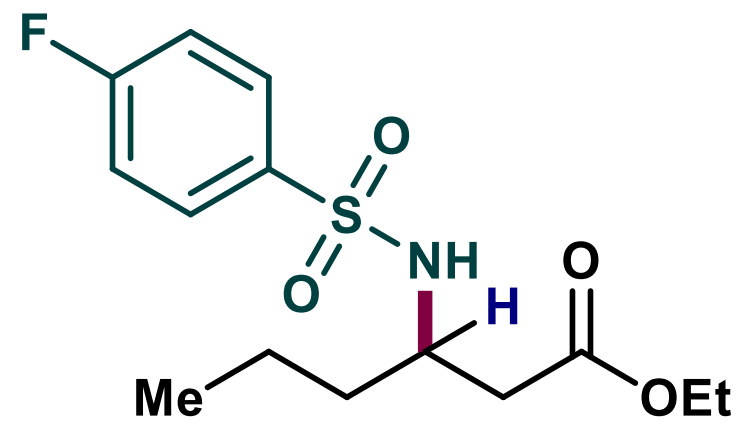

New compound, colorless oil, 36.2 mg, 57% yield.
Purification conditions for column chromatography: EtOAc:hexanes 1:49 to 1:9
R$_f$ = 0.53 (EtOAc:hexanes = 1:4 v/v)
^{1}H NMR (500 MHz, $CDCl_3$): δ 7.89 – 7.86 (m, 2H), 7.15 (t, J = 8.6 Hz, 2H), 5.51 (d, J = 9.0 Hz, 1H), 4.08 – 3.99 (m, 2H), 3.56 – 3.49 (m, 1H), 2.42 – 2.33 (m, 2H), 1.47 – 1.35 (m, 2H), 1.28 – 1.10 (m, 5H), 0.75 (t, J = 7.3 Hz, 3H).
^{13}C NMR (126 MHz, $CDCl_3$): δ 171.4, 165.0 (d, $J_{C\text{-}F}$ = 254.4 Hz), 137.3 (d, $J_{C\text{-}F}$ = 3.2 Hz), 129.8 (d, $J_{C\text{-}F}$ = 9.2 Hz), 116.2 (d, $J_{C\text{-}F}$ = 22.5 Hz), 60.8, 50.7, 39.2, 36.9, 18.9, 14.1, 13.5.
^{19}F NMR (471 MHz, $CDCl_3$): δ -105.6 (d, $J_{C\text{-}F}$ = 4.8 Hz, 1F).
HR-MS: m/z calcd. $[C_{14}H_{20}FNO_4SNa]^+$: 340.0995; found (ESI): 340.0991.

Ethyl 3-((4-chlorophenyl)sulfonamido)hexanoate (63)

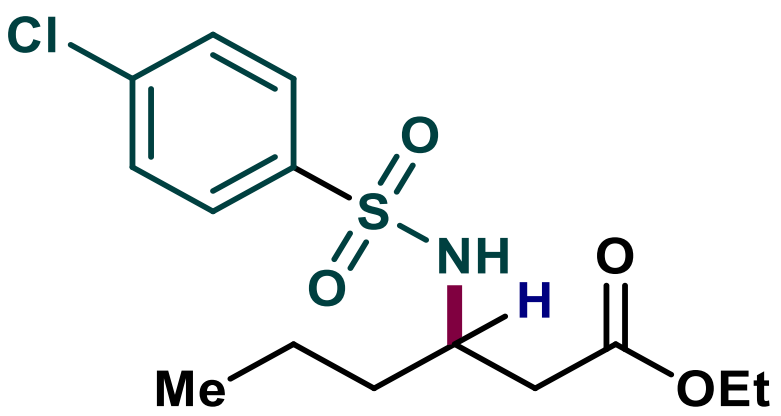

New compound, colorless oil, 41.4 mg, 77% yield.
Purification conditions for column chromatography: EtOAc:hexanes 1:49 to 1:9
R$_f$ = 0.53 (EtOAc:hexanes = 1:4 v/v)
^{1}H NMR (500 MHz, $CDCl_3$): δ 7.81 (d, J = 8.6 Hz, 2H), 7.46 (d, J = 8.6 Hz, 2H), 5.43 (d, J = 9.1 Hz, 1H), 4.10 – 4.00 (m, 2H), 3.58 – 3.51 (m, 1H), 2.43 – 2.34 (m, 2H), 1.50 – 1.37 (m, 2H), 1.29 – 1.14 (m, 5H), 0.79 (t, J = 7.3 Hz, 3H).
^{13}C NMR (126 MHz, $CDCl_3$): δ 171.4, 139.9, 139.1, 129.4, 128.6, 60.9, 50.8, 38.9, 37.0, 19.1, 14.2, 13.6.
HR-MS: m/z calcd. $[C_{14}H_{20}ClNO_4SNa]^+$: 356.0699; found (ESI): 356.0695.

Ethyl 3-(thiophene-2-sulfonamido)hexanoate (64)

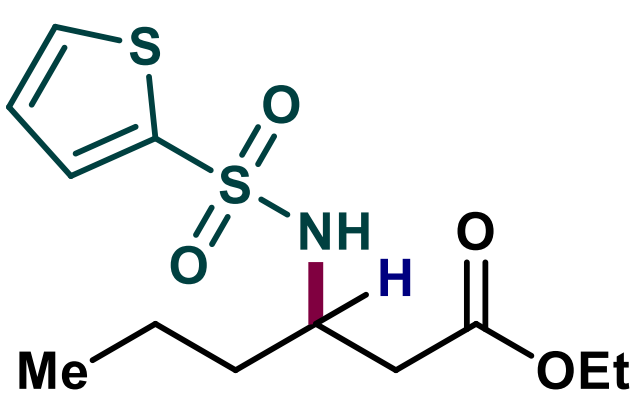

New compound, colorless oil, 22.6 mg, 37% yield.
Purification conditions for column chromatography: EtOAc:hexanes 1:49 to 1:9
R$_f$ = 0.53 (EtOAc:hexanes = 1:4 v/v)
^{1}H NMR (500 MHz, $CDCl_3$): δ 7.58 (ddd, J = 6.2 Hz, 4.4 Hz, 1.2 Hz, 2H), 7.05 (dd, J = 4.9 Hz, 3.8 Hz, 1H), 5.50 (d, J = 8.9 Hz, 1H), 4.10 – 4.04 (m, 1H), 3.64 – 3.57 (m, 1H), 2.46 – 2.36 (m, 2H), 1.51 – 1.39 (m, 2H), 1.33 – 1.17 (m, 5H), 0.79 (t, J = 7.3 Hz, 3H).
^{13}C NMR (126 MHz, $CDCl_3$): δ 171.4, 142.2, 132.1, 131.8, 127.4, 60.8, 50.9, 38.9, 36.6, 18.9, 14.2, 13.6.
HR-MS: m/z calcd. $[C_{12}H_{19}NO_4S_2Na]^+$: 328.0653; found (ESI): 328.0645.

Ethyl 3-(naphthalene-2-sulfonamido)hexanoate (65)

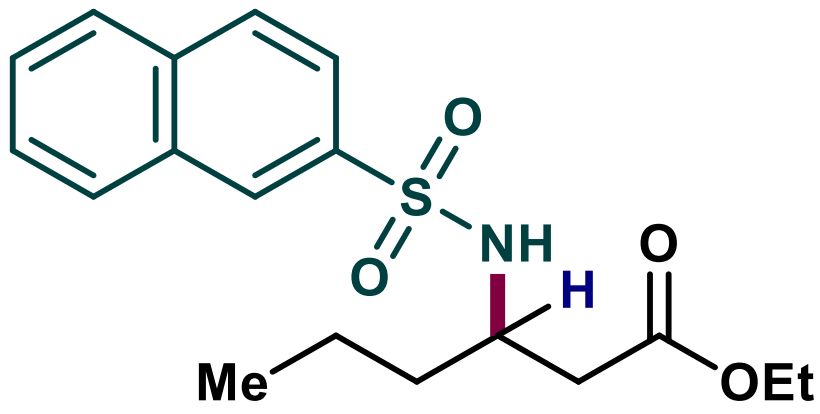

New compound, colorless oil, 34.2 mg, 49% yield.
Purification conditions for column chromatography: EtOAc:hexanes 1:49 to 1:9
R_f = 0.53 (EtOAc:hexanes = 1:4 v/v)
^{1}H NMR (500 MHz, $CDCl_3$): δ 8.46 (s, 1H), 7.94 (d, J = 8.6 Hz, 2H), 7.89 – 7.84 (m, 2H), 7.63 – 7.57 (m, 2H), 5.57 (d, J = 9.1 Hz, 1H), 4.04 – 3.93 (m, 2H), 3.65 – 3.59 (m, 1H), 2.37 (qd, J = 16.0, 5.3 Hz, 2H), 1.52 – 1.39 (m, 2H), 1.32 – 1.12 (m, 5H), 0.74 (t, J = 7.3 Hz, 3H).
^{13}C NMR (126 MHz, $CDCl_3$): δ 171.4, 138.0, 134.8, 132.2, 129.5, 129.3, 128.8, 128.2, 127.9, 127.6, 122.4, 60.7, 50.7, 39.0, 36.9, 19.0, 14.1, 13.6.
HR-MS: m/z calcd. $[C_{18}H_{23}NO_4SNa]^+$: 372.1245; found (ESI): 372.1239.

Ethyl 3-((4-(trifluoromethyl)phenyl)sulfonamido)hexanoate (66)

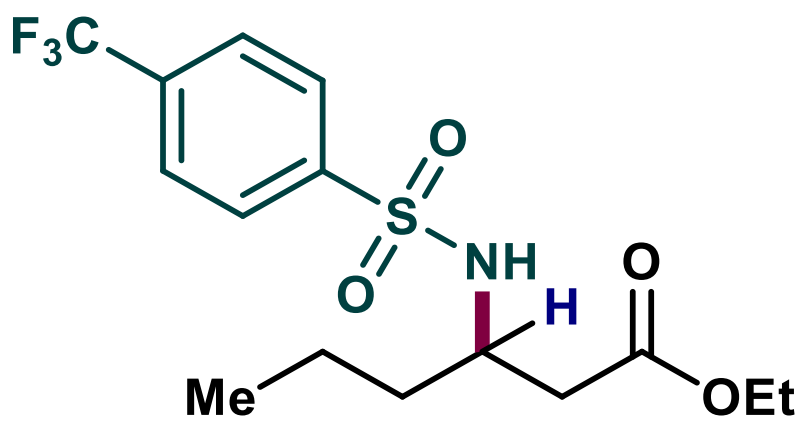

New compound, colorless oil, 30.9 mg, 42% yield.
Purification conditions for column chromatography: EtOAc:hexanes 1:49 to 1:9
R_f = 0.53 (EtOAc:hexanes = 1:4 v/v)
^{1}H NMR (500 MHz, $CDCl_3$): δ 8.01 (d, J = 8.2 Hz, 2H), 7.77 (d, J = 8.3 Hz, 2H), 5.45 (d, J = 9.2 Hz, 1H), 4.10 – 4.00 (m, 2H), 3.62 – 3.56 (m, 1H), 2.44 – 2.36 (m, 2H), 1.54 – 1.40 (m, 2H), 1.31 – 1.15 (m, 5H), 0.80 (t, J = 7.3 Hz, 3H).
^{13}C NMR (126 MHz, $CDCl_3$): δ 171.5, 145.0 (d, J_{C-F} = 1.1 Hz), 134.4 (d, J_{C-F} = 33.1 Hz), 127.6, 126.3 (q, J_{C-F} = 3.7 Hz), 123.4 (d, J_{C-F} = 272.9 Hz), 61.0, 51.0, 38.8, 37.0, 19.1, 14.2, 13.6.
^{19}F NMR (471 MHz, $CDCl_3$): δ -63.11 (s, 3F).
HR-MS: m/z calcd. $[C_{15}H_{20}F_3NO_4SNa]^+$: 390.0963; found (ESI): 390.0962.

5-(Dimethylamino)-*N*-(1-phenylethyl)naphthalene-2-sulfonamide (67)

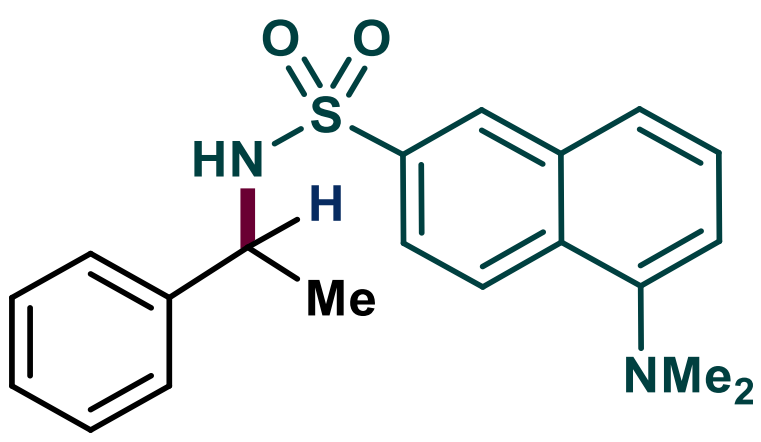

New compound, brownish oil, 10.7 mg, 15% yield.
Purification conditions for column chromatography: EtOAc:hexanes 1:49 to 3:7
R_f = 0.53 (EtOAc:hexanes = 1:4 v/v)
mp: 92 °C.
^{1}H NMR (500 MHz, $CDCl_3$): δ 8.41 (d, J = 8.5 Hz, 1H), 8.21 (d, J = 8.6 Hz, 1H), 8.09 (dd, J = 7.3 Hz, 1.1 Hz, 1H), 7.51 (dd, J = 8.4 Hz, 7.7 Hz, 1H), 7.36 (dd, J = 8.4 Hz, 7.4 Hz, 1H), 7.14 (d, J = 7.5 Hz, 1H), 7.01 – 6.93 (m, 3H), 6.87 – 6.85 (m, 2H), 4.89 (d, J = 7.0 Hz, 1H), 4.45 (p, J = 6.8 Hz, 1H), 2.87 (s, 6H), 1.35 (d, J = 6.9 Hz, 3H).
^{13}C NMR (126 MHz, $CDCl_3$): δ 152.0, 141.5, 135.3, 130.3, 129.84, 129.82, 129.7, 128.4, 128.2, 127.4, 126.0, 123.2, 118.8, 115.1, 54.0, 45.6, 23.6.
HR-MS: m/z calcd. $[C_{20}H_{22}N_2O_2SNa]^+$: 377.1300; found (ESI): 377.1301.

Ethyl 3-aminohexanoate (68)

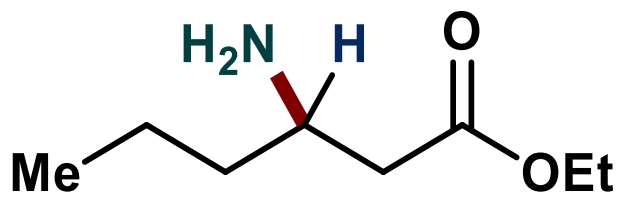

Known compound, see Ref. 35. 50 mg (0.31 mmol scale), 84% yield.
Purification conditions for column chromatography: MeOH:CH_2Cl_2 1:49 to 1:9
^{1}H NMR (500 MHz, $CDCl_3$): δ 4.11 – 4.03 (m, 2H), 3.11 (s, 1H), 2.41 – 2.30 (m, 1H), 2.20 – 2.12 (m, 1H), 1.45 (brs, 2H), 1.35 – 1.25 (m, 4H), 1.22 – 1.16 (m, 3H), 0.86 – 0.83 (m, 3H).
^{13}C NMR (126 MHz, $CDCl_3$): δ 172.6, 60.3, 48.0, 42.7, 39.8, 19.2, 14.2, 14.0.

4-Propylazetidin-2-one (69)

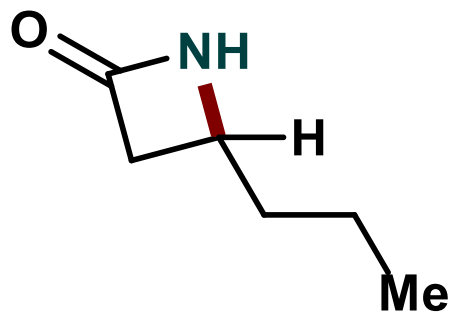

Known compound, see Ref. 36. 50 mg (1 mmol scale), 54% yield.
Purification conditions for column chromatography: EtOAc:CH_2Cl_2 1:49 to 1:9
^{1}H NMR (500 MHz, $CDCl_3$): δ 5.94 (brs, 1H), 3.62 (qd, *J* = 6.9 Hz, 2.4 Hz, 1H), 3.05 (ddd, *J* = 14.8 Hz, 4.9 Hz, 2.1 Hz, 1H), 2.56 (d, *J* = 14.7 Hz, 1H), 1.63 – 1.54 (m, 2H), 1.40 – 1.33 (m, 2H), 0.95 (t, *J* = 7.4 Hz, 3H).
^{13}C NMR (126 MHz, $CDCl_3$): δ 168.5, 48.2, 43.6, 37.7, 19.7, 14.0.

(*E*)-4-methyl-*N*-(8-oxonon-6-en-4-yl)benzenesulfonamide (70)

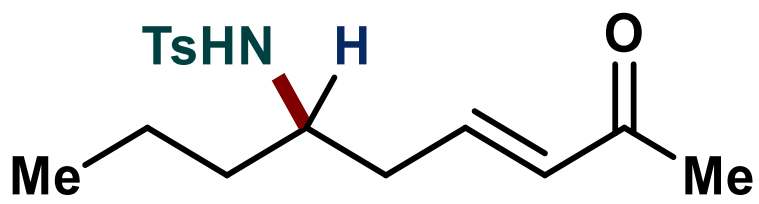

New compound, colorless oil, 122 mg (5 mmol scale), 79% yield.
Purification conditions for column chromatography: EtOAc:hexanes 1:49 to 1:9
R_f = 0.53 (EtOAc:hexanes = 1:4 v/v)
^{1}H NMR (500 MHz, $CDCl_3$): δ 7.74 (d, *J* = 8.2 Hz, 2H), 7.29 (d, *J* = 8.1 Hz, 2H), 6.64 – 6.57 (m, 1H), 5.97 (d, *J* = 15.9 Hz, 1H), 4.82 – 4.76 (m, 1H), 3.40 – 3.33 (m, 1H), 2.42 (s, 3H), 2.40 – 2.34 (m, 1H), 2.31 – 2.24 (m, 1H), 2.16 (s, 3H), 1.41 – 1.32 (m, 2H), 1.23 – 1.09 (m, 2H), 0.76 (t, *J* = 7.3 Hz, 3H).
^{13}C NMR (126 MHz, $CDCl_3$): δ 198.4, 143.6, 143.1, 138.1, 134.1, 129.8, 127.1, 53.1, 38.4, 37.3, 27.0, 21.6, 18.8, 13.7.
HR-MS: m/z calcd. $[C_{16}H_{23}NO_3SNa]^+$: 332.1296; found (ESI): 332.1292.

***N*-(1-hydroxyhexan-3-yl)-4-methylbenzenesulfonamide (71)**

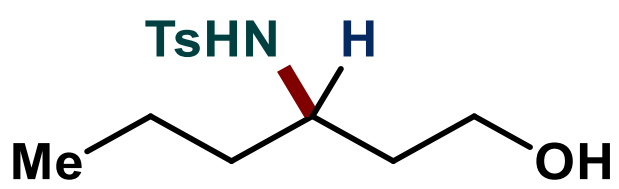

Known compound, see Ref. 3. 131.6 mg (0.5 mmol scale), 97% yield.
Purification conditions for column chromatography: EtOAc:hexanes 1:49 to 1:4
^{1}H NMR (500 MHz, $CDCl_3$): δ 7.75 (d, *J* = 8.0 Hz, 2H), 7.28 (d, *J* = 8.0 Hz, 2H), 5.28 – 5.22 (m, 1H), 3.82 – 3.78 (m, 1H), 3.60 – 3.58 (m, 1H), 3.36 (brs, 1H), 2.70 (brs, 1H), 2.40 (s, 3H), 1.73 – 1.69 (m, 1H), 1.44 – 1.38 (m, 1H), 1.33 – 1.23 (m, 2H), 1.14 – 1.00 (m, 2H), 0.69 (t, *J* = 7.3 Hz, 3H).
^{13}C NMR (126 MHz, $CDCl_3$): δ 143.4, 138.1, 129.7, 127.1, 59.0, 51.3, 37.7, 37.2, 21.6, 18.7, 13.8.

2-Propyl-1-tosylazetidine (72)

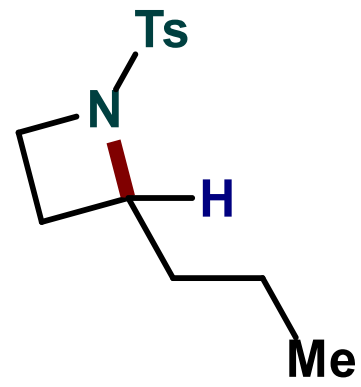

New compound, colorless oil, 55 mg (0.22 mmol scale), 99% yield.
Purification conditions for column chromatography: EtOAc:hexanes 1:49 to 1:9
R$_f$ = 0.53 (EtOAc:hexanes = 1:4 v/v)
^{1}H NMR (500 MHz, $CDCl_3$): δ 7.70 (d, *J* = 8.1 Hz, 2H), 7.35 (d, *J* = 8.0 Hz, 2H), 3.86 – 3.80 (m, 1H), 3.68 – 3.64 (m, 1H), 3.49 (q, *J* = 8.6 Hz, 1H), 2.44 (s, 3H), 1.95 – 1.81 (m, 3H), 1.68 – 1.60 (m, 1H), 1.36 – 1.22 (m, 2H), 0.89 (t, *J* = 7.4 Hz, 3H).
^{13}C NMR (126 MHz, $CDCl_3$): δ 143.9, 132.2, 129.8, 128.4, 64.2, 47.7, 38.3, 22.4, 21.7, 17.7, 14.0
HR-MS: m/z calcd. $[C_{13}H_{19}NO_2SNa]^+$: 276.1034; found (ESI): 276.1032.

3-((4-methylphenyl)sulfonamido)hexanoic acid (73)

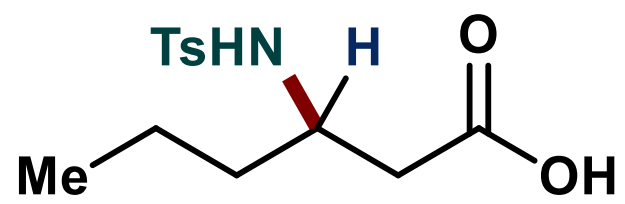

New compound, white solid, 56.5 mg, 99% yield.
Purification conditions for column chromatography: EtOAc:CH_2Cl_2 1:49 to 3:7
R$_f$ = 0.53 (EtOAc:hexanes = 1:4 v/v)
mp: 92 °C.
^{1}H NMR (500 MHz, $CDCl_3$): δ 7.76 (d, *J* = 8.1 Hz, 2H), 7.30 (d, *J* = 8.0 Hz, 2H), 5.34 (d, *J* = 9.0 Hz, 1H) 3.56 – 3.50 (m, 1H), 2.48 – 2.47 (m, 2H), 2.42 (s, 3H), 1.51 – 1.41 (m, 2H), 1.33 – 1.25 (m, 1H), 1.20 – 1.13 (m, 1H), 0.79 (t, *J* = 7.3 Hz, 3H).
^{13}C NMR (126 MHz, $CDCl_3$): δ 176.6, 143.7, 138.0, 129.9, 127.2, 50.3, 38.7, 36.8, 21.7, 19.1, 13.6.
HR-MS: m/z calcd. $[C_{13}H_{19}NO_4SNa]^+$: 308.0933; found (ESI): 308.0929.

4-Methyl-*N*-(4-oxo-4-(3-(trifluoromethyl)-5,6-dihydro-[1,2,4]triazolo[4,3-a]pyrazin-7(8*H*)-yl)-1-(2,4,5-trifluorophenyl)butan-2-yl)benzenesulfonamide (74)

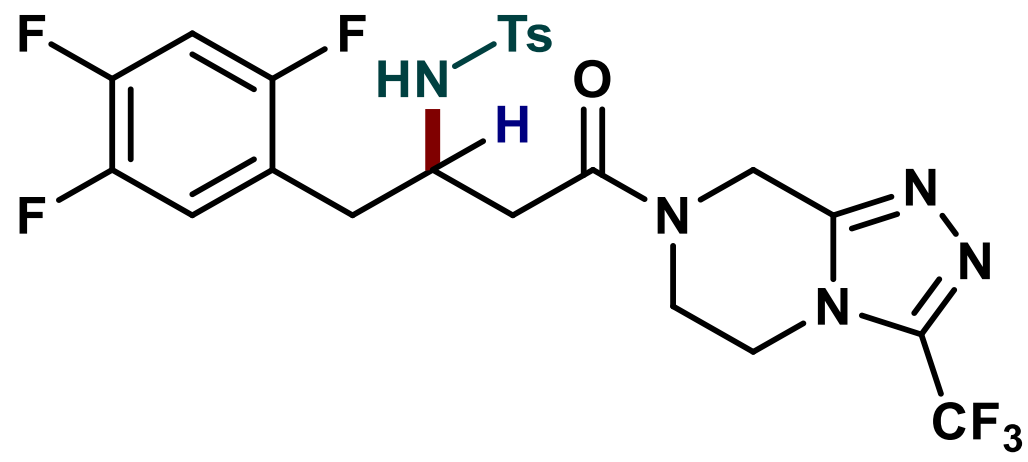

Known compound, see Ref. 37, 38. 443 mg (1 mmol scale), 79% yield.
Purification conditions for column chromatography: EtOAc:CH_2Cl_2 1:49 to 3:7
^{1}H NMR (500 MHz, DMSO-*d$_6$*): δ 7.80 (brs, 1H), 7.36 (t, *J* = 9.6 Hz, 2H), 7.22 – 7.11 (m, 4H), 5.00 – 4.92 (m, 1H), 4.78 (s. 1H), 4.25 – 3.69 (m, 6H), 2.81 – 2.53 (m, 4H), 2.32 – 2.30 (m, 3H).
^{13}C NMR (126 MHz, DMSO-*d$_6$*): δ 168.9, 168.8, 150.9, 150.8, 142.3, 142.2, 138.33, 138.30, 129.2, 129.1, 125.9, 119.6, 117.4, 105.6, 105.4, 105.4, 105.2, 59.8, 51.332, 51.330, 51.32, 51.31, 51.28, 51.27, 43.6, 43.0, 42.1, 41.2, 38.3, 37.4, 33.4, 33.2, 20.8, 14.1.
^{19}F NMR (471 MHz, DMSO-*d$_6$*): δ -61.9 (d, *J* = 19.8 Hz, 3F), -118.7 (ddd, *J* = 154.9 Hz, 15.3 Hz, 2.4 Hz, 1F), -137.2 (t, *J* = 22.2 Hz, 1F), -144.16 (ddd, *J* = 38.5 Hz, 22.7 Hz, 15.5 Hz, 1F).
HR-MS: m/z calcd. $[C_{23}H_{22}F_6N_5O_3S]^+$: 562.1348; found (ESI): 562.1349.
§ Note: The compound was observed as a mixture of rotamers. Furthermore, with described issue, 13C NMR spectra shows an excess number of peaks.

3-Amino-1-(3-(trifluoromethyl)-5,6-dihydro-[1,2,4]triazolo[4,3-a]pyrazin-7(8*H*)-yl)-4-(2,4,5-

trifluorophenyl)butan-1-one (75)

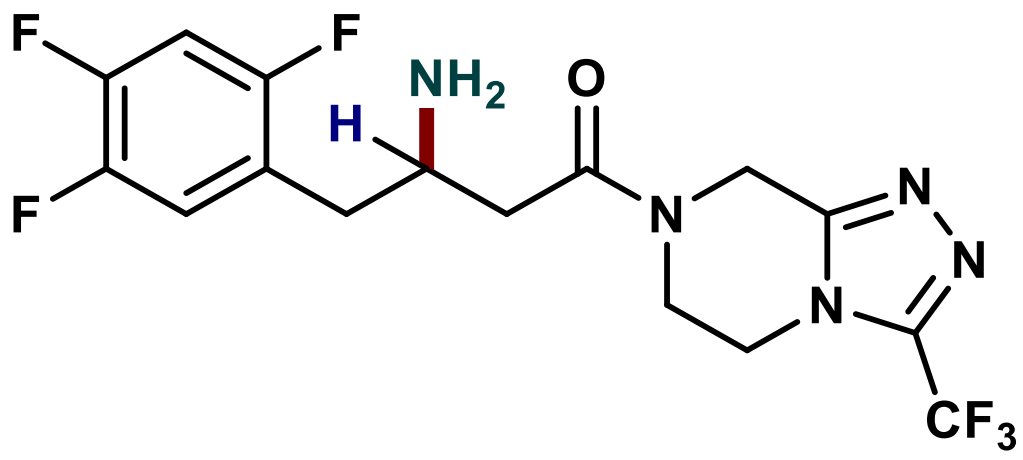

Known compound, see Ref. 39, 40. 34.2 mg (0.1 mmol scale), 84% yield.
Purification conditions for column chromatography: MeOH:CH_2Cl_2 1:49 to 1:9
^{1}H NMR (500 MHz, $CDCl_3$): δ 7.04 (dd, J = 16.3, 8.8 Hz, 1H), 6.88 (dd, J = 15.8, 8.7 Hz, 1H), 5.07 – 4.85 (m, 2H), 4.20 – 3.93 (m, 4H), 3.53 (s, 1H), 2.78 – 2.74 (m, 1H), 2.66 – 2.62 (m, 1H), 2.54 – 2.41 (m, 2H), 1.79 (s, 2H).
^{13}C NMR (126 MHz, $CDCl_3$): δ 170.34, 170.1, 155.74 (dd, $J_{C\text{-}F}$ = 243.5 Hz, 8.0 Hz), 150.3, 149.7, 149.7 – 149.1 (m), 147.16 (dd, $J_{C\text{-}F}$ = 22.8 Hz, 12.7 Hz), 147.10 (t, $J_{C\text{-}F}$ = 12.7 Hz), 145.1(dd, $J_{C\text{-}F}$ = 12.4, 3.0 Hz), 143.6 – 142.5 (m), 121.8 (d, $J_{C\text{-}F}$ = 18.8 Hz), 121.1, 118.6 (dd, $J_{C\text{-}F}$ = 18.1 Hz, 2.5 Hz), 117.9 (q, $J_{C\text{-}F}$ = 268.3 Hz). 105.0 (dd, $J_{C\text{-}F}$ = 18.1 Hz, 2.5 Hz), 54.8 (dd, $J_{C\text{-}F}$ = 18.3 Hz, 2.2 Hz), 48.3, 43.4, 42.9, 41.9, 41.2, 41.0, 39.8 (d, $J_{C\text{-}F}$ = 16.0 Hz), 38.5, 37.5, 35.8, 14.8.
^{19}F NMR (471 MHz, $CDCl_3$): δ -63.3 (d, J = 21.6 Hz, 3F), -119.2 (d, J = 14.2 Hz, 1F), -136.7 (dd, J = 42.0 Hz, 21.4 Hz, 1F), -143.6 (dd, J = 38.5 Hz, 22.4 Hz, 1F).
HR-MS: m/z calcd. $[C_{16}H_{16}F_6N_5O]^+$: 408.1259; found (ESI): 408.1257.

Methyl (*Z*)-3-(methylsulfonamido)-4-(2,4,5-trifluorophenyl)but-2-enoate (12a)

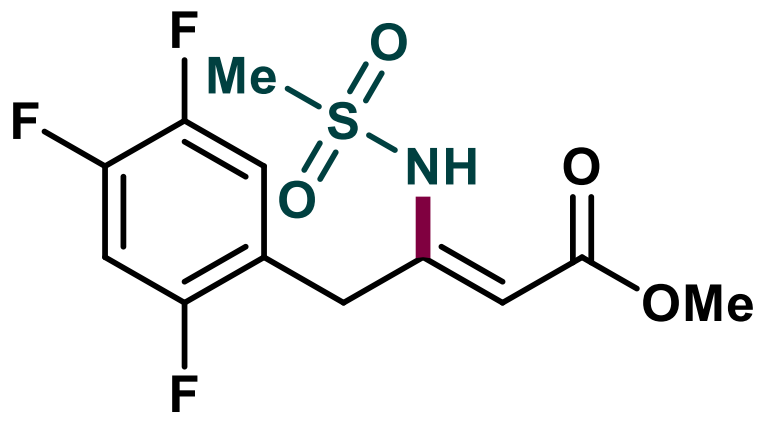

New compound, white solid, 55.6 mg, 86% yield.
Purification conditions for column chromatography: EtOAc:hexanes 1:49 to 1:9
R_f = 0.53 (EtOAc:hexanes = 1:4 v/v)
^{1}H NMR (500 MHz, $CDCl_3$): δ 10.9 (s, 1H), 7.12 (dd, J = 16.8 Hz, 8.7 Hz, 1H), 6.95 (td, J = 9.4, 7.0 Hz, 1H), 4.83 (s ,1H), 3.89 (s, 2H), 3.69 (s, 3H), 3.09 (s, 3H).
^{13}C NMR (126 MHz, $CDCl_3$): δ 169.3, 156.1 (ddd, $J_{C\text{-}F}$ = 246.0 Hz, 9.2 Hz, 2.5 Hz), 153.5, 149.6 (ddd, $J_{C\text{-}F}$ = 251.9 Hz, 14.2 Hz, 12.5 Hz), 146.9 (ddd, $J_{C\text{-}F}$ = 245.8 Hz, 12.6 Hz, 3.6 Hz), 119.1 (dd, $J_{C\text{-}F}$ = 20.0 Hz, 4.6 Hz), 106.0 (dd, $J_{C\text{-}F}$ = 28.0 Hz, 20.9 Hz), 98.1, 51.7, 43.0, 30.9 (d, $J_{C\text{-}F}$ = 2.4 Hz).
^{19}F NMR (471 MHz, $CDCl_3$): δ -118.1 (dd, J = 15.3 Hz, 3.9 Hz, 1F), -133.7 (dd, J = 21.4 Hz, 3.8 Hz, 1F), -141.7 (dd, J = 21.4 Hz, 15.3Hz, 1F).
HR-MS: m/z calcd. $[C_{12}H_{12}F_3NO_4SNa]^+$: 346.0337; found (ESI): 346.0335.

Ethyl 3-((3,5-bis(trifluoromethyl)phenyl)amino)hexanoate (1a)

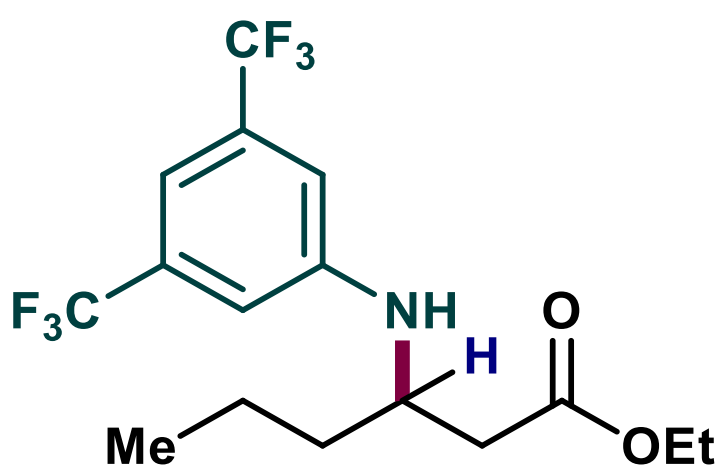

New compound, colorless oil, 6.7 mg, 9% yield.
Purification conditions for column chromatography: EtOAc:hexanes 1:49 to 1:9
R_f = 0.53 (EtOAc:hexanes = 1:4 v/v)
^{1}H NMR (500 MHz, $CDCl_3$): δ 7.12 (s, 1H), 6.95 (s, 2H), 4.32 (d, J = 9.4 Hz, 1H), 4.16 – 4.10 (m, 2H), 3.89 –

3.83 (m, 1H), 2.54 (qd, J = 15.3, 5.7 Hz, 2H), 1.60 (dd, J = 15.2, 7.2 Hz, 2H), 1.49 – 1.35 (m, 2H), 1.23 (t, J = 7.1 Hz, 3H), 0.94 (t, J = 7.3 Hz, 3H).
^{13}C NMR (126 MHz, $CDCl_3$): δ 171.6, 148.2, 132.7 (q, $J_{C\text{-}F}$ = 32.6 Hz), 123.7 (d, $J_{C\text{-}F}$ = 272.7 Hz), 112.3, 110.3, 60.9, 50.2, 39.2, 37.2, 19.5, 14.2, 14.0.
^{19}F NMR (471 MHz, $CDCl_3$): δ -63.2 (s, 1F).
HR-MS: m/z calcd. $[C_{16}H_{20}F_6NO_2]^+$: 372.1398; found (ESI): 372.1398.

Ethyl 3-benzamidohexanoate (1b)

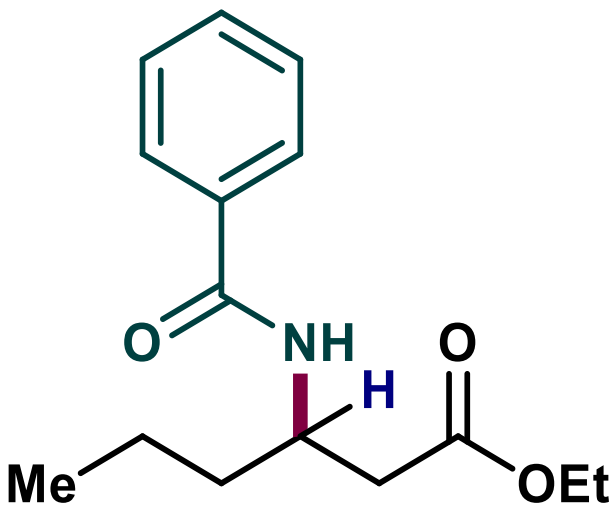

Known compound, see Ref. 41. 2.6 mg, 5% yield.
Purification conditions for column chromatography: EtOAc:hexanes 1:49 to 1:9
^{1}H NMR (500 MHz, $CDCl_3$): δ 7.78 (d, J = 7.5 Hz, 2H), 7.49 (t, J = 7.1, 1H), 7.43 (t, J = 7.6 Hz, 2H), 6.94 (d, J = 8.7 Hz, 1H), 4.50 – 4.44 (m, 1H), 4.21 – 4.13 (m, 2H), 2.64 (qd, J = 16.0 Hz, 4.8 Hz, 2H), 1.72 – 1.64 (m, 1H), 1.61 – 1.53 (m, 1H), 1.42 (dq, J = 15.0, 7.5 Hz, 2H), 1.27 (t, J = 7.1 Hz, 3H), 0.94 (t, J = 7.3 Hz, 3H).
^{13}C NMR (126 MHz, $CDCl_3$): δ 172.5, 166.8, 134.8, 131.5, 128.7, 127.0, 60.8, 46.3, 38.5, 36.5, 19.7, 14.3, 14.0.

Ethyl 3-(((benzyloxy)carbonyl)amino)hexanoate (1c)

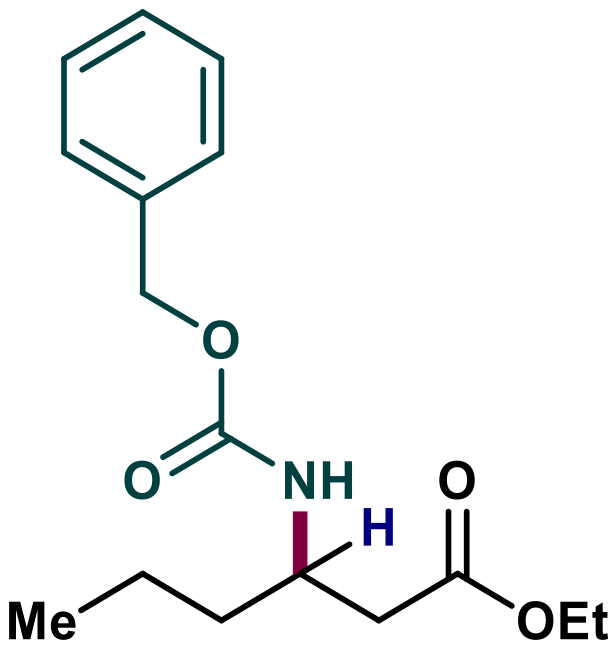

Known compound, see Ref. 42. 4.7 mg, 8% yield.
Purification conditions for column chromatography: EtOAc:hexanes 1:49 to 1:9
^{1}H NMR (500 MHz, $CDCl_3$): δ 7.35 – 7.26 (m, 5H), 5.20 (d, J = 8.4 Hz, 1H), 5.09 (s, 2H), 4.12 (q, J = 7.1 Hz, 2H), 4.03 – 3.96 (m, 1H), 2.52 (qd, J = 15.7 Hz, 5.3 Hz, 2H), 1.55 – 1.45 (m, 2H), 1.43 – 1.33 (m, 2H), 1.24 (t, J = 7.1 Hz, 3H), 0.91 (t, J = 7.2 Hz, 3H).
^{13}C NMR (126 MHz, $CDCl_3$): δ 171.7, 156.0, 136.7, 128.6, 128.2, 66.7, 60.7, 48.1, 39.2, 36.8, 19.5, 14.3, 13.9.

Ethyl 3-((((9*H*-fluoren-9-yl)methoxy)carbonyl)amino)hexanoate (1d)

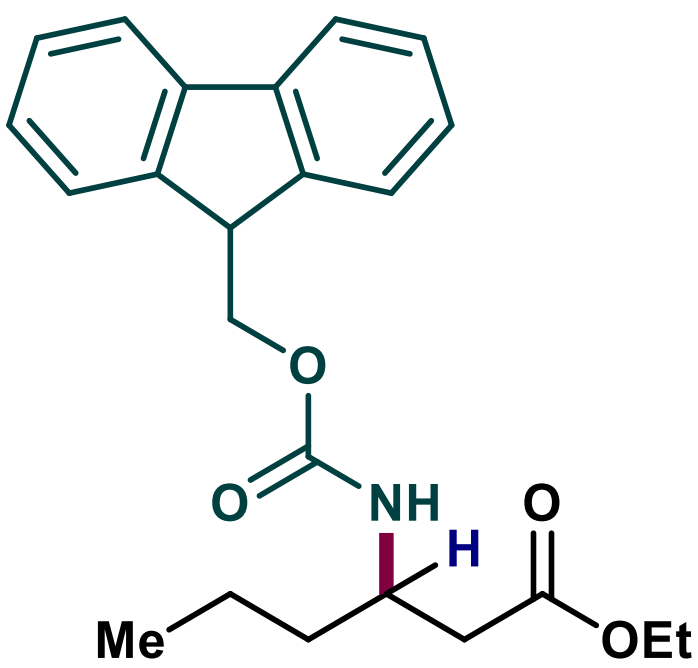

New compound, white soild, 5.3 mg, 7% yield.
Purification conditions for column chromatography: EtOAc:hexanes 1:49 to 1:9
R_f = 0.53 (EtOAc:hexanes = 1:4 v/v)
^{1}H NMR (500 MHz, $CDCl_3$): δ 7.76 (d, *J* = 7.5 Hz, 2H), 7.60 (d, *J* = 7.5 Hz, 2H), 7.40 (t, *J* = 7.4 Hz, 2H), 7.32 (t, *J* = 7.3 Hz, 2H), 5.27 (d, *J* = 9.0 Hz, 1H), 4.43 – 4.36 (m, 2H), 4.23 (t, *J* = 6.9 Hz, 1H), 4.16 (q, *J* = 7.0 Hz, 2H), 4.04 – 3.98 (m, 1H), 2.54 (qd, *J* = 15.7 Hz, 5.3 Hz, 2H), 1.58 – 1.47 (m, 2H), 1.42 – 1.35 (m, 2H), 1.26 (t, *J* = 7.1 Hz, 3H), 0.94 (t, *J* = 7.2 Hz, 3H).
^{13}C NMR (126 MHz, $CDCl_3$): δ 171.7, 155.9, 144.0, 141.4, 127.7, 127.1, 125.2, 120.0, 66.6, 60.6, 48.0, 47.4, 39.2, 36.7, 19.4, 14.3, 13.9.
HR-MS: m/z calcd. $[C_{23}H_{27}NO_4Na]^+$: 404.1838; found (ESI): 404.1835.

7. NMR Spectra of the Products

^{1}H NMR spectra (**1**)

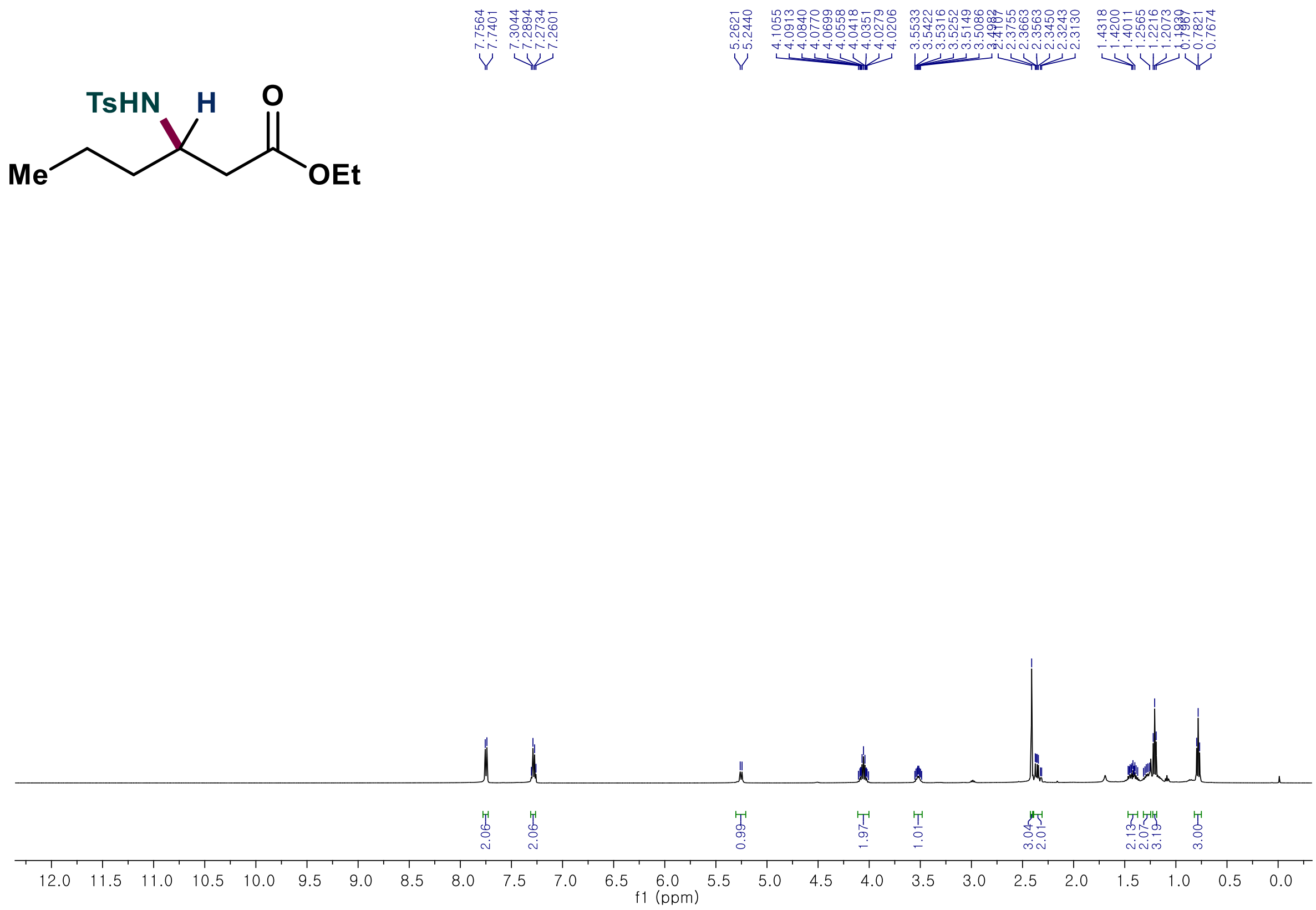

^{13}C NMR spectra (**1**)

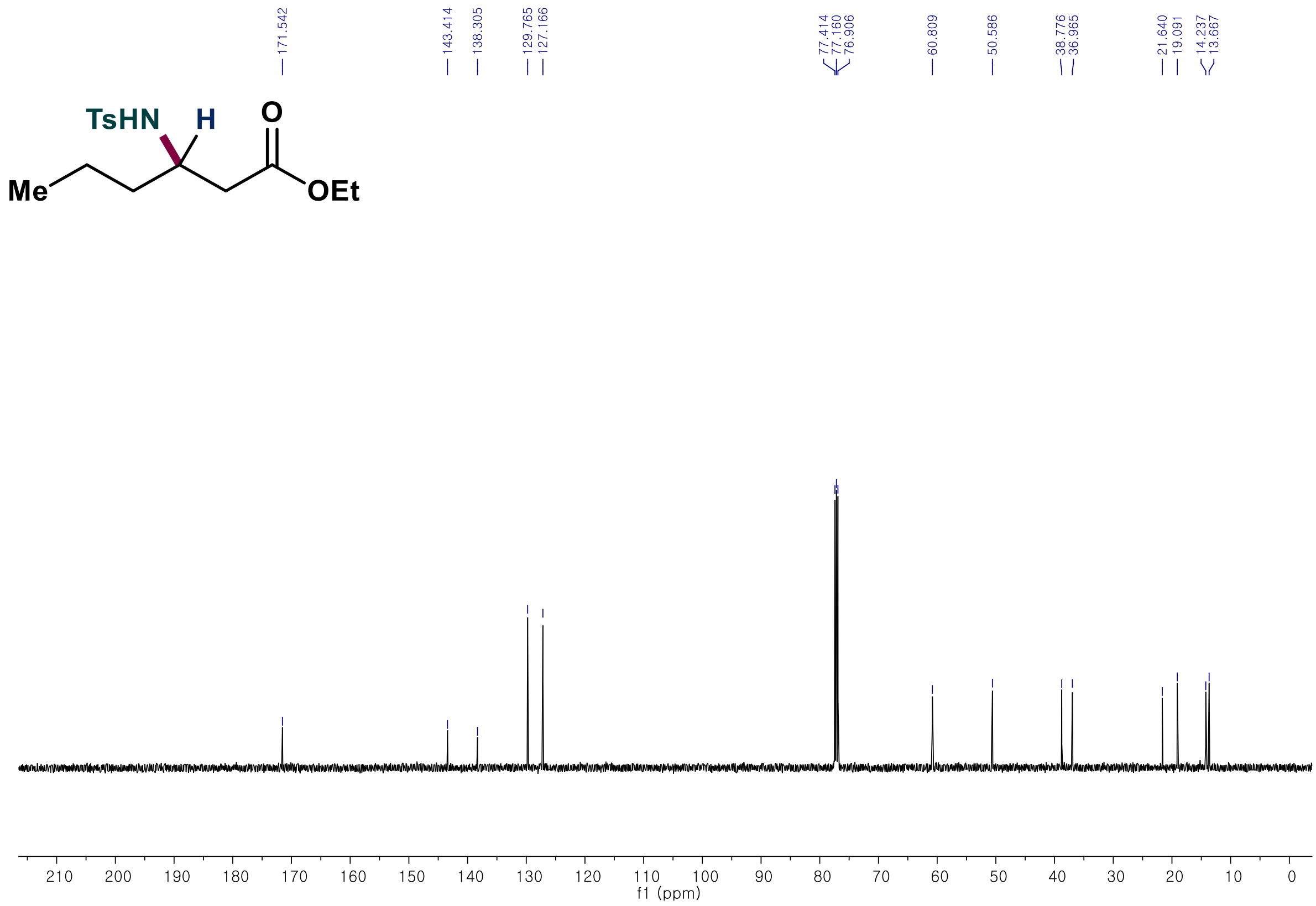

^{1}H NMR spectra (**2**)

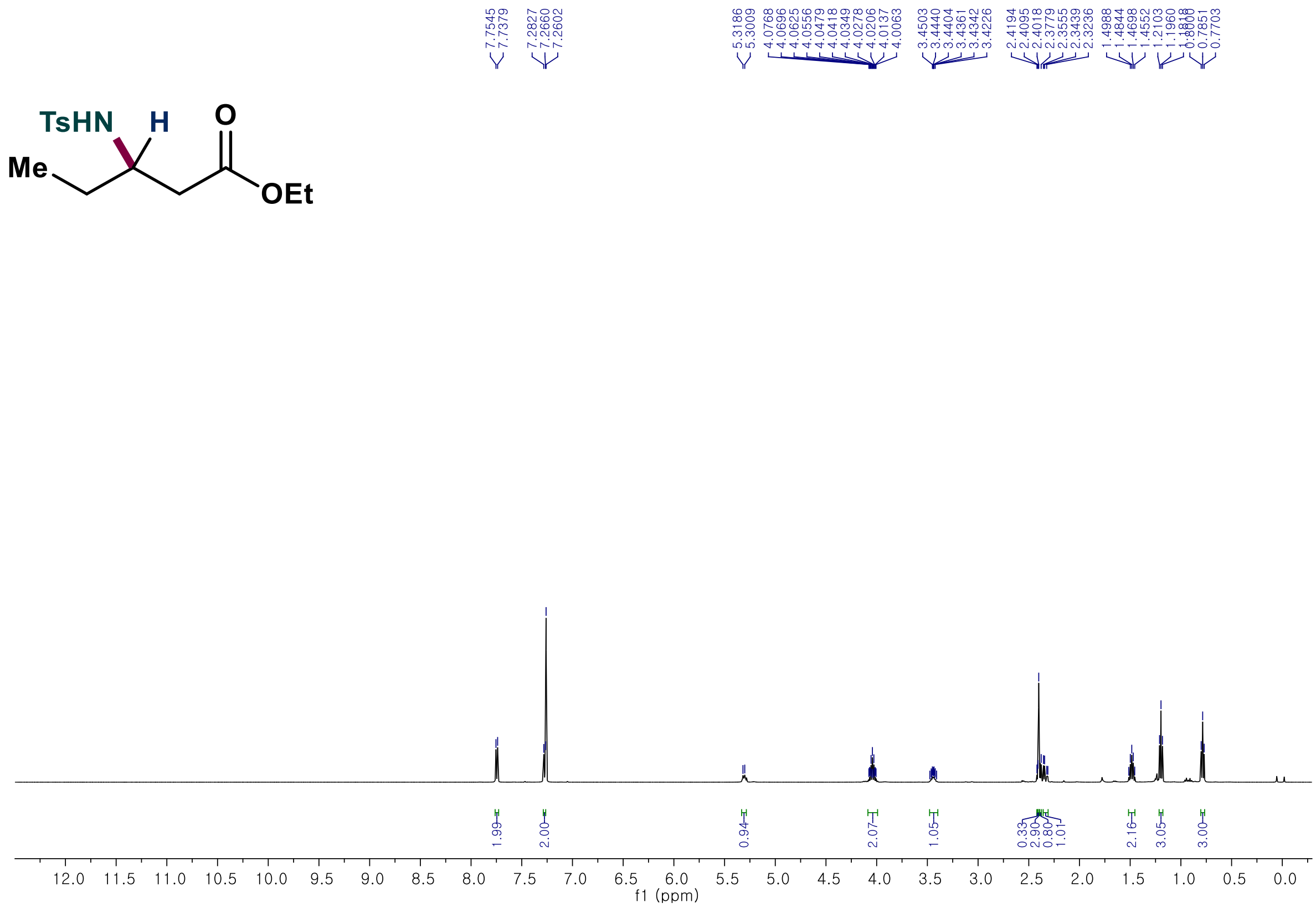

^{13}C NMR spectra (**2**)

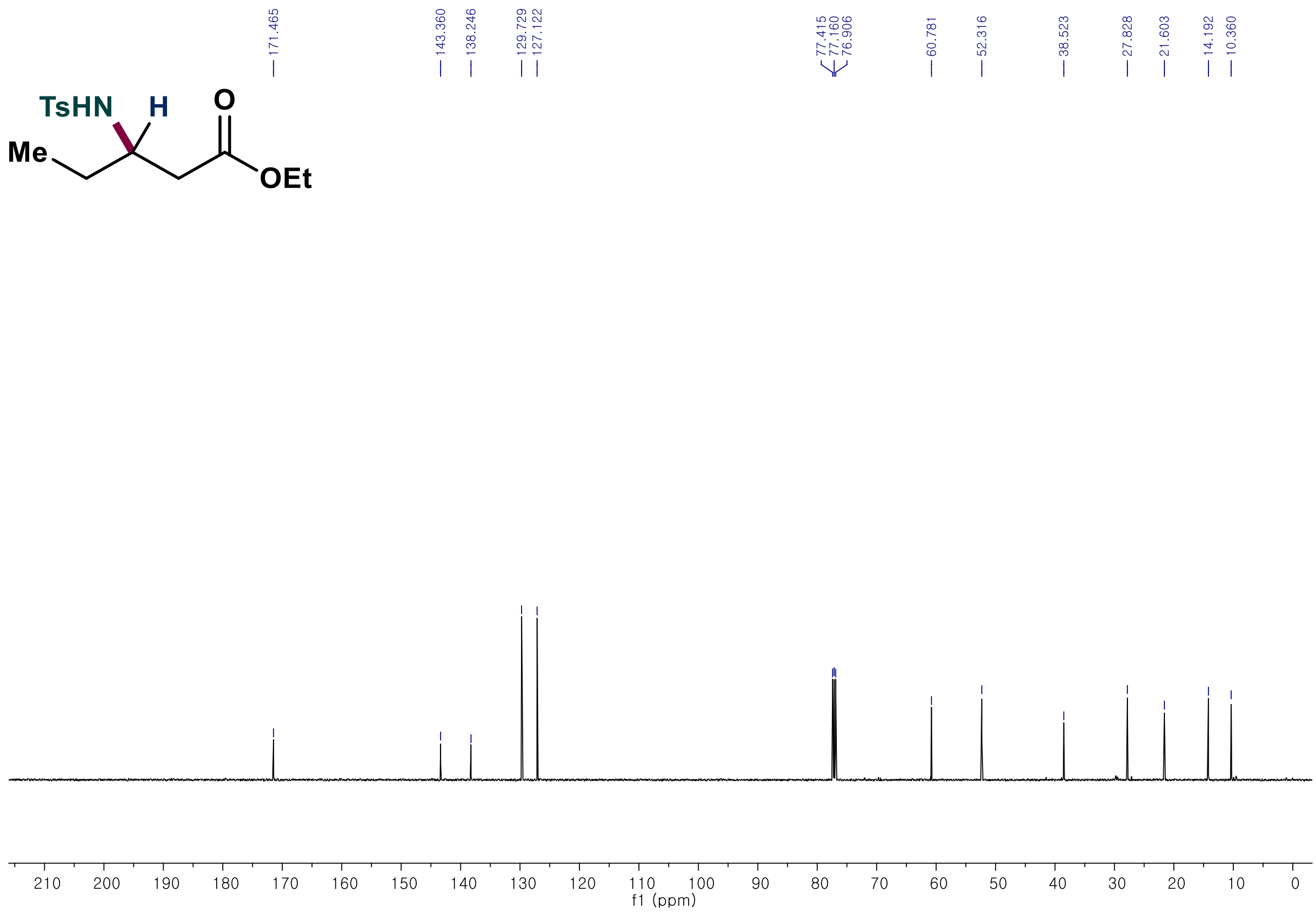

^{1}H NMR spectra (**3**)

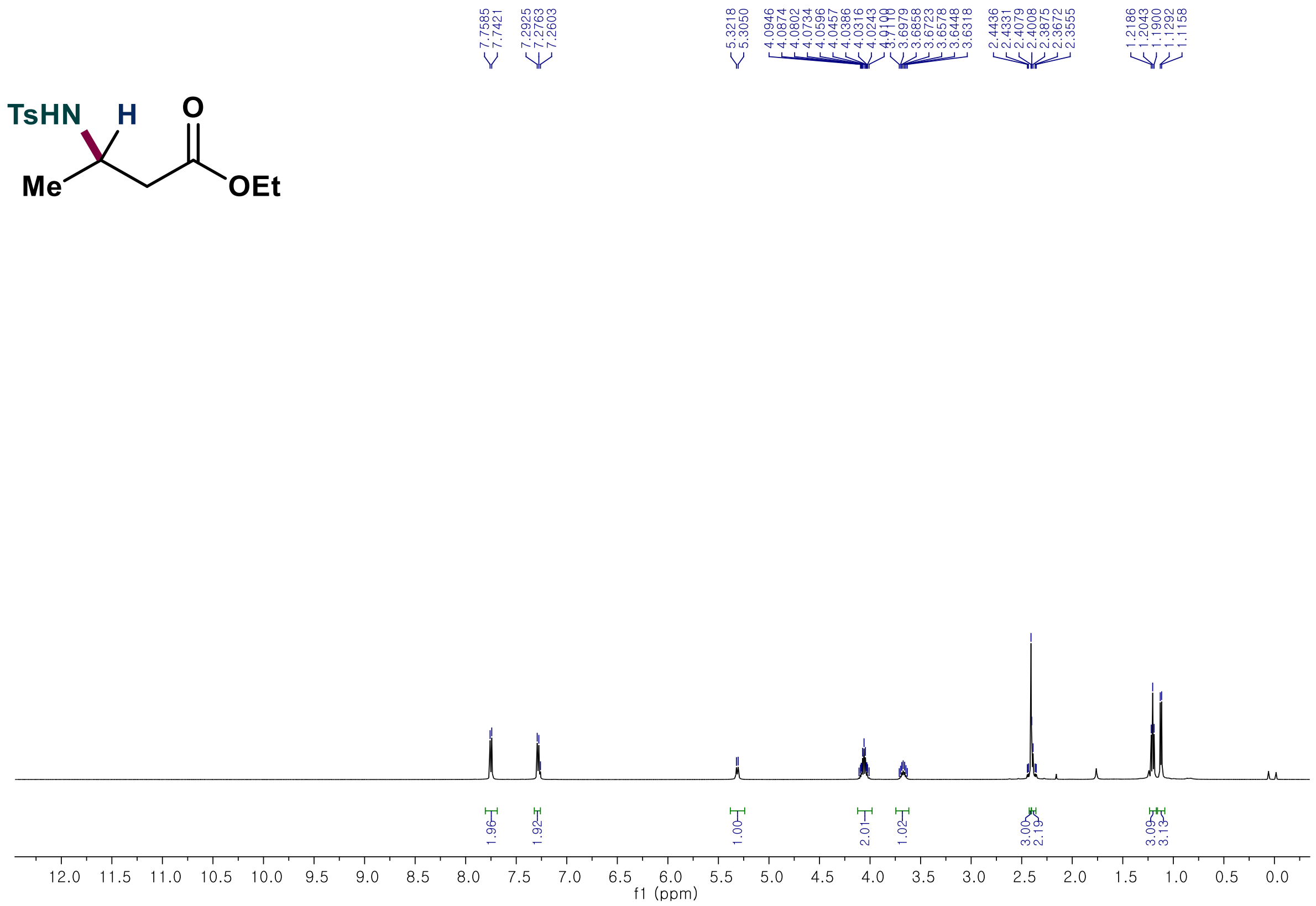

^{13}C NMR spectra (**3**)

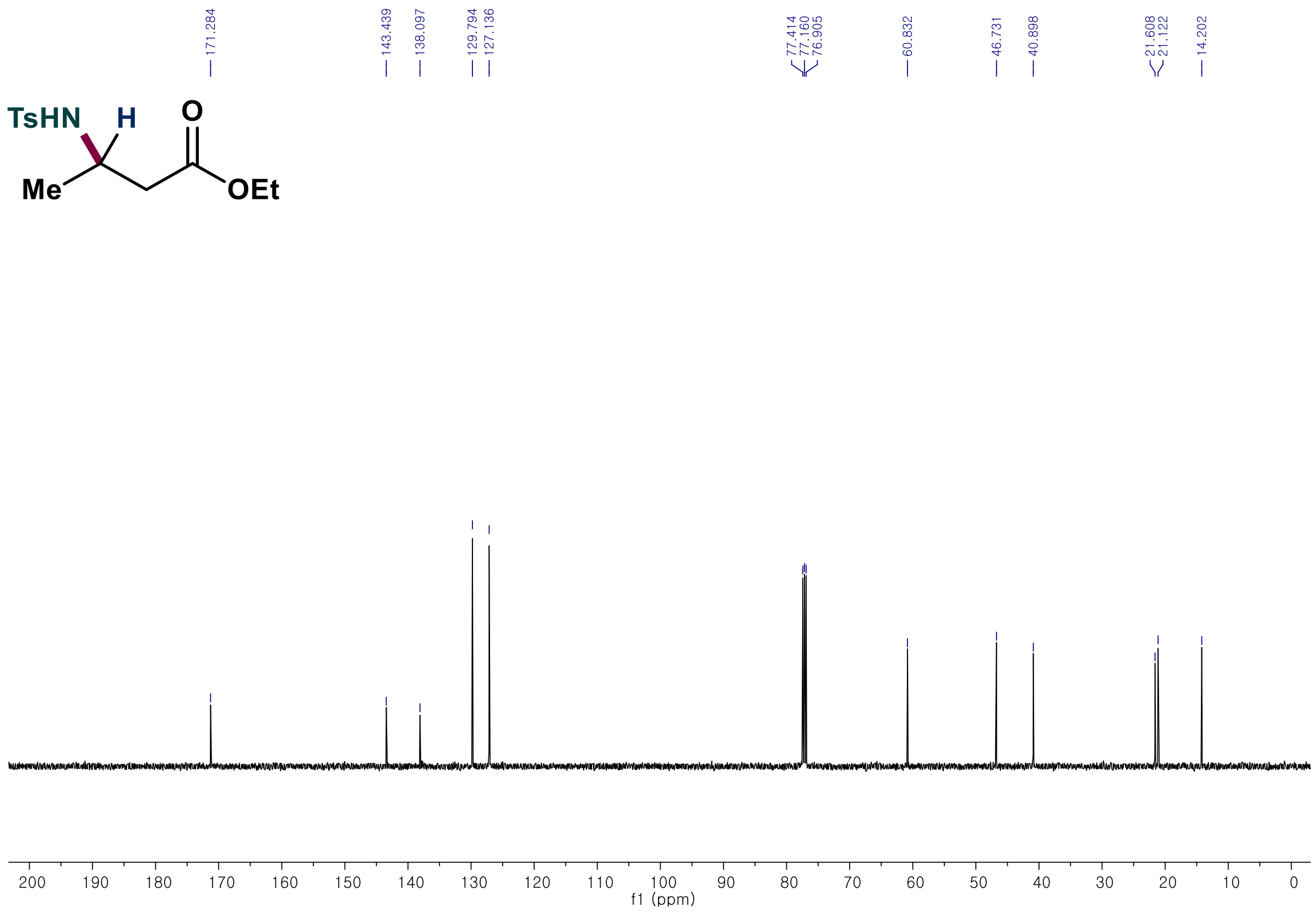

^{1}H NMR spectra (**4**)

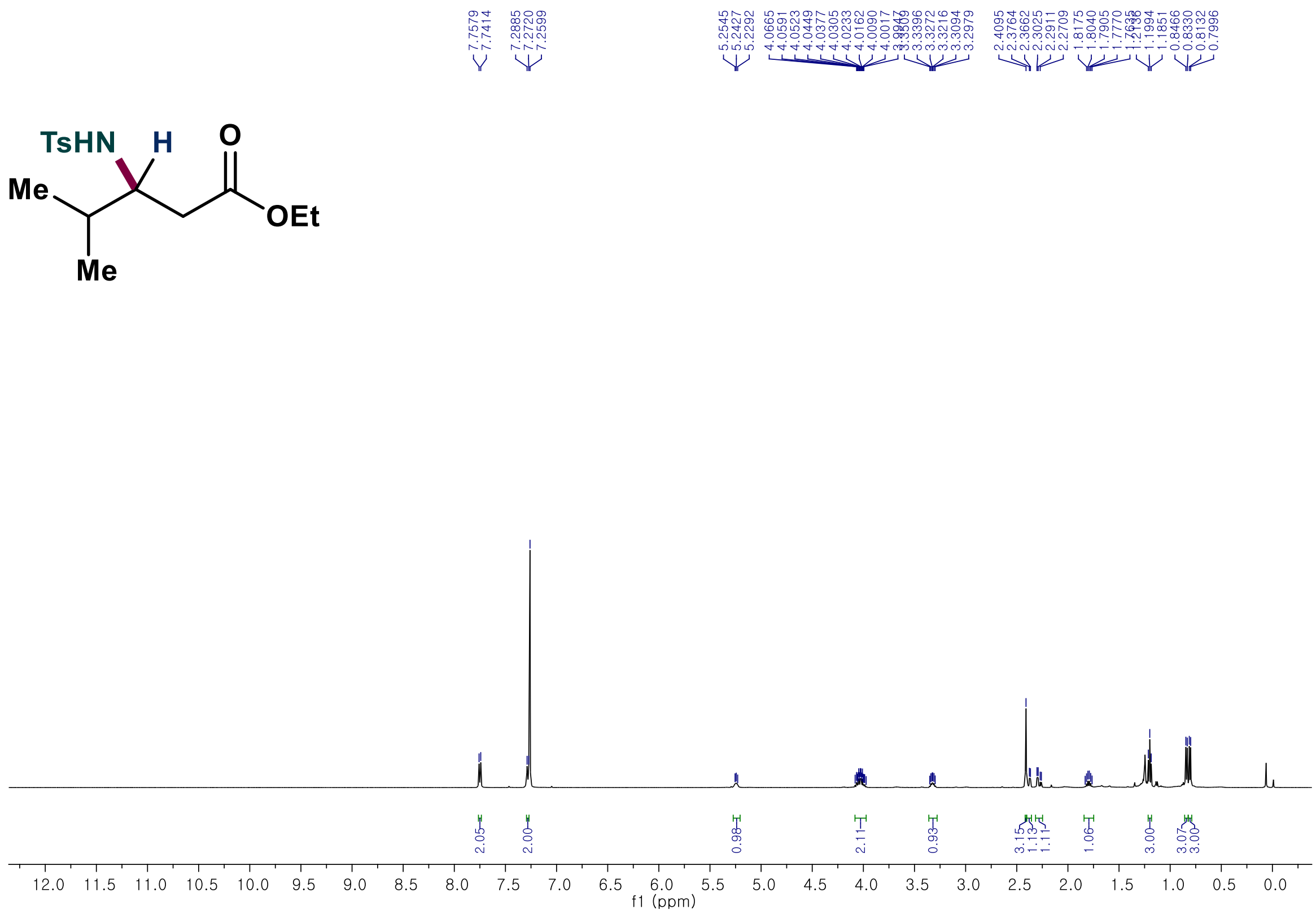

^{13}C NMR spectra (**4**)

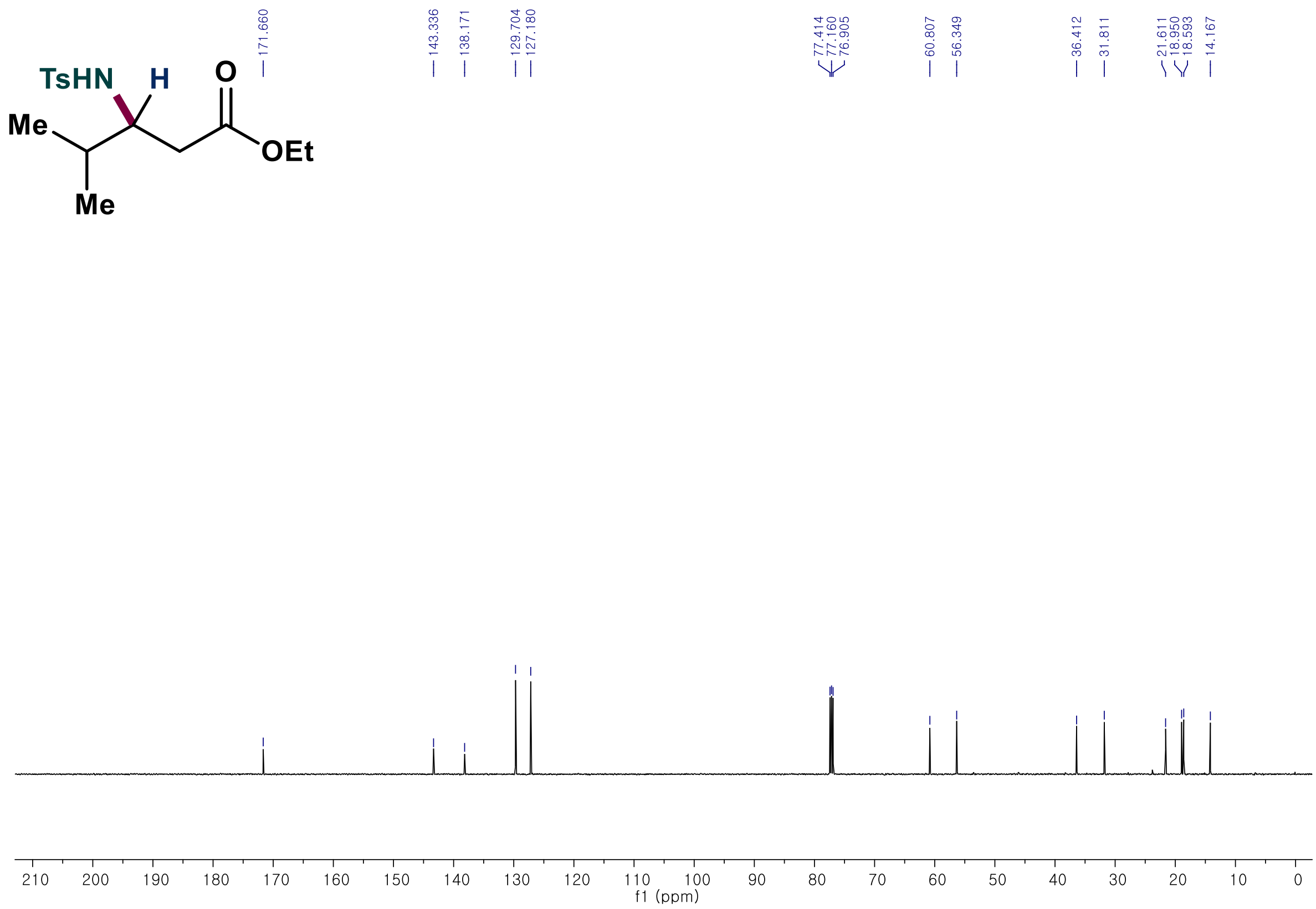

^{1}H NMR spectra (**5**)

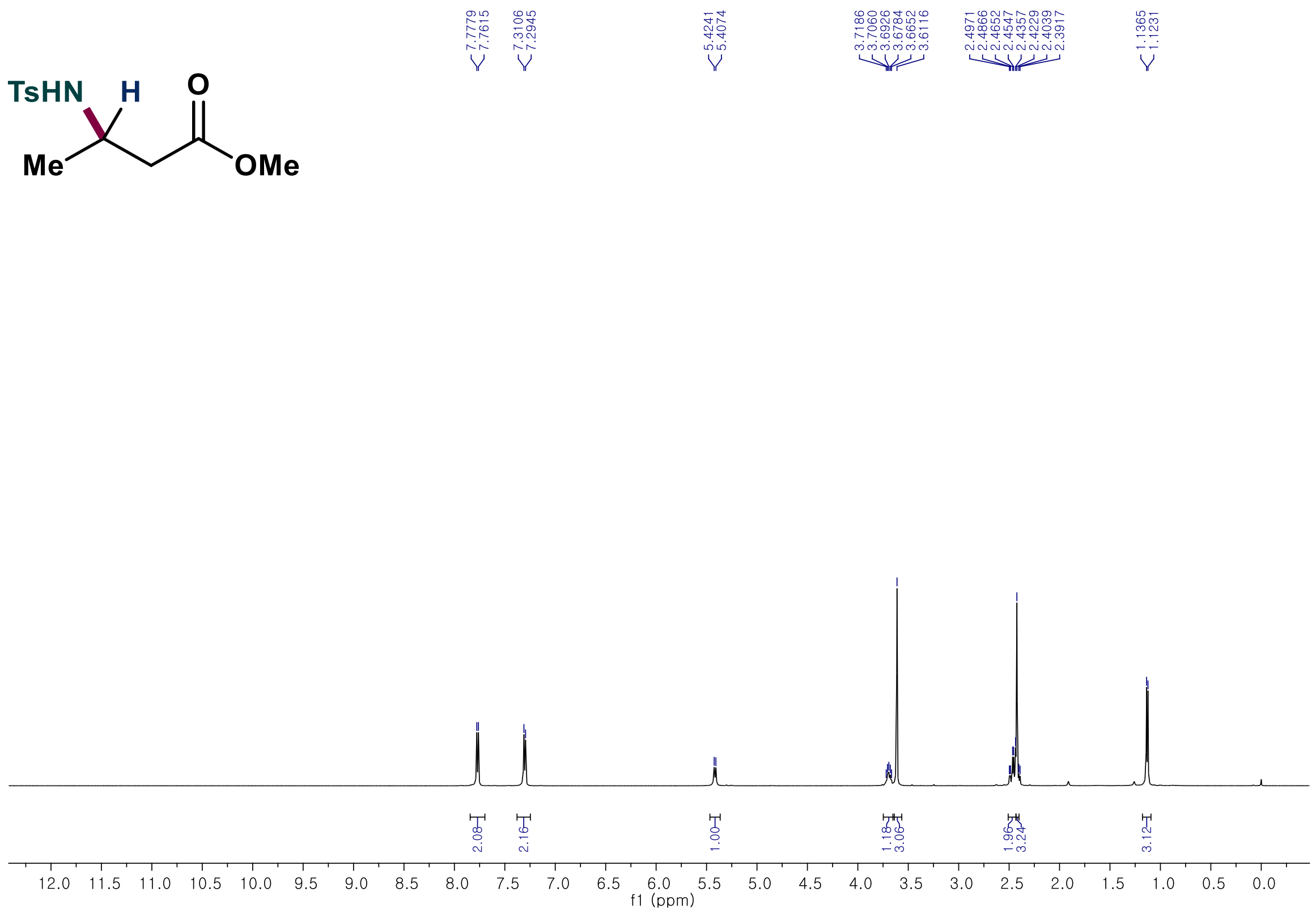

^{13}C NMR spectra (**5**)

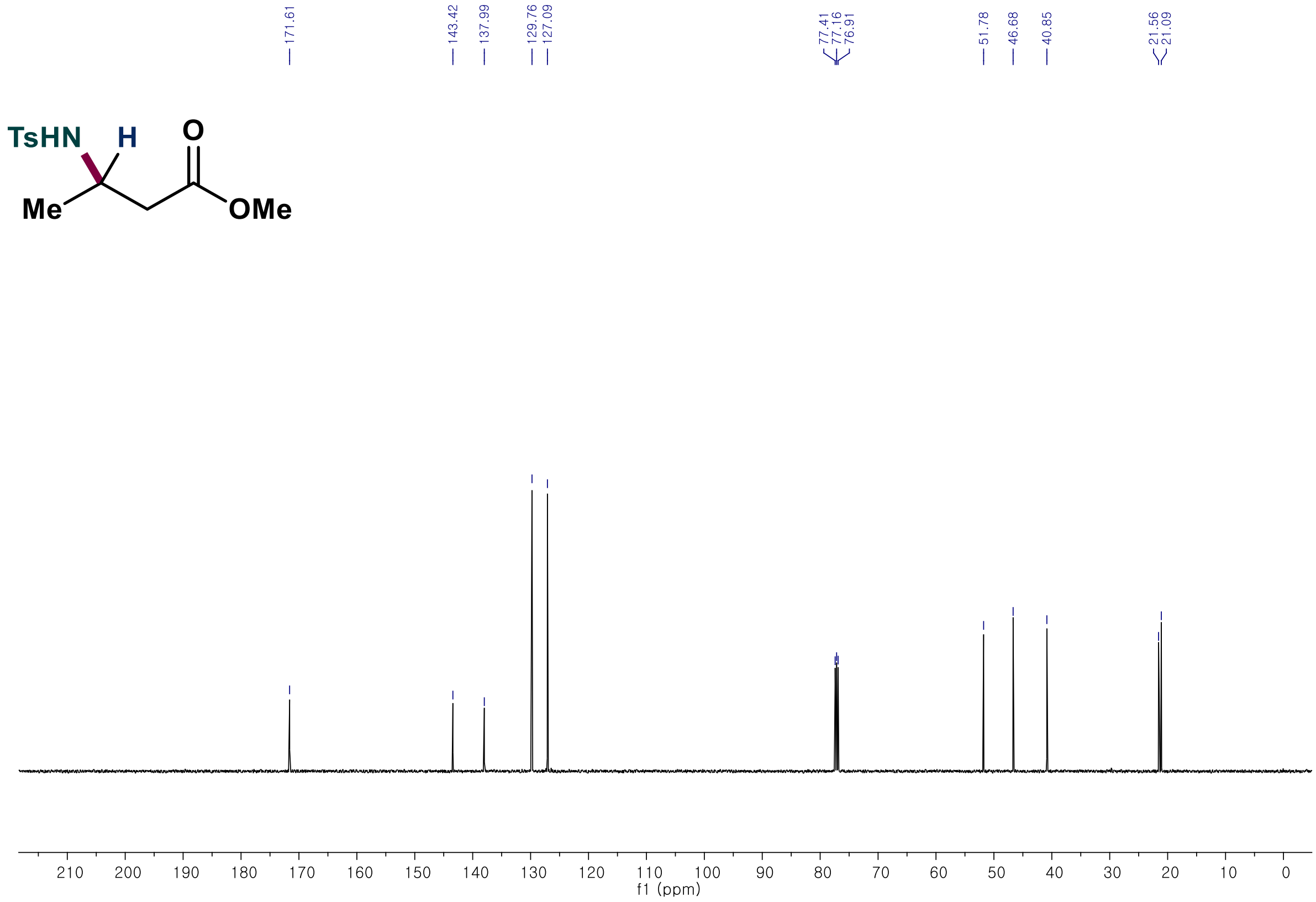

^{1}H NMR spectra (**6**)

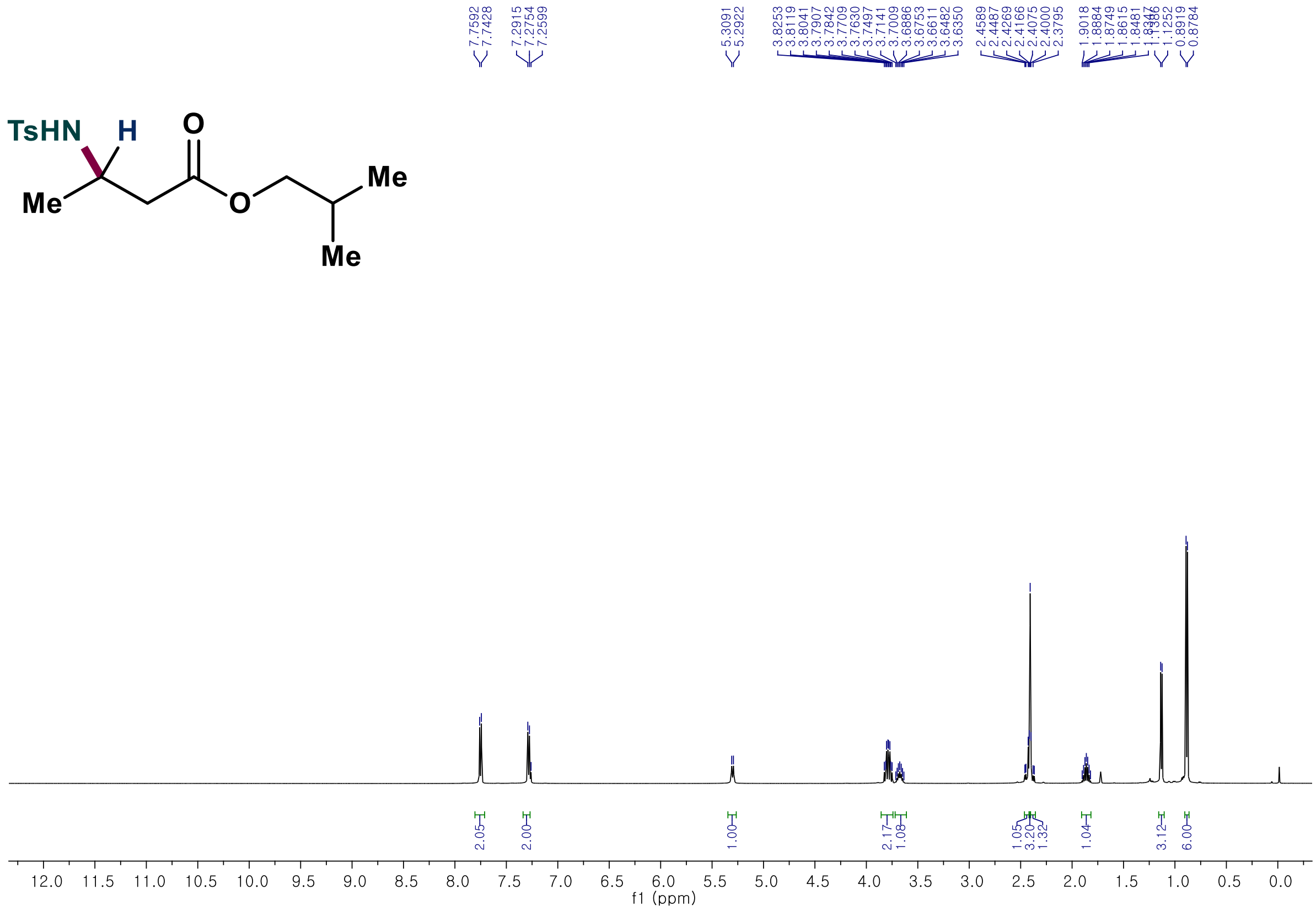

^{13}C NMR spectra (**6**)

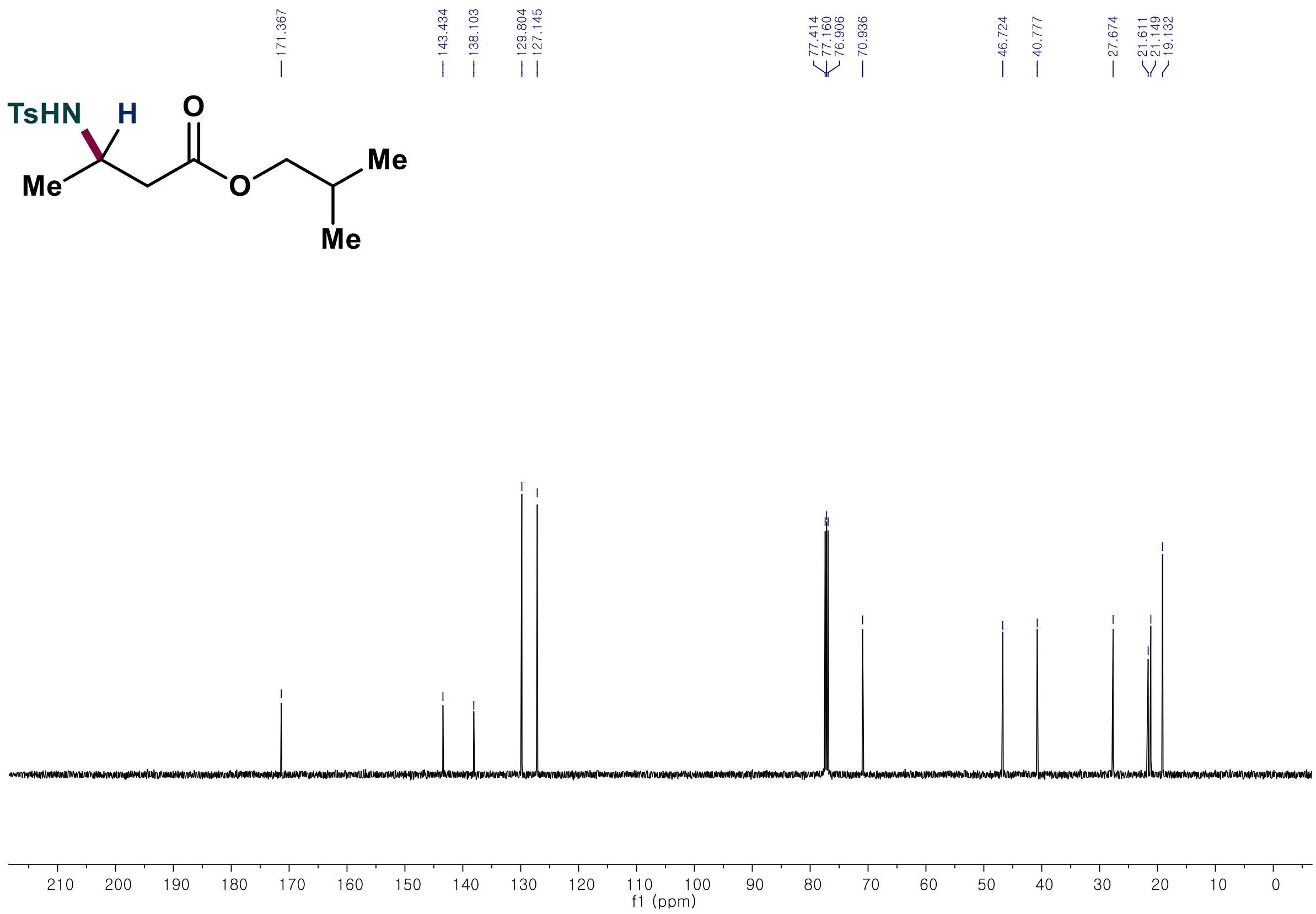

^{1}H NMR spectra (**7**)

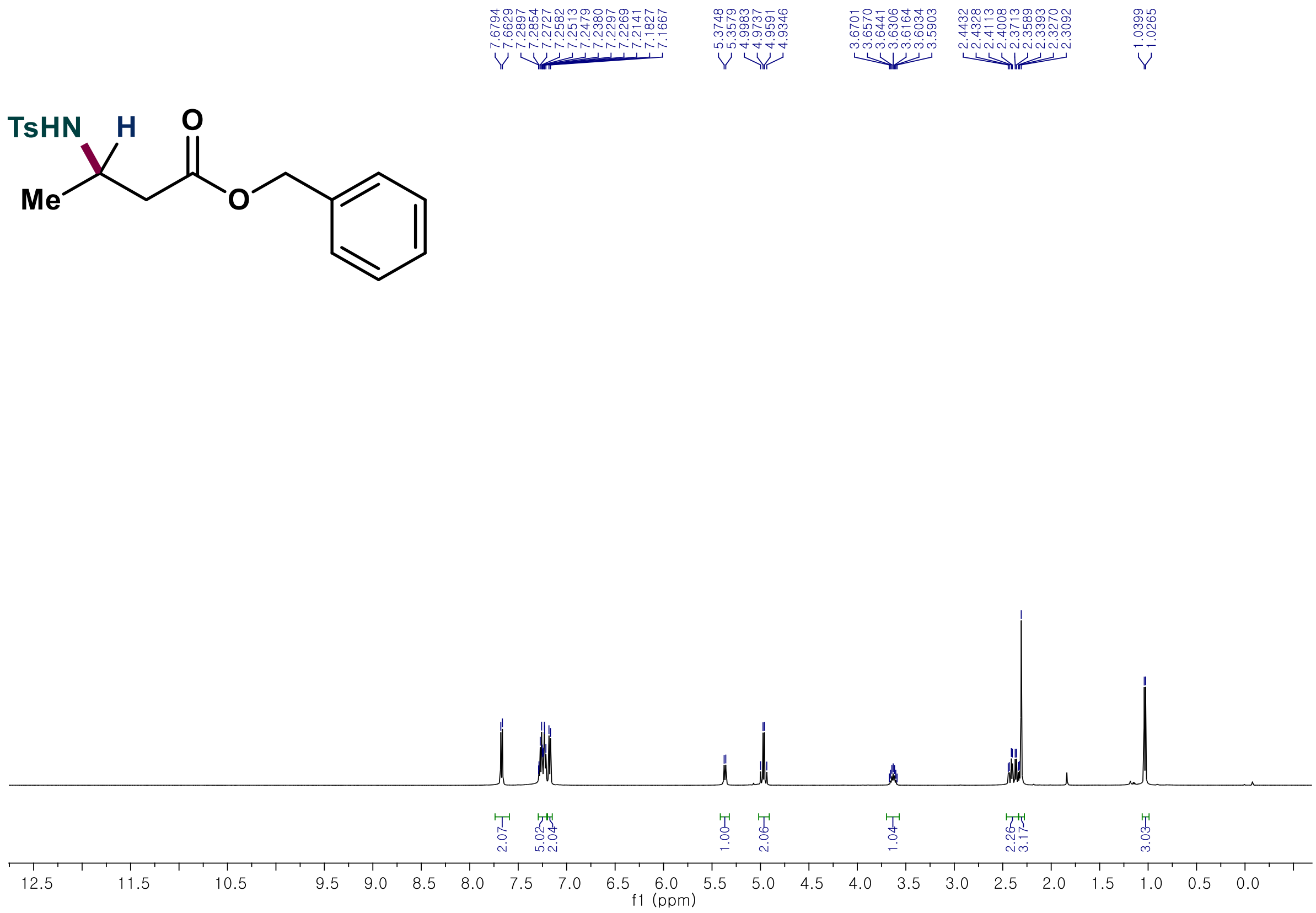

^{13}C NMR spectra (**7**)

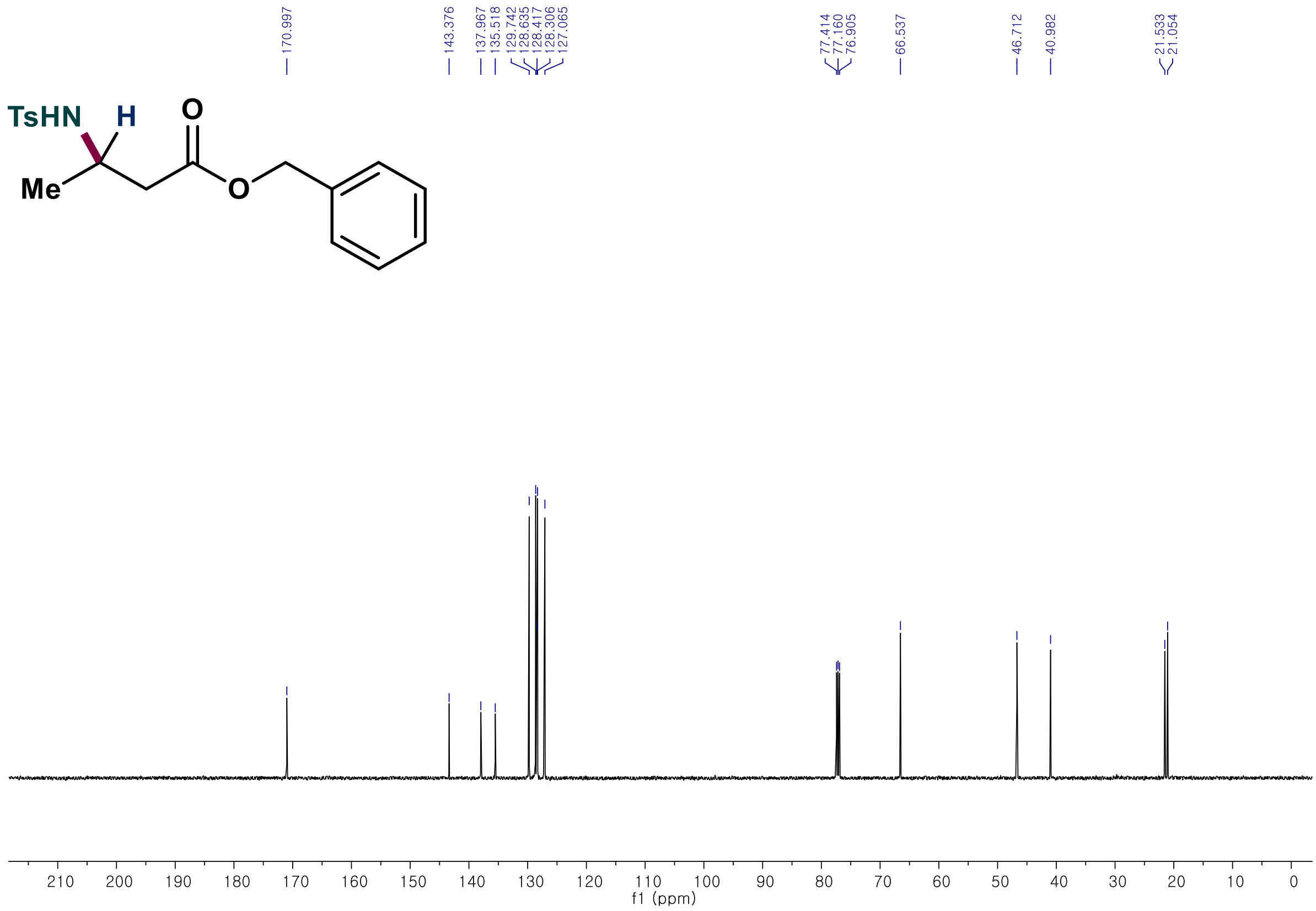

^{1}H NMR spectra (**8**)

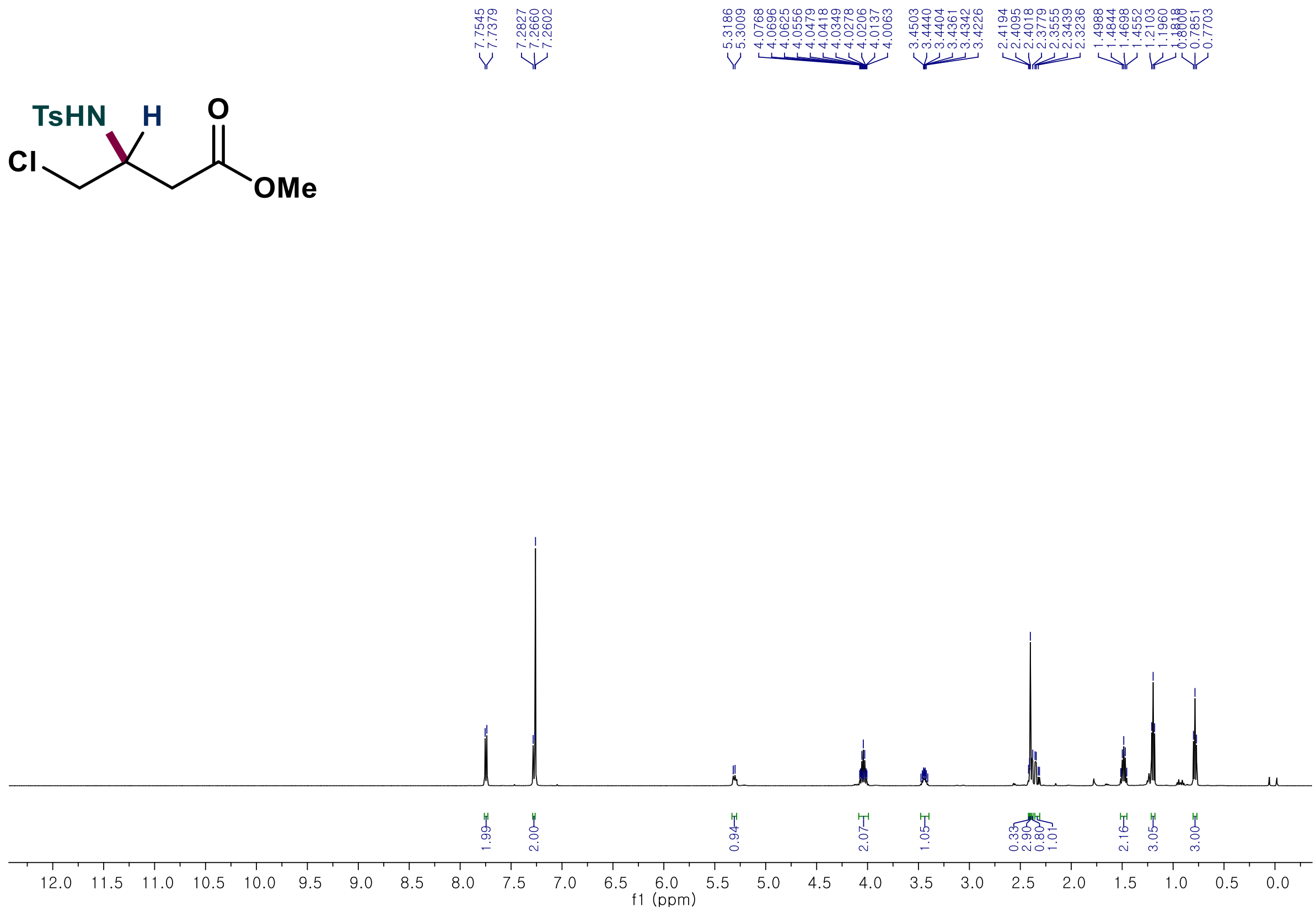

^{13}C NMR spectra (**8**)

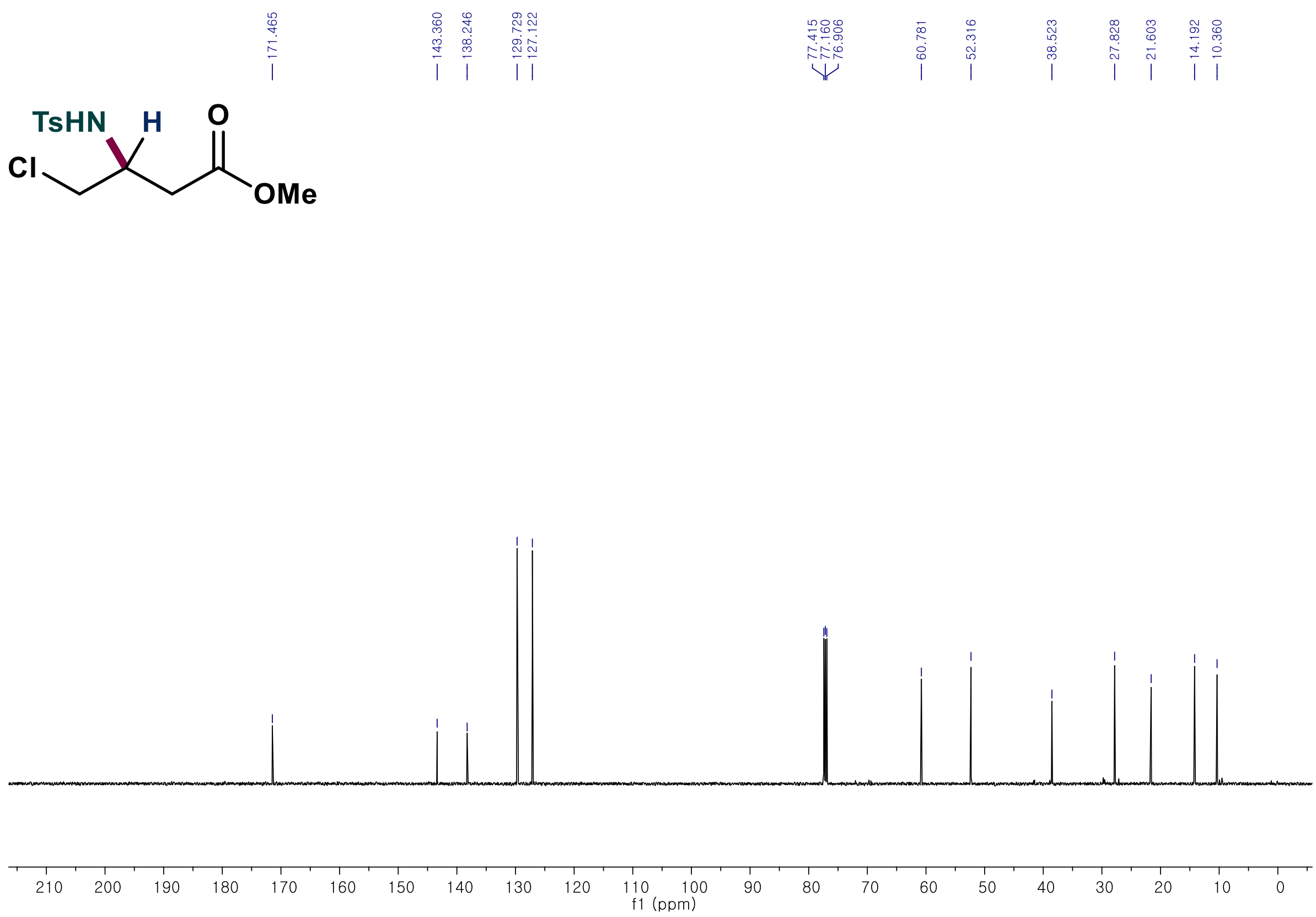

^{1}H NMR spectra (**9**)

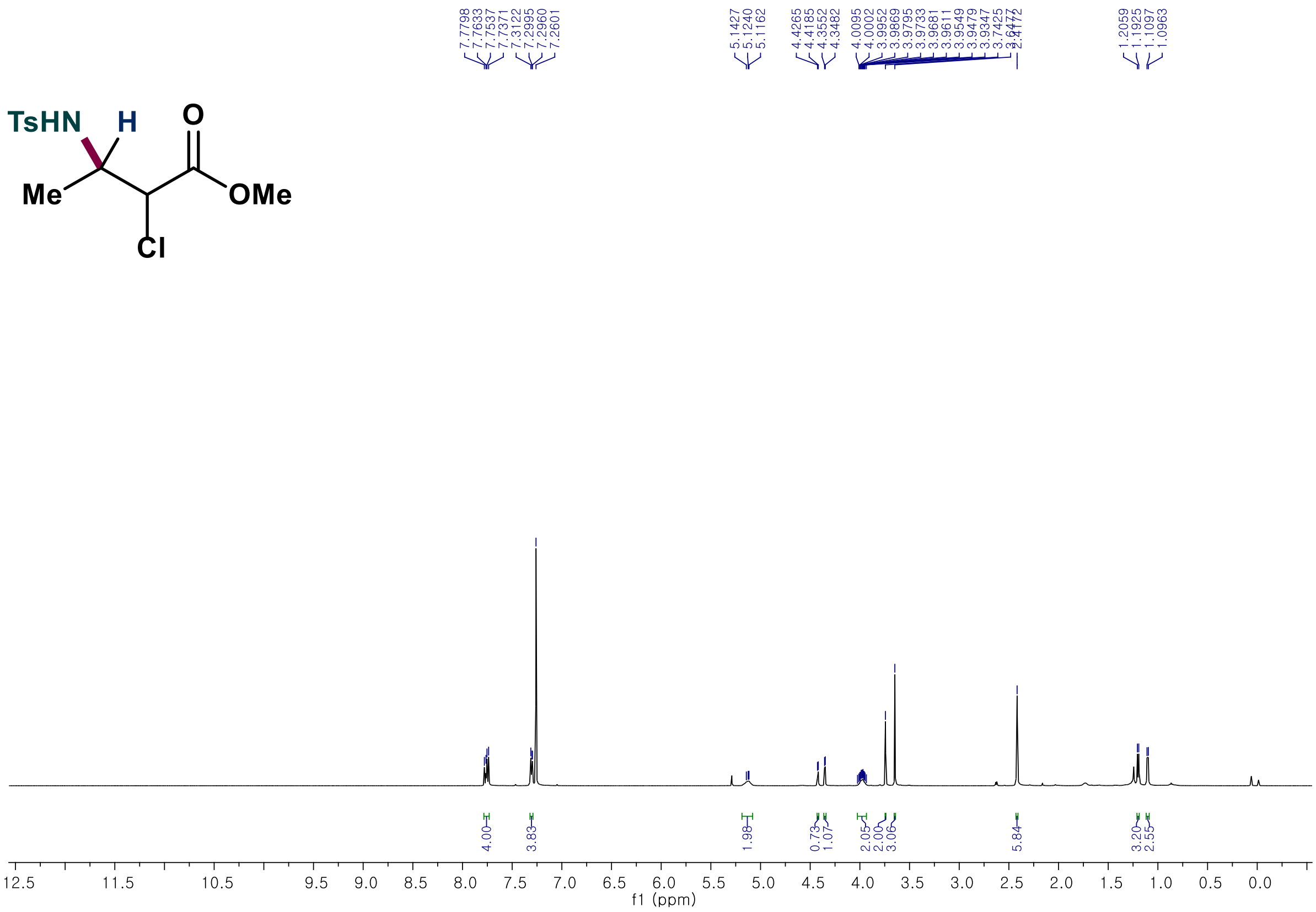

^{13}C NMR spectra (**9**)

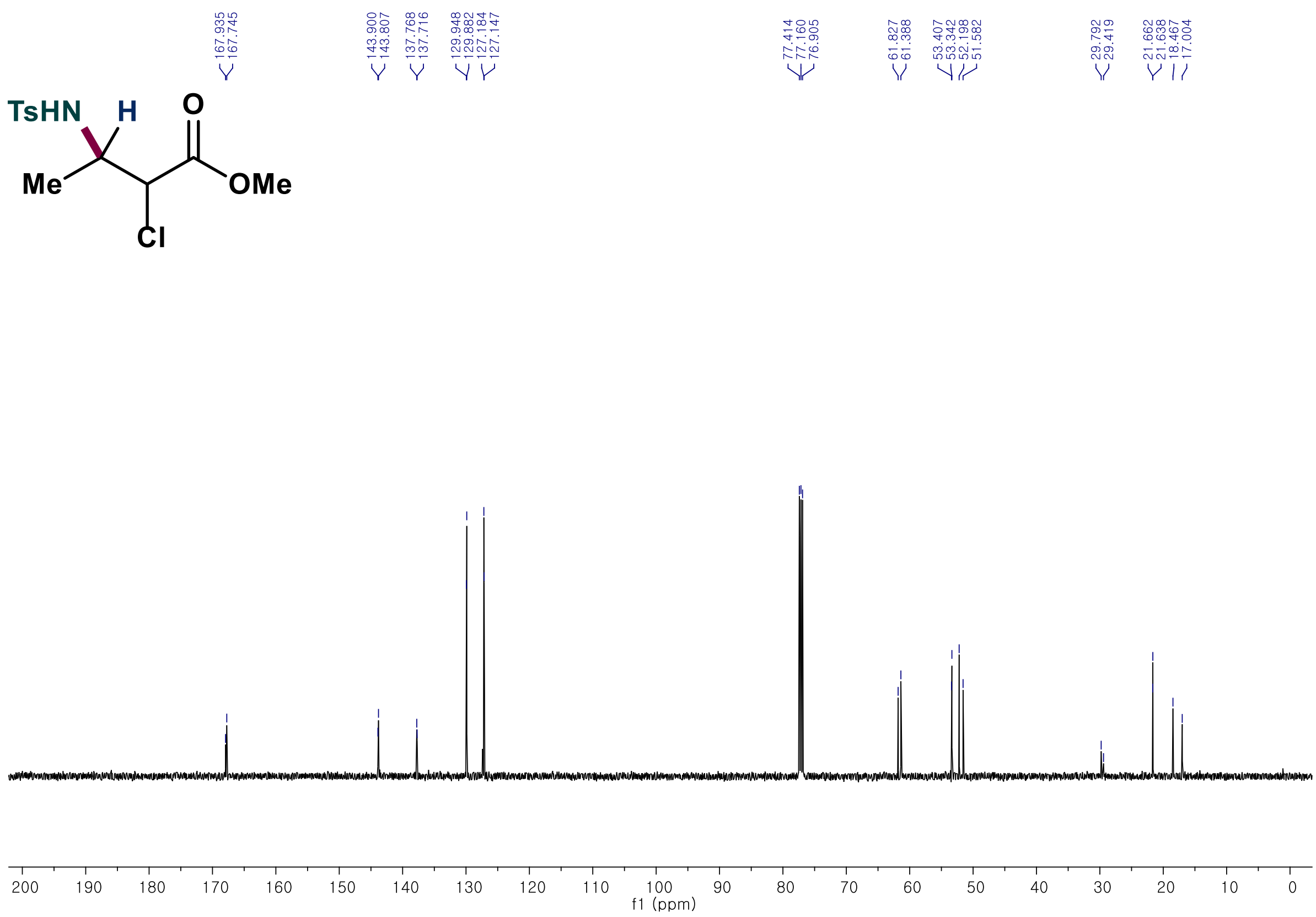

^{1}H NMR spectra (**10**)

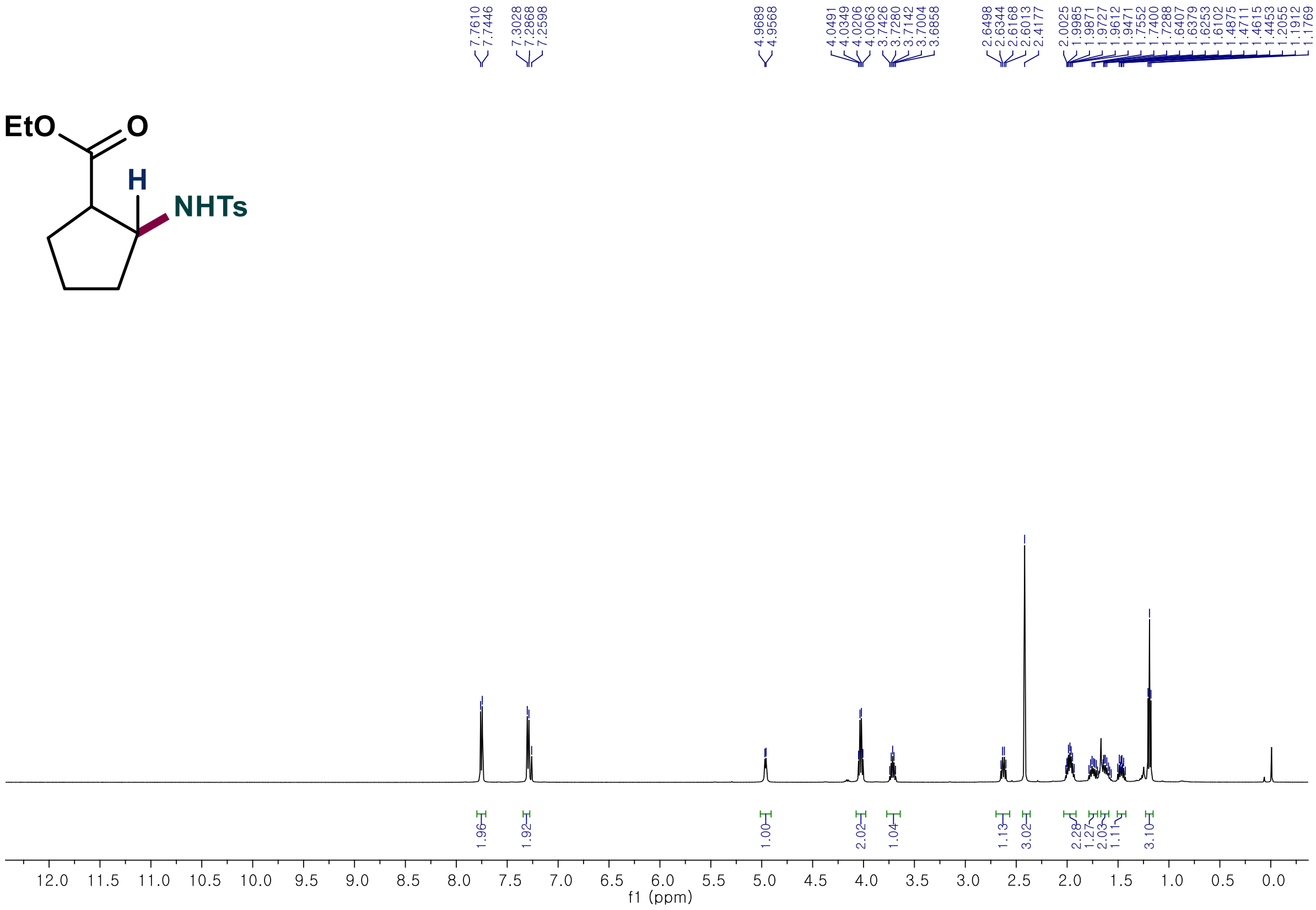

^{13}C NMR spectra (**10**)

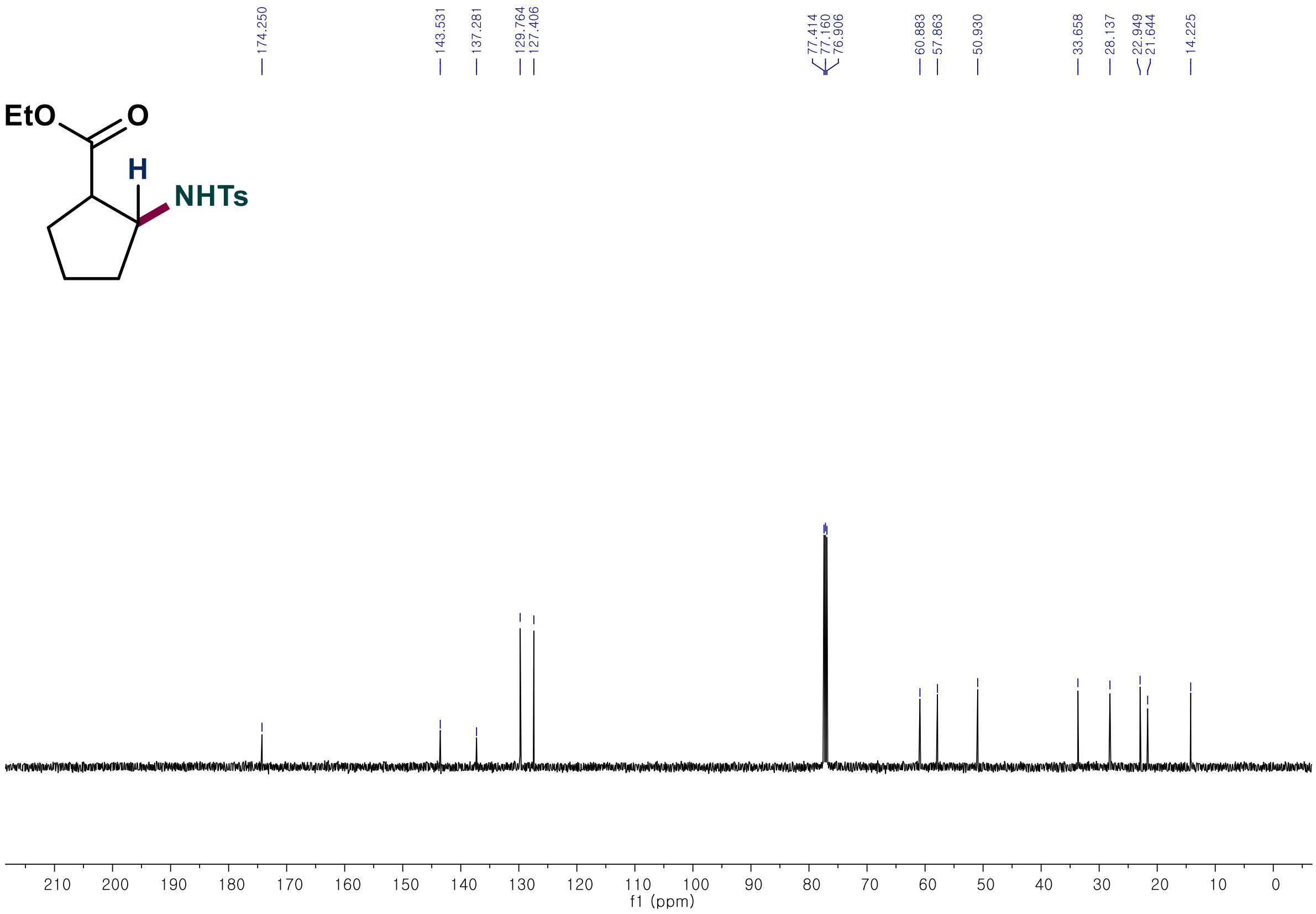

^{1}H NMR spectra (**11**)

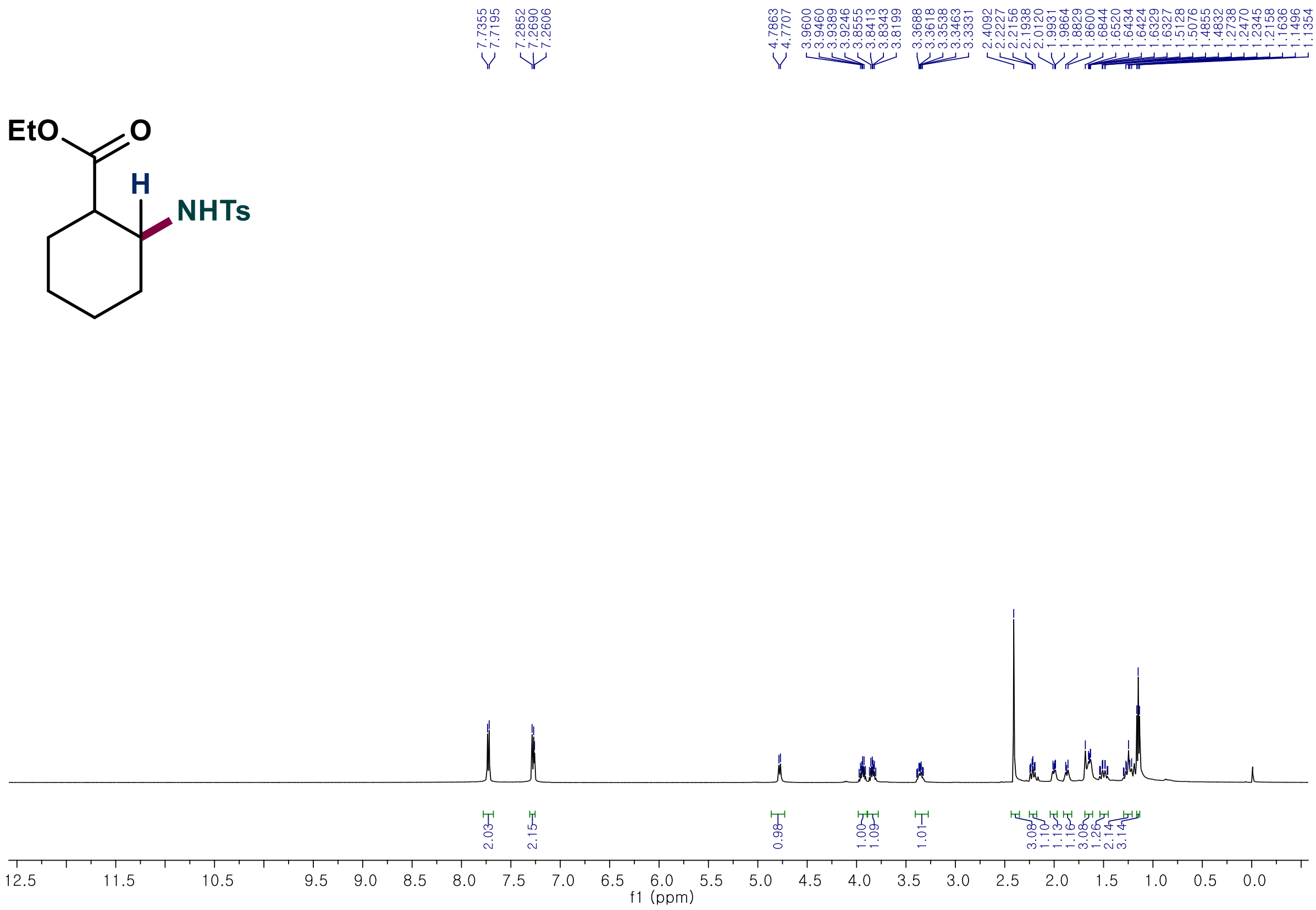

^{13}C NMR spectra (**11**)

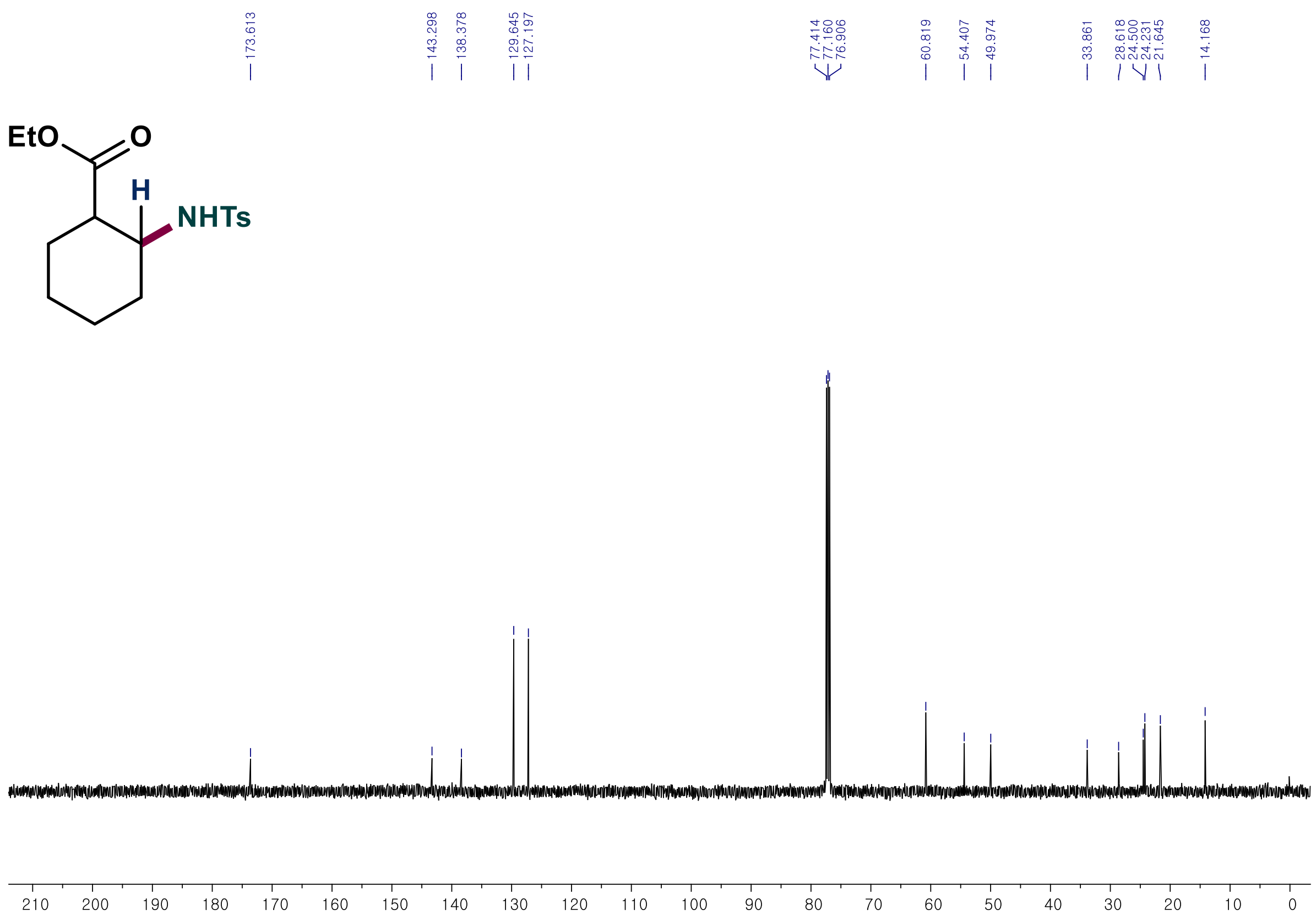

^{1}H NMR spectra (**12**)

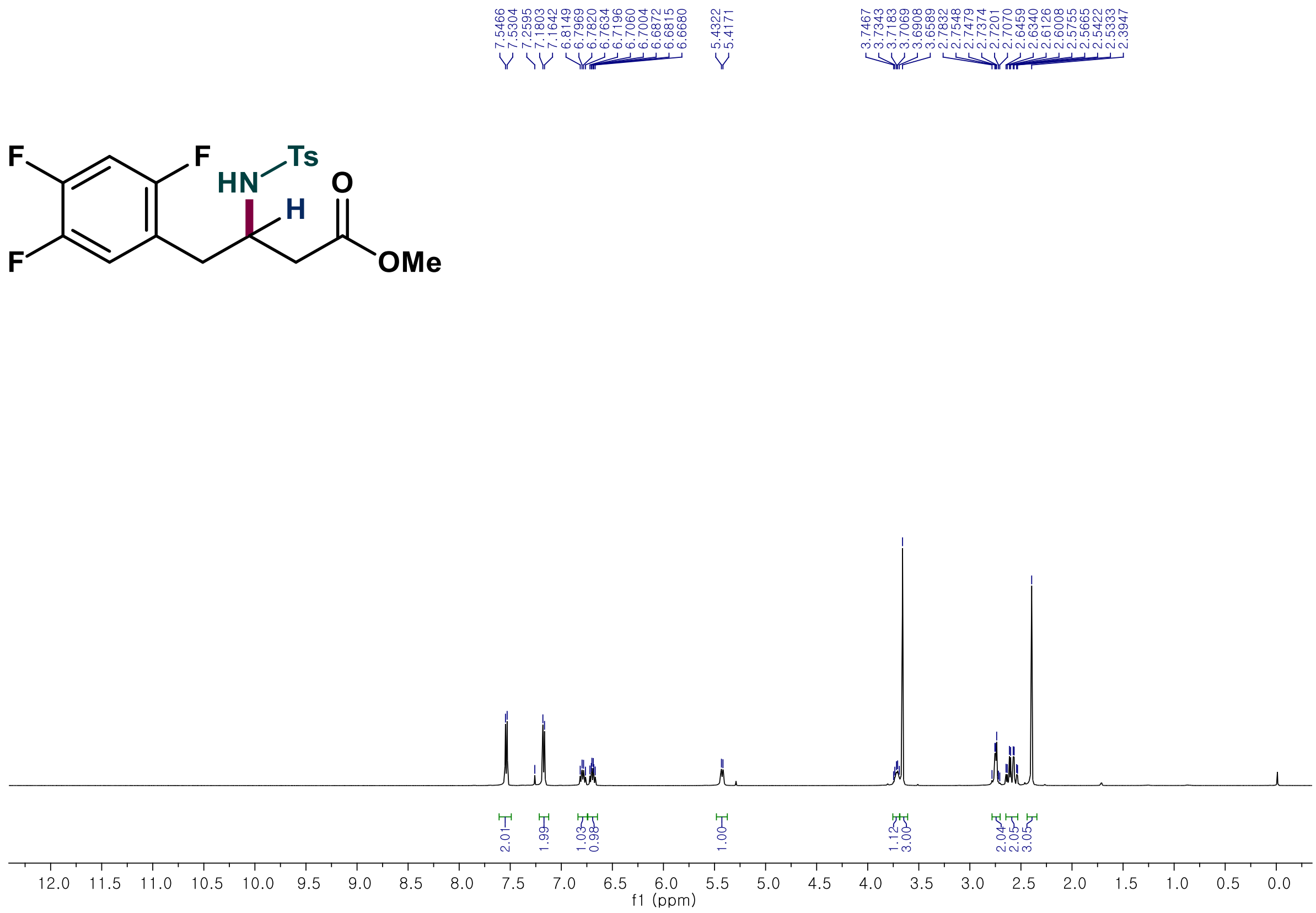

^{13}C NMR spectra (**12**)

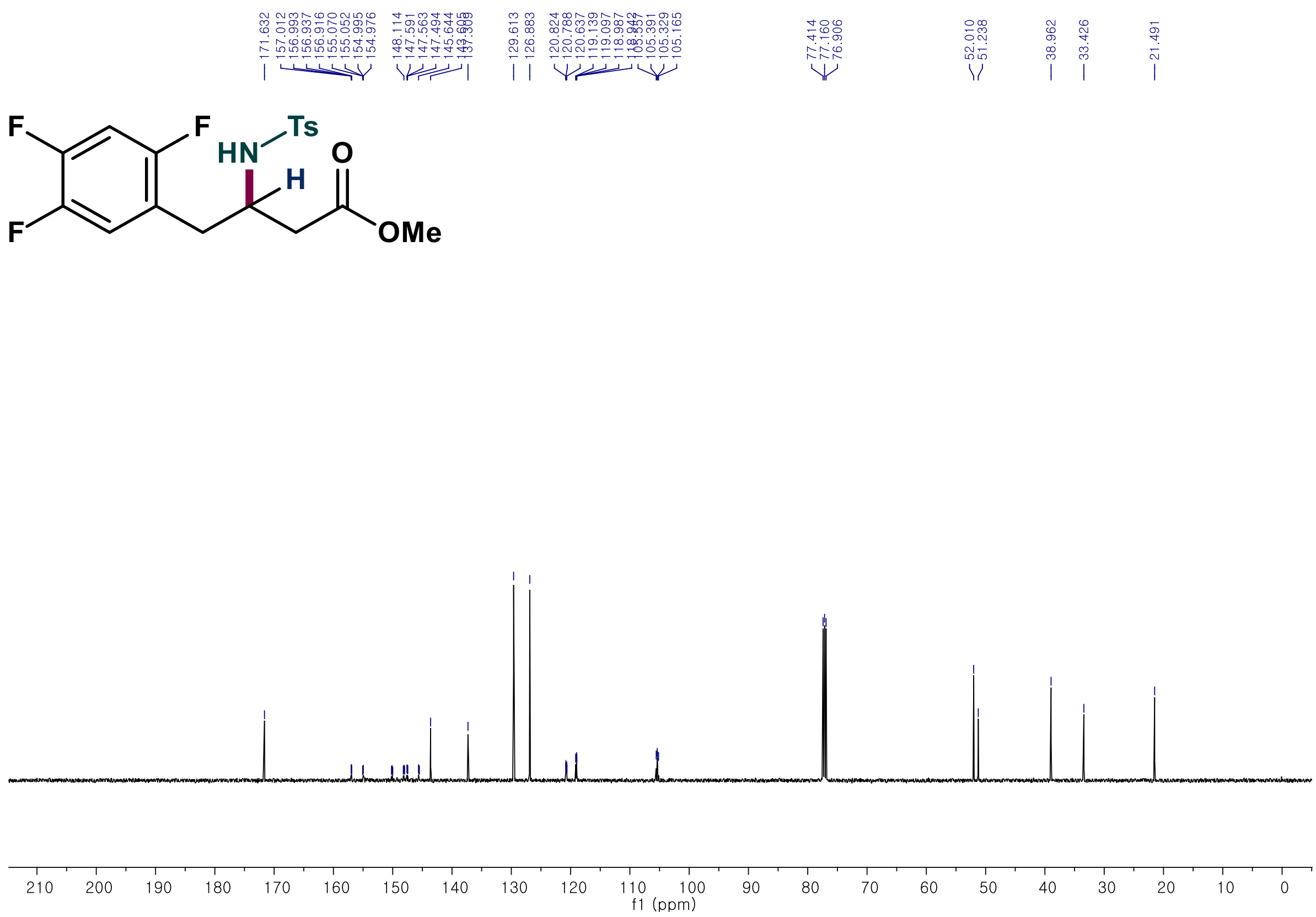

^{19}F NMR spectra (**12**)

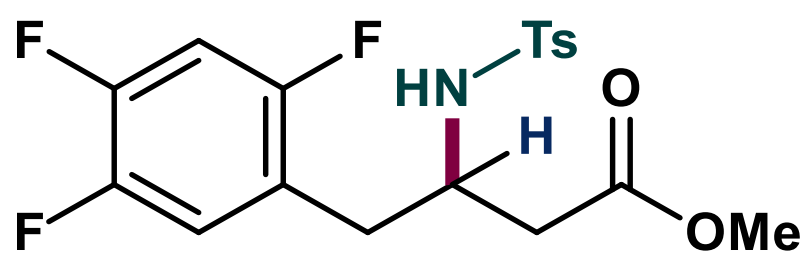

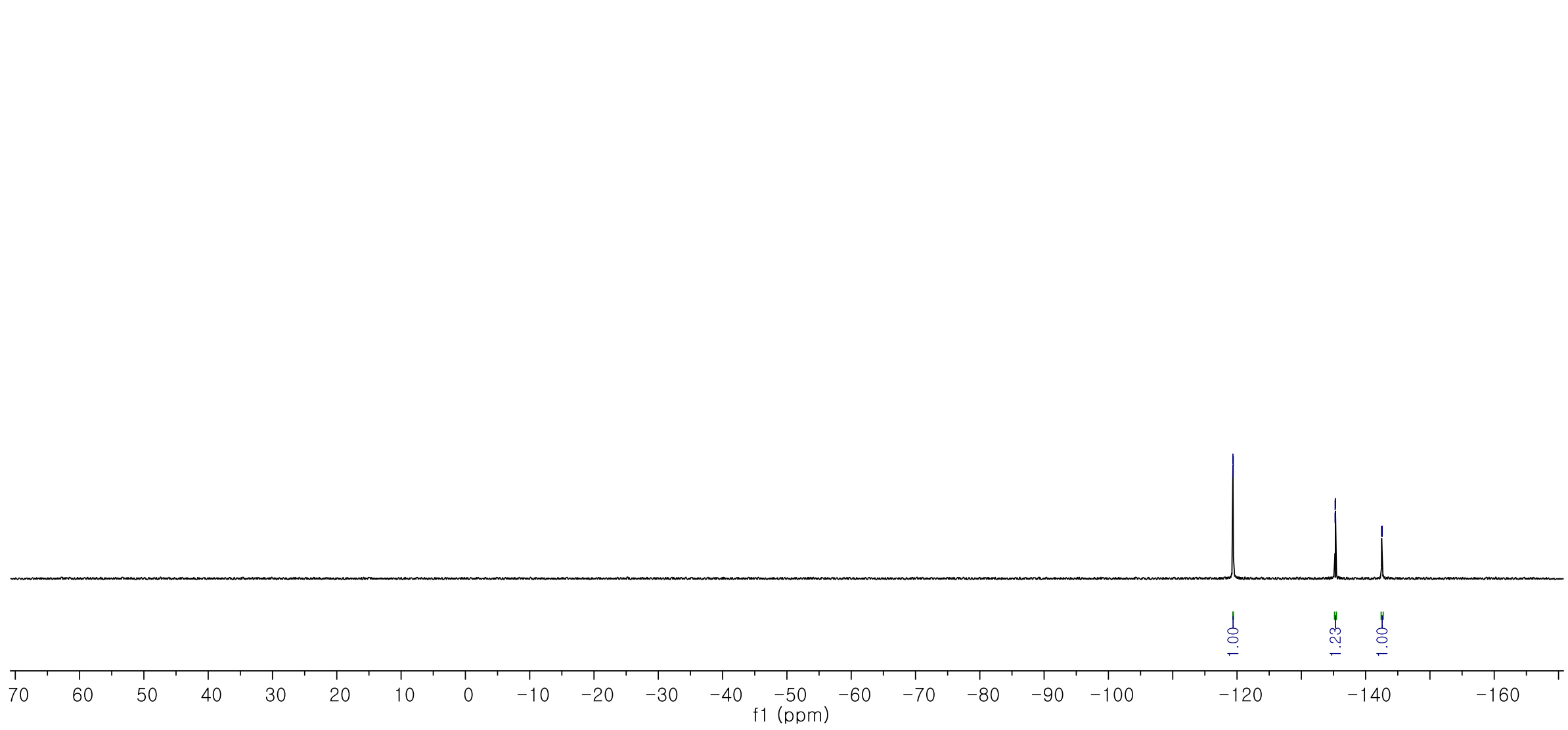

^{1}H NMR spectra (**13**)

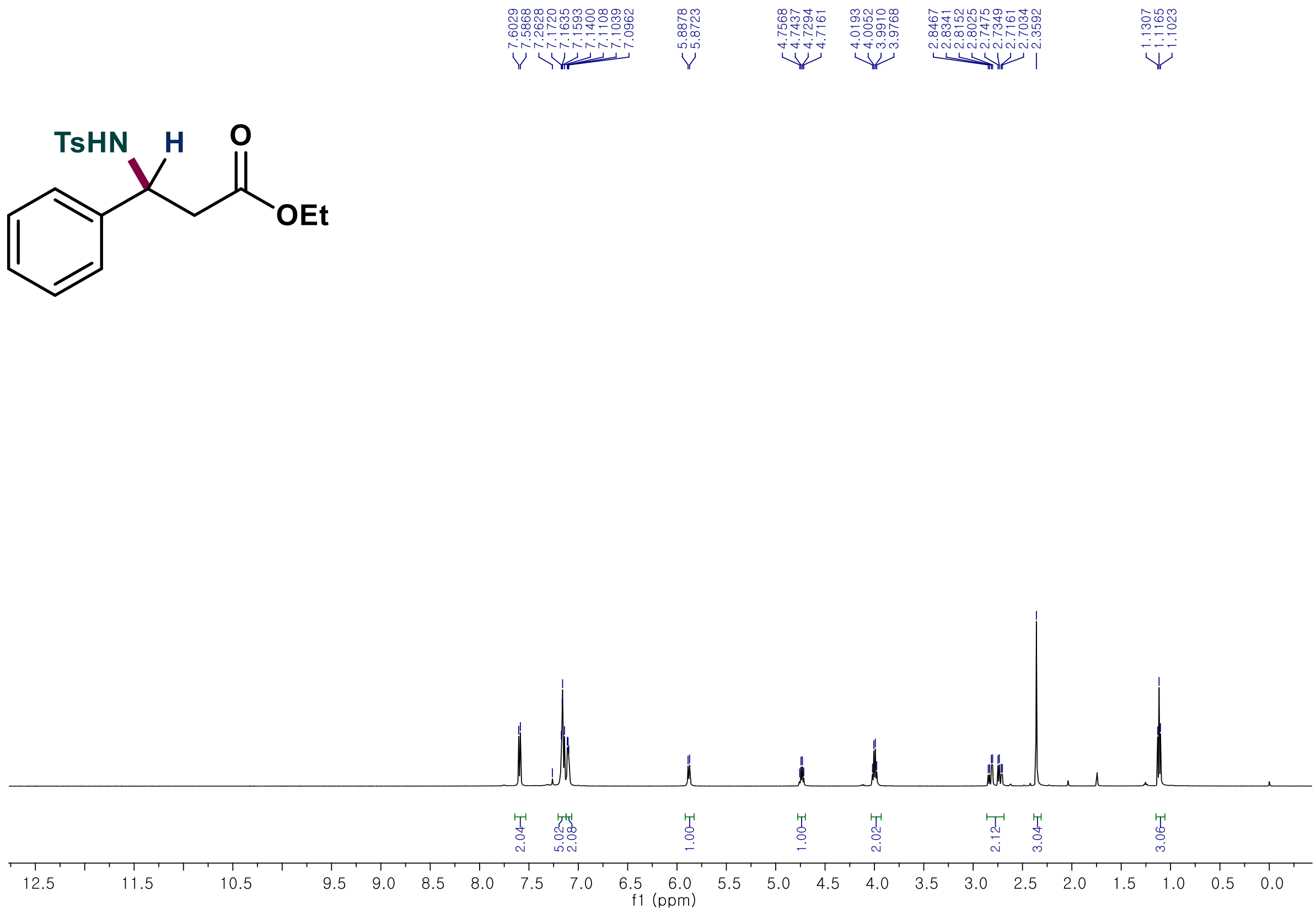

^{13}C NMR spectra (**13**)

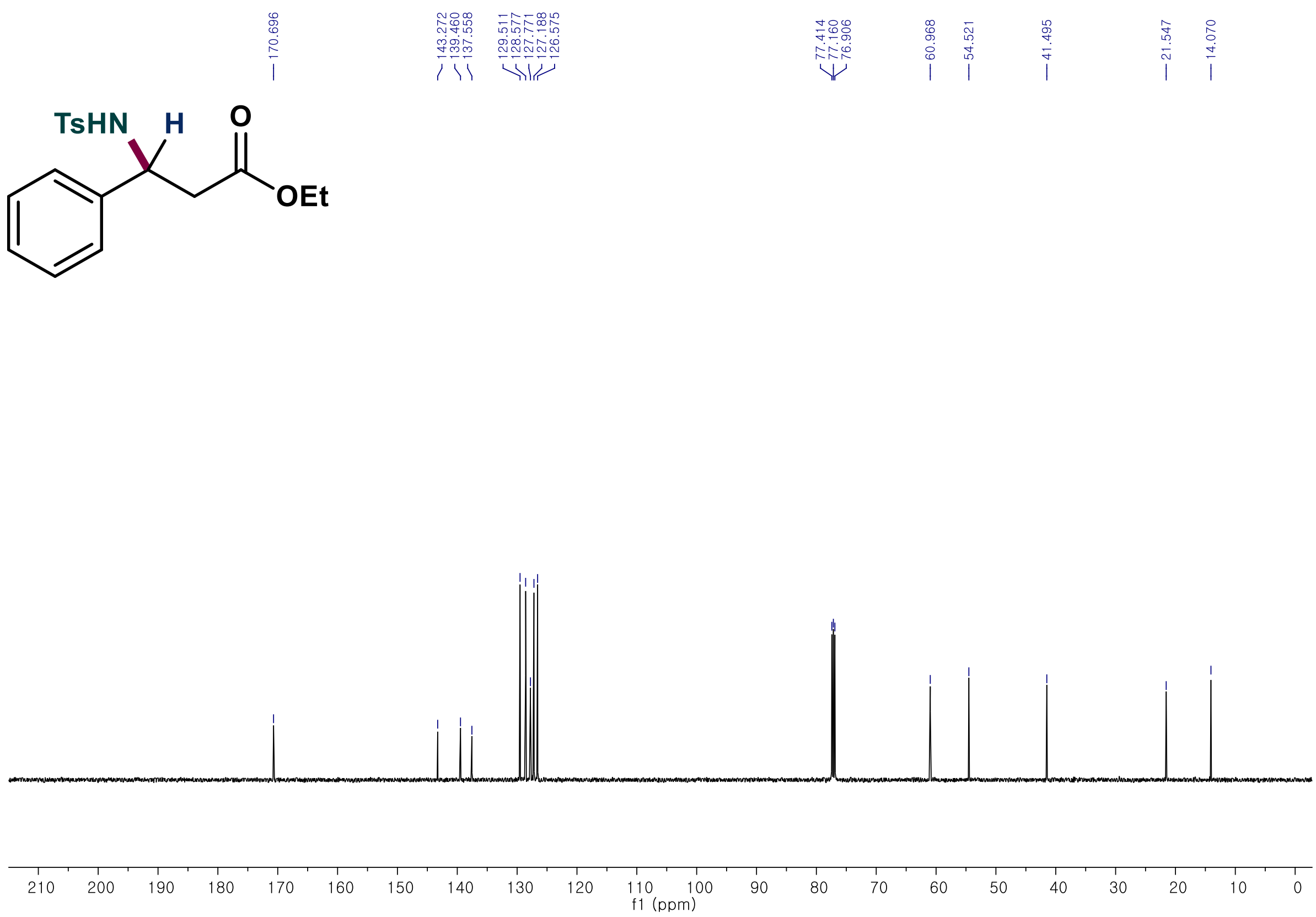

^{1}H NMR spectra (**14**)

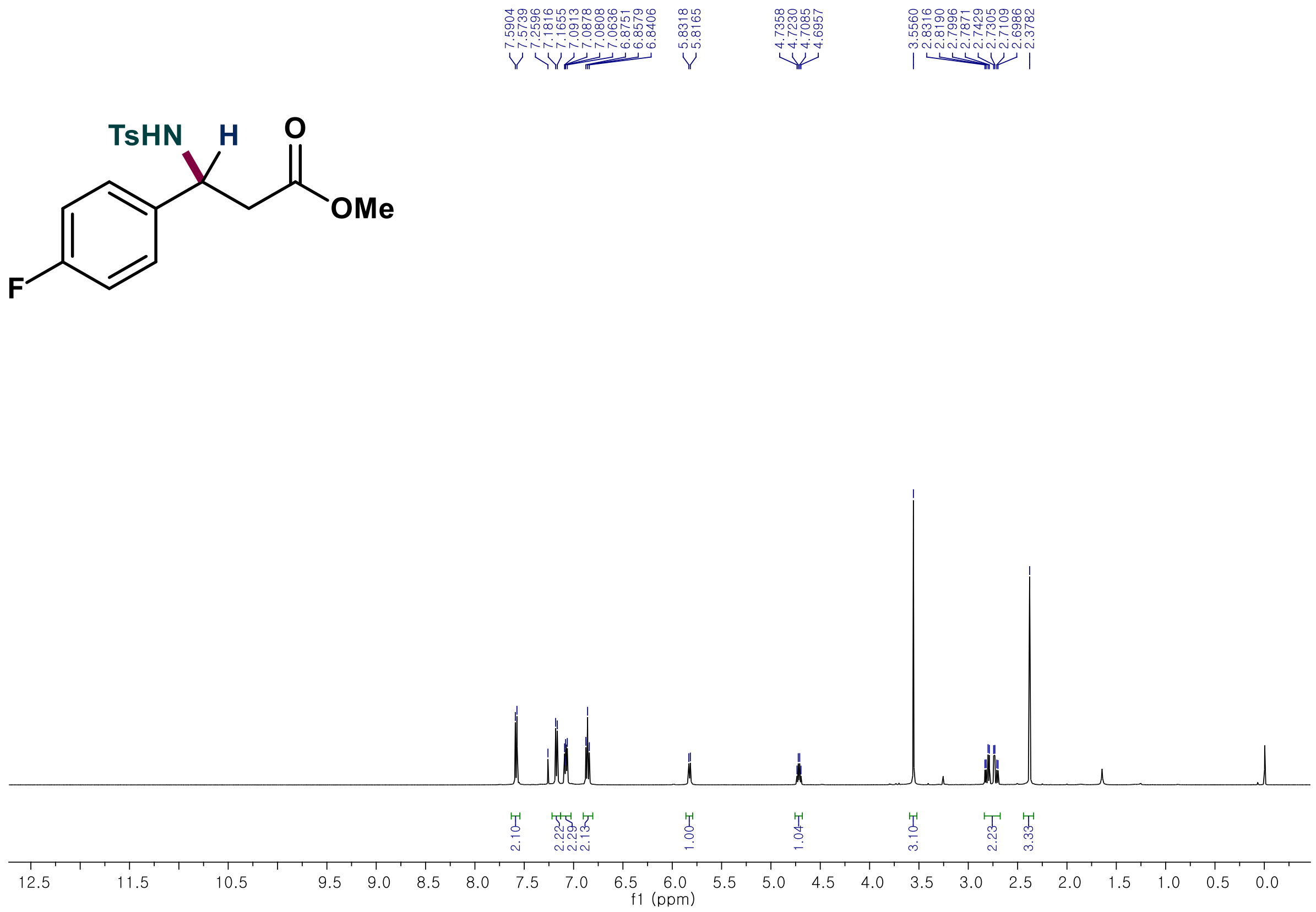

^{13}C NMR spectra (**14**)

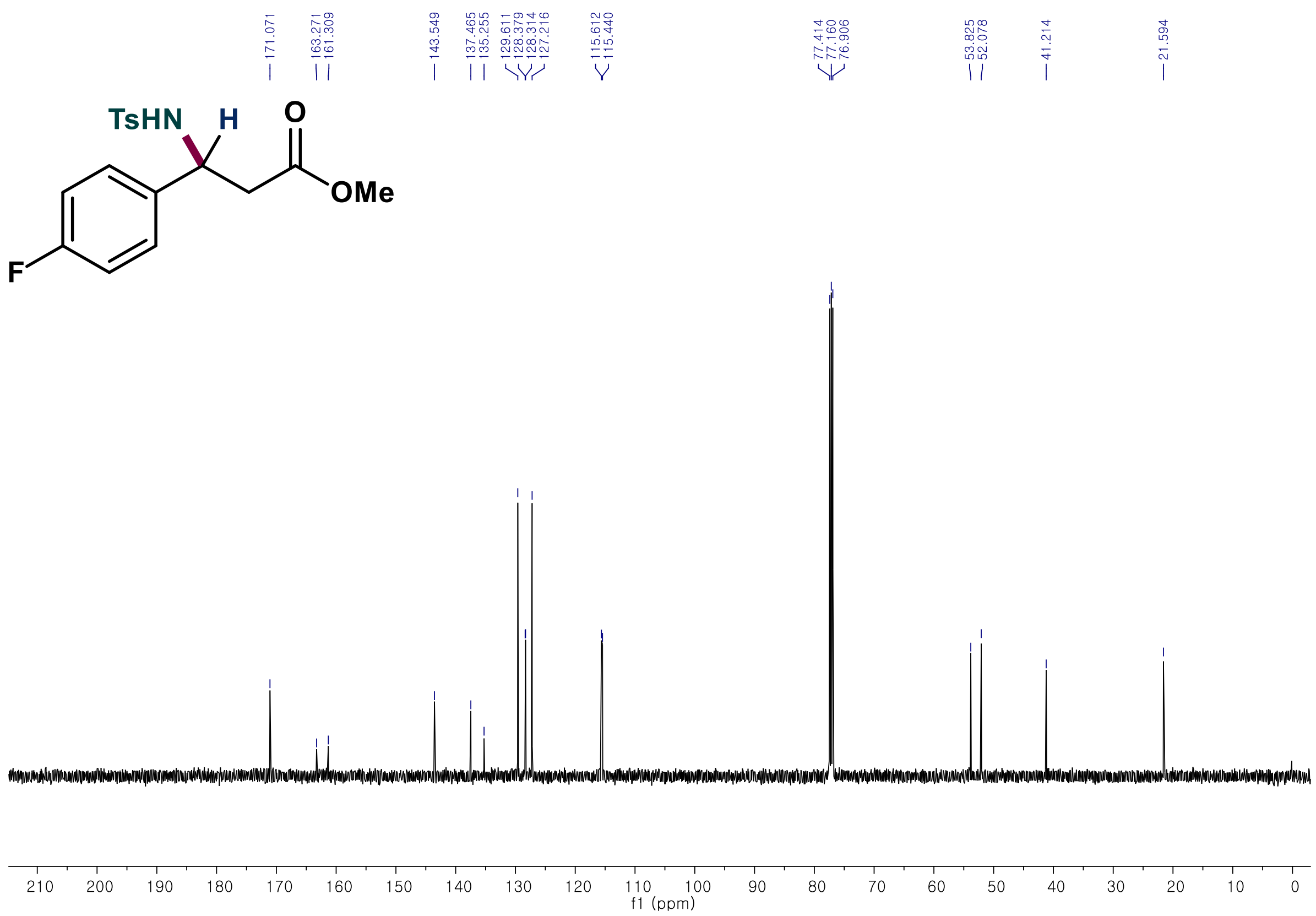

^{19}F NMR spectra (**14**)

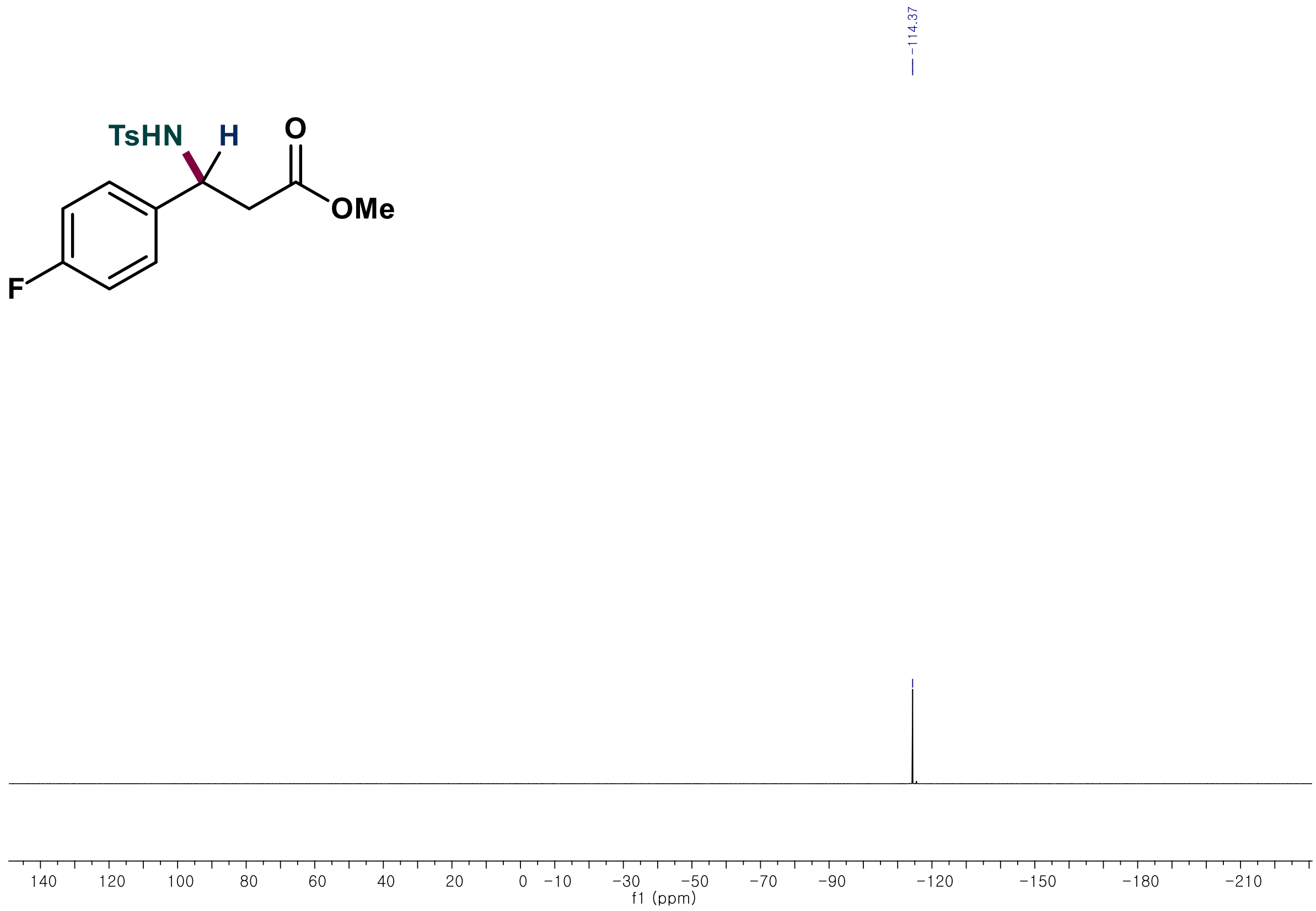

^{1}H NMR spectra (**15**)

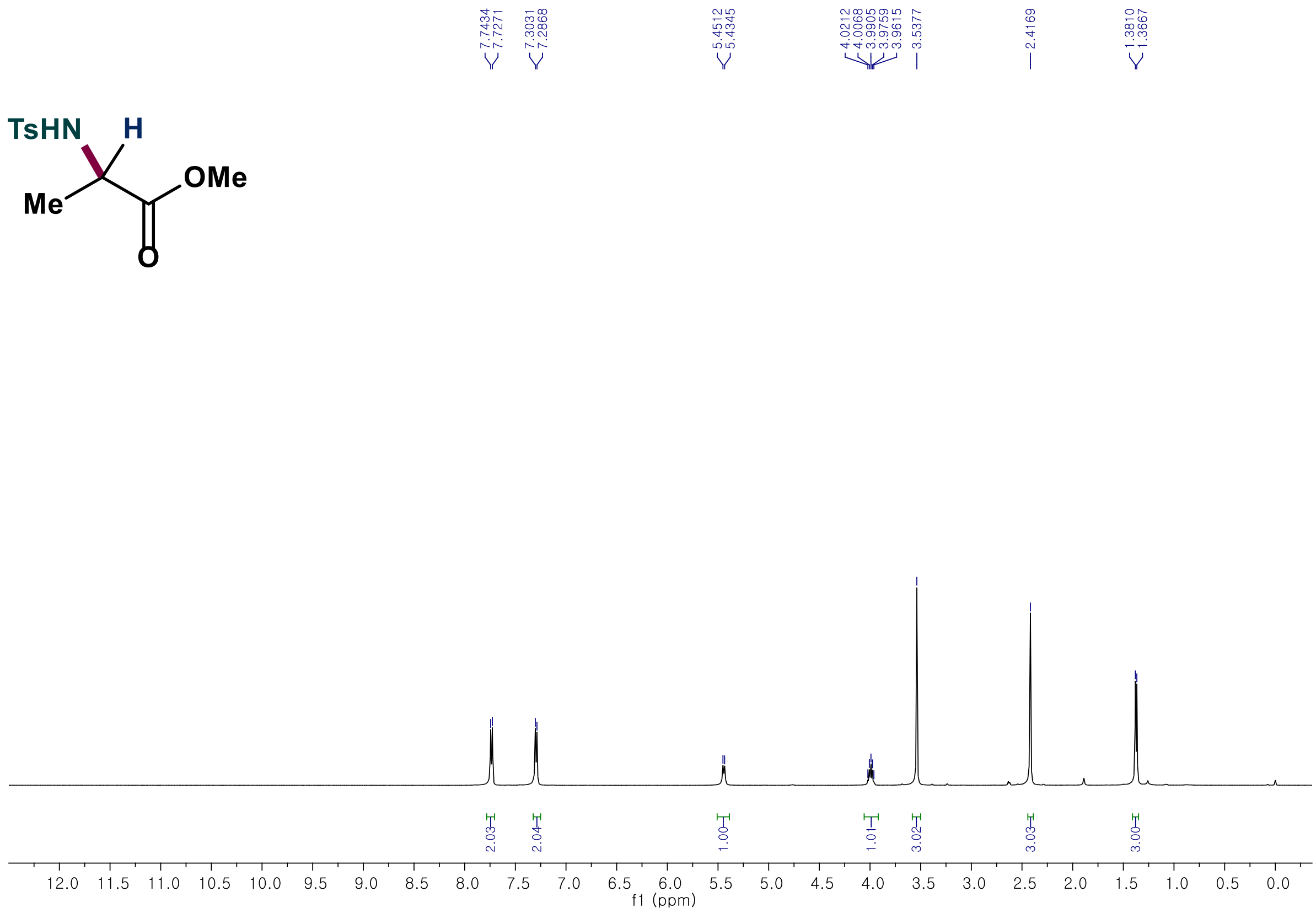

^{13}C NMR spectra (**15**)

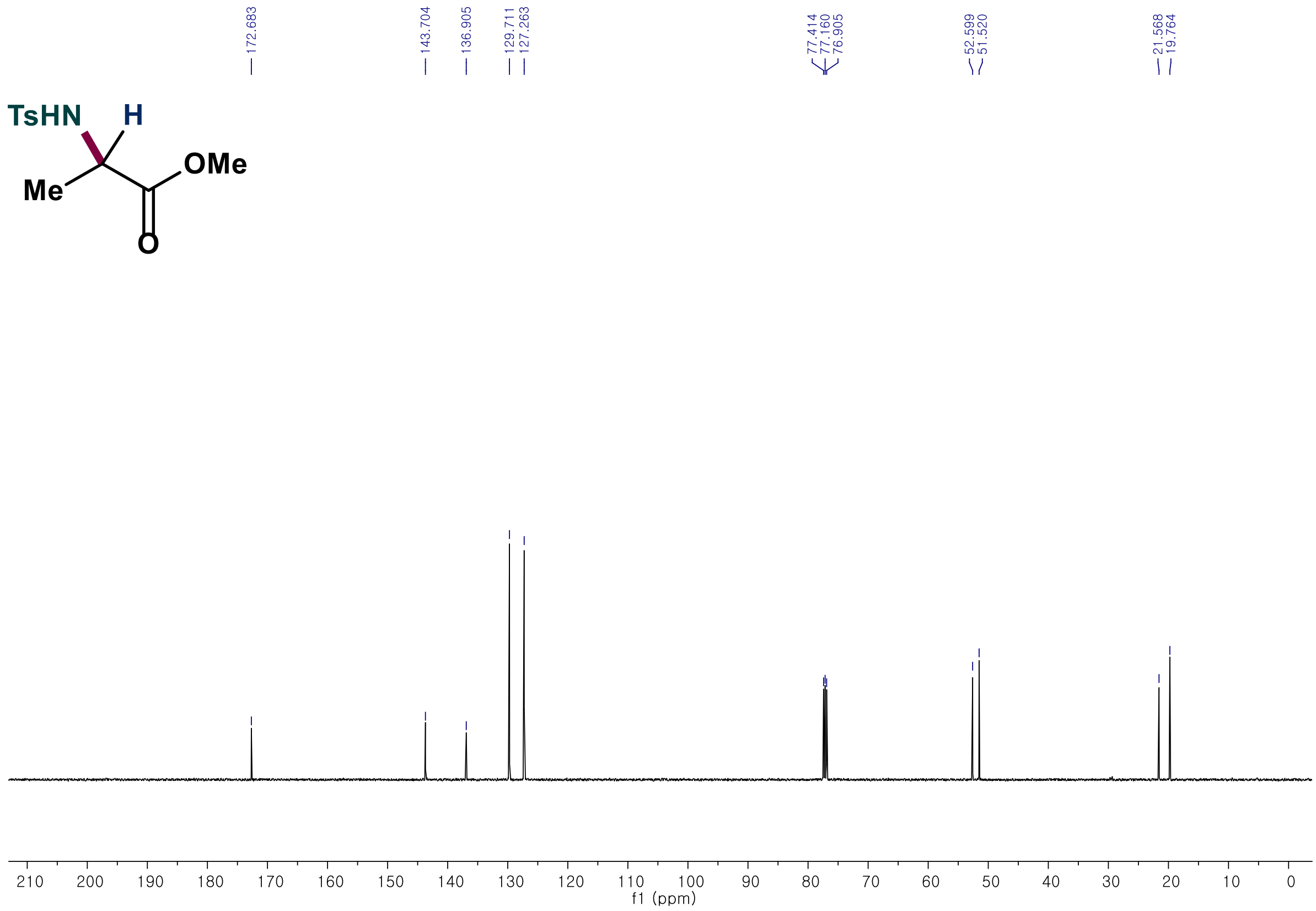

^{1}H NMR spectra (**16**)

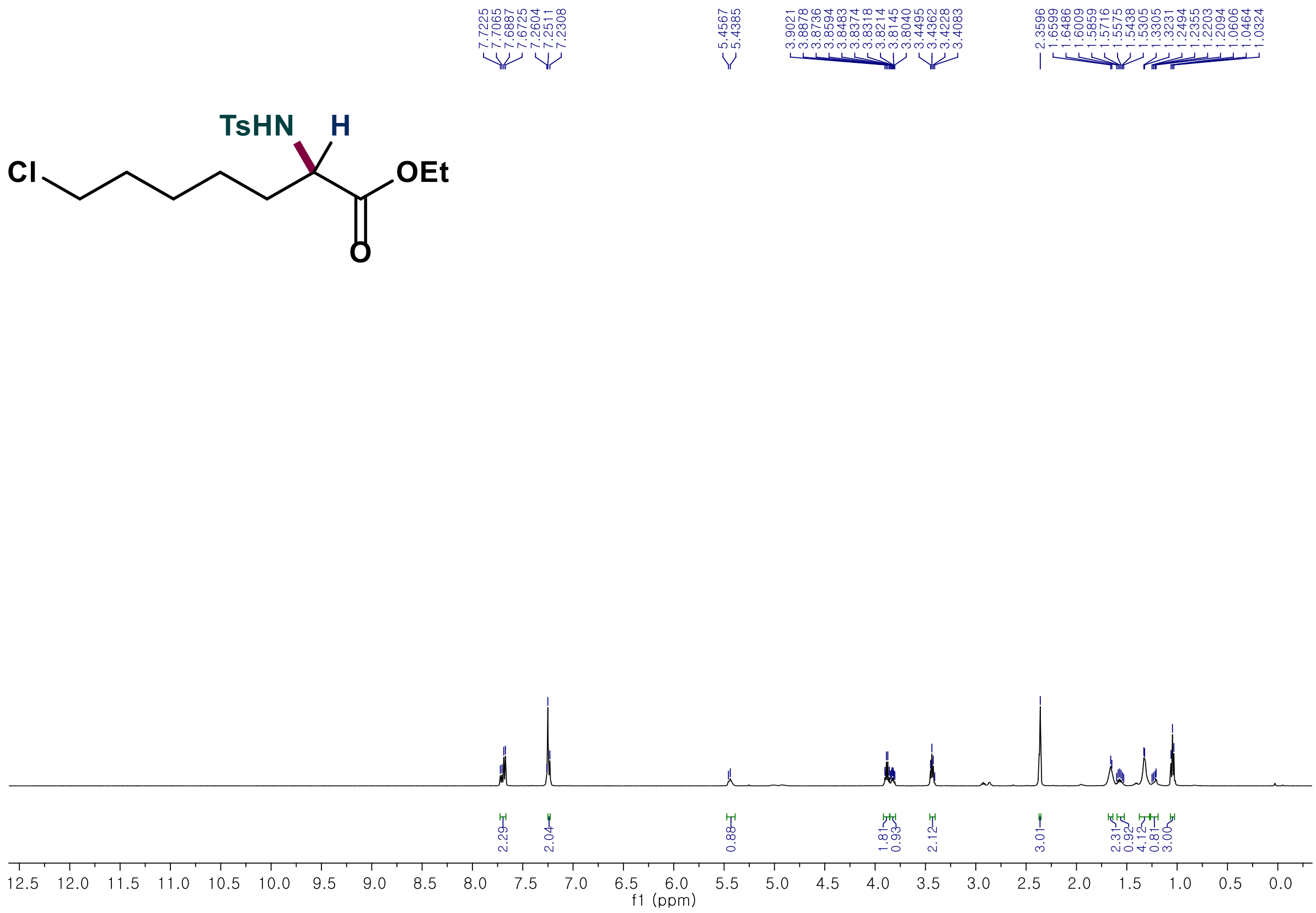

^{13}C NMR spectra (**16**)

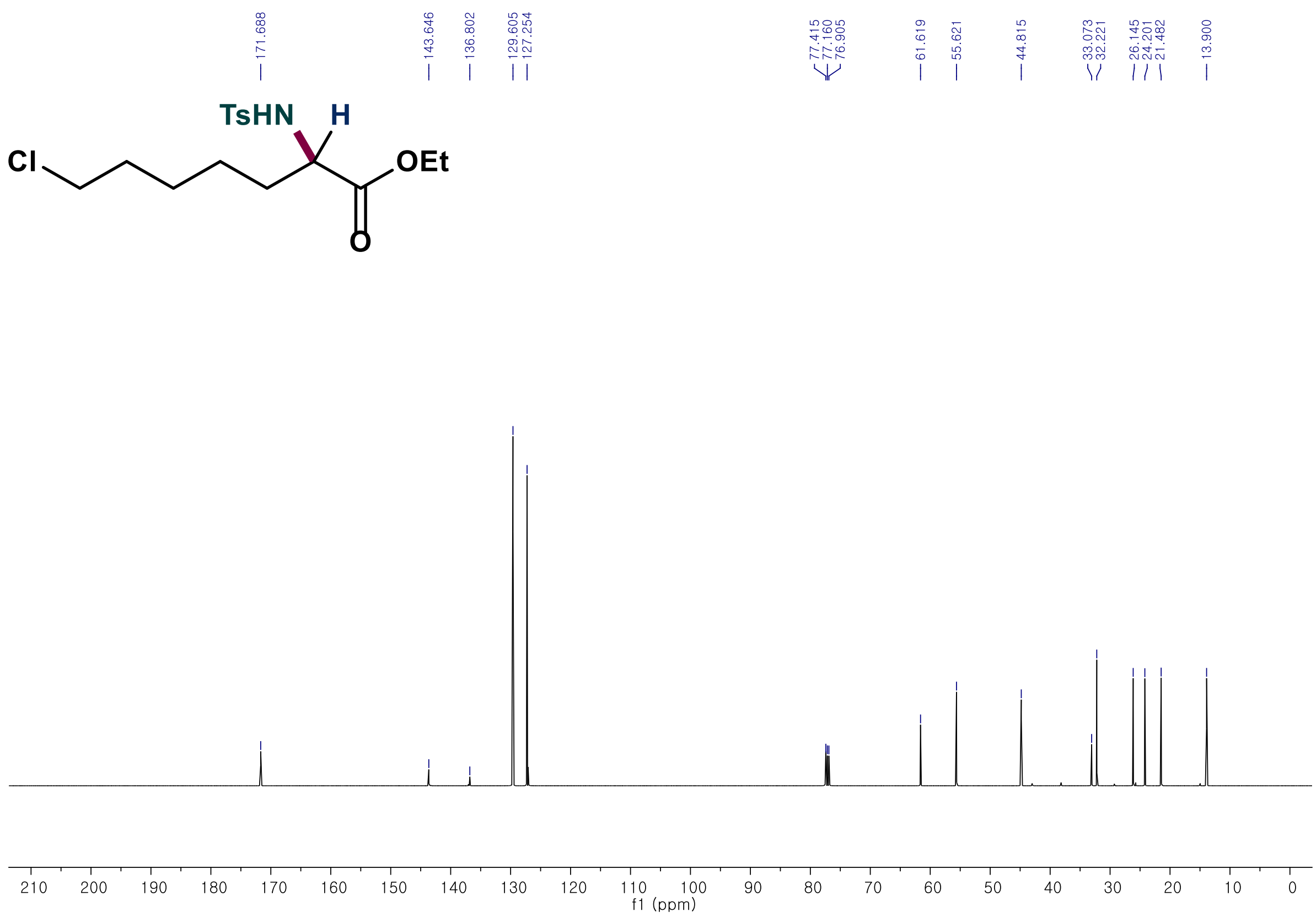

^{1}H NMR spectra (**17**)

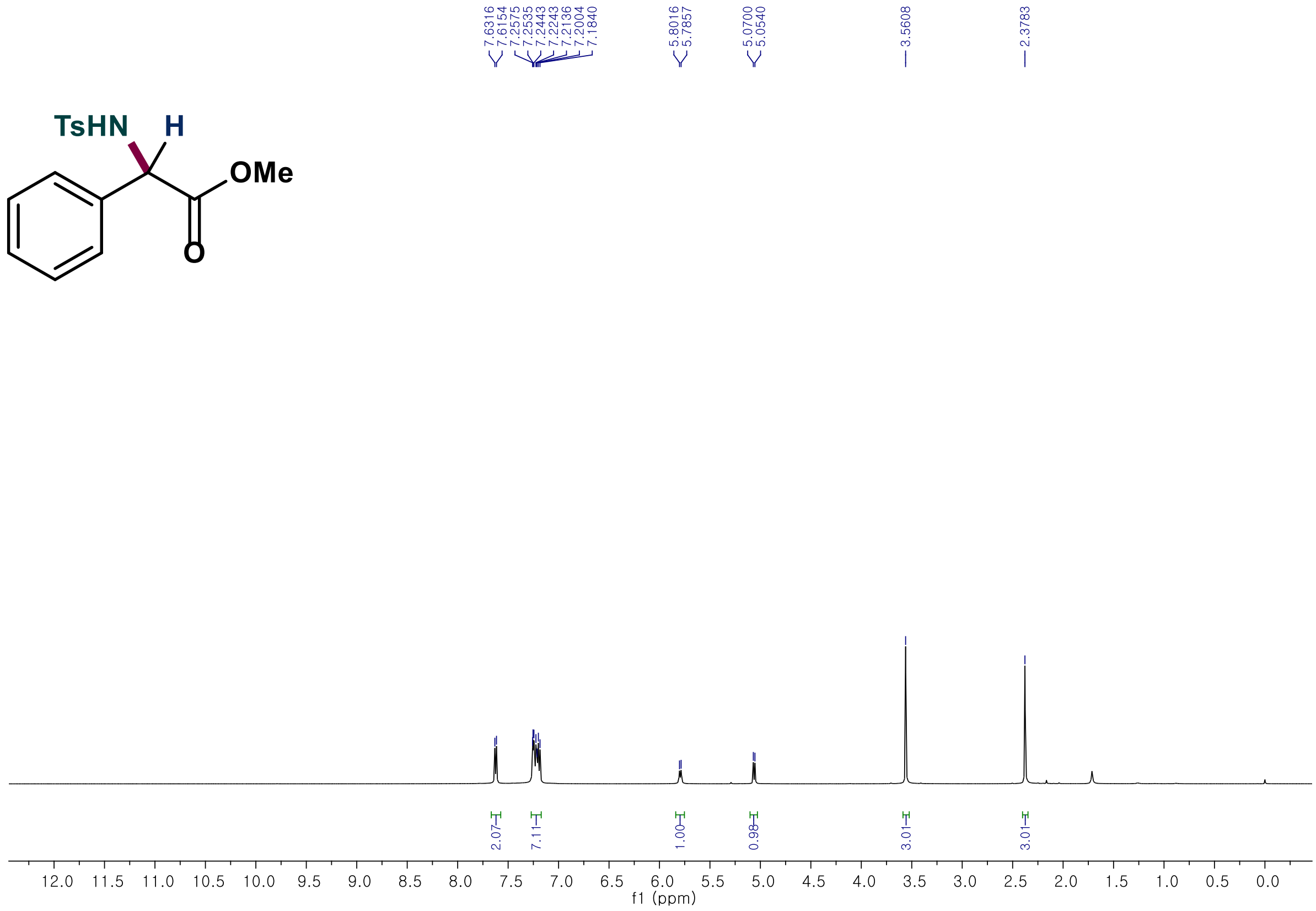

^{13}C NMR spectra (**17**)

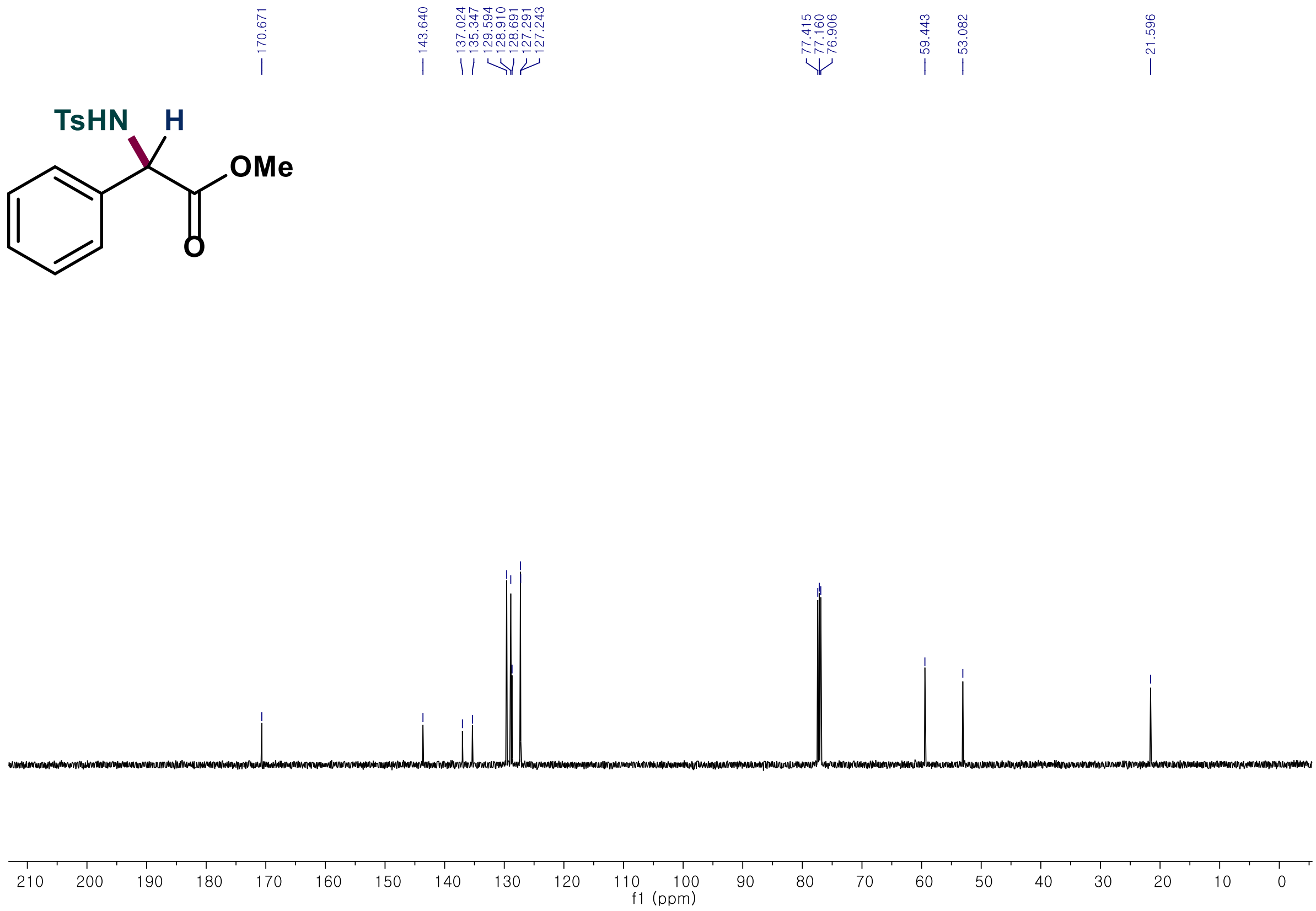

^1H NMR spectra (**18**)

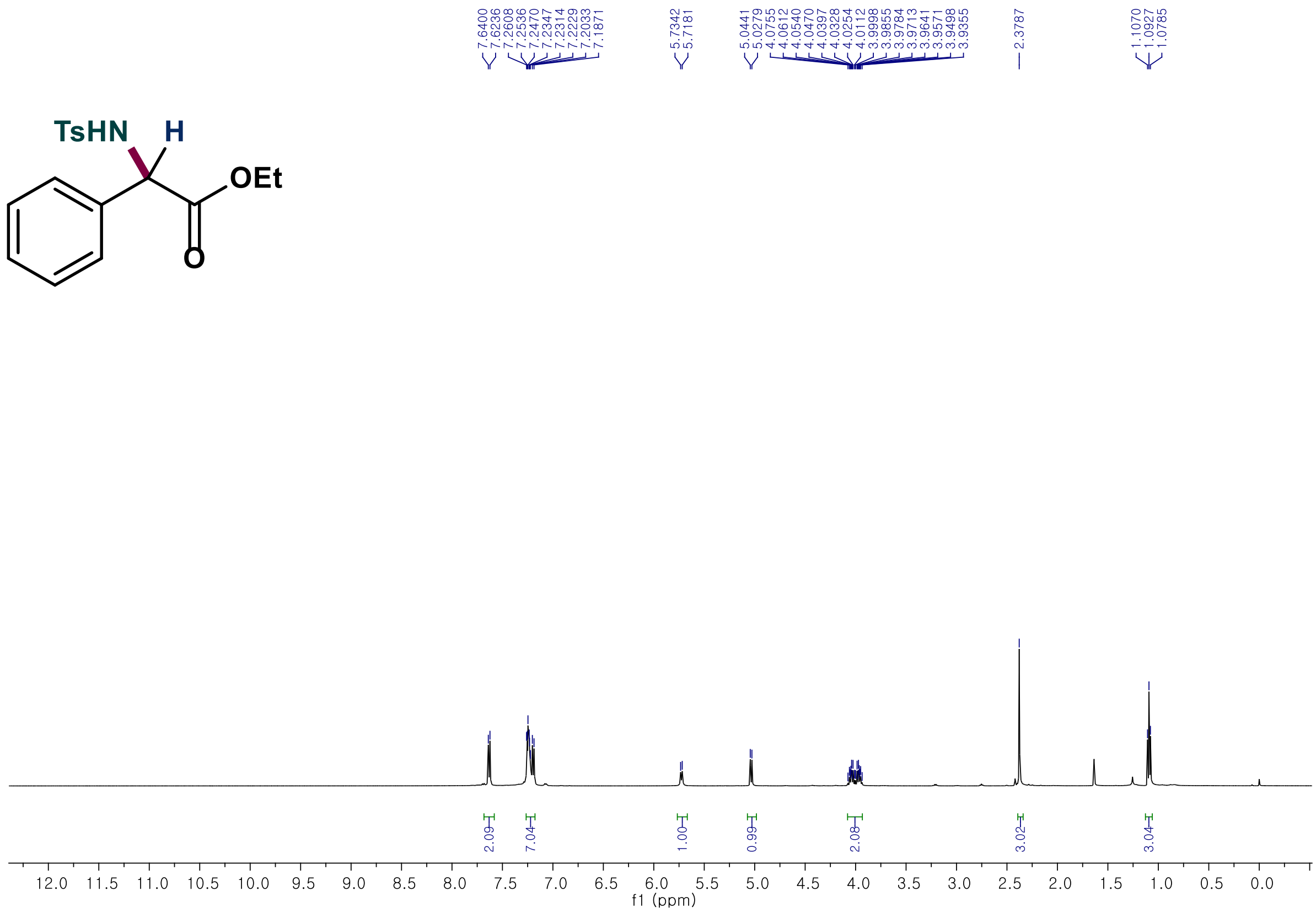

^{13}C NMR spectra (**18**)

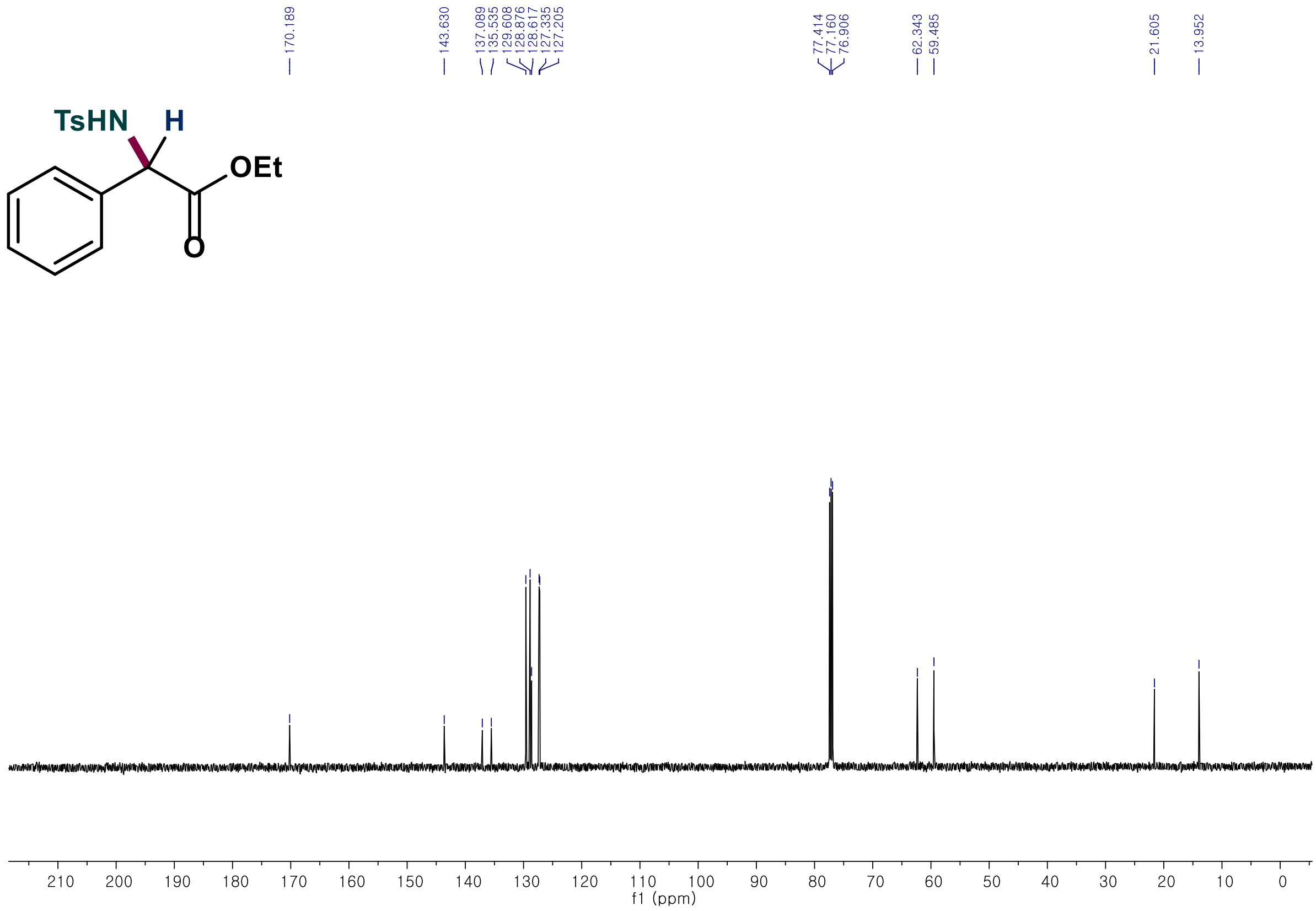

^{1}H NMR spectra (**19**)

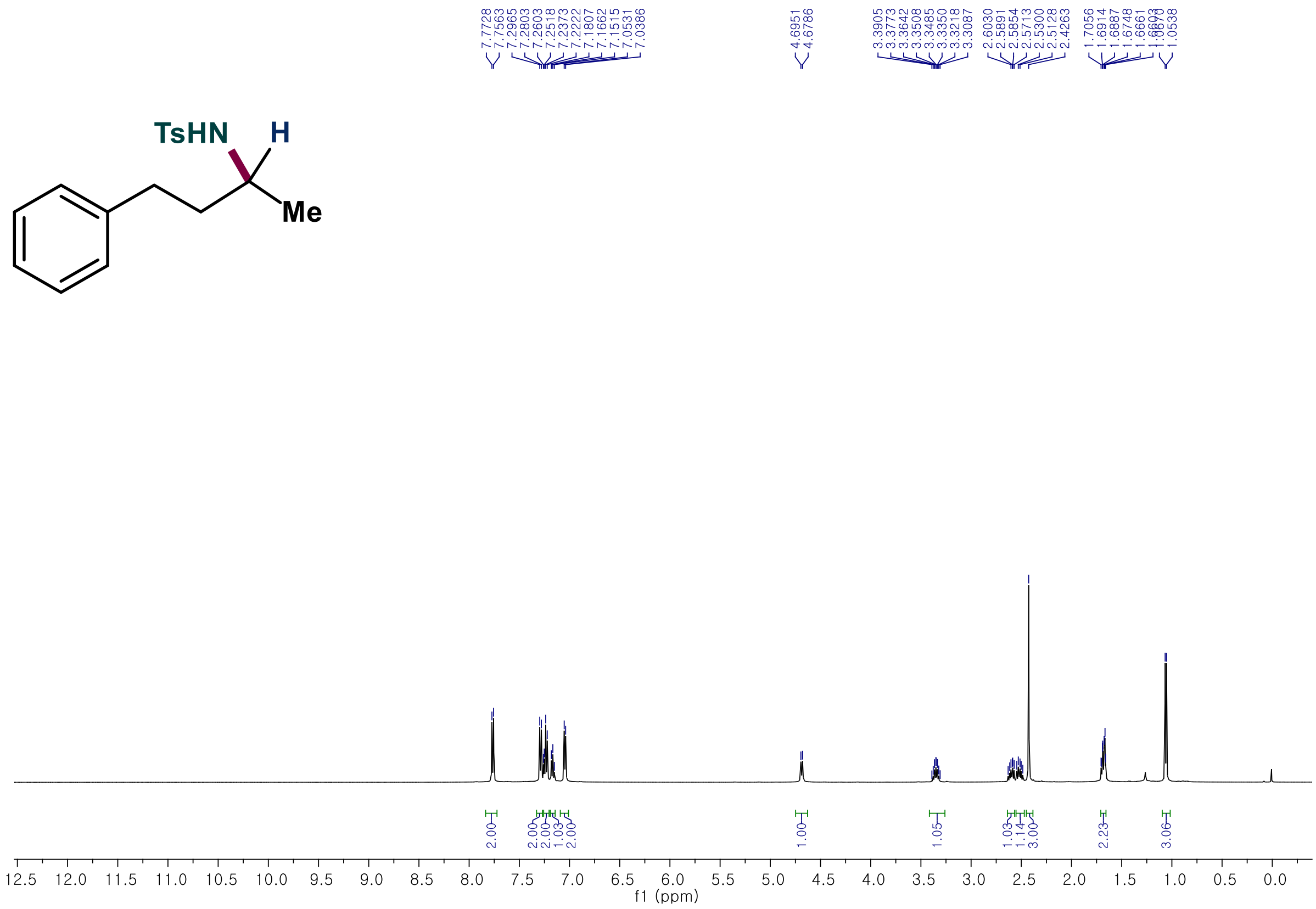

^{13}C NMR spectra (**19**)

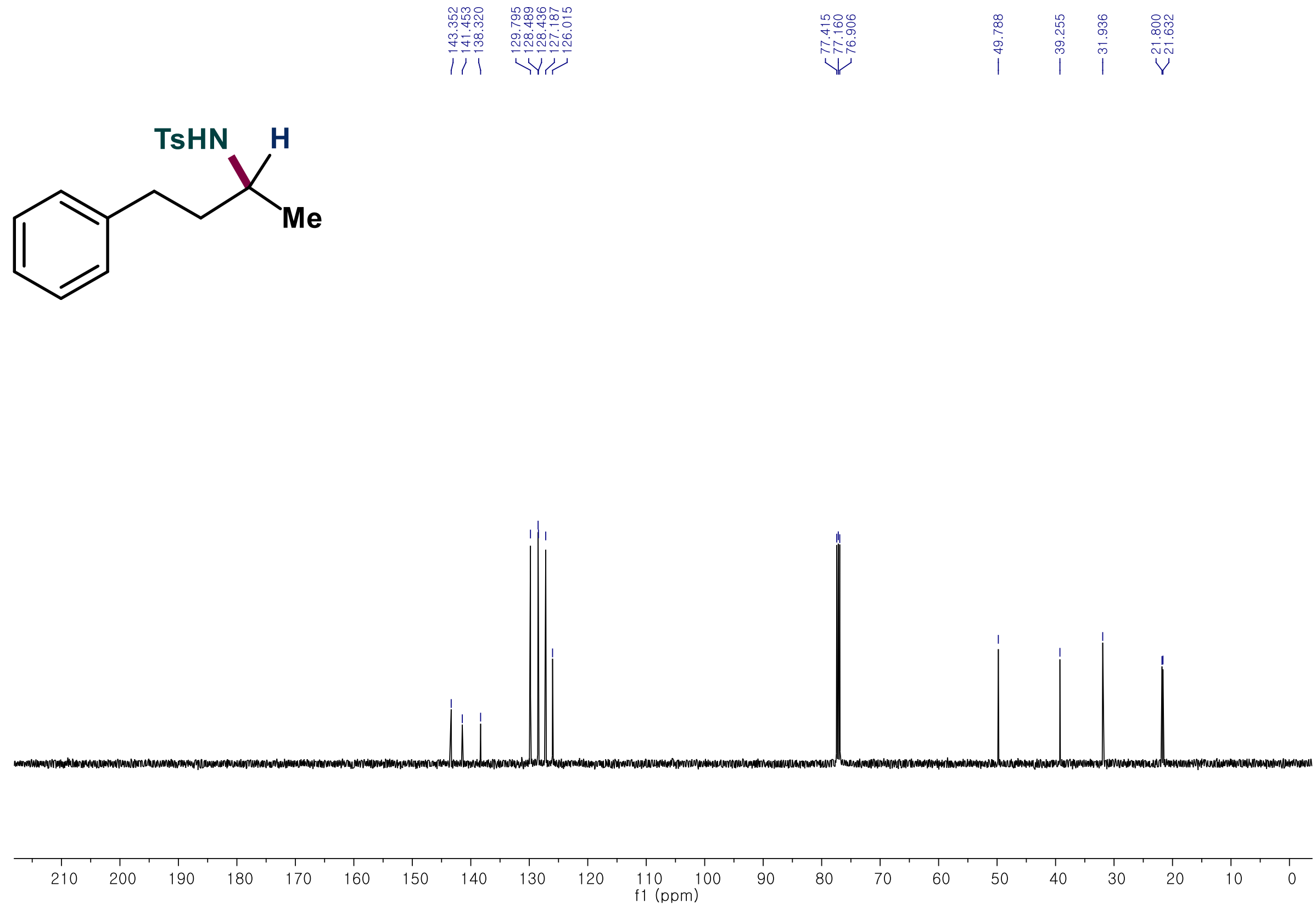

^{1}H NMR spectra (**20**)

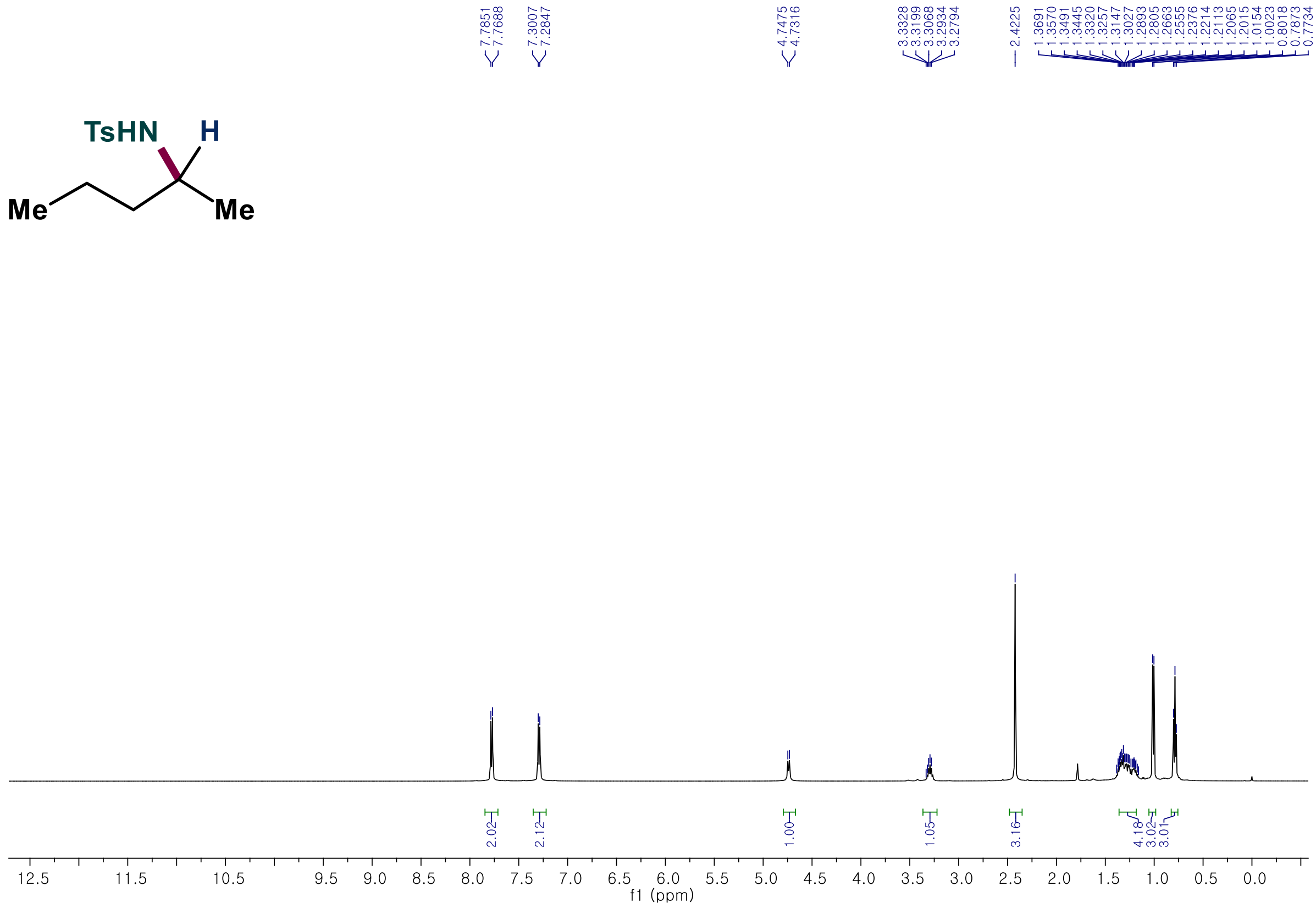

^{13}C NMR spectra (**20**)

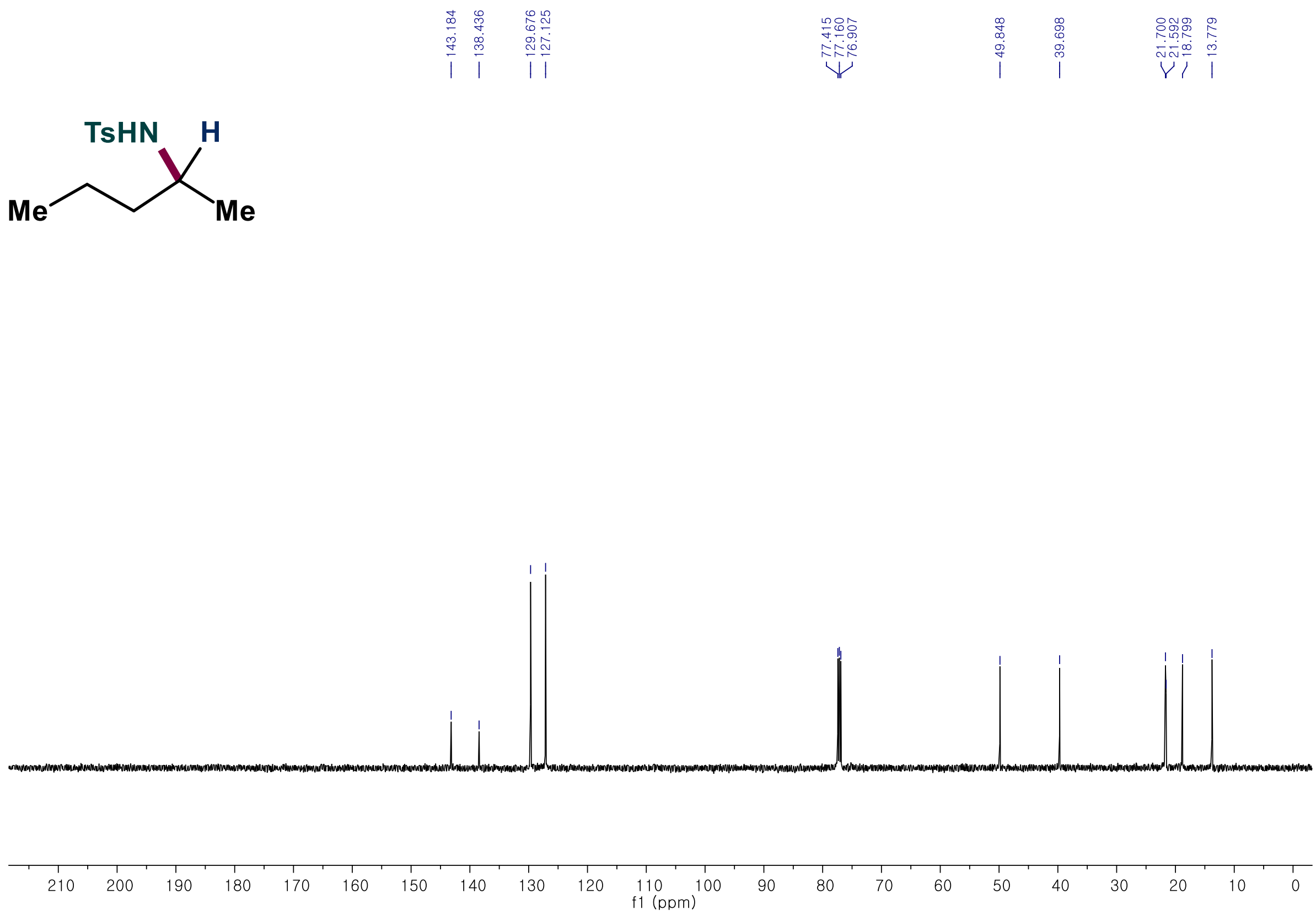

^{1}H NMR spectra (**21**)

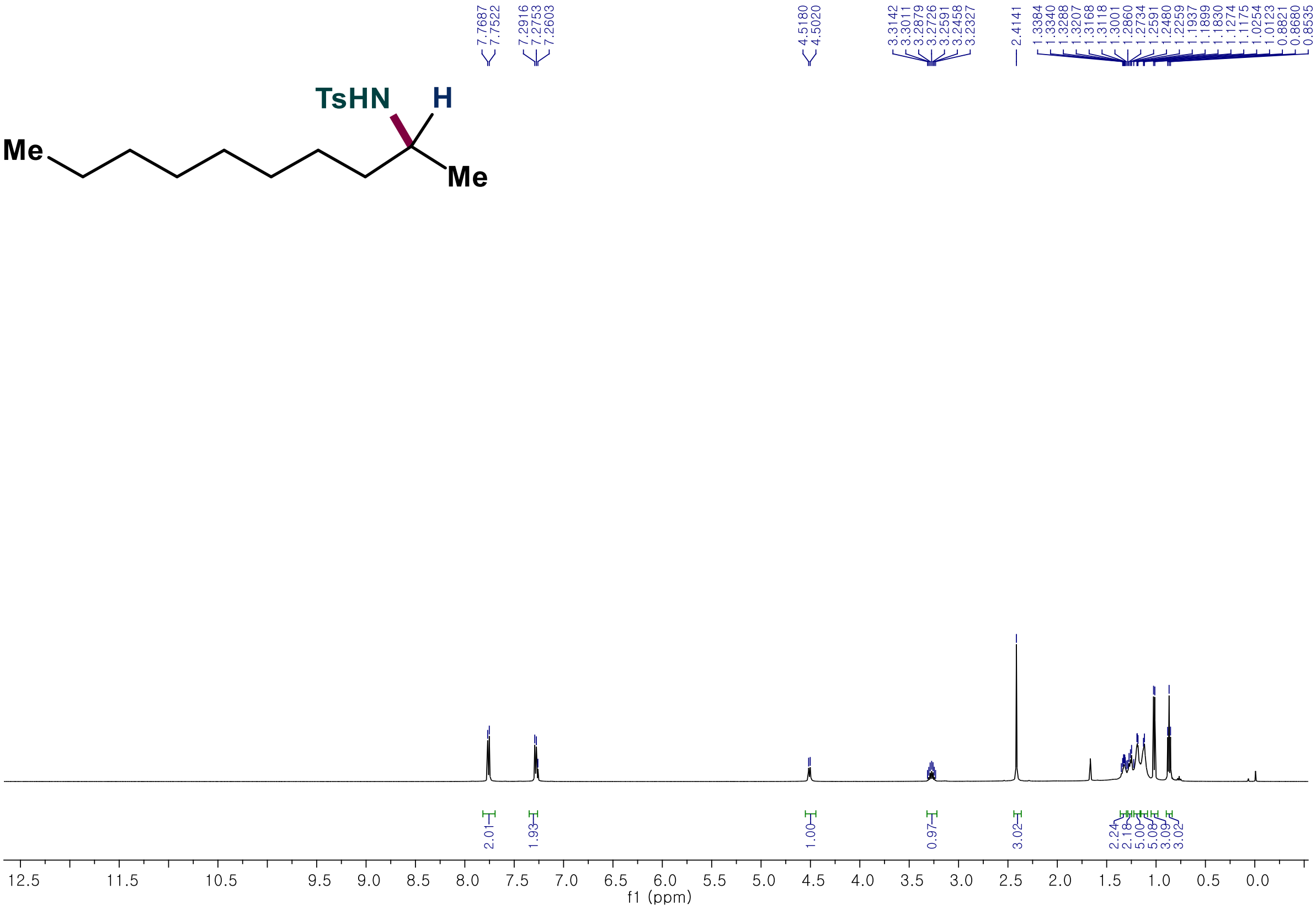

^{13}C NMR spectra (**21**)

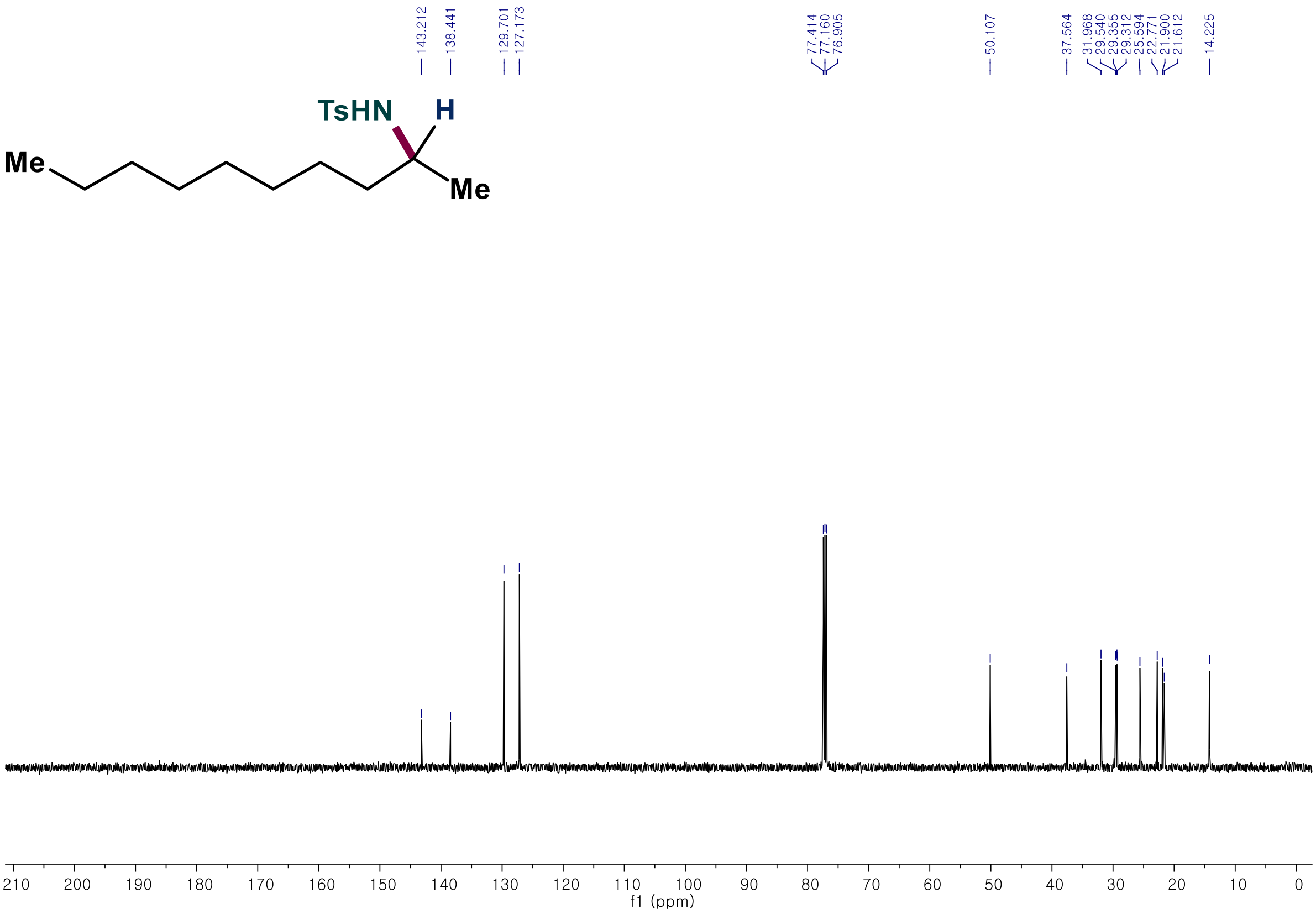

^{1}H NMR spectra (**22**)

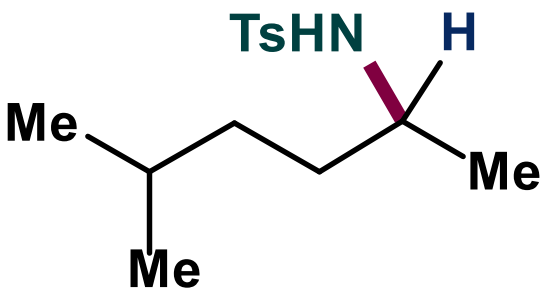

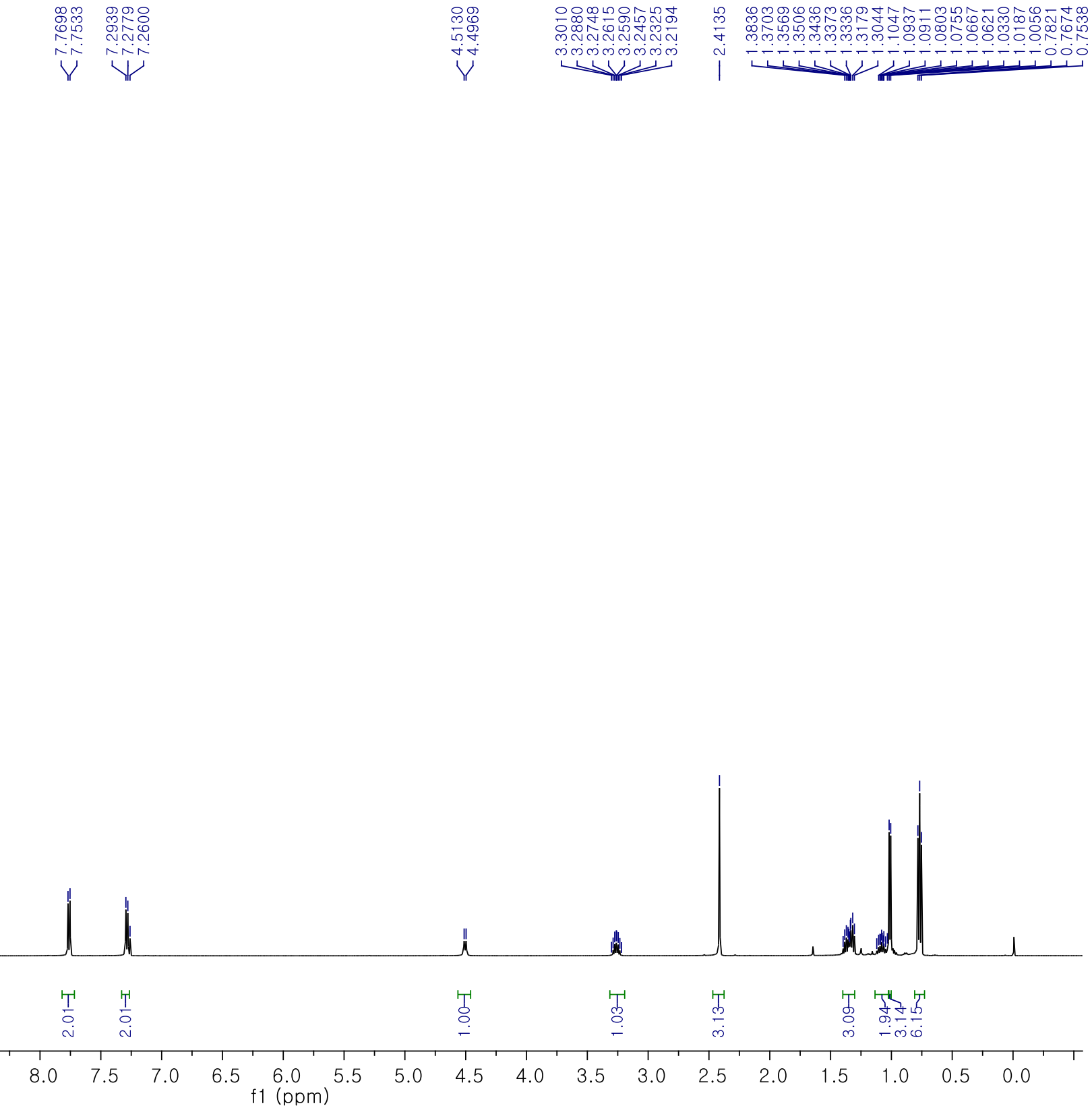

^{13}C NMR spectra (**22**)

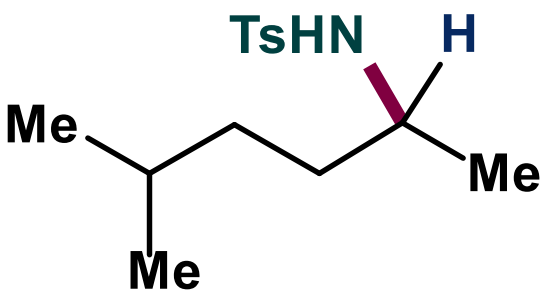

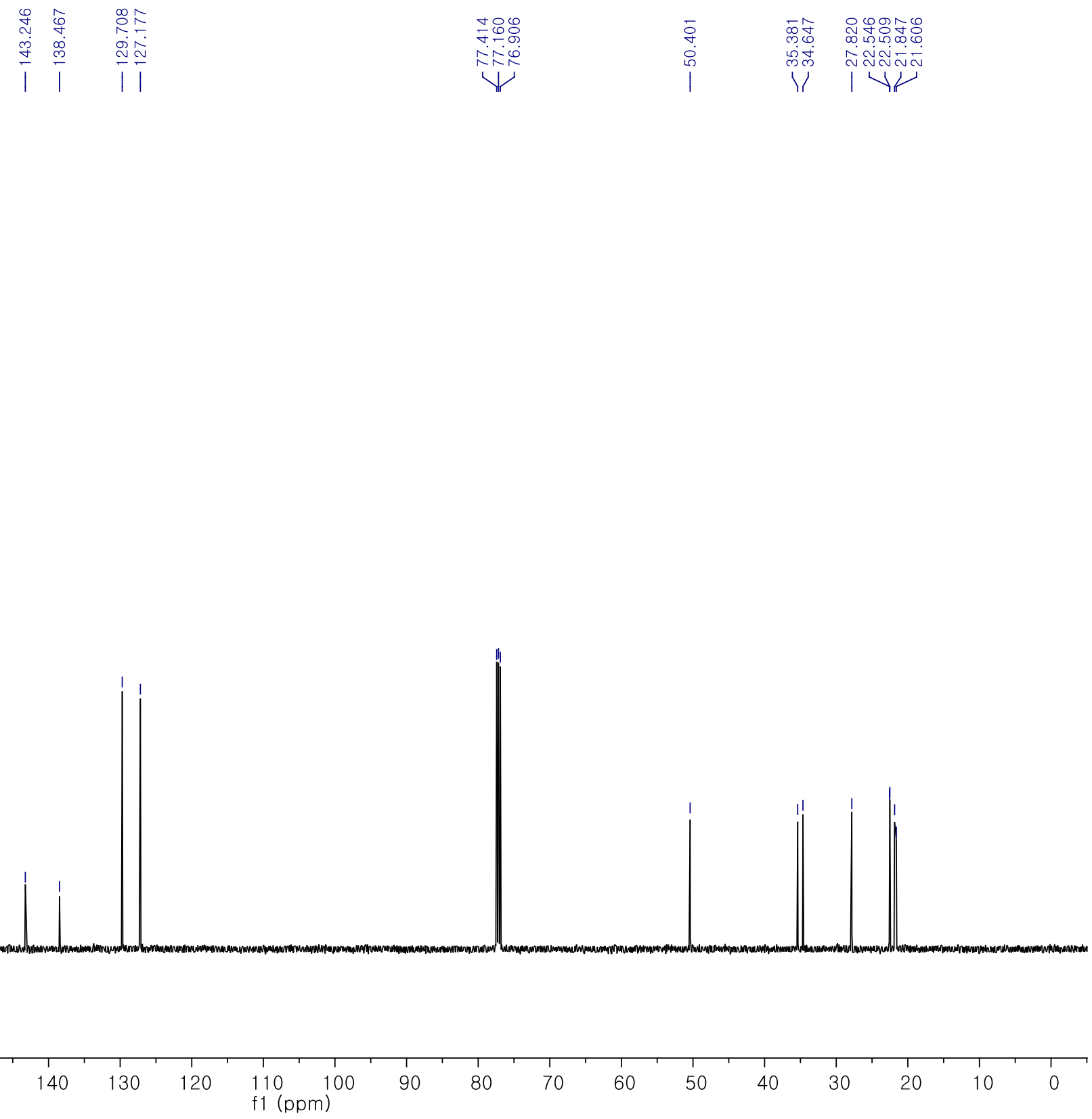

^{1}H NMR spectra (**23**)

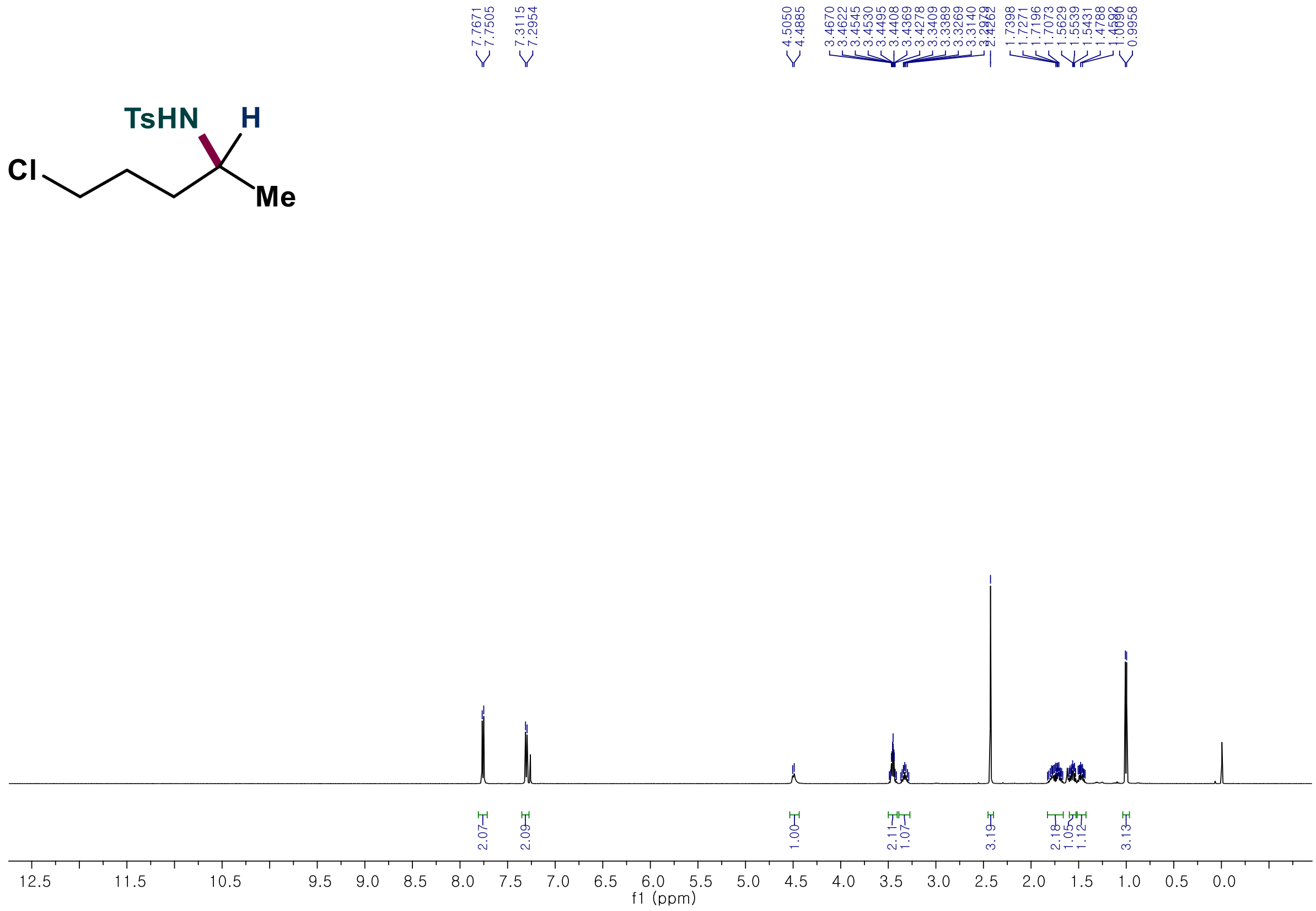

^{13}C NMR spectra (**23**)

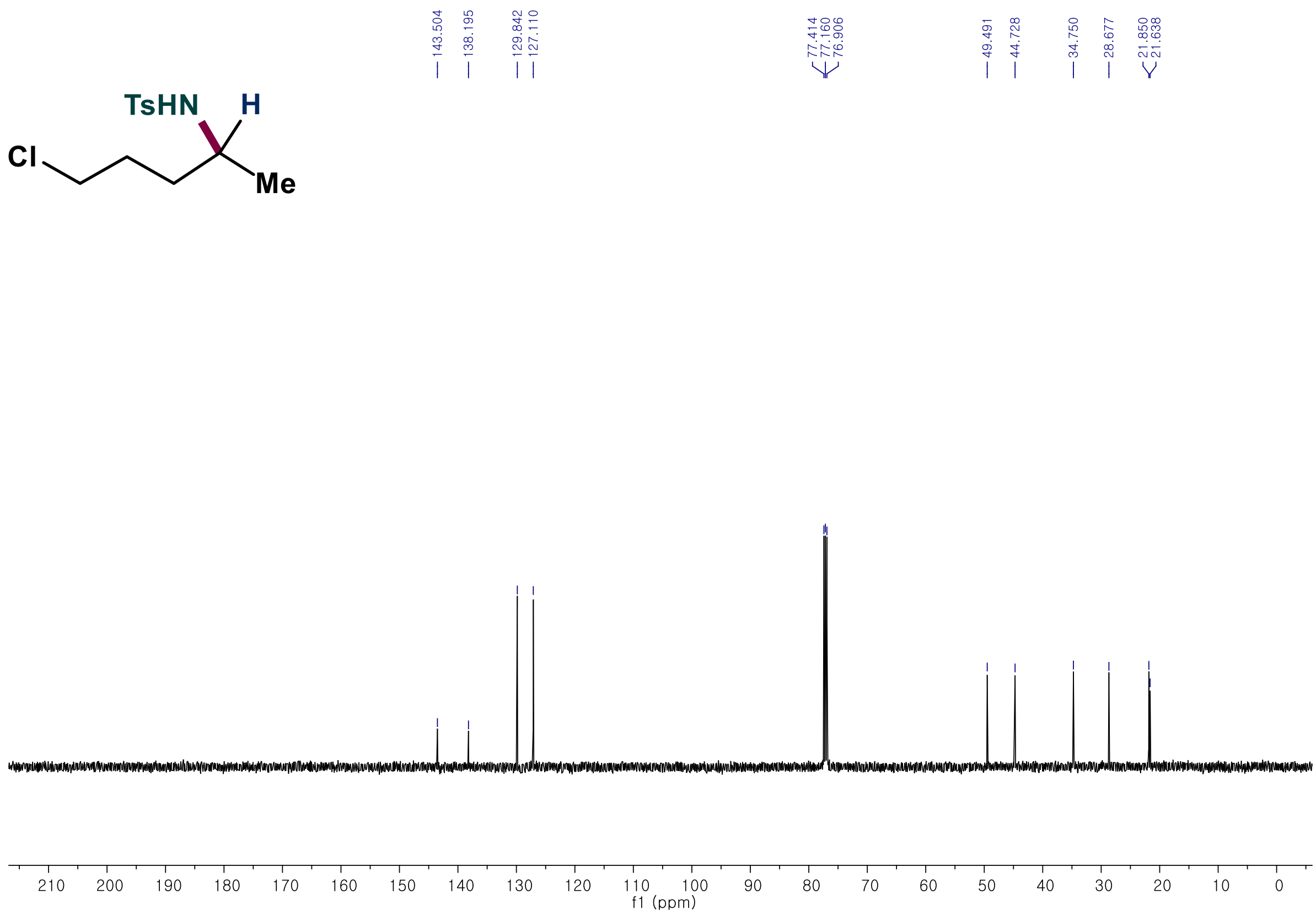

^{1}H NMR spectra (**24**)

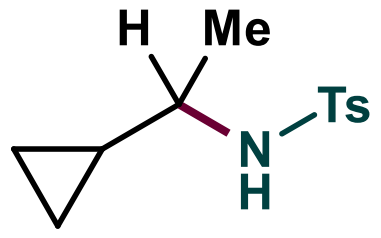

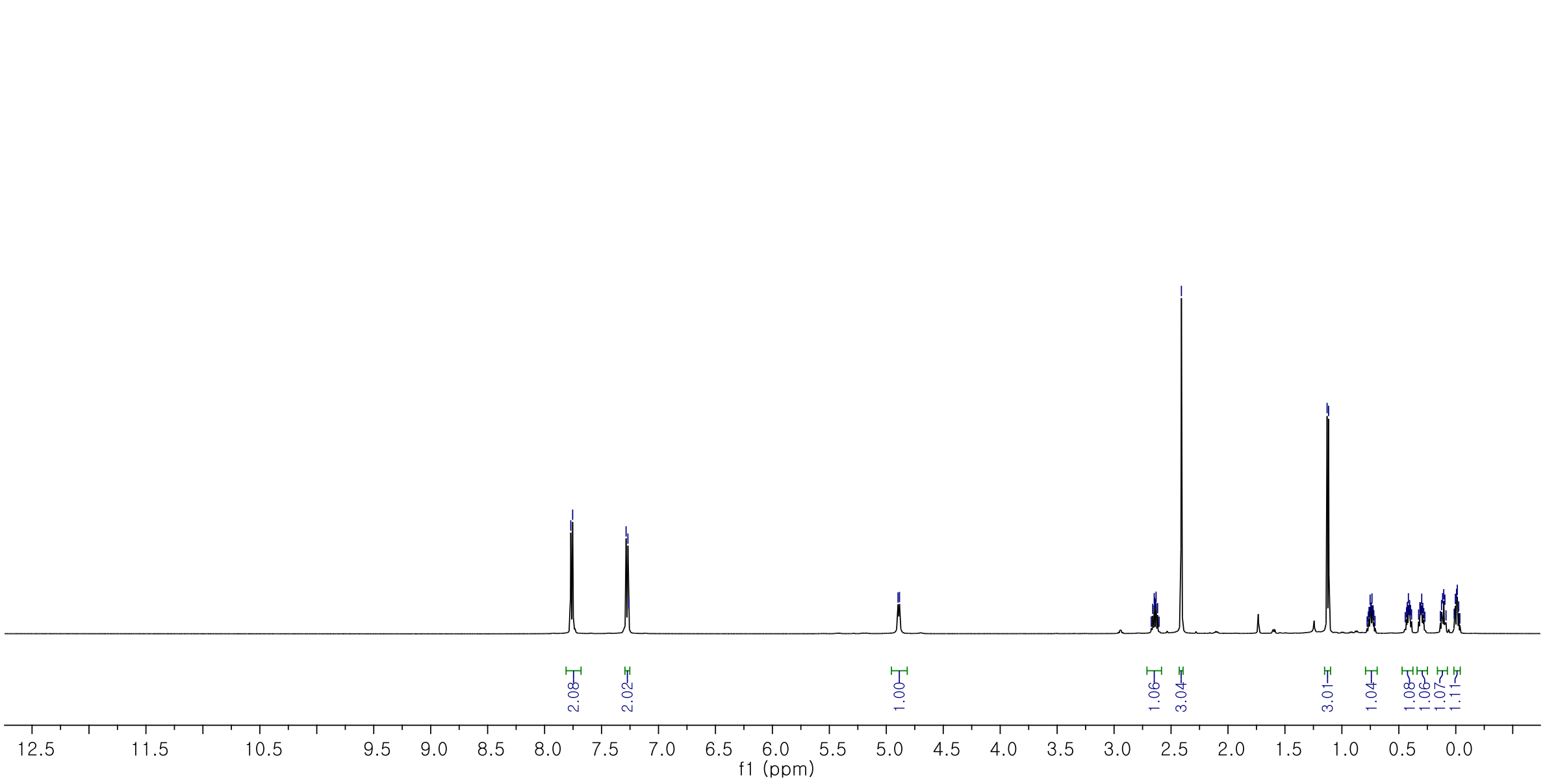

^{13}C NMR spectra (**24**)

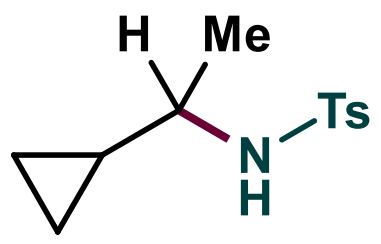

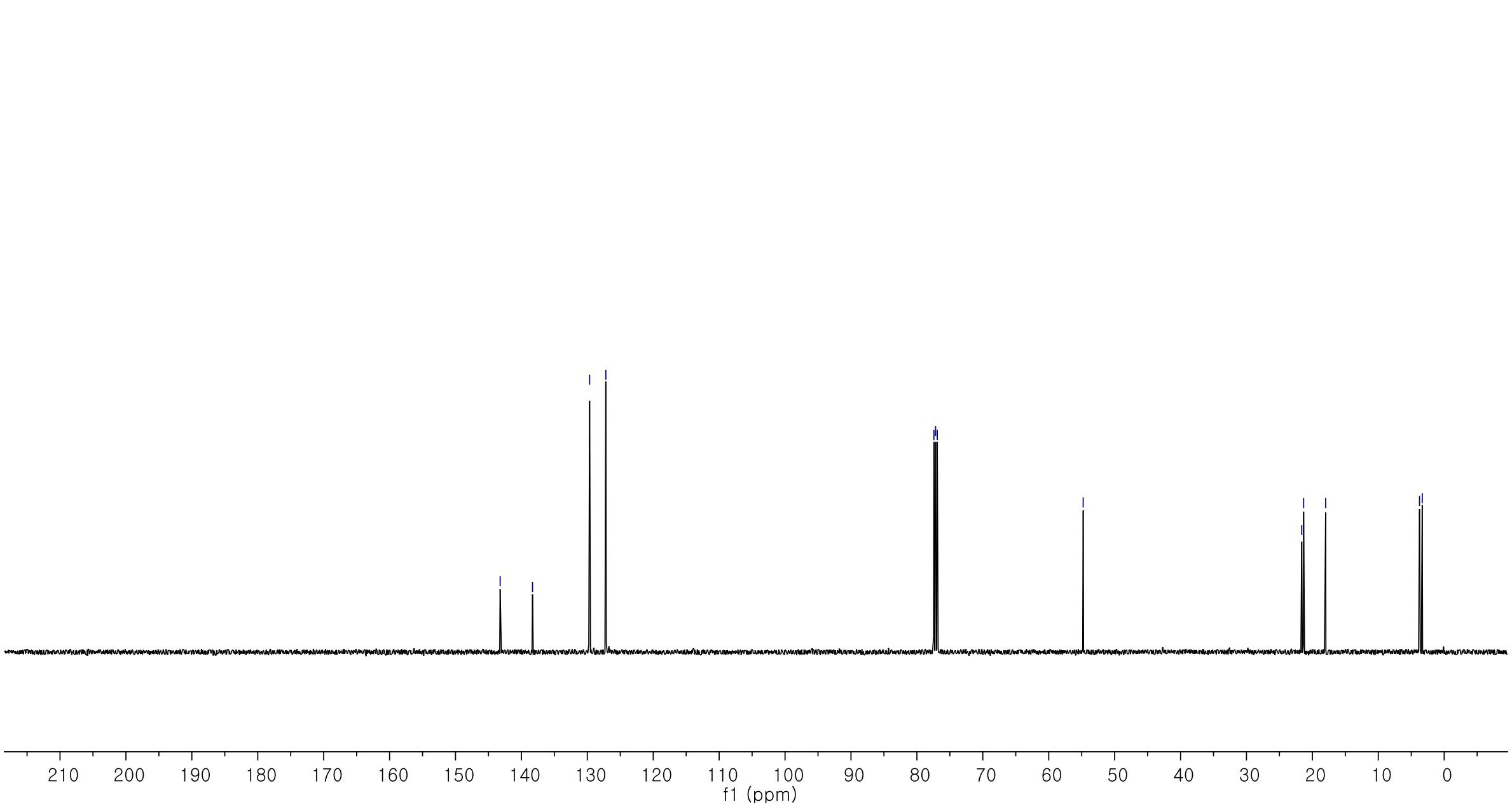

^{1}H NMR spectra (**25**)

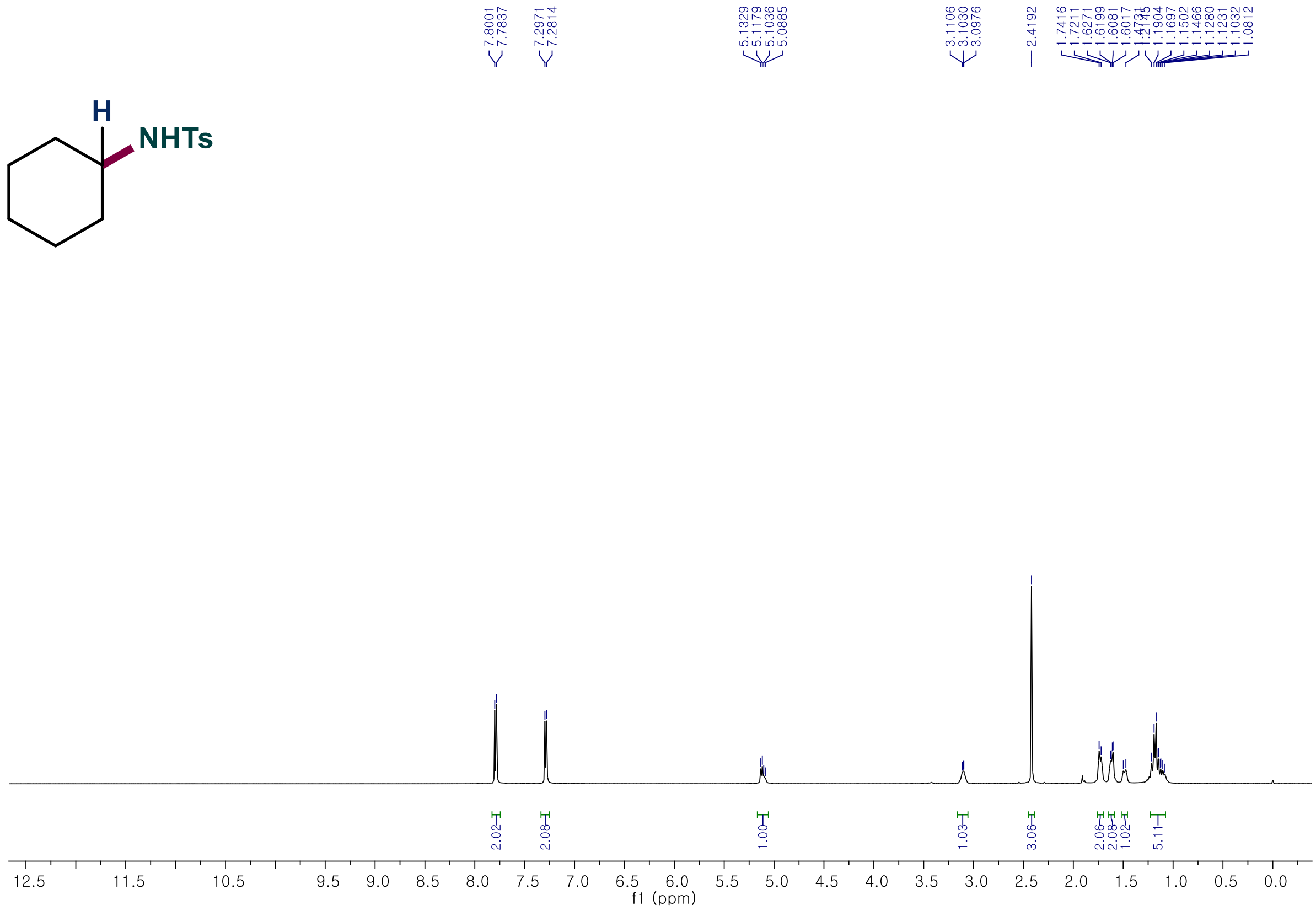

^{13}C NMR spectra (**25**)

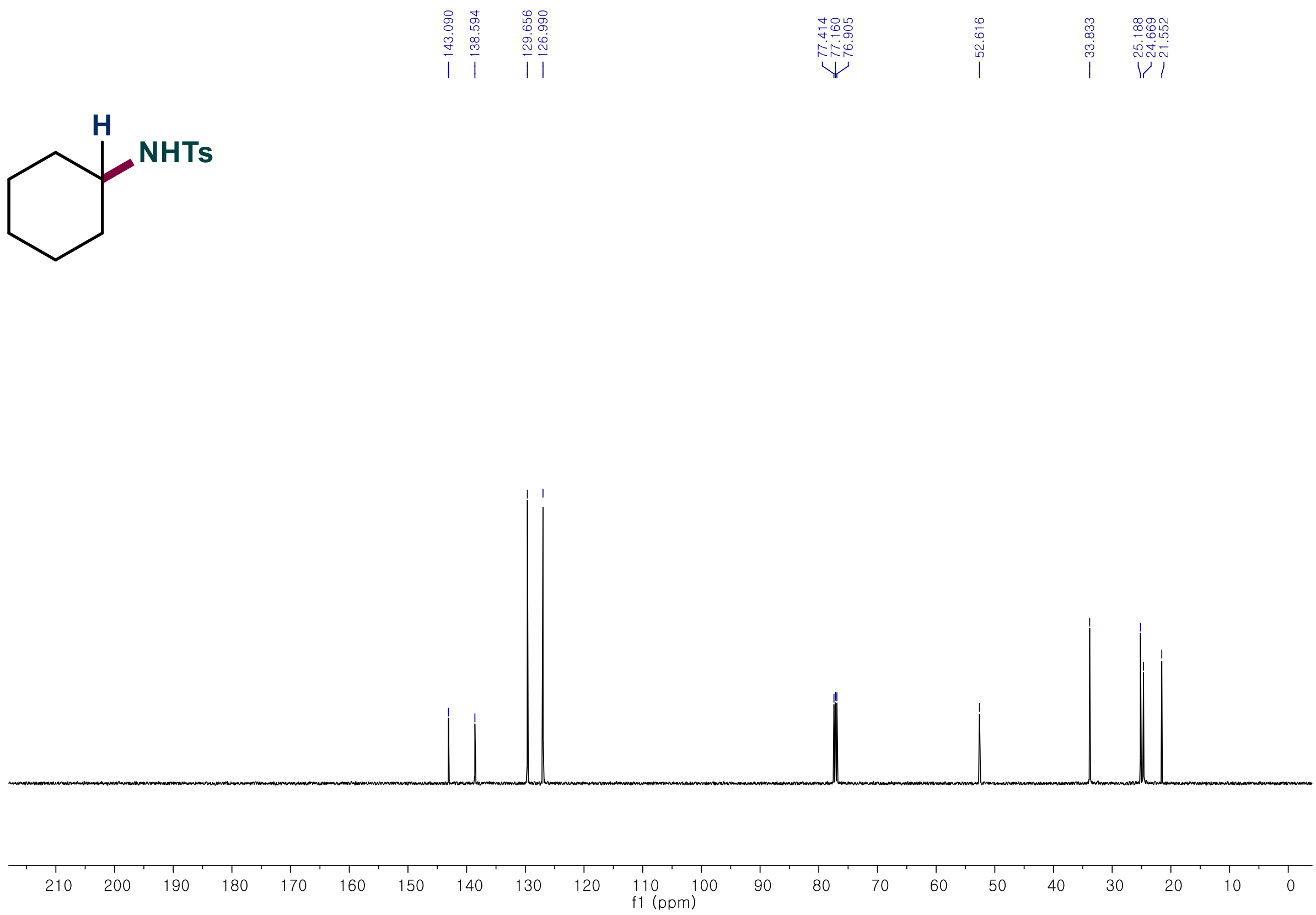

^{1}H NMR spectra (**26**)

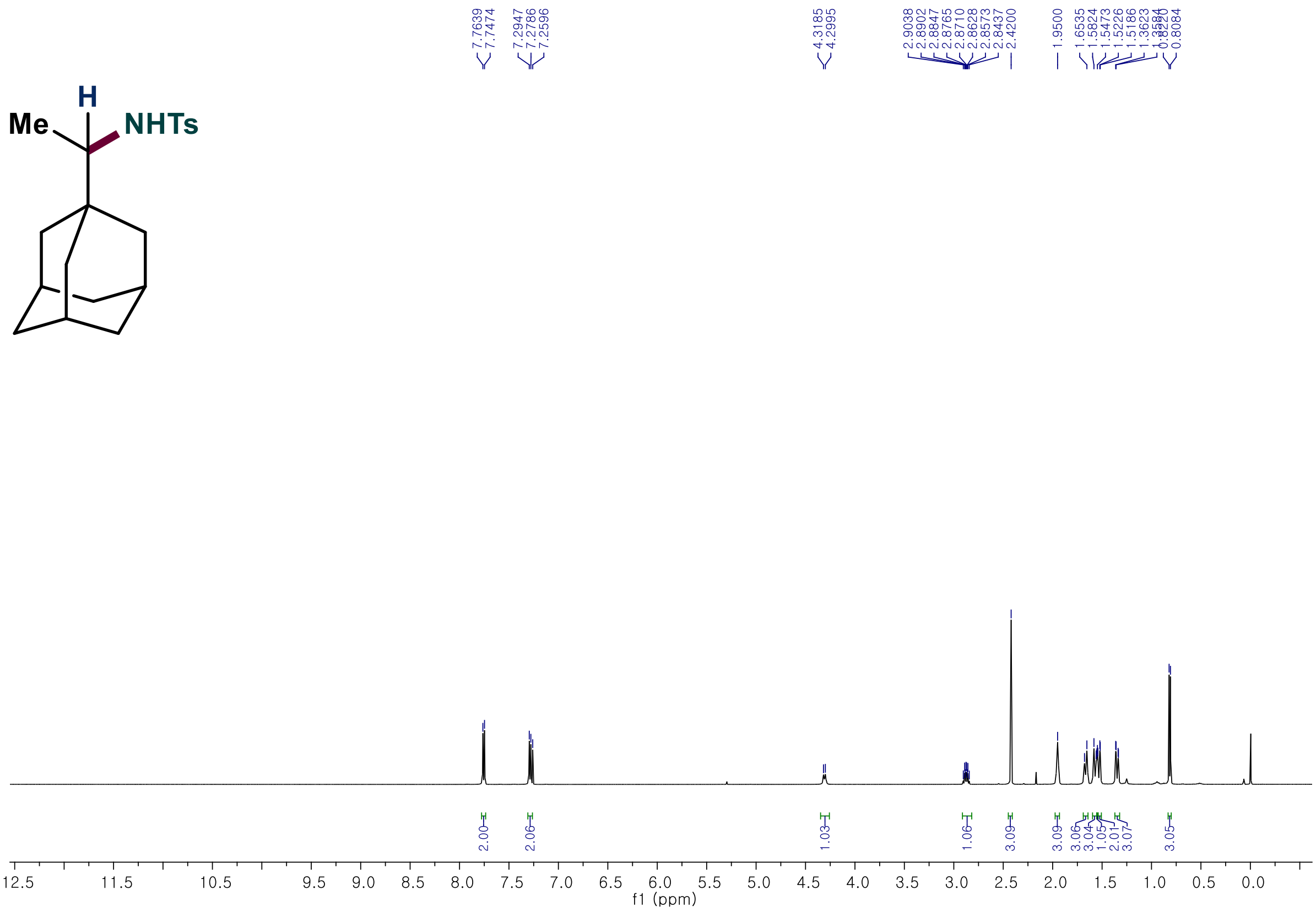

^{13}C NMR spectra (**26**)

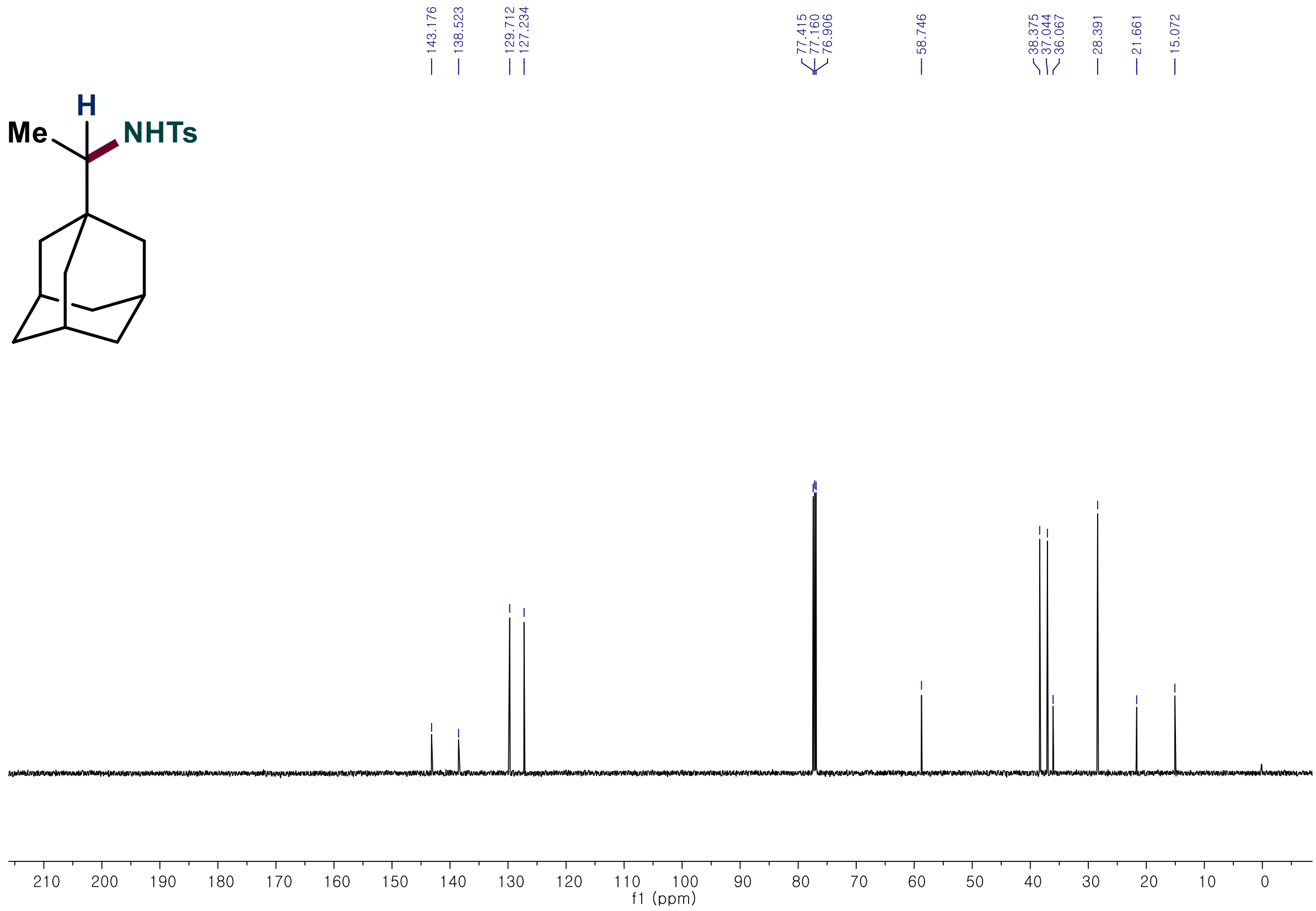

^{1}H NMR spectra (**27**)

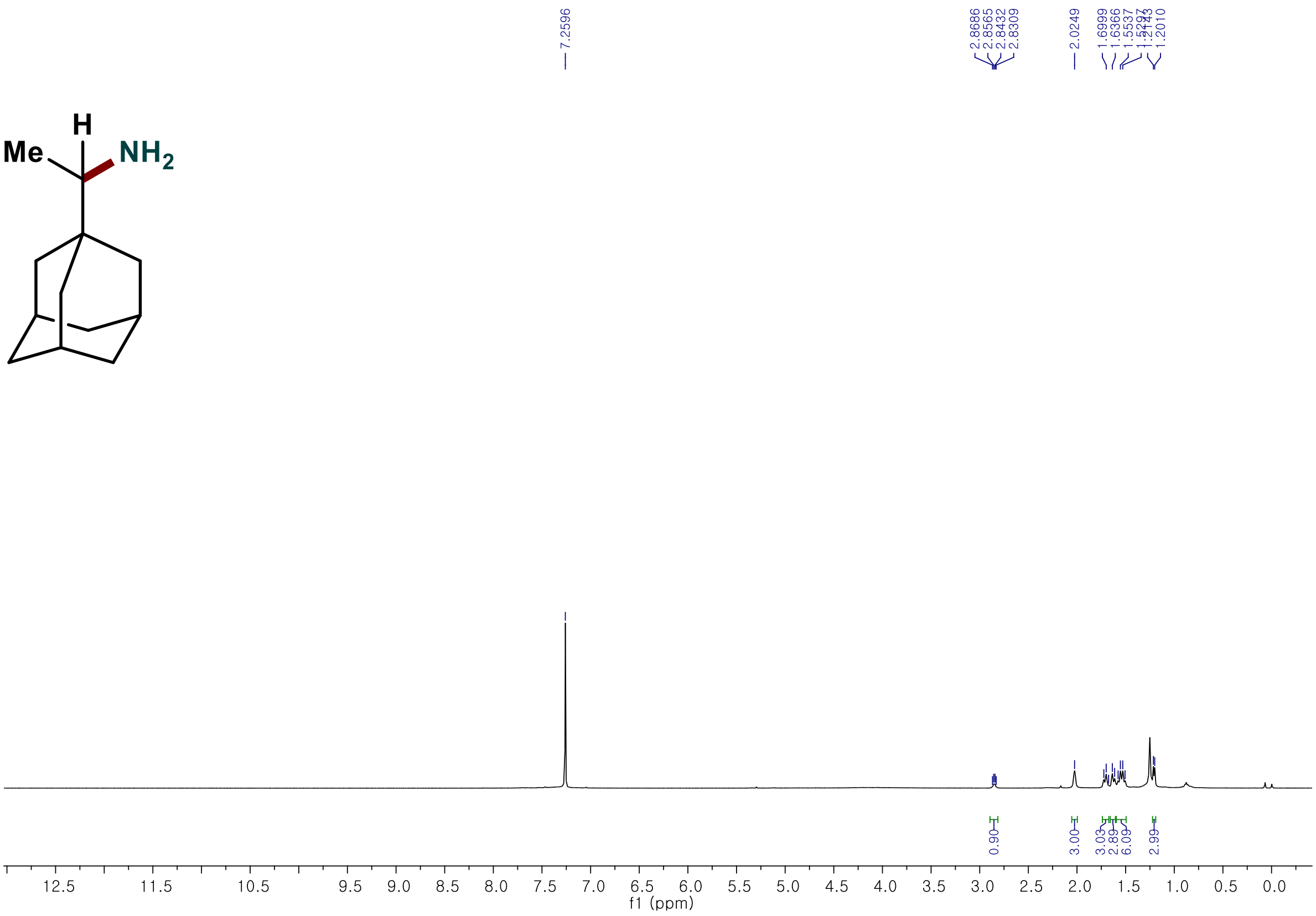

^{13}C NMR spectra (**27**)

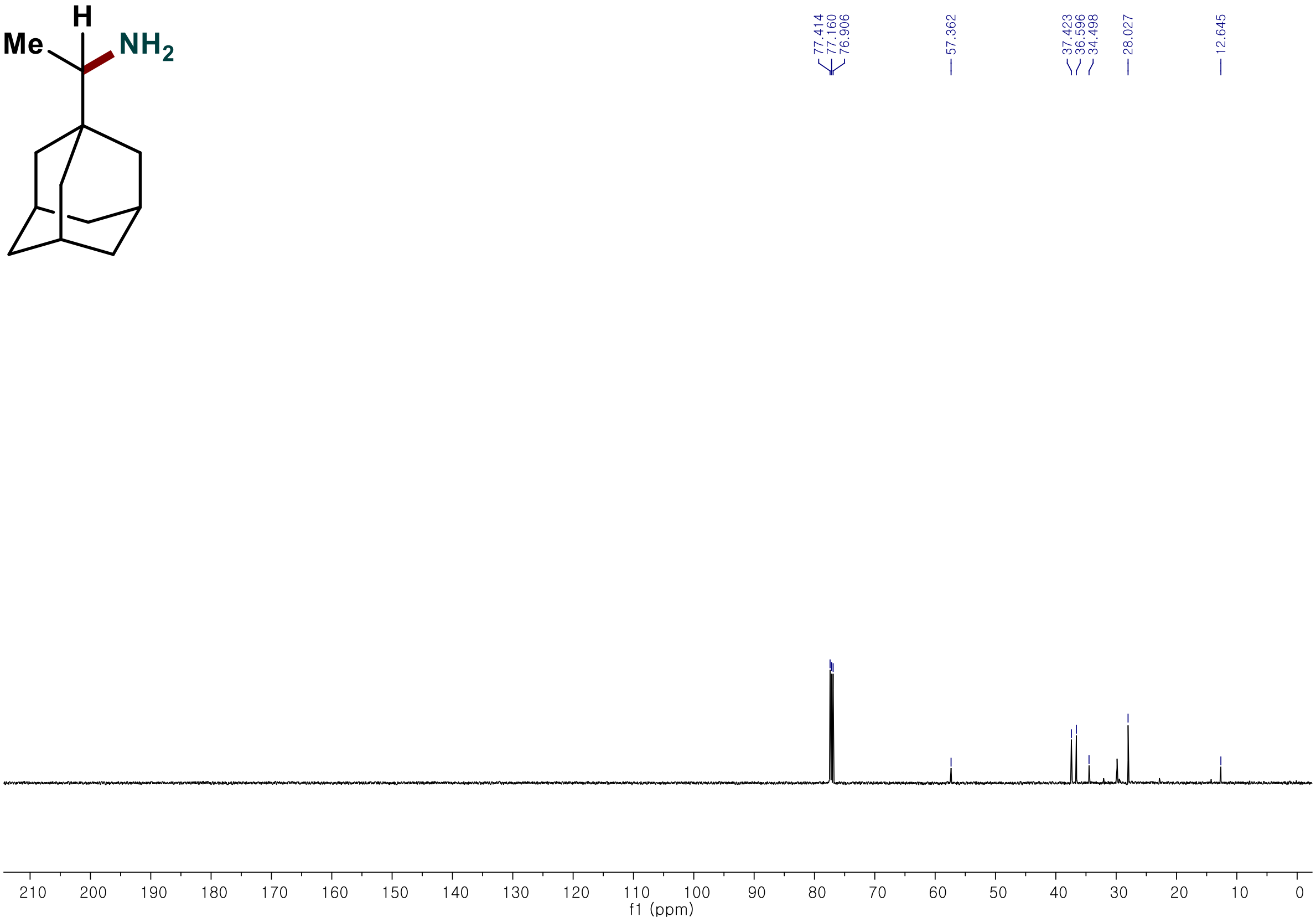

^{1}H NMR spectra (**28**)

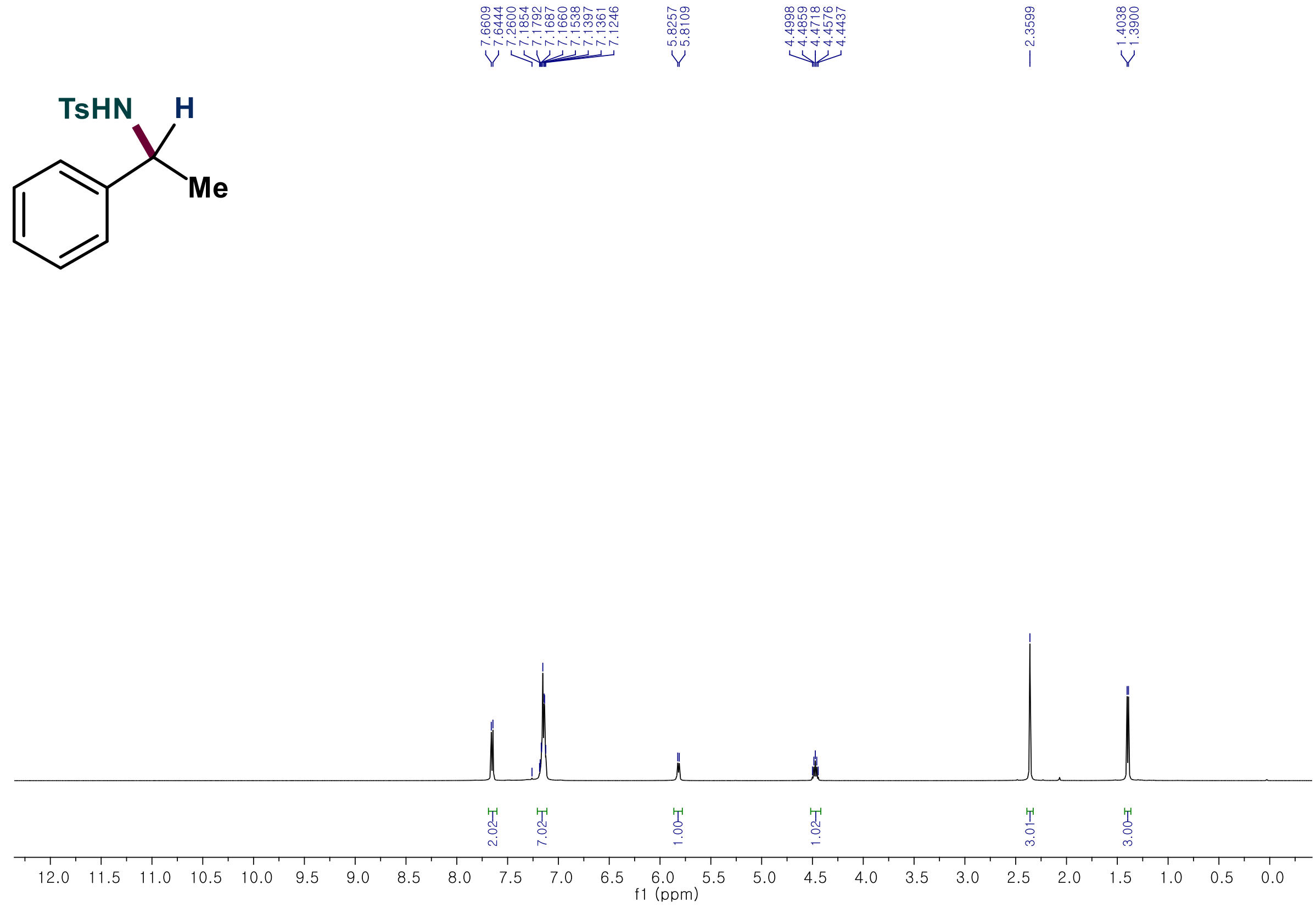

^{13}C NMR spectra (**28**)

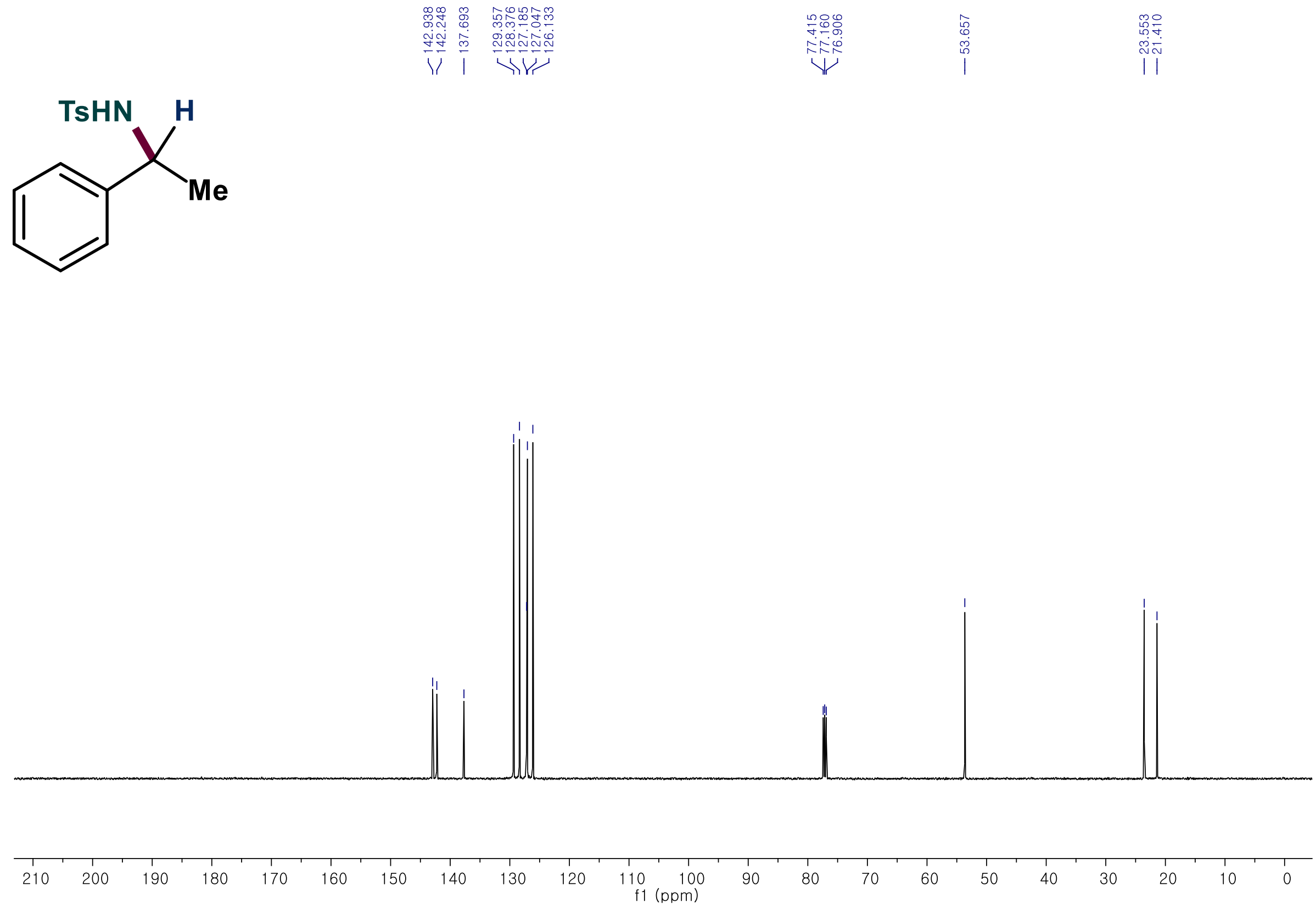

^{1}H NMR spectra (**29**)

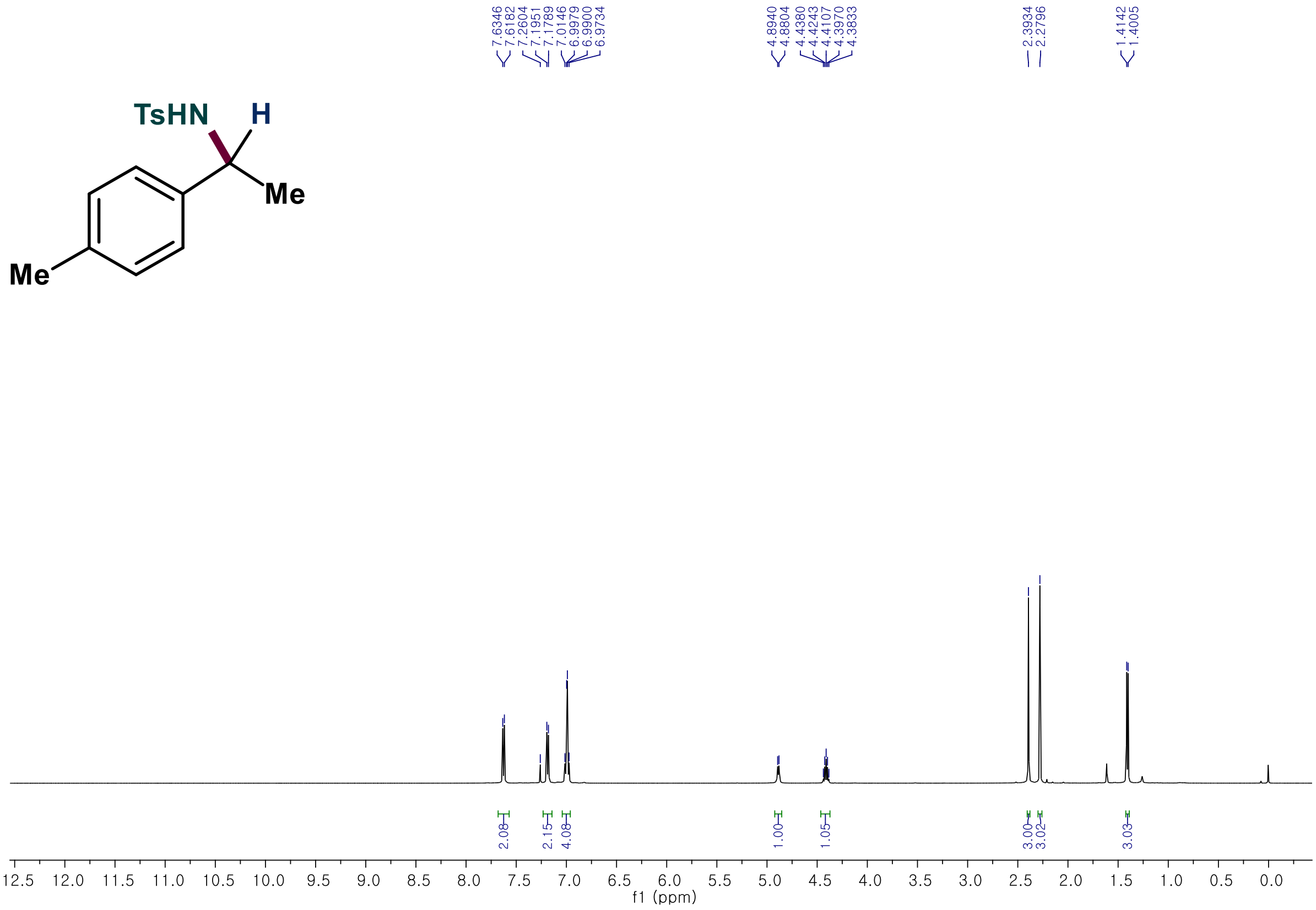

^{13}C NMR spectra (**29**)

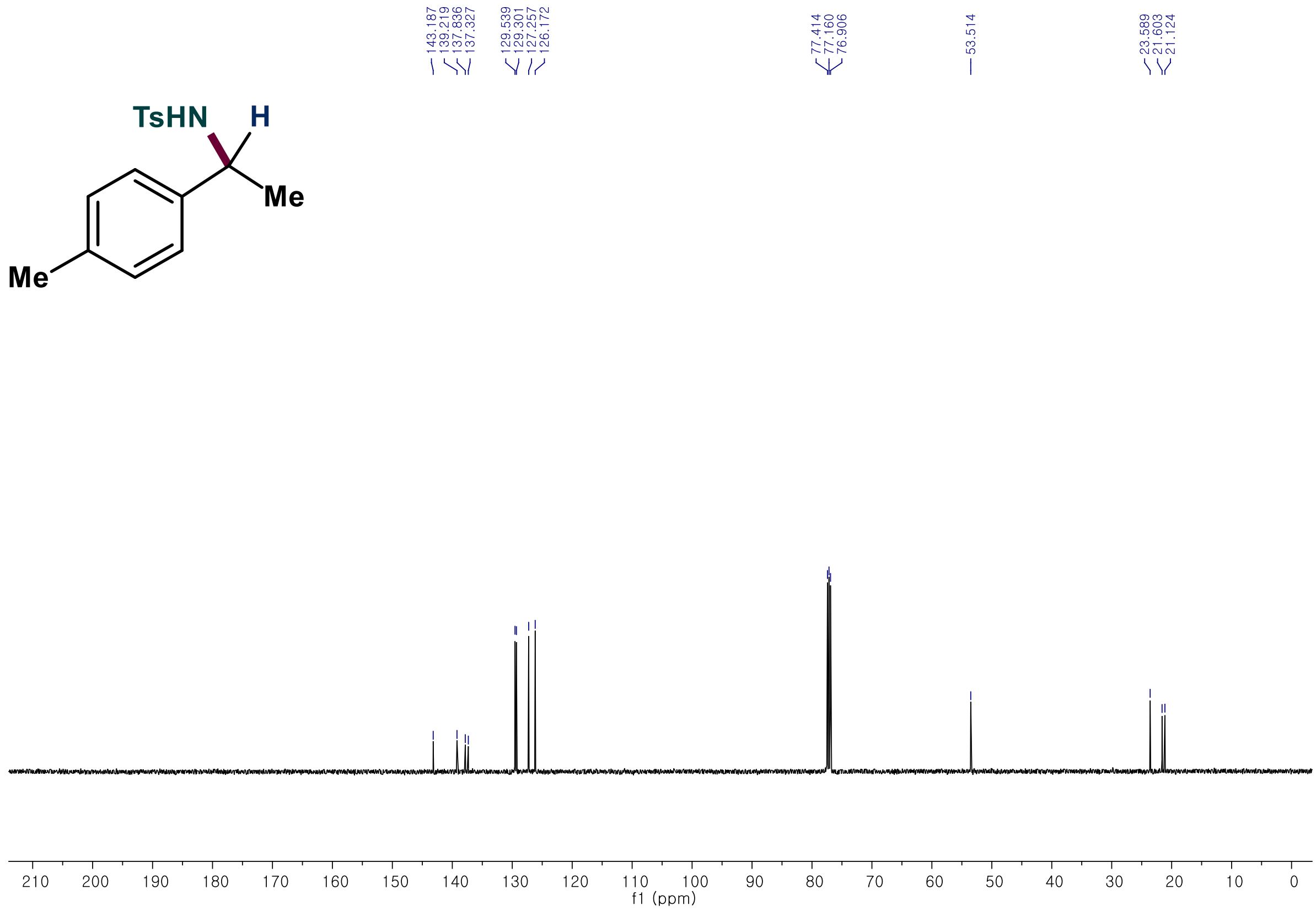

^{1}H NMR spectra (**30**)

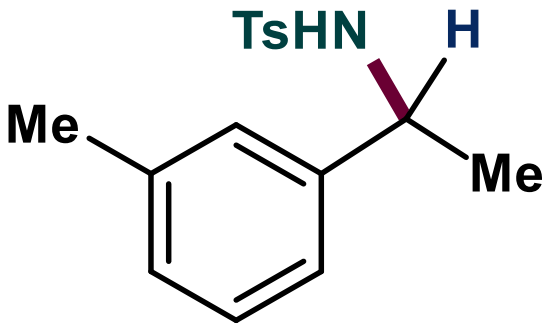

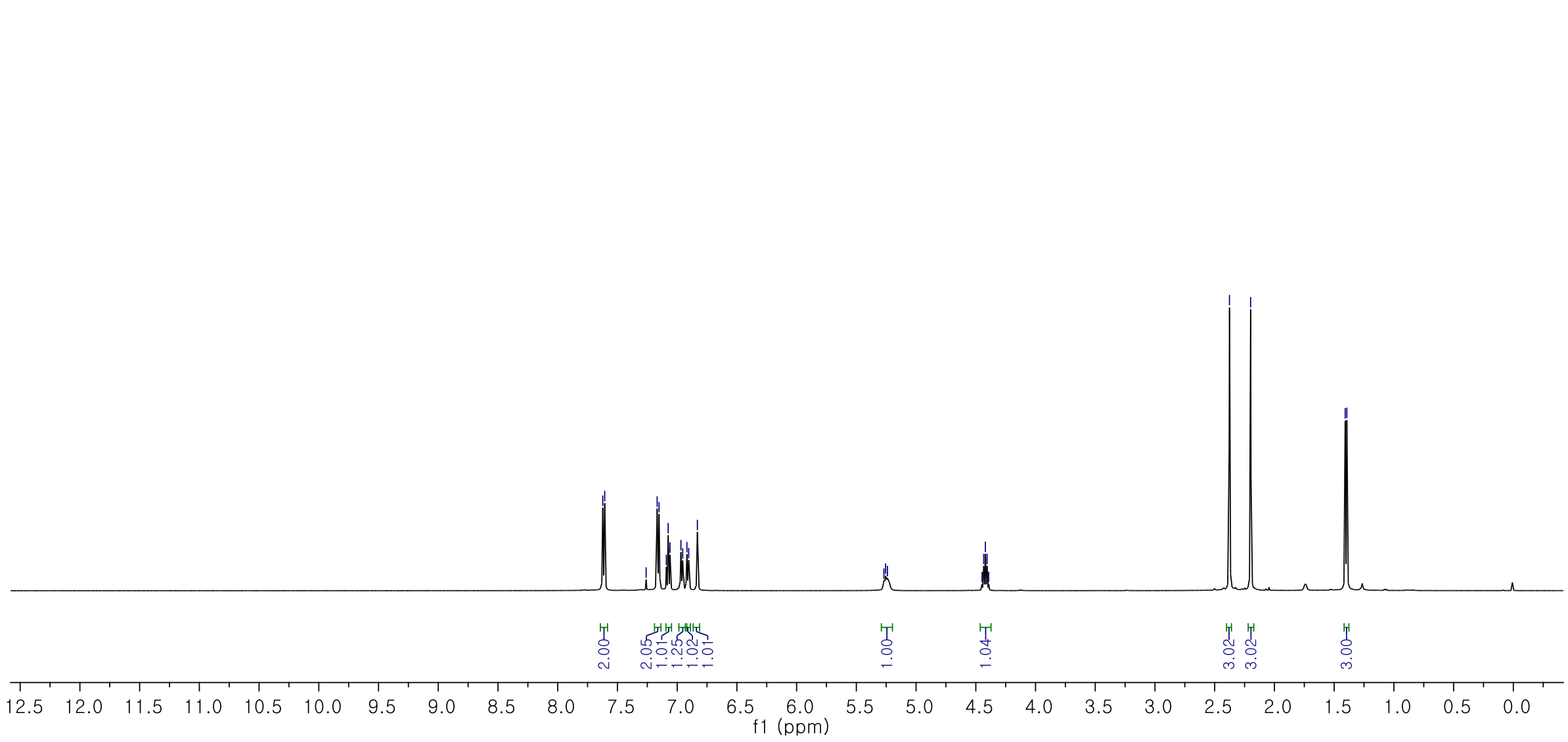

^{13}C NMR spectra (**30**)

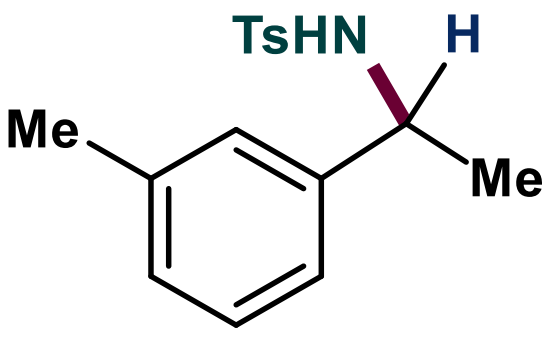

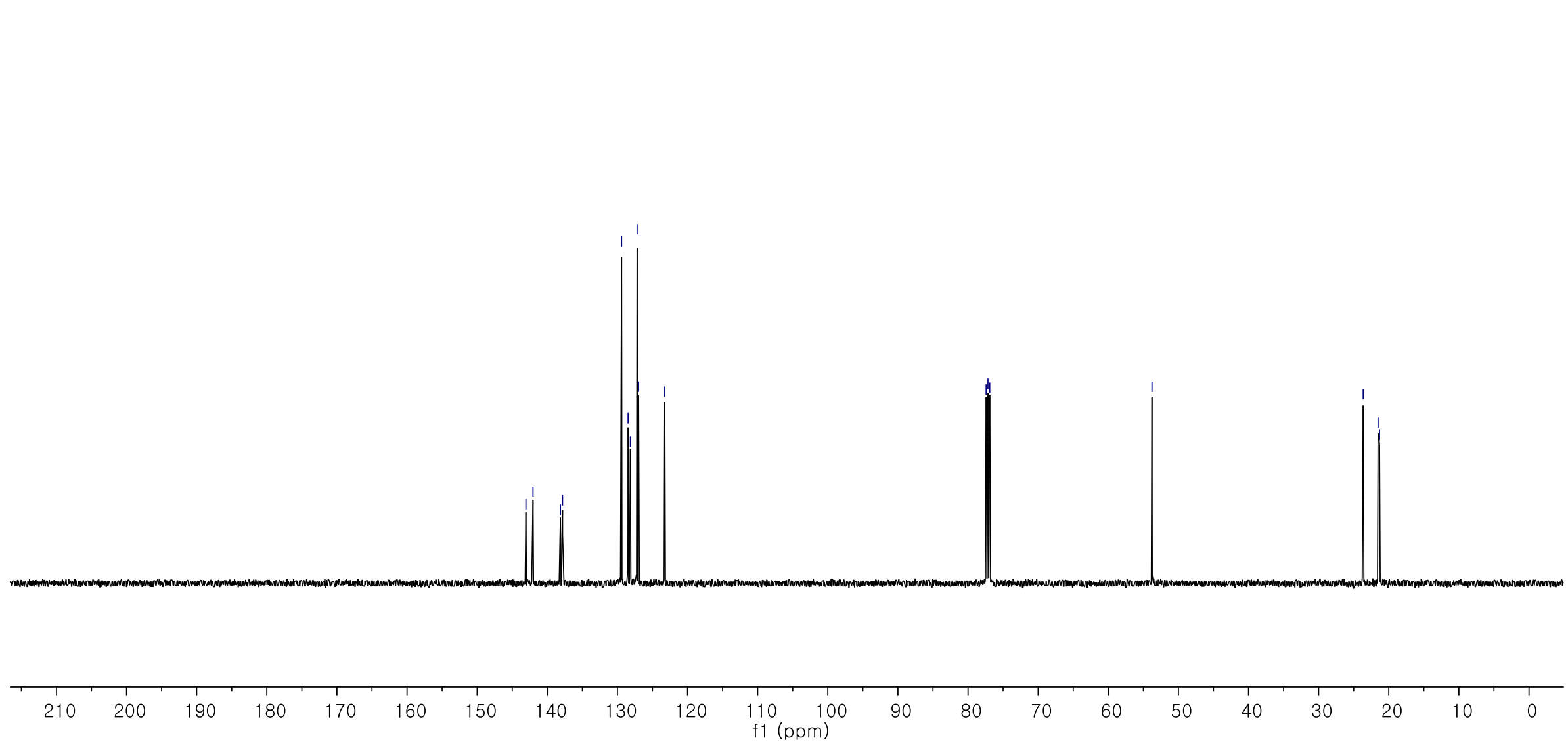

^{1}H NMR spectra (**31**)

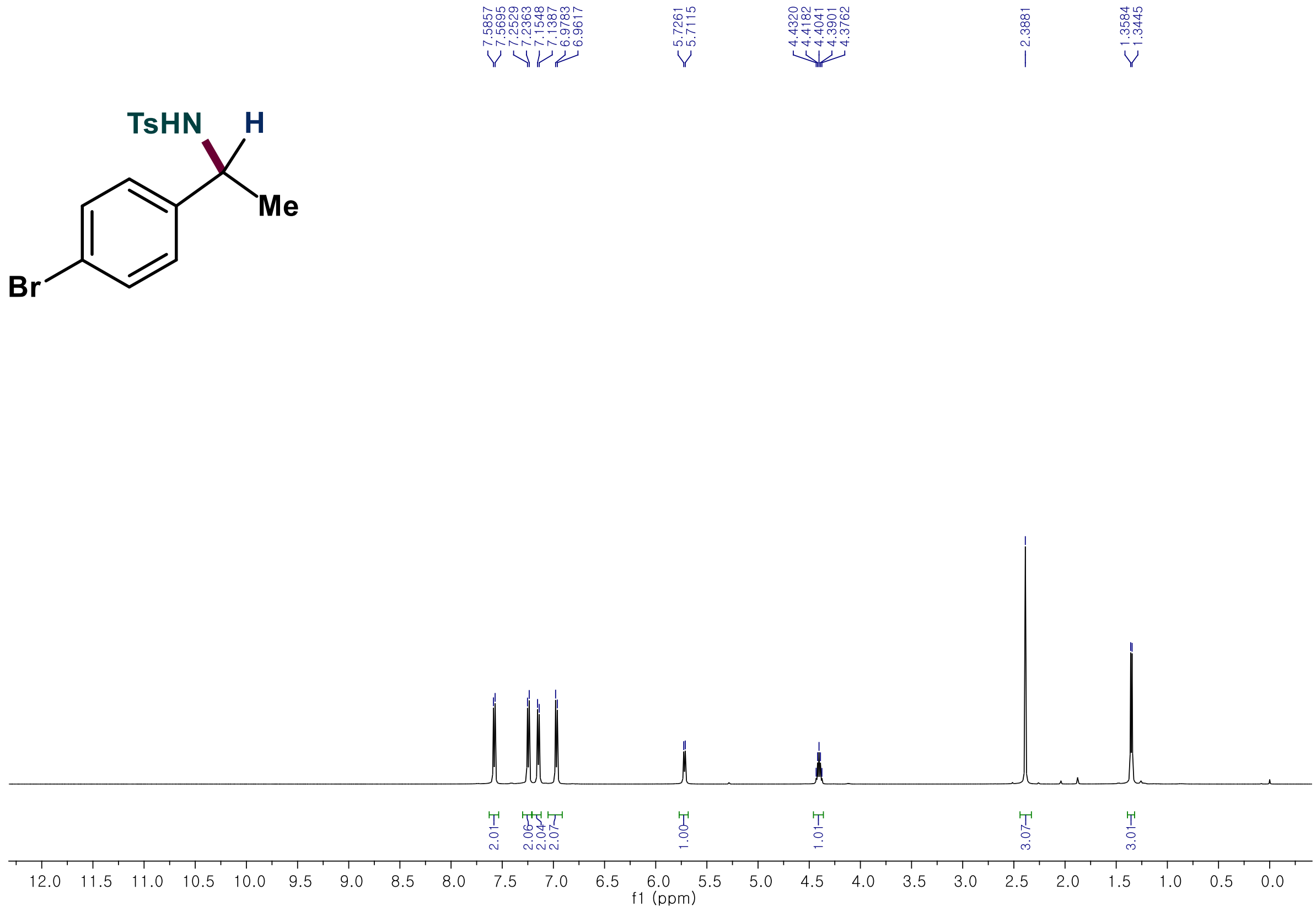

^{13}C NMR spectra (**31**)

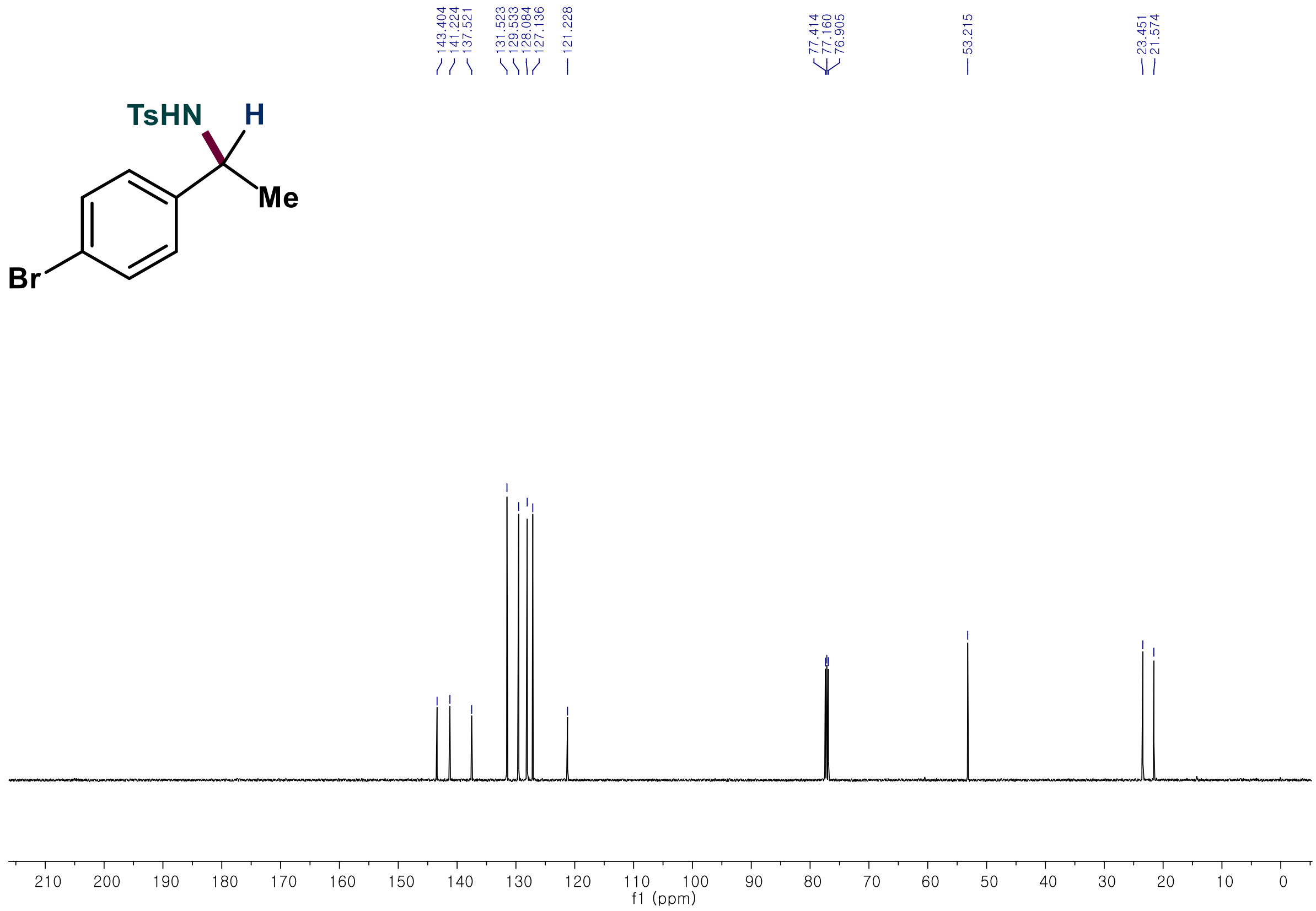

^{1}H NMR spectra (**32**)

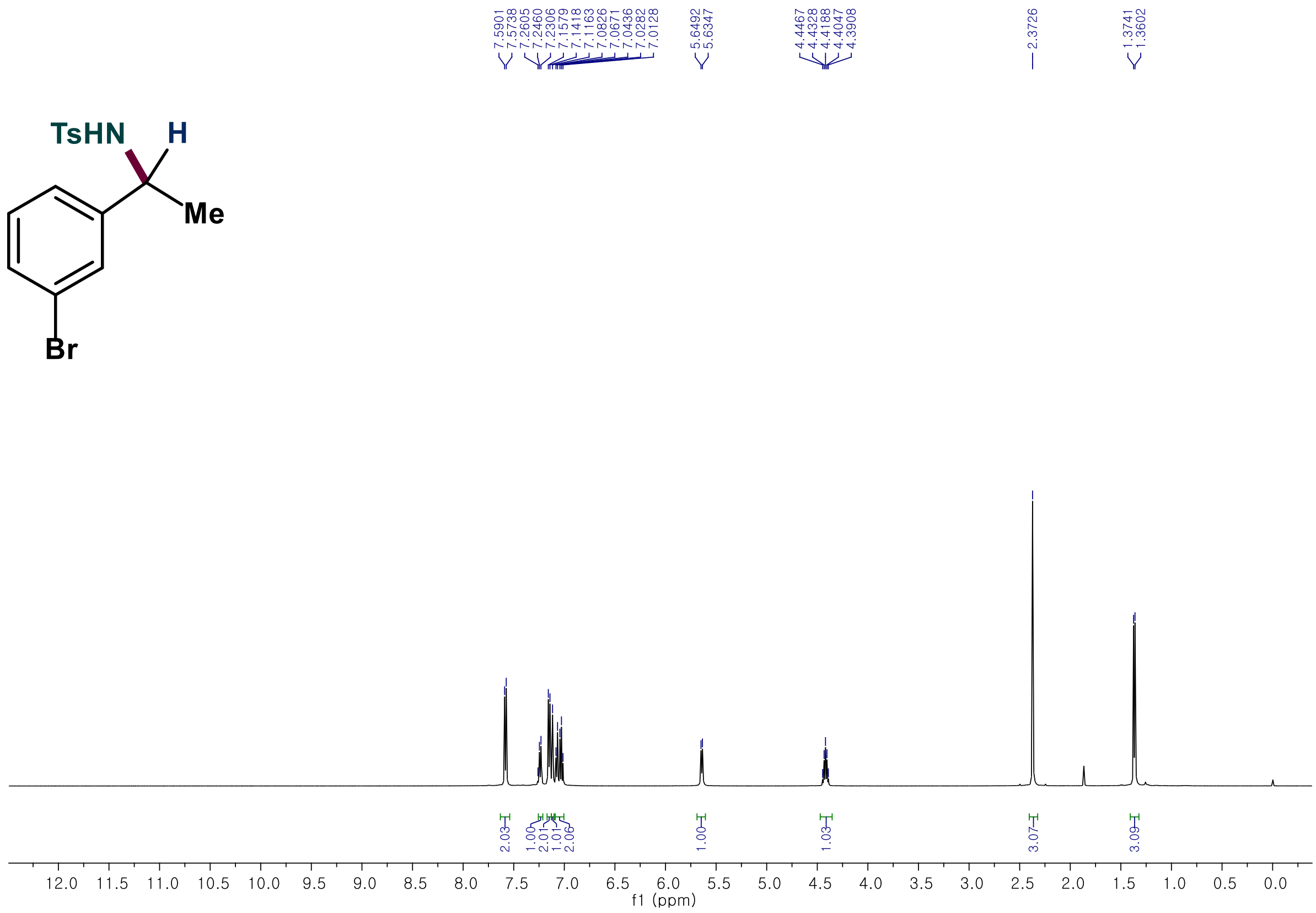

^{13}C NMR spectra (**32**)

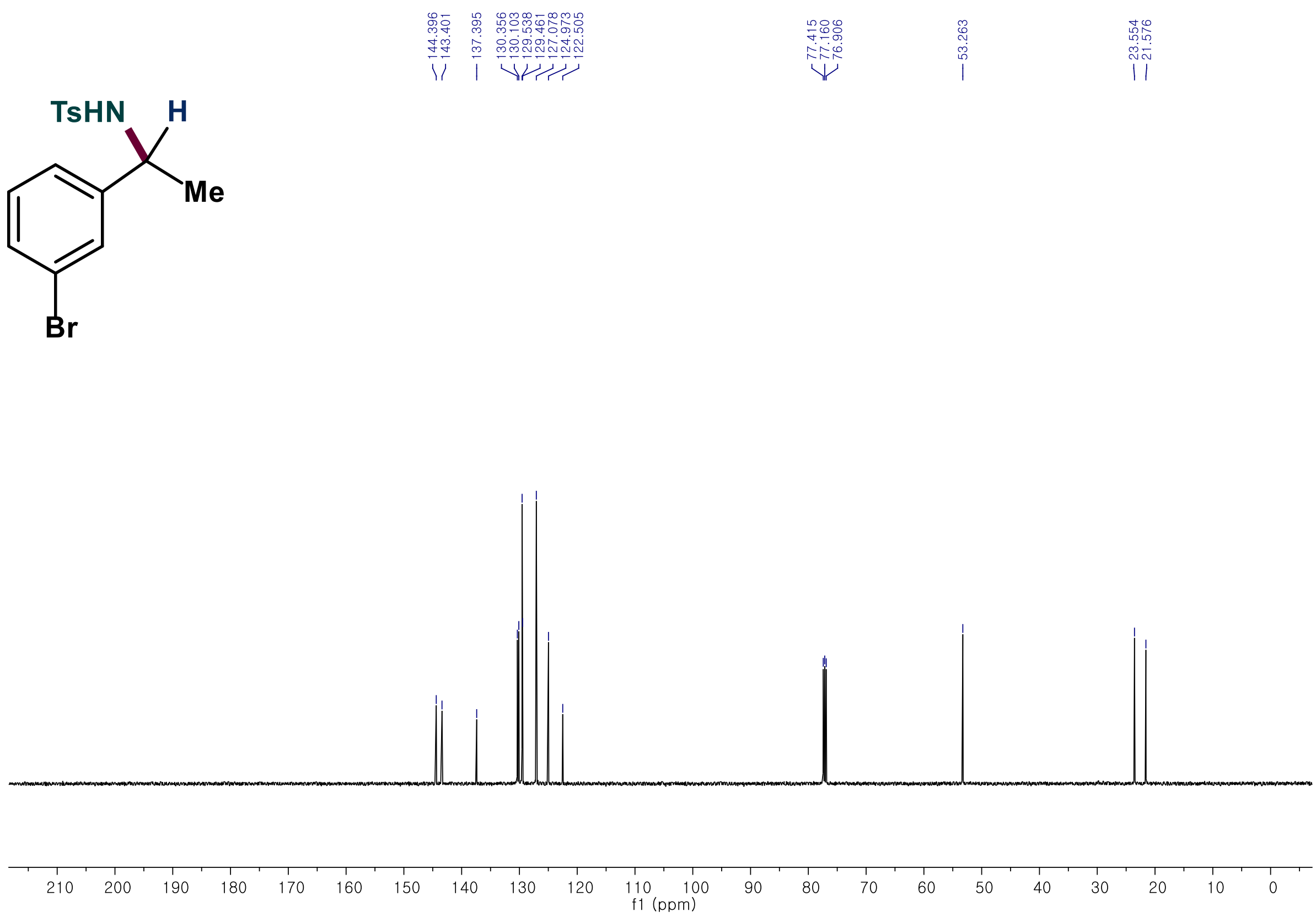

^{1}H NMR spectra (**33**)

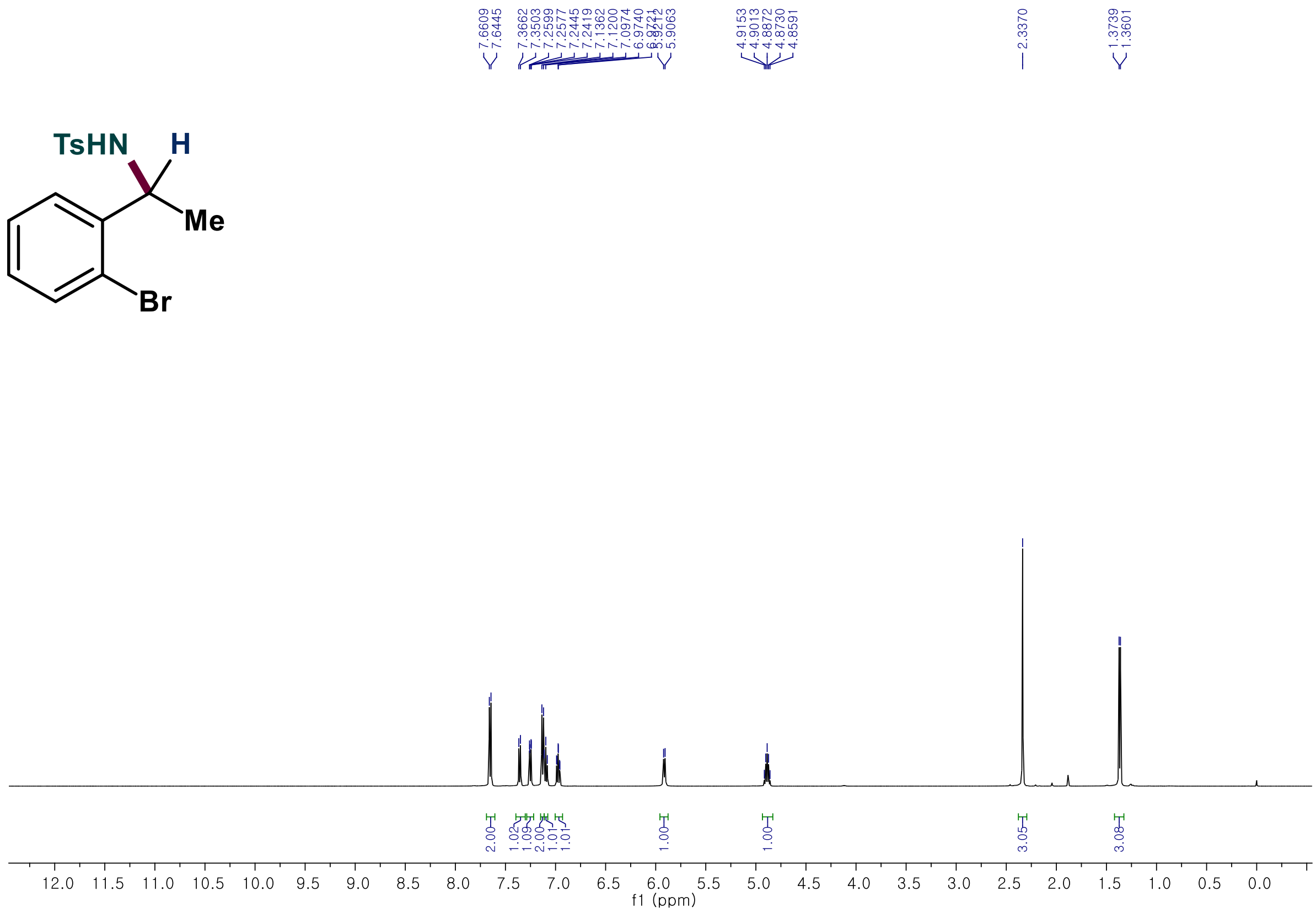

^{13}C NMR spectra (**33**)

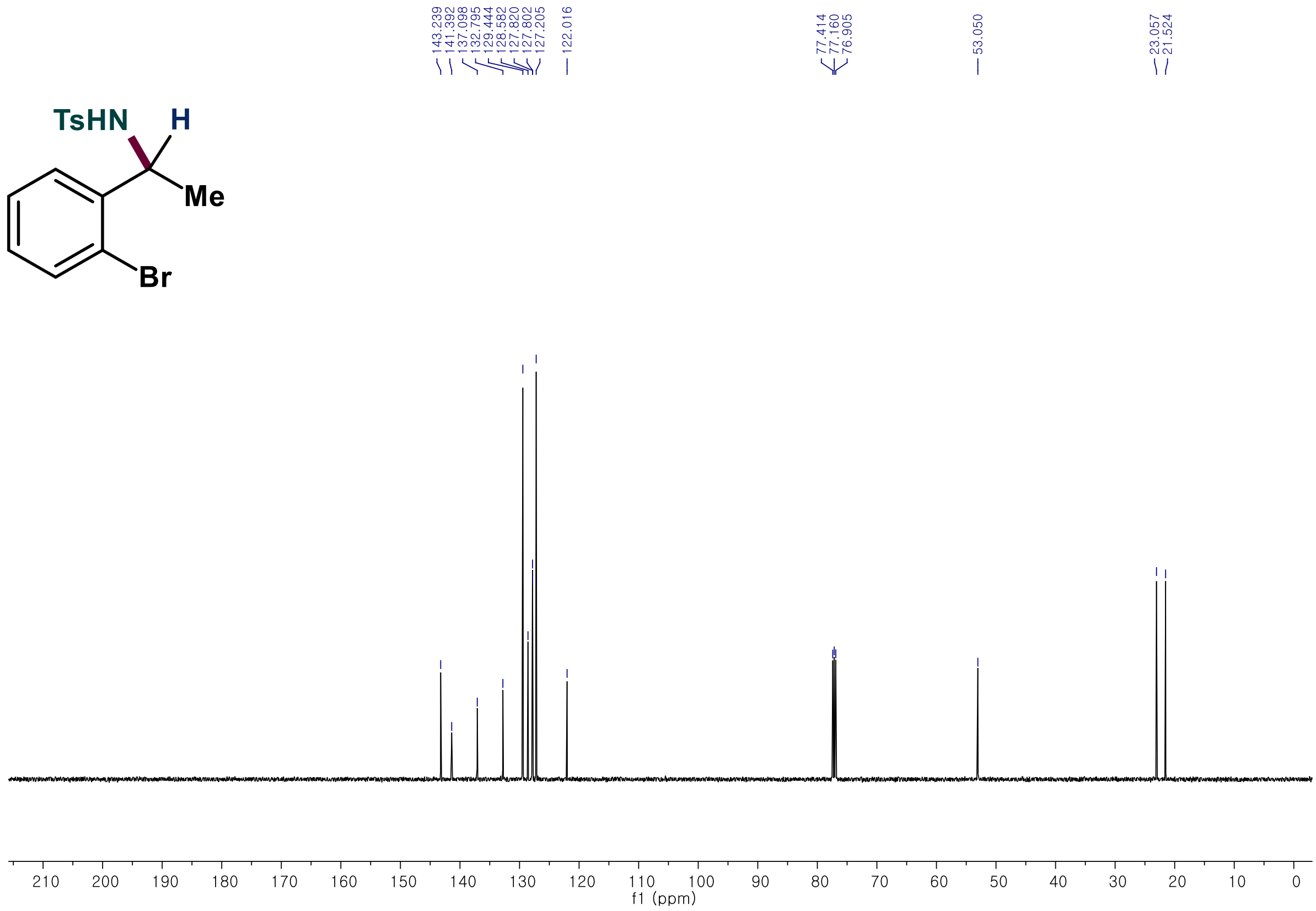

^1H NMR spectra (**34**)

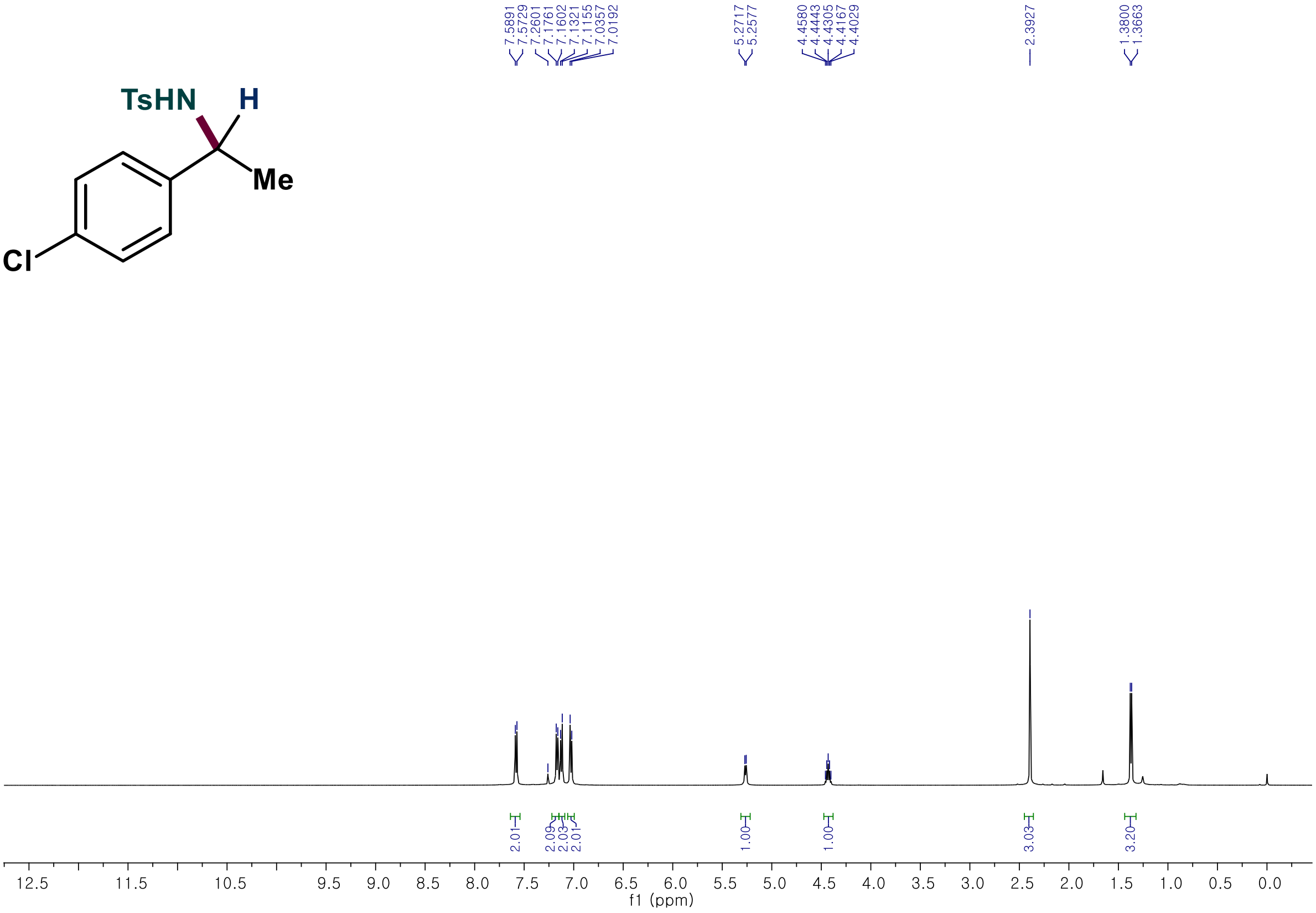

^{13}C NMR spectra (**34**)

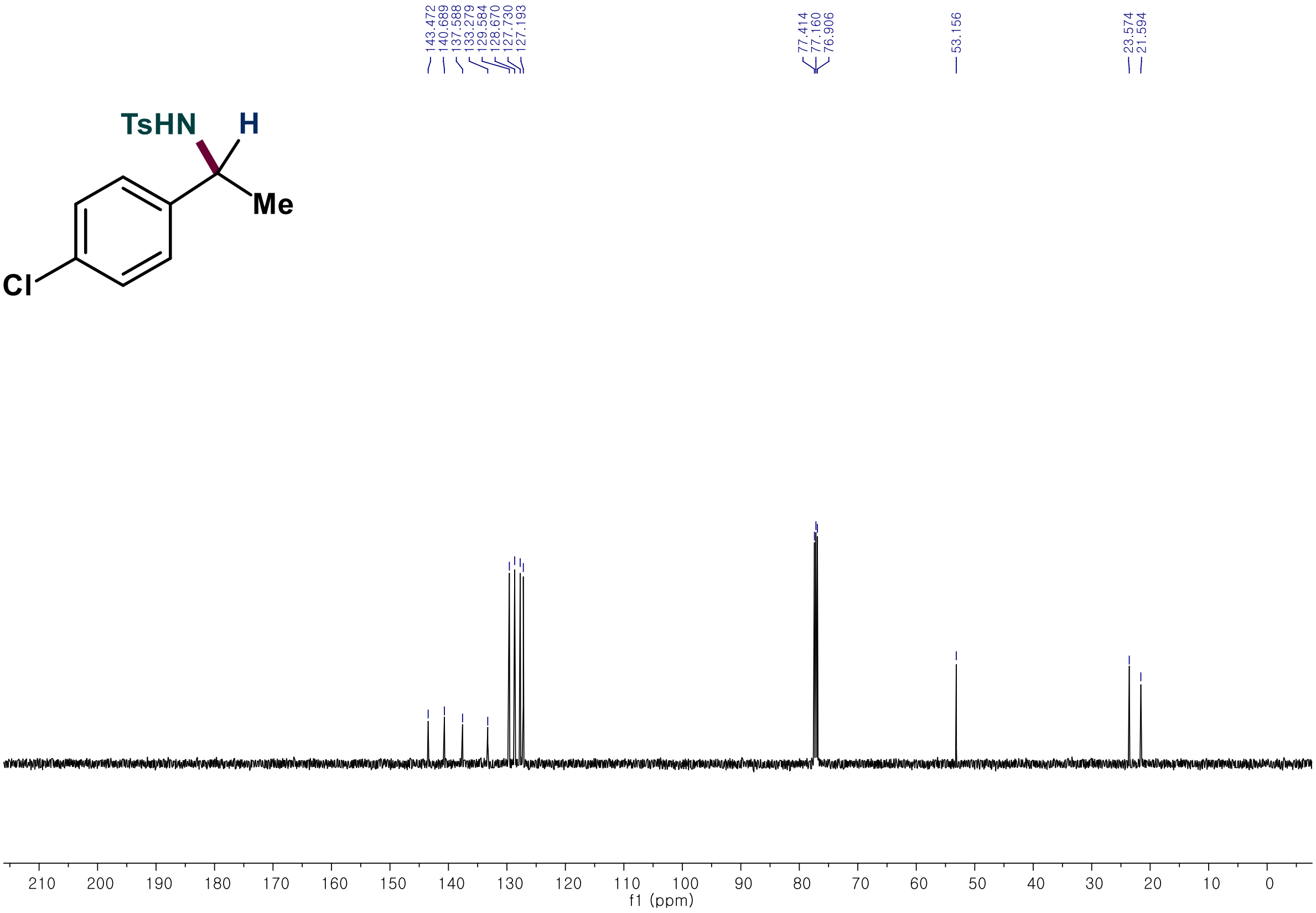

^{1}H NMR spectra (**35**)

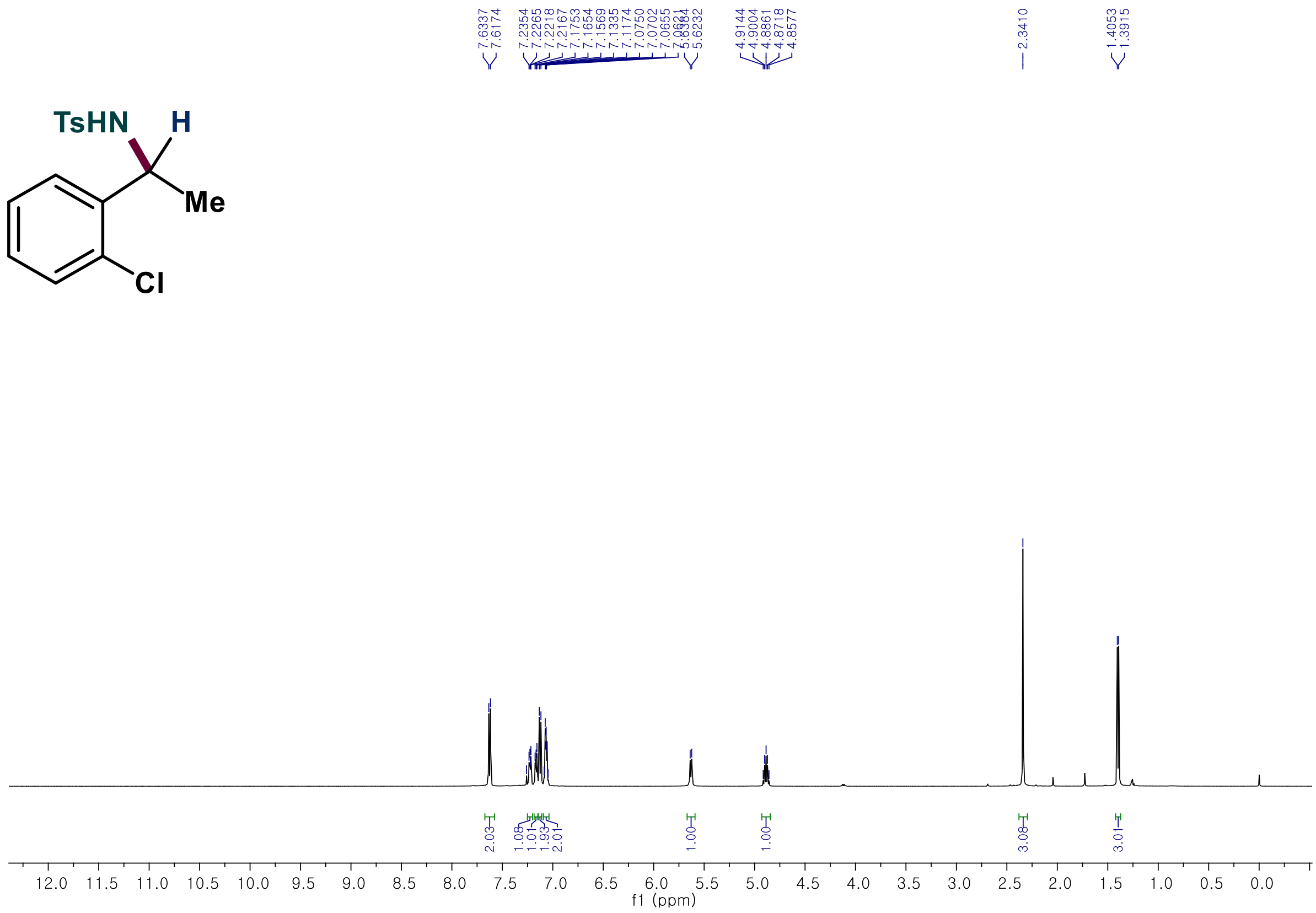

^{13}C NMR spectra (**35**)

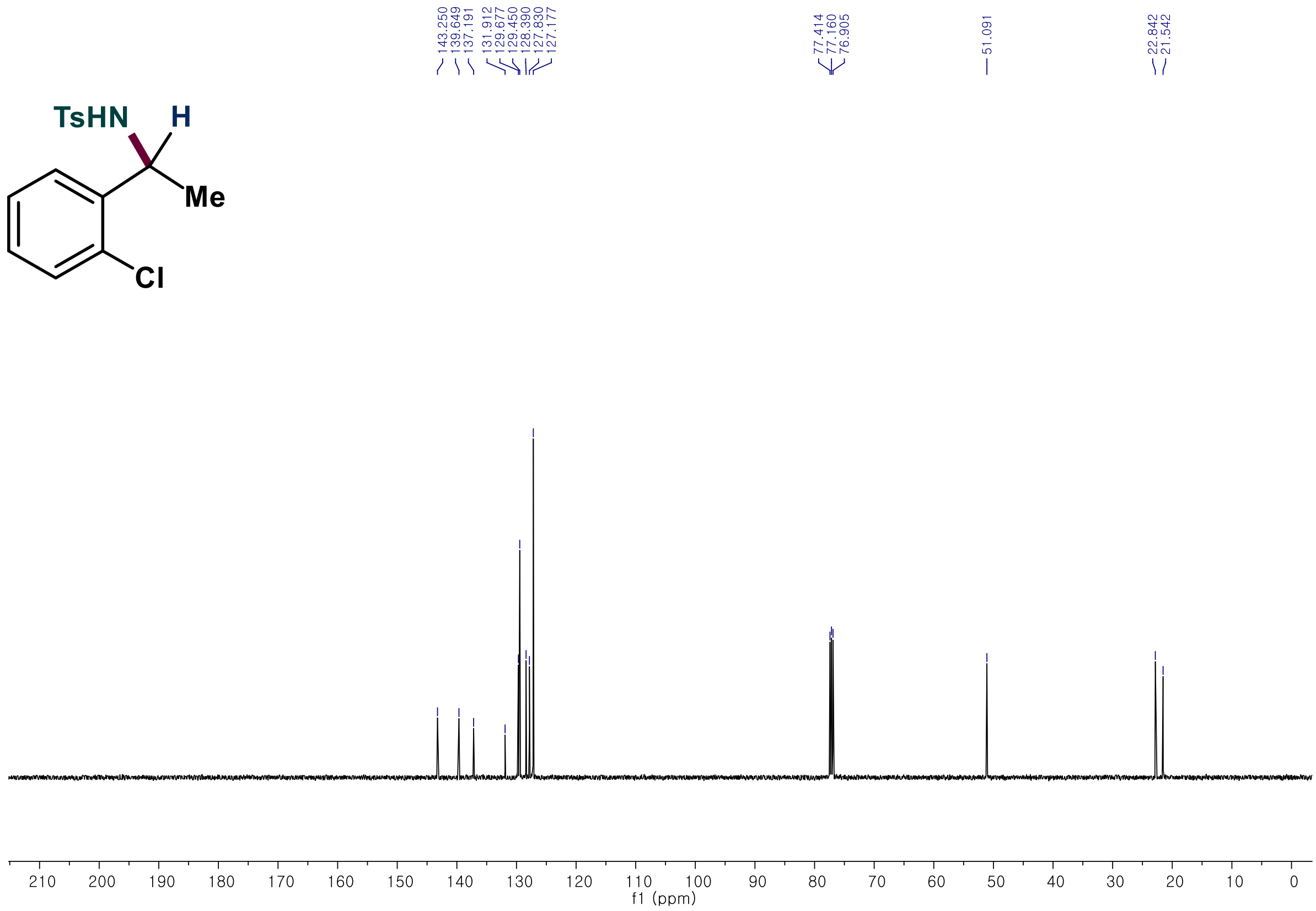

^{1}H NMR spectra (**36**)

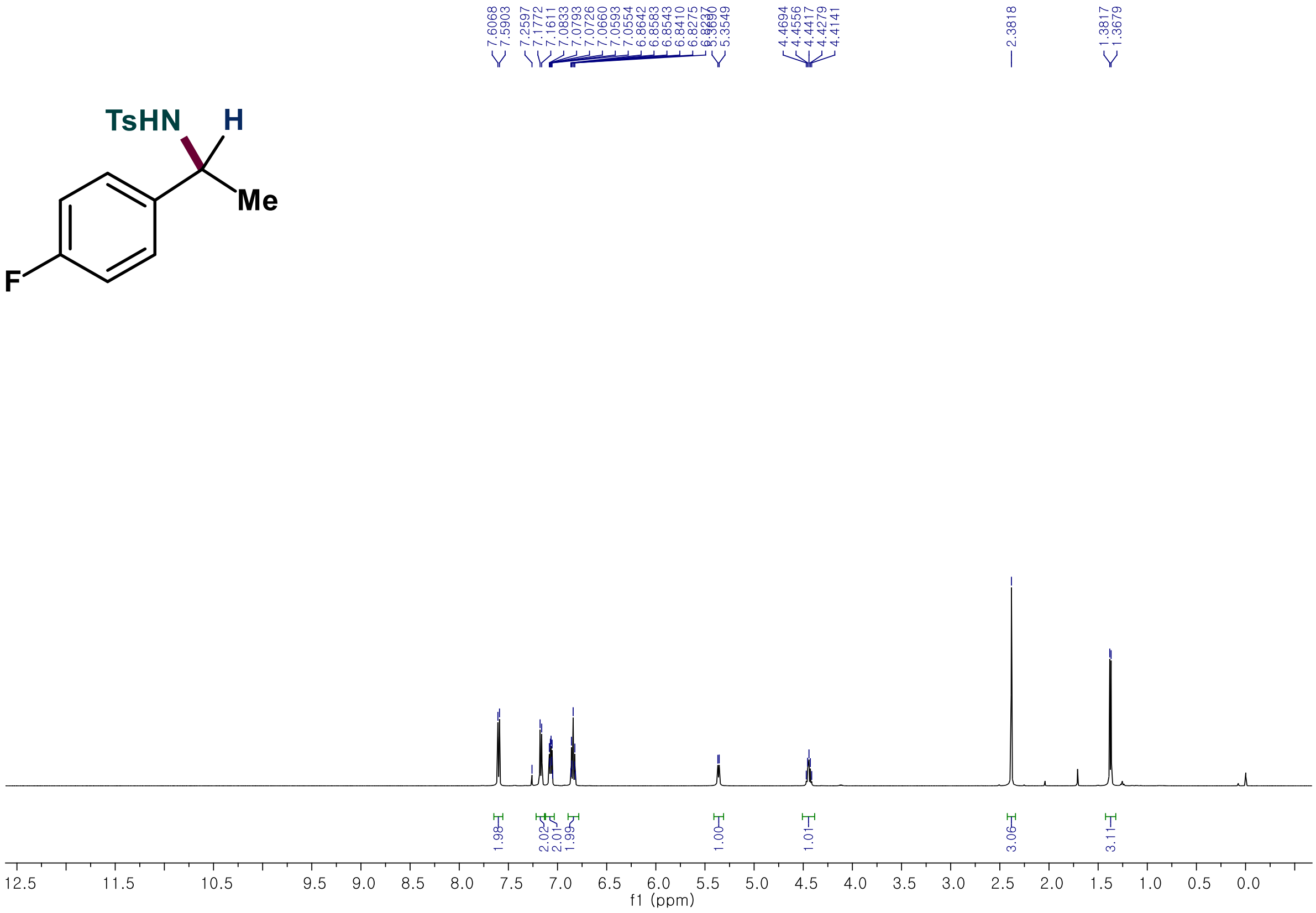

^{13}C NMR spectra (**36**)

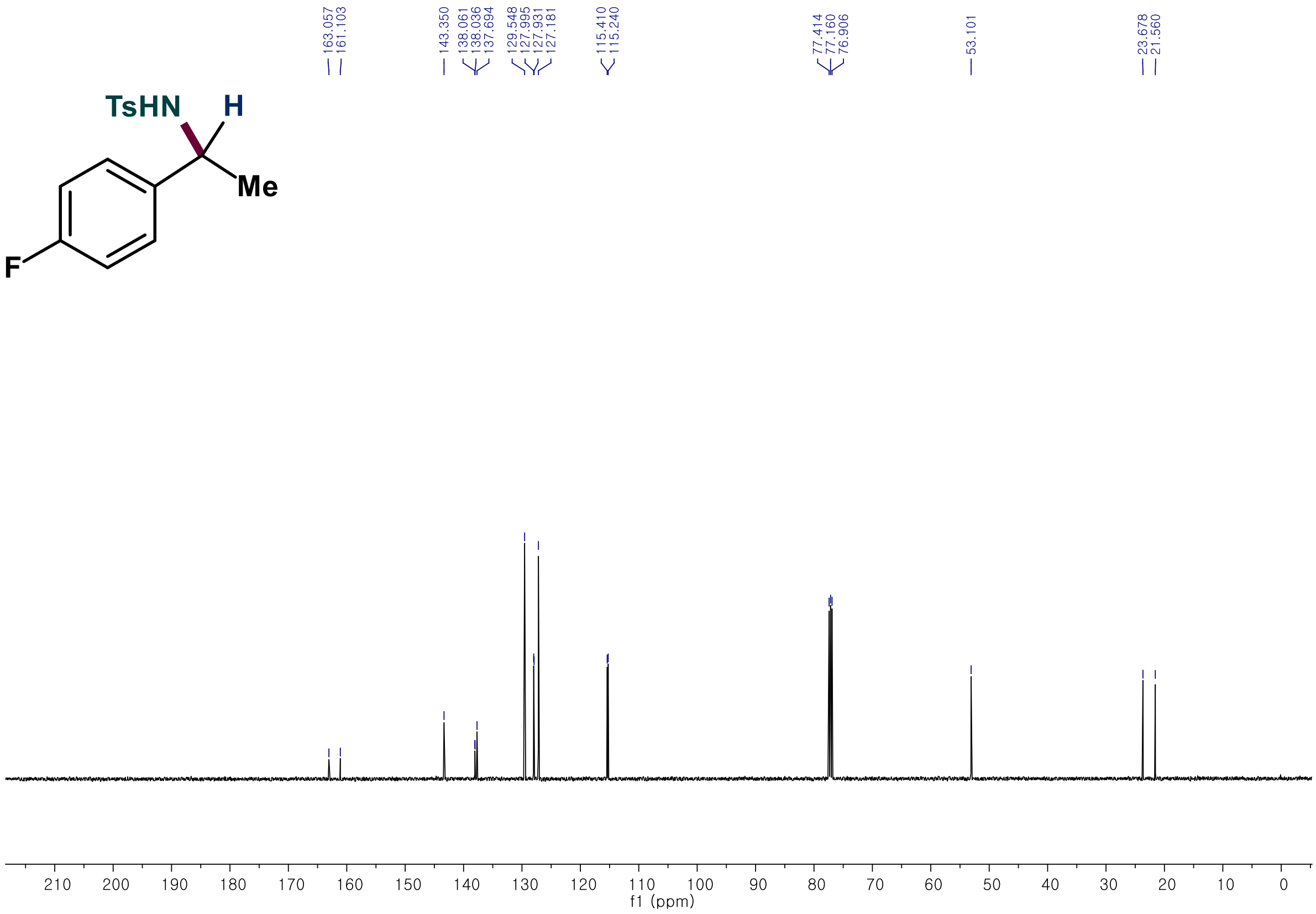

^{19}F NMR spectra (**36**)

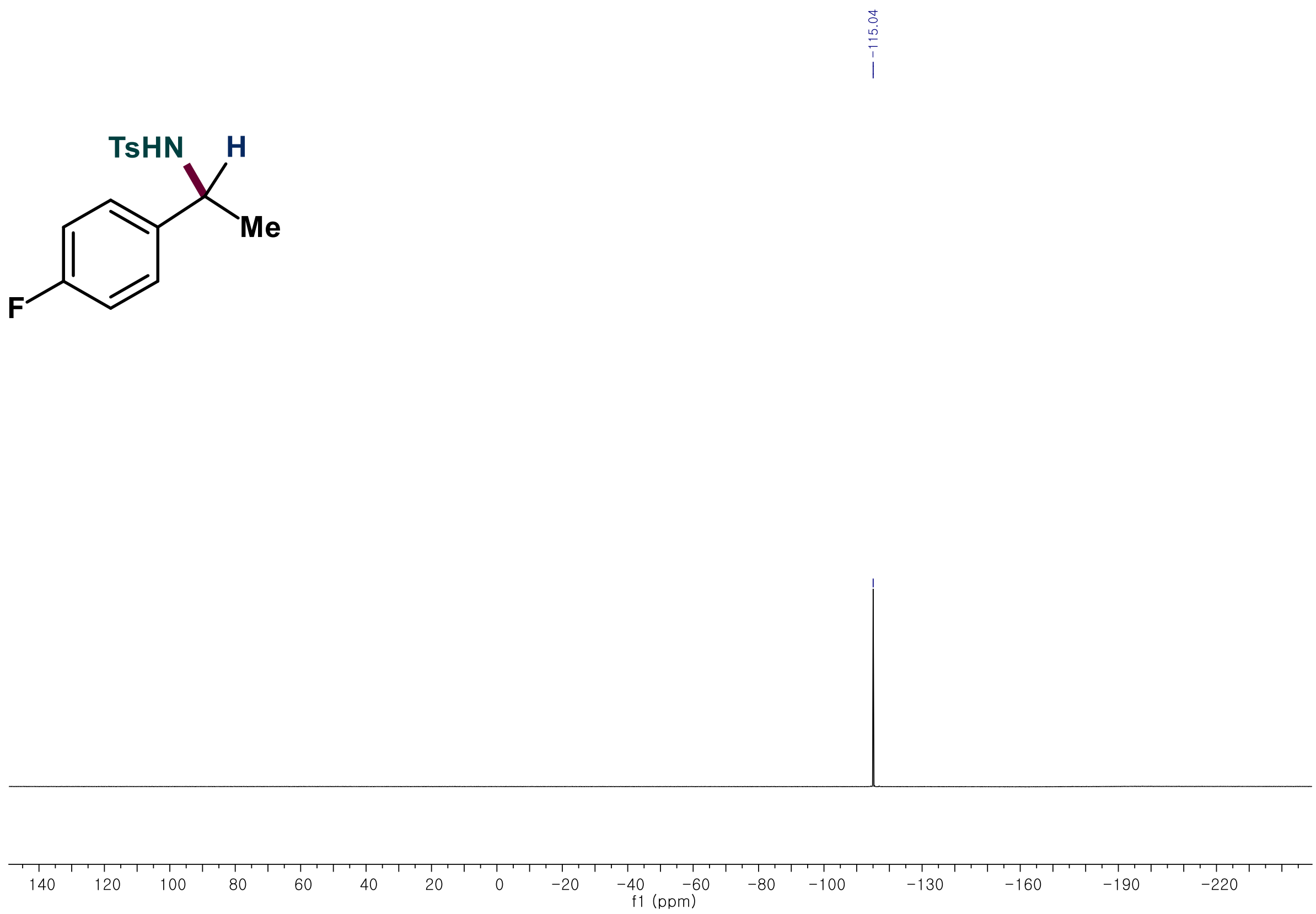

^{1}H NMR spectra (**37**)

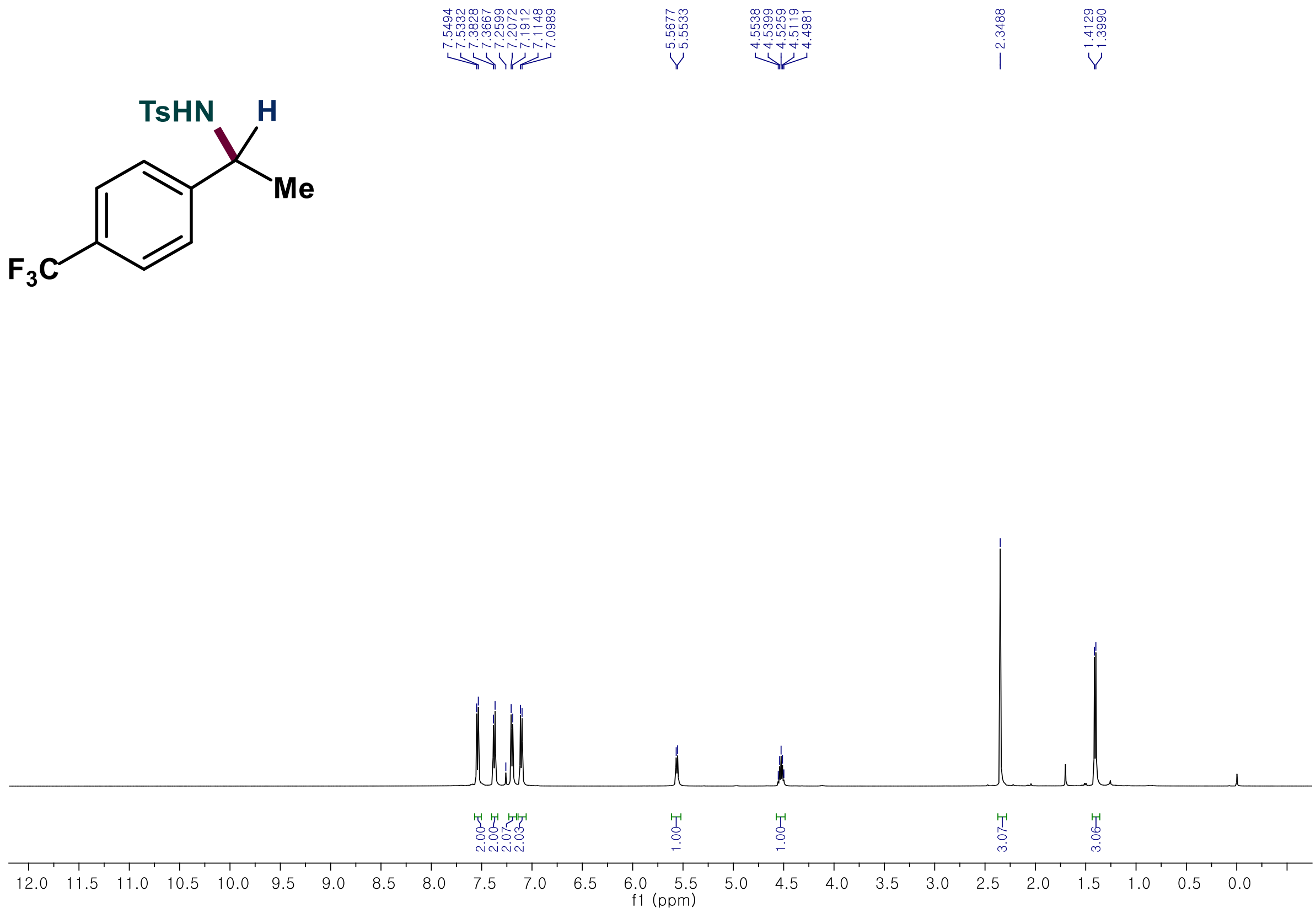

^{13}C NMR spectra (**37**)

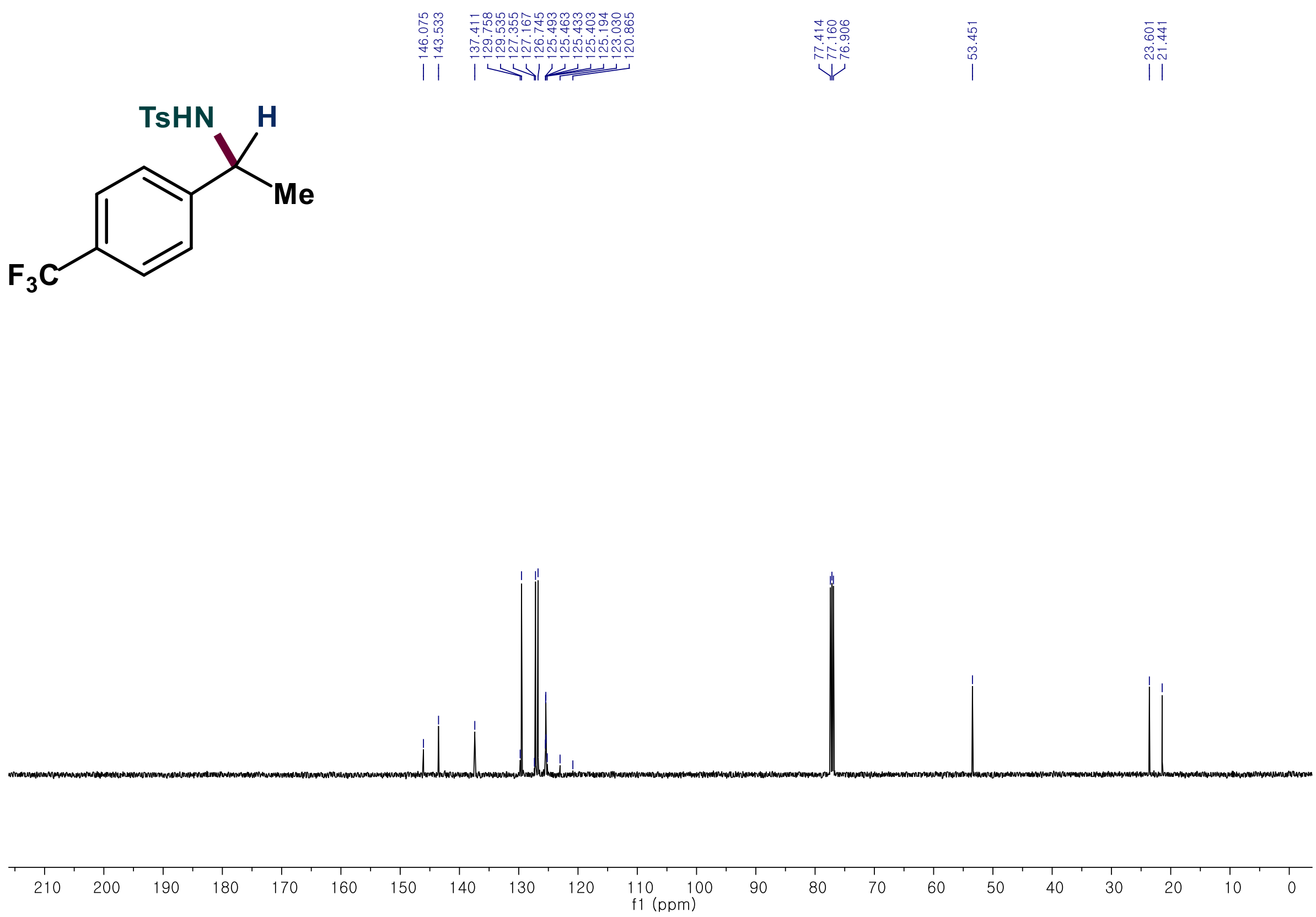

^{19}F NMR spectra (**37**)

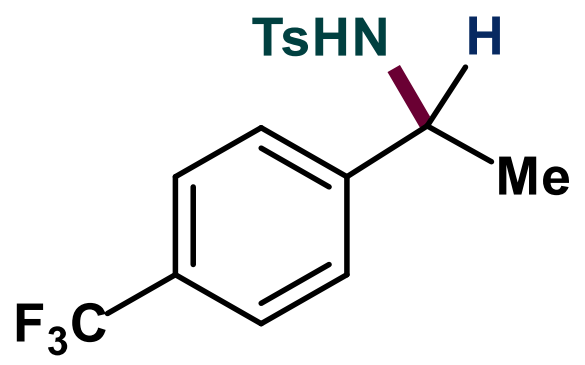

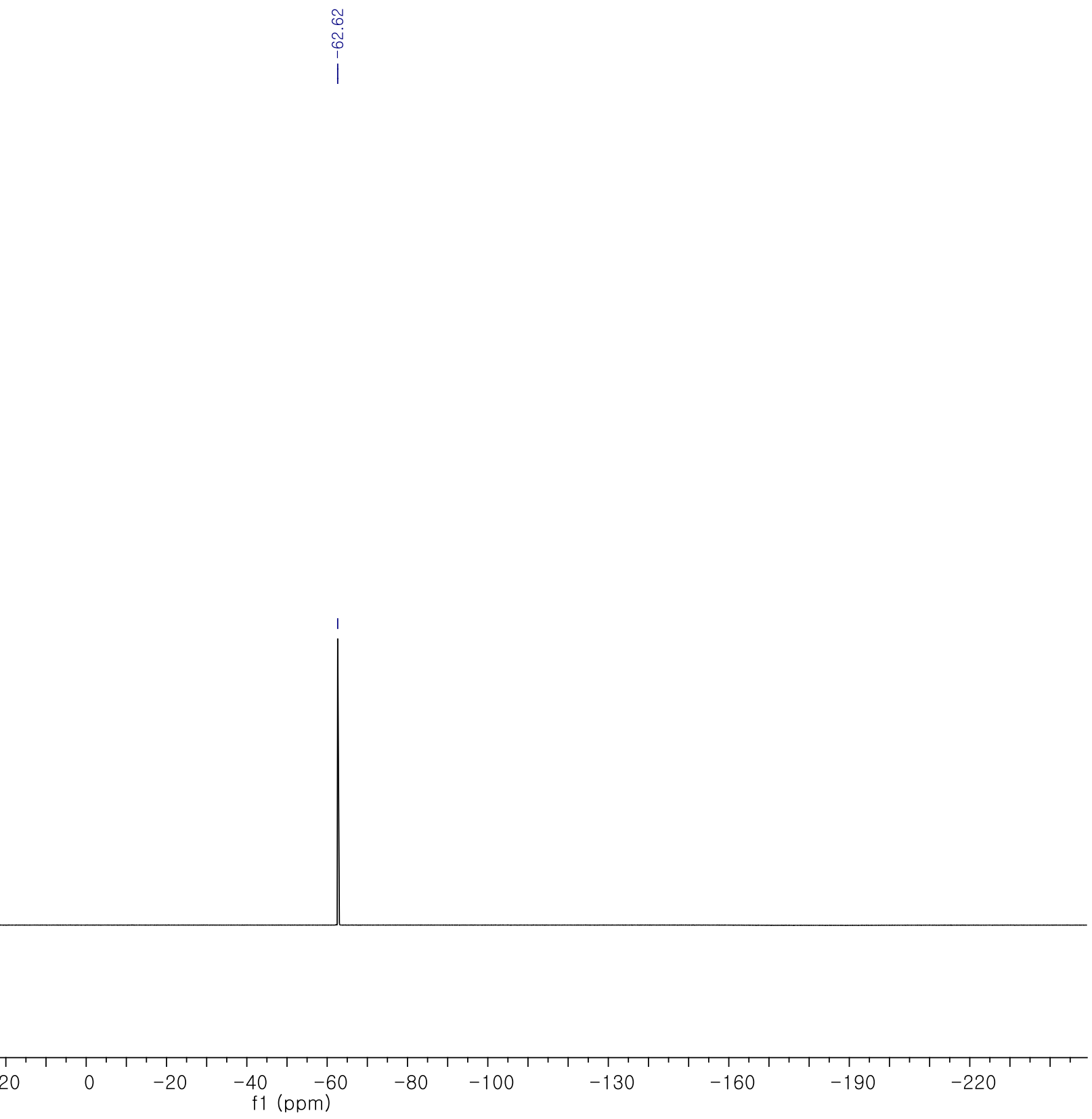

^{1}H NMR spectra (**38**)

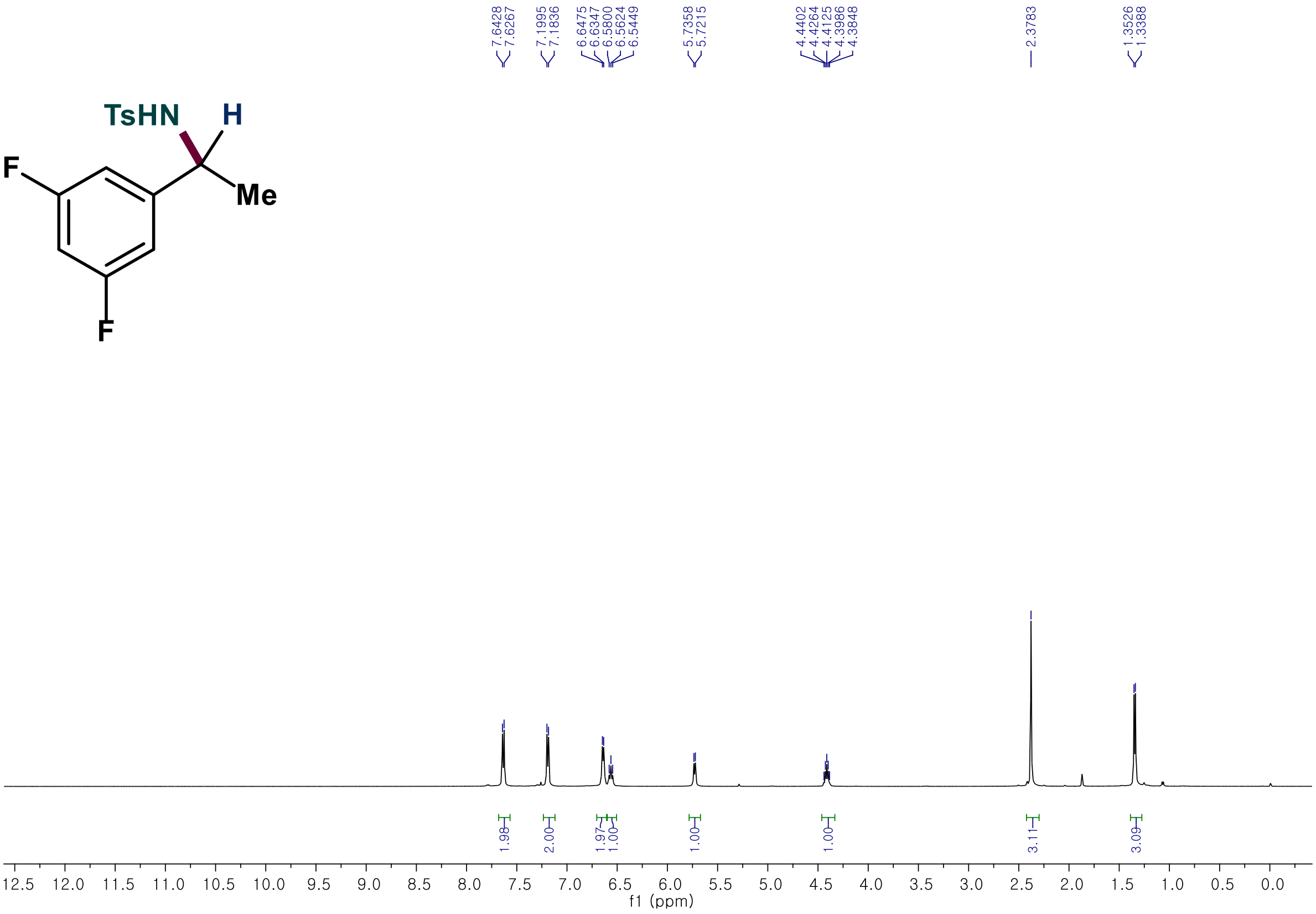

^{13}C NMR spectra (**38**)

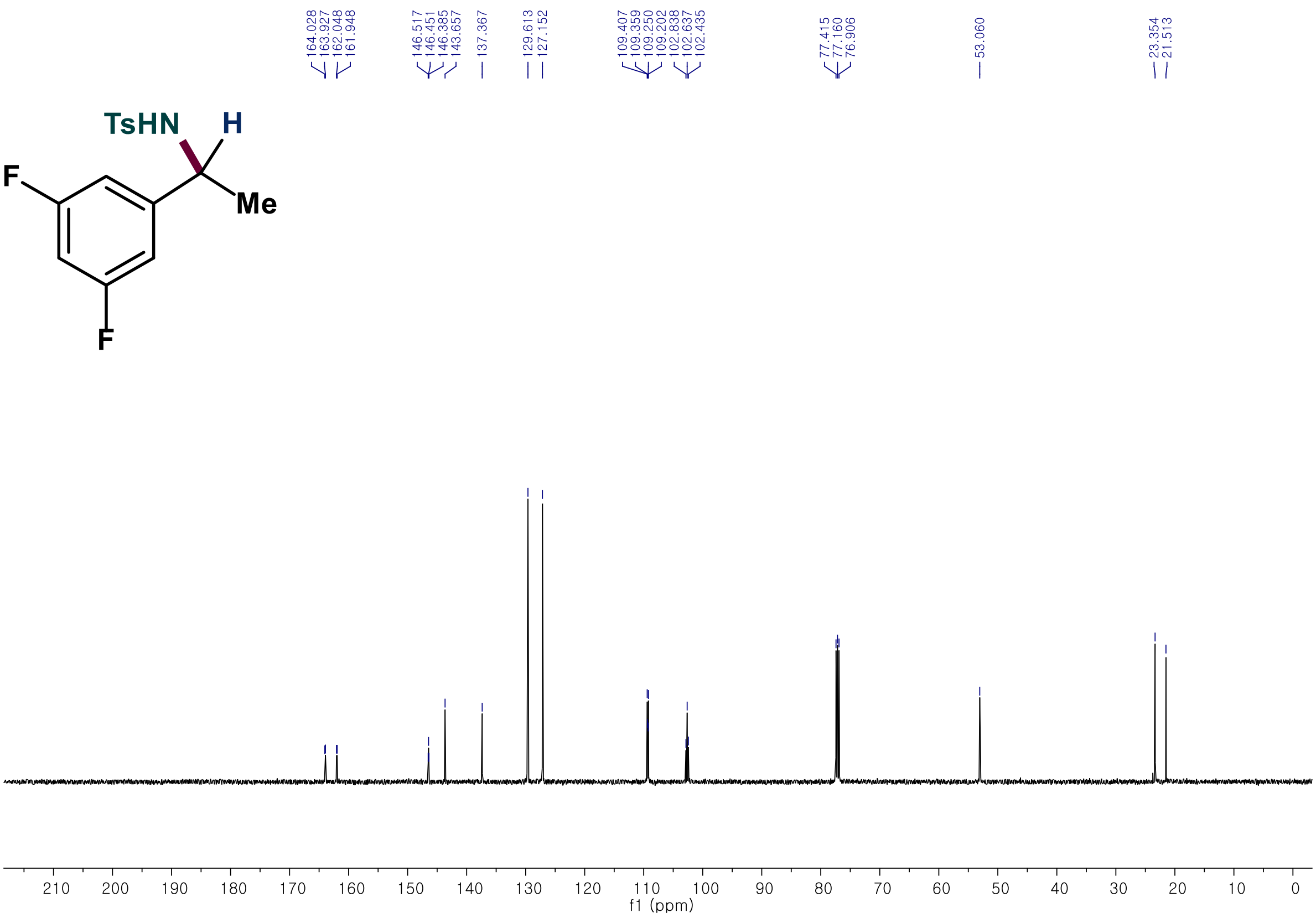

^{19}F NMR spectra (**38**)

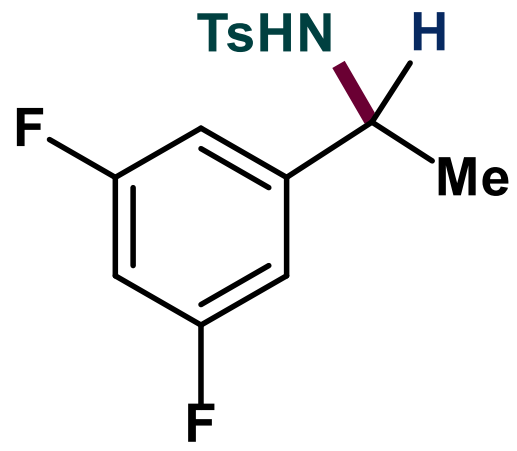

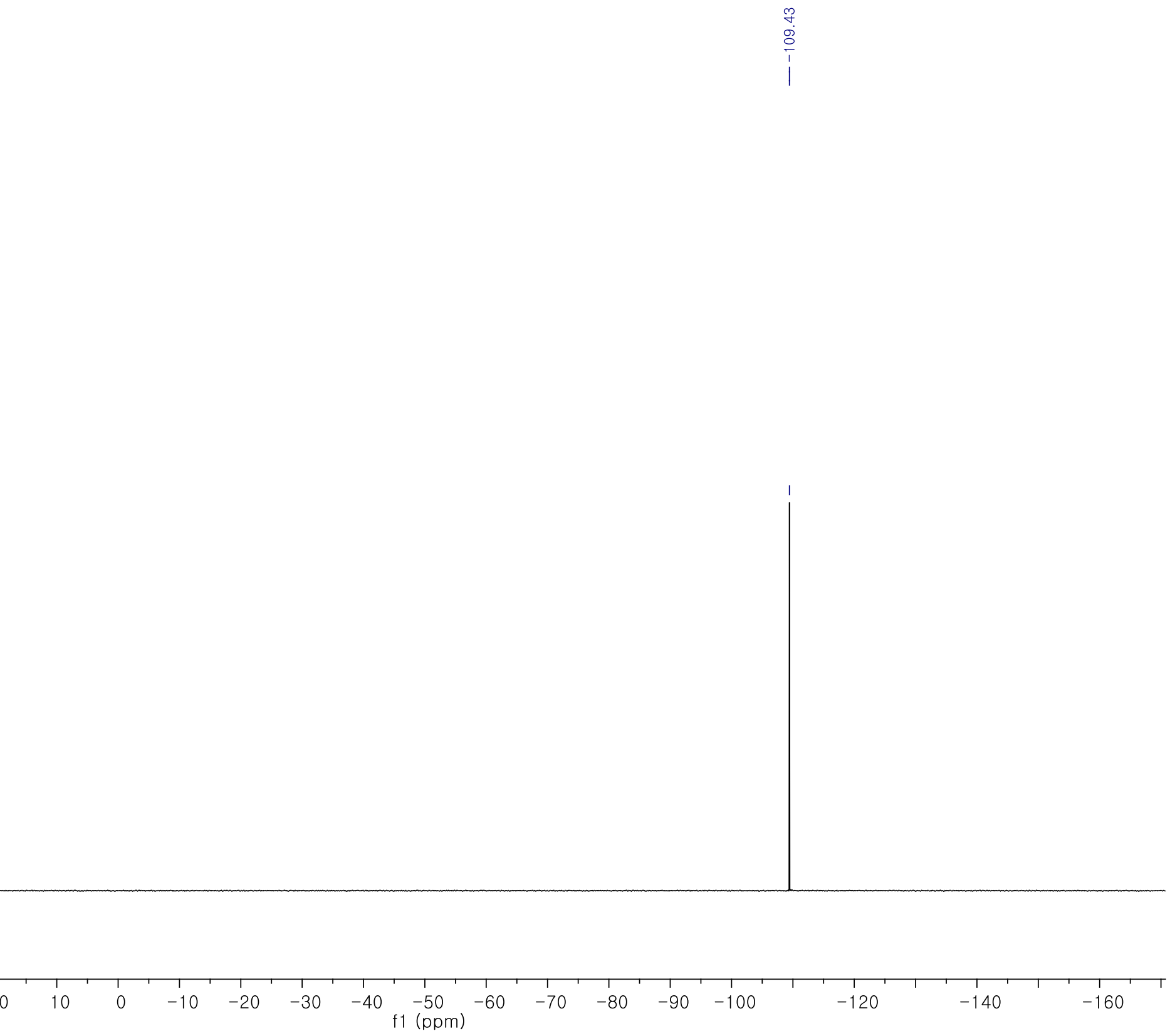

^{1}H NMR spectra (**39**)

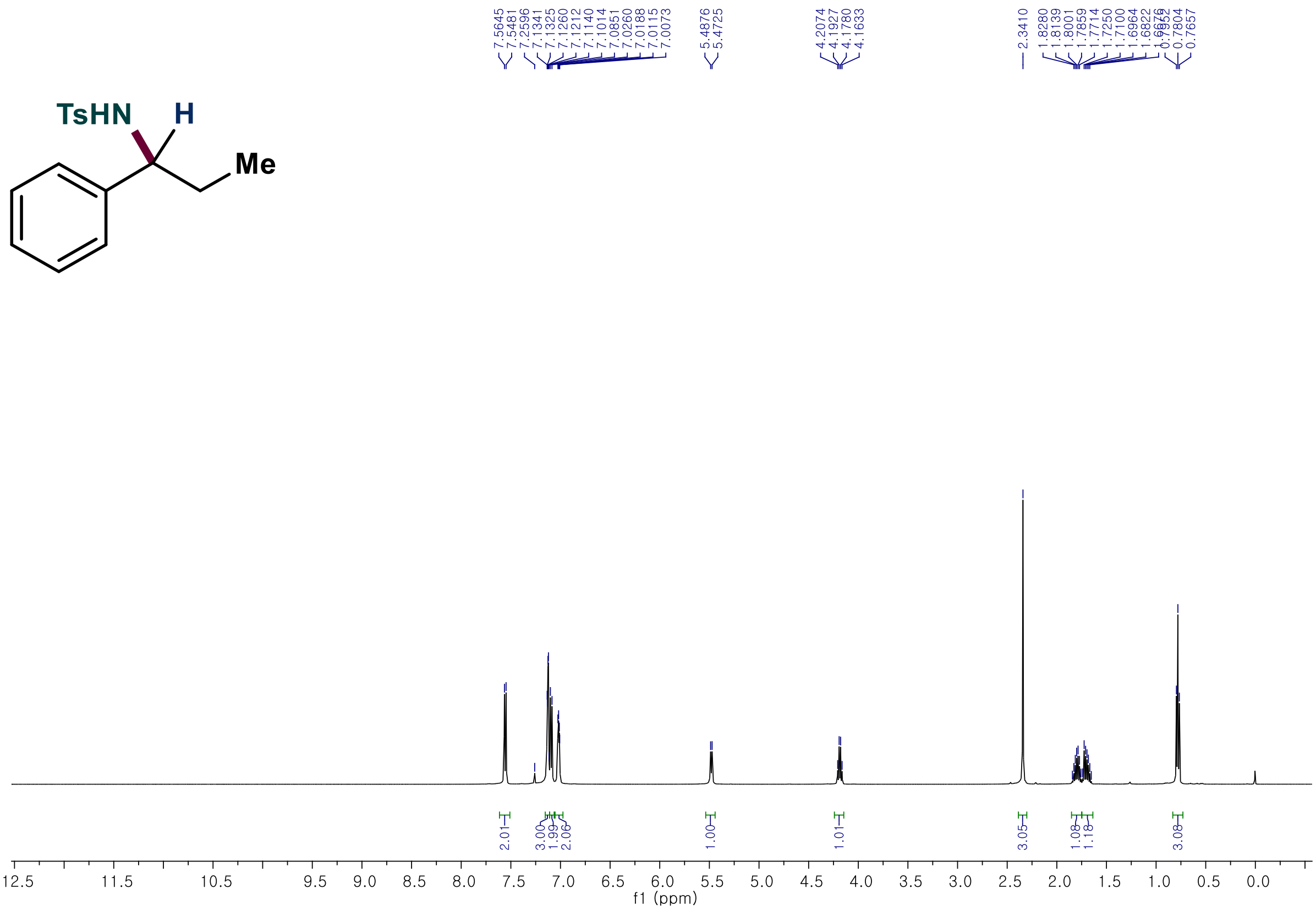

^{13}C NMR spectra (**39**)

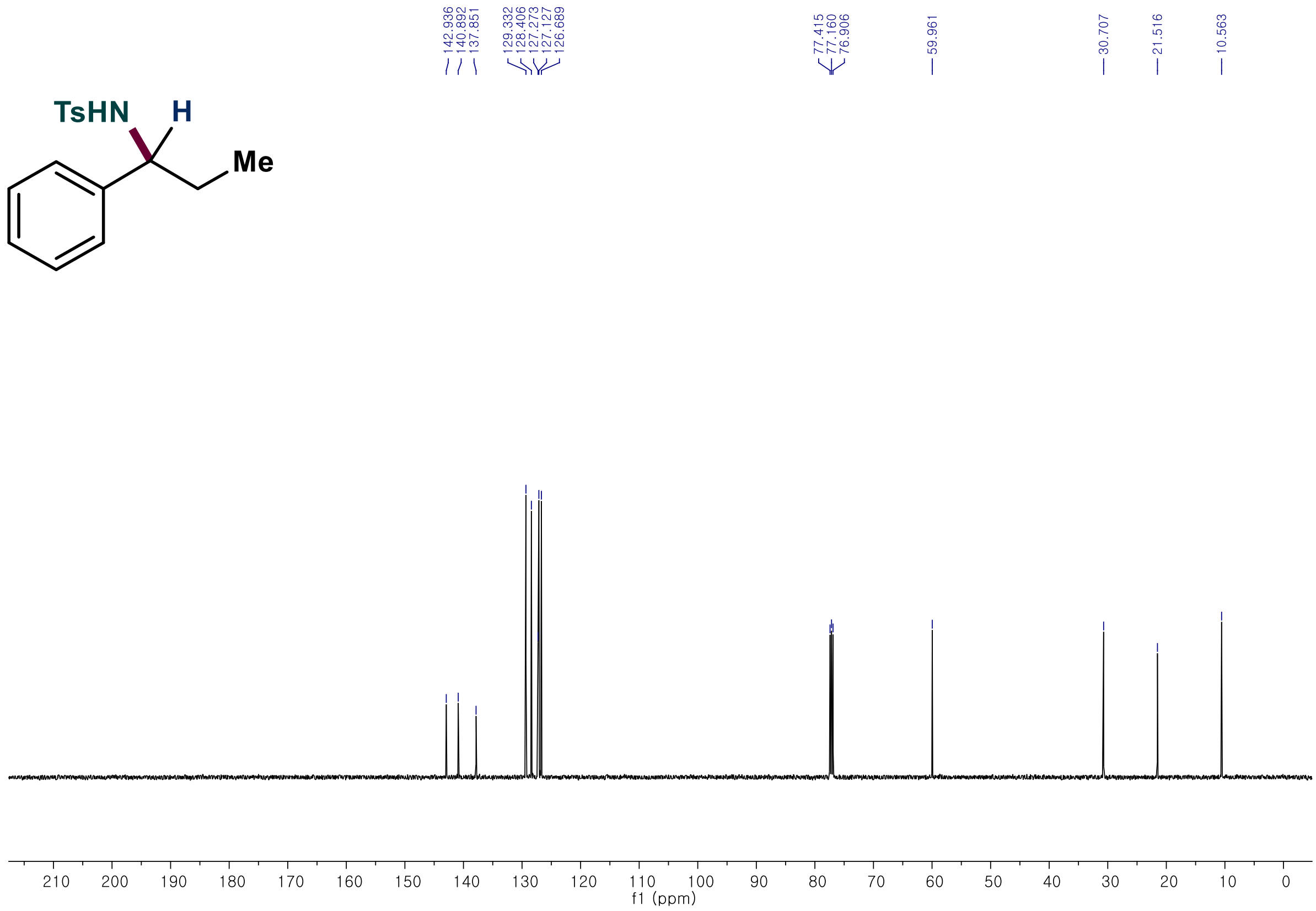

^{1}H NMR spectra (**40**)

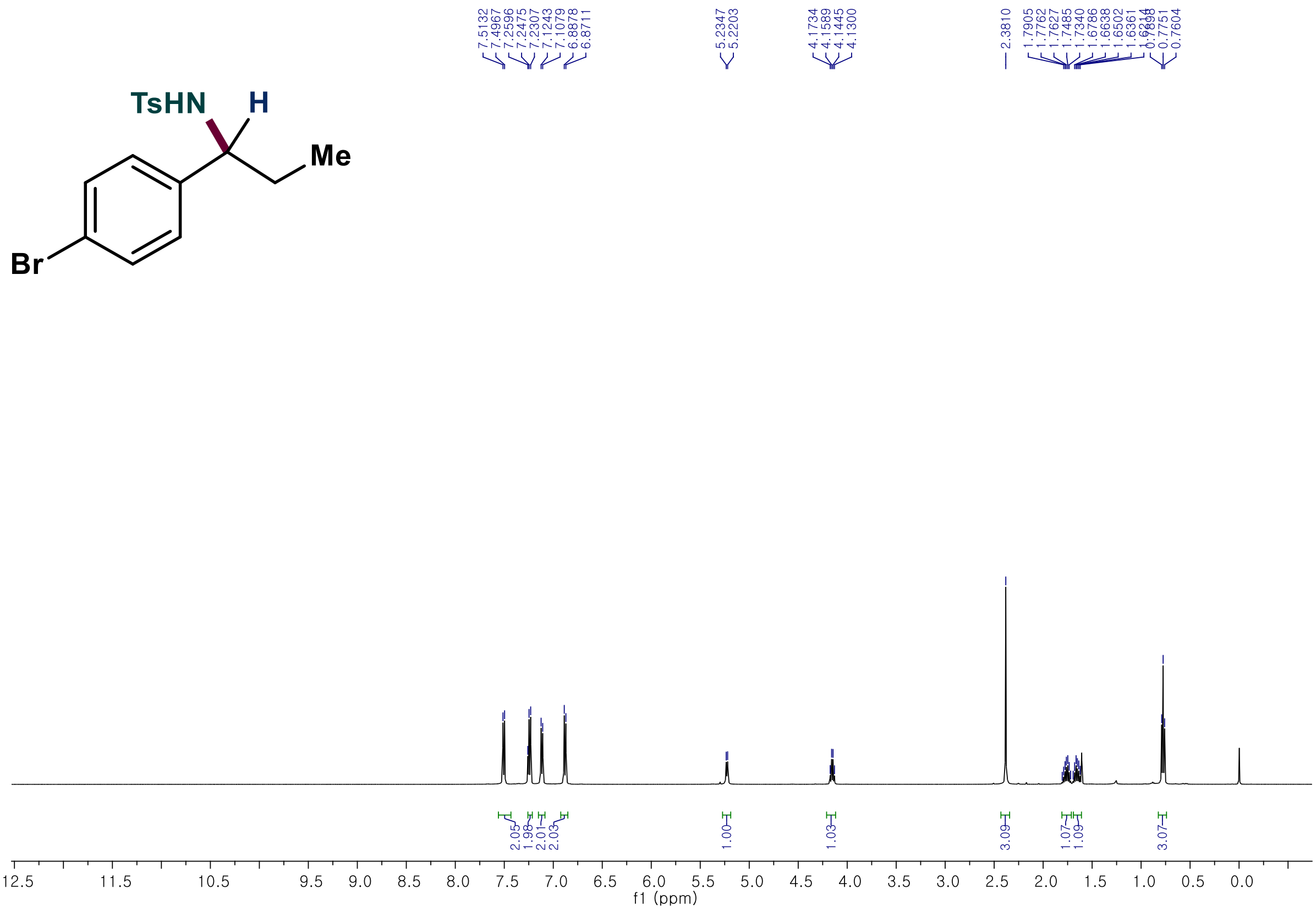

^{13}C NMR spectra (**40**)

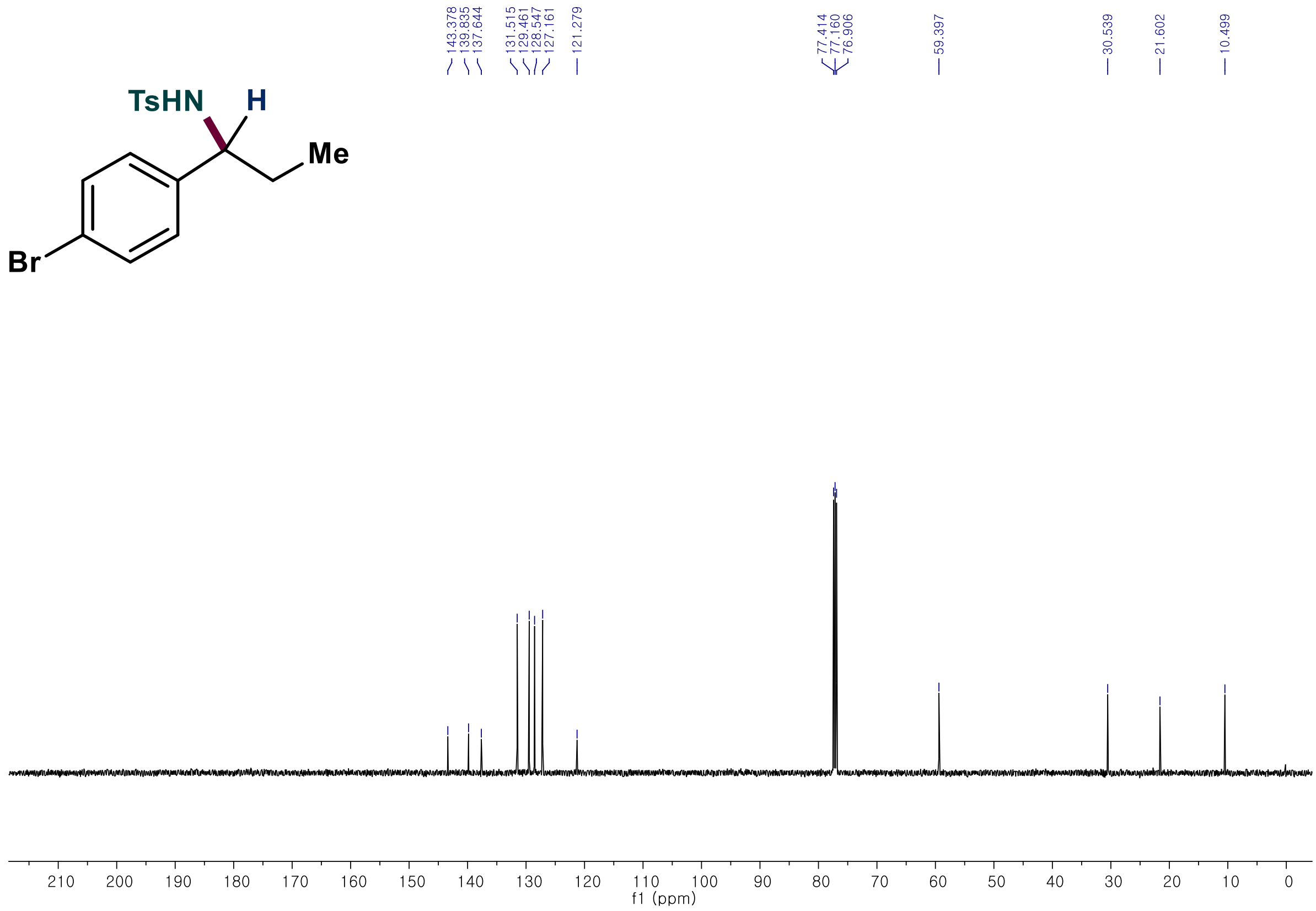

^{1}H NMR spectra (**41**)

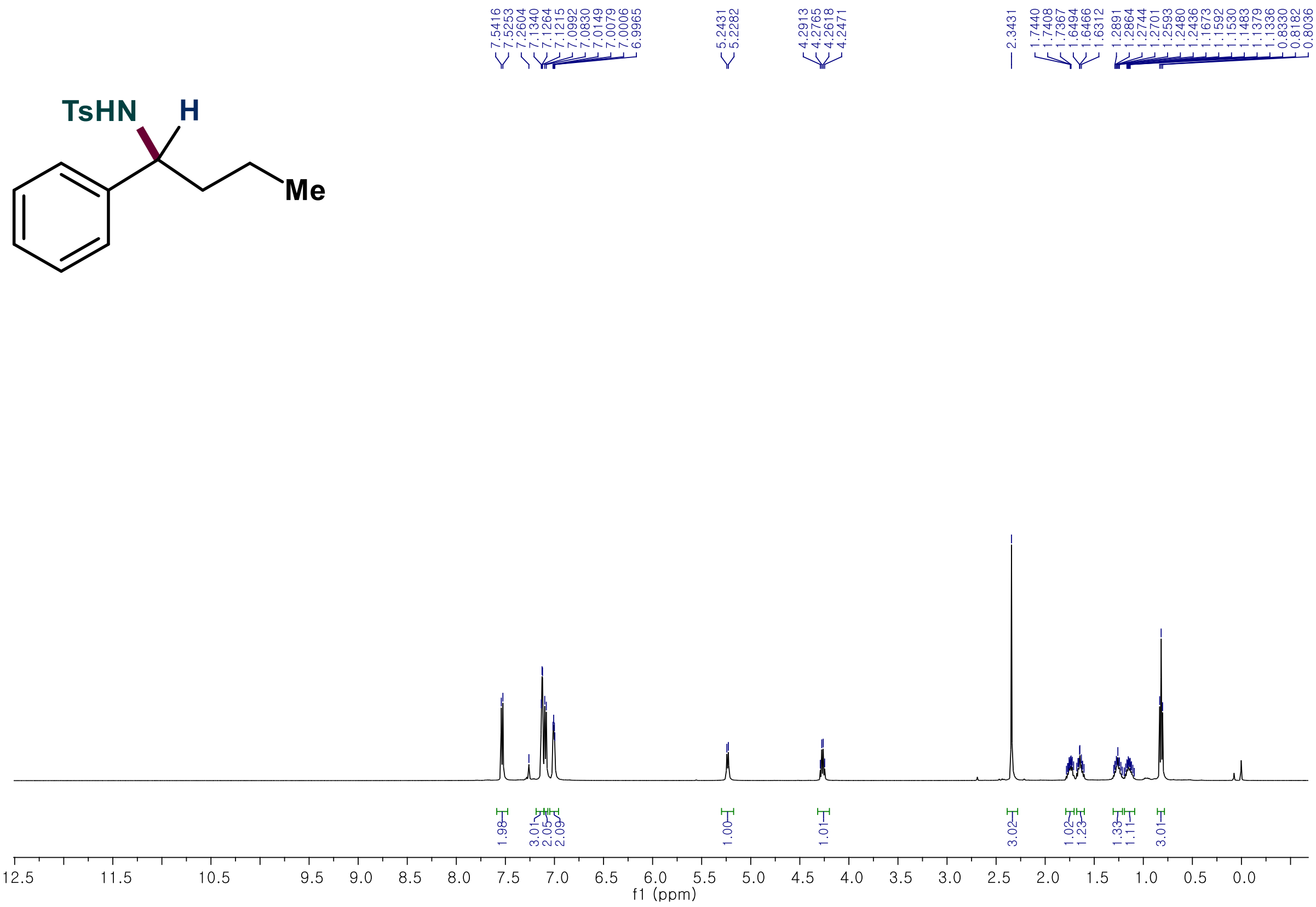

^{13}C NMR spectra (**41**)

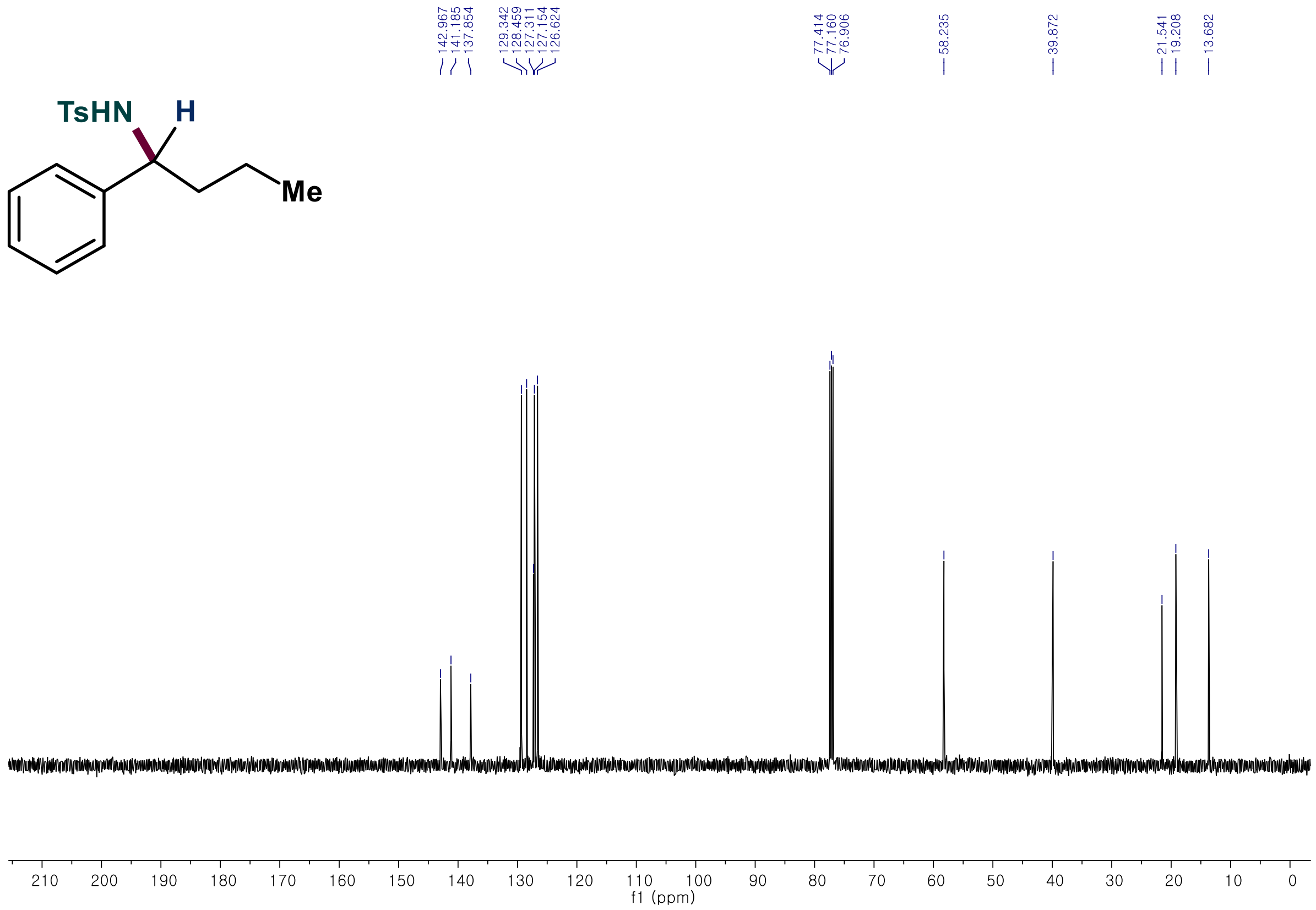

^{1}H NMR spectra (**42**)

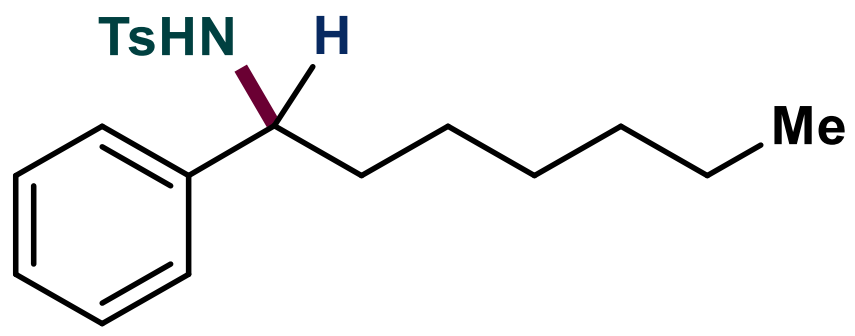

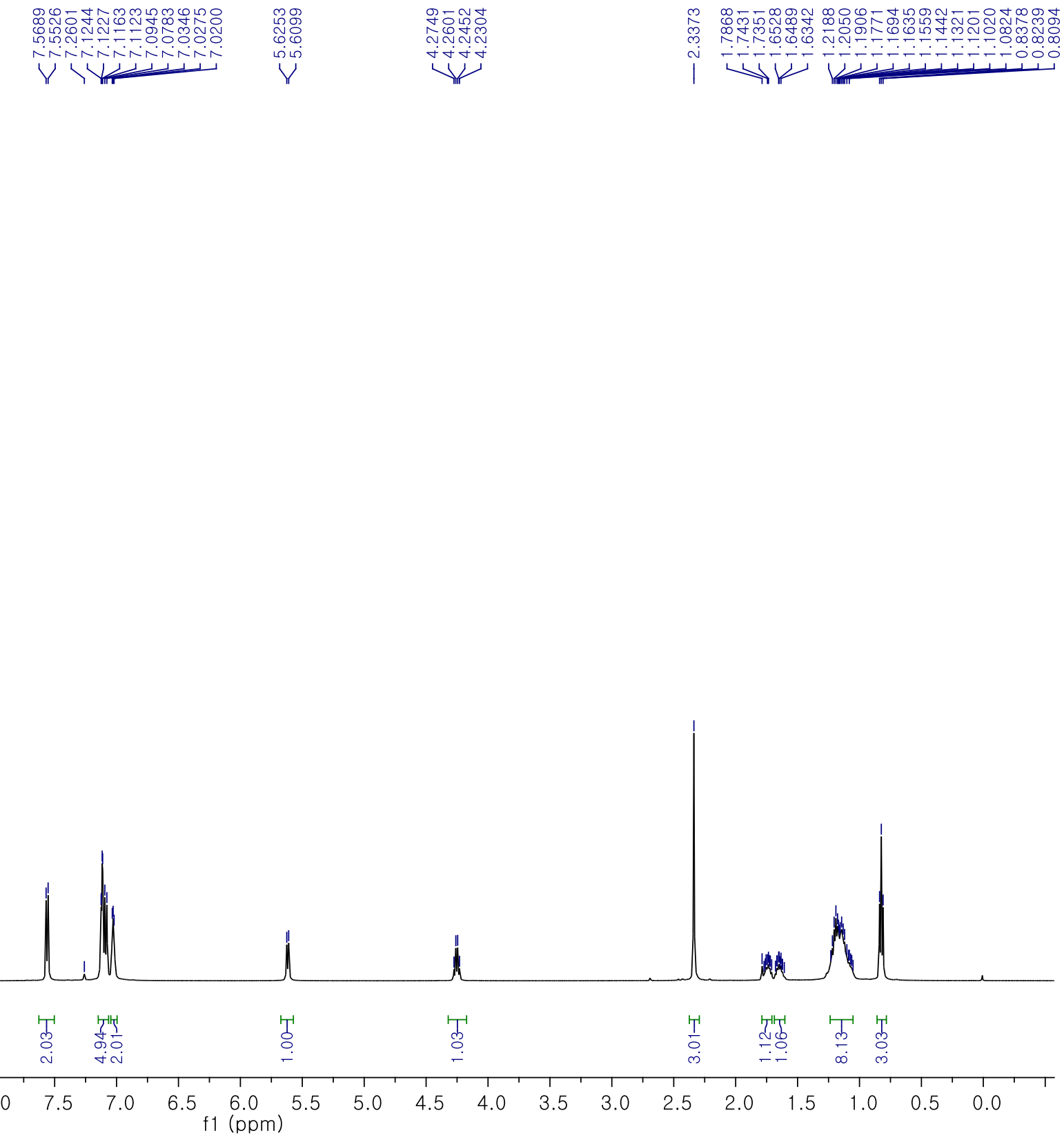

^{13}C NMR spectra (**42**)

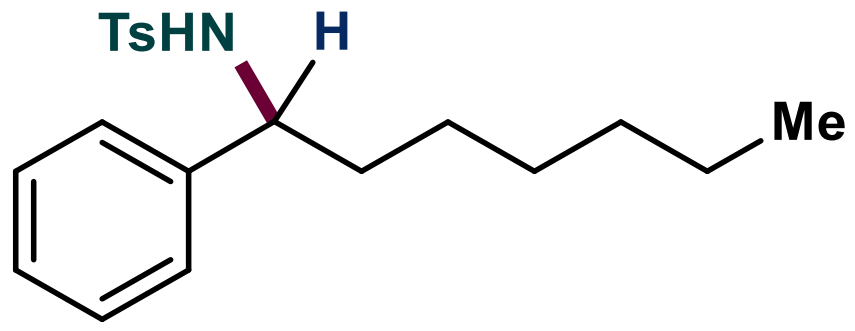

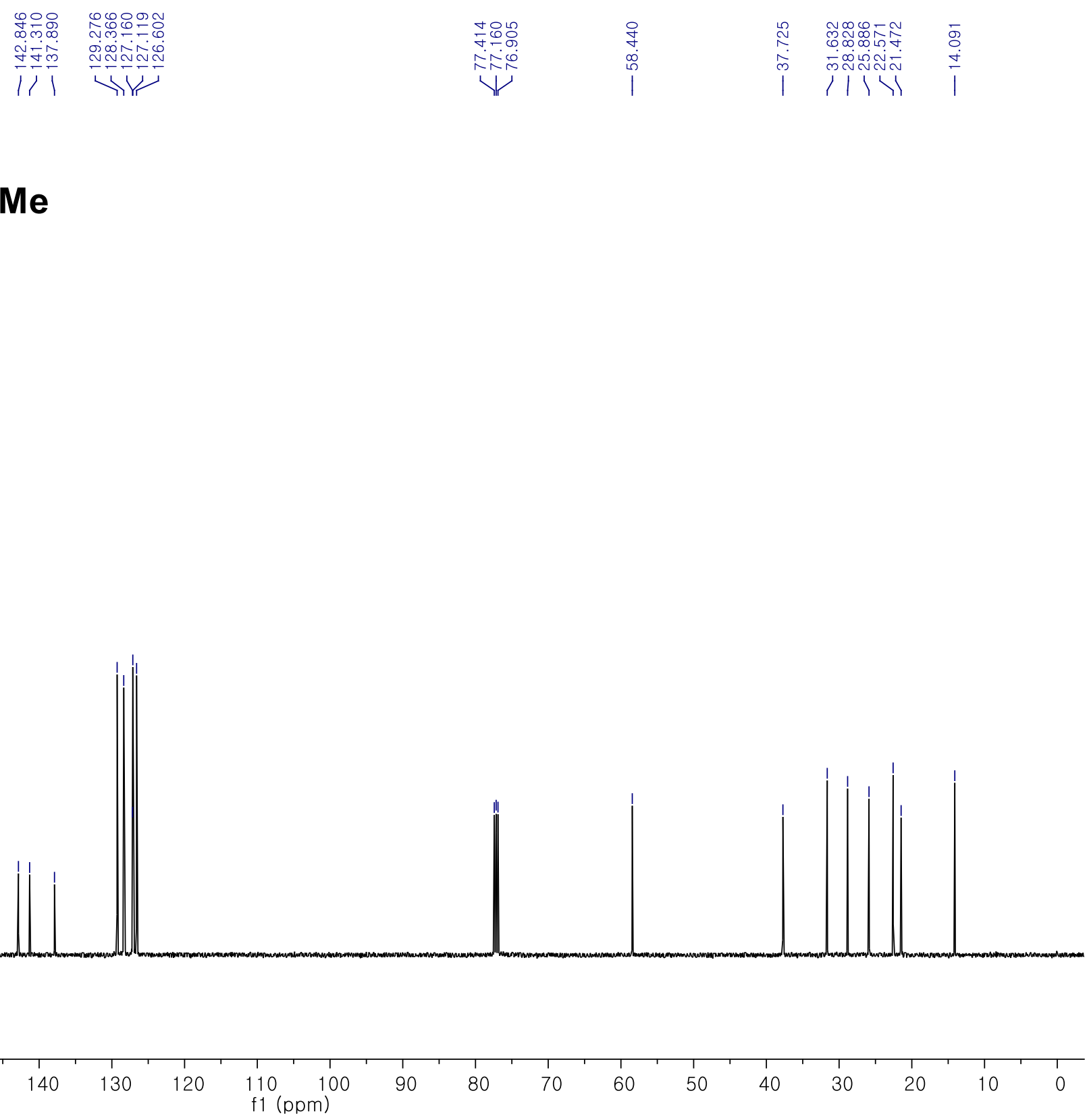

^{1}H NMR spectra (**43**)

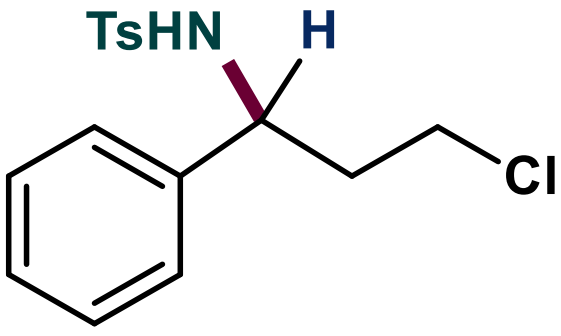

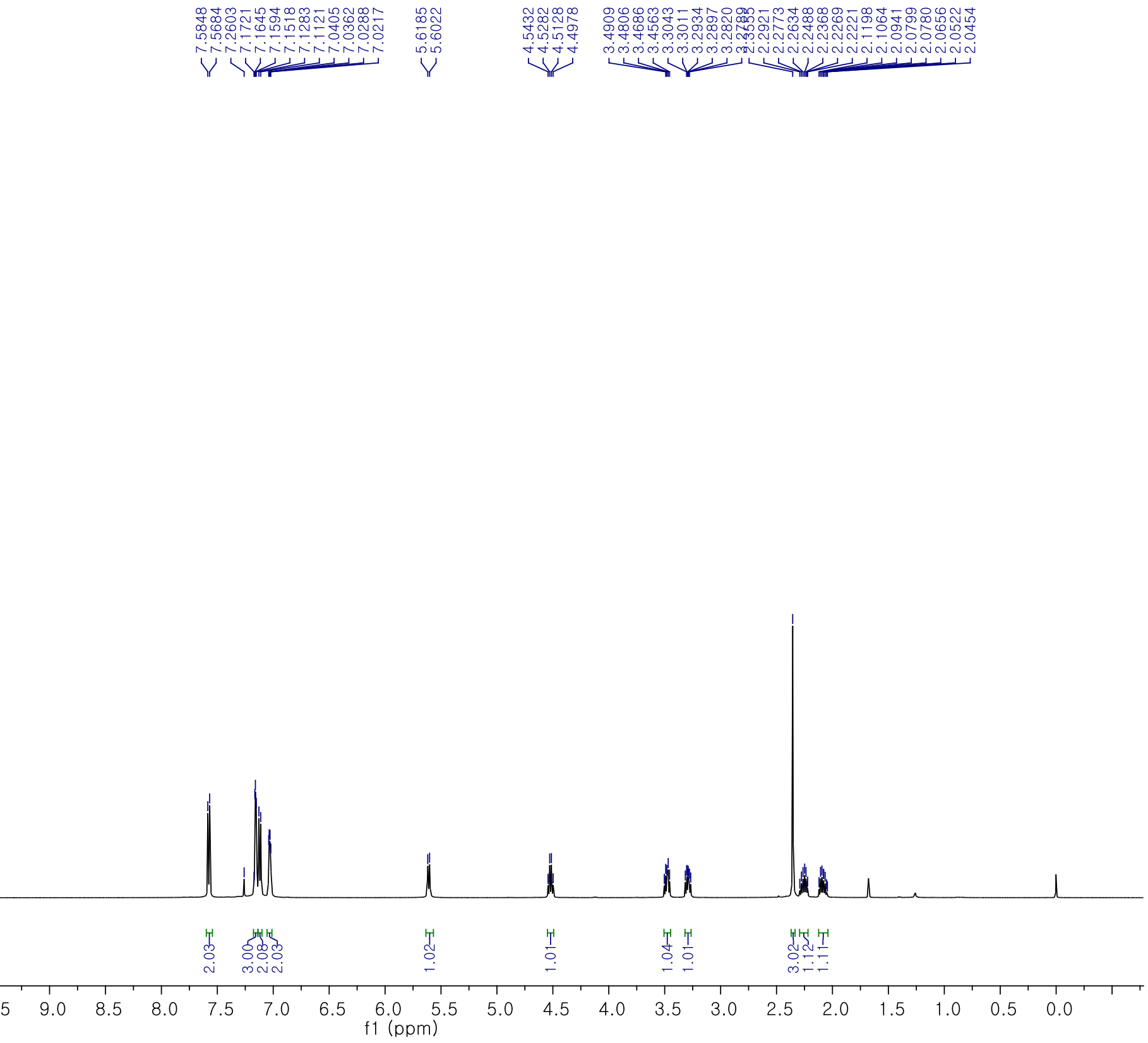

^{13}C NMR spectra (**43**)

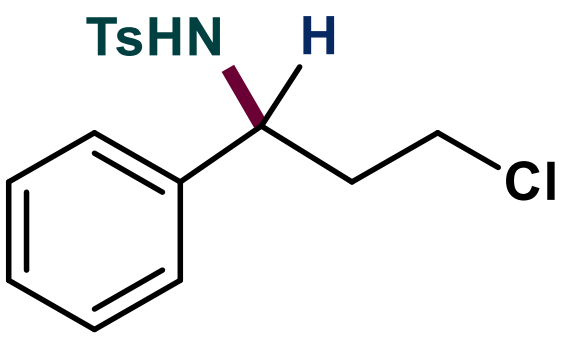

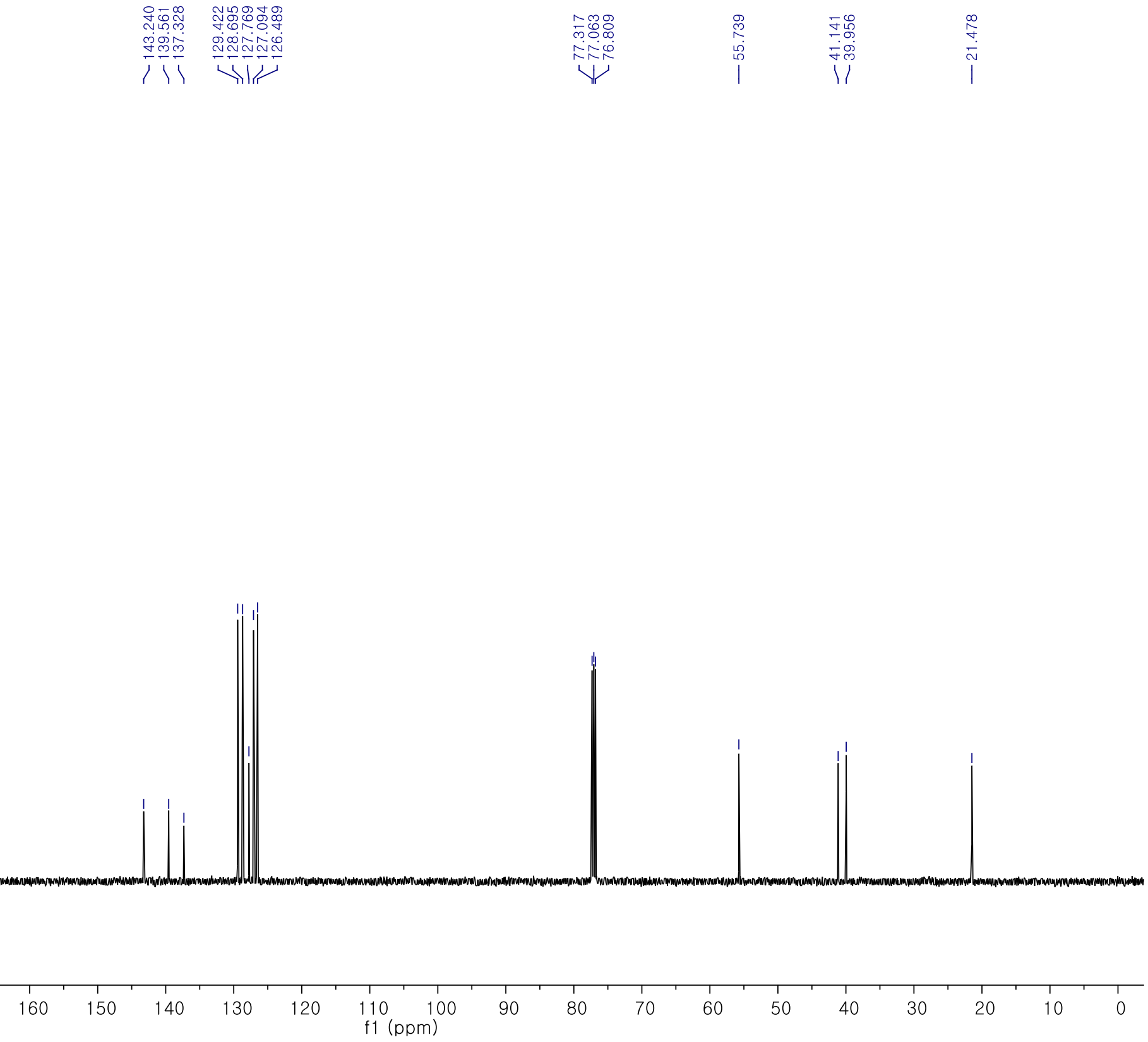

^{1}H NMR spectra (**44**)

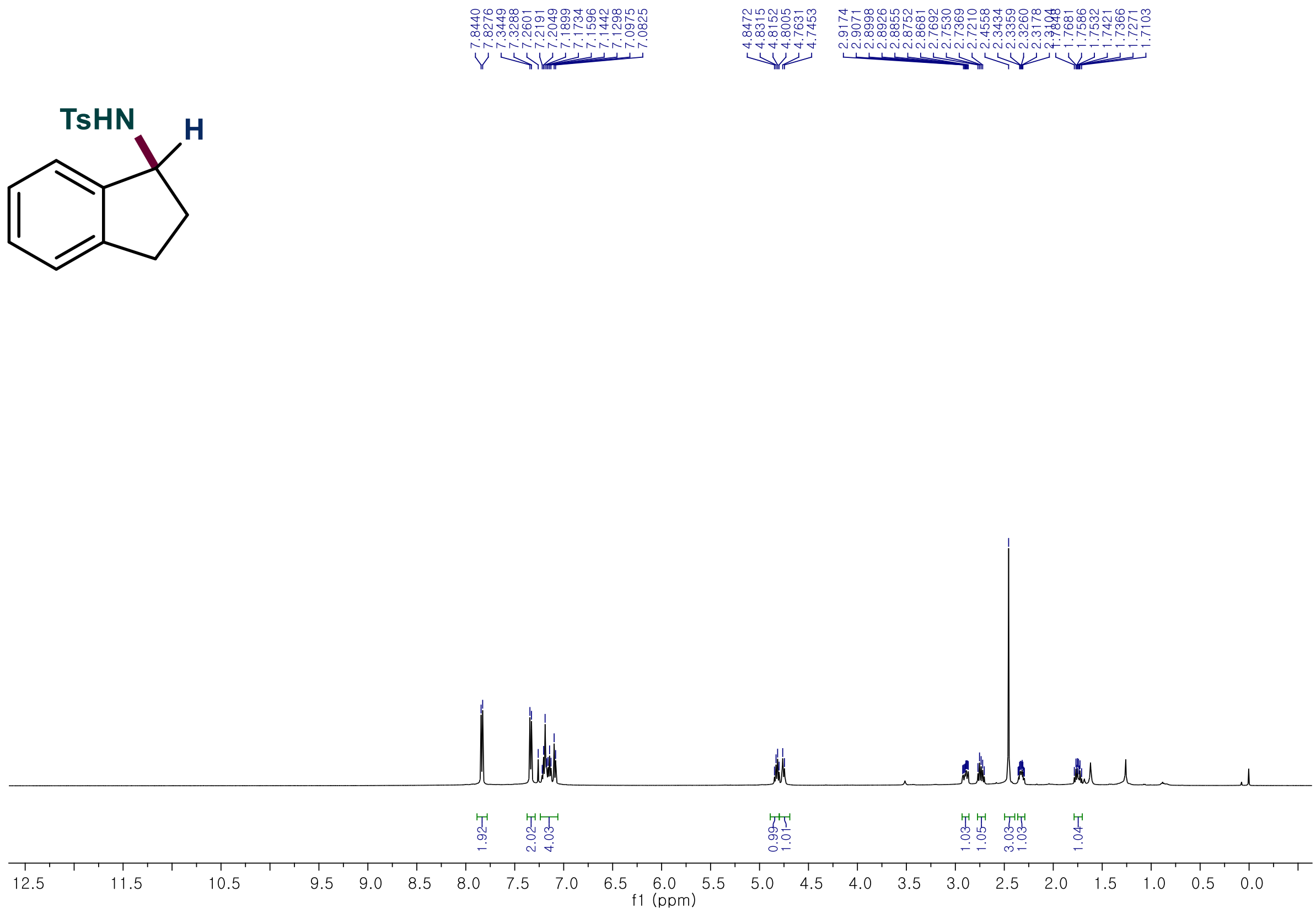

^{13}C NMR spectra (**44**)

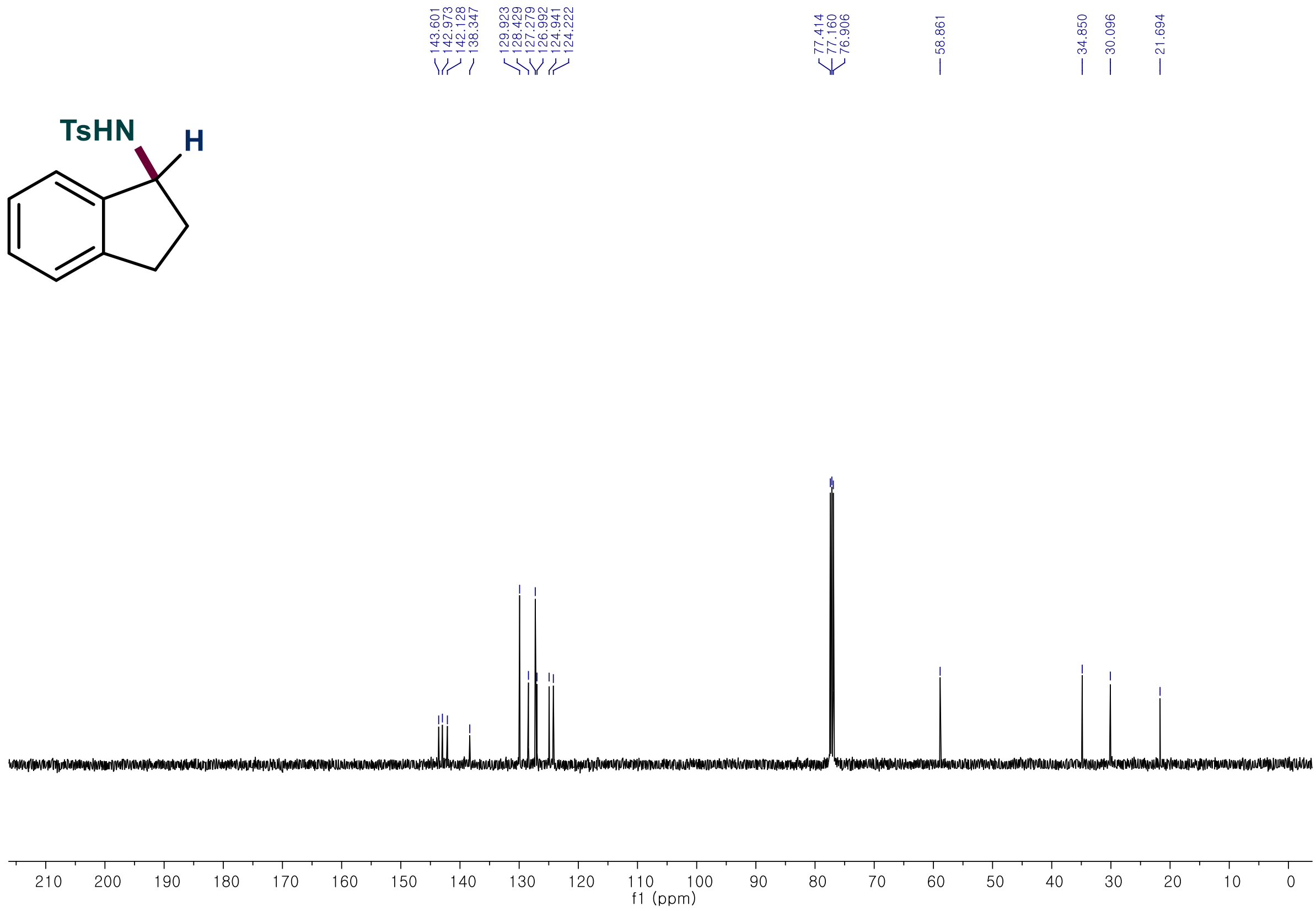

^{1}H NMR spectra (**45**)

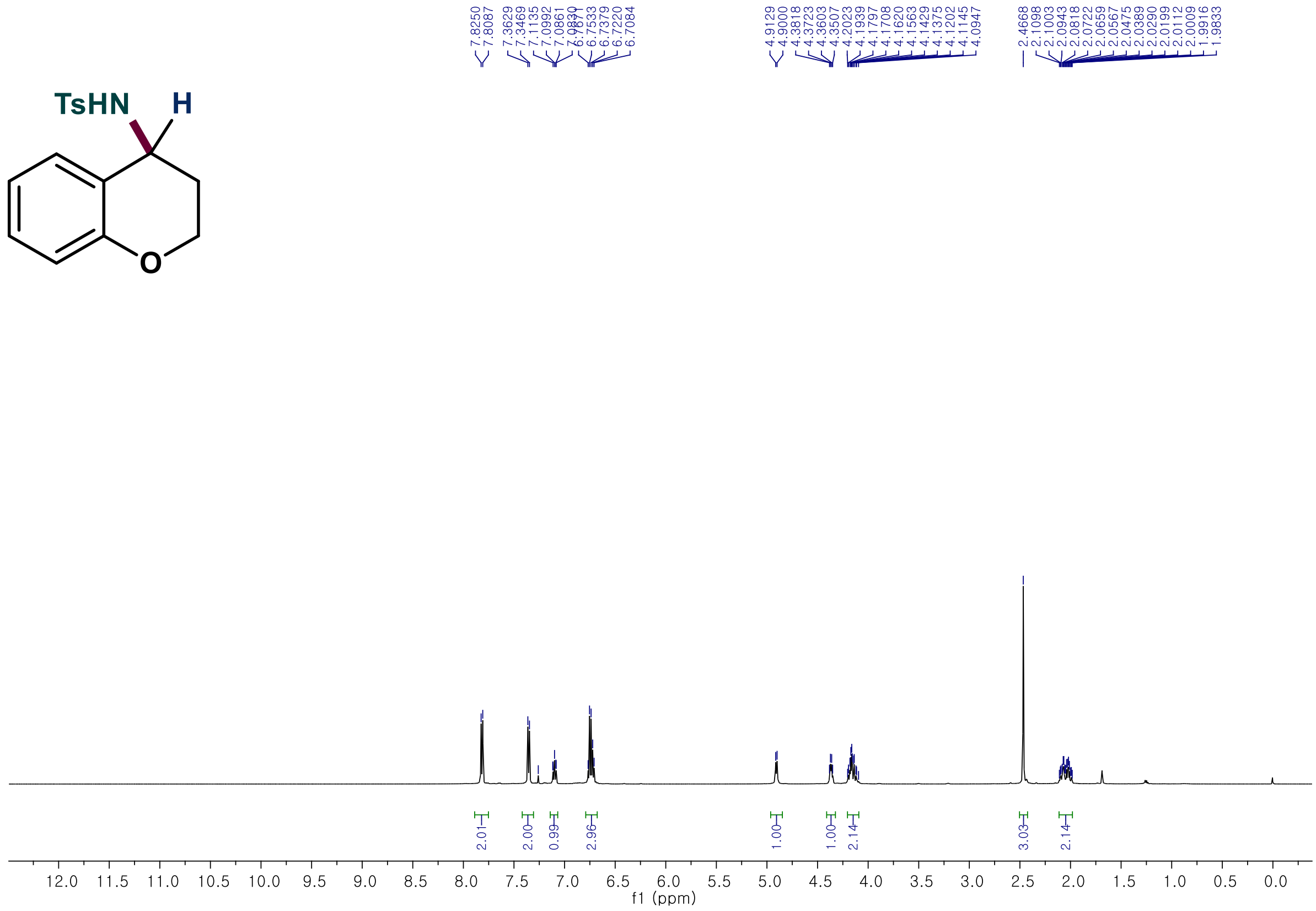

^{13}C NMR spectra (**45**)

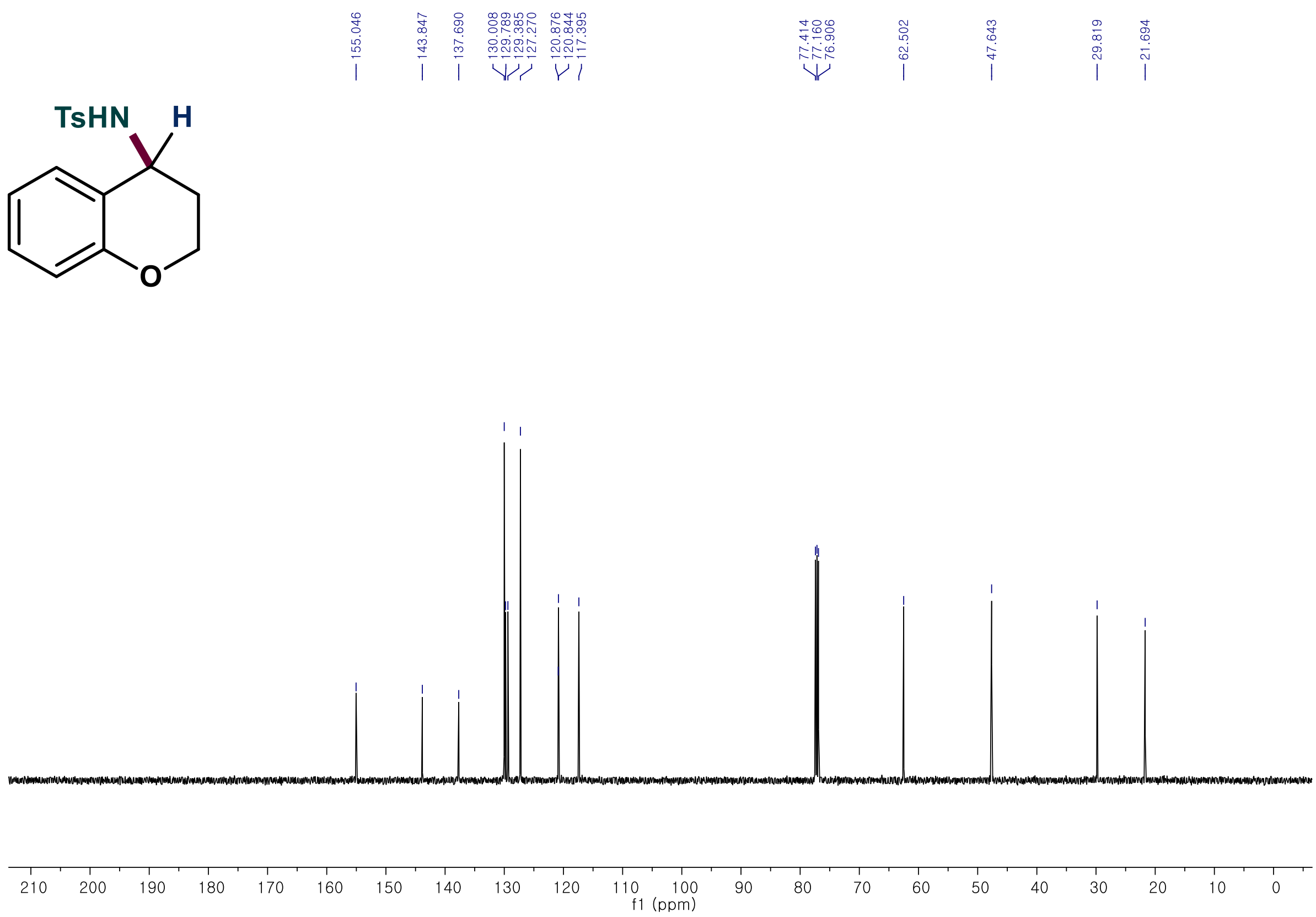

^{1}H NMR spectra (**46**)

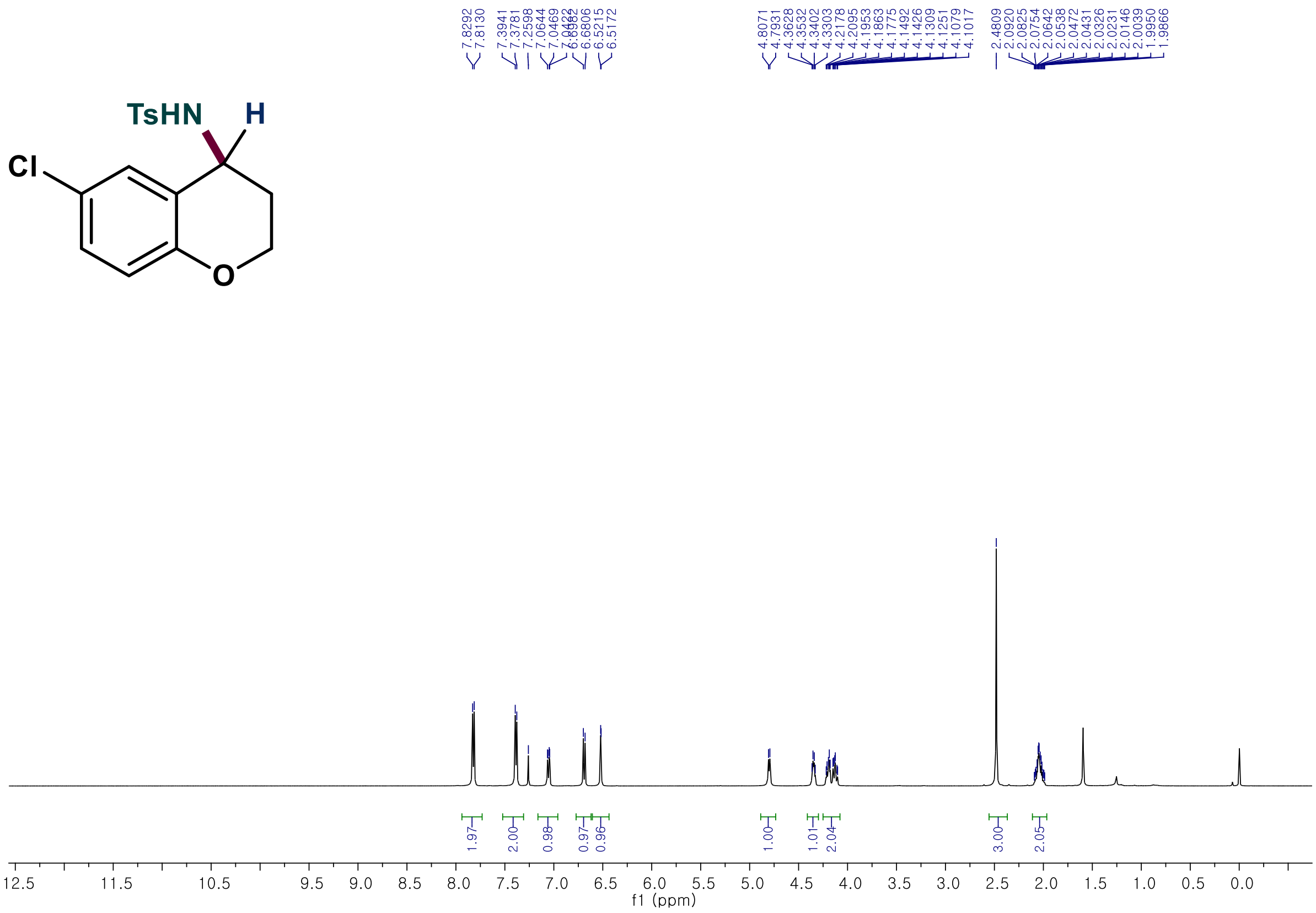

^{13}C NMR spectra (**46**)

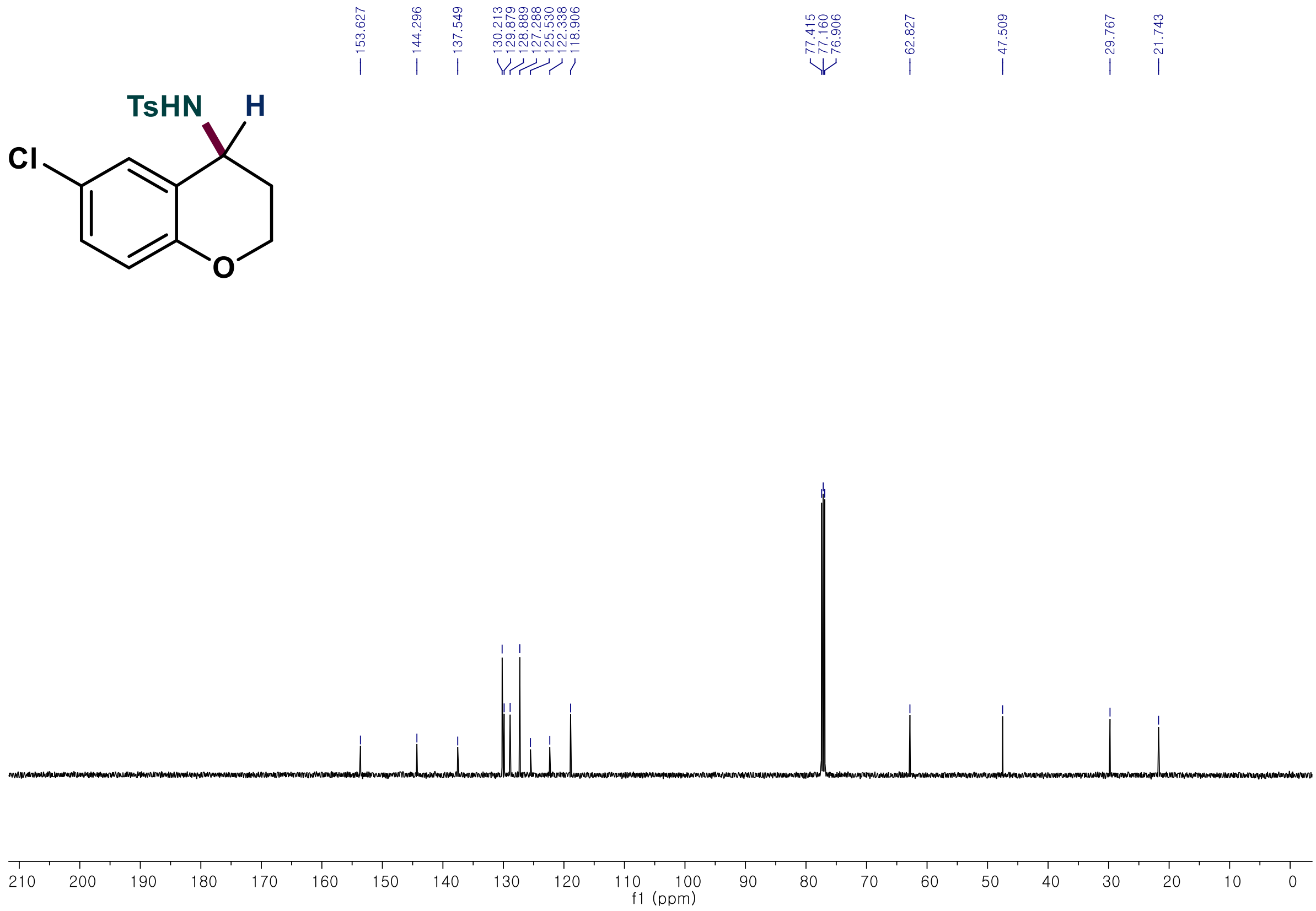

^{1}H NMR spectra (**47**)

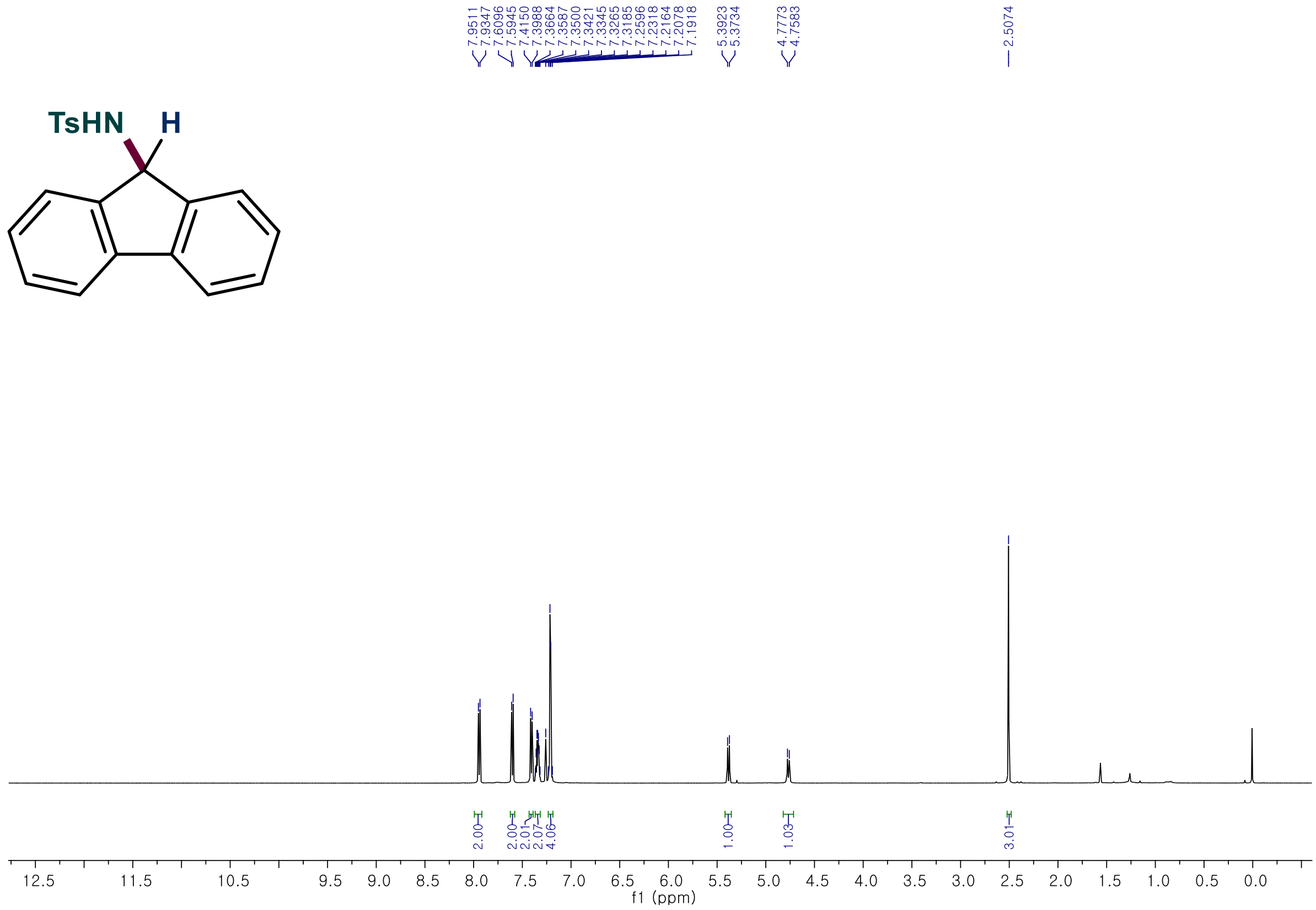

^{13}C NMR spectra (**47**)

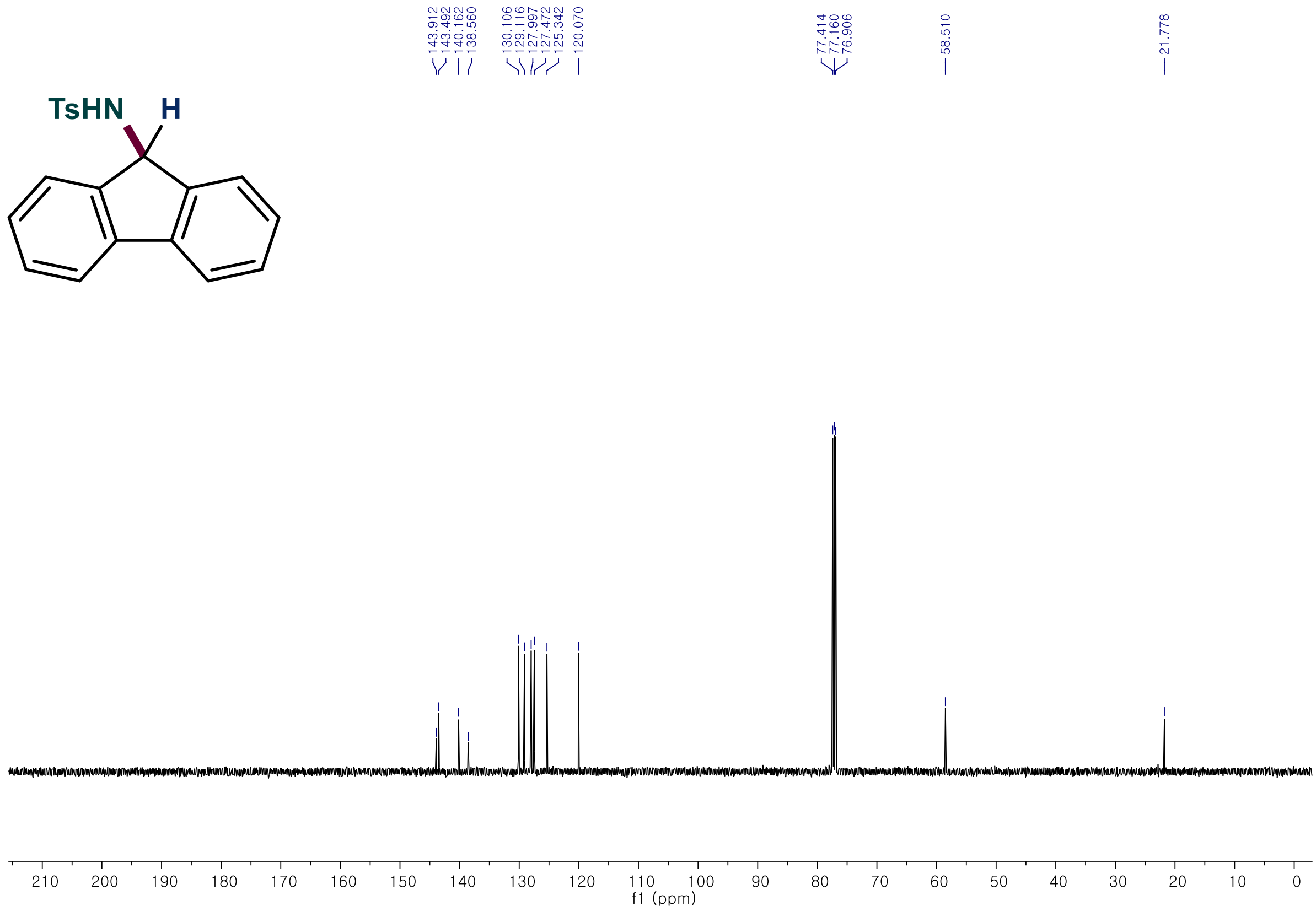

^{1}H NMR spectra (**48**)

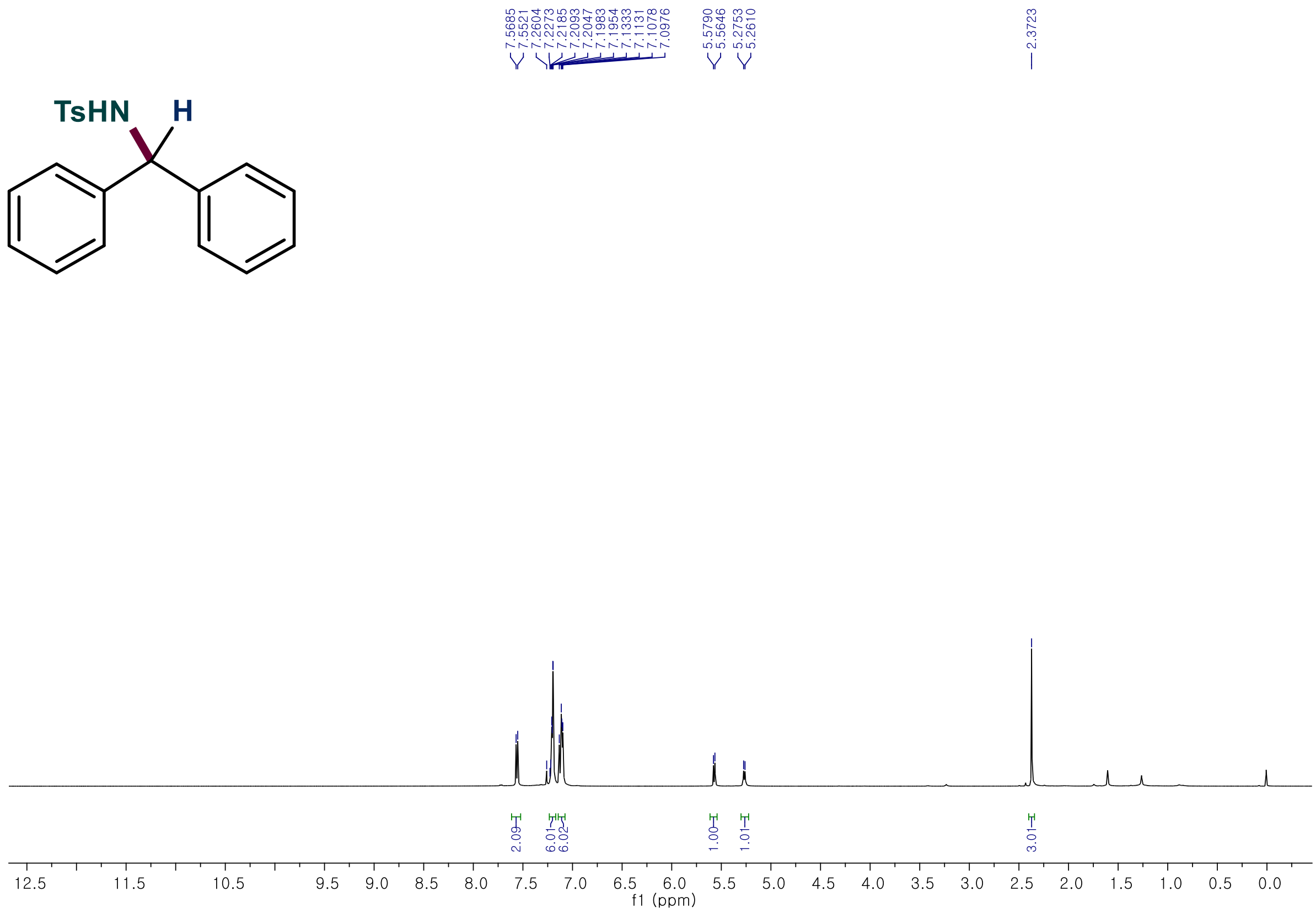

^{13}C NMR spectra (**48**)

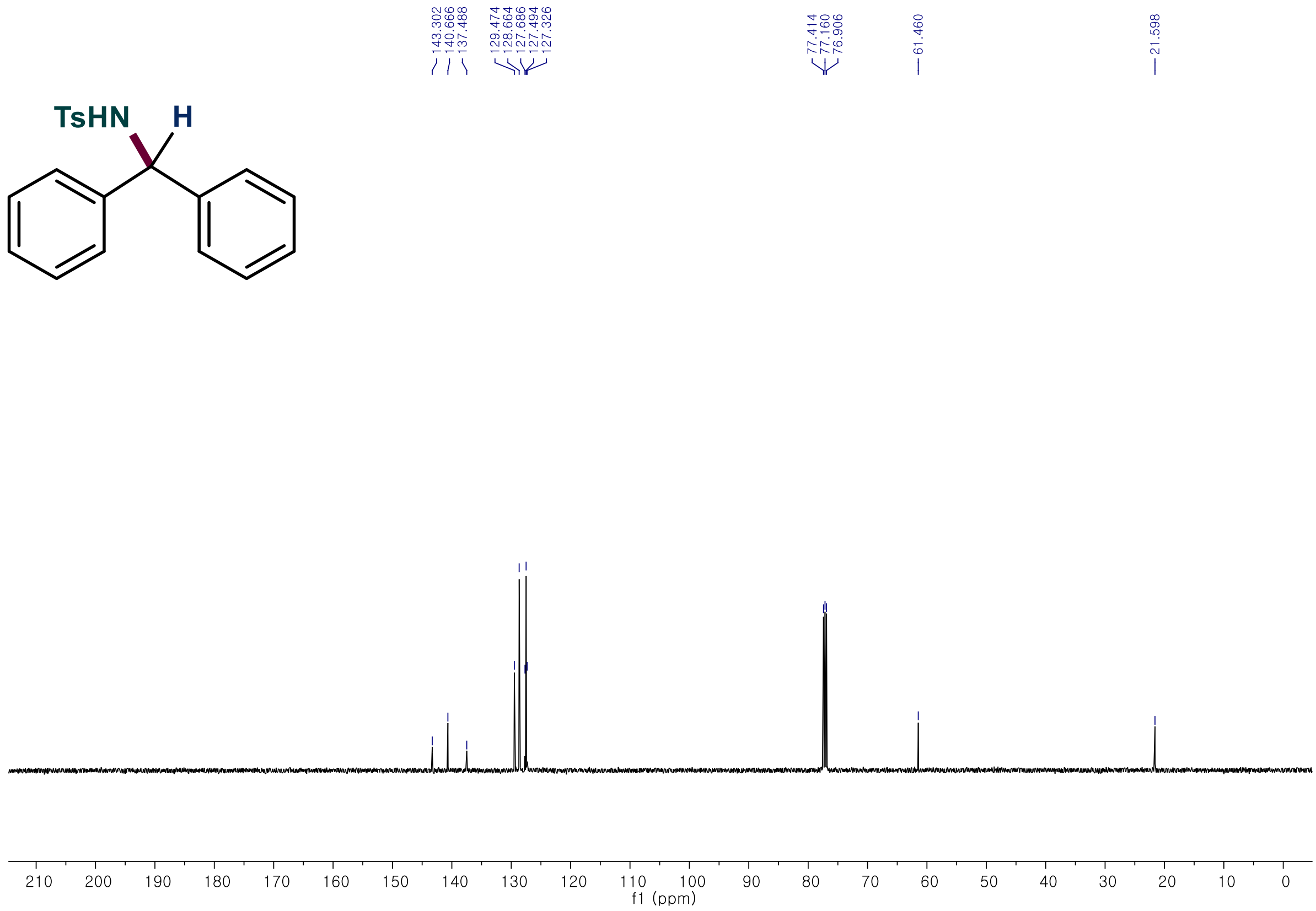

^{1}H NMR spectra (**49**)

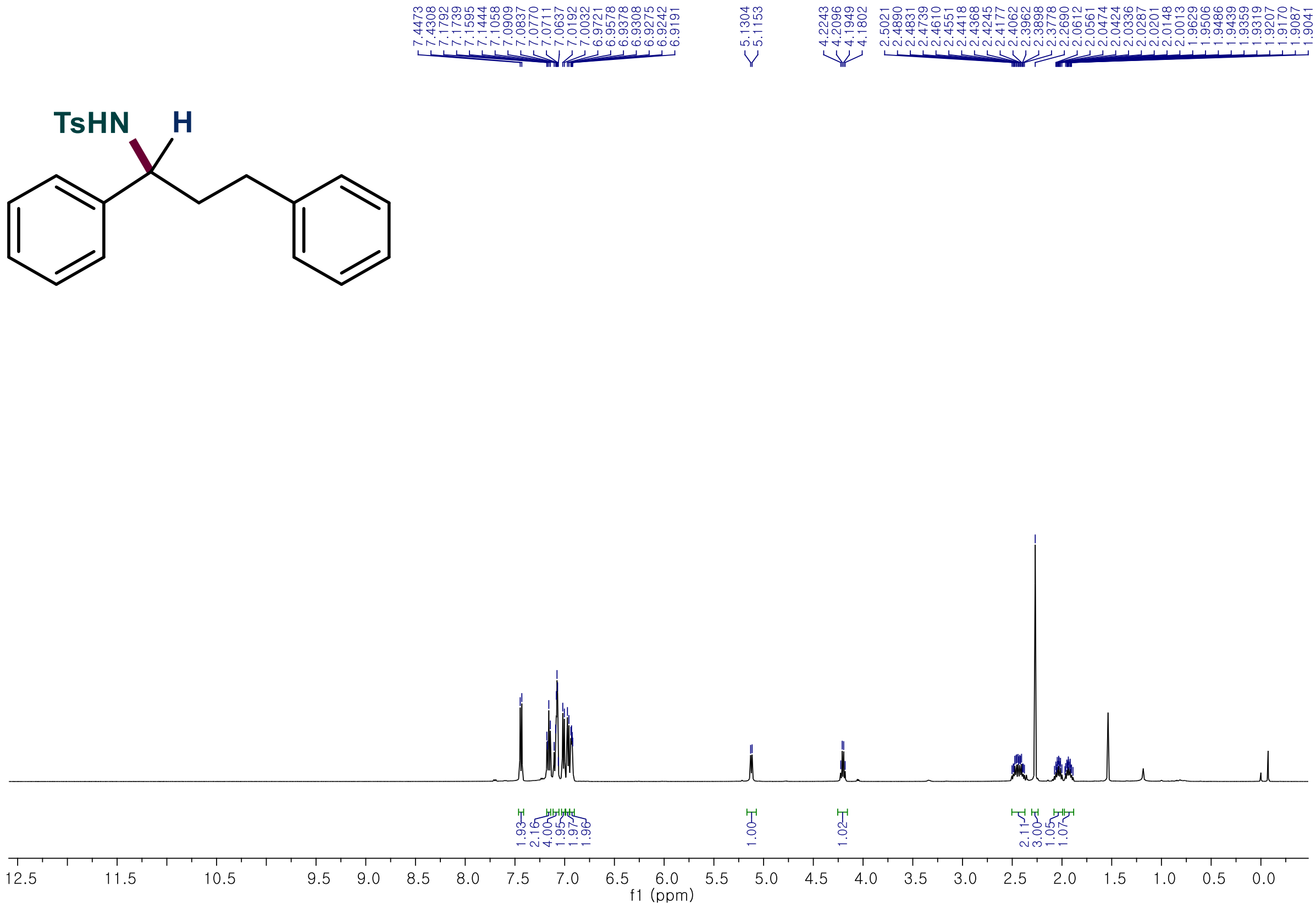

^{13}C NMR spectra (**49**)

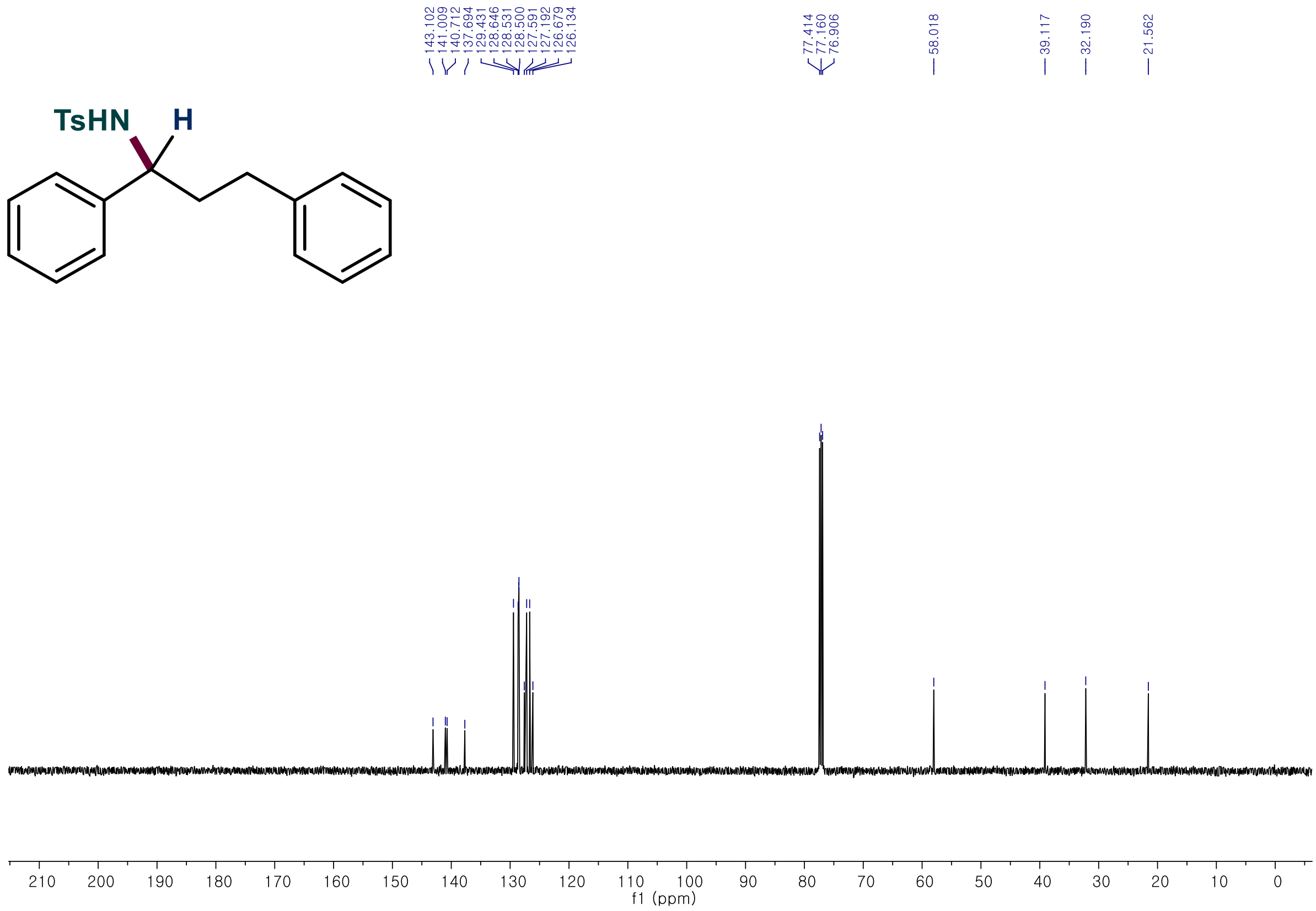

^{1}H NMR spectra (**50**)

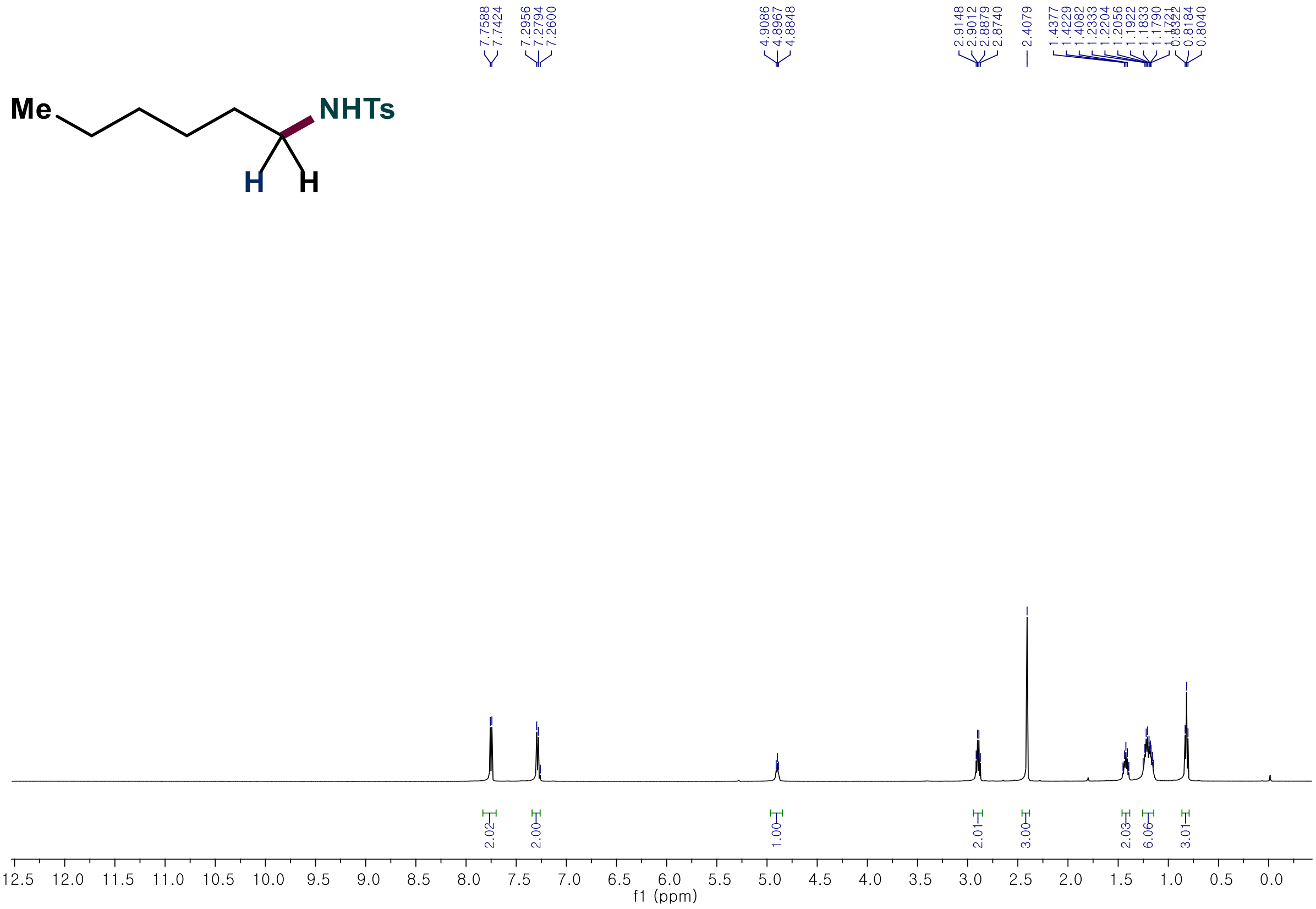

^{13}C NMR spectra (**50**)

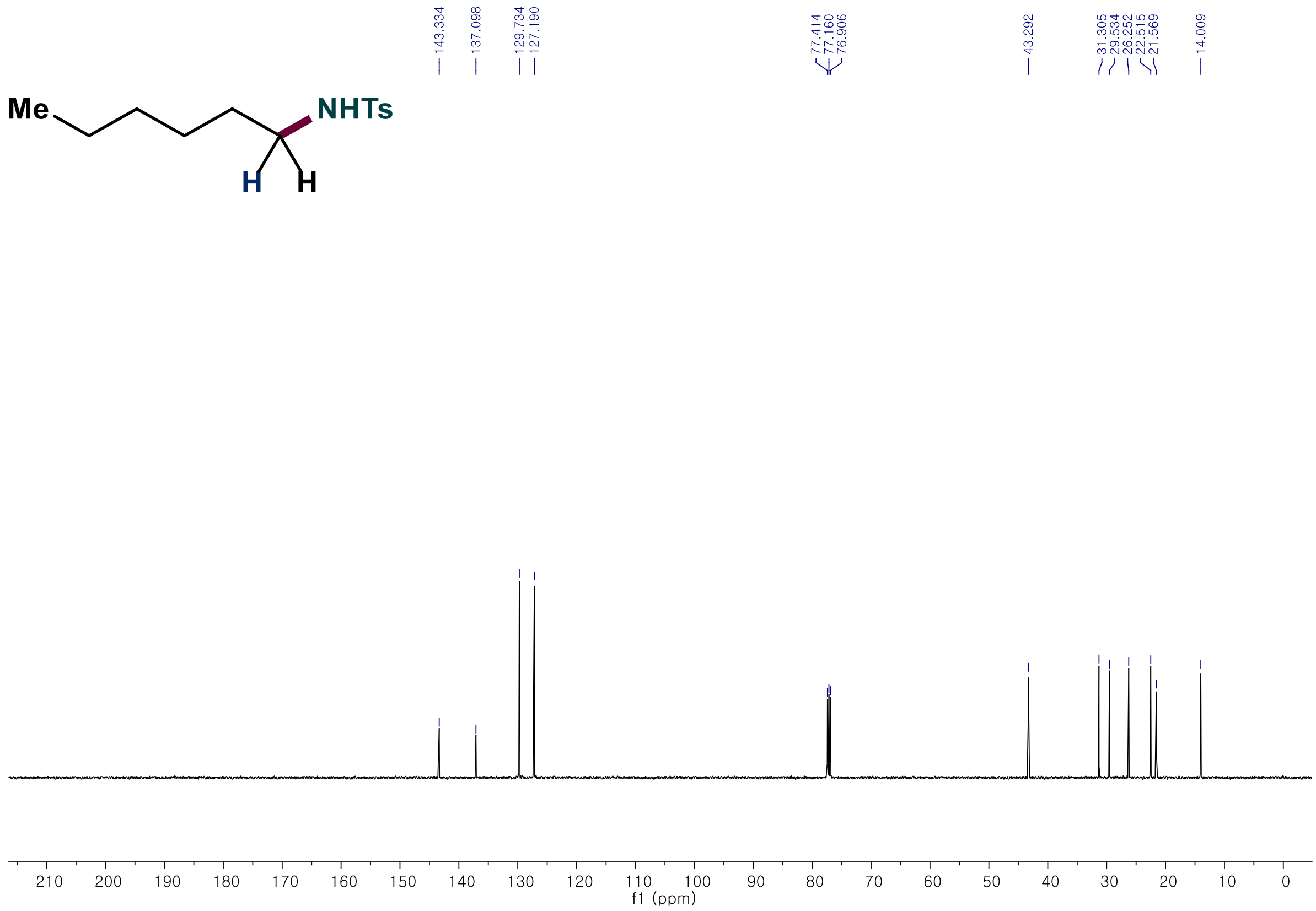

^{1}H NMR spectra (**51**)

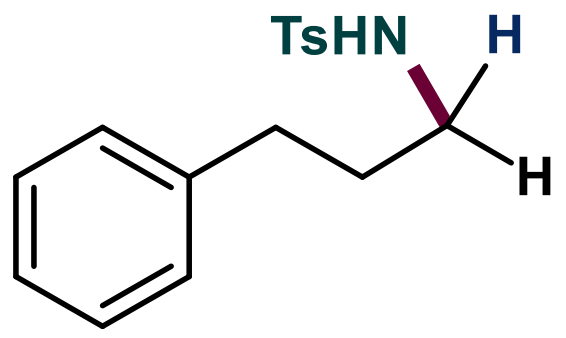

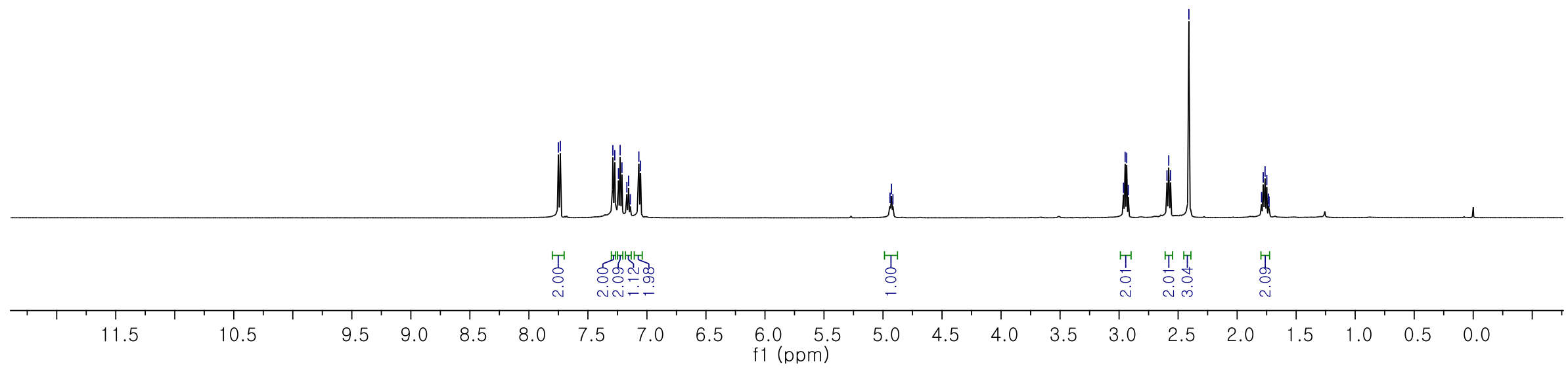

^{13}C NMR spectra (**51**)

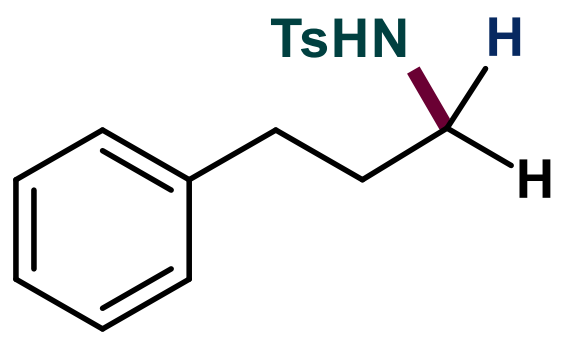

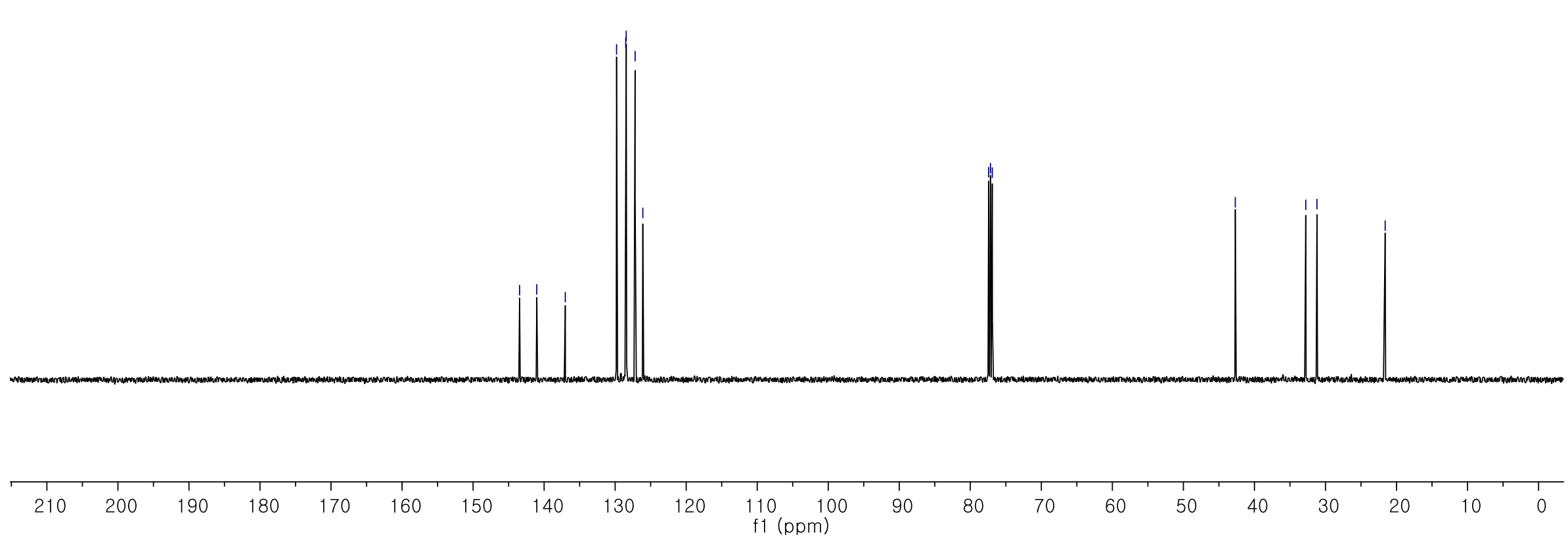

^{1}H NMR spectra (**52**)

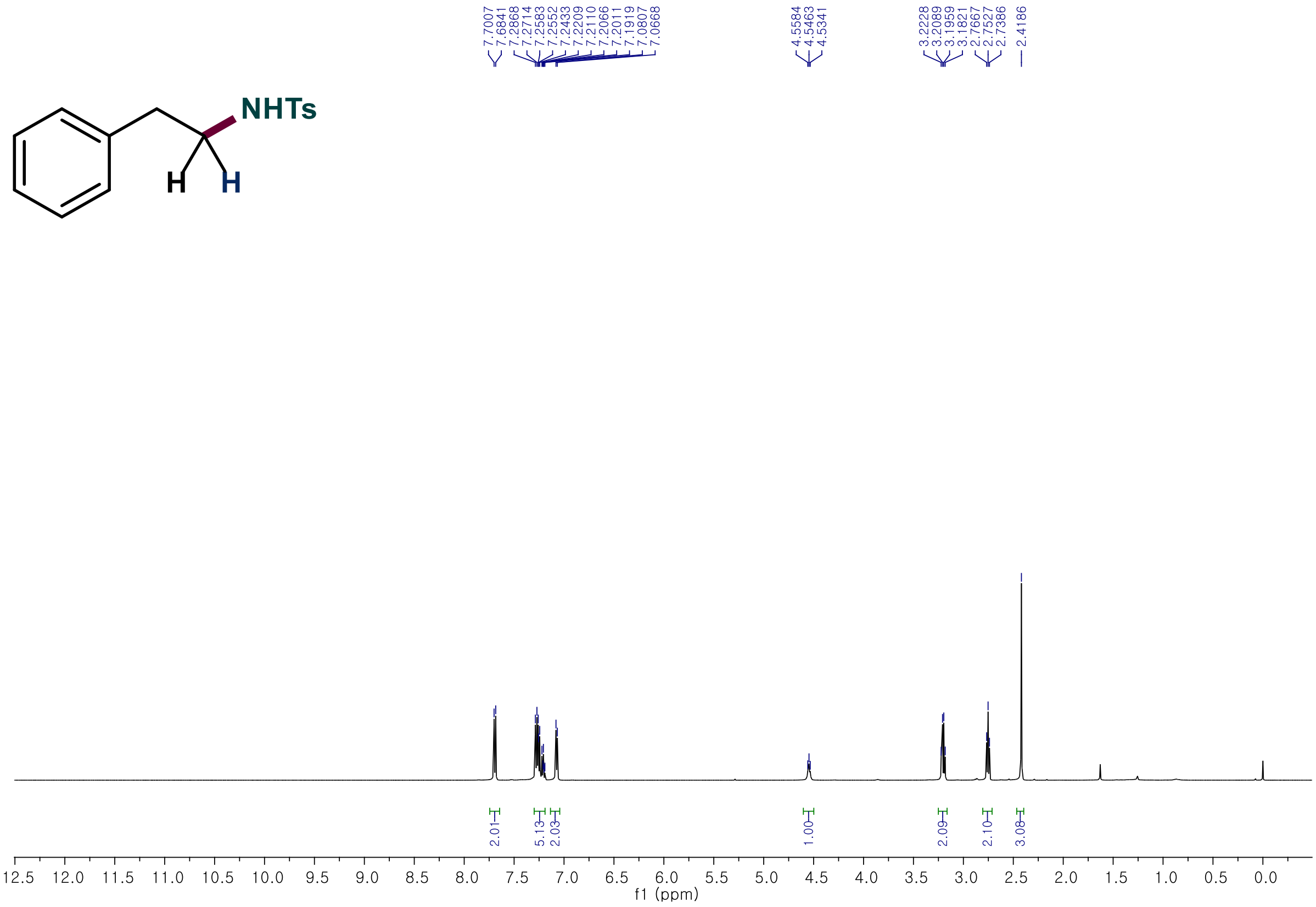

^{13}C NMR spectra (**52**)

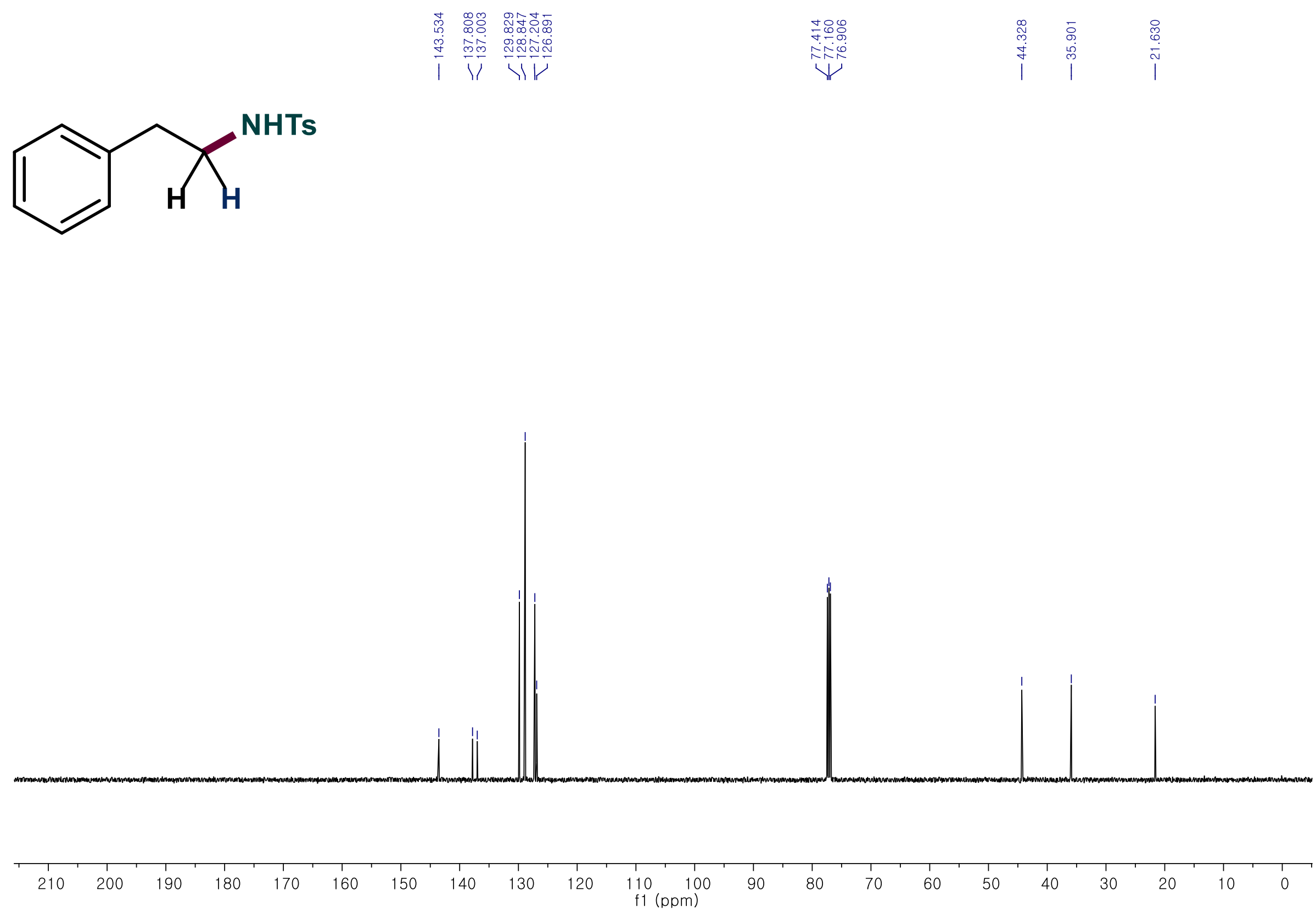

^{1}H NMR spectra (**53**)

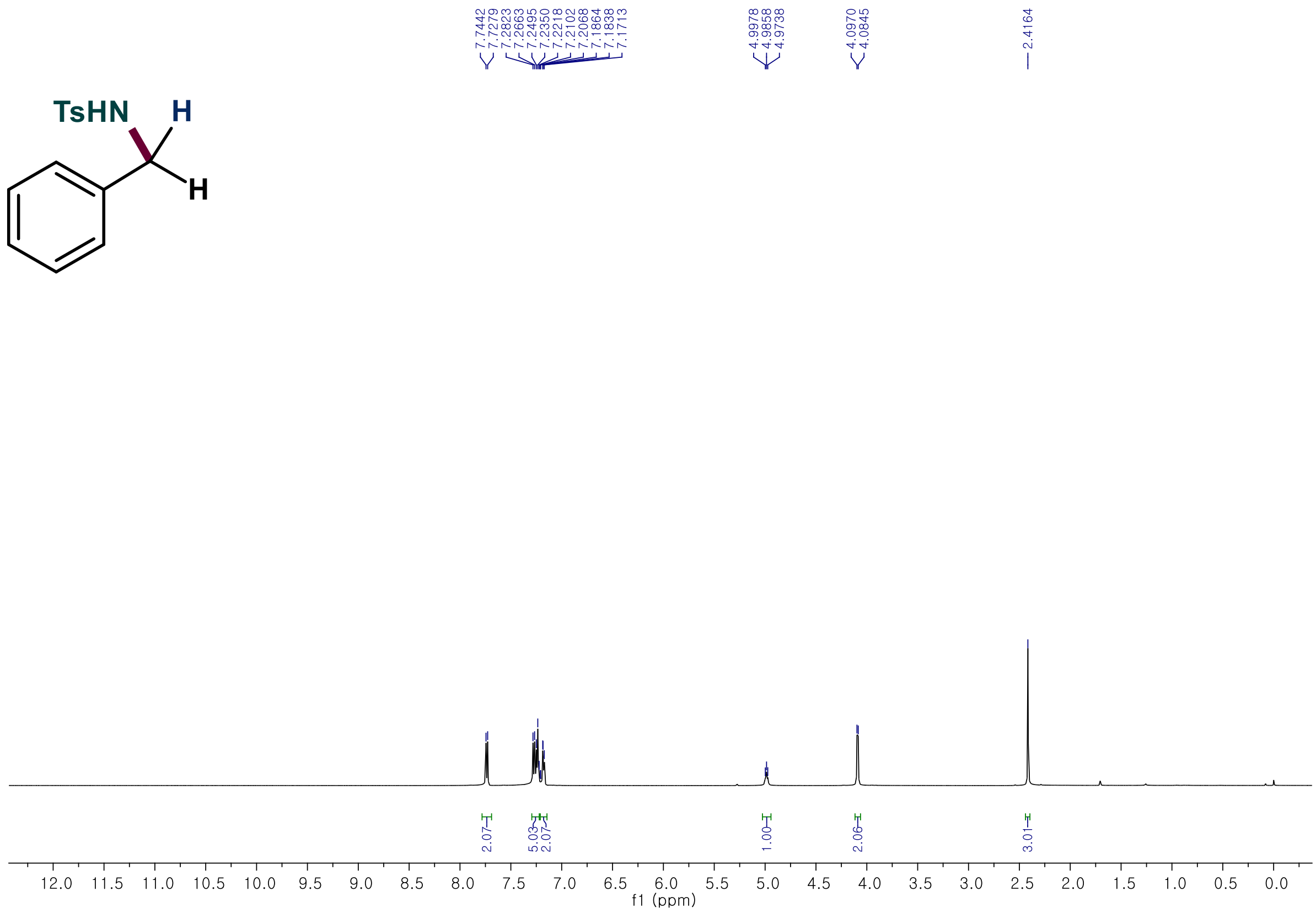

^{13}C NMR spectra (**53**)

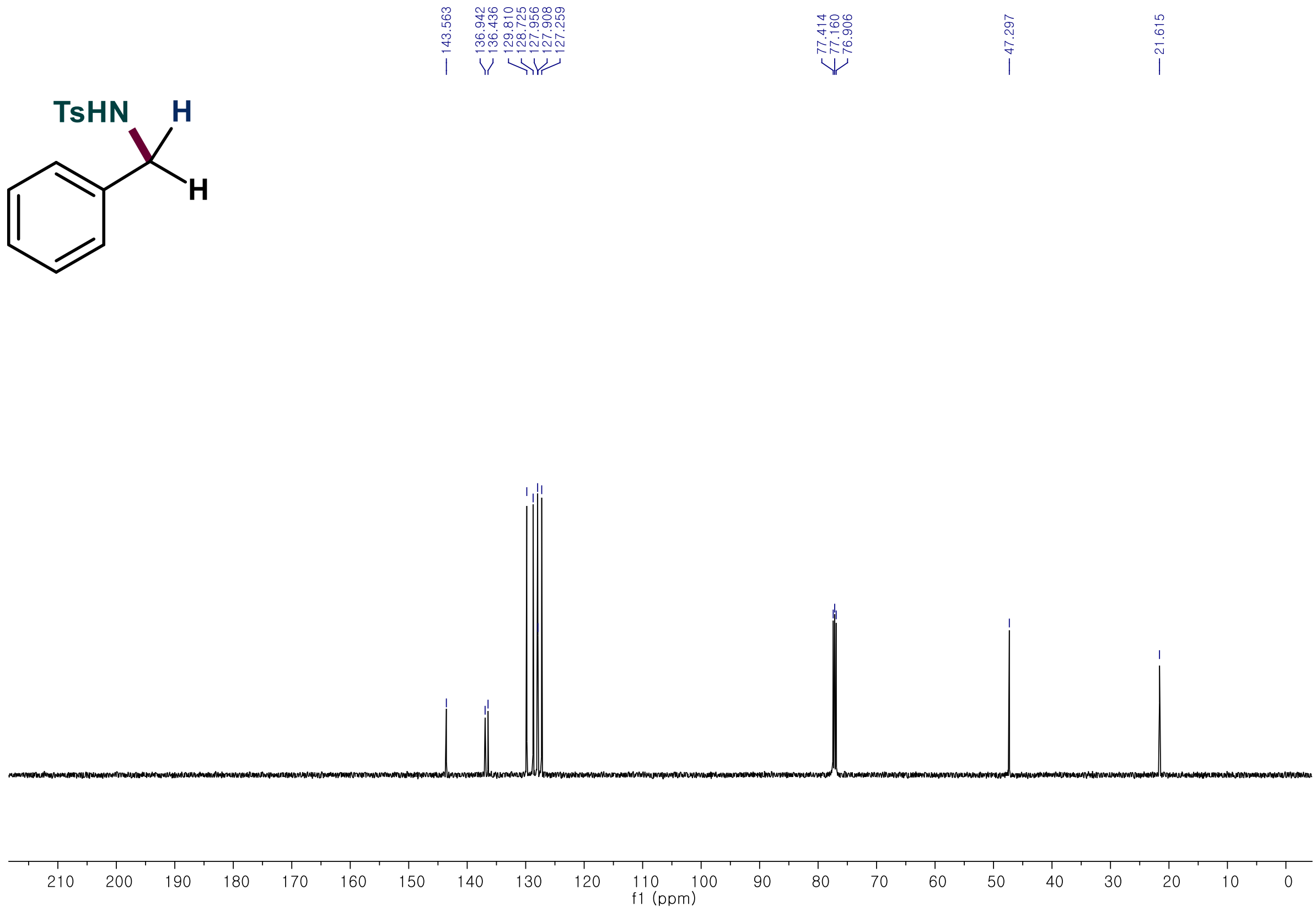

^{1}H NMR spectra (**54**)

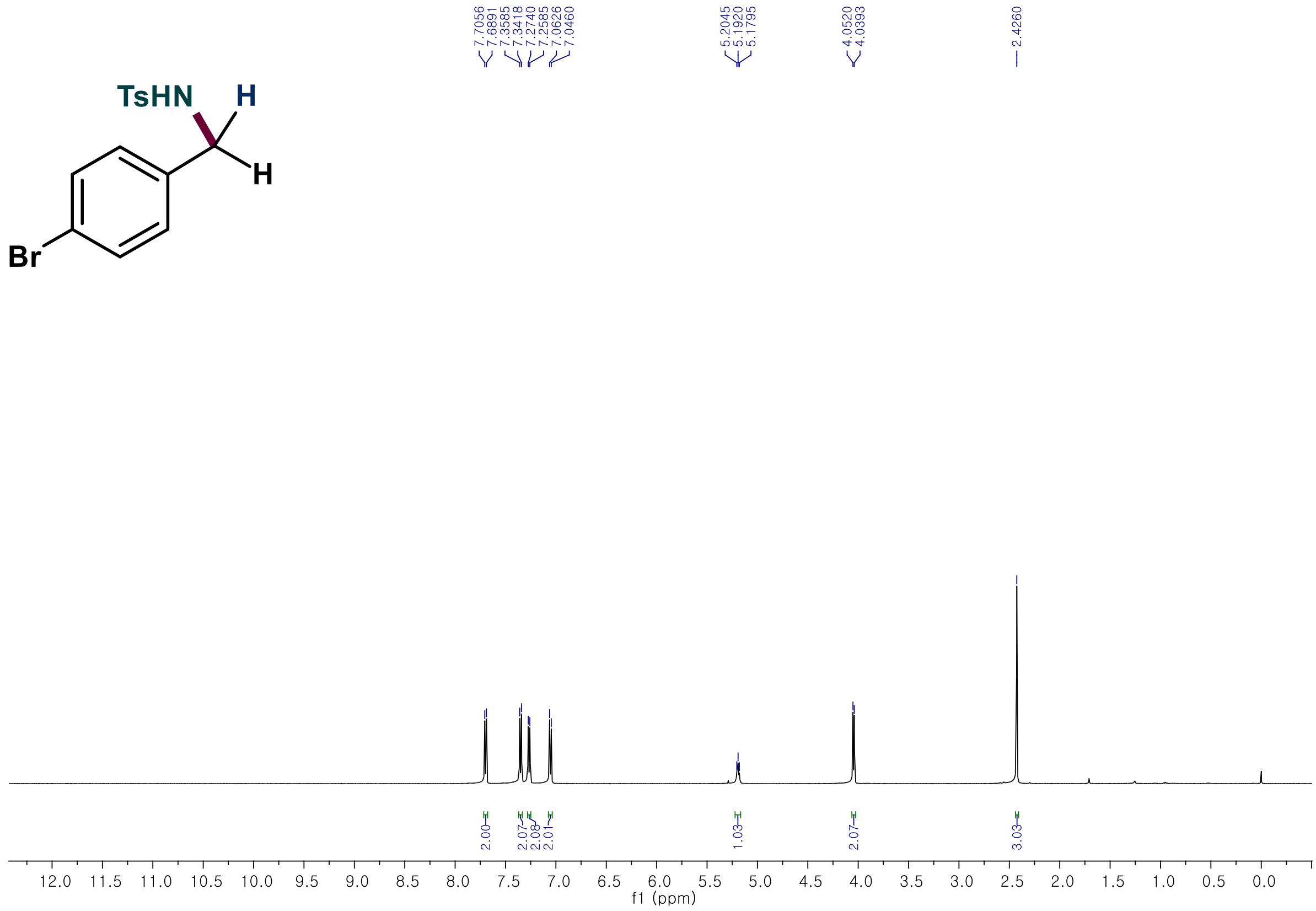

^{13}C NMR spectra (**54**)

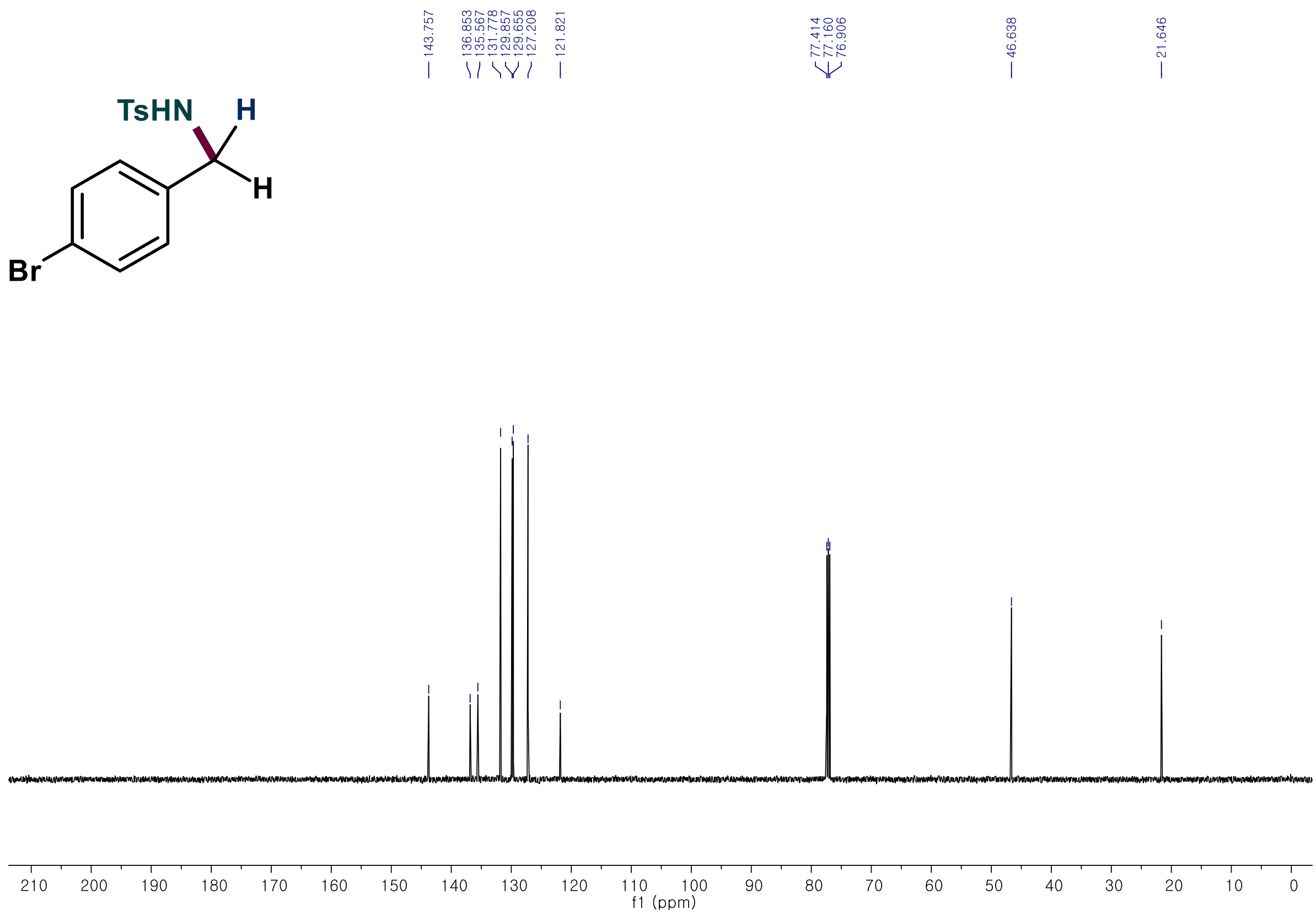

^{1}H NMR spectra (**55**)

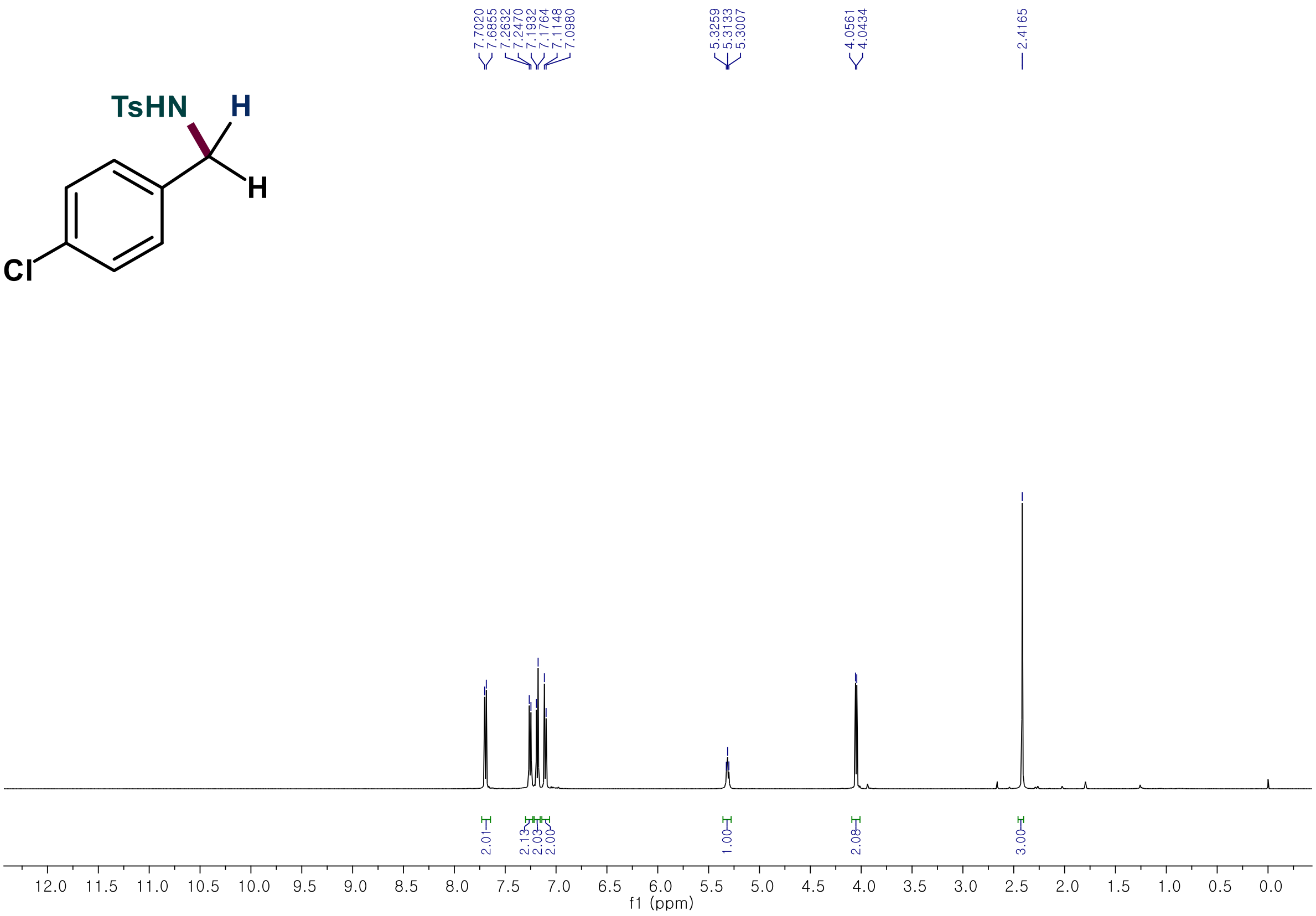

^{13}C NMR spectra (**55**)

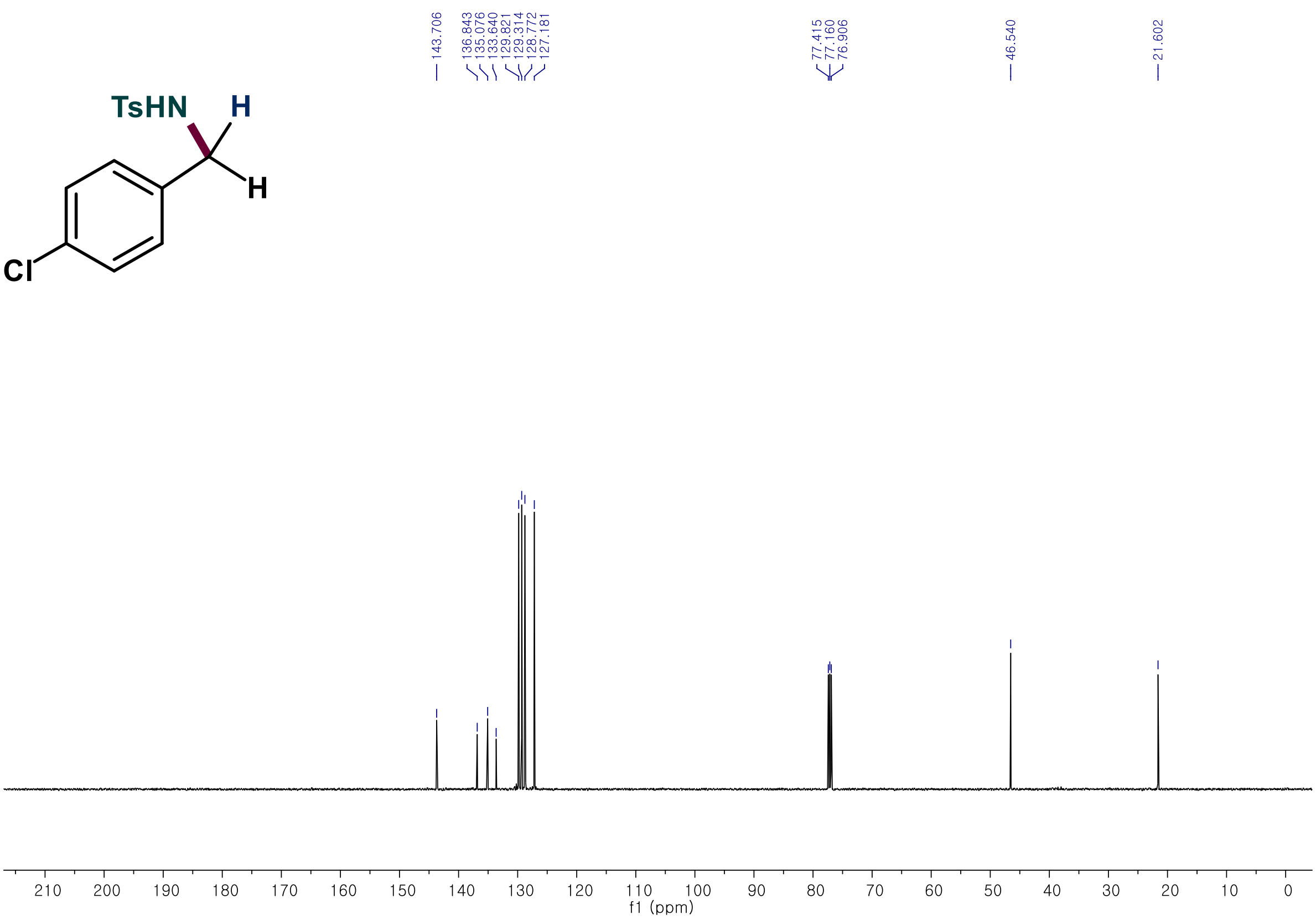

^{1}H NMR spectra (**56**)

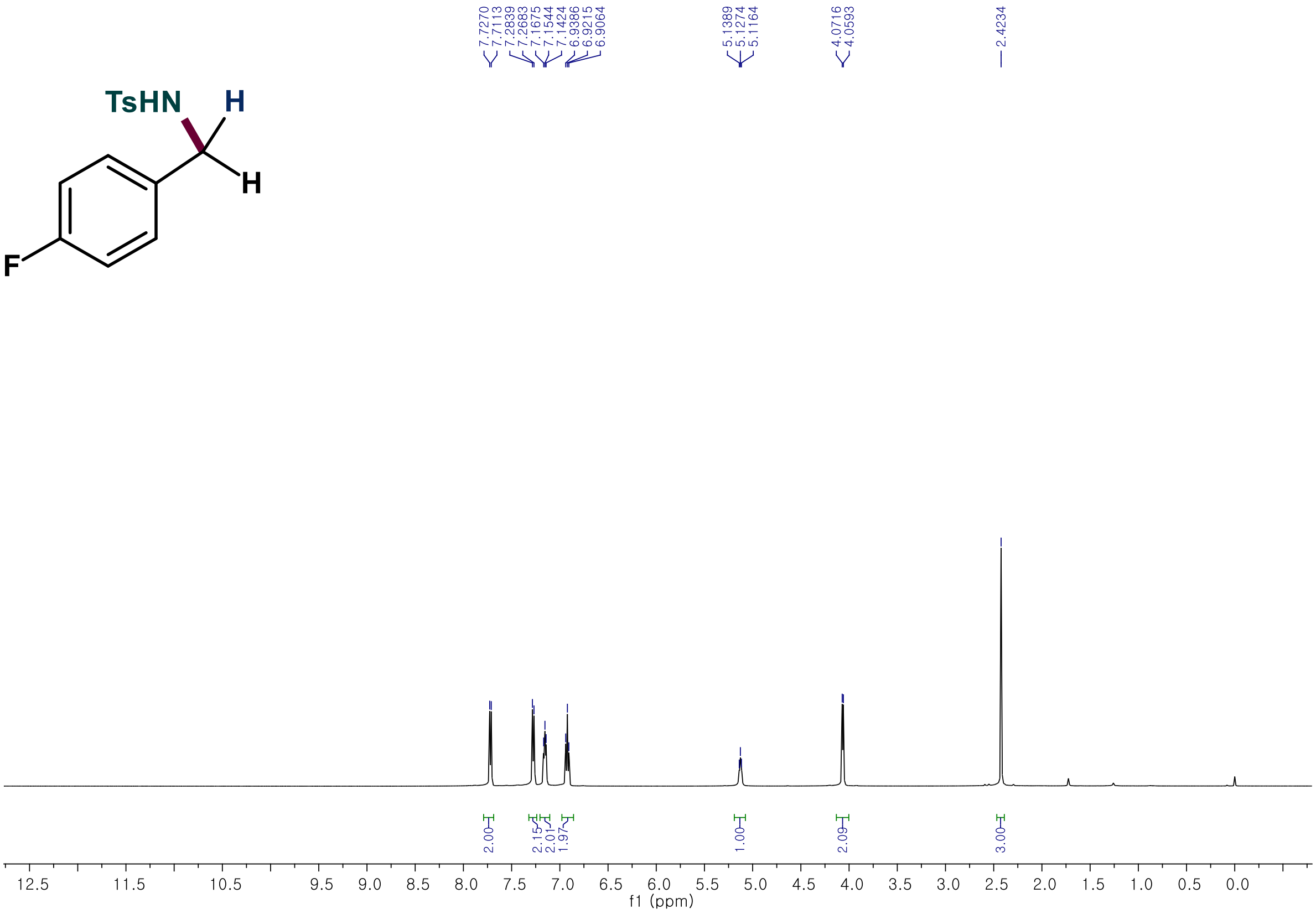

^{13}C NMR spectra (**56**)

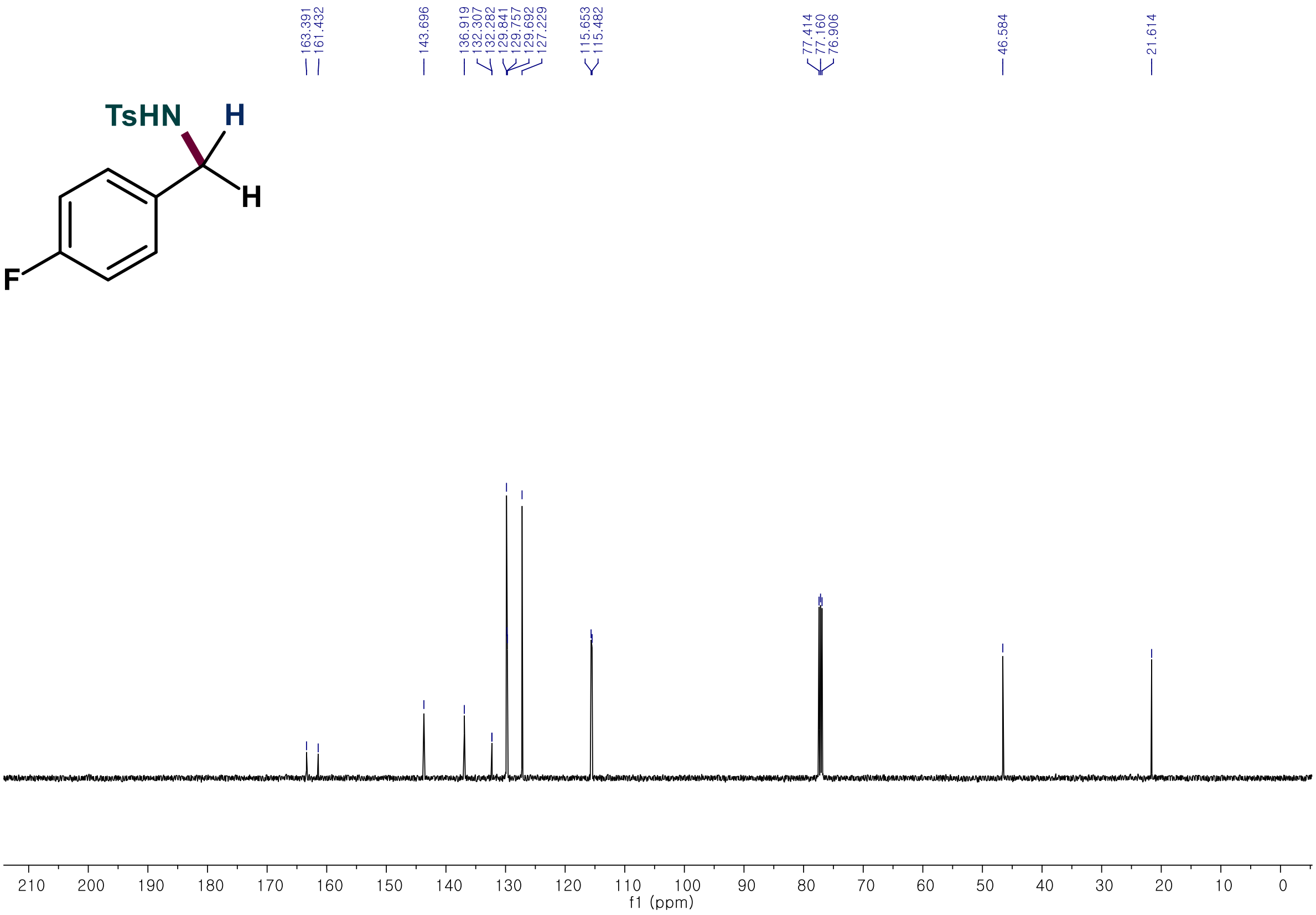

^{19}F NMR spectra (**56**)

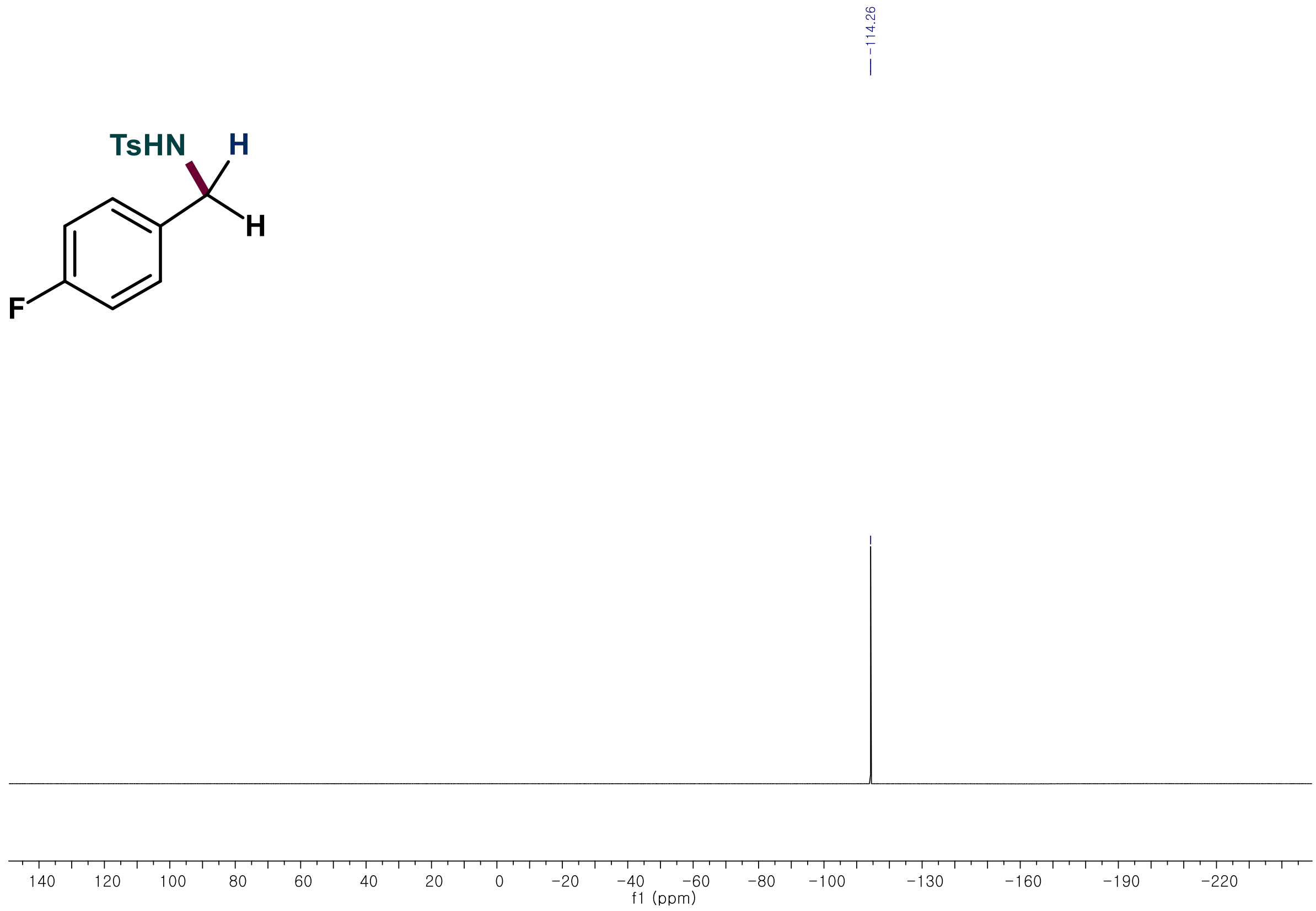

^{1}H NMR spectra (**57**)

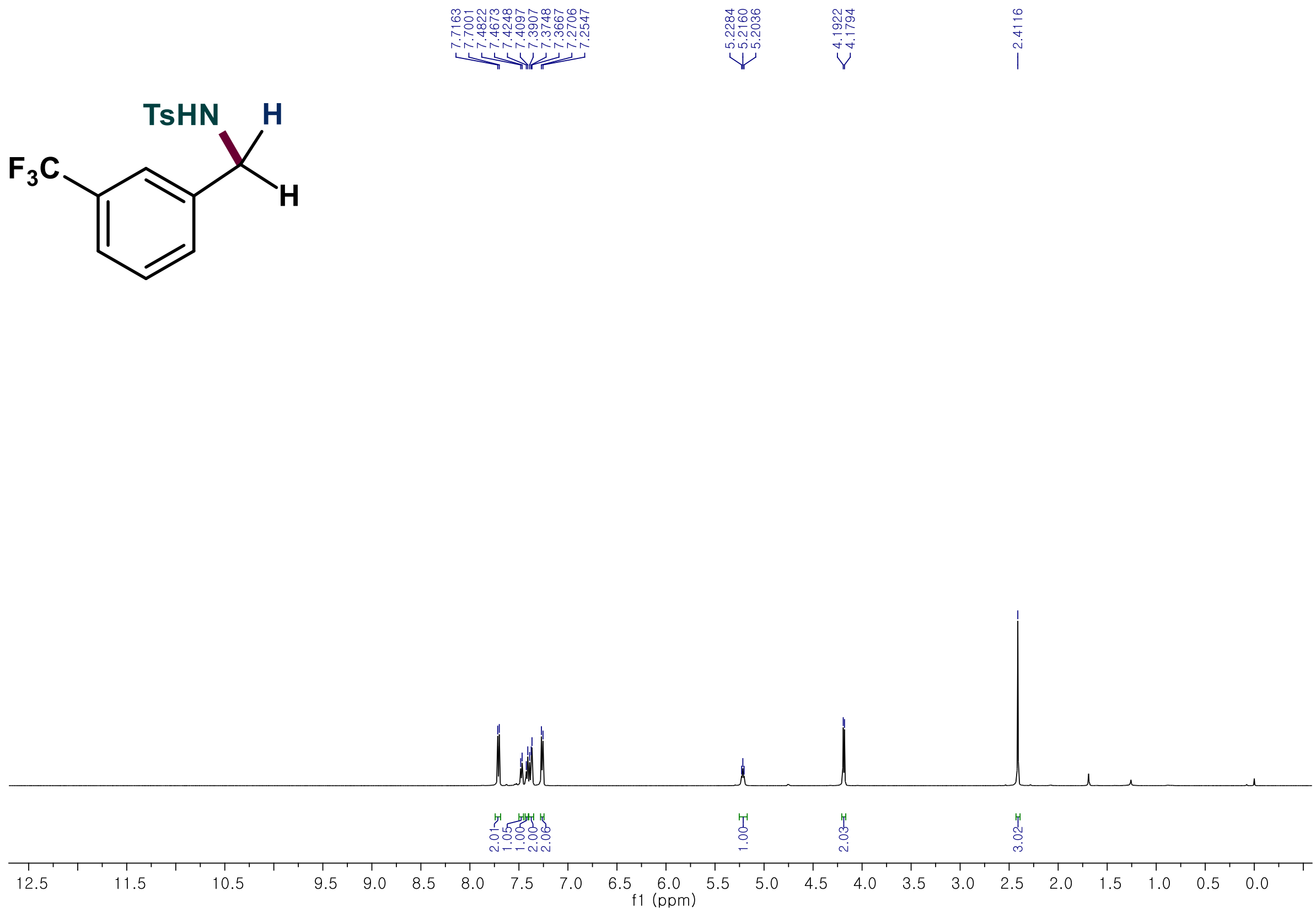

^{13}C NMR spectra (**57**)

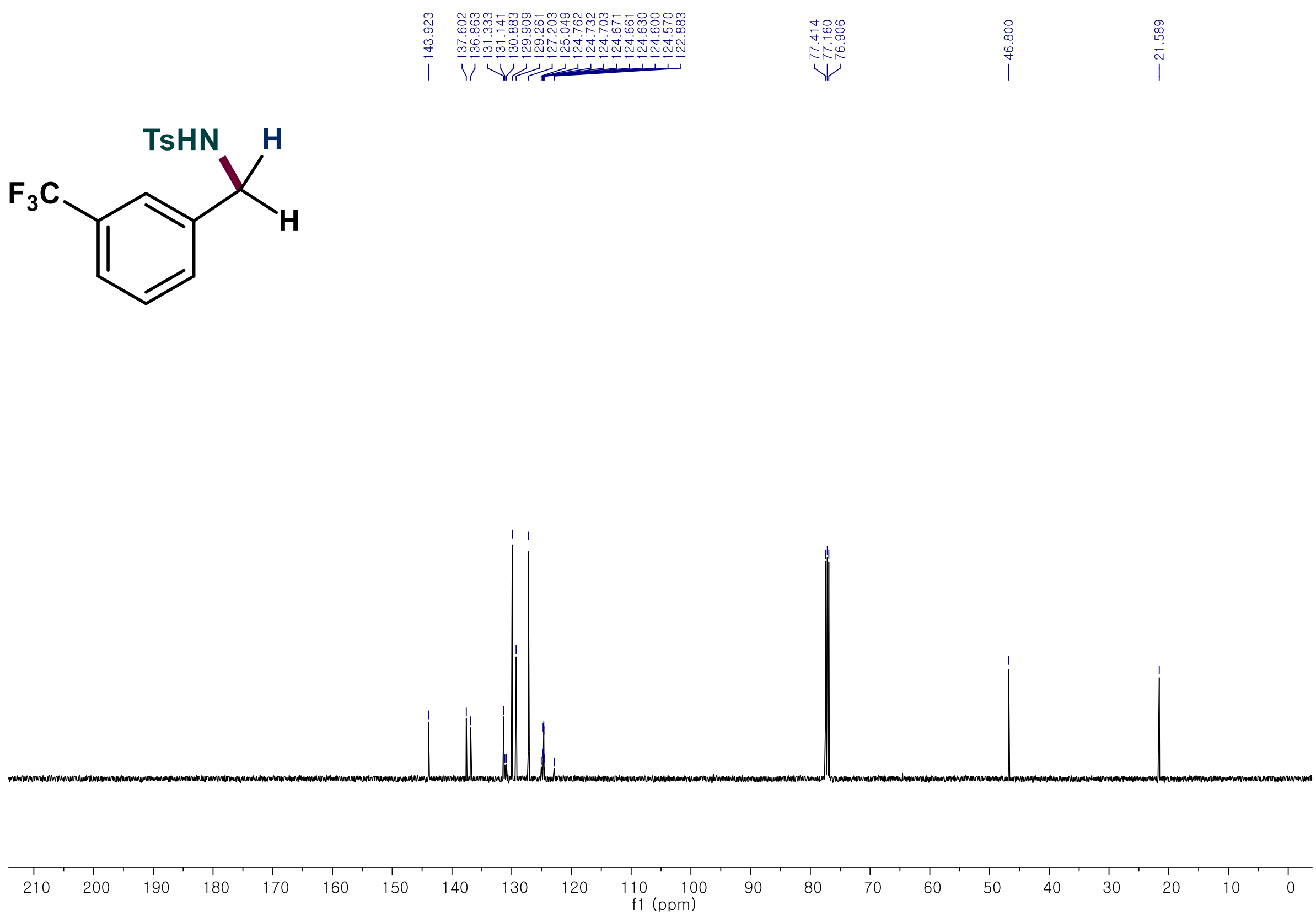

^{19}F NMR spectra (**57**)

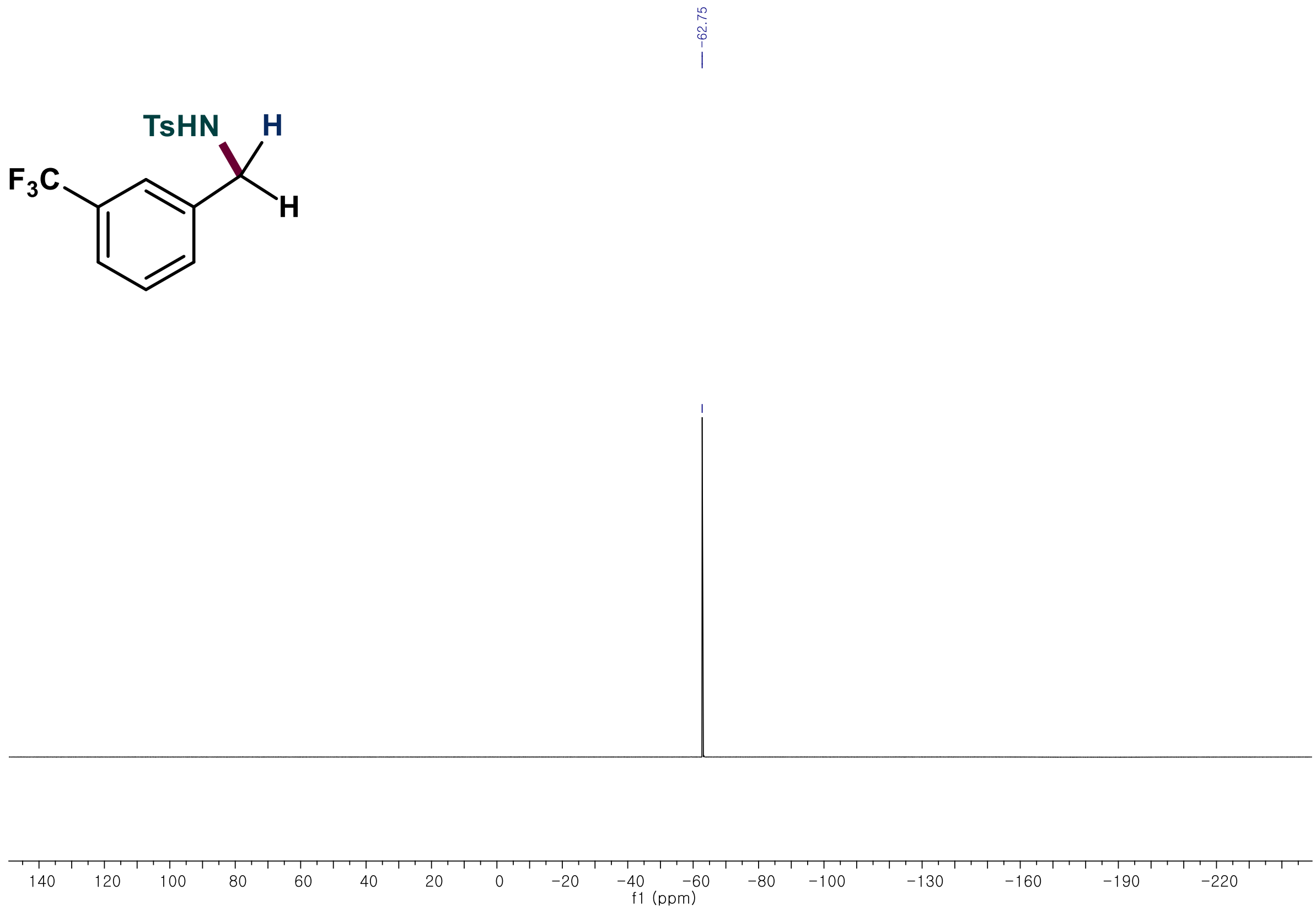

^{1}H NMR spectra (**58**)

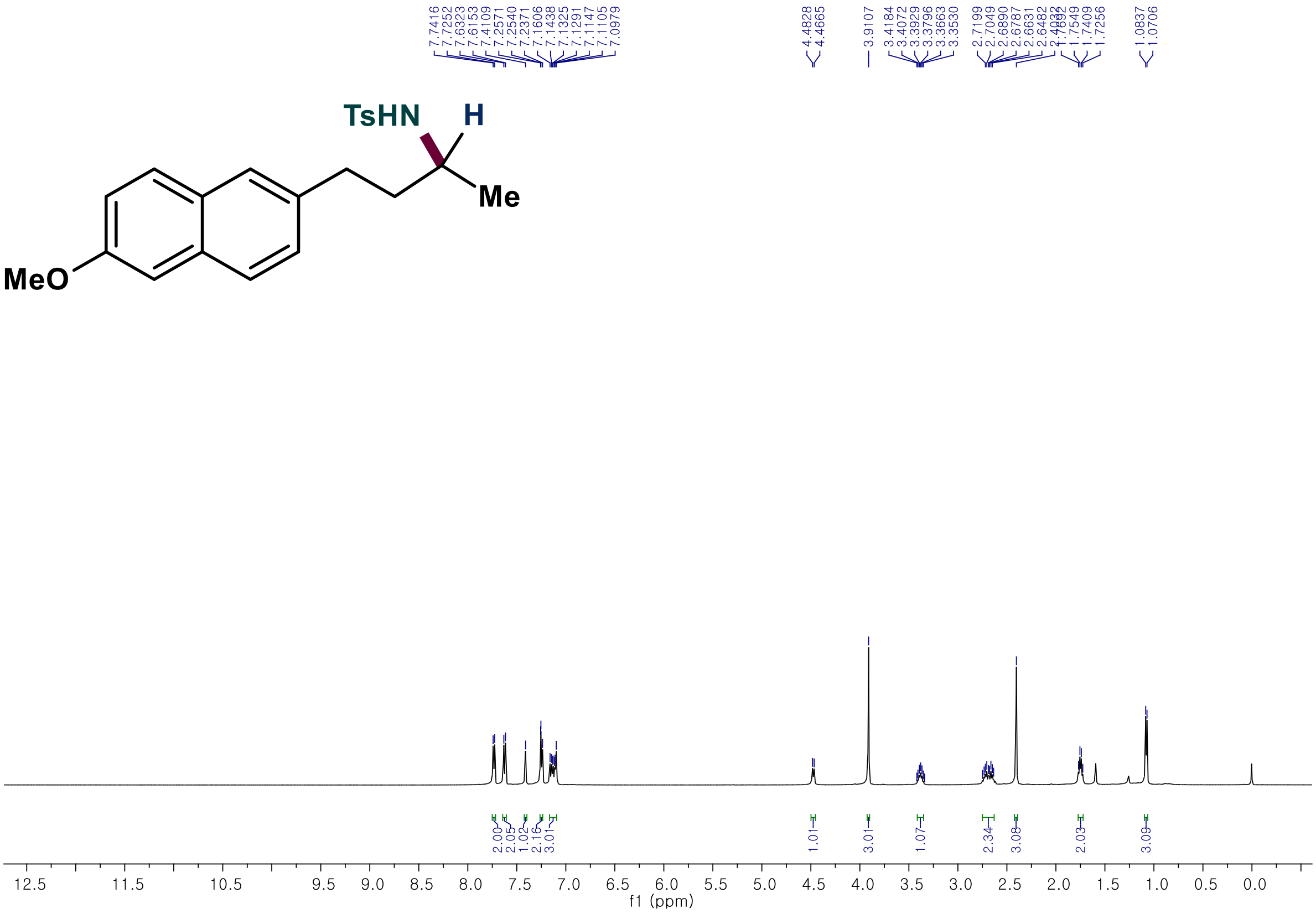

^{13}C NMR spectra (**58**)

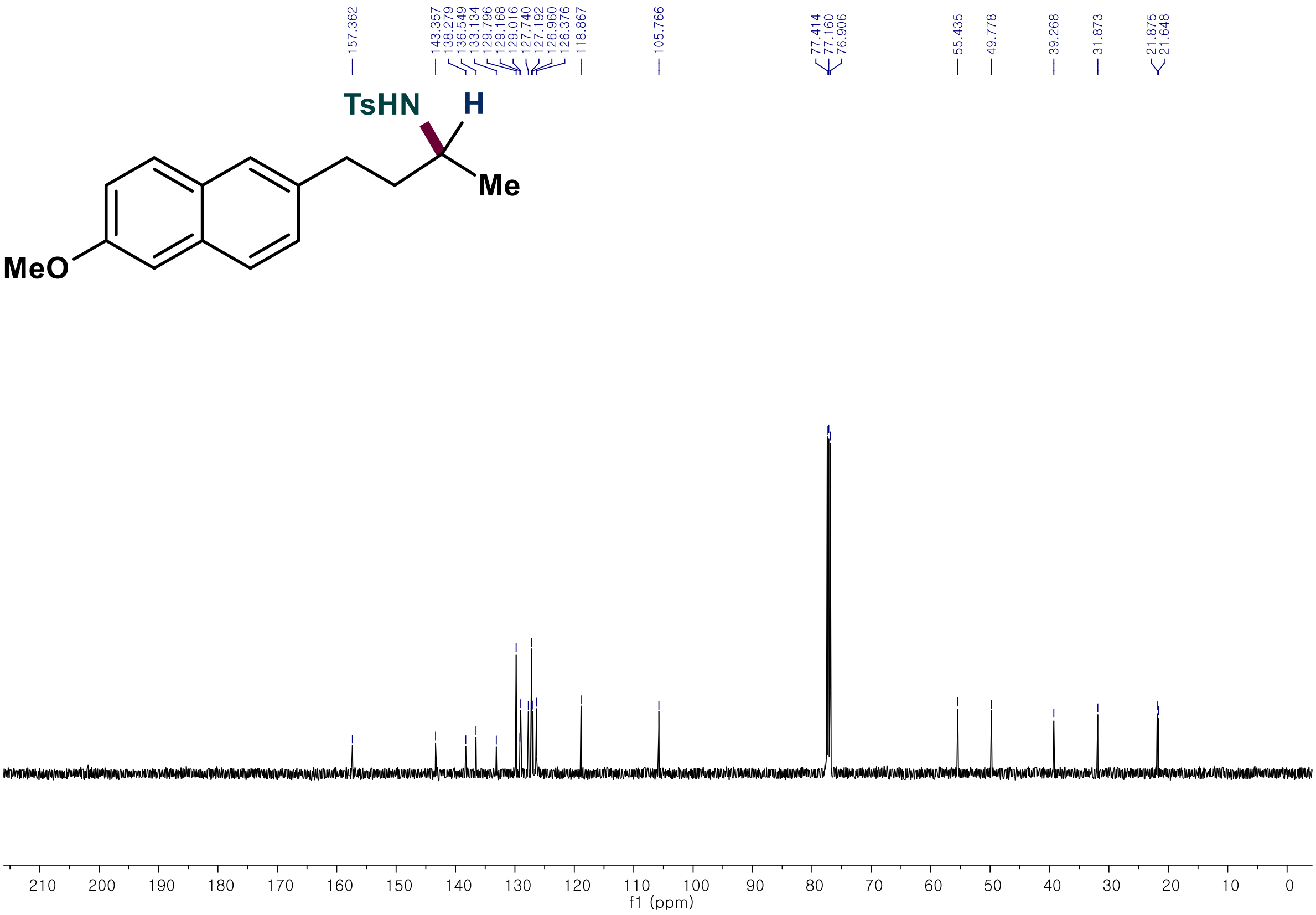

^{1}H NMR spectra (**59**)

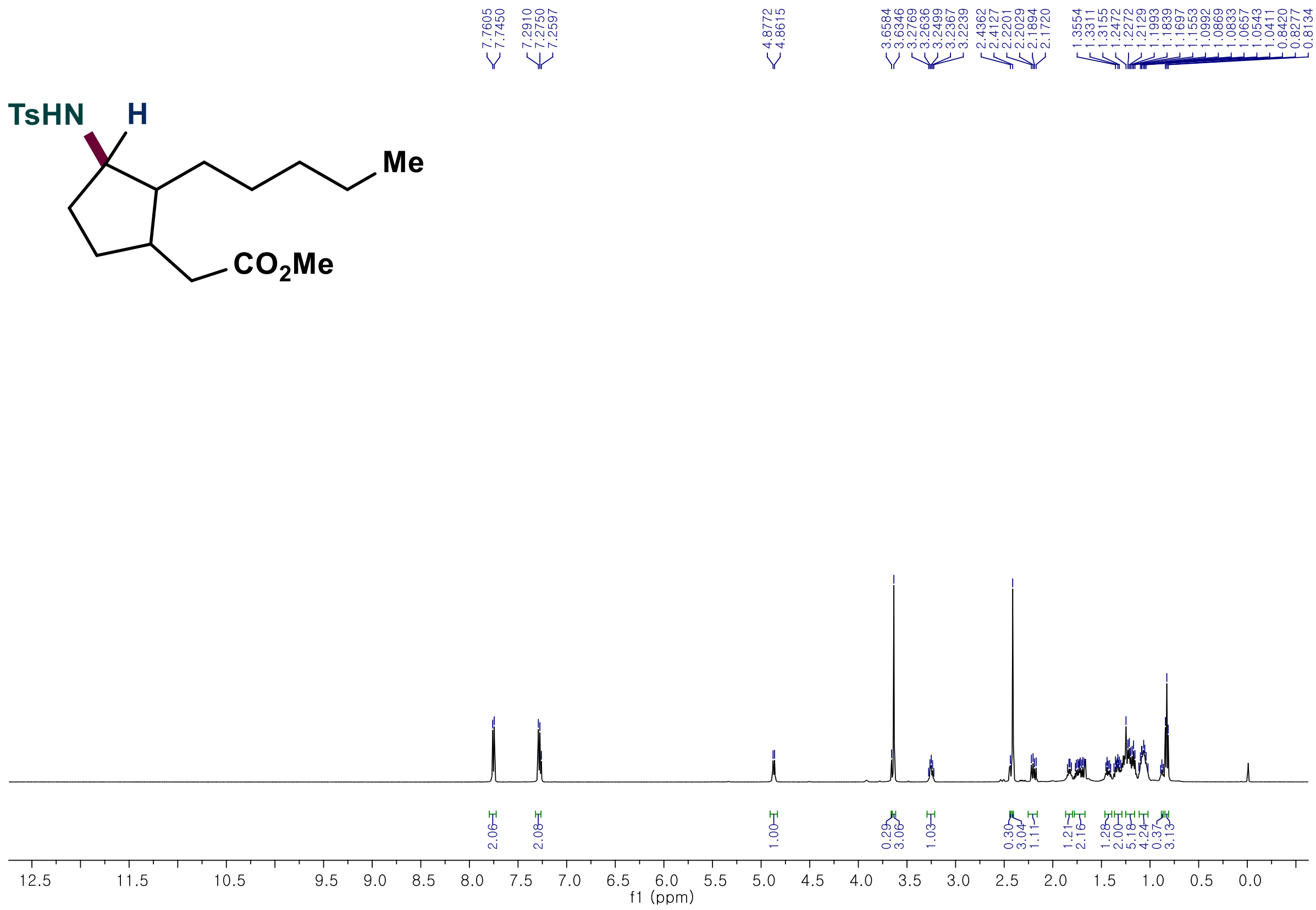

^{13}C NMR spectra (**59**)

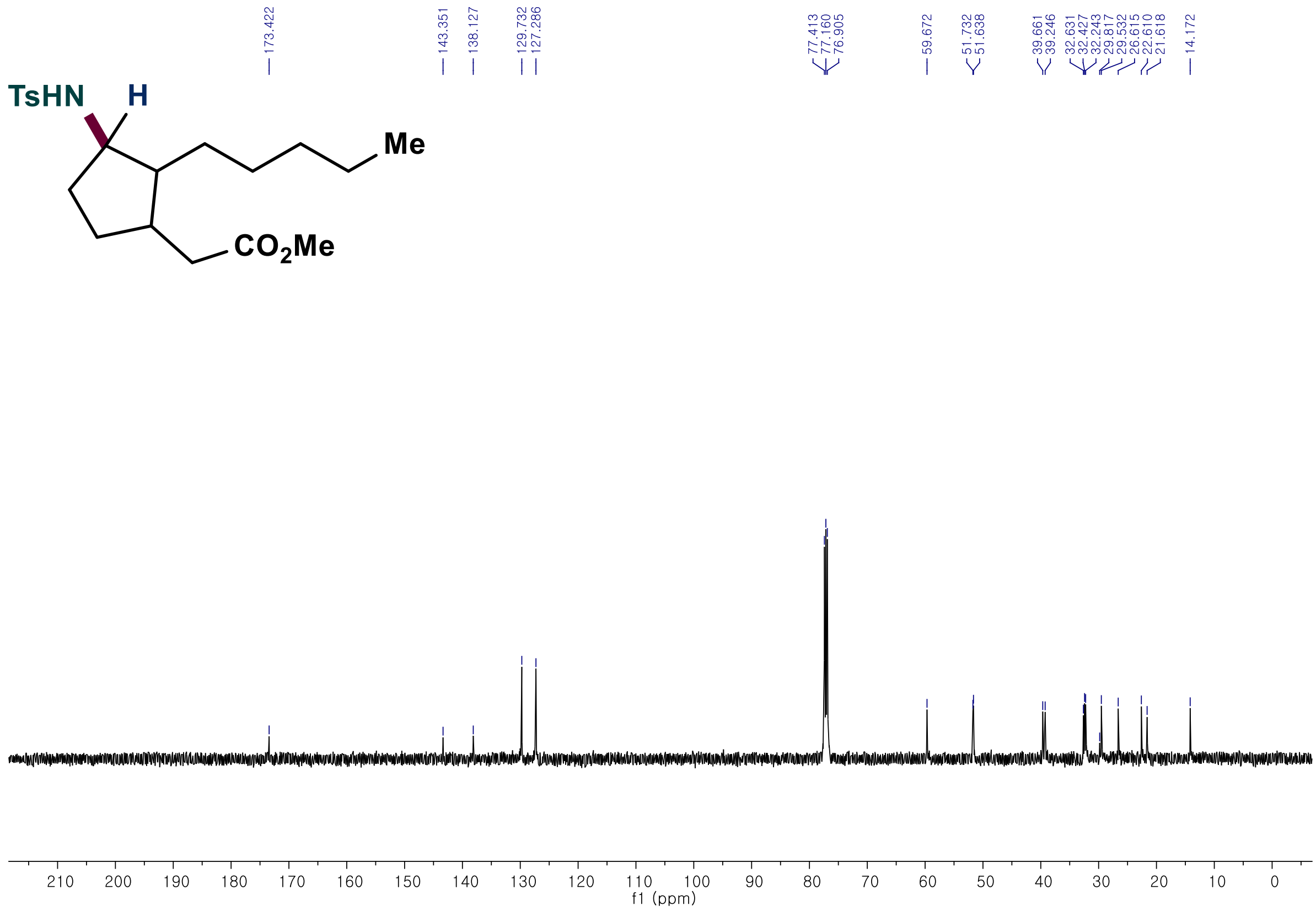

^{1}H NMR spectra (**60**)

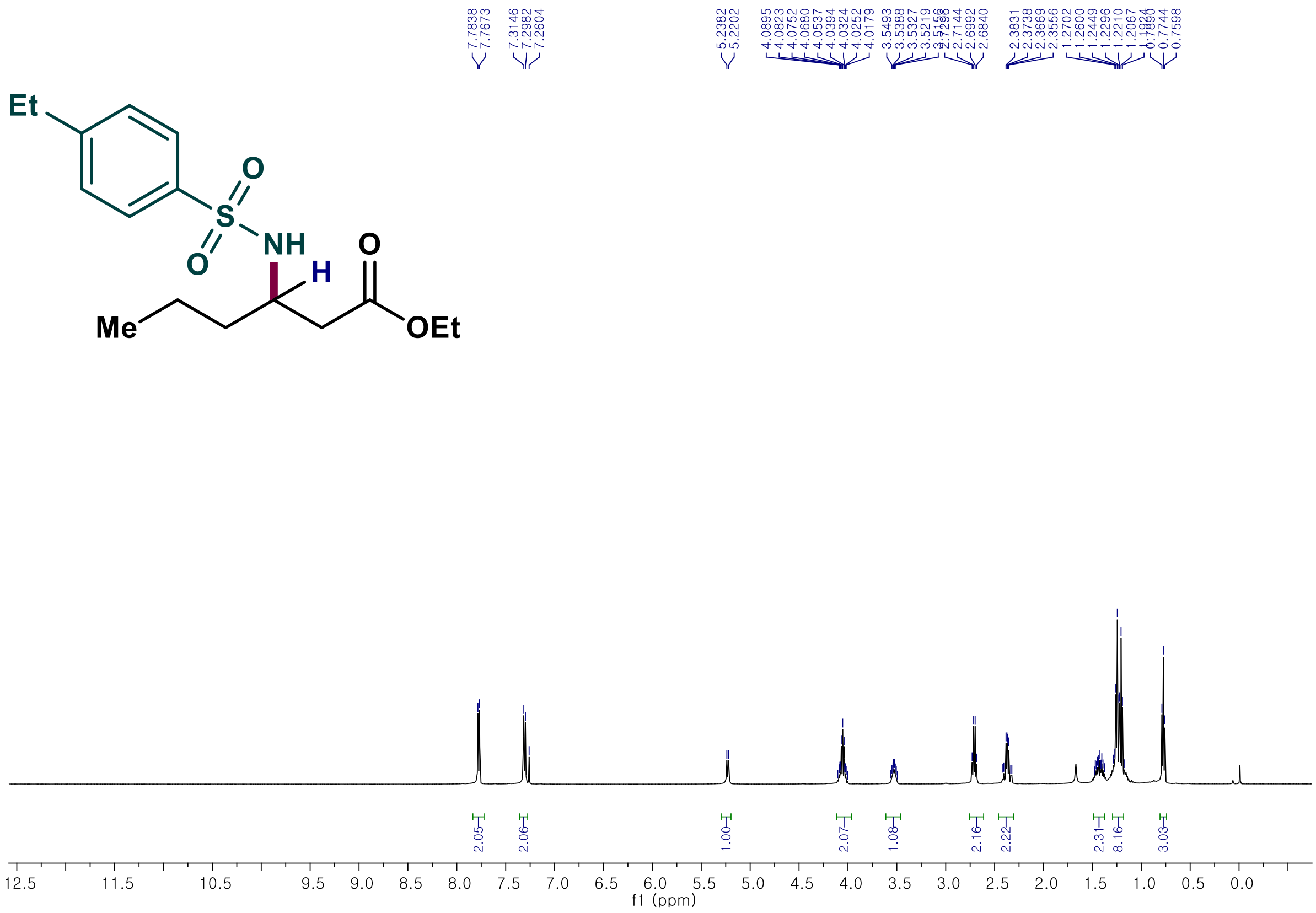

^{13}C NMR spectra (**60**)

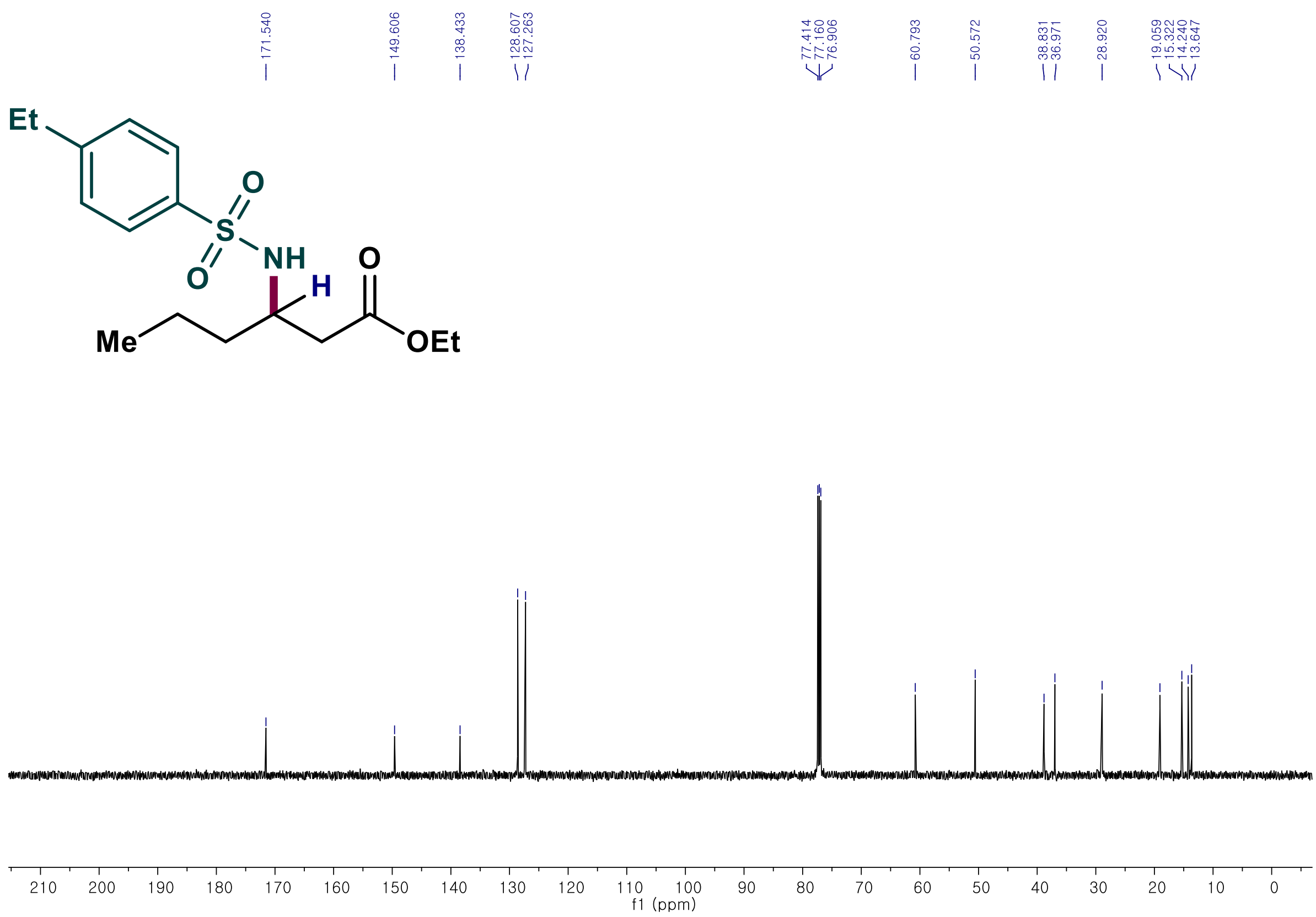

^{1}H NMR spectra (**61**)

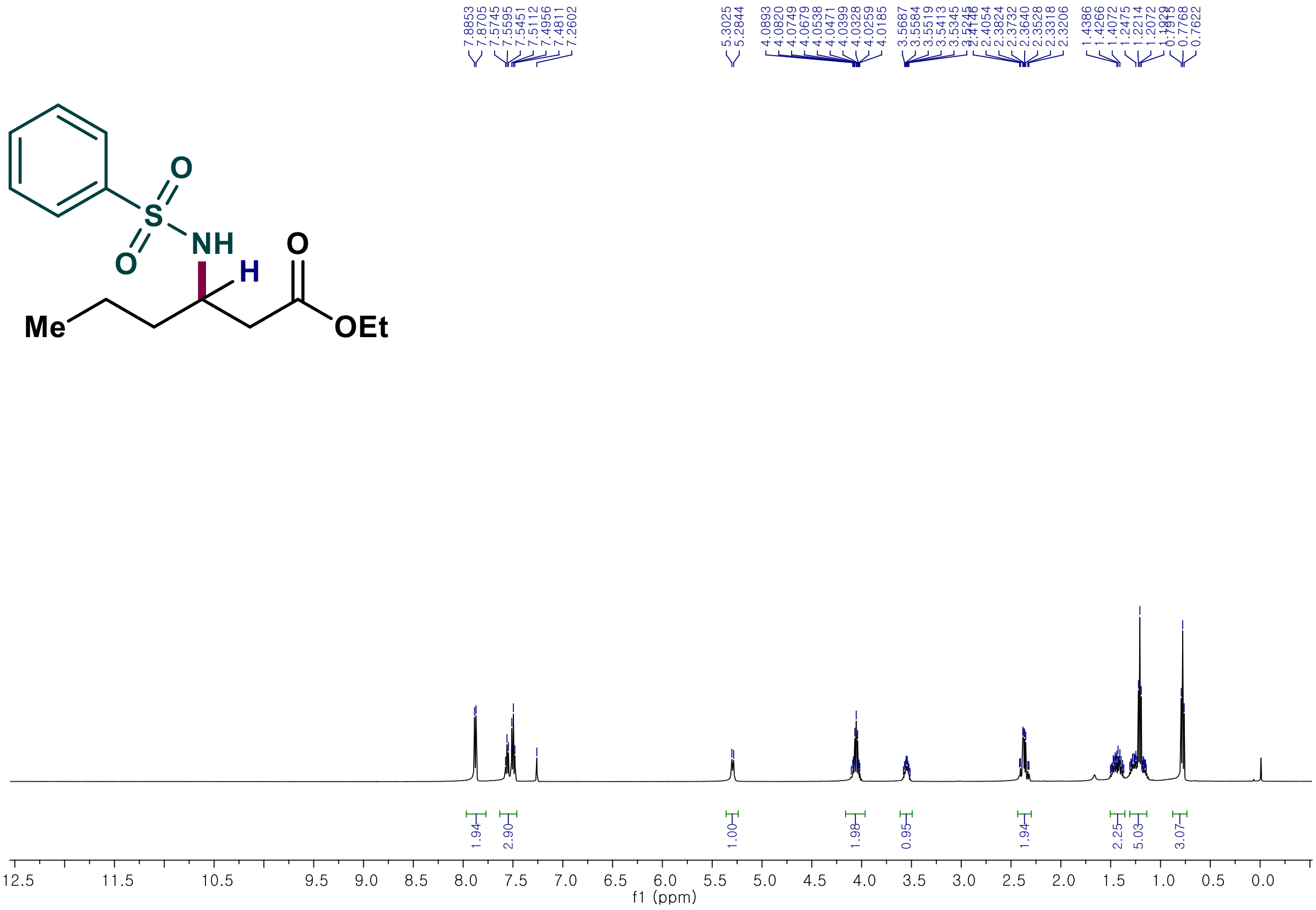

^{13}C NMR spectra (**61**)

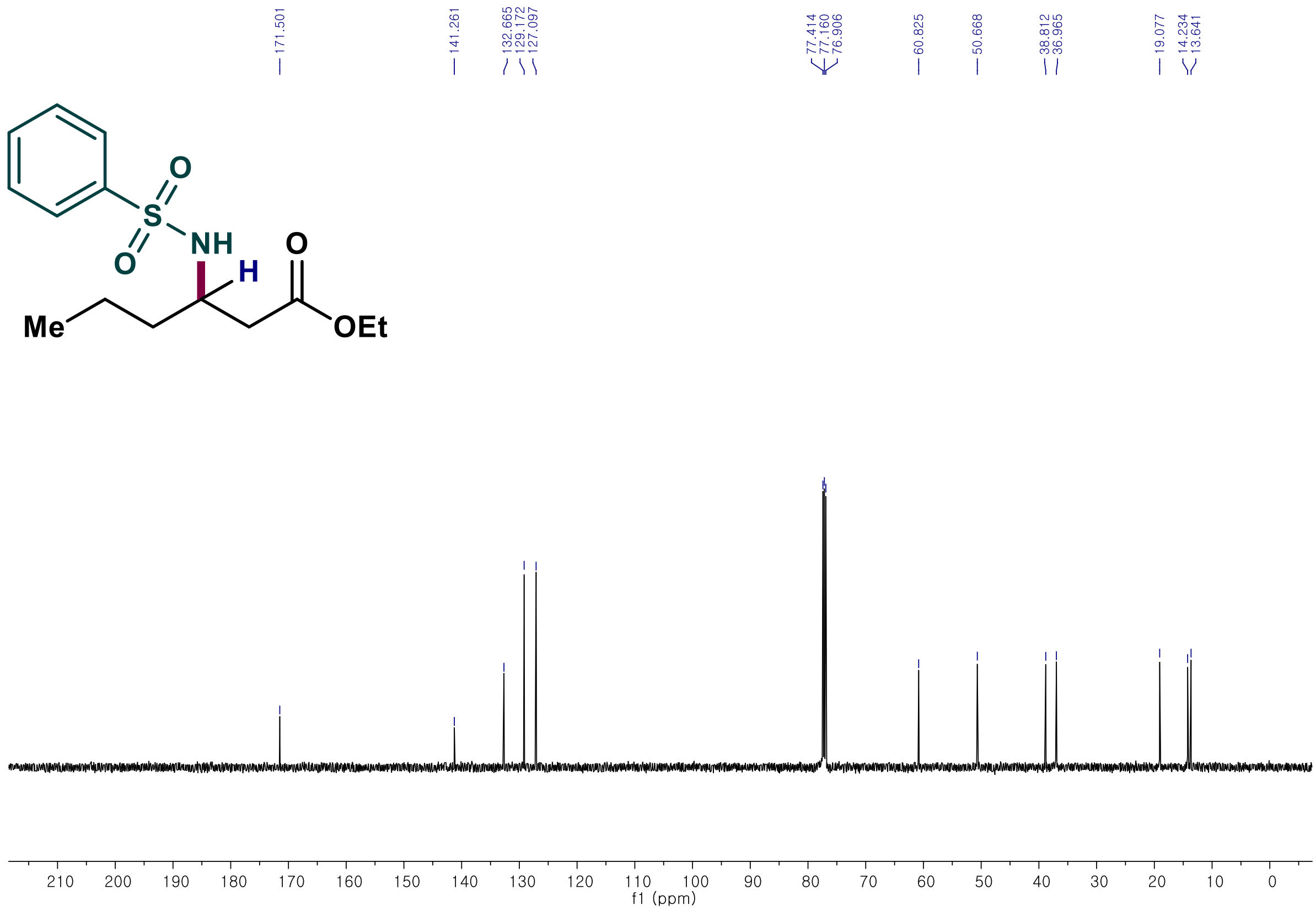

^{1}H NMR spectra (**62**)

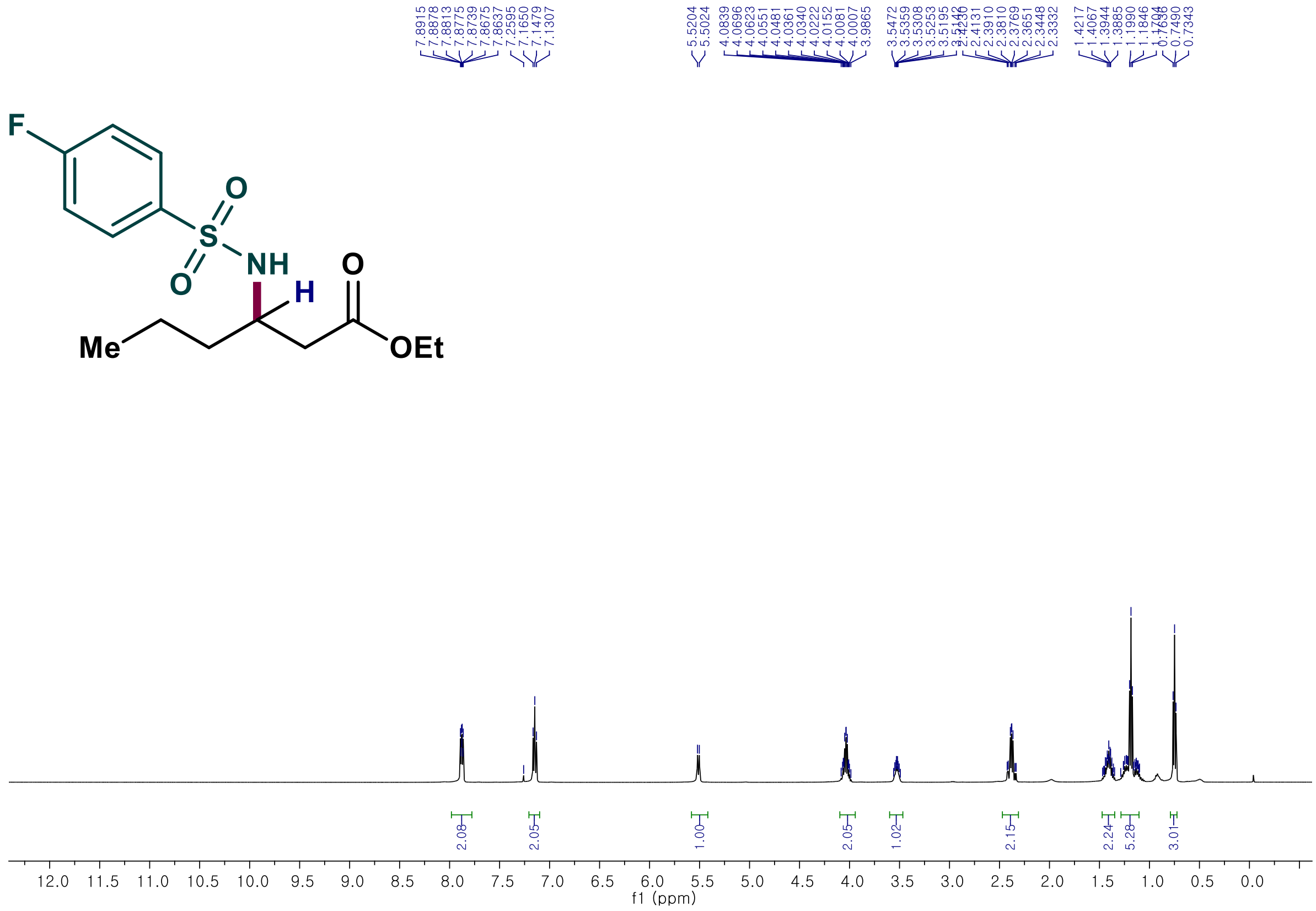

^{13}C NMR spectra (**62**)

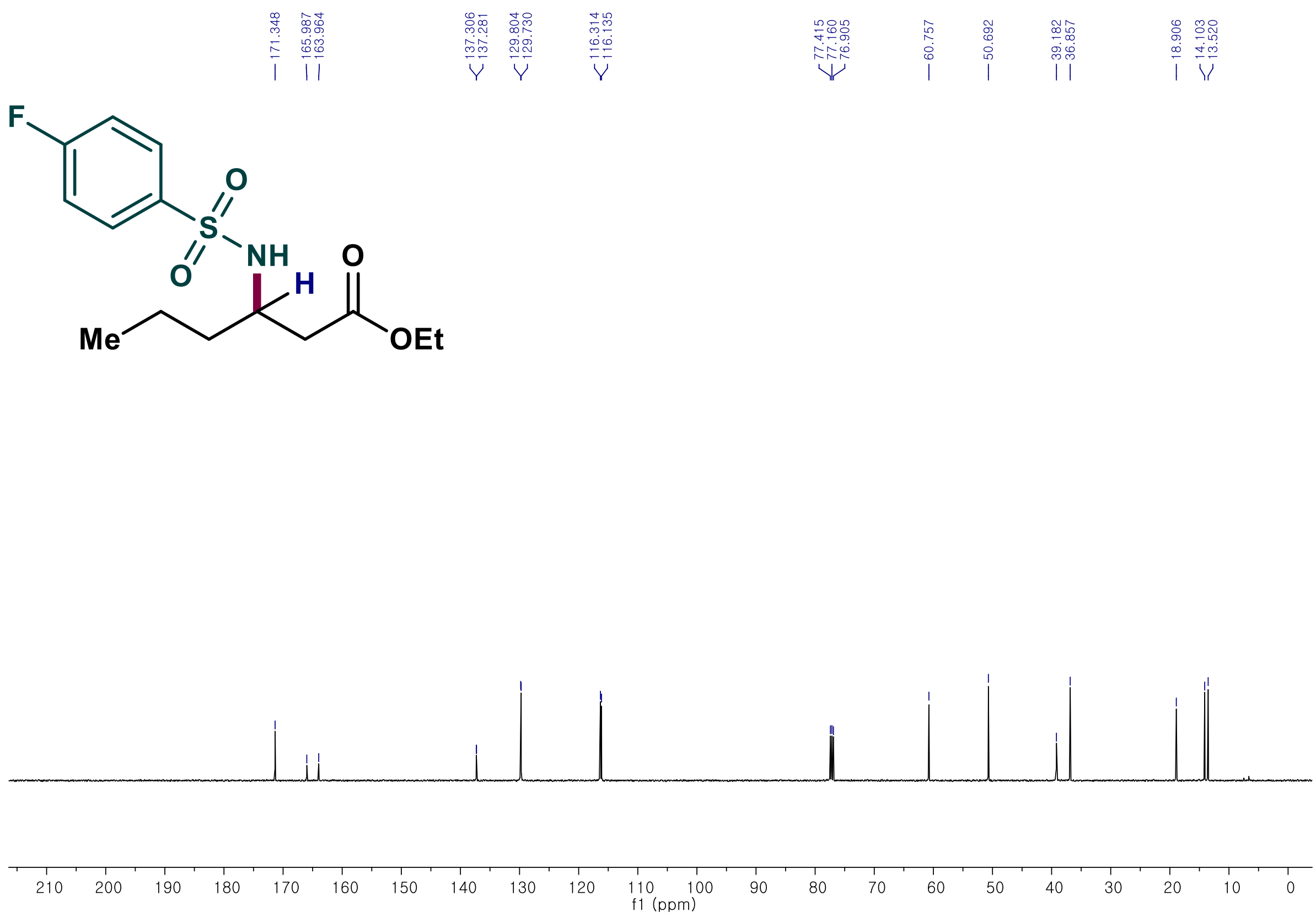

^{19}F NMR spectra (**62**)

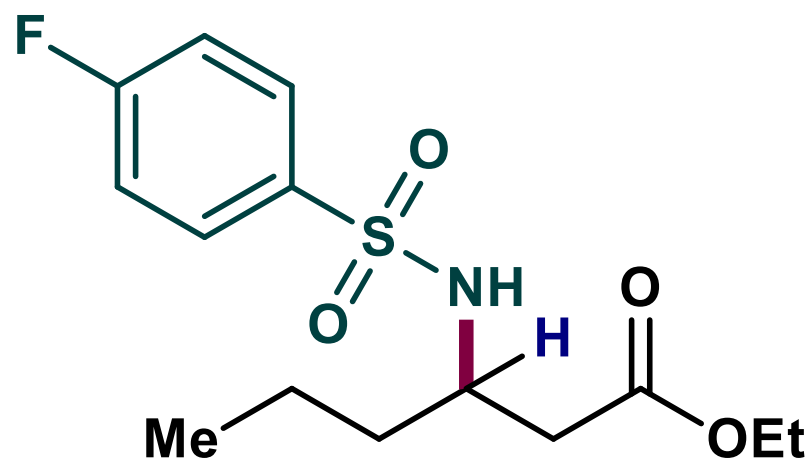

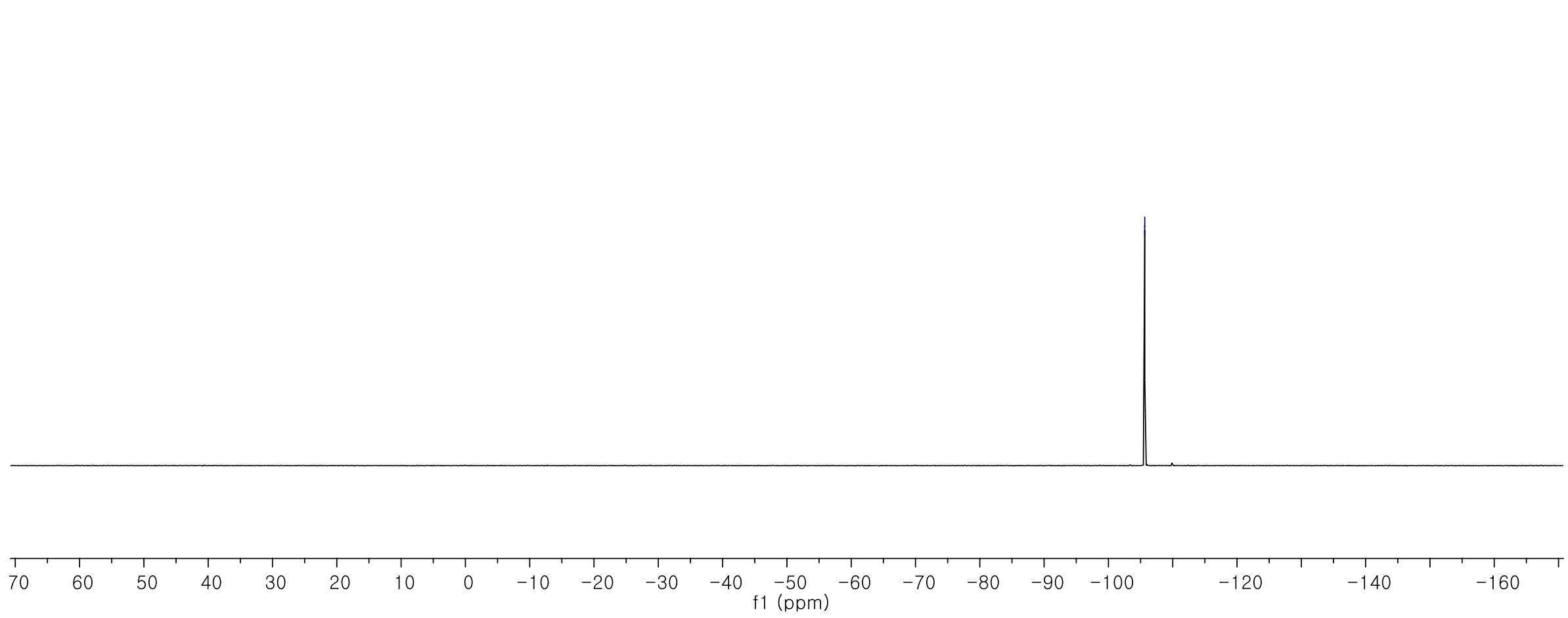

^{1}H NMR spectra (**63**)

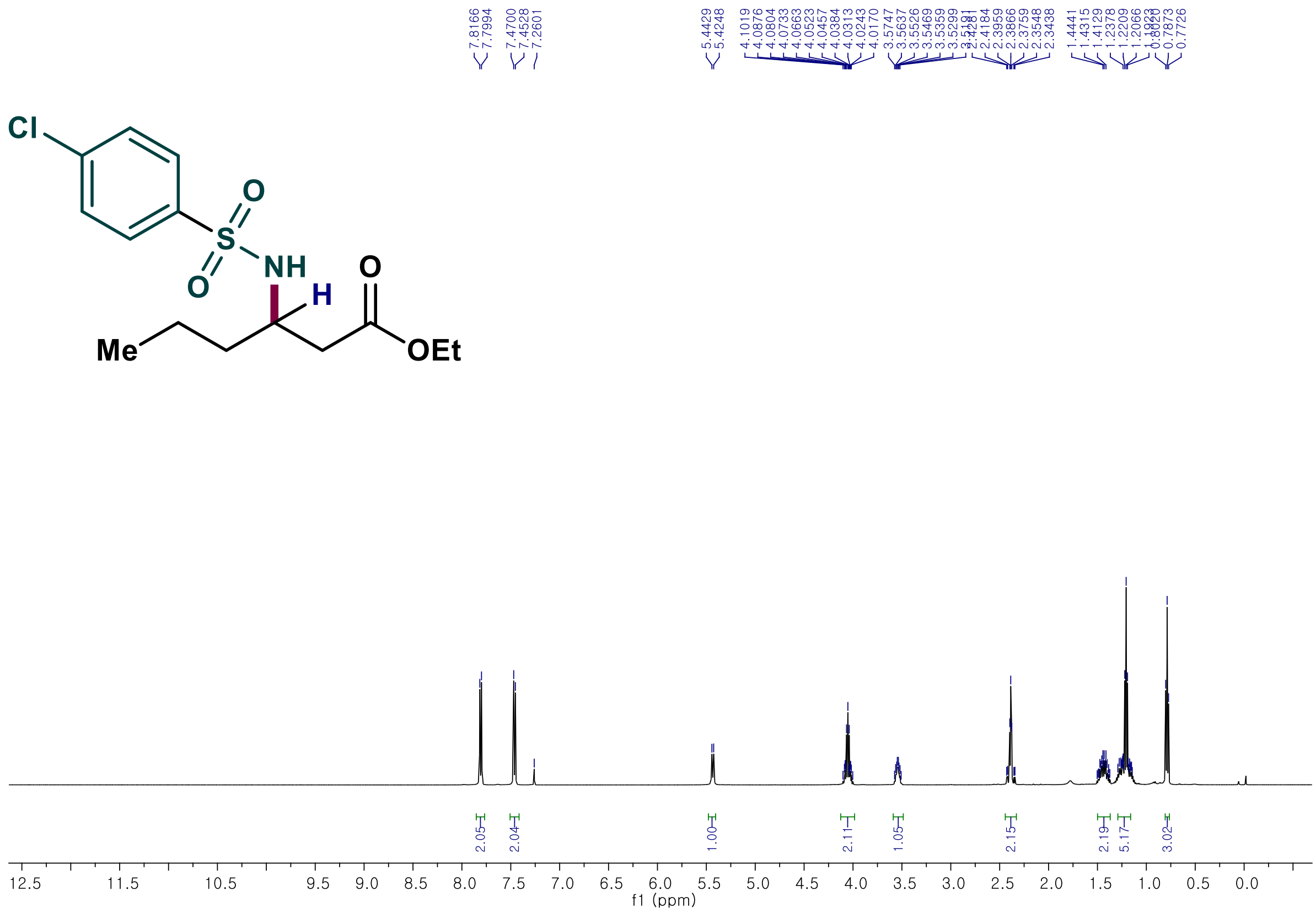

^{13}C NMR spectra (**63**)

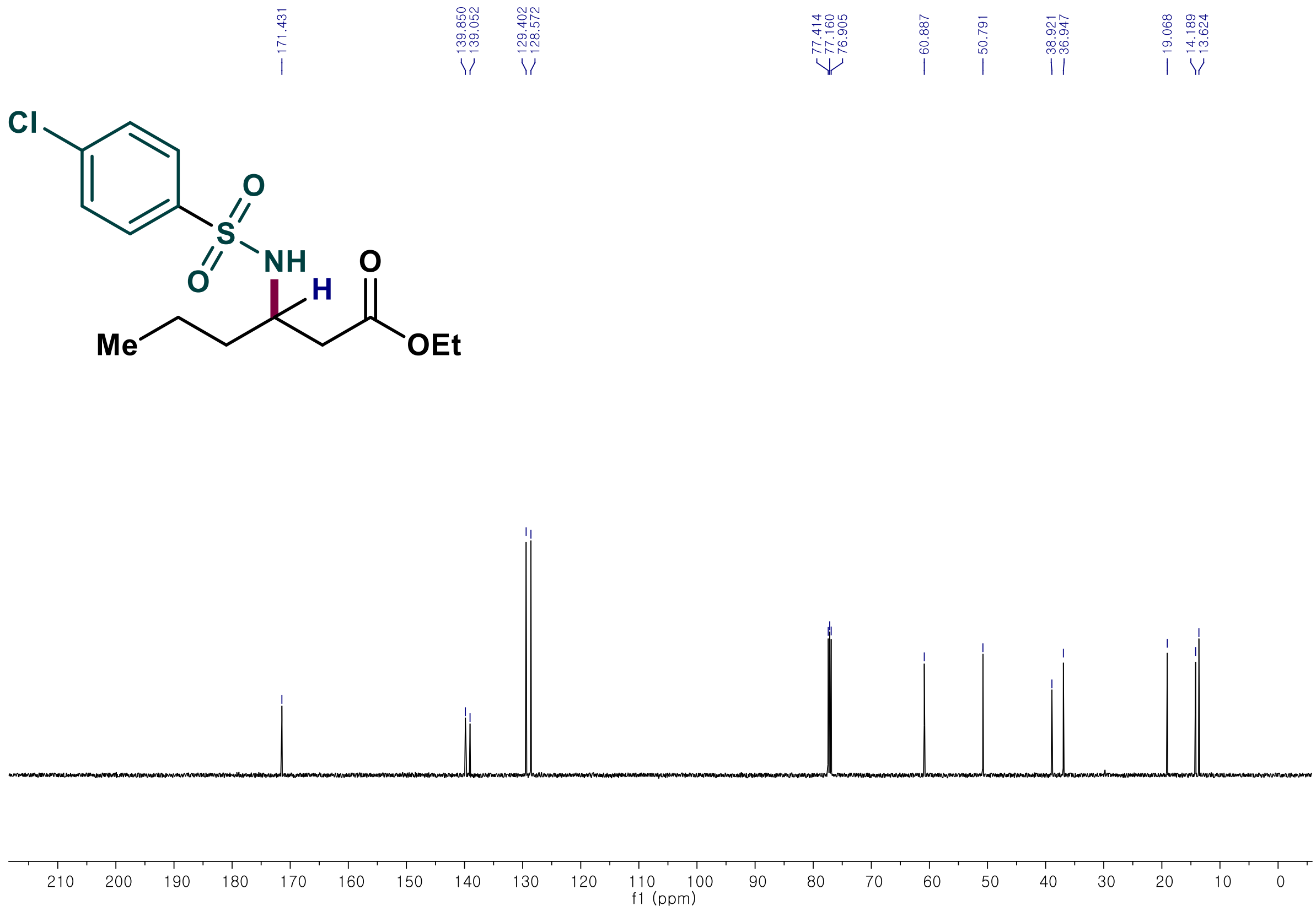

^{1}H NMR spectra (**64**)

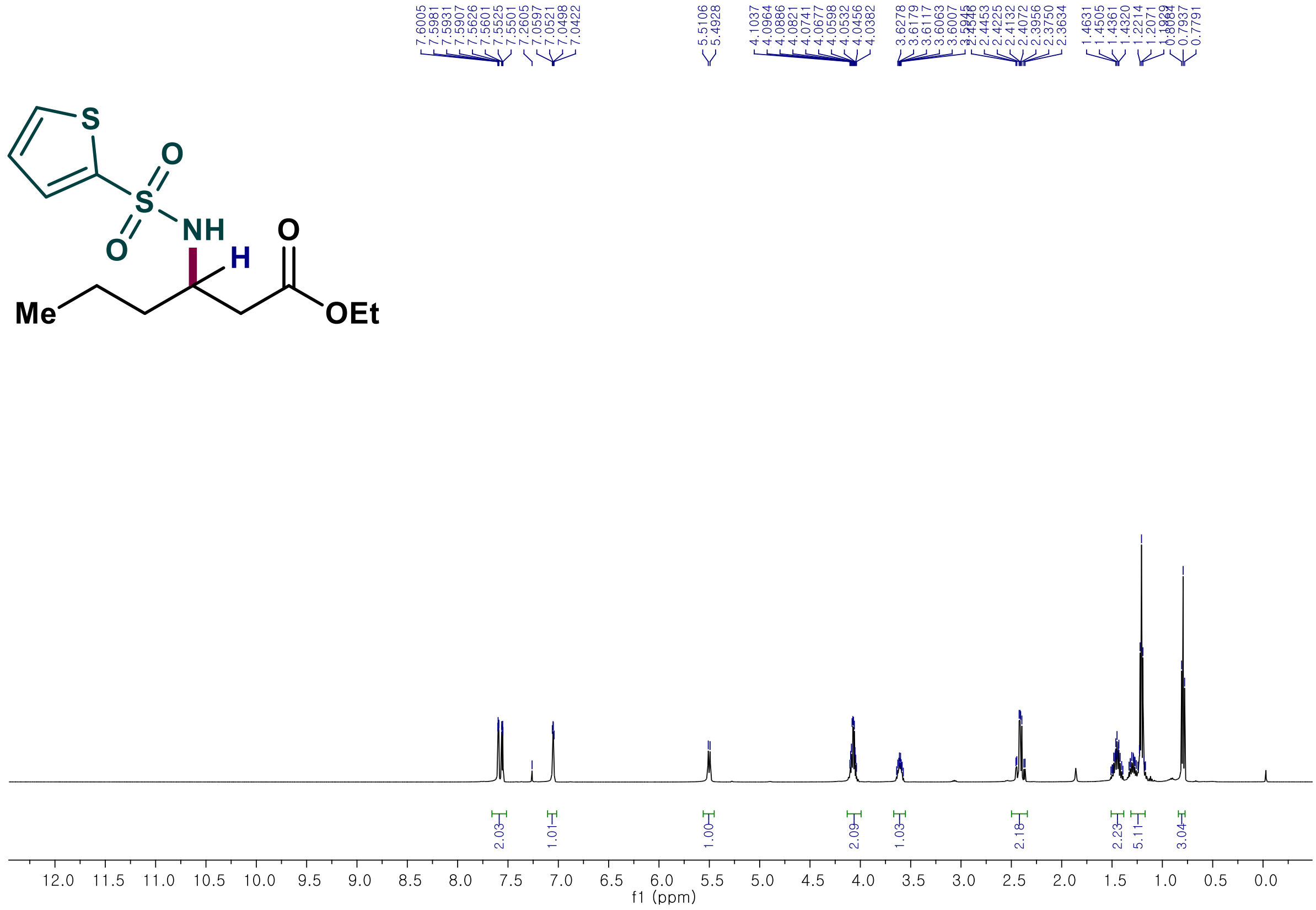

^{13}C NMR spectra (**64**)

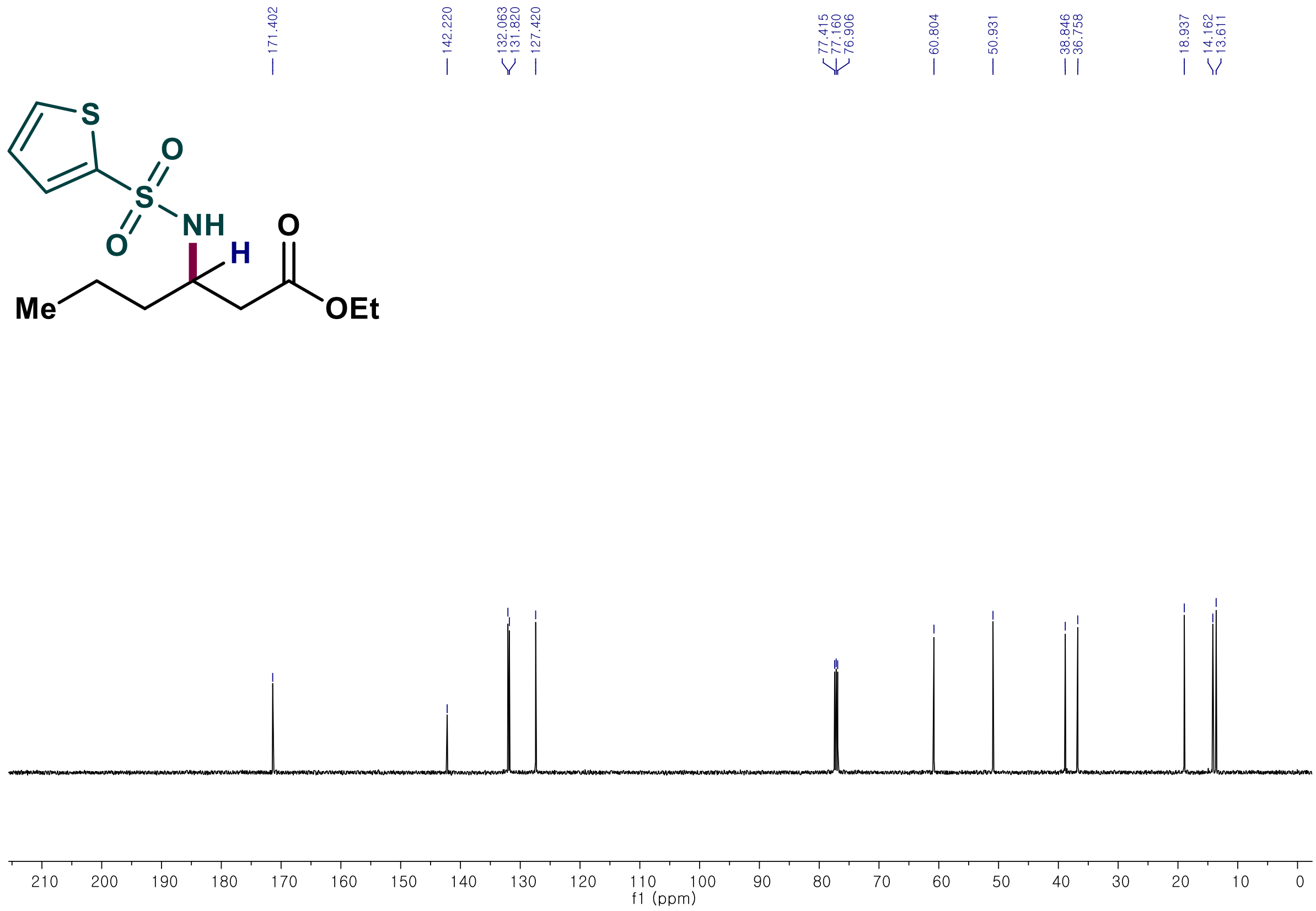

^{1}H NMR spectra (**65**)

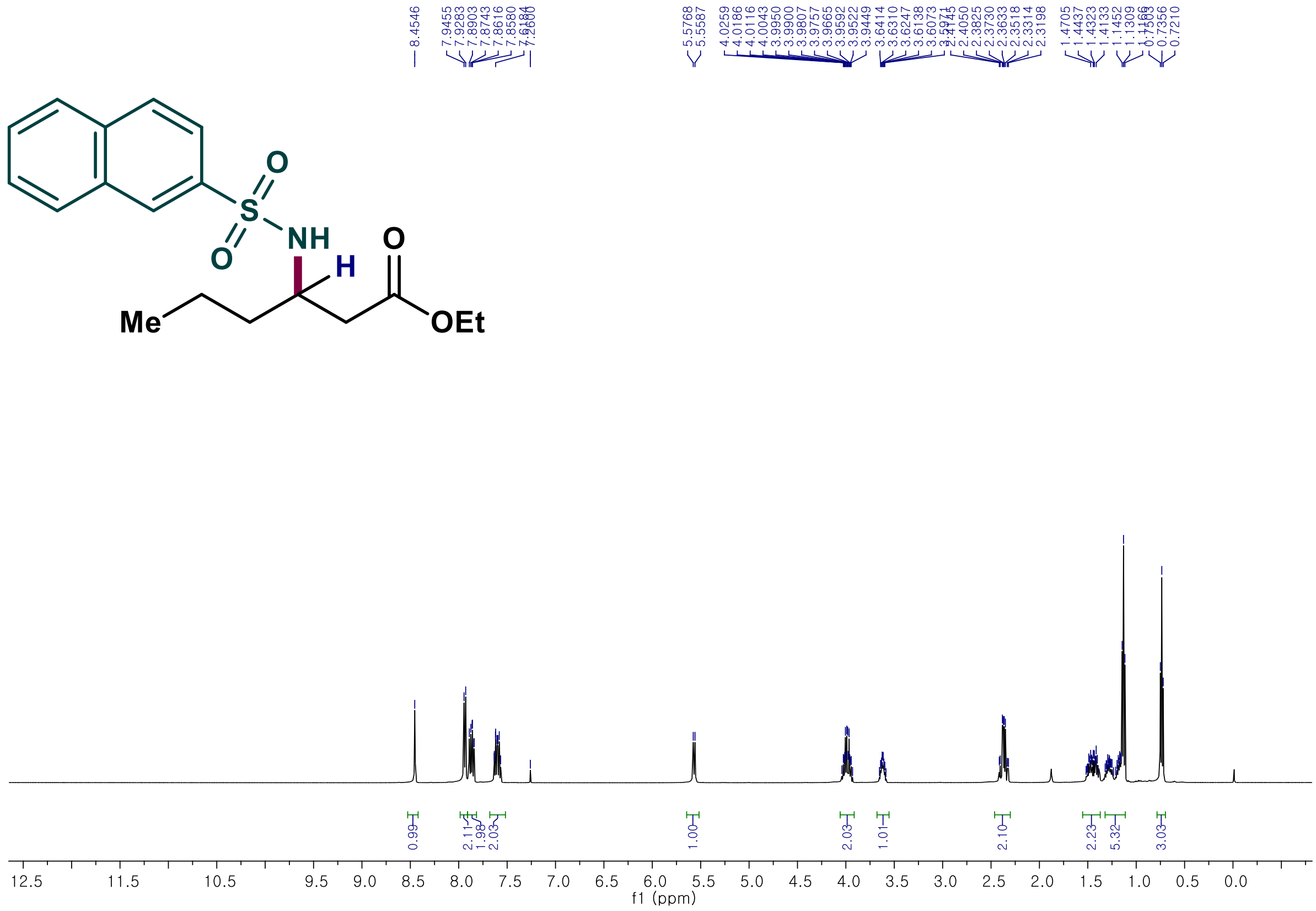

^{13}C NMR spectra (**65**)

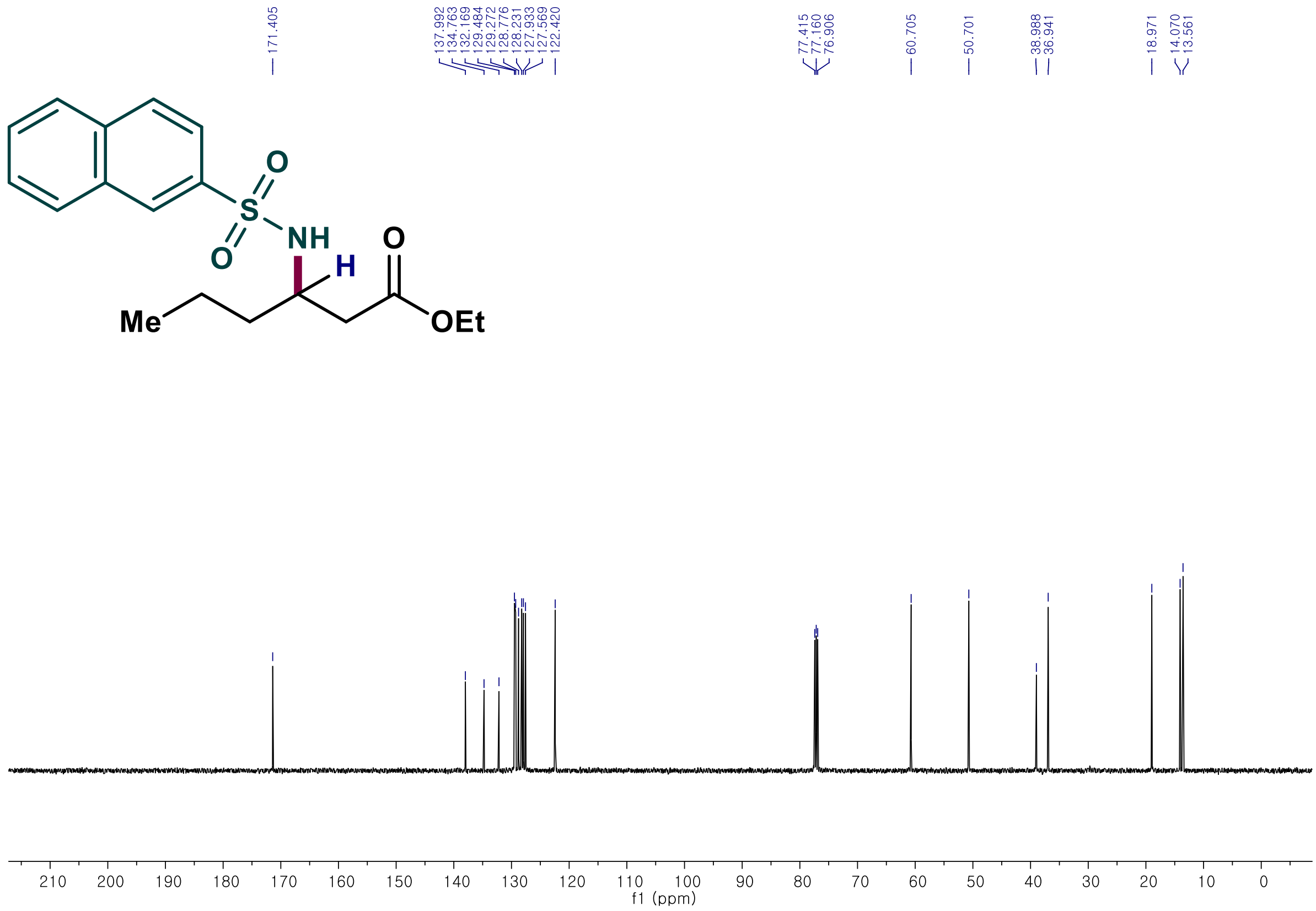

^{1}H NMR spectra (**66**)

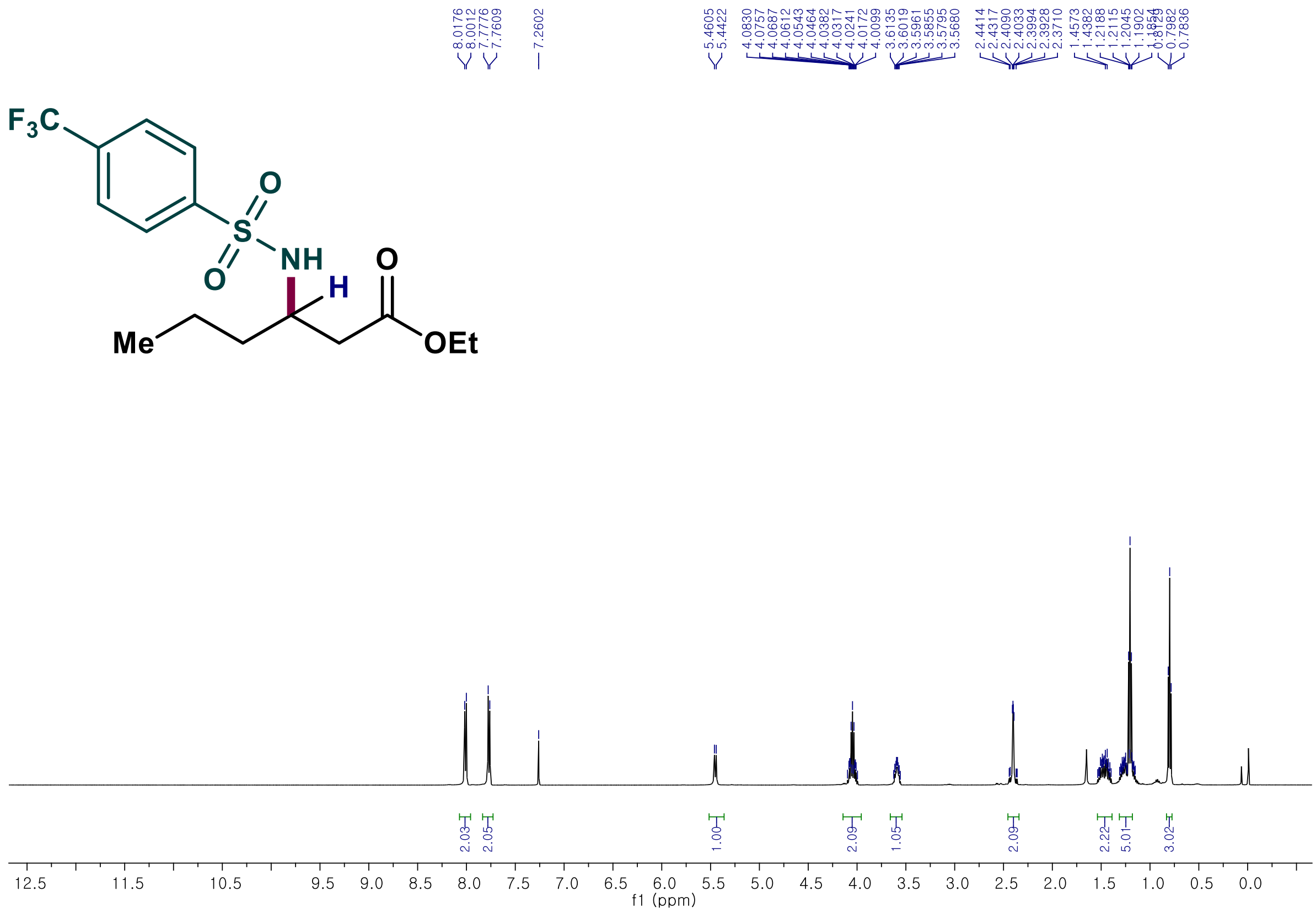

^{13}C NMR spectra (**66**)

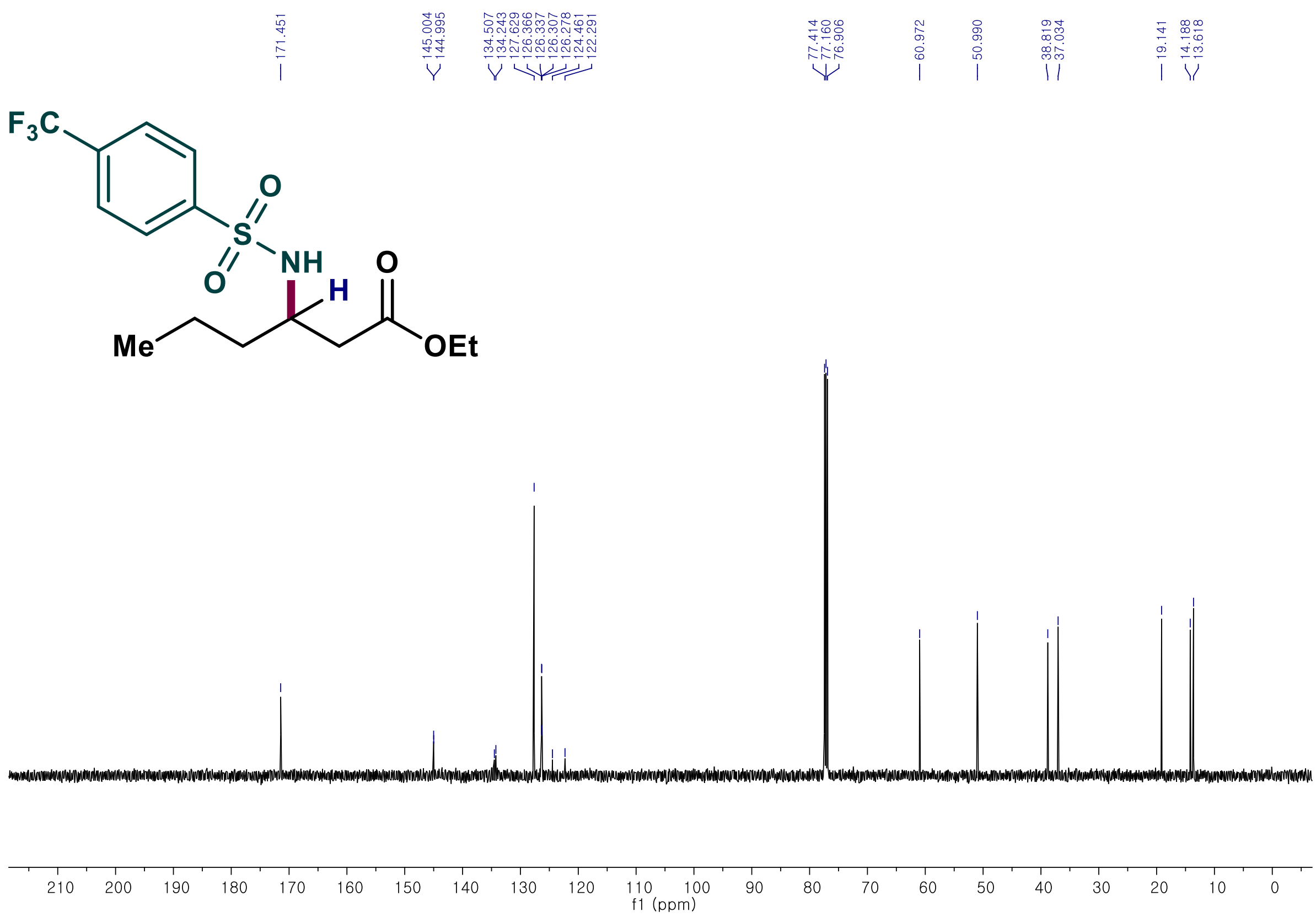

^{19}F NMR spectra (**66**)

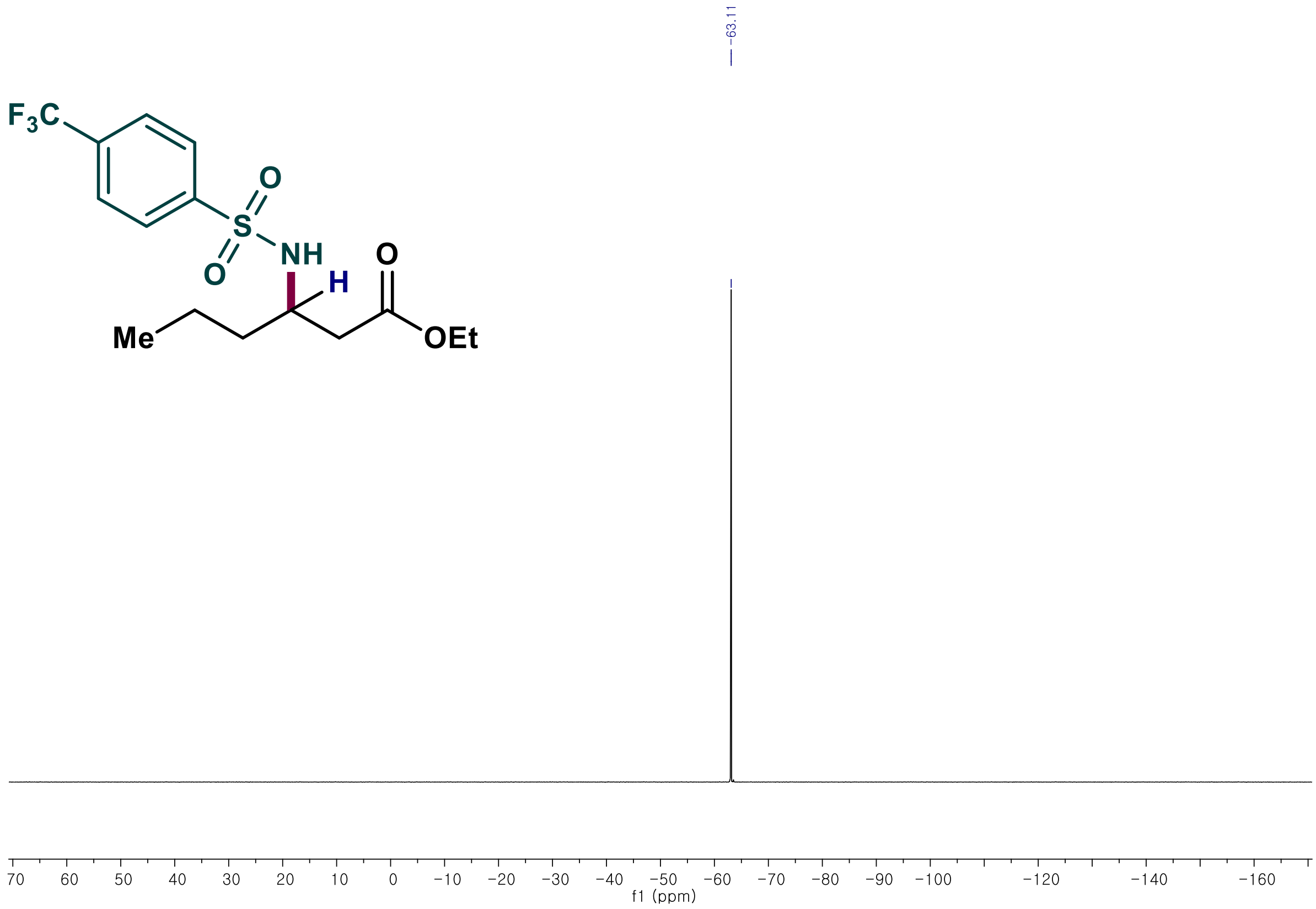

^{1}H NMR spectra (**67**)

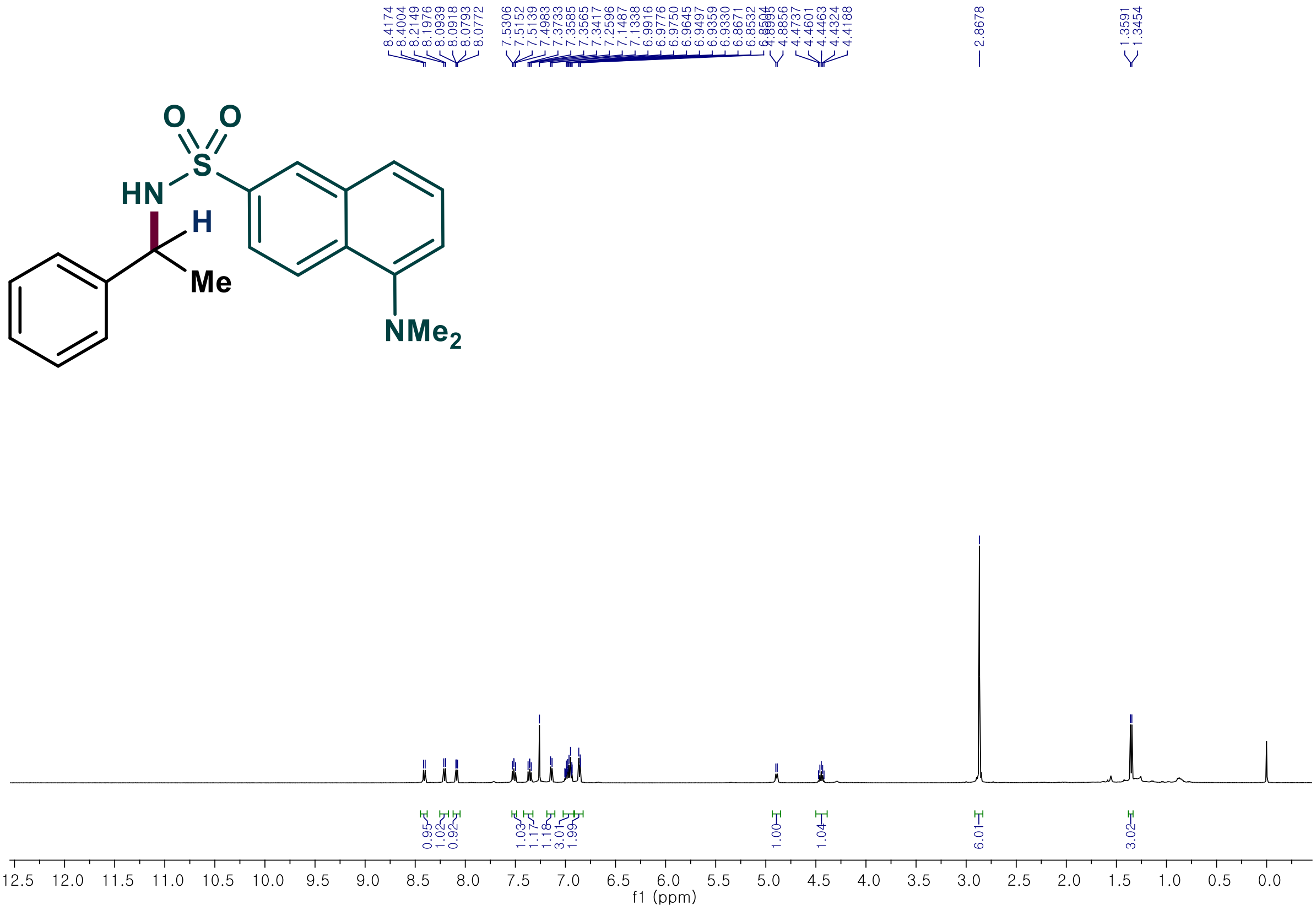

^{13}C NMR spectra (**67**)

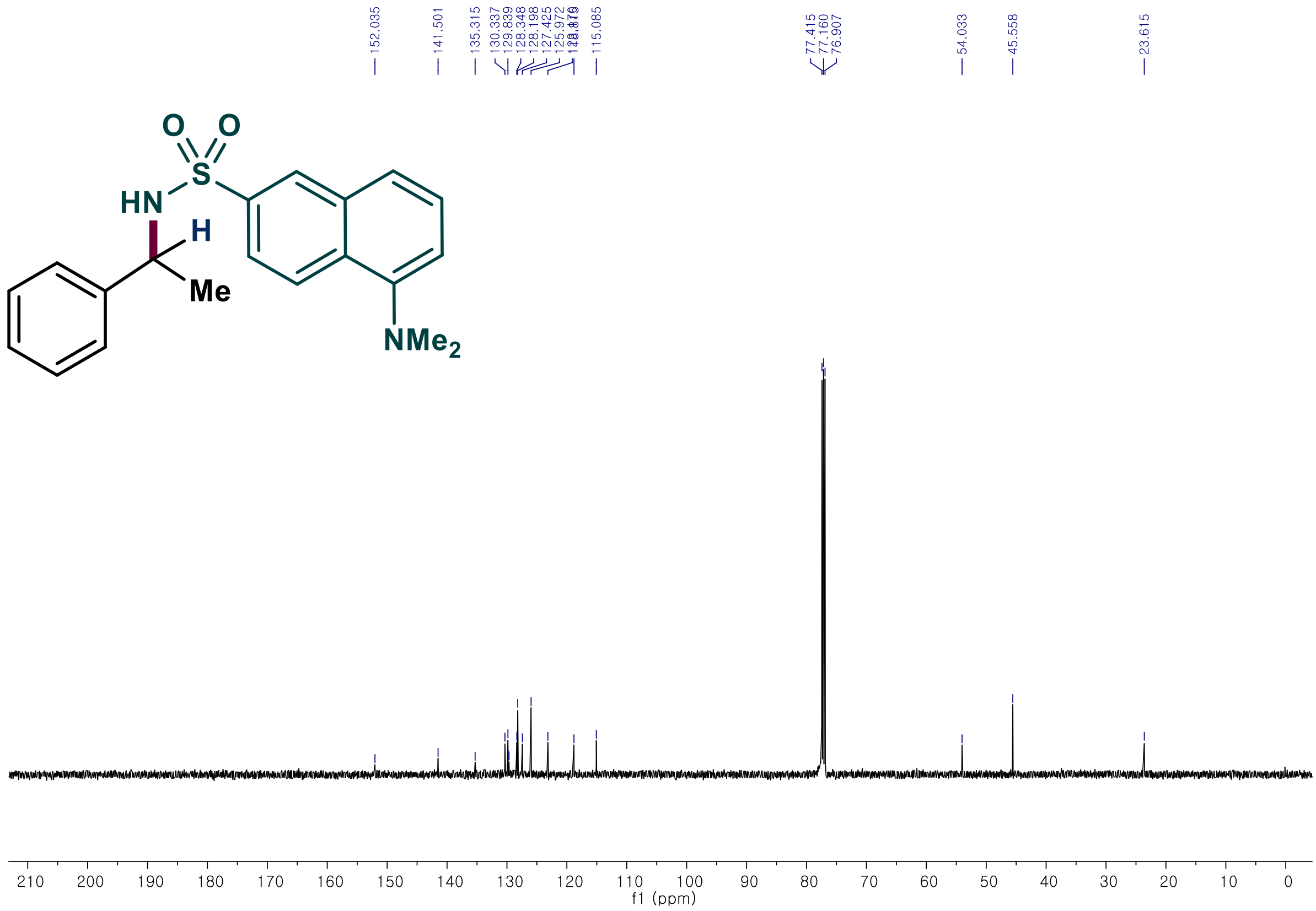

^{1}H NMR spectra (**68**)

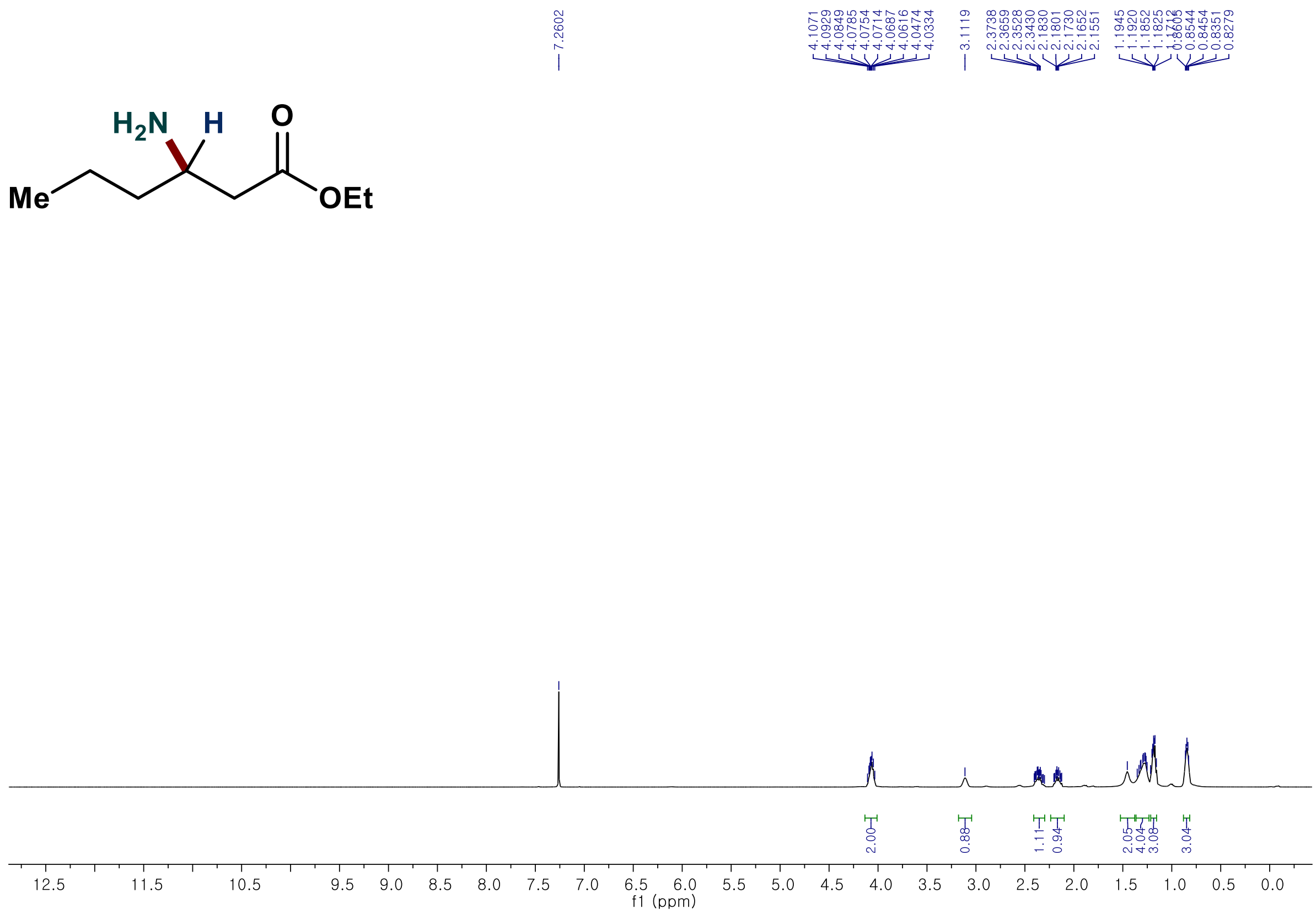

^{13}C NMR spectra (**68**)

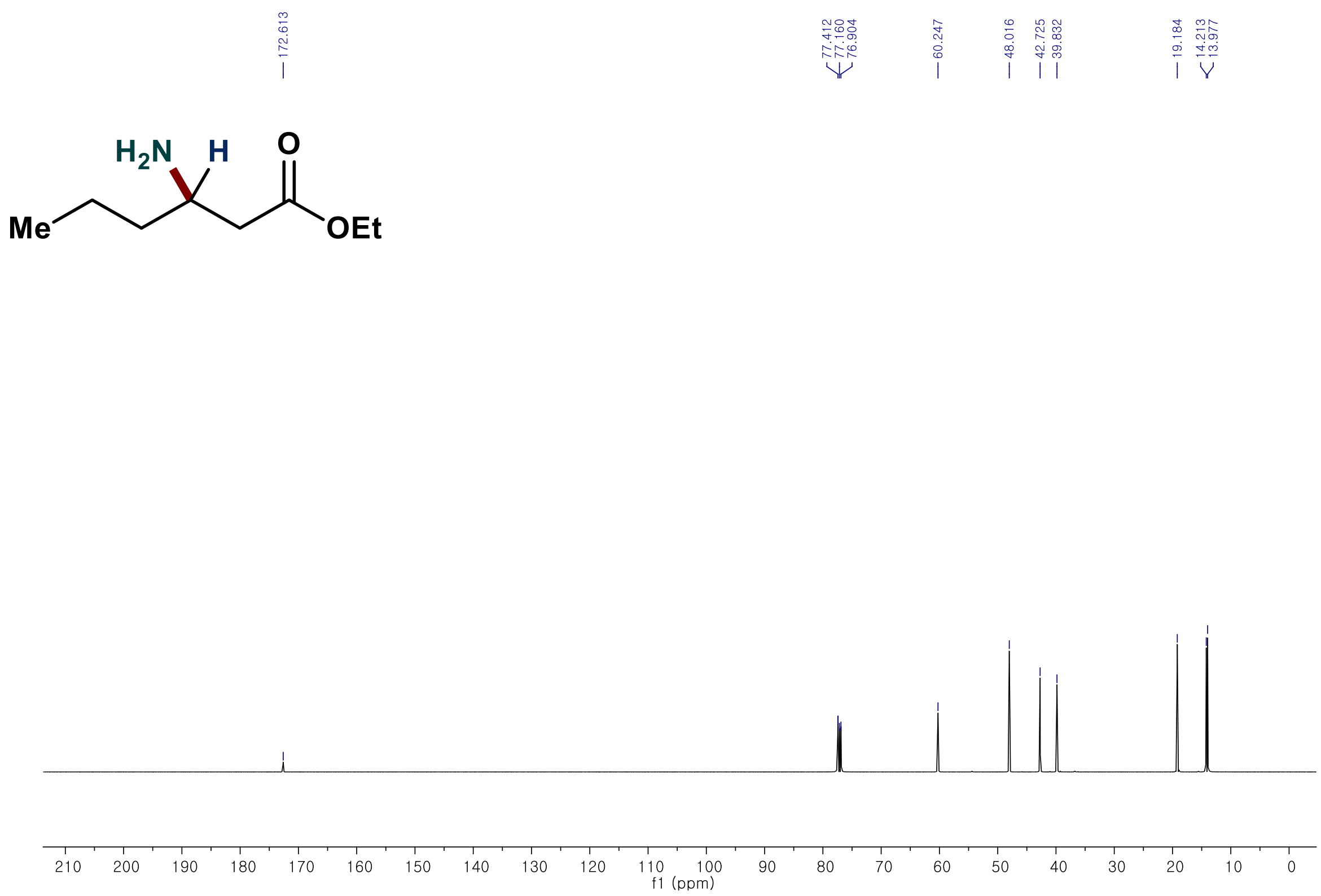

^{1}H NMR spectra (**69**)

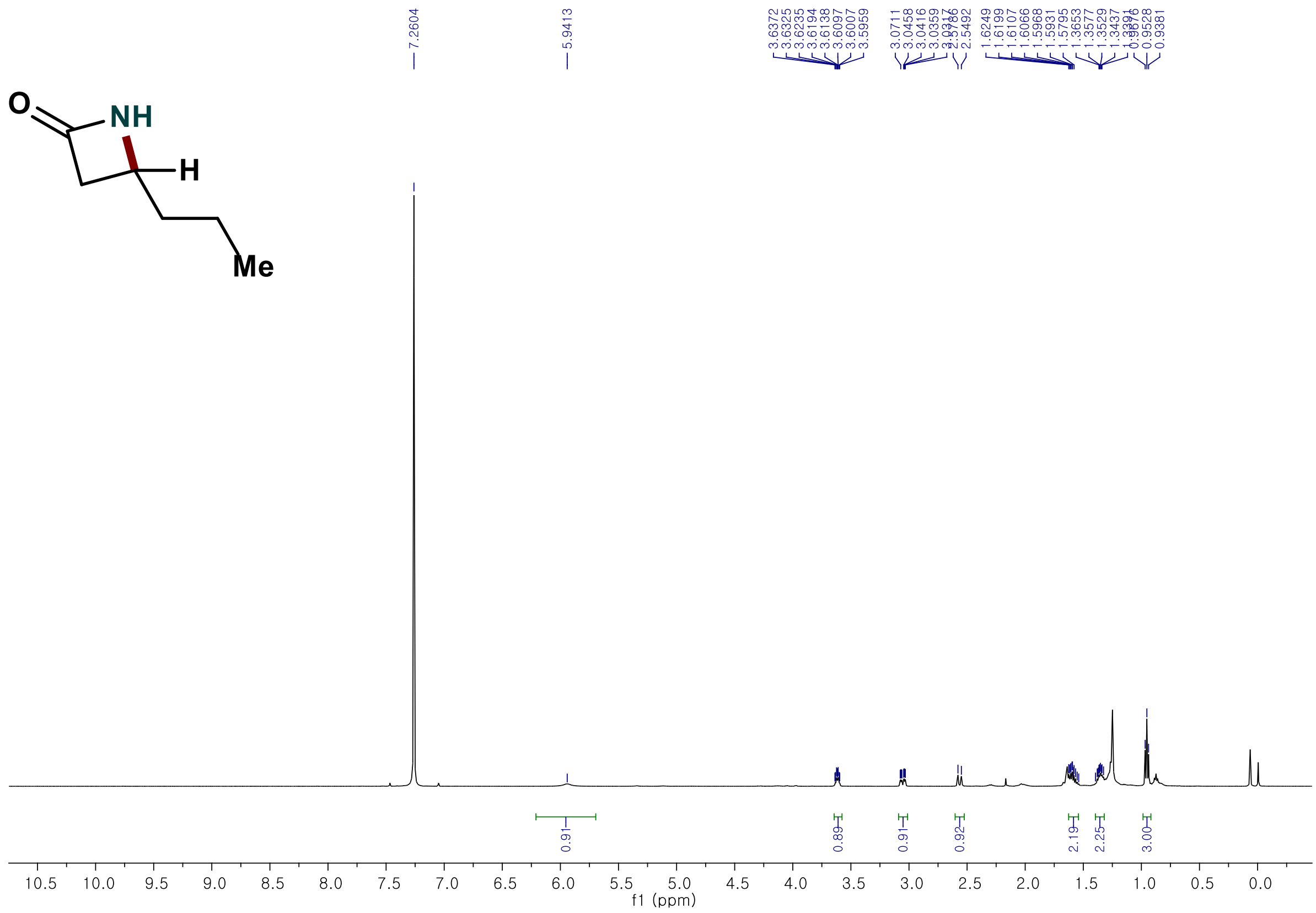

^{13}C NMR spectra (**69**)

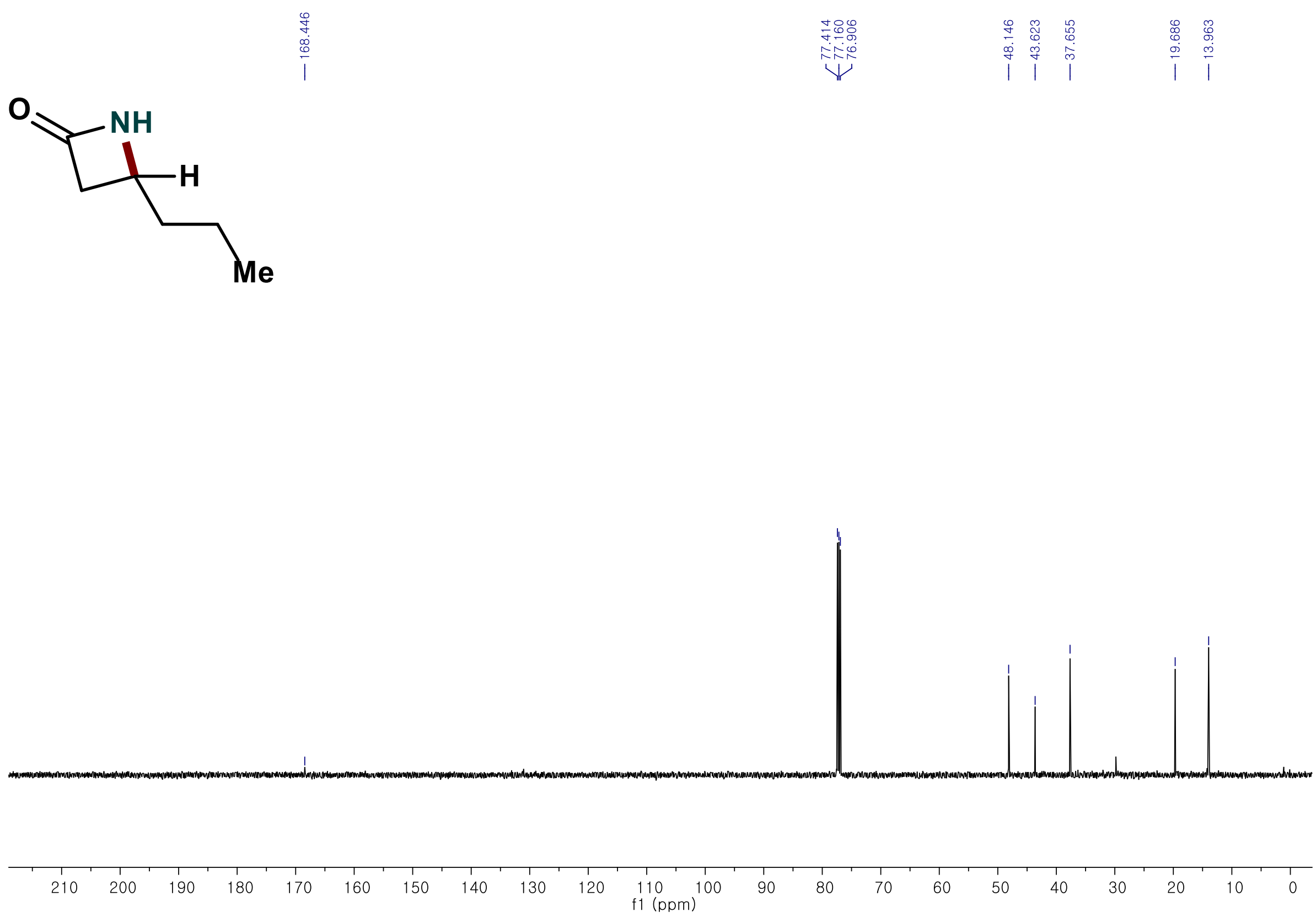

^{1}H NMR spectra (**70**)

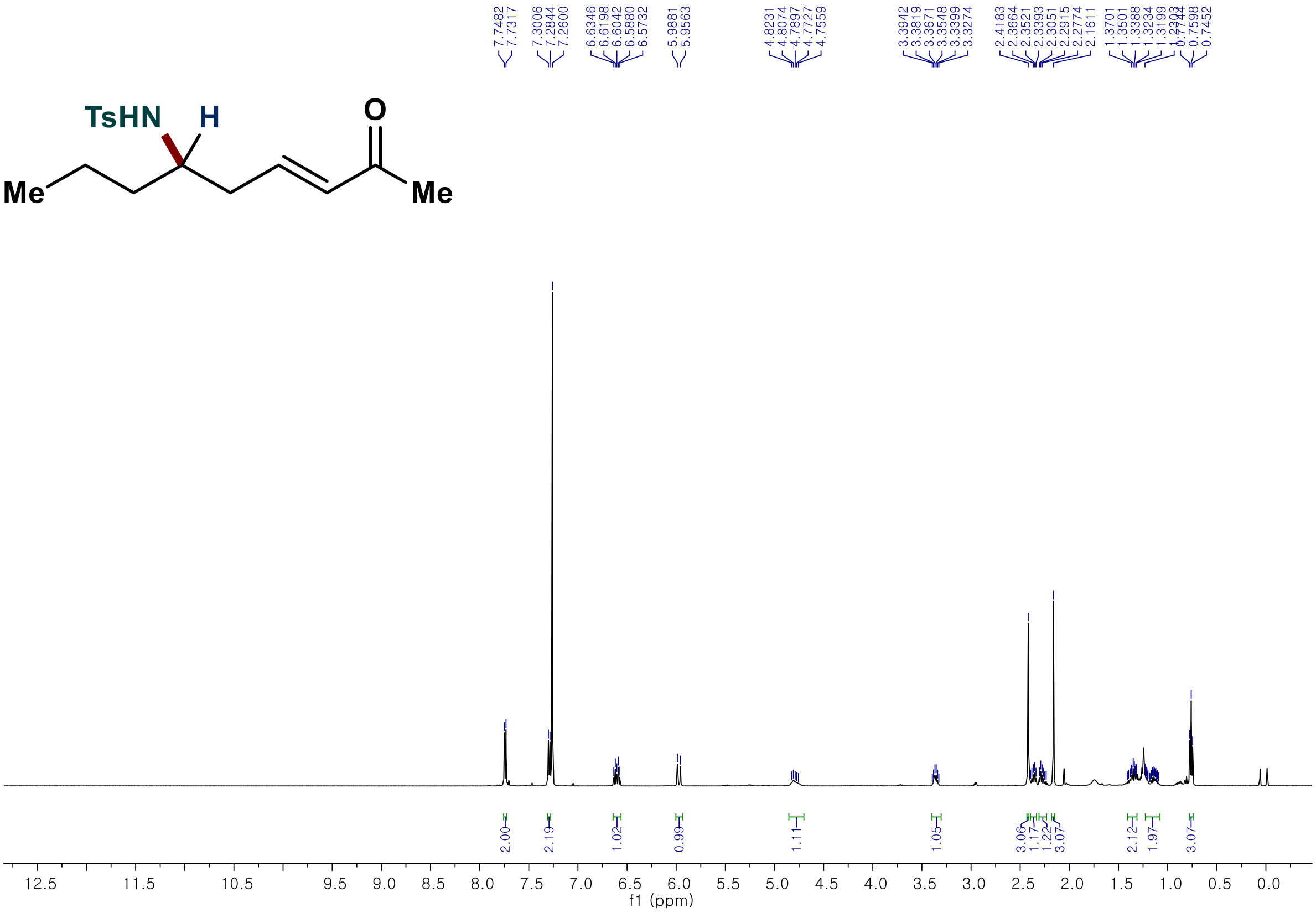

^{13}C NMR spectra (**70**)

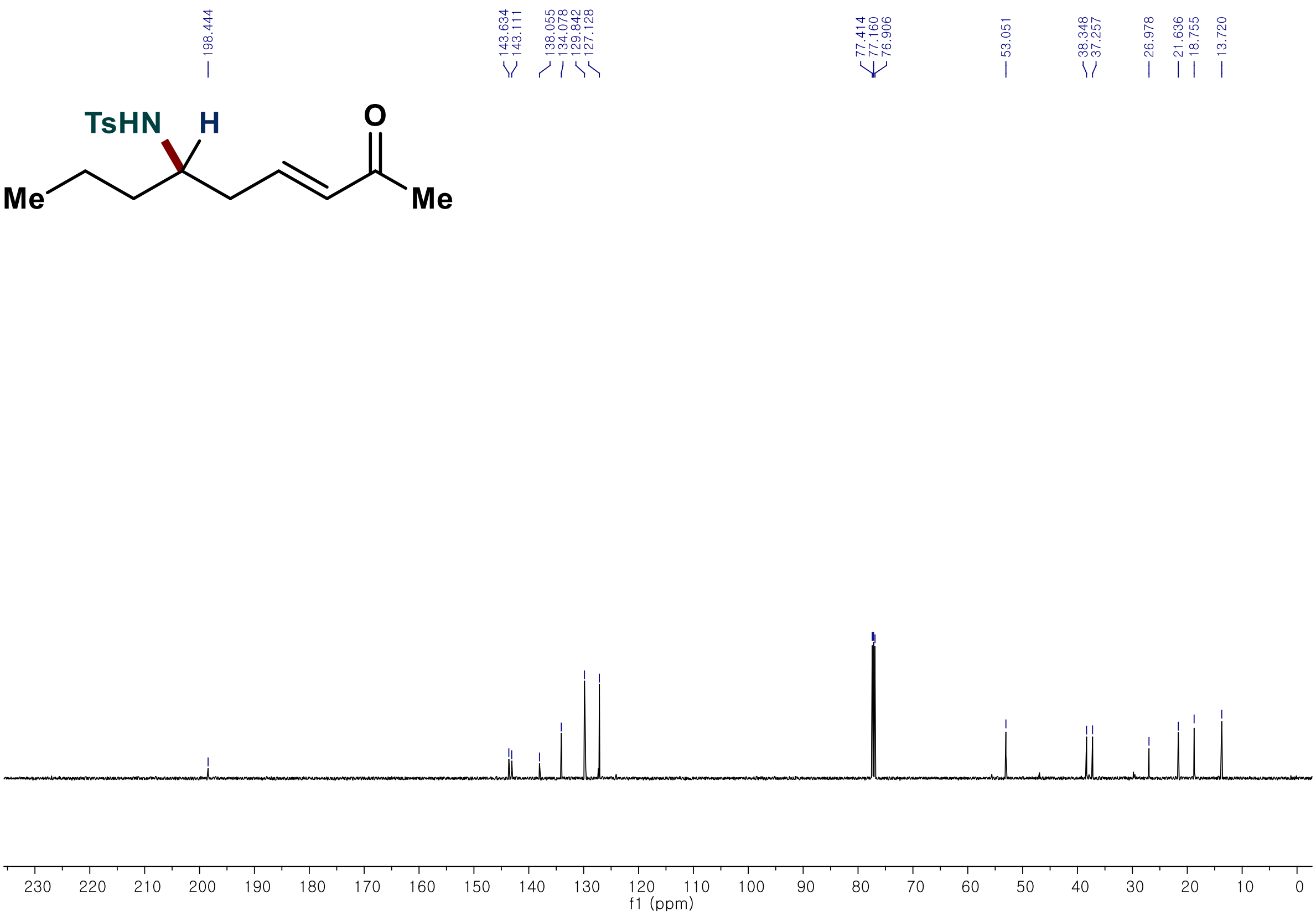

^{1}H NMR spectra (**71**)

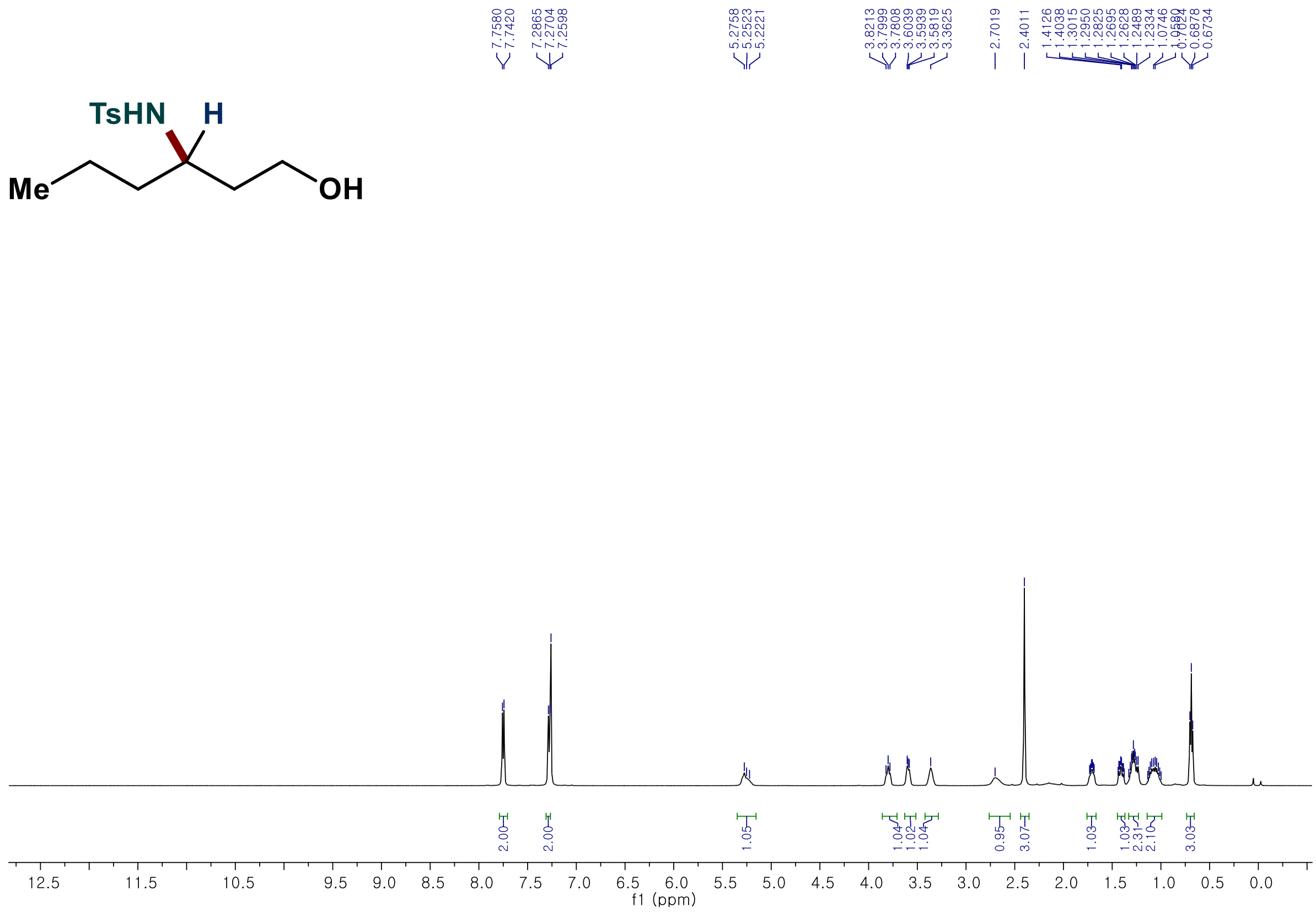

^{13}C NMR spectra (**71**)

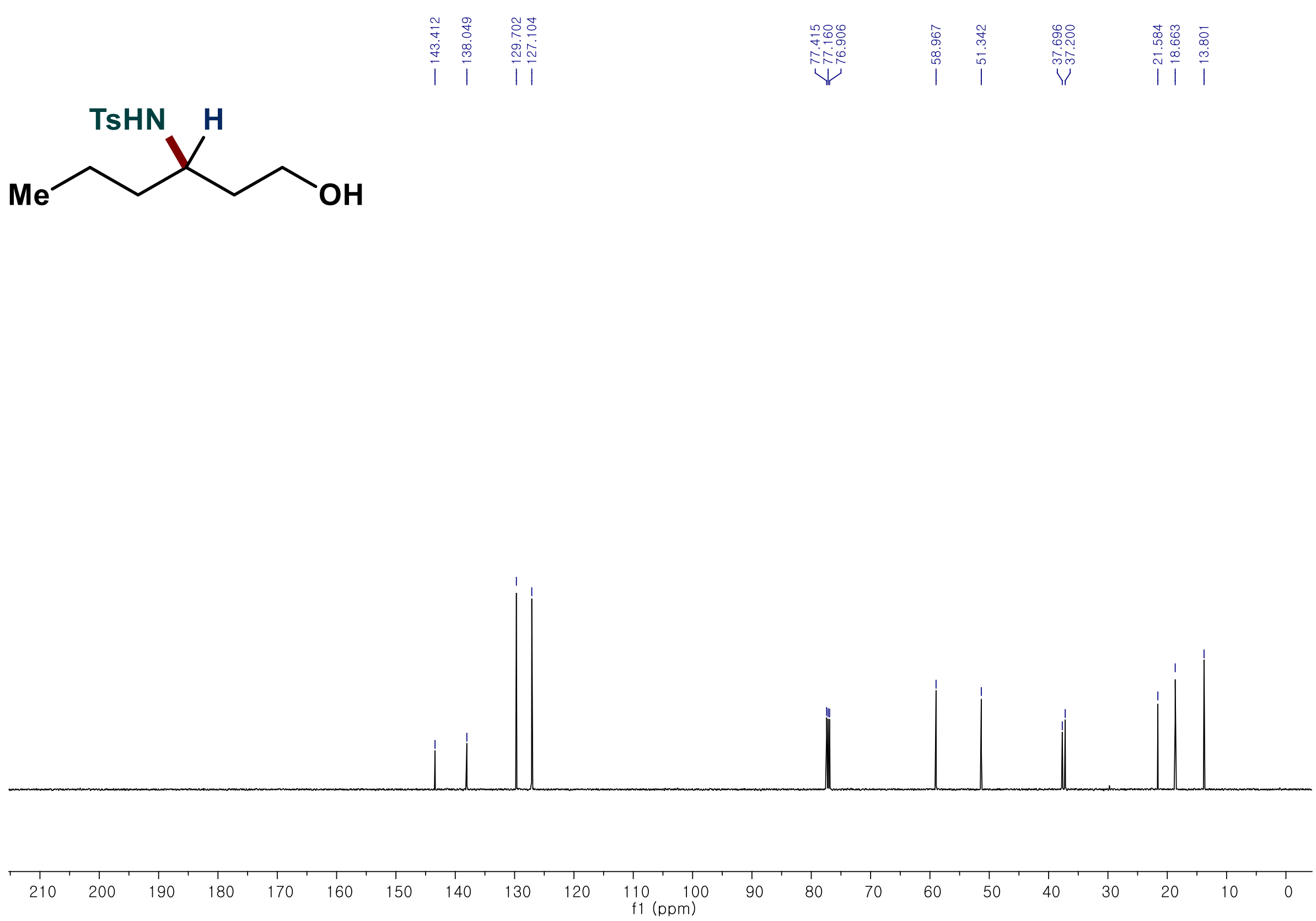

^{1}H NMR spectra (**72**)

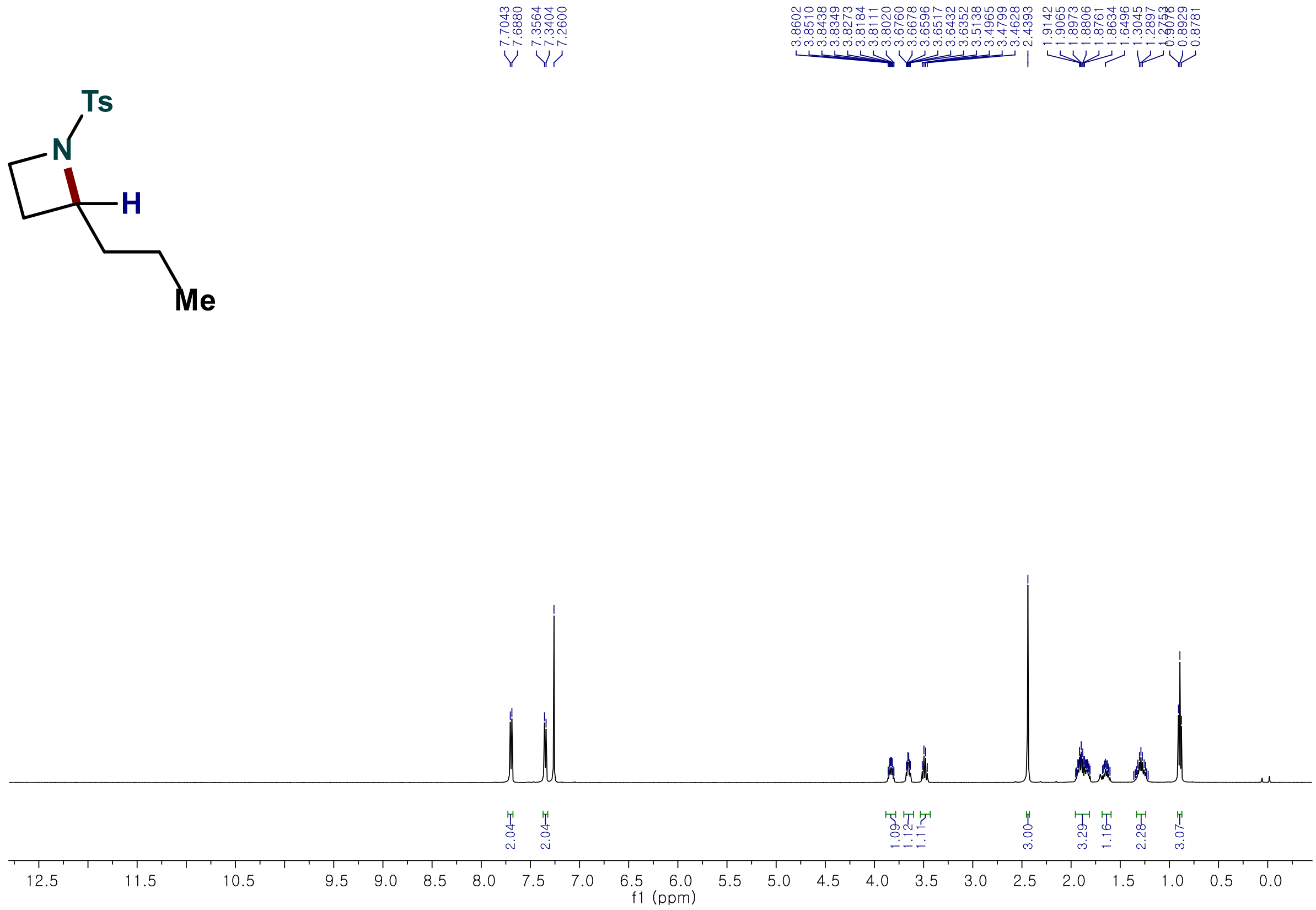

^{13}C NMR spectra (**72**)

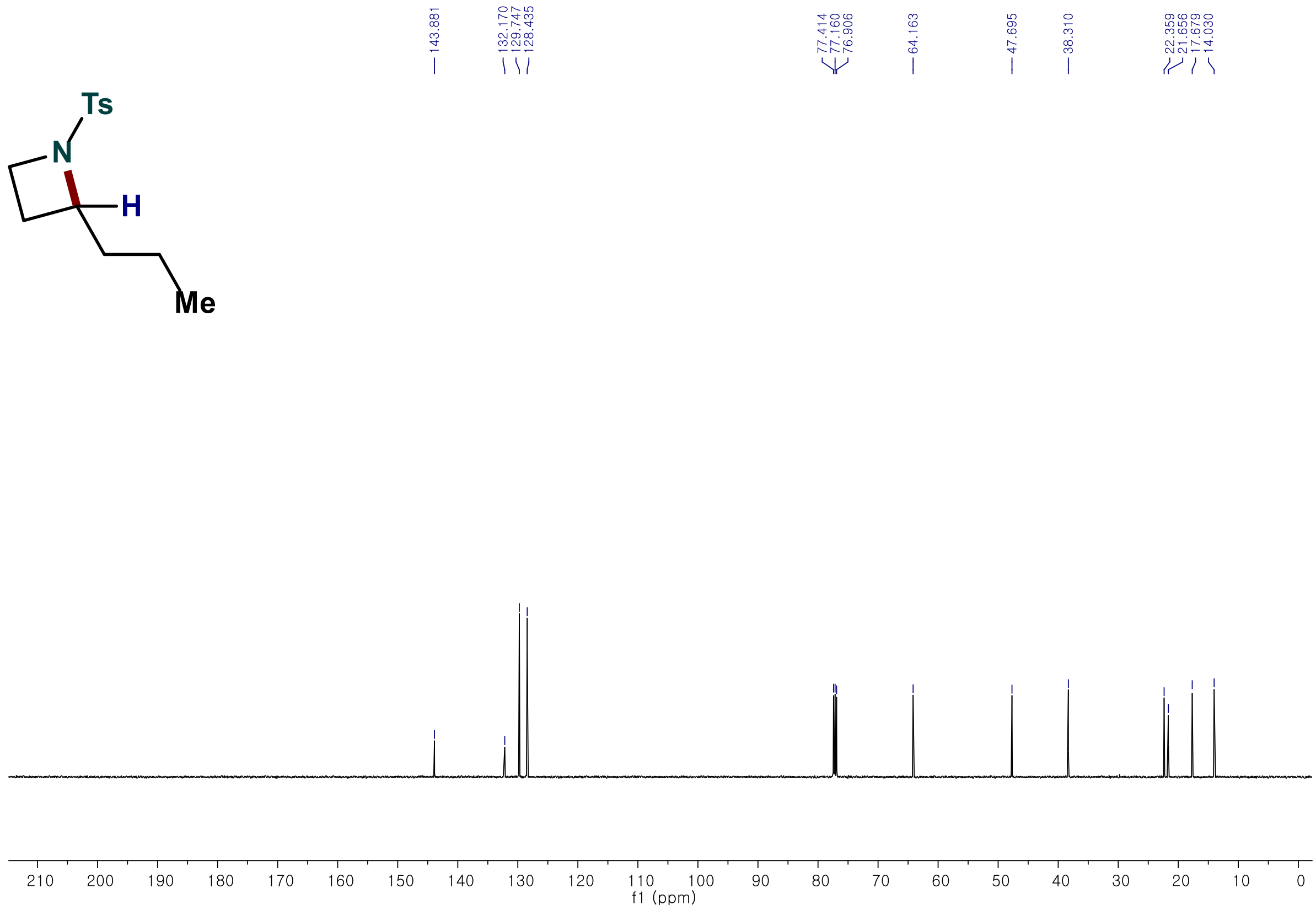

^{1}H NMR spectra (**73**)

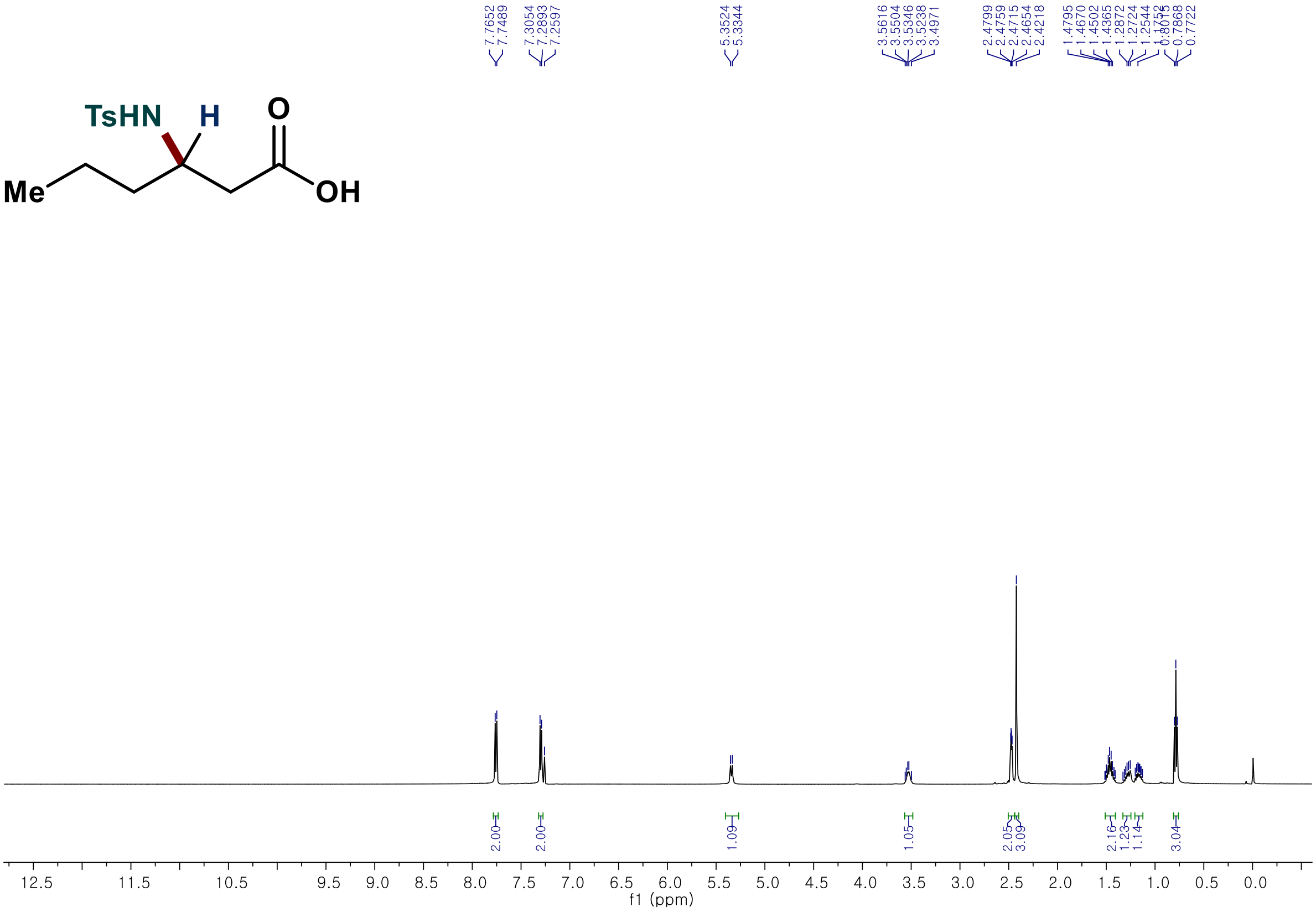

^{13}C NMR spectra (**73**)

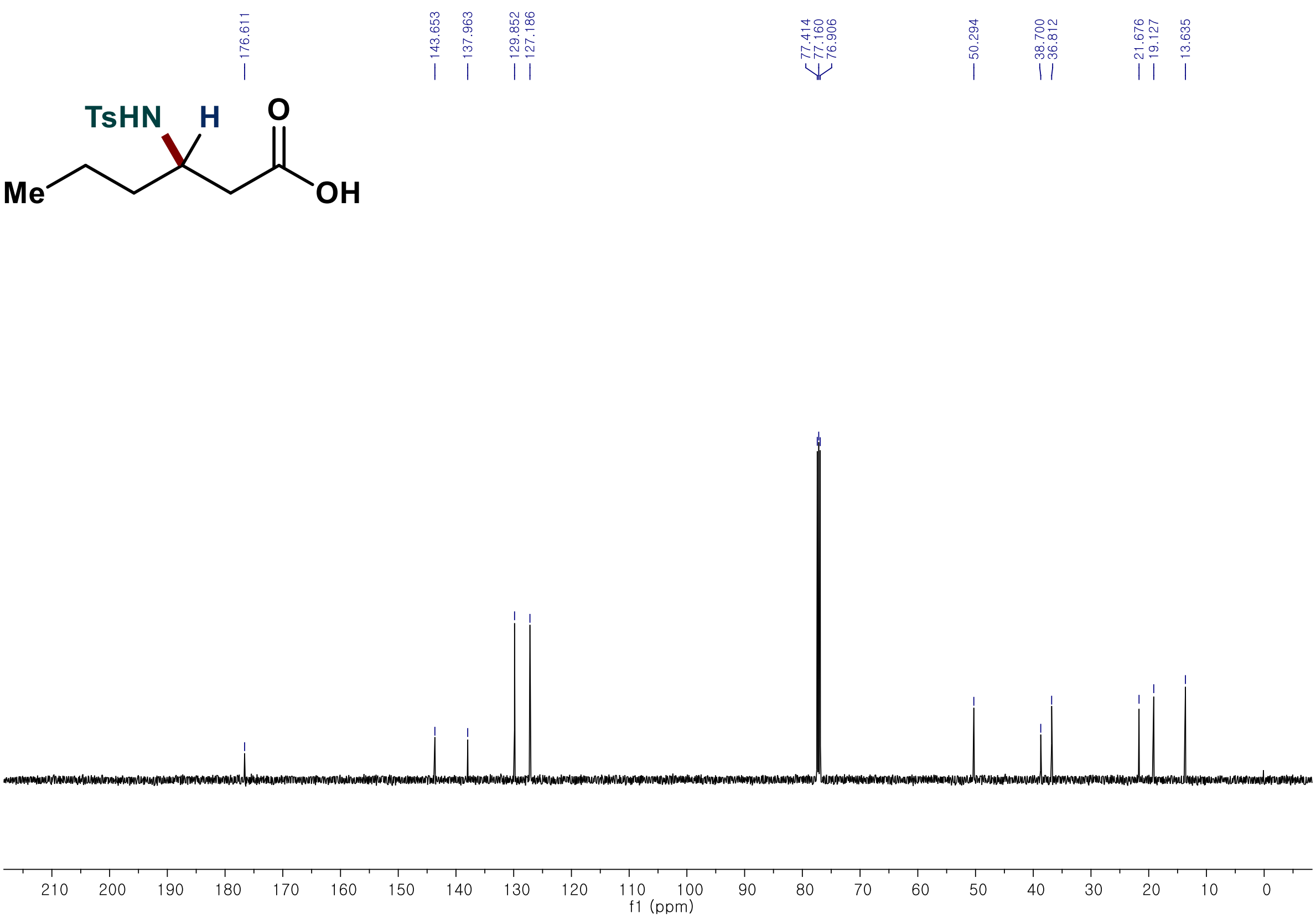

^{1}H NMR spectra (**74**)

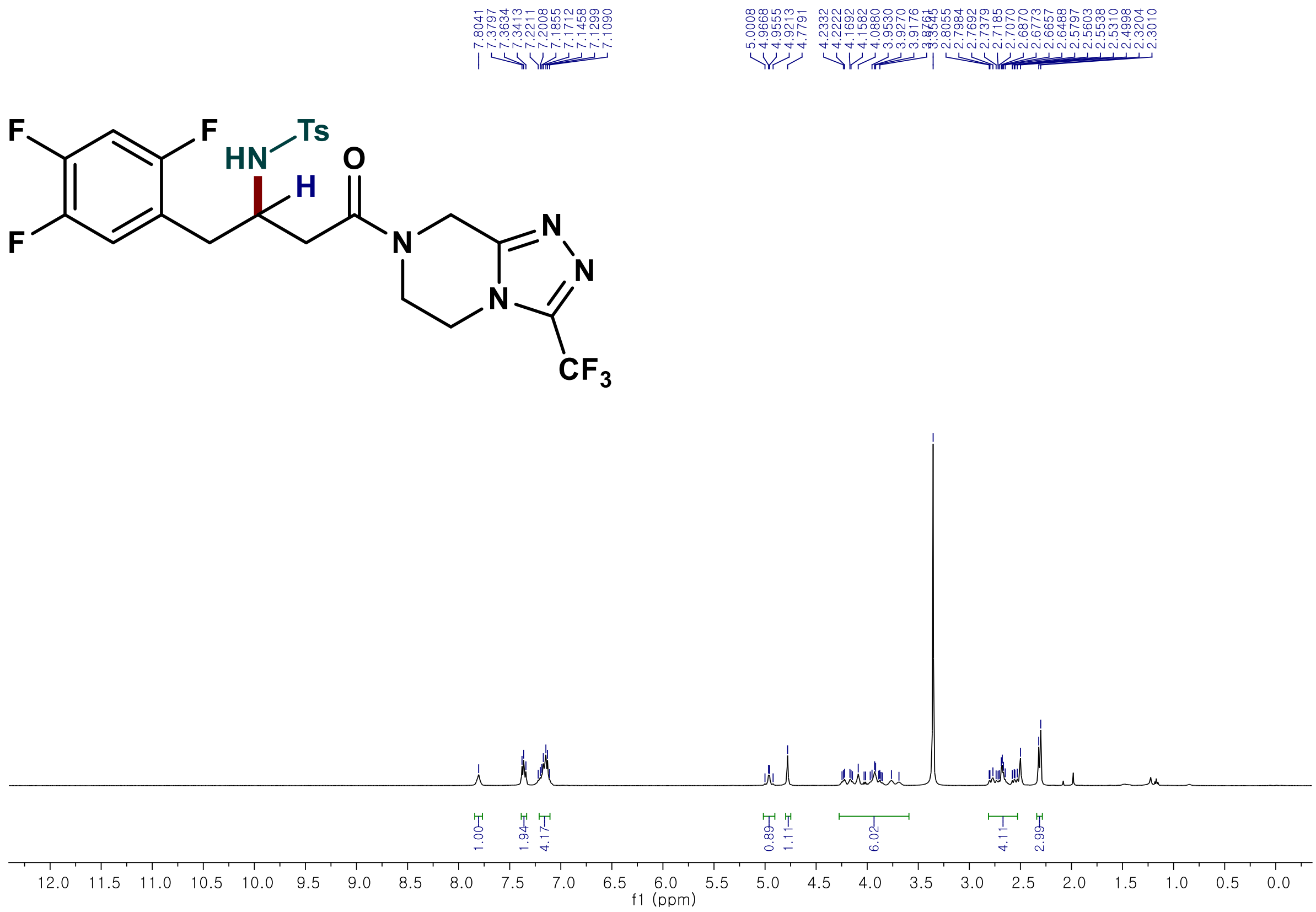

^{13}C NMR spectra (**74**)

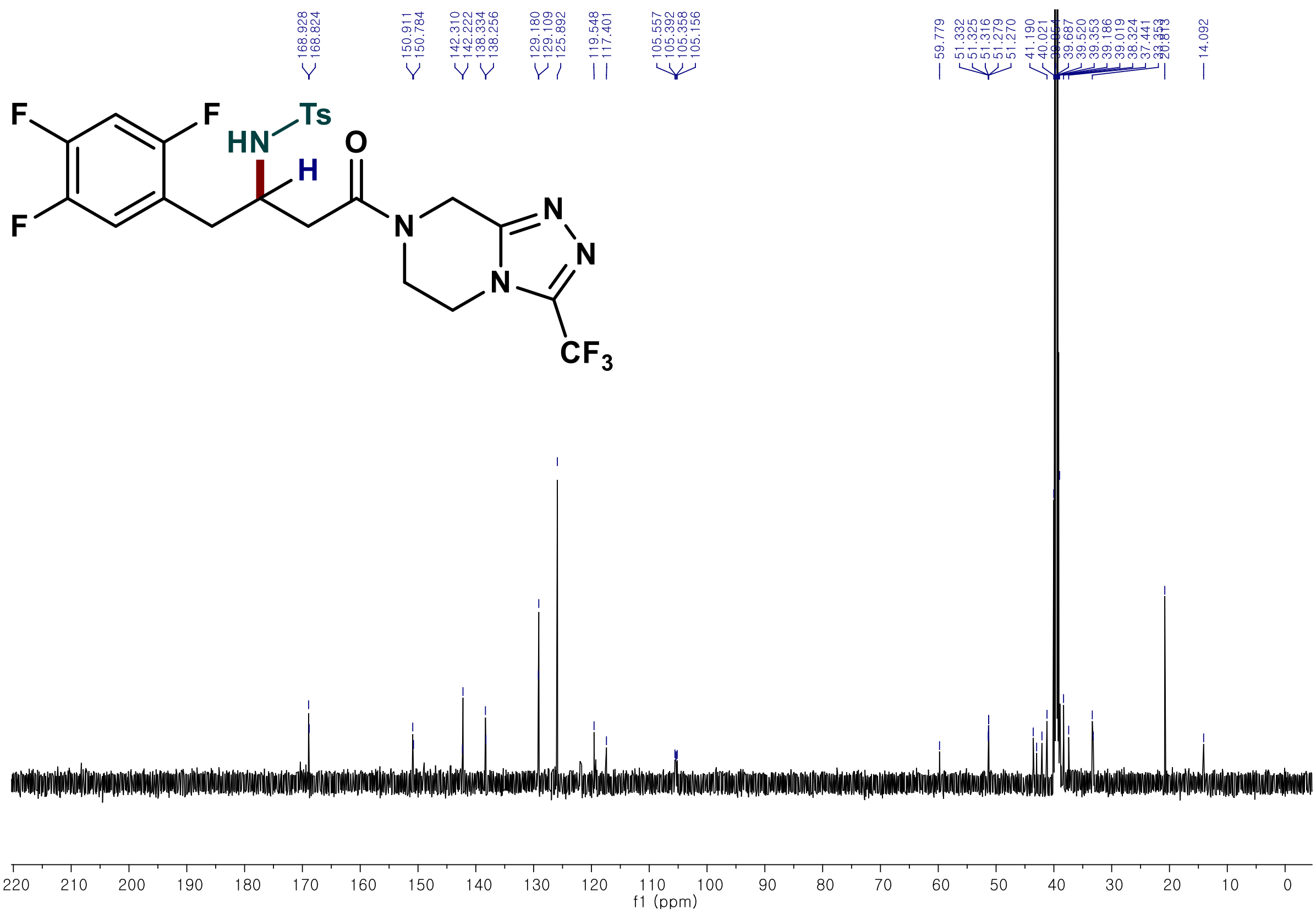

^{19}F NMR spectra (**74**)

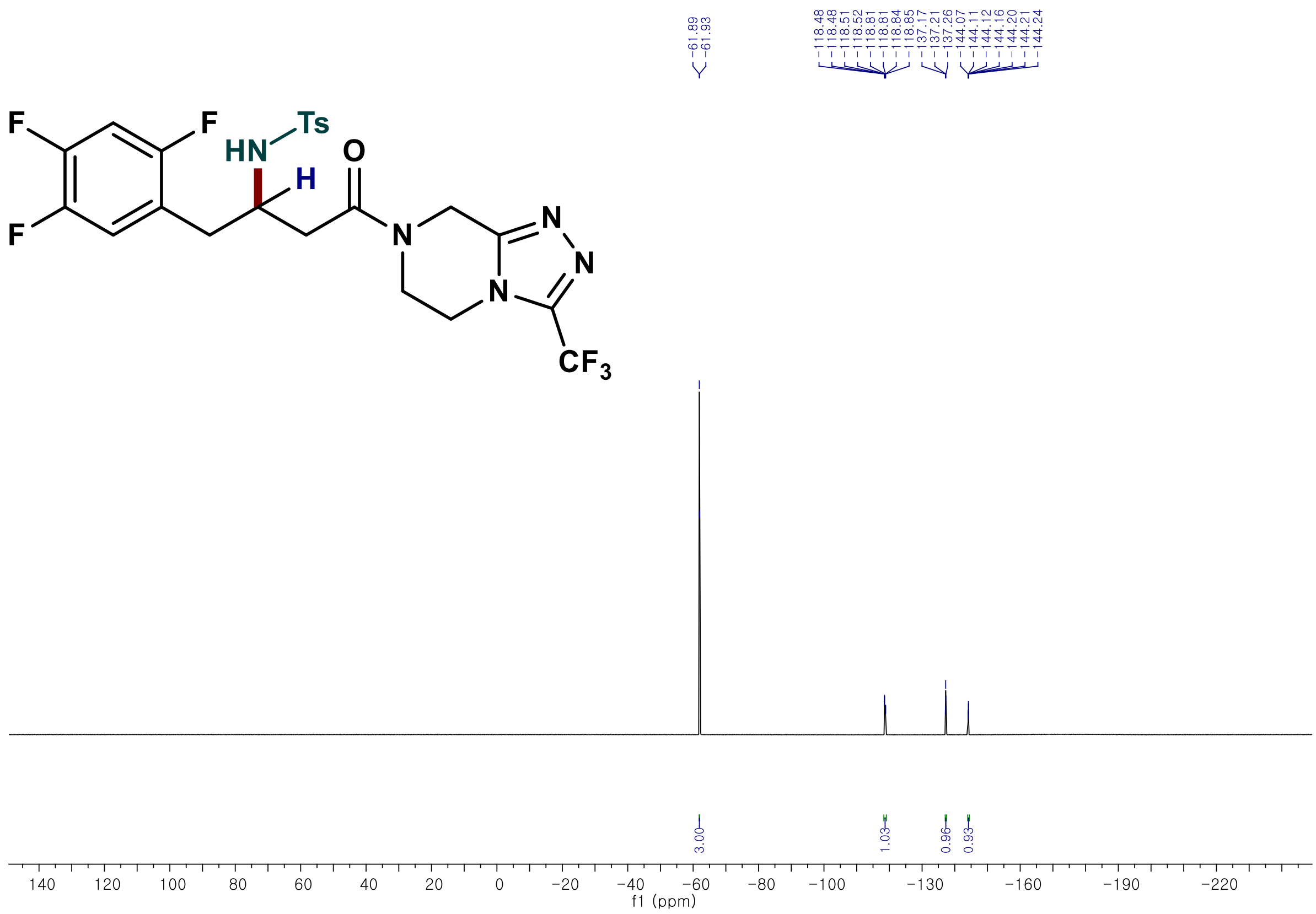

^{1}H NMR spectra (**75**)

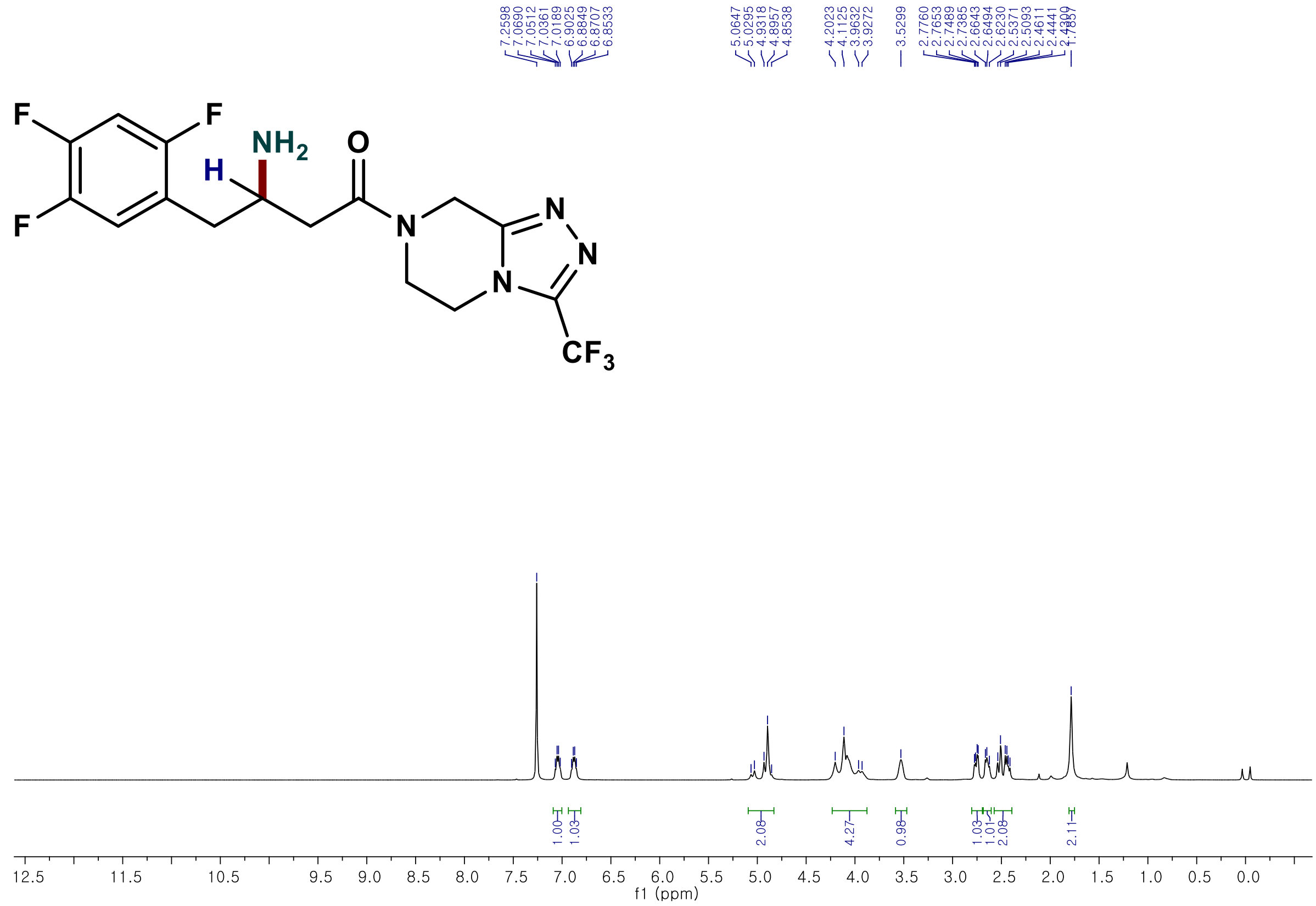

^{13}C NMR spectra (**75**)

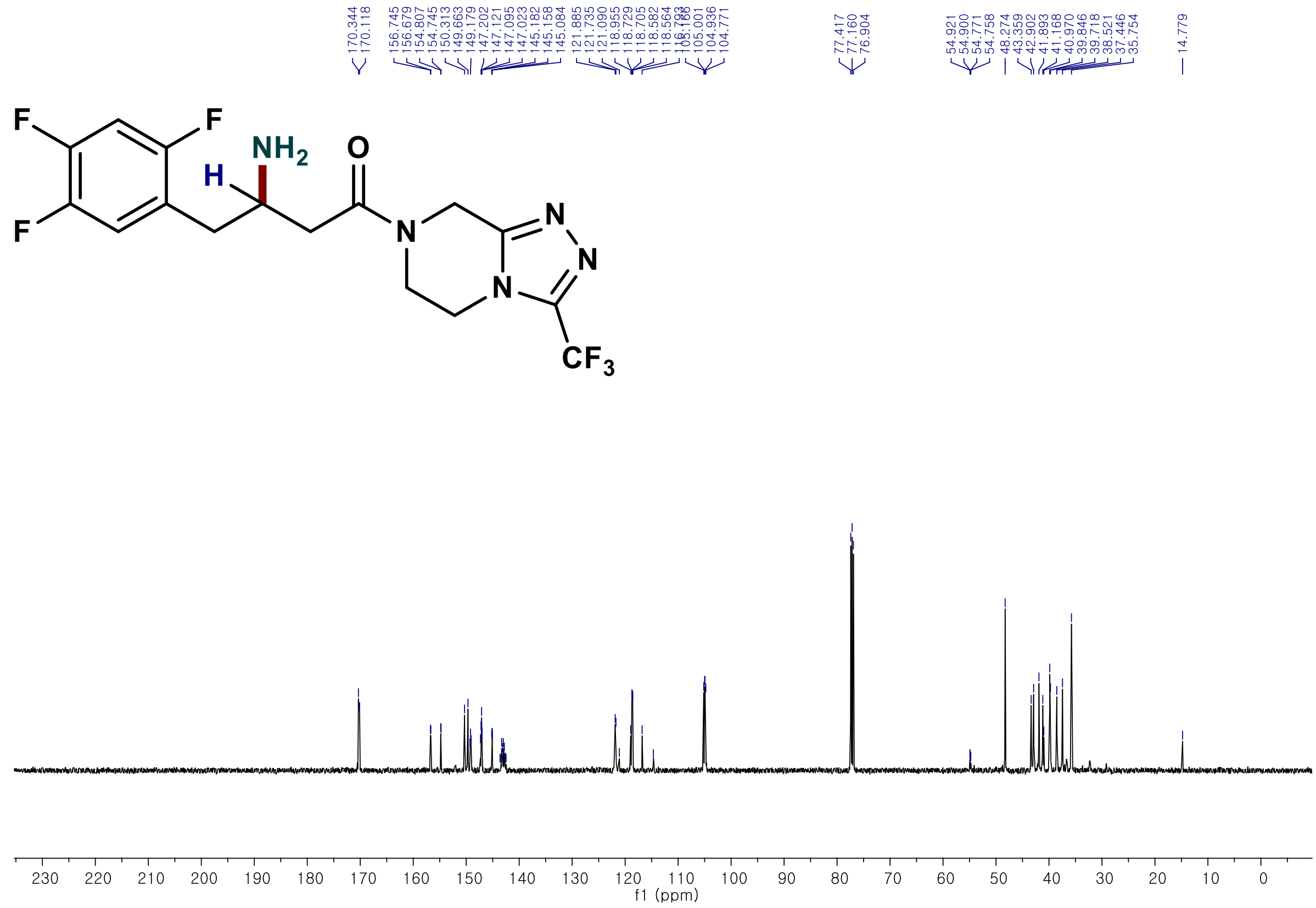

^{19}F NMR spectra (**75**)

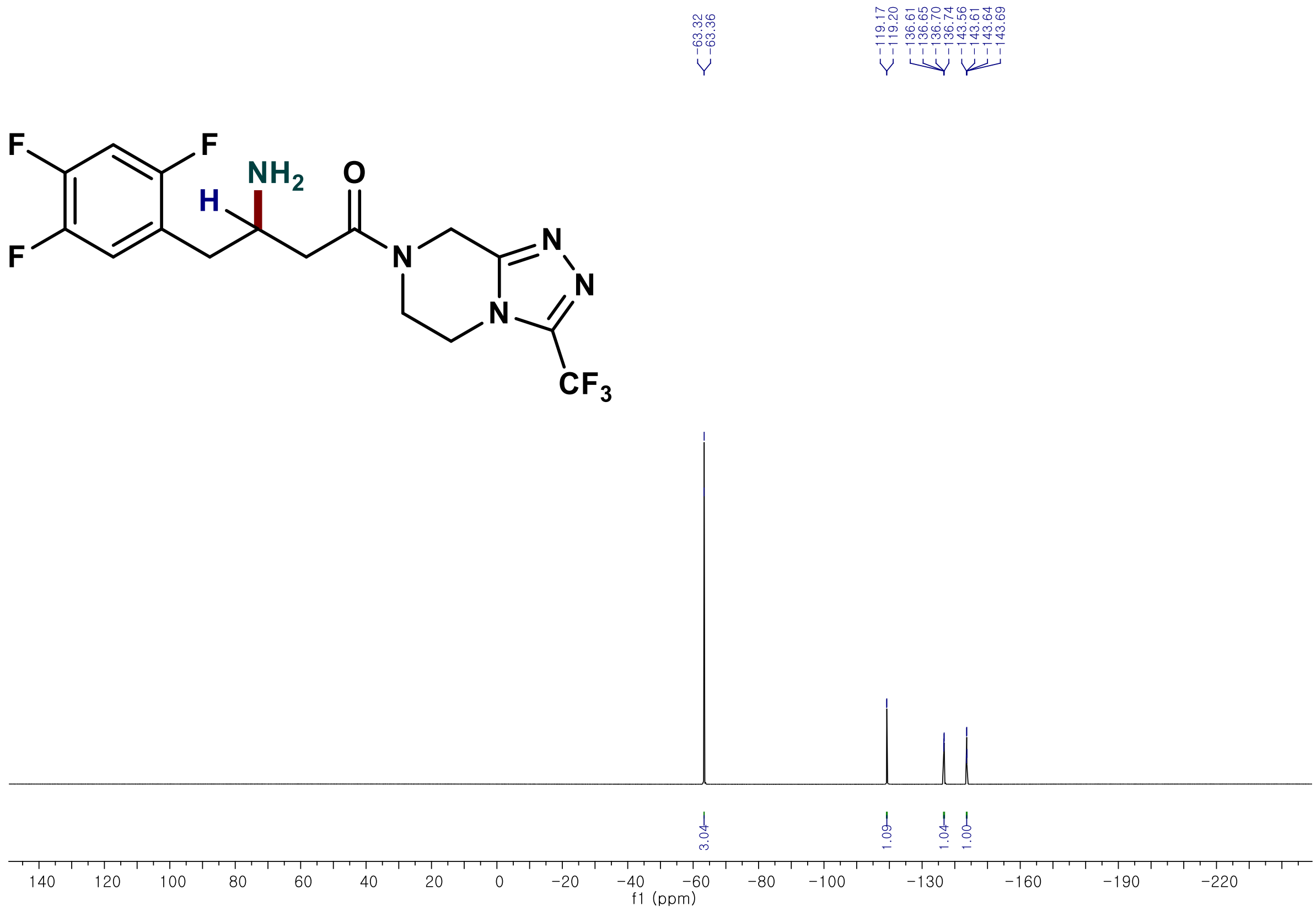

^{1}H NMR spectra (**12a**)

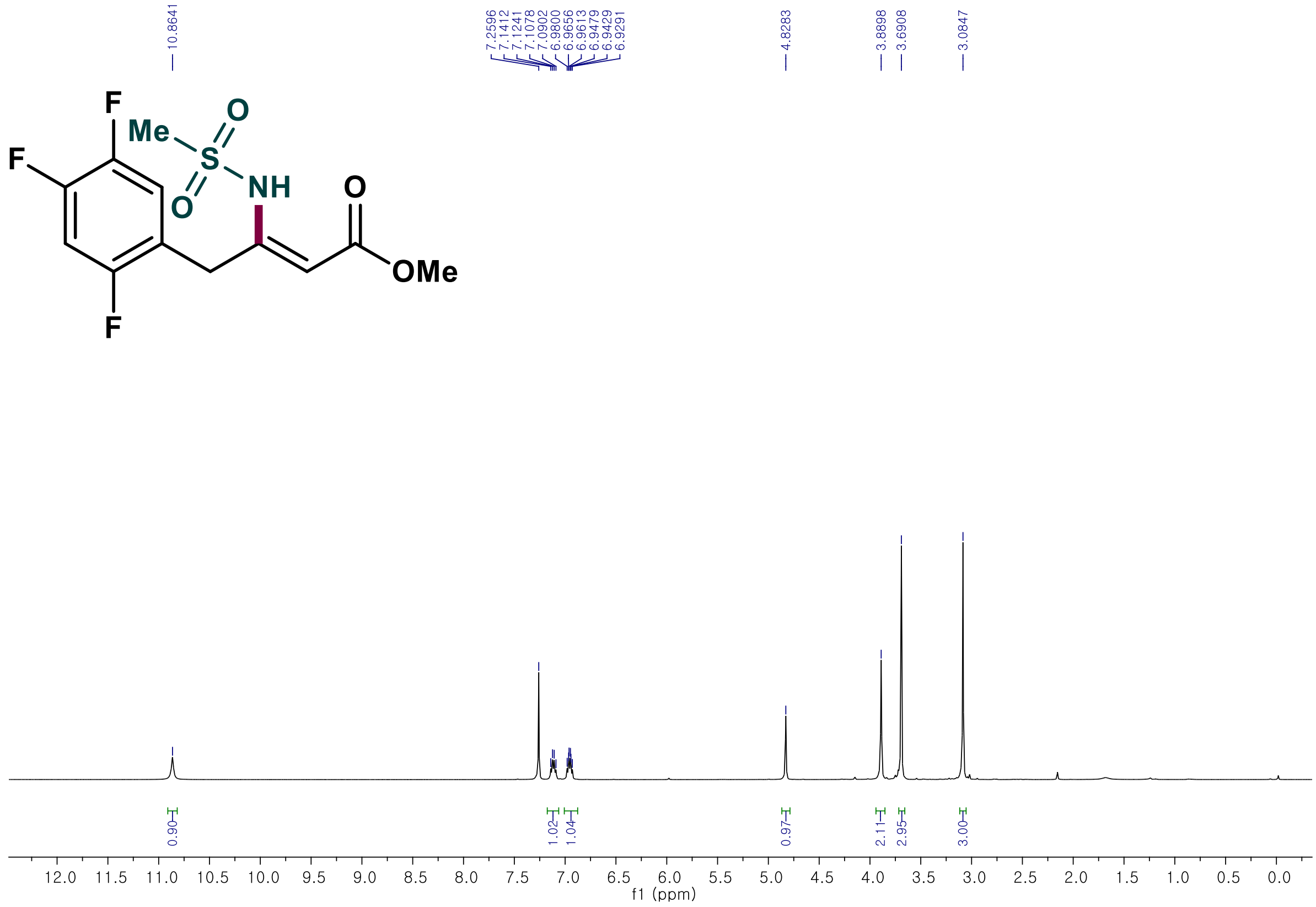

^{13}C NMR spectra (**12a**)

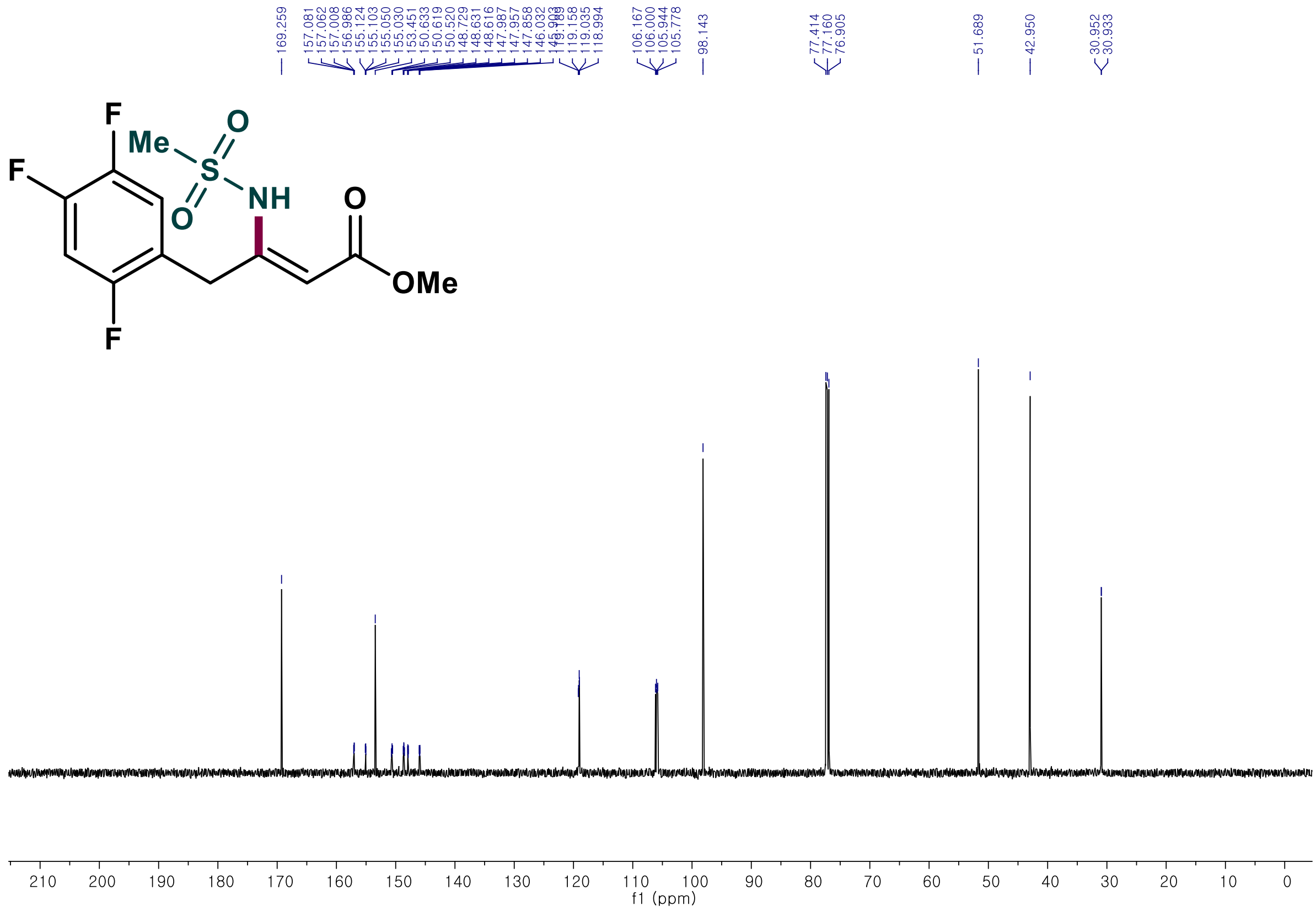

^{19}F NMR spectra (**12a**)

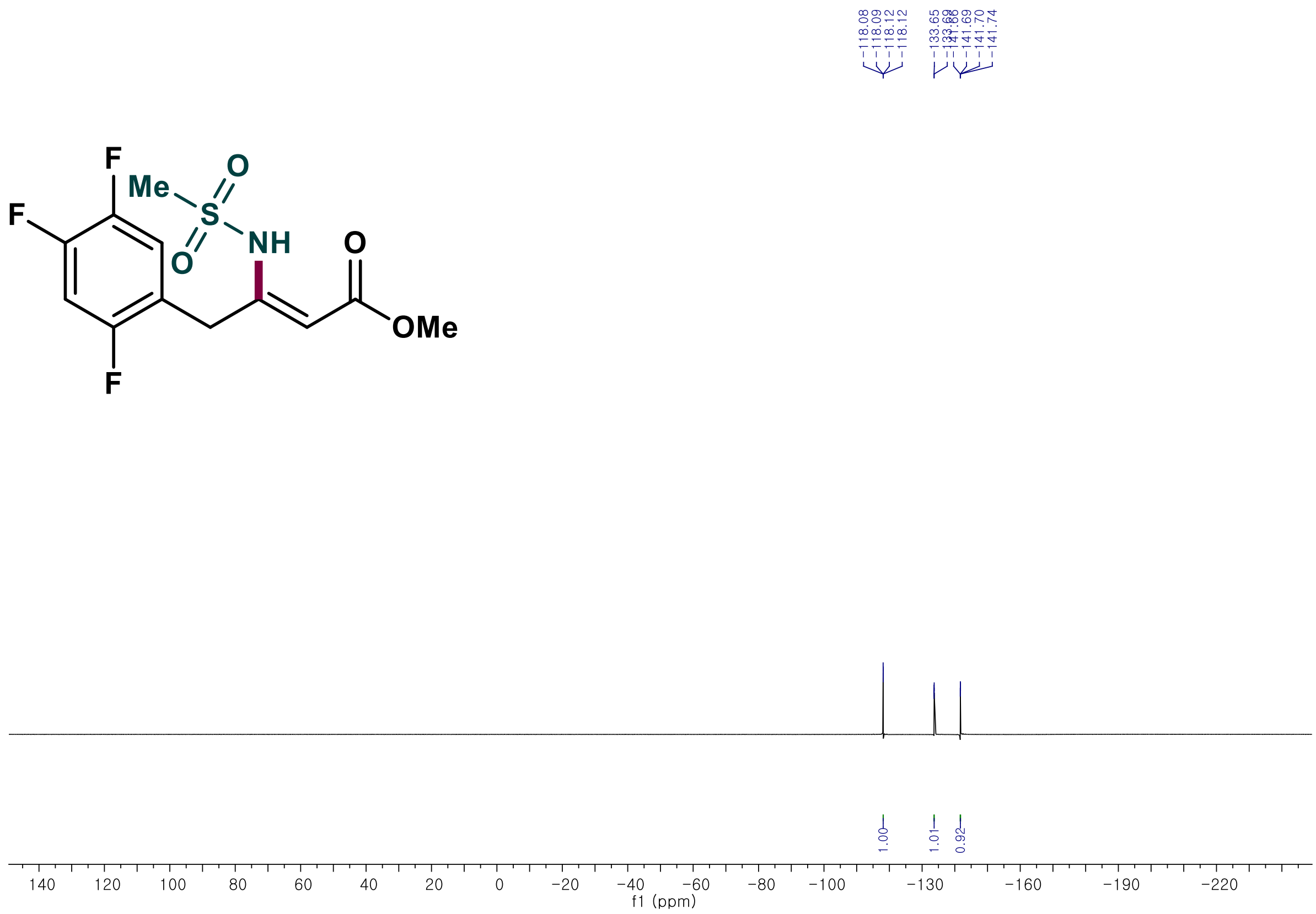

^{1}H NMR spectra (**1a**)

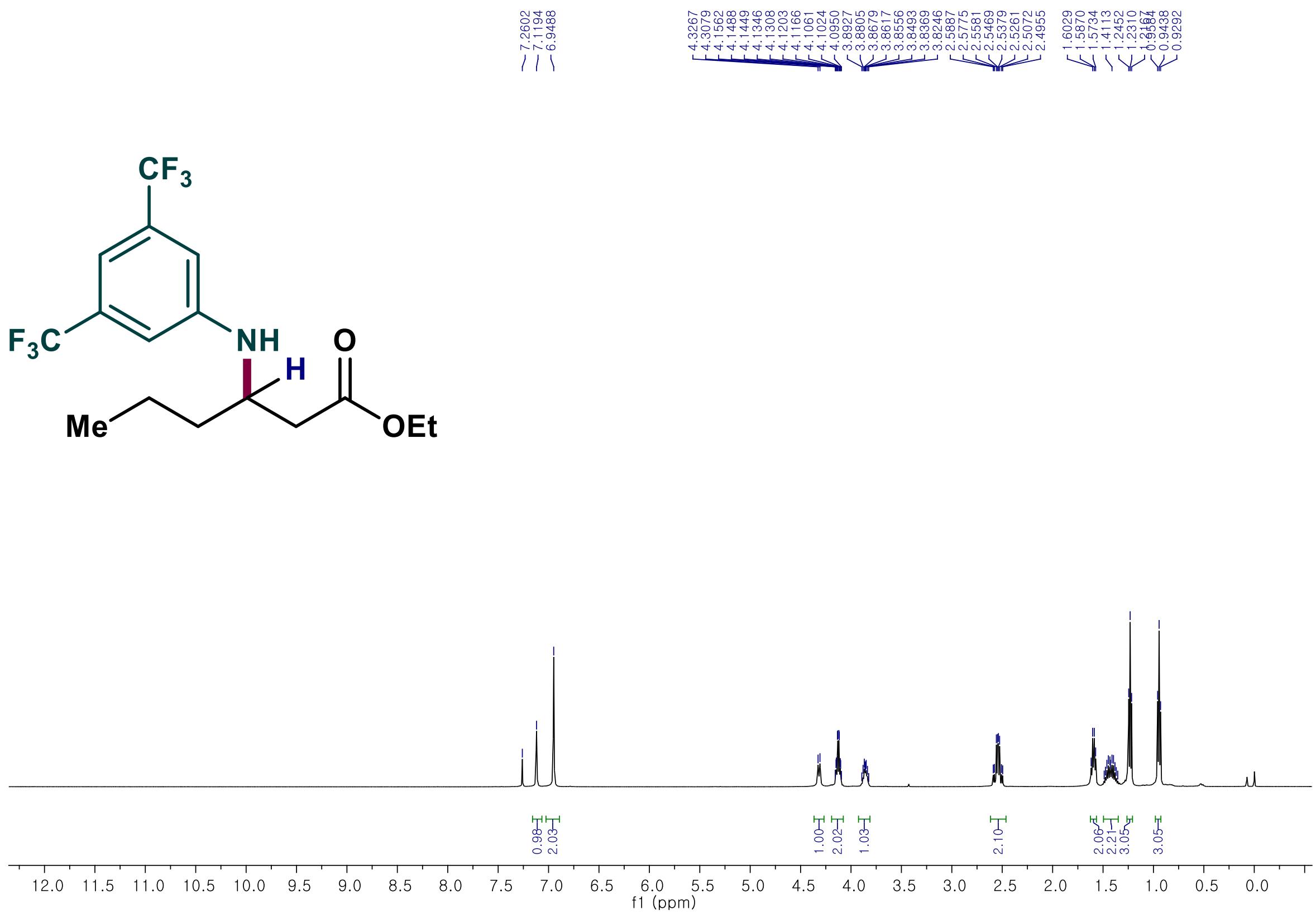

^{13}C NMR spectra (**1a**)

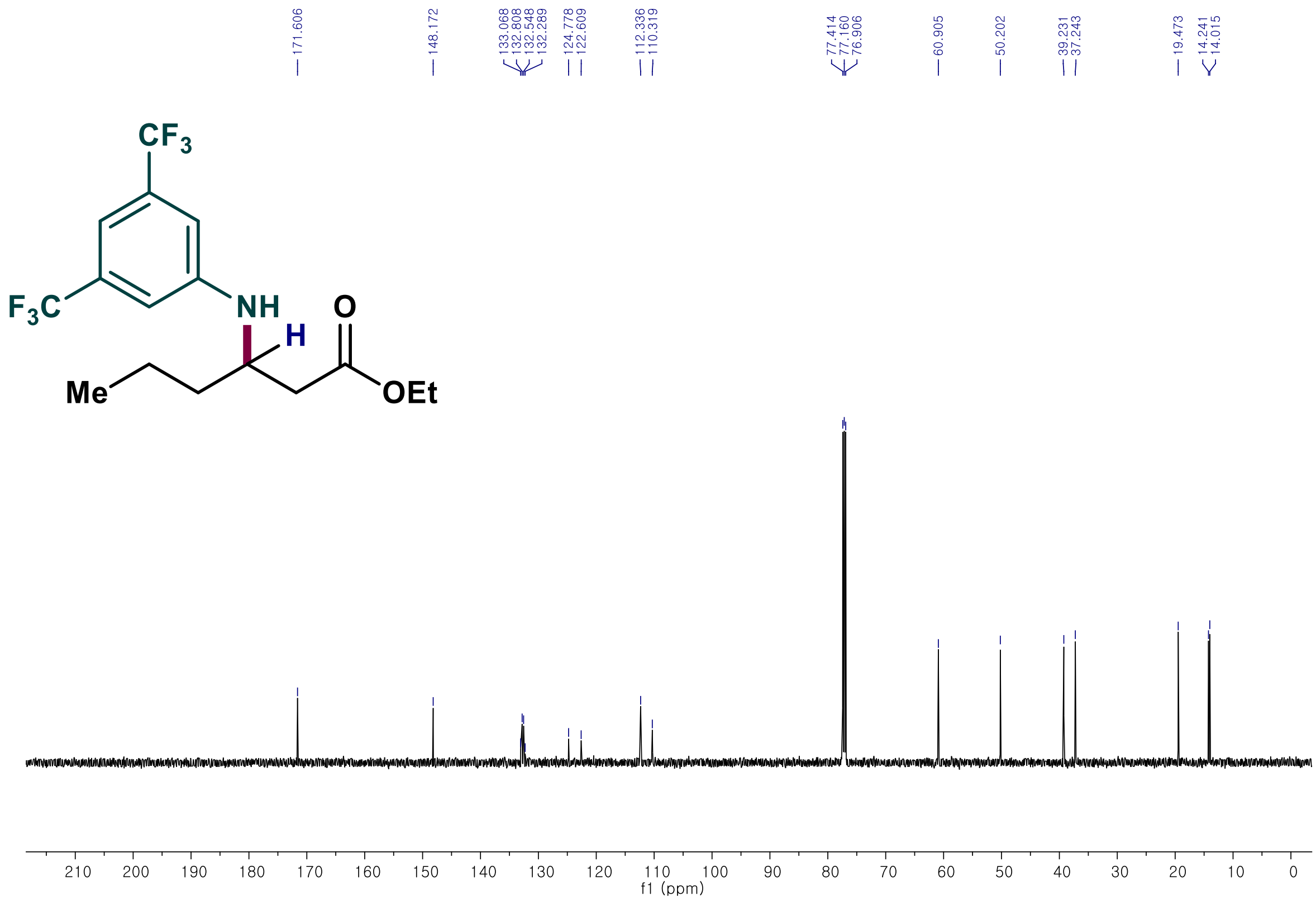

^{19}F NMR spectra (**1a**)

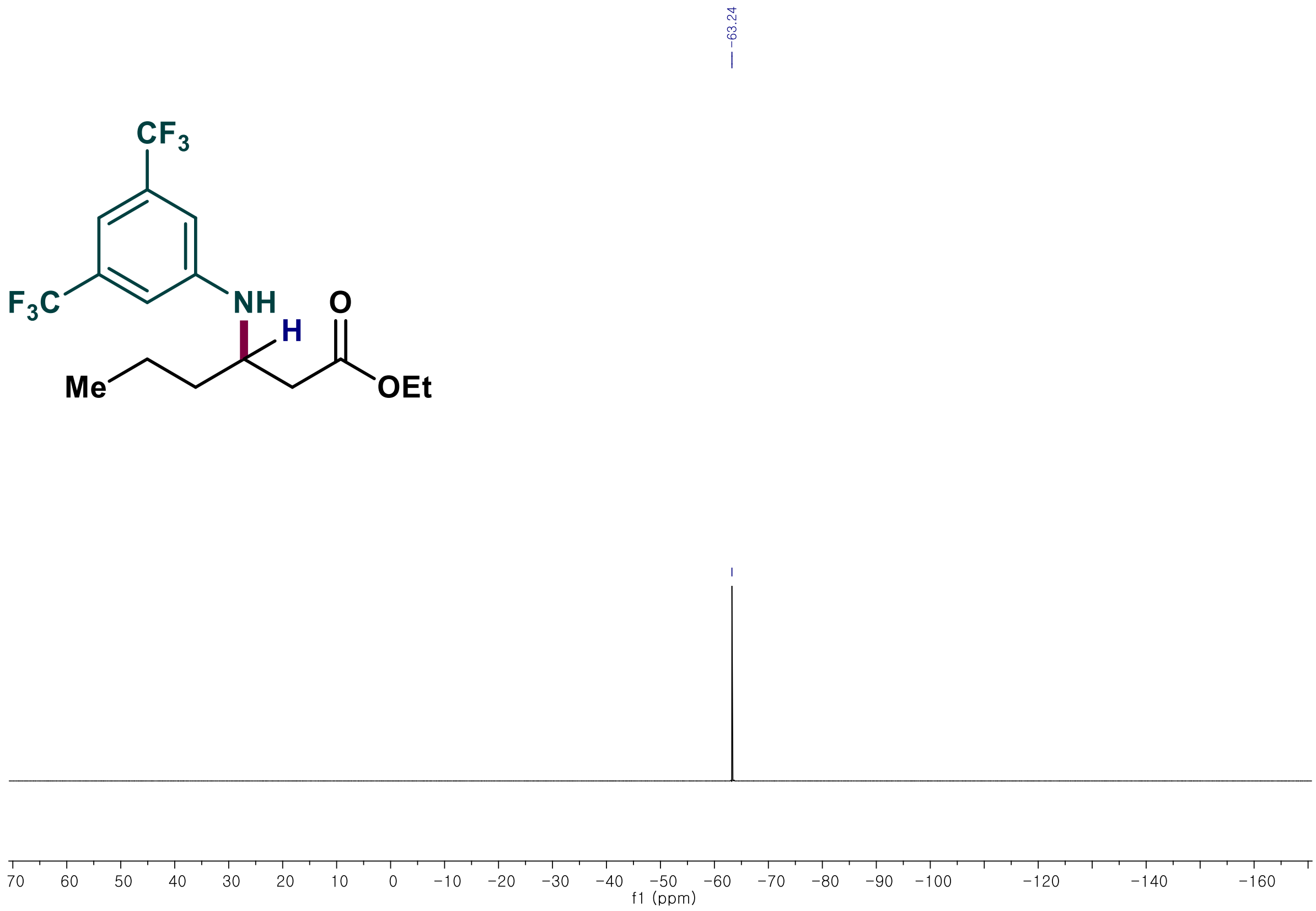

^{1}H NMR spectra (**1b**)

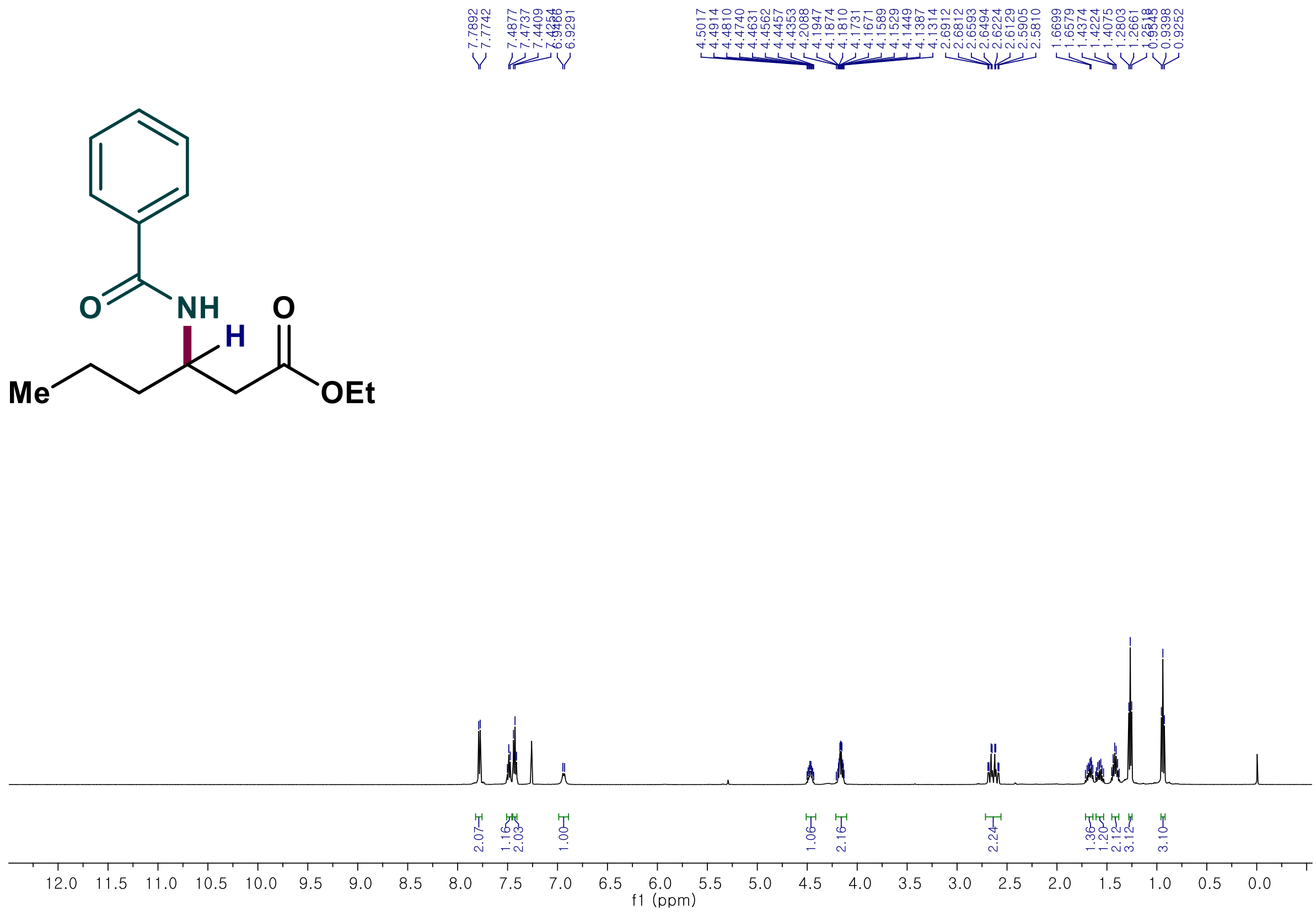

^{13}C NMR spectra (**1b**)

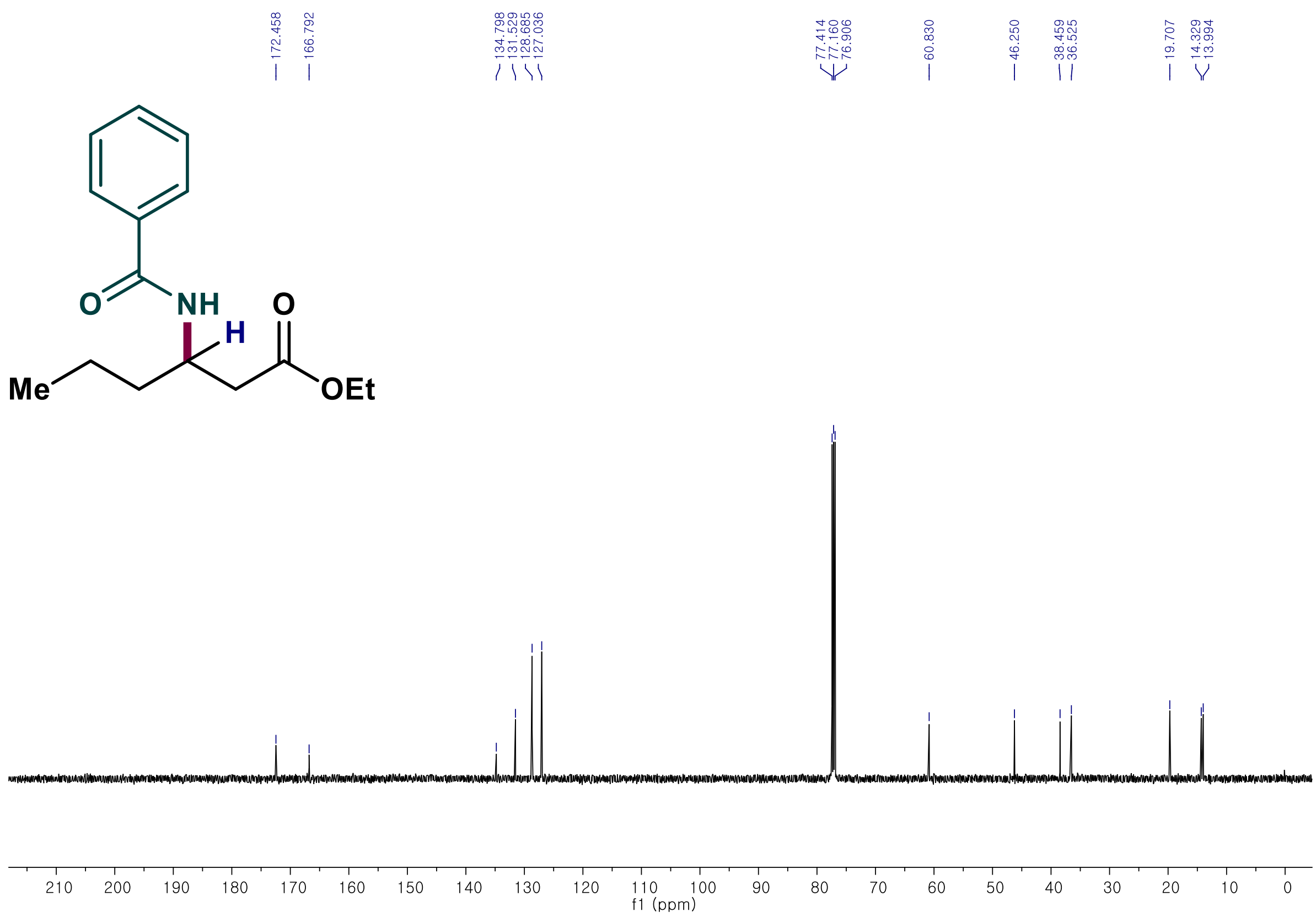

^{1}H NMR spectra (**1c**)

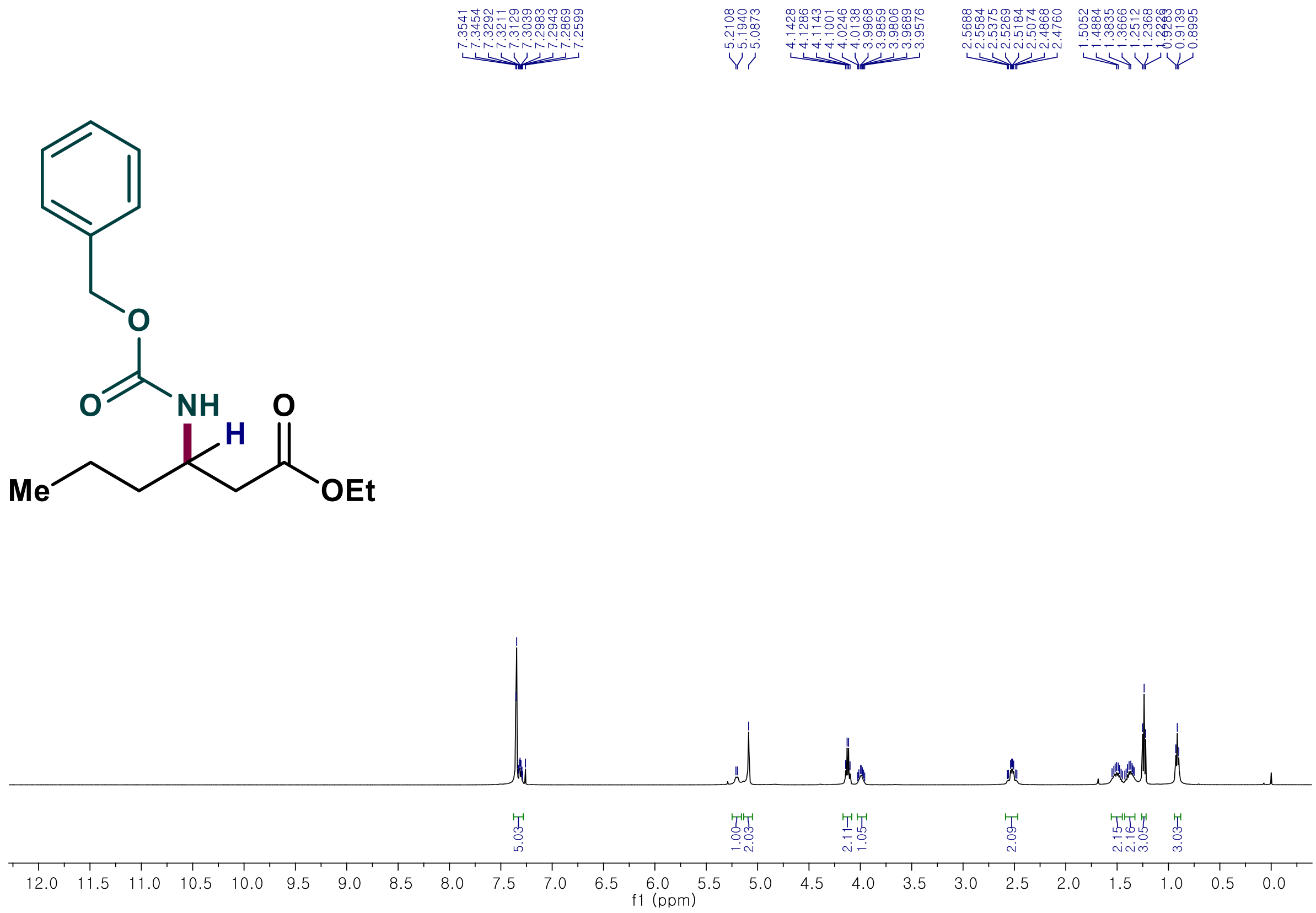

^{13}C NMR spectra (**1c**)

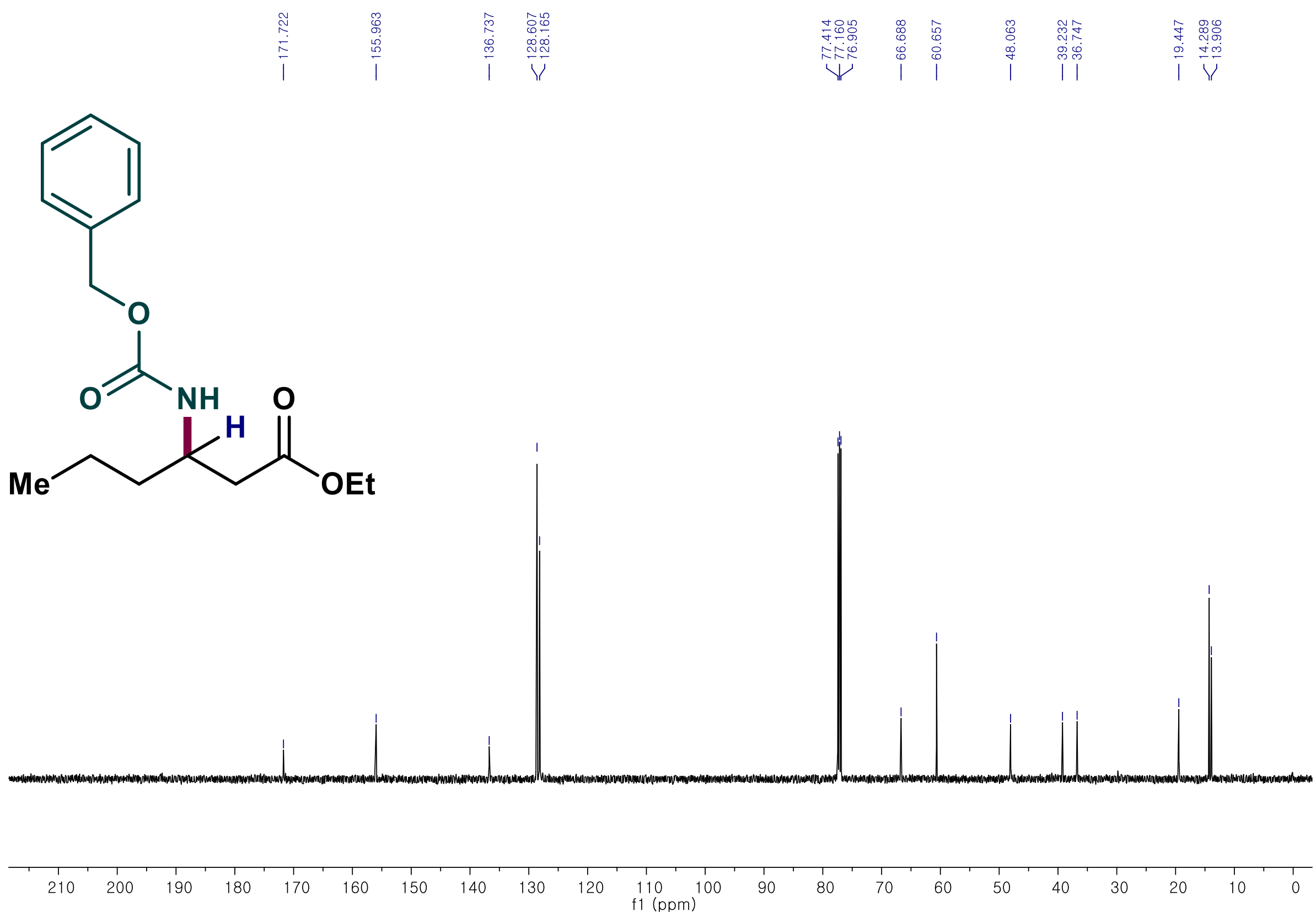

^{1}H NMR spectra (**1d**)

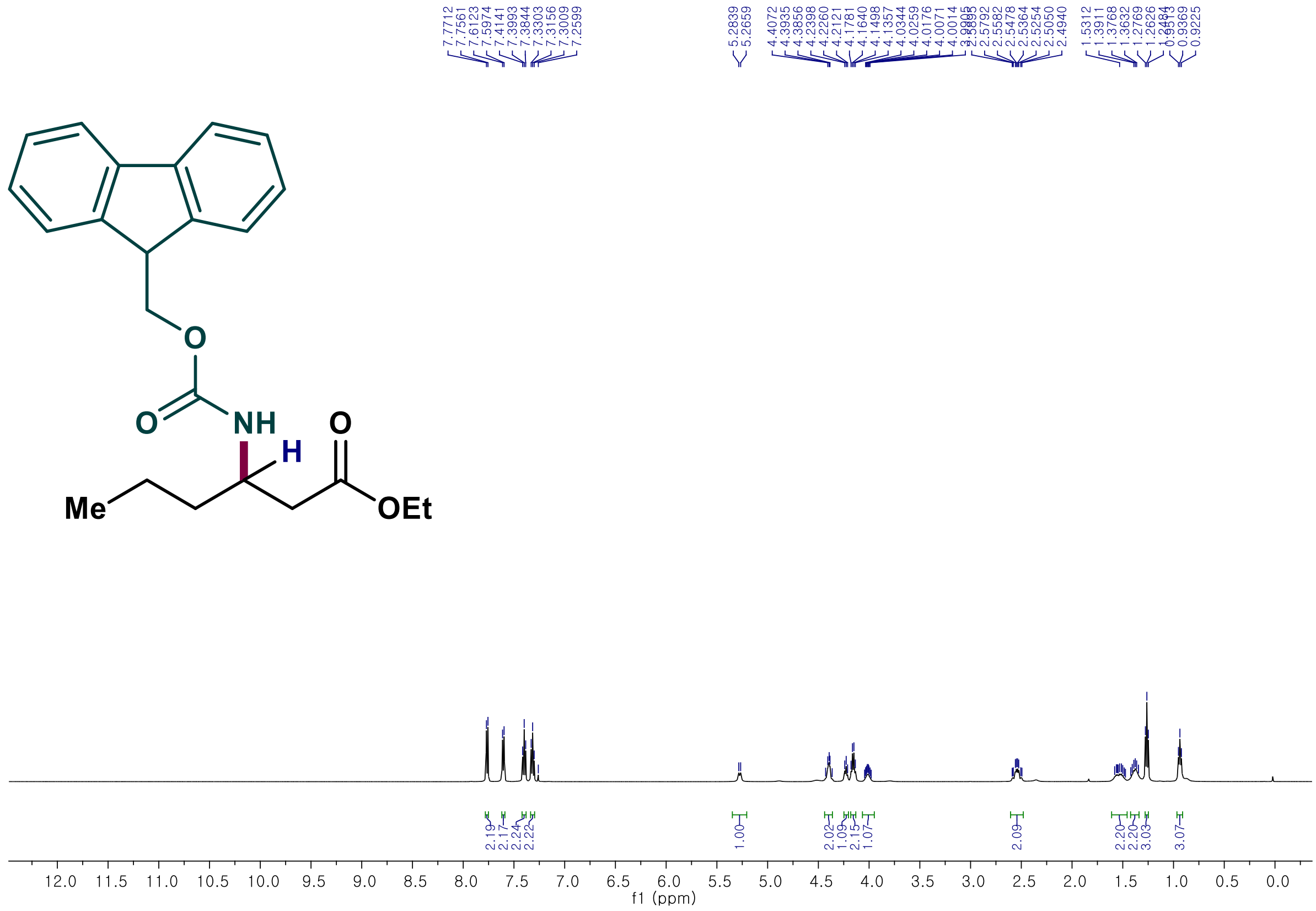

^{13}C NMR spectra (**1d**)

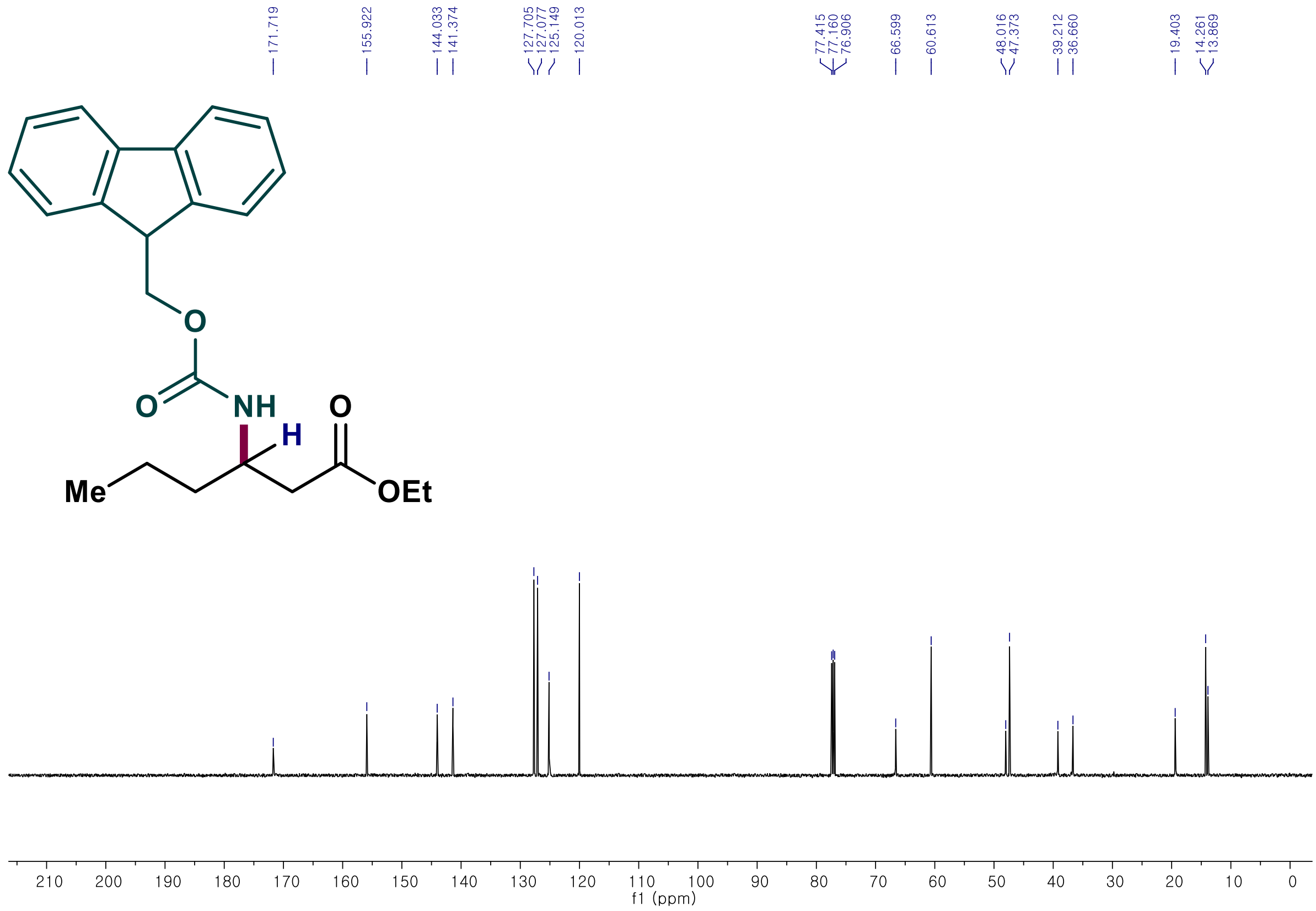

^{1}H NMR spectra (**Trityl tetrakis(3,5-bis(trifluoromethyl)phenyl)borate**)

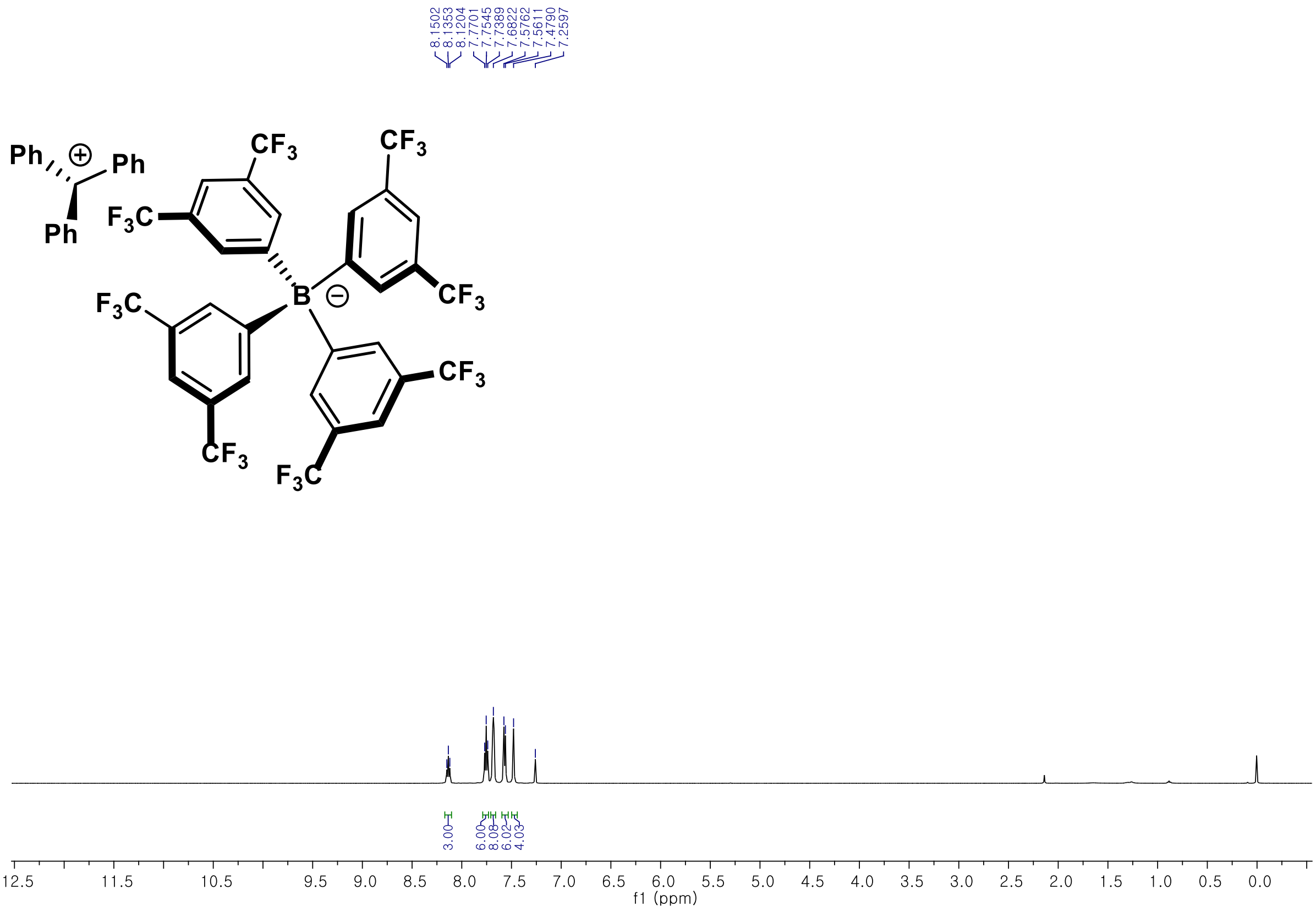

^{13}C NMR spectra (**Trityl tetrakis(3,5-bis(trifluoromethyl)phenyl)borate**)

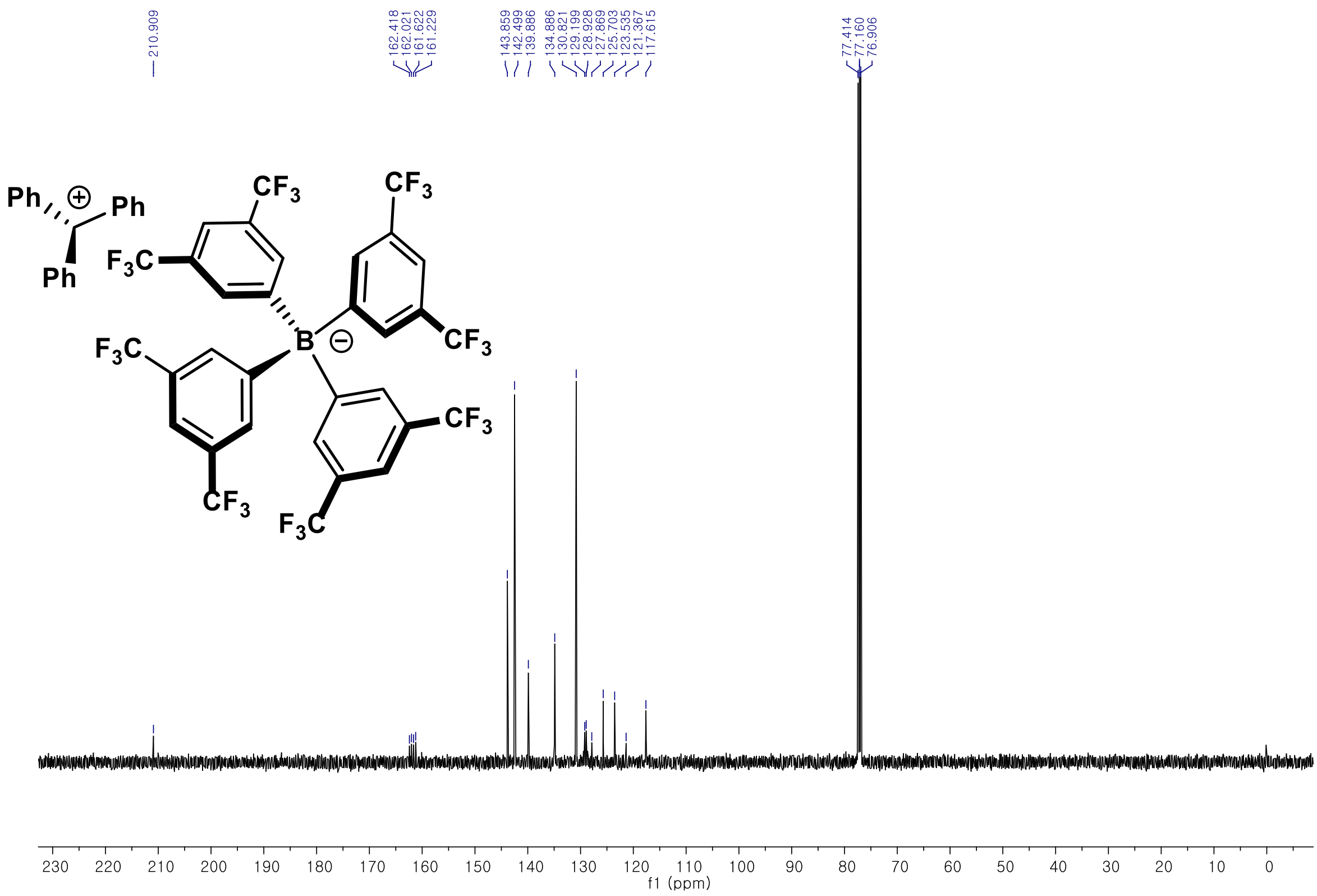

^{19}F NMR spectra (**Trityl tetrakis(3,5-bis(trifluoromethyl)phenyl)borate**)

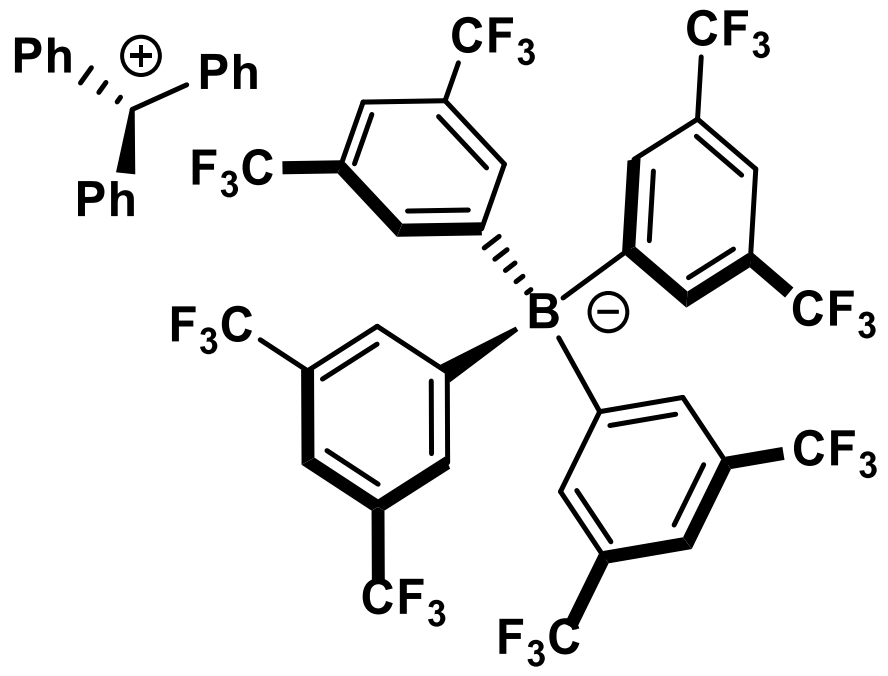

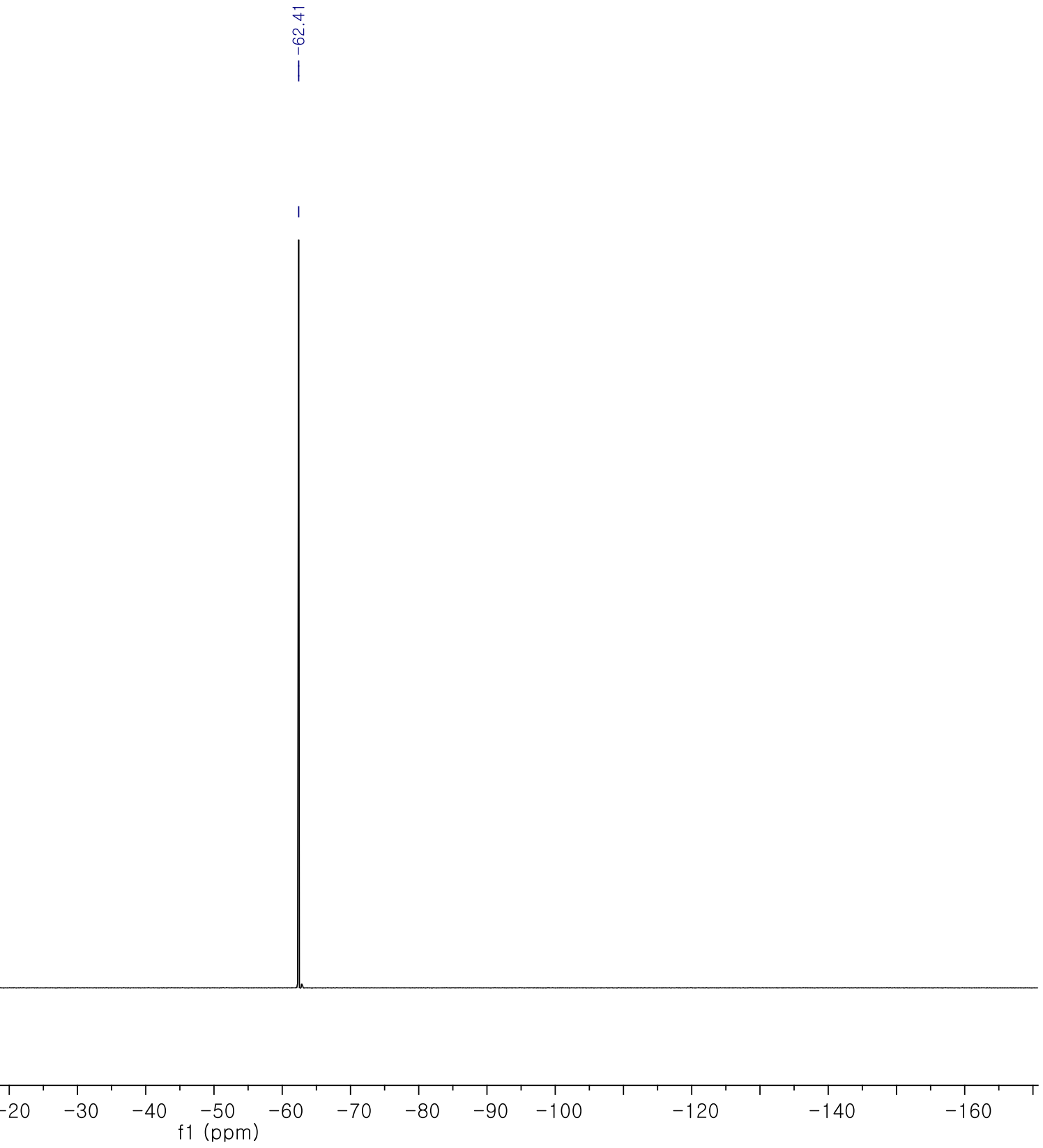

8. NMR experiment

^{1}H NMR spectra in $CDCl_3$ **(Crude mixture of $[Ph_3C^+B(C_6F_5)_4^-]$: $[Et_2SiH_2]$)**

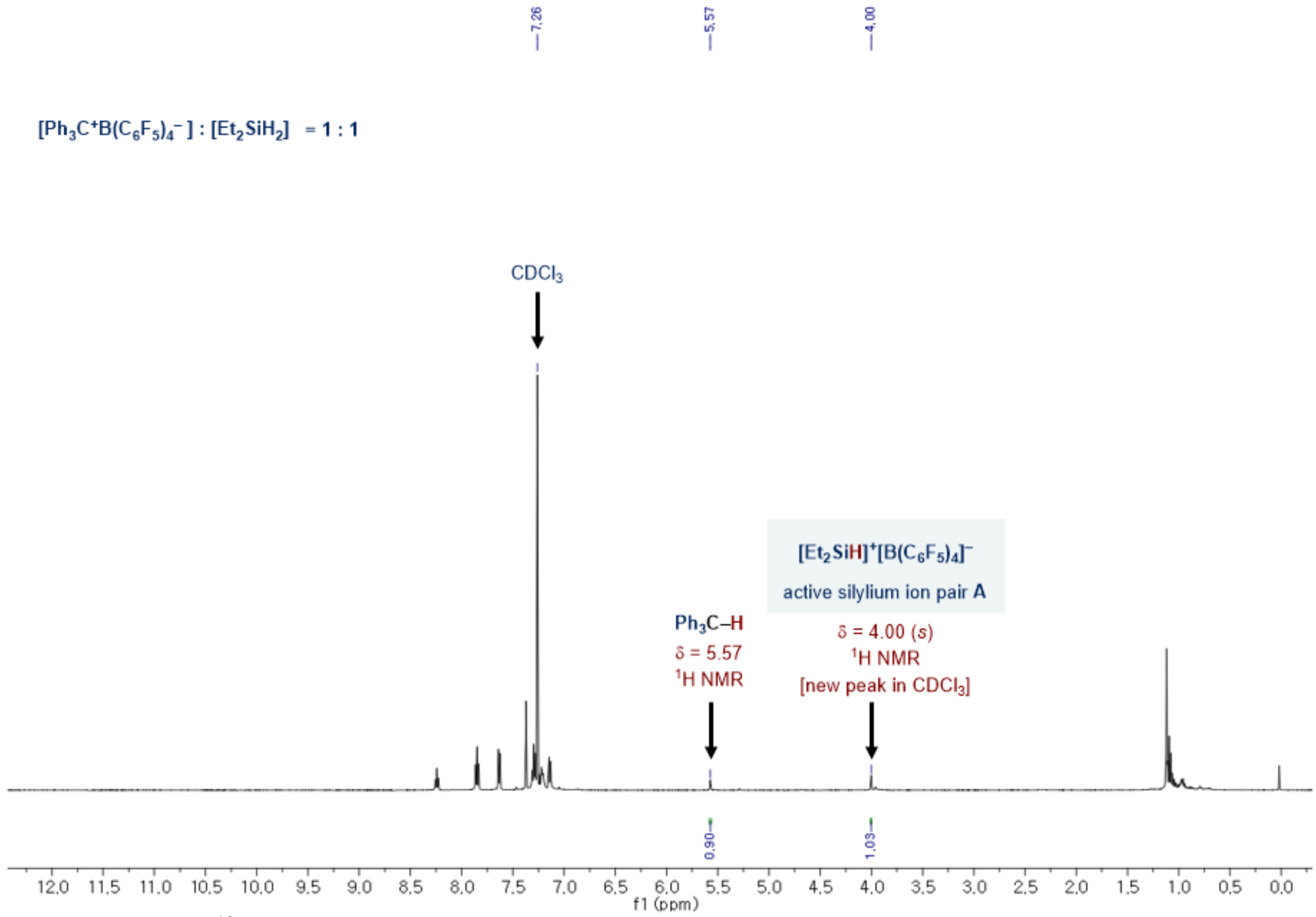

^{13}C NMR spectra in $CDCl_3$ **(Crude mixture of $[Ph_3C^+B(C_6F_5)_4^-]$: $[Et_2SiH_2]$)**

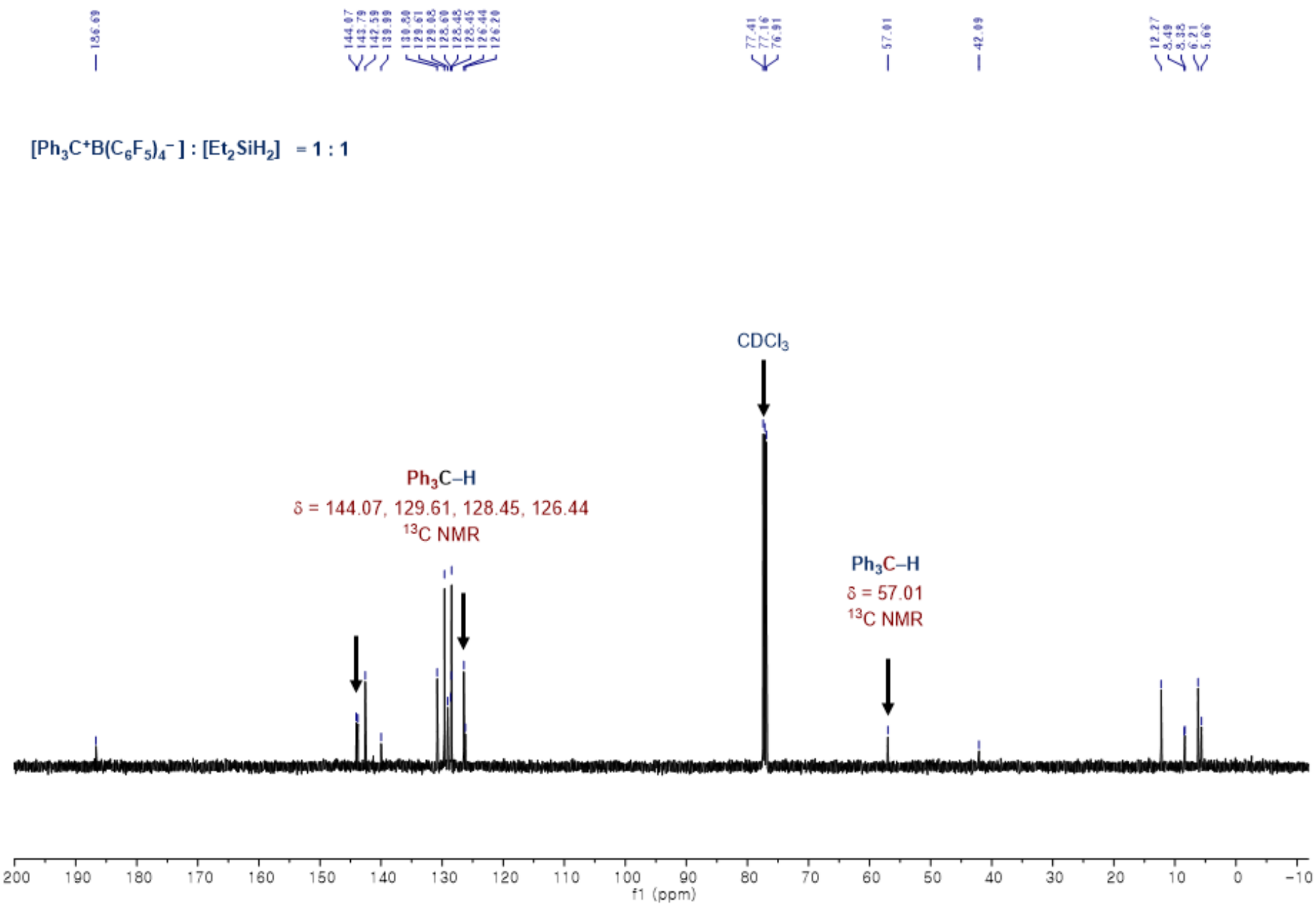

9. Computational Studies on Mechanistic Investigation

Density functional theory (DFT) calculation was conducted at the B3LYP/def2SVP level.[43,44] To further investigate the details of the silylium catalyst and provide theoretical insights into the catalyst structure, computational studies were conducted using the larger def2-TZVP basis set. All calculations were implemented at 353.15 K without solvent, the same as the experimental condition. All geometries were optimized and to confirm the stationary points of the optimization structures, vibration frequency analysis was involved. All calculations were performed with Gaussian 16 software.[45]

9.1. Cartesian coordinate of the Figure 4 [def2-SVP]

Path B : $[Et_3Si]^+[B(C_6F_5)_4]^-$, Ethyl 3-oxohexanoate, $TsNH_2$, Et_3SiH

$[Et_3Si]^+[B(C_6F_5)_4]^-$

Si	4.00583400	0.32893300	0.29712500
C	4.72533800	-1.39758600	0.26958300
C	4.62539200	1.43757900	1.66595200
H	4.62301400	0.85350000	2.60098600
C	3.89170200	2.77735000	1.84857400
H	5.69435900	1.61035000	1.43135900
H	5.79655200	-1.22697100	0.49887100
H	4.34961700	-1.94581500	1.14815000
C	4.57947300	-2.22306300	-1.01874900
H	5.04701700	-1.72091600	-1.88021800
H	3.52856700	-2.40683500	-1.27947900
H	5.07434900	-3.19950400	-0.90373600
H	3.94169500	3.40397900	0.94467100
H	2.82752200	2.63469600	2.08610100
H	4.34015000	3.35429100	2.67151100
C	-0.91221800	-1.37330500	-1.11493700
C	0.26924000	-1.83982600	-1.69681000
C	-2.04426200	-2.13008300	-1.44233800
C	0.36041400	-2.92541900	-2.56263100
C	-2.00923800	-3.22796100	-2.31245900

C	-0.79982600	-3.63512800	-2.87400500
C	-0.02580200	-0.86731000	1.24101800
C	1.29076900	-0.74858000	1.63683000
C	-0.71028800	-1.84289900	1.99283500
C	1.90991100	-1.38862700	2.70861400
C	-0.15897300	-2.54284500	3.06713700
C	1.17090500	-2.31693900	3.43385700
C	-2.29635900	0.50483200	0.43926300
C	-2.69125300	0.79747200	1.74921900
C	-3.19823500	0.93721500	-0.54308800
C	-3.89994100	1.42270600	2.07401800
C	-4.41501700	1.56190800	-0.26983000
C	-4.76998100	1.80960500	1.05705100
C	-0.08077700	1.23963400	-0.62675700
C	0.30153300	2.27043700	0.24223600
C	0.03003300	1.56776700	-1.98517700
C	0.76104100	3.52209900	-0.16917200
C	0.47338300	2.81418300	-2.44704800
C	0.84508100	3.80051300	-1.53461400
B	-0.83263300	-0.12084500	-0.02915500
F	-5.22895800	1.93459000	-1.25732500
F	-5.92239400	2.40927300	1.34603200
F	3.19118000	-1.14947600	3.02565300
F	2.16646300	0.15745800	0.92601500
F	1.71959200	-2.96715700	4.45183200
F	-0.88007800	-3.42965300	3.73915900
F	-1.97761100	-2.12813100	1.70036700
F	-2.90169100	0.75783400	-1.84049900
F	1.44283100	-1.21070900	-1.41580800
F	1.54065300	-3.30167800	-3.06747000

F	-0.75056400	-4.68267300	-3.69225900
F	-3.12358100	-3.90068200	-2.59217500
F	-3.24364000	-1.84805700	-0.92510500
F	-0.28865000	0.68893800	-2.94213000
F	0.55609800	3.05959100	-3.75408500
F	1.27920400	4.98614400	-1.95603100
F	1.13274900	4.44238400	0.72488700
F	0.24182000	2.07713100	1.57573600
F	-1.90716600	0.48794600	2.79850100
F	-4.21749700	1.66037100	3.34744600
C	3.68103600	1.13664400	-1.34836500
H	2.99167700	1.98140400	-1.19565000
H	3.15027700	0.41708000	-1.99079300
C	4.98181300	1.62377700	-2.02240800
H	4.75391100	2.08604500	-2.99503400
H	5.50578300	2.38165400	-1.41793300
H	5.68877600	0.80029500	-2.21427000

Ethyl 3-oxohexanoate

C	1.23473500	-0.54034200	0.11637000
O	1.51947400	-1.45785000	-0.61931700
C	-0.07652800	-0.57987600	0.91806900
H	0.10108500	-0.21275300	1.94313400
H	-0.43351700	-1.61578500	0.96085200
C	-1.14891000	0.31242900	0.31126200
O	-0.97811200	1.45401600	-0.04724500
C	2.09955300	0.69317800	0.28860800
H	1.45063300	1.54965300	0.03070800
H	2.30437000	0.81308700	1.36987300
C	3.39430000	0.69419400	-0.52569200

H	3.15236600	0.44653000	-1.57250100
H	3.79699100	1.72129300	-0.53349900
C	4.46280300	-0.27068800	-0.00617500
H	4.10440400	-1.31052400	-0.03517100
H	5.37824700	-0.21493800	-0.61674100
H	4.74481400	-0.03396700	1.03427800
O	-2.32540700	-0.32251300	0.23234000
C	-3.43328300	0.42318500	-0.31070500
H	-3.16635600	0.76460300	-1.32369800
H	-3.58815200	1.32535200	0.30303700
C	-4.64742000	-0.48113100	-0.31419800
H	-4.89718300	-0.81152600	0.70580500
H	-5.51498600	0.05852600	-0.72460000
H	-4.47185900	-1.37446400	-0.93294500

$TsNH_2$

C	-2.68842200	-0.00139900	0.03364200
C	-1.96068500	-1.20552600	-0.00588600
C	-0.56881300	-1.20748300	-0.06069700
C	0.11193000	0.01436300	-0.06930400
C	-0.58003800	1.22504900	-0.04192100
C	-1.97638600	1.20733500	0.01222800
H	-2.49716900	-2.15870000	-0.00010300
H	-0.00797100	-2.14260200	-0.10937400
H	-0.02687000	2.16518100	-0.07589300
H	-2.52251900	2.15433100	0.03244400
C	-4.19594100	-0.02235400	0.08013900
H	-4.61448500	-0.44716700	-0.84796700
H	-4.56107300	-0.64690500	0.91176400
H	-4.61281800	0.98751900	0.20444400

N	2.36186000	-0.21066400	1.47153500
H	3.04750200	-0.96534400	1.51278900
H	2.73139100	0.65534400	1.86412400
O	2.32141700	1.36597400	-0.54888500
O	2.33385800	-1.18344800	-0.85344500
S	1.90243300	0.02205500	-0.14147100

Et_3SiH

Si	0.15933400	-0.20441100	0.24563500
H	0.12214400	-0.10210200	1.74382400
C	-1.13970400	-1.48056800	-0.30641700
C	-2.58523900	-1.26545900	0.16638900
H	-0.77789000	-2.46395200	0.04739100
H	-1.10709000	-1.54004500	-1.41043400
H	-3.24276500	-2.09447300	-0.14480000
H	-2.64715300	-1.19414800	1.26500300
H	-3.01865600	-0.33930000	-0.24393500
C	1.87444500	-0.84041400	-0.27711400
C	3.07545500	-0.02045400	0.21703900
H	1.89130000	-0.90993600	-1.38086500
H	1.96019000	-1.88233400	0.08230500
H	4.03316600	-0.46257900	-0.10490300
H	3.05309000	1.01339900	-0.16561300
H	3.09781900	0.04172800	1.31781100
C	-0.13851100	1.53238000	-0.47591700
H	-0.26446900	1.42771500	-1.56986400
H	0.80197700	2.09837600	-0.34522400
C	-1.30297300	2.34104600	0.11671300
H	-1.36632000	3.34875900	-0.32719100
H	-2.27538000	1.85164400	-0.05256400

H	-1.19148300	2.46981100	1.20601000

Path A : $[Et_2SiH]^+[B(C_6F_5)_4]^-$, Et_2SiH_2, Ethyl 3-oxohexanoate, $TsNH_2$

Ethyl 3-oxohexanoate and $TsNH_2$ are the same as the structures of Path B

$[Et_2SiH]^+[B(C_6F_5)_4]^-$

Si	3.82636000	0.77899300	-0.13479700
H	3.19563600	1.23478800	-1.38973700
C	4.53515600	-0.94735200	-0.14088700
C	4.70265800	2.10353000	0.83438700
H	4.92018400	1.69069700	1.83496200
C	3.98396900	3.45999600	0.93102300
H	5.68946600	2.21622100	0.34239600
H	5.34942400	-0.97833600	0.60274500
H	3.75704800	-1.63838600	0.22324400
C	5.01682100	-1.40555600	-1.53215700
H	5.74978300	-0.71235200	-1.97633300
H	4.16986100	-1.50769300	-2.22571100
H	5.50448800	-2.38941100	-1.45829000
H	3.81682800	3.90730500	-0.06106400
H	3.00154100	3.37113000	1.41746300
H	4.58190100	4.17291600	1.51859500
C	-0.61228300	-1.45347900	-0.98366100
C	0.60808200	-1.83015800	-1.55002200
C	-1.65579700	-2.35309300	-1.23537300
C	0.81201000	-2.95856900	-2.33820300
C	-1.50679400	-3.50173900	-2.02419000
C	-0.26588100	-3.81101500	-2.57982000
C	0.23676500	-0.65667900	1.29850500
C	1.53161400	-0.34592600	1.64439600

C	-0.27763600	-1.68030800	2.12063500
C	2.30110600	-0.86579300	2.68232700
C	0.41701000	-2.25866500	3.18505900
C	1.72828800	-1.85748000	3.47041400
C	-2.18991200	0.37240000	0.44016700
C	-2.60733200	0.69466000	1.73545600
C	-3.14050600	0.63443200	-0.55582800
C	-3.87860000	1.19472100	2.03736700
C	-4.41988300	1.12971700	-0.30515400
C	-4.79292800	1.41533400	1.00942200
C	-0.06366200	1.25789300	-0.73285700
C	0.21317100	2.39486800	0.03832300
C	0.06043300	1.45986500	-2.11457000
C	0.61625200	3.62367700	-0.48449100
C	0.45606700	2.67670900	-2.68639400
C	0.74269900	3.76690800	-1.86687900
B	-0.67279600	-0.11078400	-0.00706700
F	-5.27822800	1.34344800	-1.30202500
F	-6.00557800	1.89393100	1.27638000
F	3.55882300	-0.45274200	2.88310000
F	2.24833300	0.68072100	0.90337500
F	2.40734100	-2.40817300	4.46740800
F	-0.14530200	-3.20613600	3.92205500
F	-1.50590800	-2.14302100	1.90121100
F	-2.83092100	0.40487900	-1.84226100
F	1.70931900	-1.05469800	-1.34206800
F	2.01911400	-3.23169100	-2.84641300
F	-0.10917200	-4.90170400	-3.32419400
F	-2.54153900	-4.31191800	-2.23489000
F	-2.87395600	-2.16640800	-0.71989800

F	-0.19200400	0.47882600	-2.98813200
F	0.56561000	2.79710100	-4.00855500
F	1.13915600	4.92452600	-2.39030800
F	0.90486000	4.64815900	0.32292300
F	0.12414900	2.32821700	1.38110400
F	-1.78431500	0.54147800	2.78917300
F	-4.21495300	1.47162200	3.29782400

Et_2SiH_2

Si	0.00000000	-0.43458000	0.00000800
H	-0.00000800	-1.32472800	1.20557500
H	0.00000800	-1.32472900	-1.20555900
C	-1.56912400	0.63210000	-0.00000700
C	-2.88796100	-0.15514000	-0.00000400
H	-1.52442100	1.29910100	-0.88034200
H	-1.52443200	1.29912400	0.88031000
H	-3.76341800	0.51536700	-0.00001800
H	-2.97326200	-0.80429200	-0.88723100
H	-2.97327200	-0.80426700	0.88724000
C	1.56912400	0.63210000	0.00001300
C	2.88796100	-0.15514000	-0.00001400
H	1.52443600	1.29909800	0.88035000
H	1.52441600	1.29912700	-0.88030100
H	3.76341800	0.51536700	-0.00001000
H	2.97327600	-0.80429500	0.88720900
H	2.97325800	-0.80426400	-0.88726100

I_B

Si	-4.14043400	1.93701000	-0.12590300
C	-3.45794900	2.25168700	1.59318100
C	-2.90977200	1.57217300	-1.48532000
H	-2.25643300	2.46338800	-1.53324600
C	-3.50280500	1.26402100	-2.87038800
H	-2.23188000	0.77077800	-1.14909100
H	-4.31331200	2.24658400	2.29380500
H	-2.81623900	1.40636500	1.88360700
C	-2.66459600	3.56716400	1.73749200
H	-3.27757300	4.45154900	1.50292700
H	-1.77592100	3.58537100	1.08981000
H	-2.31202100	3.68499100	2.77381300
H	-4.11253900	0.34577000	-2.86352200
H	-4.14280100	2.08219100	-3.23720900
H	-2.70568400	1.11639600	-3.61232700
C	2.36895700	1.71325700	-0.02803000
C	1.35611200	2.67819000	0.00959900
C	3.65878700	2.23933900	0.11366000
C	1.56820700	4.04850200	0.13383100
C	3.92489000	3.60926600	0.24121700
C	2.87367800	4.52387800	0.25707600
C	1.30984200	-0.09727400	1.45539100
C	-0.04350900	-0.16997900	1.77776600
C	2.15182200	-0.05498200	2.57806600
C	-0.53825600	-0.25681100	3.08068400
C	1.70847400	-0.13866200	3.89963900
C	0.34009300	-0.23590500	4.15877200
C	3.22173300	-0.93517000	-0.37783500
C	3.49976600	-2.12343900	0.30647200
C	4.01883800	-0.72515300	-1.51131700

C	4.51383000	-3.01302200	-0.06543700
C	5.04344100	-1.57896600	-1.91982300
C	5.29339500	-2.74006200	-1.18704800
C	0.91208100	-0.28238400	-1.30718300
C	0.26699500	-1.52540100	-1.31144500
C	0.68209000	0.48051500	-2.45909300
C	-0.58088500	-1.97381300	-2.32366000
C	-0.14732900	0.06353600	-3.50777000
C	-0.79195100	-1.16824400	-3.44194800
B	1.96192000	0.10050300	-0.06813900
F	5.77130800	-1.30703300	-3.00448700
F	6.26106100	-3.57774000	-1.55814100
F	-1.86809600	-0.32097800	3.30113300
F	-0.99955700	-0.15175900	0.81007100
F	-0.11624100	-0.30817000	5.40850900
F	2.57177700	-0.11014200	4.91359400
F	3.47335100	0.06620200	2.41090300
F	3.80650300	0.35626200	-2.27964700
F	0.05791800	2.29545400	-0.08235300
F	0.53389100	4.90105000	0.15626200
F	3.10907900	5.82899400	0.38517700
F	5.17897700	4.04488900	0.36375200
F	4.73483100	1.44495800	0.14202100
F	1.23941400	1.68557600	-2.62571900
F	-0.37056900	0.86374800	-4.55652600
F	-1.61897900	-1.55431600	-4.41474200
F	-1.20074000	-3.16014100	-2.22762800
F	0.43397800	-2.36928200	-0.27466200
F	2.78501000	-2.49577200	1.38576100
F	4.72986900	-4.12709400	0.63888200

C	-5.54887200	3.07053700	-0.64238400
H	-6.24551200	3.16316000	0.20991300
H	-6.11284700	2.54573500	-1.43369200
C	-5.12630200	4.46558200	-1.13983600
H	-6.00402600	5.05153600	-1.45426500
H	-4.61062500	5.04638300	-0.35969400
H	-4.44800200	4.40559400	-2.00587600
C	-5.08173800	-0.82029200	0.42373600
O	-5.12205100	0.37600700	0.04281900
C	-3.85483600	-1.38989000	1.05738900
H	-4.03479100	-1.42535800	2.14880800
H	-2.98761900	-0.73127800	0.93282900
C	-3.44290300	-2.81370400	0.63380300
O	-4.06157400	-3.52633500	-0.11938800
C	-6.29889600	-1.65077100	0.25711100
H	-5.96063300	-2.49234500	-0.38534000
H	-6.47984000	-2.15921200	1.22408700
C	-7.54813200	-0.95456600	-0.27751400
H	-7.81790900	-0.11997800	0.39143800
H	-7.31561300	-0.49756600	-1.25330300
C	-8.72749500	-1.91774400	-0.41885400
H	-9.00390300	-2.36357200	0.55076400
H	-9.61448100	-1.39746700	-0.81023900
H	-8.49168200	-2.74342900	-1.10956100
O	-2.30967500	-3.11932200	1.23553600
C	-1.70119500	-4.41254300	0.94022100
H	-1.37212200	-4.38709600	-0.10744700
H	-2.48152700	-5.18218500	1.04115700
C	-0.54986600	-4.62041100	1.89749900
H	-0.89159200	-4.59479100	2.94376100

H	-0.10048600	-5.60781400	1.70868000
H	0.23096500	-3.86026600	1.75523800

I_A

Si	3.35045200	1.38478100	0.48084900
H	2.51906200	0.34423000	-0.15407600
C	3.48391200	1.32352900	2.34253000
C	3.42519200	3.06888400	-0.34519500
H	4.41010300	3.48430300	-0.06197100
C	2.31112400	4.07940600	-0.01472500
H	3.46537400	2.90070600	-1.43631700
H	4.42450400	1.83368700	2.61928700
H	2.67184100	1.97280700	2.71141900
C	3.38989400	-0.06880200	2.98972900
H	4.19904500	-0.73778800	2.65984200
H	2.43633600	-0.56076900	2.74474400
H	3.44913700	0.01320700	4.08622500
H	1.34683300	3.76855300	-0.43602800
H	2.17365600	4.20989800	1.06891200
H	2.55913800	5.06212300	-0.44443300
C	-1.84599100	-1.40578800	0.72824700
C	-0.68986500	-1.86625300	1.36731300
C	-2.97024600	-2.21385600	0.93911100
C	-0.61299000	-3.03147100	2.12474000
C	-2.94363100	-3.39512000	1.69327800
C	-1.75535300	-3.81456300	2.28794100
C	-1.68436300	1.10851800	1.24082400
C	-0.54127600	1.78832100	1.65799900
C	-2.76884500	1.25048800	2.12125900
C	-0.46737000	2.58725800	2.80104200

C	-2.74935300	2.03402300	3.27650300
C	-1.57984700	2.71130100	3.62727400
C	-3.14700100	0.35917500	-1.00946400
C	-3.89832800	1.53936800	-1.03747100
C	-3.50587900	-0.57521500	-1.99001500
C	-4.95229900	1.76334200	-1.93106600
C	-4.54790300	-0.39886700	-2.89976700
C	-5.28018000	0.78866300	-2.87073800
C	-0.57626700	0.19194100	-1.15851700
C	-0.23408100	1.44897900	-1.67664000
C	0.13955000	-0.87091000	-1.72270600
C	0.78401300	1.66761800	-2.60576200
C	1.15535200	-0.70181400	-2.67079200
C	1.50027100	0.57727400	-3.09534800
B	-1.82304500	0.06326700	-0.05484500
F	-4.84021900	-1.34081600	-3.79934600
F	-6.27716200	0.98773000	-3.73205400
F	0.67813300	3.21768400	3.11866400
F	0.61955200	1.71286200	0.94735600
F	-1.52986800	3.46082200	4.72624200
F	-3.82635200	2.13031100	4.05405100
F	-3.91664100	0.61111200	1.86606100
F	-2.82249900	-1.72979700	-2.08785000
F	0.46754600	-1.16727900	1.25649600
F	0.55246600	-3.41302900	2.67234600
F	-1.70957700	-4.93796400	3.00293100
F	-4.04958400	-4.12294800	1.84706200
F	-4.16260500	-1.89437900	0.42609100
F	-0.09556100	-2.14339700	-1.36653900
F	1.84387200	-1.75232100	-3.13777700

F	2.53477800	0.75653600	-3.92797800
F	1.11492300	2.90600100	-2.99002500
F	-0.87102700	2.54825100	-1.24372100
F	-3.64034500	2.55098800	-0.19353600
F	-5.63571400	2.90932900	-1.89827600
C	5.56879800	-0.13900400	-0.66616400
O	5.05122400	0.79396300	-0.01633300
C	4.73481000	-1.16916500	-1.37306300
H	5.32929800	-1.68151200	-2.14418400
H	3.86302900	-0.72331900	-1.87108100
C	4.27370700	-2.22619000	-0.35101200
O	4.79201200	-2.33978300	0.73705500
C	7.04976900	-0.28856700	-0.65860600
H	7.17705000	-1.23674800	-0.09212900
H	7.37489300	-0.53883400	-1.68476100
C	7.87193300	0.84558800	-0.04604400
H	7.45938900	1.09453500	0.94471900
H	8.88725300	0.45779200	0.13394800
C	7.94926500	2.09876400	-0.92169300
H	6.95530700	2.53845400	-1.10082600
H	8.56825800	2.87166100	-0.44147600
H	8.40086400	1.87600100	-1.90242300
O	3.29719900	-2.95245100	-0.84024000
C	2.70799300	-3.99365900	-0.00187100
H	2.09615000	-3.49136900	0.76062900
H	3.52470000	-4.52524400	0.50837400
C	1.88420000	-4.88950500	-0.89799300
H	2.51559600	-5.40360500	-1.63886700
H	1.37938200	-5.65145800	-0.28459100
H	1.11989100	-4.30380300	-1.42704400

II_B

Si	3.43734000	3.62273600	-0.67631400
C	2.73350500	3.18233800	-2.35967200
C	2.18078500	3.84520700	0.69220600
H	1.54744100	4.68175800	0.33811700
C	2.72274300	4.15341500	2.09870900
H	1.49878900	2.97909600	0.70493500
H	3.54187900	3.31527500	-3.10086800
H	2.48652500	2.11025900	-2.36226300
C	1.48404500	3.98897200	-2.76845800
H	1.64759000	5.07820000	-2.72837900
H	0.62185800	3.74849100	-2.12834800
H	1.19091000	3.74410000	-3.80107400
H	3.31786400	3.31982500	2.50148400
H	3.35561300	5.05542500	2.10208400
H	1.89515100	4.32910800	2.80069900
C	-3.54524300	0.70935700	-1.30171000
C	-3.13183100	1.70826200	-2.19246900
C	-4.77080800	0.11865200	-1.62886000
C	-3.86957800	2.13643400	-3.29452700
C	-5.54858600	0.51351200	-2.72449500
C	-5.09595600	1.52794500	-3.56539500
C	-1.31396200	-0.51587900	-0.97342200
C	-0.05442500	-0.00115400	-1.28387700
C	-1.59544700	-1.73581400	-1.61007700
C	0.86438600	-0.64322700	-2.11830000
C	-0.70576600	-2.42025900	-2.43934700
C	0.54527200	-1.86447400	-2.69782000
C	-3.19857000	-0.75818800	1.05837400

C	-2.62954900	-1.92437500	1.57469600
C	-4.37659900	-0.36600600	1.71020900
C	-3.18594100	-2.67514500	2.61344000
C	-4.97611000	-1.08328700	2.74705900
C	-4.37337800	-2.25598700	3.20704700
C	-1.97923000	1.49847400	0.85700200
C	-0.91719000	1.29885700	1.74596500
C	-2.58961800	2.75456200	0.97385000
C	-0.45647600	2.25286100	2.64987400
C	-2.16196300	3.74556200	1.86892500
C	-1.08551800	3.49472200	2.71614900
B	-2.51686700	0.23523000	-0.08782600
F	-6.10610000	-0.65499400	3.31028600
F	-4.92041000	-2.95493100	4.20013500
F	2.07045100	-0.09355800	-2.35689400
F	0.38466600	1.17664700	-0.77466800
F	1.43708200	-2.50252500	-3.47310300
F	-1.03185800	-3.60219100	-2.96756000
F	-2.77972100	-2.33121300	-1.41819800
F	-4.99055700	0.76848600	1.34411000
F	-1.94566400	2.32390200	-1.99857200
F	-3.41252000	3.10546100	-4.09275700
F	-5.82139200	1.91153300	-4.61506800
F	-6.71386200	-0.08443300	-2.97742100
F	-5.27659300	-0.88599600	-0.90035600
F	-3.64013200	3.09252800	0.21997500
F	-2.77848100	4.92646200	1.91629300
F	-0.64100500	4.43235400	3.55669000
F	0.60768200	1.99951700	3.43257000
F	-0.25072100	0.12411000	1.74896300

F	-1.46050700	-2.40396700	1.09842200
F	-2.57085200	-3.78642900	3.04386200
C	4.77026500	4.94758700	-0.70287000
H	5.53784800	4.63537800	-1.43303100
H	5.27251800	4.94814200	0.28055600
C	4.26046700	6.36137700	-1.03698600
H	5.08611600	7.09012300	-1.03202800
H	3.79437300	6.40665200	-2.03435500
H	3.51343300	6.71323000	-0.30725700
C	4.55953100	1.04809700	0.25864500
O	4.50576700	2.18970400	-0.26178500
C	3.33744200	0.29655700	0.68361500
H	3.38958600	-0.70035600	0.21732600
H	2.41253900	0.78207200	0.34286400
C	3.27017200	0.14584100	2.20811900
O	3.88226500	0.85327200	2.97444100
C	5.88844400	0.42456200	0.46781100
H	5.99979500	0.43234300	1.57347600
H	5.79809300	-0.65351000	0.23332200
C	7.08397800	1.09363300	-0.20936000
H	6.91720200	1.11010100	-1.30032500
H	7.14406200	2.14844000	0.10458300
C	8.39599500	0.37438100	0.10736500
H	8.36731900	-0.67808200	-0.21785900
H	9.24403900	0.86035200	-0.39839700
H	8.60437600	0.37945600	1.18968200
O	2.43917200	-0.82294900	2.54781600
C	2.11102300	-0.96632700	3.95797600
H	1.65970700	-0.02038700	4.28836200
H	3.04850600	-1.11382900	4.51554400

C	1.15848600	-2.13204300	4.09967600
H	1.63359300	-3.07913100	3.80136800
H	0.84879800	-2.22469100	5.15200600
H	0.25949700	-1.97798900	3.48555900
C	0.39679300	-5.12091100	-0.23434900
C	0.96971300	-4.22666800	0.68469300
C	2.25615700	-3.71990500	0.49761400
C	2.98422200	-4.12540500	-0.62308000
C	2.46233500	-5.05281900	-1.53378000
C	1.17114600	-5.53671600	-1.33440000
H	0.38499600	-3.90054400	1.54529400
H	2.68441700	-3.01301500	1.20889200
H	3.06272800	-5.38790500	-2.38197200
H	0.74975400	-6.24588300	-2.05149000
C	-1.02753200	-5.58402600	-0.07457400
H	-1.12550100	-6.66520700	-0.25961600
H	-1.42043600	-5.35498600	0.92528600
H	-1.66900000	-5.06612300	-0.80734900
N	4.22074200	-2.15986900	-2.00328200
H	5.07171400	-1.95588200	-2.53593800
H	3.49188400	-2.44948600	-2.66172800
O	5.44838400	-4.42153700	-1.59456600
O	5.09170400	-2.76519700	0.29259100
S	4.59819600	-3.43404300	-0.92526200

II$_A$

Si	-1.66510900	-2.76909500	-0.61608300
H	-1.17342100	-1.46557300	-0.13055400
C	-1.00293200	-4.29237600	0.23649800
C	-1.97251400	-2.91851400	-2.46435300

H	-2.63689200	-3.79498200	-2.57892600
C	-0.74827900	-3.05763200	-3.38763400
H	-2.57479000	-2.04309500	-2.76774400
H	-1.68341100	-5.12405700	-0.02285400
H	-0.05195600	-4.51481300	-0.27822800
C	-0.78319900	-4.20752500	1.75614200
H	-1.71795500	-4.00676700	2.30053400
H	-0.07401600	-3.40780100	2.01663000
H	-0.37002100	-5.15487100	2.13630400
H	-0.15599700	-2.13359900	-3.41859700
H	-0.07630600	-3.86741000	-3.06751100
H	-1.07416200	-3.27699900	-4.41621000
C	2.89217100	0.26948400	1.47781700
C	2.03960700	-0.54773000	2.22803300
C	3.90412300	0.87744200	2.23140300
C	2.14560300	-0.75319900	3.60071900
C	4.04547600	0.70957700	3.61587000
C	3.15735100	-0.10801000	4.31144700
C	3.24790400	-1.24095500	-0.57062800
C	2.43996700	-2.31423200	-0.94355400
C	4.59383800	-1.59278000	-0.38393000
C	2.90355500	-3.61249000	-1.16694400
C	5.10999900	-2.87257600	-0.59525600
C	4.25268900	-3.90241600	-0.98817700
C	3.62498300	1.50018400	-0.92194400
C	4.45870600	1.34732200	-2.03546100
C	3.44589600	2.82884800	-0.51476100
C	5.10377900	2.41591800	-2.66955500
C	4.06672000	3.92492000	-1.11244400
C	4.90631600	3.71509200	-2.20715200

C	1.20238000	0.65986300	-0.70497100
C	0.87146600	0.45790100	-2.05276700
C	0.19303000	1.27301100	0.04726300
C	-0.36477600	0.77466900	-2.61681800
C	-1.05874100	1.62031000	-0.47681000
C	-1.33971500	1.36169800	-1.81408700
B	2.74824200	0.30215500	-0.18008900
F	3.85610200	5.16361400	-0.66061500
F	5.50770500	4.74402300	-2.80276000
F	2.04872000	-4.58589300	-1.52879000
F	1.10001000	-2.15638100	-1.11661800
F	4.71549300	-5.13423500	-1.18853200
F	6.40462100	-3.12537500	-0.41057100
F	5.47037100	-0.66506800	0.02064100
F	2.62908800	3.10156000	0.51967200
F	1.02600400	-1.20581400	1.61508000
F	1.27227100	-1.54698900	4.24253200
F	3.27413400	-0.27839900	5.62765700
F	5.02693000	1.32613700	4.27393200
F	4.82230900	1.66268100	1.65911100
F	0.36188200	1.56059600	1.34629300
F	-2.00460200	2.16579100	0.30280100
F	-2.55159200	1.65734800	-2.32939200
F	-0.63427100	0.49594500	-3.89544500
F	1.75923200	-0.10324900	-2.88470900
F	4.68660800	0.14074800	-2.57624200
F	5.89433800	2.20037600	-3.72268700
C	-4.18392300	-1.95232900	0.56863100
O	-3.41927300	-2.72394200	-0.05926400
C	-3.69294400	-0.65670400	1.14711800

H	-4.53234800	0.03652700	1.29618200
H	-2.98449600	-0.15544100	0.47711000
C	-3.01423800	-0.89475900	2.50014900
O	-3.03566400	-1.95685700	3.07870600
C	-5.58127500	-2.37484300	0.83156200
H	-5.63645900	-2.39027900	1.93983800
H	-6.23375700	-1.53434100	0.52536800
C	-6.05125600	-3.69758400	0.22352000
H	-5.32259200	-4.49114000	0.45693200
H	-6.98437200	-3.97716400	0.73808600
C	-6.31023600	-3.63298700	-1.28469400
H	-5.38322800	-3.44295000	-1.84947800
H	-6.72364300	-4.58518300	-1.65066400
H	-7.03087200	-2.83538000	-1.52543800
O	-2.43611500	0.21329000	2.92001400
C	-1.68671700	0.16962700	4.16706600
H	-0.83122900	-0.50463000	4.02146300
H	-2.33374200	-0.26531800	4.94417000
C	-1.25197300	1.58263600	4.48460900
H	-2.11950200	2.23788700	4.65865600
H	-0.63141400	1.58113200	5.39368500
H	-0.65868500	1.99530600	3.65610100
C	-5.03557700	4.21522200	1.06928800
C	-5.84503000	3.18440300	1.57389400
C	-6.27032600	2.13318800	0.75874300
C	-5.87916300	2.11628700	-0.58235900
C	-5.10853600	3.15044200	-1.12571000
C	-4.68452300	4.18259300	-0.29260900
H	-6.15340800	3.20666000	2.62237400
H	-6.91679600	1.34375200	1.14650400

H	-4.84536500	3.15040500	-2.18471000
H	-4.07028400	4.98504200	-0.70919500
C	-4.53921300	5.32574400	1.95793800
H	-4.65268300	6.30821200	1.47372700
H	-5.07381400	5.35013400	2.91832800
H	-3.46502600	5.19446300	2.17370300
N	-4.98154900	-0.14784700	-1.79277700
H	-5.21508300	-0.90797700	-2.44121100
H	-4.25773200	0.42964000	-2.23579200
O	-6.80909200	1.32162000	-2.93577500
O	-7.34277500	-0.09038800	-0.88712900
S	-6.42256500	0.78031900	-1.63429600

TS_B

Si	3.89757151	3.20482753	-0.47753098
C	3.25241451	2.92801753	-2.22775598
C	2.53732351	3.68302153	0.72249402
H	1.99874451	4.50982153	0.22051302
C	2.95819251	4.10973053	2.13863002
H	1.80112451	2.86252653	0.76418202
H	4.07311251	2.50065353	-2.83389798
H	2.47424251	2.15004053	-2.16984698
C	2.67232051	4.16282153	-2.94146898
H	3.42819051	4.94884253	-3.09411398
H	1.83851951	4.60776053	-2.37561698
H	2.27810951	3.89266153	-3.93363198
H	3.44826251	3.28486353	2.67485102
H	3.64875751	4.96818053	2.11694502
H	2.07949051	4.40684753	2.73003302

C	-3.77272249	0.54076053	-1.12228498
C	-3.54590249	1.52944353	-2.08864598
C	-5.00886349	-0.10364347	-1.23665498
C	-4.46684249	1.90052553	-3.06675998
C	-5.96721349	0.23389953	-2.20019798
C	-5.69526849	1.24059353	-3.12396898
C	-1.41993949	-0.49561847	-1.13486098
C	-0.18600849	0.02925853	-1.52153098
C	-1.78983349	-1.65512347	-1.83717698
C	0.61498151	-0.54249347	-2.51450498
C	-1.02913049	-2.24564447	-2.84609798
C	0.18499651	-1.66608547	-3.20736698
C	-2.94760249	-0.90853747	1.13203702
C	-2.21929049	-2.03877147	1.51269202
C	-4.01917749	-0.60654847	1.98463402
C	-2.53562049	-2.82910447	2.62059902
C	-4.38118149	-1.37260647	3.09412502
C	-3.62448249	-2.49904947	3.42217902
C	-2.00027249	1.46923453	0.79679702
C	-0.87175049	1.33922653	1.61293002
C	-2.66758549	2.69306853	0.94845202
C	-0.41069349	2.32053453	2.48610202
C	-2.23196349	3.71687753	1.80165602
C	-1.09320949	3.53149253	2.58090802
B	-2.53977949	0.15704853	-0.07787698
F	-5.42236849	-1.03003747	3.85283002
F	-3.93390149	-3.23990347	4.48534502
F	1.83858151	-0.02466747	-2.79555598
F	0.33828751	1.13336653	-0.94356498
F	0.94988951	-2.21974447	-4.15504698

F	-1.42427949	-3.37262347	-3.44206298
F	-2.93637549	-2.27483547	-1.53958598
F	-4.76023949	0.48628053	1.75451902
F	-2.37376249	2.19561153	-2.09299198
F	-4.18412149	2.86433653	-3.94700798
F	-6.59429949	1.56921153	-4.05082698
F	-7.13228849	-0.41454047	-2.25181398
F	-5.34878049	-1.11164447	-0.42104498
F	-3.79311749	2.96505953	0.28086402
F	-2.90746249	4.86300953	1.88075402
F	-0.64979249	4.49500453	3.39034602
F	0.69147651	2.11287453	3.22547902
F	-0.14396749	0.19931653	1.58304302
F	-1.12266449	-2.43686047	0.83051902
F	-1.76748449	-3.88577647	2.93551602
C	5.42123951	4.30779453	-0.35959598
H	6.17328851	3.92286453	-1.07169998
H	5.85099851	4.13797253	0.64411602
C	5.19953051	5.81370653	-0.58250098
H	6.13551951	6.37716153	-0.44078898
H	4.84045751	6.03405453	-1.59976598
H	4.46156751	6.22973353	0.12192502
C	4.54615551	0.40580453	0.31532302
O	4.62257251	1.65452953	-0.03988698
C	3.21430951	-0.14559047	0.81043202
H	3.12318351	-1.21103147	0.57106002
H	2.37242651	0.37013353	0.32131002
C	3.02432651	-0.00383847	2.32046602
O	3.51440851	0.86618253	3.00014302
C	5.79507151	-0.11601947	0.99521602

H	5.69343751	0.25208553	2.03168802
H	5.75633451	-1.21313747	1.04489402
C	7.13314251	0.35965053	0.42430302
H	7.23169251	0.02724253	-0.62178598
H	7.14999051	1.46080953	0.41678502
C	8.32005351	-0.17931047	1.22383402
H	8.35003951	-1.28034447	1.19586002
H	9.27246551	0.18974853	0.81366202
H	8.26824651	0.13153353	2.28034902
O	2.24805651	-0.98444247	2.76552902
C	1.80238151	-0.93809647	4.14313202
H	1.10587951	-0.09260347	4.23637902
H	2.67145251	-0.73158747	4.78642802
C	1.14600951	-2.26361547	4.46148602
H	1.87076151	-3.09048947	4.39713702
H	0.74467551	-2.24140847	5.48638202
H	0.31678451	-2.47742747	3.77160102
C	1.80273051	-4.72384147	0.00181902
C	2.99560051	-4.47854947	0.70938002
C	4.02562051	-3.71986147	0.15748302
C	3.85065751	-3.18933247	-1.12786298
C	2.69939651	-3.45180247	-1.87958298
C	1.68574851	-4.21583247	-1.30543698
H	3.11842451	-4.89226847	1.71315502
H	4.95379251	-3.55126947	0.70571302
H	2.60264851	-3.06905747	-2.89545298
H	0.78139951	-4.41548947	-1.88521798
C	0.66832751	-5.48288147	0.63207102
H	1.01625751	-6.13758547	1.44405002
H	-0.06213249	-4.77562247	1.06079802

H	0.13256051	-6.09420247	-0.10935898
N	4.60184351	-0.48201347	-1.41492898
H	5.27253451	0.10303153	-1.92986198
H	3.68489951	-0.32389347	-1.86183198
O	5.02706351	-2.20495947	-3.30090898
O	6.38179951	-2.36658747	-1.14371398
S	5.11754151	-2.18777647	-1.84695198

TS_A

Si	2.16003123	2.62665614	-0.54886724
H	1.53059523	1.37267214	-0.07387024
C	1.61888223	4.15059514	0.39278076
C	2.20039623	2.86137214	-2.41827024
H	2.99362823	3.61144314	-2.59798324
C	0.89517623	3.30127014	-3.10470924
H	2.56025923	1.92386314	-2.88006924
H	2.36627123	4.94318114	0.20587276
H	0.69377923	4.48101814	-0.11105324
C	1.35095523	3.98251014	1.89694776
H	2.25343623	3.68362114	2.45058976
H	0.57645423	3.21939014	2.07542276
H	0.98744223	4.92562314	2.33490776
H	0.13162623	2.51353914	-3.06639324
H	0.46243223	4.19895214	-2.63761424
H	1.07770123	3.53176214	-4.16601124
C	-2.91903977	-0.44197086	1.48161376
C	-2.08590277	0.22374214	2.38889076
C	-3.98309277	-1.12664886	2.08190776
C	-2.25170877	0.20361414	3.77165876
C	-4.18820977	-1.17820786	3.46713676

C	-3.31469677	-0.51127786	4.32258276
C	-3.09758577	1.36976814	-0.34366924
C	-2.22433177	2.44086814	-0.53922824
C	-4.43331877	1.75928414	-0.16216824
C	-2.62262777	3.77813414	-0.61153324
C	-4.88310877	3.07928414	-0.22170524
C	-3.96500477	4.10672914	-0.44733624
C	-3.56920177	-1.27698186	-1.10256724
C	-4.35815777	-0.93299986	-2.20585424
C	-3.43749877	-2.65684686	-0.89730224
C	-5.00647777	-1.87418086	-3.01486024
C	-4.06281077	-3.63125786	-1.67398324
C	-4.85763977	-3.23352586	-2.74976624
C	-1.13135577	-0.57610386	-0.65437424
C	-0.73869877	-0.20537486	-1.94879124
C	-0.16222677	-1.29036386	0.06047576
C	0.52955323	-0.43416286	-2.48235724
C	1.12118023	-1.54875486	-0.43510824
C	1.47300123	-1.08769686	-1.69703124
B	-2.68923677	-0.23609686	-0.15374324
F	-3.89842477	-4.93019486	-1.40973124
F	-5.46247577	-4.14252086	-3.51403624
F	-1.71577777	4.74679214	-0.82105924
F	-0.88908077	2.24285614	-0.67436324
F	-4.36569277	5.37539914	-0.50556124
F	-6.17283077	3.37005414	-0.05363324
F	-5.36661877	0.82936414	0.08127876
F	-2.66605377	-3.10699986	0.10993976
F	-1.02782877	0.93576714	1.93358476
F	-1.39329677	0.84898414	4.57794276

F	-3.48829377	-0.55203986	5.64325476
F	-5.21711077	-1.85846286	3.97330676
F	-4.89162577	-1.78161686	1.35065976
F	-0.40123377	-1.76719486	1.28896376
F	2.04116723	-2.18082786	0.31310576
F	2.74926323	-1.21980786	-2.14240124
F	0.86968123	0.01432014	-3.69486724
F	-1.59009677	0.45455814	-2.74505024
F	-4.53479177	0.34669714	-2.56680124
F	-5.75264377	-1.47867786	-4.04834924
C	4.71025723	1.49893614	0.29482276
O	3.87986623	2.38579914	-0.19928124
C	4.13838823	0.36717914	1.14088876
H	4.95205023	-0.31799586	1.42221276
H	3.40215823	-0.22948786	0.59159776
C	3.48777423	0.84380314	2.43991776
O	3.82370923	1.82881914	3.05651476
C	6.01915823	2.09340614	0.78319176
H	5.80717523	2.30891514	1.84509876
H	6.80018723	1.31968414	0.76425976
C	6.49786323	3.38789414	0.11185276
H	5.72823423	4.16401314	0.24723676
H	7.37501023	3.72835814	0.68636476
C	6.88552223	3.30042014	-1.36764024
H	6.00397223	3.16822714	-2.02036124
H	7.36212423	4.23632114	-1.69751424
H	7.59719023	2.48173214	-1.55909124
O	2.53522723	-0.00498586	2.79388976
C	1.78412823	0.23609014	4.01495276
H	0.98328023	0.94800214	3.77105676

H	2.45409823	0.70072714	4.75283676
C	1.21603523	-1.08791386	4.47606976
H	2.01429123	-1.78809086	4.76814676
H	0.55887223	-0.92115486	5.34208476
H	0.62097723	-1.54664986	3.67306476
C	5.28816023	-4.34613486	0.93996676
C	6.27134423	-3.45405686	1.40384876
C	6.64238623	-2.33484686	0.65965076
C	6.01240423	-2.11062986	-0.57062224
C	5.05463923	-2.99694786	-1.08082024
C	4.69601723	-4.10070286	-0.31319024
H	6.75957523	-3.64168686	2.36322476
H	7.41843223	-1.65481286	1.01470076
H	4.59797823	-2.82417186	-2.05537924
H	3.93905623	-4.78965986	-0.69559524
C	4.86491023	-5.53588686	1.75879276
H	4.87601123	-6.45747486	1.15569376
H	5.51565623	-5.68487086	2.63170376
H	3.83214723	-5.40332386	2.12325076
N	5.17219123	0.52361114	-1.24522924
H	5.41469323	1.27602614	-1.90480524
H	4.30156923	0.09473514	-1.59596624
O	6.36189123	-0.99722086	-2.96060424
O	7.69555723	-0.09549486	-0.97541524
S	6.50085123	-0.71272986	-1.53923124

Int-1$_B$

Si	3.75449800	3.17585000	-0.40606000
C	3.05634100	3.08035100	-2.16221800
C	2.41273900	3.61864500	0.83558900

H	1.87601500	4.47431900	0.38288800
C	2.85283200	3.97163400	2.26583600
H	1.66763300	2.80502200	0.85059400
H	3.84900600	2.67754700	-2.82130300
H	2.25637300	2.32180100	-2.15005100
C	2.49916900	4.38214000	-2.76412100
H	3.27702500	5.15347400	-2.87596600
H	1.69669000	4.80942000	-2.14188100
H	2.07006300	4.20552500	-3.76329000
H	3.34451800	3.11821300	2.75326900
H	3.54928400	4.82564900	2.27854700
H	1.98432600	4.24493300	2.88409400
C	-3.93234300	0.59313700	-1.10305500
C	-3.71070600	1.62944400	-2.01953200
C	-5.16804400	-0.04667000	-1.24468200
C	-4.63674500	2.04816200	-2.97343000
C	-6.13125300	0.33774500	-2.18544500
C	-5.86470400	1.39030500	-3.05825200
C	-1.57837000	-0.43908000	-1.17569200
C	-0.34609300	0.10874000	-1.53630700
C	-1.94495000	-1.56464600	-1.93234100
C	0.46069300	-0.41805100	-2.54941800
C	-1.18047800	-2.10589000	-2.96591400
C	0.03384400	-1.50839900	-3.29540100
C	-3.09984000	-0.96602500	1.07261000
C	-2.37270900	-2.11442700	1.39581500
C	-4.16735200	-0.70328600	1.94347200
C	-2.68476900	-2.95431300	2.46779000
C	-4.52532500	-1.51967700	3.01783600
C	-3.76922500	-2.66146800	3.28923800

C	-2.14969300	1.42341400	0.85437800
C	-1.01828200	1.24967700	1.65858100
C	-2.81240400	2.64015300	1.06864700
C	-0.54982100	2.18486400	2.57727400
C	-2.36975600	3.61891500	1.96948200
C	-1.22829200	3.39185300	2.73381500
B	-2.69533300	0.15920200	-0.08370000
F	-5.56255400	-1.21180800	3.79623600
F	-4.07455000	-3.45131600	4.31764600
F	1.68829300	0.10860700	-2.79886000
F	0.16968300	1.18796500	-0.91109300
F	0.80679400	-2.02033300	-4.26154800
F	-1.56995600	-3.20747900	-3.61235700
F	-3.08959700	-2.20126600	-1.66564800
F	-4.90789300	0.39957600	1.76827900
F	-2.53900800	2.29508900	-1.99638600
F	-4.35918200	3.05497400	-3.80558700
F	-6.76832000	1.76385100	-3.96334700
F	-7.29565800	-0.30926500	-2.26470200
F	-5.50280900	-1.09569100	-0.47985500
F	-3.94007000	2.94746100	0.41950300
F	-3.04151800	4.76163900	2.10810600
F	-0.77977300	4.31249100	3.58815900
F	0.55323600	1.93748300	3.30084600
F	-0.29425800	0.10997200	1.56760200
F	-1.28082200	-2.48451300	0.68881300
F	-1.91734600	-4.02727600	2.72904600
C	5.28679200	4.26809300	-0.24378500
H	6.02409700	3.92567200	-0.99252100
H	5.73181000	4.02569500	0.73827000

C	5.08051800	5.78742600	-0.35970000
H	6.02332500	6.33216300	-0.18984800
H	4.71277600	6.08135100	-1.35502300
H	4.35364700	6.16136700	0.37909900
C	4.35658800	0.28511700	0.11181100
O	4.45536600	1.61849900	-0.11160000
C	3.01441900	-0.20946800	0.68814400
H	2.83308300	-1.25073500	0.39561600
H	2.18766000	0.38151000	0.26094300
C	2.87554000	-0.14276400	2.20273500
O	3.36139500	0.70259500	2.91434100
C	5.58746400	-0.20360900	0.88915000
H	5.46054400	0.20719400	1.90120000
H	5.55231400	-1.29999100	0.98411700
C	6.94521700	0.24598600	0.33907600
H	7.08897900	-0.12463000	-0.69000400
H	6.95888600	1.34571900	0.28908500
C	8.10620300	-0.25319900	1.20080000
H	8.14165700	-1.35455300	1.22509600
H	9.07201500	0.10195300	0.80974200
H	8.01475200	0.10305100	2.24002700
O	2.12958500	-1.16172600	2.62867900
C	1.71963400	-1.17196000	4.01600600
H	1.03527100	-0.32463900	4.16732200
H	2.60580400	-1.00400600	4.64742900
C	1.05559100	-2.50339500	4.29285500
H	1.76843600	-3.33466800	4.17265500
H	0.68109500	-2.52386700	5.32789200
H	0.20619000	-2.67671800	3.61602300
C	1.53766800	-4.75703400	-0.15718200

C	2.73550600	-4.55837800	0.55945500
C	3.76921600	-3.78120900	0.04713600
C	3.59080000	-3.18546100	-1.21148400
C	2.43084900	-3.38972100	-1.97152000
C	1.41658000	-4.17689500	-1.43471800
H	2.85698600	-5.02374200	1.54024200
H	4.69730000	-3.64430400	0.60378400
H	2.33205300	-2.94965200	-2.96343400
H	0.50785700	-4.33770400	-2.01934500
C	0.40271000	-5.54194600	0.43455800
H	0.74880800	-6.24241500	1.20793900
H	-0.31834900	-4.85151000	0.90477400
H	-0.14236800	-6.10497400	-0.33753700
N	4.39993200	-0.35231500	-1.35768700
H	5.11326800	0.18322300	-1.87169200
H	3.50408000	-0.15704700	-1.83604800
O	4.72229700	-2.02204700	-3.32495800
O	6.13591500	-2.41885600	-1.23118700
S	4.86175200	-2.18271900	-1.88742500

Int-1$_A$

Si	2.09140000	2.59467200	-0.60807000
H	1.41436300	1.36047900	-0.13964900
C	1.54762400	4.12678400	0.32288500
C	2.08120300	2.83233400	-2.48082600
H	2.87061300	3.58105800	-2.68283500
C	0.75952900	3.27179600	-3.13402700
H	2.42864800	1.89452500	-2.95248300
H	2.27872800	4.92745000	0.10742700
H	0.60498200	4.43782100	-0.16065900

C	1.32097100	3.97821400	1.83524300
H	2.24046500	3.69320500	2.36845100
H	0.55675800	3.21202600	2.04351900
H	0.96191900	4.92339500	2.27293300
H	-0.00633600	2.48793800	-3.06641100
H	0.34350100	4.17456400	-2.66125900
H	0.90917400	3.49389200	-4.20251300
C	-3.10792300	-0.45981000	1.46735900
C	-2.28693400	0.21514600	2.37893700
C	-4.17558800	-1.14464300	2.06077900
C	-2.46748200	0.20396800	3.76004000
C	-4.39527100	-1.18757600	3.44403700
C	-3.53396700	-0.51093800	4.30425300
C	-3.26564700	1.34564200	-0.36635600
C	-2.38635400	2.41362900	-0.55584300
C	-4.60198400	1.73850200	-0.19894100
C	-2.78111400	3.75183200	-0.63430000
C	-5.04772900	3.05945200	-0.26565900
C	-4.12446900	4.08369600	-0.48440100
C	-3.73261300	-1.30576600	-1.11848700
C	-4.51549100	-0.96807200	-2.22803500
C	-3.59758300	-2.68450000	-0.90793700
C	-5.15536100	-1.91428600	-3.03803400
C	-4.21431400	-3.66364400	-1.68547900
C	-5.00354900	-3.27222000	-2.76762000
C	-1.29946100	-0.59972000	-0.65183500
C	-0.89959300	-0.23657000	-1.94625600
C	-0.33222300	-1.30270200	0.07635500
C	0.37415500	-0.46060800	-2.46832900
C	0.95619000	-1.55629700	-0.40761200

C	1.31487300	-1.09934800	-1.66860700
B	-2.86205900	-0.25971400	-0.16627400
F	-4.04684700	-4.96144000	-1.41607500
F	-5.60019300	-4.18598400	-3.53301600
F	-1.87059600	4.71769300	-0.83654100
F	-1.05221200	2.21183600	-0.67727000
F	-4.52162900	5.35344100	-0.54920900
F	-6.33860000	3.35418300	-0.11093800
F	-5.53991800	0.81097400	0.03816400
F	-2.83118100	-3.12902400	0.10585100
F	-1.22793800	0.92810000	1.92950300
F	-1.62134100	0.86006500	4.57033600
F	-3.72301700	-0.54239600	5.62317900
F	-5.42719600	-1.86841500	3.94392600
F	-5.07404100	-1.80813700	1.32427600
F	-0.57746200	-1.77237000	1.30631000
F	1.87483400	-2.17959800	0.35143000
F	2.60096000	-1.21577100	-2.09874000
F	0.72162800	-0.01954100	-3.68121900
F	-1.74869200	0.41185200	-2.75367000
F	-4.69370100	0.30941600	-2.59494600
F	-5.89606600	-1.52492500	-4.07789500
C	4.64209400	1.37432200	0.16362300
O	3.78142000	2.32635000	-0.27154900
C	4.05271100	0.32247800	1.11652700
H	4.86173600	-0.34615300	1.44959000
H	3.31218700	-0.30923900	0.61244900
C	3.41079600	0.88274000	2.38258600
O	3.84027900	1.81583100	3.02081400
C	5.93497900	2.01467600	0.68783400

H	5.69990400	2.28235800	1.72890100
H	6.72542000	1.25004700	0.72368100
C	6.42804400	3.27809300	-0.03376600
H	5.66031200	4.06101100	0.06326700
H	7.30196000	3.63750700	0.53446100
C	6.82949500	3.13781700	-1.50629200
H	5.95333100	3.00707200	-2.16813500
H	7.32678600	4.05372900	-1.86103200
H	7.52880900	2.30191600	-1.66937700
O	2.34626800	0.15426800	2.69402800
C	1.60121100	0.43776300	3.90827200
H	0.76675300	1.09489500	3.62726000
H	2.25826600	0.97592300	4.60596400
C	1.09359400	-0.87718700	4.46052600
H	1.92374800	-1.52321500	4.78717600
H	0.43691700	-0.68385400	5.32127200
H	0.51122700	-1.41067800	3.69499700
C	5.00472700	-4.44019800	0.96185900
C	6.01212400	-3.57881500	1.43534000
C	6.41329500	-2.46346700	0.70408400
C	5.78852800	-2.21394100	-0.52614000
C	4.80467000	-3.06693200	-1.04607800
C	4.41957700	-4.16842200	-0.28952800
H	6.49380100	-3.78810400	2.39337400
H	7.20526400	-1.80727800	1.06835800
H	4.34926800	-2.86948400	-2.01615800
H	3.64456900	-4.83324700	-0.67799200
C	4.54607200	-5.62109100	1.77250700
H	4.48021600	-6.52854400	1.15226700
H	5.21743800	-5.82387000	2.61843000

H	3.53655100	-5.43528700	2.17750400
N	4.98290300	0.53220300	-1.17922800
H	5.26582300	1.23476700	-1.87879000
H	4.10753900	0.10917600	-1.52978100
O	6.09287600	-1.04529600	-2.90042100
O	7.53848800	-0.25968600	-0.93932800
S	6.31581900	-0.83138500	-1.48004100

Int-2$_B$

Si	-3.92318700	1.16171900	1.96520700
C	-3.76542700	-0.08405100	3.38109500
C	-2.48761400	2.37707700	1.90608800
H	-1.78145400	2.07615800	2.69580400
C	-2.84387300	3.86410400	2.07590600
H	-1.93663600	2.23749400	0.96454700
H	-4.66108900	-0.73094300	3.38139300
H	-2.91620900	-0.75382000	3.15614900
C	-3.54771800	0.52431400	4.77995000
H	-4.36766600	1.19565100	5.07855100
H	-2.60767300	1.09268300	4.83346000
H	-3.48191200	-0.27108000	5.53838200
H	-3.53264300	4.20706300	1.29011100
H	-3.30743000	4.07215900	3.05375700
H	-1.93363100	4.48074100	2.01347800
C	3.99020100	-0.13286100	0.56857300
C	4.62145900	0.57948400	1.59695200
C	4.66656900	-1.29188800	0.17268400
C	5.84399200	0.22114100	2.16473200
C	5.89324700	-1.69392000	0.71286400
C	6.48792700	-0.93322800	1.71707800

C	1.54984900	-0.03404700	1.38506600
C	1.04117800	0.87164000	2.32364200
C	1.33962500	-1.37782600	1.72020400
C	0.35461700	0.48258400	3.47996000
C	0.64454300	-1.80812700	2.85017900
C	0.13044900	-0.86544000	3.73912600
C	1.99737800	-0.39630900	-1.35623900
C	0.74745600	-0.95089800	-1.62621600
C	2.83598400	-0.33313700	-2.48151300
C	0.35402300	-1.42280000	-2.88060100
C	2.49624100	-0.80917300	-3.74937200
C	1.23049800	-1.36016600	-3.95787100
C	2.37340900	1.94662800	-0.38202600
C	1.11388800	2.50561900	-0.61933600
C	3.43828300	2.80689400	-0.67803000
C	0.89157500	3.80742000	-1.05527400
C	3.26707600	4.12316100	-1.12954100
C	1.98394000	4.63018400	-1.32528900
B	2.48585500	0.34351500	0.05788500
F	3.35514400	-0.72572100	-4.76348100
F	0.86714100	-1.80659900	-5.15858500
F	-0.13928400	1.40839500	4.31469100
F	1.16840800	2.19579700	2.16677800
F	-0.57384900	-1.24810500	4.80618800
F	0.43605800	-3.11480000	3.07757300
F	1.81519500	-2.34622800	0.91702600
F	4.04796800	0.22233600	-2.37304400
F	4.03748400	1.68408200	2.09289100
F	6.39228600	0.95671700	3.13338400
F	7.65329400	-1.30403400	2.24553000

F	6.48963900	-2.80871700	0.28291300
F	4.16065000	-2.11049800	-0.76675500
F	4.70829700	2.41154800	-0.54814200
F	4.32482100	4.89101500	-1.38764700
F	1.79931800	5.87540400	-1.76227800
F	-0.35932400	4.26697600	-1.23197800
F	-0.00339200	1.76322200	-0.40626400
F	-0.21694100	-1.04429400	-0.66866500
F	-0.89283000	-1.90961200	-3.05510500
C	-5.66919000	1.85525600	1.80682100
H	-6.32391400	1.02388900	1.49394200
H	-5.67140600	2.58077900	0.97998100
C	-6.24161000	2.49630900	3.08531600
H	-7.24826600	2.90246400	2.89696600
H	-6.33475200	1.77033600	3.90798600
H	-5.62087100	3.33105000	3.44695800
C	-3.83665100	-0.27706500	-0.93171700
O	-3.70599300	-0.08376200	0.60437400
C	-2.90575800	0.72888700	-1.62870700
H	-2.77890200	0.39837800	-2.66886700
H	-1.90871700	0.67876800	-1.16247000
C	-3.32482600	2.18450100	-1.63030100
O	-4.00167200	2.70779800	-0.77003300
C	-5.30912700	-0.09233200	-1.31870400
H	-5.55235200	0.95835000	-1.10979900
H	-5.33792500	-0.21039100	-2.41555500
C	-6.39021000	-0.97928100	-0.68718000
H	-6.27288900	-2.02067700	-1.01222700
H	-6.28196200	-0.98602700	0.40962300
C	-7.79216100	-0.48948700	-1.05848200

H	-7.93982400	-0.49057500	-2.15108200
H	-8.56461100	-1.13981300	-0.62041400
H	-7.97483300	0.53769200	-0.70091200
O	-2.82882200	2.81942300	-2.68196200
C	-3.06846700	4.24660900	-2.78549800
H	-2.77980700	4.71587600	-1.83472900
H	-4.15082100	4.39894600	-2.92499000
C	-2.25736800	4.77412800	-3.94758400
H	-2.52087300	4.26104300	-4.88505400
H	-2.45528100	5.84958000	-4.07506800
H	-1.18216000	4.64429500	-3.75877100
C	0.14413100	-5.41279000	-0.84455600
C	-0.81597700	-5.40591700	-1.87144200
C	-2.00629200	-4.69227700	-1.74327900
C	-2.22733700	-3.96760100	-0.56614700
C	-1.30304600	-3.96757400	0.48428000
C	-0.12750600	-4.69803800	0.33611000
H	-0.62738700	-5.96647800	-2.79029200
H	-2.75174000	-4.69476200	-2.53988300
H	-1.49866600	-3.41644100	1.40405100
H	0.60123100	-4.68957000	1.14735700
C	1.45050300	-6.14594900	-0.99522600
H	1.61192600	-6.84471700	-0.15862100
H	1.49669500	-6.71467700	-1.93459800
H	2.29164700	-5.43246400	-0.98362800
N	-3.27474900	-1.56746300	-1.23474100
H	-3.79135500	-1.02769700	0.97510100
H	-2.26217900	-1.55808900	-1.37951100
O	-3.95499200	-2.60331800	0.97959800
O	-4.78609600	-3.70133100	-1.15294500

S	-3.71516000	-3.01997300	-0.43792200

Int-2$_A$

Si	1.99060200	-0.88100500	1.90842600
H	1.17352200	-0.47715100	0.74308900
C	1.36646900	-2.36623400	2.86385700
C	2.56010600	0.52346600	3.00457200
H	3.38314400	0.14148200	3.63374100
C	1.43078400	1.11932400	3.87205900
H	2.99943200	1.32463800	2.39228500
H	2.19420100	-2.70300000	3.51521200
H	0.61536500	-1.93037700	3.54545900
C	0.73339900	-3.54766600	2.11166800
H	1.45767100	-4.08910300	1.48515300
H	-0.08360400	-3.21537200	1.45408100
H	0.31256100	-4.27244400	2.82614200
H	0.67034300	1.61769900	3.25396100
H	0.91466600	0.36397800	4.48375500
H	1.84540400	1.87609100	4.55566900
C	-3.46116400	-0.96781600	-0.94891700
C	-2.60424200	-2.07330100	-0.91267300
C	-4.65519700	-1.19814300	-1.64361600
C	-2.85586700	-3.29075600	-1.53905600
C	-4.95817100	-2.40855100	-2.28223500
C	-4.04992500	-3.46406200	-2.23788600
C	-3.26772500	-0.09754100	1.47353500
C	-2.26905000	-0.40623600	2.39772300
C	-4.56296300	-0.38428600	1.93230600
C	-2.50958100	-0.90078100	3.68227600
C	-4.85762300	-0.88034600	3.20326200

C	-3.81576400	-1.14837000	4.09296600
C	-3.97891900	1.74255500	-0.49371000
C	-4.58393100	2.62338800	0.41006500
C	-4.07443400	2.13858200	-1.83431500
C	-5.26956600	3.77997000	0.01994000
C	-4.74690200	3.27911900	-2.27175300
C	-5.35157100	4.11274500	-1.33024100
C	-1.52313000	0.95218000	-0.44863200
C	-0.91915300	1.91249300	0.37617200
C	-0.76151100	0.60350700	-1.57071700
C	0.36492800	2.42697400	0.18167800
C	0.51892500	1.10702400	-1.81766100
C	1.09580100	2.00164300	-0.92443500
B	-3.06678700	0.41181500	-0.10287000
F	-4.80541600	3.58586900	-3.56994000
F	-5.99574400	5.21296300	-1.71811700
F	-1.48539200	-1.16077100	4.51332400
F	-0.95682100	-0.24254800	2.09473700
F	-4.06632900	-1.62817000	5.30962000
F	-6.11730000	-1.11355300	3.57032600
F	-5.61252400	-0.17174600	1.12766700
F	-3.49193400	1.39161700	-2.79029500
F	-1.41814600	-1.98824400	-0.26032100
F	-1.94873700	-4.28189700	-1.49975200
F	-4.31237500	-4.61761000	-2.85215300
F	-6.11001900	-2.55763000	-2.93645900
F	-5.59570900	-0.25335800	-1.74245400
F	-1.20623400	-0.26872300	-2.48902300
F	1.23230500	0.68269500	-2.87570000
F	2.37491400	2.39617200	-1.10230800

F	0.91150400	3.26831200	1.06171800
F	-1.56201900	2.36056600	1.46250800
F	-4.53445300	2.40870100	1.73361700
F	-5.83294000	4.57342100	0.93333500
C	4.32364900	-0.98245000	-0.10384000
O	3.44399600	-1.60113300	1.00725500
C	3.55504500	-1.15995200	-1.43894600
H	4.27911100	-1.17354100	-2.26810600
H	2.91047700	-0.29276000	-1.64160700
C	2.65954700	-2.37898400	-1.56054000
O	2.48875500	-3.18573600	-0.64840100
C	5.66731200	-1.73165900	-0.04753600
H	6.30122800	-1.21363800	-0.78528400
H	6.10613800	-1.53835600	0.94049500
C	5.72236300	-3.24402300	-0.33404000
H	5.12250100	-3.50558100	-1.22161400
H	6.76475700	-3.45122200	-0.62807800
C	5.37429300	-4.17208800	0.83385800
H	4.31541300	-4.12727100	1.12452300
H	5.58857300	-5.21825800	0.56618900
H	5.97503200	-3.92583300	1.72429600
O	2.05885600	-2.44443900	-2.71414900
C	0.99134000	-3.43297100	-2.90297100
H	0.34640400	-3.40468000	-2.01465100
H	1.46415800	-4.42533100	-2.95884100
C	0.24232700	-3.05936900	-4.15990200
H	0.88999900	-3.10584900	-5.04842200
H	-0.59292500	-3.76264600	-4.29756300
H	-0.17115000	-2.04475400	-4.06787800
C	9.27202800	2.16250400	-1.41359900

C	9.34035700	1.24911700	-0.35056400
C	8.21554100	0.95074500	0.42331500
C	7.00381000	1.57325400	0.12086900
C	6.90547900	2.50478200	-0.92104100
C	8.03649700	2.78329800	-1.68302000
H	10.29231900	0.76618700	-0.11545200
H	8.27683200	0.25381700	1.26060700
H	5.95982700	3.01203600	-1.12295300
H	7.96461500	3.50778000	-2.49878400
C	10.48741800	2.49539300	-2.24034000
H	10.78835300	3.54572000	-2.08914100
H	11.34481300	1.85905500	-1.97913300
H	10.28334200	2.37247100	-3.31625900
N	4.47087600	0.40691600	0.11944900
H	3.08936500	-2.45452000	0.55386300
H	3.76359000	1.04696400	-0.24498700
O	4.88014100	2.52014000	1.35281400
O	5.95482500	0.36454300	2.23008700
S	5.56659100	1.24999300	1.13244100

Int-3$_B$

C	-2.25550700	-1.90863800	-0.56280000
C	-0.93064400	-2.34067200	-0.67570900
C	-3.19618400	-2.92238900	-0.79693200
C	-0.54968000	-3.62752700	-1.04166300
C	-2.85815200	-4.23311800	-1.16248000
C	-1.51971200	-4.59637400	-1.28556700
C	-2.05854500	-0.40199200	1.53715400
C	-0.92102100	0.22742900	2.04419300
C	-2.69973300	-1.24144100	2.46108800

C	-0.47158500	0.08985600	3.35954900
C	-2.28543000	-1.42193800	3.78207500
C	-1.15033000	-0.74888500	4.23646500
C	-4.20362600	0.04130700	-0.19012000
C	-5.02650500	0.58702600	0.80020100
C	-4.79872900	-0.03701100	-1.45636200
C	-6.34844500	0.98685900	0.57099200
C	-6.10982700	0.34691500	-1.73403100
C	-6.89494700	0.86886500	-0.70479600
C	-1.87093500	0.84377700	-0.93645800
C	-1.99561500	2.17155000	-0.49819400
C	-1.22829300	0.70916500	-2.17054300
C	-1.50680700	3.27565900	-1.19493600
C	-0.70586700	1.78617100	-2.89776300
C	-0.85054600	3.07971700	-2.40936000
B	-2.59991700	-0.35994600	-0.03844000
F	-6.61405400	0.23047300	-2.96427600
F	-8.14990700	1.24944900	-0.94070100
F	0.63641300	0.73234700	3.76759100
F	-0.13623200	1.01575600	1.26702500
F	-0.70786600	-0.92072200	5.48497700
F	-2.94717300	-2.23513100	4.60377500
F	-3.78302100	-1.93268500	2.08526900
F	-4.08860600	-0.52176300	-2.49185400
F	0.09425200	-1.47902400	-0.43410000
F	0.75827000	-3.95604800	-1.15390200
F	-1.16961300	-5.83408500	-1.62753000
F	-3.80934400	-5.13926100	-1.37833800
F	-4.50698300	-2.69858700	-0.67797300
F	-1.03054100	-0.49336500	-2.73556600

F	-0.03128000	1.57437300	-4.03320600
F	-0.32646800	4.12164000	-3.06825500
F	-1.63581100	4.51632700	-0.70472500
F	-2.59551000	2.43504600	0.67118200
F	-4.58253400	0.77326500	2.05328800
F	-7.08339400	1.49500400	1.56276000
C	2.96143100	1.00156600	-0.56494500
C	2.77427100	0.00636100	0.54542200
H	3.15571800	-0.98709200	0.22476700
H	1.68630900	-0.15094700	0.64408400
C	3.33042000	0.35436400	1.91242400
O	3.92177400	1.39210700	2.17675300
C	2.48381200	0.61162400	-1.92521600
H	1.57262300	0.01190800	-1.79044800
H	2.24049600	1.49920200	-2.51987800
C	3.52066200	-0.26137800	-2.69598300
H	3.70531700	-1.18503300	-2.12568000
H	3.00645400	-0.56848400	-3.62067000
C	4.83435800	0.43603900	-3.04390400
H	5.39388900	0.75687100	-2.14902400
H	5.48927900	-0.25137200	-3.60157400
H	4.67059700	1.32834400	-3.66621900
O	3.10096100	-0.59735800	2.78979500
C	3.54297700	-0.37645800	4.16078500
H	3.22809200	0.63292100	4.45901000
H	4.64407700	-0.40441700	4.16018500
C	2.93942200	-1.44715900	5.04046600
H	3.21022900	-2.45423600	4.69080300
H	3.32255500	-1.32573400	6.06562700
H	1.84469600	-1.35990200	5.07665600

C	1.73911000	6.76438500	1.12154300
C	1.13126600	6.12882000	0.02185500
C	1.80941300	5.16914800	-0.72360800
C	3.11811700	4.83584400	-0.34942900
C	3.76132800	5.45481900	0.72891800
C	3.06052000	6.41739300	1.45429900
H	0.10556500	6.37915900	-0.25689300
H	1.33531700	4.70386000	-1.58822400
H	4.78993500	5.19480800	0.98492800
H	3.55135700	6.91037100	2.29690400
C	0.97893700	7.79394700	1.91447800
H	1.58220400	8.20690400	2.73498400
H	0.65880600	8.62834400	1.26916000
H	0.06433600	7.35578700	2.34672900
N	3.53901200	2.12984900	-0.27357300
H	3.85794200	2.19472100	0.72944400
O	5.41237300	3.68344500	-1.02904100
O	3.39555800	3.41378200	-2.58096800
S	3.98059700	3.59300400	-1.26172900
Si	4.18239900	-4.42475300	-0.37152300
C	3.74359900	-5.49146800	-1.87646200
C	3.60669500	-4.77256400	-3.22796400
H	2.79345000	-6.00431400	-1.64039200
H	4.49916700	-6.29664000	-1.94414600
H	3.34672500	-5.47658800	-4.03523800
H	2.81241000	-4.01041400	-3.19258900
H	4.53696300	-4.26266700	-3.52518100
C	3.62212000	-5.32934800	1.19323600
C	3.91057300	-4.61241200	2.52032300
H	4.07834400	-6.33688100	1.19259100

H	2.53523800	-5.50453300	1.08796700
H	3.48377800	-5.15419400	3.38062200
H	4.99349100	-4.51449100	2.70377600
H	3.48483300	-3.59591800	2.52384800
C	6.01139500	-3.96499300	-0.25102200
H	6.56091600	-4.89391900	-0.00868400
H	6.12278400	-3.32433100	0.64304800
C	6.65503800	-3.27955200	-1.46578800
H	7.70816100	-3.01597400	-1.27299600
H	6.64260400	-3.92932300	-2.35558300
H	6.12907600	-2.34913200	-1.73422400
O	3.35919100	-2.92961700	-0.47869600
H	2.41258600	-3.03310300	-0.66356900

Int-3$_A$

C	-2.12483200	-1.89944300	-0.59339700
C	-0.78964600	-2.24563600	-0.81726200
C	-3.01979500	-2.96035100	-0.79075900
C	-0.36121500	-3.49058200	-1.26275100
C	-2.63174200	-4.23707400	-1.22360000
C	-1.28688700	-4.51193300	-1.46063500
C	-1.83333500	-0.45234200	1.52736200
C	-0.66092100	0.17311400	1.95585800
C	-2.38618700	-1.32819100	2.47424300
C	-0.09256000	-0.01002000	3.21972100
C	-1.86132800	-1.54306200	3.74977400
C	-0.69568900	-0.87492900	4.12617500
C	-4.12452800	-0.05002700	-0.01790500
C	-4.88567200	0.44970300	1.04377400
C	-4.81621300	-0.13751600	-1.23334600

C	-6.23720200	0.79546200	0.92832900
C	-6.16065200	0.19311500	-1.39803100
C	-6.88037200	0.66877700	-0.30086400
C	-1.88705900	0.86973800	-0.91533200
C	-2.00273900	2.17763800	-0.41982200
C	-1.34968600	0.79374700	-2.20453600
C	-1.59604300	3.31705300	-1.11274900
C	-0.90648000	1.90853000	-2.92872000
C	-1.02755600	3.18078100	-2.37900000
B	-2.50026600	-0.38416200	-0.00001600
F	-6.75762400	0.07104300	-2.58529900
F	-8.16492800	0.99827600	-0.42870900
F	1.05166900	0.61524400	3.54672400
F	0.04579100	0.99880000	1.14277600
F	-0.15092300	-1.08318600	5.32886600
F	-2.44554500	-2.38608800	4.59947700
F	-3.48541300	-2.02727100	2.16624800
F	-4.17164300	-0.57387000	-2.33111400
F	0.19162500	-1.32905300	-0.61663000
F	0.95180700	-3.72614900	-1.49751100
F	-0.88928800	-5.71561100	-1.86852500
F	-3.53979400	-5.19522900	-1.39599200
F	-4.32843900	-2.81767200	-0.56950600
F	-1.18920300	-0.38129200	-2.83319200
F	-0.33288600	1.75236700	-4.12706500
F	-0.57976700	4.25541000	-3.04184500
F	-1.72246000	4.53599400	-0.56914200
F	-2.51345800	2.38570900	0.80178200
F	-4.34832200	0.64009700	2.25908200
F	-6.90949600	1.26021500	1.98363100

C	3.13268200	0.89248800	-0.29376600
C	2.97791800	-0.30403300	0.59540900
H	3.35124500	-1.20899300	0.05743700
H	1.90489700	-0.50620900	0.71938800
C	3.65839900	-0.26421700	1.94744500
O	4.52128900	0.54070900	2.26521600
C	2.26578200	0.98078200	-1.50476400
H	1.23277500	0.90040400	-1.12798300
H	2.38367500	1.95438900	-1.99108000
C	2.49211200	-0.14395400	-2.54866400
H	2.30246700	-1.12298400	-2.08719500
H	1.70276900	-0.00645400	-3.30362400
C	3.87160800	-0.11796900	-3.20270300
H	4.67551200	-0.31735900	-2.47421800
H	3.94069000	-0.89484500	-3.97941200
H	4.08294600	0.85568500	-3.67054200
O	3.22610500	-1.22623800	2.73270800
C	3.78148900	-1.30708300	4.07396300
H	3.63761900	-0.33016600	4.55847100
H	4.86463100	-1.48254300	3.98121500
C	3.07235500	-2.42122400	4.80927800
H	3.17265200	-3.37753100	4.27392200
H	3.51840200	-2.53787700	5.80904500
H	2.00548800	-2.19098800	4.93782900
C	1.20262400	6.18854200	0.64667900
C	1.19724200	5.67452200	-0.66388600
C	2.20013000	4.81829500	-1.10821600
C	3.23128200	4.47720000	-0.22238200
C	3.28738300	4.99496600	1.07757900
C	2.26642600	5.84455900	1.49973200

H	0.38254100	5.92951600	-1.34316900
H	2.18248400	4.42859300	-2.12701800
H	4.11569300	4.74117400	1.74158900
H	2.29476200	6.24753000	2.51495600
C	0.07489100	7.06846400	1.11274400
H	0.22656100	7.42150300	2.14222800
H	-0.03733300	7.94741800	0.45738200
H	-0.87640500	6.51324300	1.07031800
N	3.99885500	1.80067900	0.04562500
H	4.50733900	1.61307200	0.94569200
O	5.73895400	3.61635700	-0.02987500
O	4.43717200	3.14520400	-2.18369500
S	4.50102300	3.36637200	-0.74783500
Si	4.83262700	-4.05820500	-1.28782100
H	4.95301500	-3.82473900	-2.76230900
C	6.42990200	-3.55603400	-0.42452900
C	6.84648500	-2.08456500	-0.57046000
H	7.22578600	-4.21183000	-0.82373200
H	6.33678900	-3.82312800	0.64407900
H	7.81130100	-1.88329000	-0.07705900
H	6.95414800	-1.79510300	-1.62895300
H	6.10587400	-1.40462400	-0.11834800
C	4.40679300	-5.86832900	-0.97006500
C	3.23029500	-6.44259200	-1.77741300
H	4.22641500	-5.98815900	0.11423800
H	5.32372900	-6.45040800	-1.18125400
H	3.05927100	-7.50487600	-1.53942300
H	2.28576800	-5.91143500	-1.57976200
H	3.41749600	-6.37685700	-2.86185700
O	3.62712000	-3.03084800	-0.67194600

H	2.71001500	-3.29241900	-0.84610300

P_B

C	2.31140200	1.07154100	0.02331200
C	2.14771600	1.89507500	1.14753400
C	1.51009700	1.42456500	-1.06711100
C	1.23896400	2.95152700	1.22467200
C	0.59517500	2.48283800	-1.03920800
C	0.44925900	3.24517500	0.11409800
C	4.87325700	0.82389900	0.04150500
C	5.72800900	1.05196900	1.12551800
C	5.17555600	1.57878700	-1.09962900
C	6.82336400	1.92267300	1.07430000
C	6.25443500	2.45639700	-1.19885000
C	7.08906700	2.63197700	-0.09451400
C	3.40018200	-1.18659200	-1.21465400
C	4.46324900	-1.60789300	-2.02392800
C	2.21368700	-1.89594800	-1.43002200
C	4.35356200	-2.62467400	-2.98261000
C	2.05958700	-2.92031200	-2.35953600
C	3.14254400	-3.29059000	-3.15551100
C	3.36977200	-1.13285600	1.35342200
C	4.41263000	-2.02932400	1.62921000
C	2.23424300	-1.33048000	2.14183200
C	4.36712100	-3.00667800	2.62474500
C	2.13373500	-2.30153400	3.14193700
C	3.20736700	-3.15210900	3.38807500
B	3.49593400	-0.10279100	0.04997100
F	0.88040100	-3.55556100	-2.49146100
F	3.02035500	-4.26577800	-4.05614900

F	7.60706500	2.08946400	2.14279100
F	5.53871500	0.44304200	2.30540700
F	8.12534600	3.46918400	-0.15791300
F	6.48814100	3.13442900	-2.32616500
F	4.39719700	1.47060900	-2.19204900
F	1.11095400	-1.60633200	-0.69535400
F	2.87971700	1.67296200	2.24714000
F	1.10689600	3.67055300	2.34221600
F	-0.45325000	4.23794600	0.16344400
F	-0.18214100	2.73900000	-2.10595500
F	1.54865200	0.74319000	-2.22389500
F	1.11642500	-0.59161400	1.96759600
F	0.99138700	-2.44238000	3.83060900
F	3.12918100	-4.09011600	4.33267900
F	5.40619700	-3.81595100	2.83824400
F	5.54018600	-1.97838200	0.90531600
F	5.67928300	-1.06149300	-1.91930300
F	5.40620000	-2.96880600	-3.72574300
C	-5.87937100	2.03914300	0.70117500
C	-5.75645100	3.31390600	-0.15340400
H	-5.87093800	4.20015500	0.48316700
H	-6.57935100	3.33983500	-0.89068100
C	-4.46398800	3.44668100	-0.94418500
O	-3.89535800	2.49795400	-1.46543800
C	-7.26908000	1.90293600	1.34111100
H	-8.01519200	1.84720100	0.52963300
H	-7.30665700	0.93738200	1.87213200
C	-7.65981600	3.01123100	2.33142000
H	-7.71819300	3.98315500	1.81113800
H	-8.68978500	2.80088500	2.66494800

C	-6.75178000	3.13193100	3.55842400
H	-5.73021900	3.45344000	3.29797800
H	-7.15020900	3.87333000	4.26804000
H	-6.66879800	2.17093300	4.09240200
O	-4.05997500	4.69495300	-1.04585000
C	-2.88450600	4.98292500	-1.86317900
H	-3.20151900	4.92722400	-2.91697600
H	-2.13517500	4.20192700	-1.68881200
C	-2.37946700	6.35626500	-1.48615500
H	-2.05498000	6.37749800	-0.43556300
H	-1.50991200	6.60599800	-2.11306400
H	-3.15042300	7.12692100	-1.63944600
C	-8.98481300	-3.17334600	-0.85358000
C	-8.43228500	-3.26978500	0.43300900
C	-7.34584200	-2.48135600	0.81795700
C	-6.81523600	-1.57862100	-0.10624000
C	-7.34055200	-1.45242400	-1.39865200
C	-8.42065100	-2.25162200	-1.75842900
H	-8.85652500	-3.97555900	1.15120600
H	-6.91986100	-2.56052100	1.81935000
H	-6.91839800	-0.73530900	-2.10472100
H	-8.83922500	-2.15942100	-2.76405100
C	-10.15348300	-4.02730000	-1.27003100
H	-9.88953600	-4.65897500	-2.13417300
H	-10.48789400	-4.68599000	-0.45654000
H	-11.00812700	-3.40299600	-1.57834400
N	-5.58437100	0.88841000	-0.19991800
H	-4.83513000	1.13887800	-0.87812500
O	-4.26730100	-1.19103000	-0.50856500
O	-5.21772400	-0.71209600	1.82275200

S	-5.39620600	-0.63006200	0.37610700
H	-5.11217500	2.07482500	1.49334800
Si	-2.43290100	-1.47640100	-0.34860400
C	-2.02858600	-1.66731900	-2.16569400
C	-1.96924500	-0.38034100	-3.00457900
H	-1.05896700	-2.19154500	-2.21174700
H	-2.76741200	-2.37675900	-2.58119800
H	-1.80377000	-0.61740600	-4.06750700
H	-2.89687800	0.21071700	-2.93809000
H	-1.14237200	0.27252900	-2.68932800
C	-2.39323800	-3.07220600	0.63989500
C	-0.98614300	-3.62459400	0.93726000
H	-2.98554000	-3.81549200	0.07568100
H	-2.94216600	-2.89785900	1.58280200
H	-1.05314900	-4.59322300	1.45705900
H	-0.40178100	-3.77824500	0.01783300
H	-0.41155100	-2.94636800	1.58437100
C	-1.72295100	0.04647500	0.47396900
H	-0.64547100	-0.01253200	0.23806900
H	-2.08749100	0.92876500	-0.08203600
C	-1.89557800	0.22308700	1.99168100
H	-1.42200500	1.16001500	2.32441400
H	-2.95259400	0.24895600	2.29738600
H	-1.40460500	-0.58924000	2.54727400

Et_3SiOH

Si	0.14601700	-0.19293000	0.01375000
C	-1.13943300	-1.49054300	-0.50374700
C	-2.58733000	-1.24990500	-0.05000900
H	-0.78441800	-2.46357000	-0.11264500

H	-1.09477800	-1.59516900	-1.60415200
H	-3.24367300	-2.09952900	-0.30175400
H	-2.64602200	-1.09573900	1.03978800
H	-3.01948600	-0.35458100	-0.52487300
C	1.85312400	-0.83352200	-0.51191400
C	3.05047200	-0.00365800	-0.02459400
H	1.86615600	-0.91471100	-1.61522800
H	1.94599900	-1.87441400	-0.14784900
H	4.01260800	-0.45930500	-0.31196800
H	3.03695300	1.01642700	-0.44242700
H	3.04472500	0.09954200	1.07268400
C	-0.17061700	1.52995600	-0.69548400
H	-0.34447500	1.42486500	-1.78277800
H	0.77744100	2.09088100	-0.60604900
C	-1.30264900	2.34143700	-0.04543200
H	-1.36789100	3.35752100	-0.46907700
H	-2.28588400	1.86549200	-0.18869600
H	-1.14501800	2.43817000	1.03984100
O	0.09617900	0.03164300	1.68873300
H	0.21269300	-0.75059200	2.23989500

P_A

C	3.76810300	1.24183500	-1.85259500
C	3.94743600	2.76814000	-1.76833100
H	3.12128000	3.26092900	-2.29410800
H	4.87892900	3.05579900	-2.28936600
C	4.03589400	3.35053300	-0.36893800
O	4.53688700	2.76562300	0.58247300
C	3.86684200	0.73480500	-3.30126700
H	4.89567800	0.93080400	-3.65085900

H	3.74414000	-0.35758200	-3.29266100
C	2.85785700	1.33455200	-4.29297200
H	3.03571300	2.41769700	-4.41409800
H	3.08291500	0.89401600	-5.27890300
C	1.38789600	1.08320500	-3.95052200
H	1.06859100	1.60768800	-3.03800300
H	0.73390400	1.43649900	-4.76280700
H	1.18002800	0.01062800	-3.80527800
O	3.57151200	4.58091300	-0.30901000
C	3.62728000	5.28726200	0.95860200
H	4.66873600	5.61365100	1.11379300
H	3.36451900	4.58309200	1.75826600
C	2.66323500	6.44952300	0.88061300
H	1.63738700	6.08337600	0.73044500
H	2.69347500	7.01841700	1.82260500
H	2.92139400	7.13059100	0.05548000
C	9.14041100	-1.47560800	1.03754900
C	8.78438200	-1.85108500	-0.26923000
C	7.48342500	-1.68460000	-0.74245000
C	6.52664400	-1.13168400	0.11417500
C	6.83982900	-0.75178200	1.42423700
C	8.14873800	-0.92389000	1.86955500
H	9.53834100	-2.28503300	-0.93059400
H	7.21073600	-1.98541800	-1.75557600
H	6.07792000	-0.33668700	2.08549200
H	8.40451300	-0.62772100	2.89002800
C	10.54121500	-1.68547900	1.54983300
H	10.85029100	-0.87161300	2.22287400
H	10.60463900	-2.62521700	2.12555600
H	11.26849800	-1.75302500	0.72801700

N	4.85298100	0.64566100	-0.99795400
H	5.01518900	1.26692200	-0.17437800
O	4.01060500	-1.14401900	0.71694900
O	4.61547200	-1.81773100	-1.65110200
S	4.89472100	-0.91659500	-0.54259600
H	2.78310400	0.98909300	-1.42517900
Si	2.26700600	-1.51946900	1.11121500
H	1.56600800	-0.52327300	0.27047400
C	1.98835100	-3.31505800	0.67164000
C	2.28939600	-1.18863200	2.95293400
H	1.30671100	-1.52219500	3.32823300
C	2.58867100	0.24752400	3.40789600
H	3.02630600	-1.89407700	3.37945700
H	2.79917300	-3.89026400	1.15632100
H	1.07269800	-3.59118200	1.22365600
C	1.85961200	-3.68776000	-0.81363100
H	2.79259000	-3.49283700	-1.36281100
H	1.05727000	-3.11929900	-1.30580200
H	1.62501100	-4.75824100	-0.92413400
H	3.51426700	0.64457300	2.95949700
H	1.77603900	0.93893400	3.14243000
H	2.70336300	0.29662000	4.50223800
C	-2.85116900	-0.61972900	-1.51347100
C	-1.65433700	-1.18408500	-1.96757100
C	-3.88455500	-0.64784900	-2.45818000
C	-1.45350800	-1.69593200	-3.24640500
C	-3.73318200	-1.15507000	-3.75594200
C	-2.50815400	-1.68265000	-4.15846200
C	-2.90816600	-1.58589800	0.87801000
C	-1.82407700	-2.10155600	1.58915500

C	-3.97410000	-2.48926100	0.74690300
C	-1.79915400	-3.37043300	2.17279500
C	-3.99942100	-3.76604300	1.31066100
C	-2.89197100	-4.21938900	2.02963200
C	-4.33949000	0.76246400	0.39950200
C	-5.22819100	0.56520700	1.46186300
C	-4.59342300	1.91414100	-0.35727100
C	-6.31101500	1.41117200	1.73016800
C	-5.65810800	2.78537400	-0.13024000
C	-6.52829600	2.53065100	0.93043200
C	-1.76788800	0.92527900	0.53715700
C	-1.61798900	1.23356700	1.89694500
C	-0.91101500	1.63605000	-0.30904100
C	-0.66542800	2.11939600	2.40175900
C	0.06626300	2.52323100	0.15411000
C	0.19173900	2.76885100	1.51607400
B	-2.97670300	-0.12707300	0.07250100
F	-5.84422200	3.85902600	-0.90267000
F	-7.55152700	3.34945000	1.17552700
F	-0.70691800	-3.78682000	2.83779300
F	-0.68229000	-1.38430000	1.76254500
F	-2.88214500	-5.43745700	2.56923400
F	-5.05703900	-4.56190600	1.15430400
F	-5.05861900	-2.13559300	0.04523400
F	-3.77812400	2.22873200	-1.38053600
F	-0.58483200	-1.24632200	-1.13910100
F	-0.25759900	-2.19031500	-3.60517500
F	-2.34528200	-2.16618200	-5.38989700
F	-4.75770000	-1.14246500	-4.60992100
F	-5.10609000	-0.18557700	-2.16934600

F	-0.95844700	1.49401800	-1.64241300
F	0.90751700	3.12808500	-0.70777000
F	1.14128400	3.59771500	1.97508700
F	-0.53520600	2.32170800	3.71588000
F	-2.40025700	0.63684100	2.80594000
F	-5.08553800	-0.46482700	2.31006700
F	-7.12803800	1.16109600	2.75619900

HEt_2SiOH_2

Si	0.00000000	0.08832300	0.29683600
H	0.00000100	0.25857600	1.78924000
C	-1.55239700	-0.83063400	-0.25072700
C	-2.87179300	-0.12983600	0.10496900
H	-1.52327100	-1.84687300	0.18453300
H	-1.48142600	-0.97361400	-1.34487500
H	-3.74773900	-0.68403300	-0.27067800
H	-2.99402400	-0.02602400	1.19639800
H	-2.90970100	0.88336000	-0.32697200
C	1.55239700	-0.83063400	-0.25072700
C	2.87179300	-0.12983600	0.10496900
H	1.48142600	-0.97361300	-1.34487500
H	1.52327200	-1.84687300	0.18453200
H	3.74773900	-0.68403300	-0.27067800
H	2.90970100	0.88336100	-0.32697200
H	2.99402400	-0.02602400	1.19639800
O	-0.00000100	1.61843200	-0.40715000
H	0.00000400	2.37745400	0.18453800

9.2. Cartesian coordinate of Figure 3D, 3E [def2-TZVP]

$[Et_2SiH]^+[B(C_6F_5)_4]^-$

Si	3.85797800	0.69625700	-0.14885600
H	3.23973300	1.18065700	-1.39003000
C	4.54870300	-1.02116500	-0.22793400
C	4.81488600	1.97408800	0.79027200
H	5.03880200	1.57525700	1.78383800
C	4.18537200	3.37306600	0.88409700
H	5.78338000	2.03054400	0.27405600
H	5.05101300	-1.25483700	0.71314900
H	3.72837700	-1.73102600	-0.34697000
C	5.52738300	-1.16140900	-1.41489800
H	6.37322300	-0.47506900	-1.33706600
H	5.02964900	-0.98071800	-2.36846900
H	5.93254200	-2.17303500	-1.44362100
H	3.98357400	3.79195300	-0.10255100
H	3.24570500	3.35706700	1.43617900
H	4.85863800	4.05718100	1.40135800
C	-0.69944600	-1.45690100	-1.00264200
C	0.47250800	-1.88594300	-1.61640700
C	-1.78255600	-2.30784300	-1.21647000
C	0.59295500	-3.01767200	-2.40125000
C	-1.71538600	-3.45504600	-2.00157500
C	-0.52188600	-3.81565200	-2.60275900
C	0.27136800	-0.70451600	1.25407800
C	1.56383100	-0.42114600	1.59813300
C	-0.24735800	-1.72174800	2.07210500
C	2.32382500	-0.94783300	2.62753300

C	0.44150100	-2.31710700	3.11768800
C	1.74375100	-1.92675200	3.40963300
C	-2.15001400	0.41638800	0.48466700
C	-2.52606400	0.72531300	1.78818100
C	-3.11420100	0.73726200	-0.47143800
C	-3.75969000	1.26593500	2.13083400
C	-4.35568200	1.27639700	-0.17836000
C	-4.68647500	1.54177500	1.14158900
C	-0.04415000	1.24131600	-0.75078600
C	0.23306100	2.37428700	0.01476700
C	0.06732400	1.45432400	-2.12464700
C	0.60834400	3.60322500	-0.50337300
C	0.43918100	2.67059500	-2.69040600
C	0.71437400	3.75566700	-1.87716000
B	-0.66634400	-0.11914300	-0.01997500
F	-5.23273600	1.54566600	-1.15121300
F	-5.87542400	2.06263700	1.45094000
F	3.57926300	-0.53493900	2.85370400
F	2.30258100	0.61240500	0.85344400
F	2.41956800	-2.48492200	4.40948600
F	-0.13199000	-3.26416500	3.85367500
F	-1.48792800	-2.16488900	1.86316100
F	-2.85241200	0.52580000	-1.77570600
F	1.61569700	-1.16132100	-1.45499000
F	1.76773300	-3.34664400	-2.95786600
F	-0.44108600	-4.91523000	-3.35325400
F	-2.79604900	-4.22162300	-2.17119200
F	-2.97705700	-2.07226300	-0.65478700
F	-0.19045000	0.47676000	-3.00729800
F	0.53366400	2.79930700	-4.01757500

F	1.08079700	4.92656200	-2.40246200
F	0.87421000	4.63740800	0.30557600
F	0.14835700	2.30585400	1.36190200
F	-1.68518800	0.51991900	2.82511900
F	-4.05348200	1.52886700	3.40968800

$[Et_3Si]^+[B(C_6F_5)_4]^-$

Si	4.03783400	0.27561900	0.22821400
C	4.76323300	-1.43655300	0.24132700
C	4.75604300	1.41140900	1.51322500
H	4.76330800	0.88630200	2.47124700
C	4.11767300	2.80148400	1.65954000
H	5.81333200	1.51256500	1.23204400
H	5.80663600	-1.23916500	-0.04950800
H	4.82429000	-1.78960700	1.27206400
C	4.17218800	-2.51509800	-0.67792000
H	4.11950400	-2.18785900	-1.71602300
H	3.16359400	-2.79224400	-0.37578900
H	4.78851700	-3.41423100	-0.64424800
H	4.13829100	3.36136800	0.72380300
H	3.07930100	2.73739800	1.98427900
H	4.65733300	3.38915300	2.40296000
C	-0.99097700	-1.36971300	-1.09774600
C	0.12979600	-1.86664600	-1.75427500
C	-2.15466200	-2.09135400	-1.35775700
C	0.13175000	-2.94388600	-2.62118100
C	-2.20600700	-3.18034300	-2.22286100
C	-1.05782100	-3.61186400	-2.86399700
C	0.03919800	-0.87142200	1.21371300

C	1.35136500	-0.75140000	1.59627600
C	-0.62609800	-1.83838100	1.98340200
C	1.98233900	-1.37407700	2.65983800
C	-0.05992300	-2.53083900	3.04209100
C	1.25920000	-2.28919200	3.39956800
C	-2.23888500	0.55074700	0.50941500
C	-2.58202400	0.84113400	1.82675700
C	-3.16404100	1.01542800	-0.42645300
C	-3.75182200	1.49333400	2.19807800
C	-4.34201600	1.66894800	-0.10469300
C	-4.64425900	1.90912900	1.22656800
C	-0.06330000	1.22727300	-0.66694200
C	0.33008400	2.27046500	0.17161100
C	0.02561400	1.53870900	-2.02369400
C	0.77406400	3.50877300	-0.26430500
C	0.46380900	2.76766500	-2.50799200
C	0.84087900	3.76445300	-1.62520700
B	-0.81439800	-0.11507300	-0.02678300
F	-5.18425400	2.07310600	-1.06170800
F	-5.77169700	2.53855600	1.56368900
F	3.25781200	-1.10670200	2.98049700
F	2.23175800	0.15498800	0.86359000
F	1.82210700	-2.92428200	4.42416900
F	-0.77197100	-3.41668100	3.73263500
F	-1.90249200	-2.12562900	1.71964800
F	-2.92621900	0.84115300	-1.74075900
F	1.33611400	-1.27058100	-1.55309000
F	1.26367900	-3.34461900	-3.21861200
F	-1.09114300	-4.65671500	-3.69295000
F	-3.35868800	-3.82265600	-2.43301100

F	-3.31793300	-1.77843300	-0.76812300
F	-0.32030200	0.65134400	-2.96952900
F	0.53003700	2.99252700	-3.82449200
F	1.27115400	4.94619700	-2.07313400
F	1.13956600	4.45492400	0.61099000
F	0.28242900	2.10449400	1.51233500
F	-1.77076600	0.50227700	2.85234600
F	-4.01668800	1.72823600	3.48883300
C	3.70416100	1.01634900	-1.43804200
H	3.10222900	1.91850700	-1.31350400
H	3.10054400	0.32246000	-2.02471800
C	5.01271600	1.35324700	-2.18507500
H	4.78272200	1.79212000	-3.15634000
H	5.62510500	2.07426800	-1.64004600
H	5.62141900	0.46545200	-2.36687900

Path A: $[Et_2SiH]^+$, $[B(C_6F_5)_4]^-$, Ethyl 3-oxohexanoate

[Ethyl 3-oxohexanoate]

C	1.18920100	-0.46578000	0.24475900
O	1.36447000	-1.49481600	-0.36133300
C	-0.05784100	-0.30353500	1.12922700
H	0.18986300	0.27949000	2.01870300
H	-0.40954200	-1.29054800	1.41678100
C	-1.14985300	0.45156300	0.39336100
O	-1.03258100	1.57139200	-0.03956100
C	2.12479500	0.72031600	0.17293500
H	1.51310300	1.56600900	-0.16155200
H	2.41647900	0.97571000	1.19942700
C	3.35288900	0.52715300	-0.71345500

H	3.03375400	0.12314800	-1.67681400
H	3.78703600	1.51018600	-0.91437700
C	4.41846500	-0.38348400	-0.10368900
H	4.02784600	-1.38639500	0.07027600
H	5.28107700	-0.47229400	-0.76677800
H	4.77606400	0.01391600	0.85035500
O	-2.26436200	-0.28403800	0.28057100
C	-3.37609600	0.32924800	-0.41885100
H	-3.04608500	0.59676500	-1.42338700
H	-3.64616600	1.24996100	0.10013700
C	-4.51078500	-0.66838100	-0.43876700
H	-4.82360100	-0.92723700	0.57358100
H	-5.36656700	-0.23811500	-0.96210700
H	-4.21812800	-1.58349900	-0.95478200

$[B(C_6F_5)_4]^-$

C	1.05875200	0.09040900	-1.28544300
C	1.34026600	1.21691400	-2.05379700
C	1.86922900	-1.00591900	-1.58218200
C	2.30846200	1.25023500	-3.05211300
C	2.84394000	-1.01826400	-2.56606400
C	3.06608300	0.12494300	-3.31566200
C	-1.05865500	-1.28550300	-0.09040500
C	-1.86910700	-1.58232500	1.00591500
C	-1.34006300	-2.05391300	-1.21690500
C	-2.84372000	-2.56630900	1.01825500
C	-2.30814200	-3.05234300	-1.25022000
C	-3.06575900	-3.31595300	-0.12493900
C	-1.05877000	1.28546800	0.09027800
C	-1.34033000	2.05380100	1.21678300

C	-1.86916500	1.58226200	-1.00609400
C	-2.30849300	3.05215100	1.25007000
C	-2.84385700	2.56616500	-1.01846700
C	-3.06604500	3.31574600	0.12474100
C	1.05858600	-0.09028100	1.28552800
C	1.34011500	-1.21679900	2.05385500
C	1.86895100	1.00609600	1.58239600
C	2.30821100	-1.25008900	3.05227200
C	2.84359000	1.01845800	2.56635000
C	3.06573000	-0.12475600	3.31593700
B	-0.00002400	0.00002600	-0.00002600
F	-3.57480200	2.80139400	-2.12050600
F	-4.00434200	4.27391000	0.14053300
F	-3.57472200	-2.80156600	2.12024900
F	-1.72642000	-0.88696100	2.15176000
F	-4.00395800	-4.27421400	-0.14075600
F	-2.52076600	-3.76015700	-2.37199800
F	-0.68437800	-1.86806200	-2.37799400
F	-1.72632600	0.88696400	-2.15196200
F	0.68457600	2.37800900	-1.86798200
F	2.52117700	2.37202300	-3.75988600
F	4.00439100	0.14076400	-4.27381600
F	3.57495500	-2.12026400	-2.80125100
F	1.72643300	-2.15178100	-0.88685900
F	1.72621200	2.15194500	0.88704700
F	3.57453300	2.12048700	2.80162600
F	4.00398800	-0.14056500	4.27414200
F	2.52098300	-2.37191000	3.75997300
F	0.68459900	-2.37796000	1.86785300
F	-0.68472400	1.86791400	2.37791400

F	-2.52126800	3.75988600	2.37186900

$[Et_2SiH]^+$

Si	0.00032300	-0.20221400	-0.11225900
H	0.00175400	-1.66949100	-0.27465500
C	-1.60149900	0.61717600	0.23420900
C	-2.87149800	-0.12504300	-0.22334500
H	-1.55742400	1.64576100	-0.13976100
H	-1.59655700	0.72655900	1.33411000
H	-3.75125200	0.40885200	0.13316700
H	-2.93327200	-0.17945300	-1.30979400
H	-2.91430900	-1.13940300	0.17353200
C	1.60039600	0.67635200	-0.27088600
C	2.87221600	-0.14598300	0.01069900
H	1.55499900	1.59710100	0.32070800
H	1.59383300	1.02567600	-1.31955400
H	3.75062700	0.45503800	-0.22013200
H	2.93546500	-0.43841600	1.05837700
H	2.91620500	-1.04783400	-0.60003400

Path C: $[Et_2SiH]^+$, $[BAr^F{}_4]^-$, Ethyl 3-oxohexanoate

$[Et_2SiH]^+$, Ethyl 3-oxohexanoate is the same as the structure of Path A

$[BAr^F{}_4]^-$

C	-1.00486000	0.41243100	1.25085100
C	-1.95839500	1.43051400	1.06936300
C	-1.01265500	-0.20750200	2.50161900
C	-2.84463700	1.80852200	2.06931600

C	-1.90303700	0.16239700	3.51336400
C	-2.82411900	1.17516600	3.30971100
C	-0.98499800	-0.30808700	-1.29052400
C	-0.86739100	0.30316500	-2.54011600
C	-2.05136800	-1.21503300	-1.15408000
C	-1.74488700	0.02766400	-3.59236400
C	-2.92656600	-1.49870400	-2.19425500
C	-2.77941100	-0.87776800	-3.43228800
C	0.91311400	-1.32279700	0.38570800
C	0.80196900	-2.57163900	-0.22853900
C	1.90973100	-1.21563100	1.37225400
C	1.61888500	-3.65073600	0.11964600
C	2.72136500	-2.28376100	1.73130300
C	2.58327600	-3.51943900	1.10397400
C	1.06585500	1.21480700	-0.33368600
C	2.08871100	1.00050100	-1.27522000
C	1.06412300	2.46732400	0.28272300
C	3.03068400	1.97204100	-1.58671900
C	2.01001800	3.45058600	-0.02035400
C	2.99888800	3.21538000	-0.95968700
B	-0.00249300	-0.00084400	0.00299800
H	-0.31598300	-1.01017200	2.70516300
H	-2.01893900	1.93185500	0.11168200
H	-3.51685100	1.45958300	4.08761400
H	0.31546200	2.69464500	1.03042700
H	2.15879700	0.03919400	-1.76831700
H	-0.07970400	1.02548200	-2.71043600
H	3.73436200	3.97103500	-1.19188700
H	-3.46274100	-1.08793700	-4.24140600
H	-2.21102800	-1.70174700	-0.20019300

H	2.05758700	-0.26634500	1.87158100
H	0.06708200	-2.72065900	-1.00883100
H	3.21293600	-4.35261000	1.37829200
C	1.42023600	-4.96474700	-0.58190700
C	3.80239700	-2.10603900	2.76003300
C	-1.85463700	-0.57335600	4.82269800
C	-3.81188100	2.93685500	1.84620400
C	-4.02019500	-2.51366800	-2.01452200
C	-1.54968400	0.74109500	-4.90045800
C	4.07356300	1.71474700	-2.63784700
C	1.94169100	4.76573300	0.70343000
F	2.88725200	5.63956200	0.29383200
F	2.10341700	4.62026900	2.04016400
F	0.74759600	5.37916400	0.53333600
F	3.53830400	-1.11052600	3.62843600
F	5.00077500	-1.81344100	2.19329300
F	4.00012300	-3.22635600	3.49437100
F	0.22003000	-5.52124300	-0.29027400
F	2.35766500	-5.88089200	-0.25362200
F	1.45830000	-4.83518100	-1.92779200
F	5.25324000	2.31228600	-2.34625300
F	4.33103500	0.40380800	-2.80980400
F	3.70039000	2.19570500	-3.85125100
F	-1.64319600	2.08522600	-4.76645500
F	-2.45790700	0.38243600	-5.83425400
F	-0.32966700	0.50011600	-5.43456000
F	-3.33758500	4.11468400	2.32623800
F	-4.08856900	3.13922600	0.54348600
F	-4.99497800	2.72848600	2.47151300
F	-2.71638800	-0.07542800	5.73641700

F	-2.15897700	-1.88576700	4.68058400
F	-0.62519900	-0.53145700	5.38588700
F	-5.13576000	-2.19450800	-2.71254300
F	-4.39238100	-2.65905700	-0.72790900
F	-3.64676700	-3.74469000	-2.44800300

$[Et_2SiH]^+[B(Ar^F)_4]^-$

C	-0.55888800	-1.01578200	-1.26673800
C	0.75505900	-1.27238700	-1.68735200
C	-1.55939600	-1.73614300	-1.92645100
C	1.05289300	-2.17407800	-2.70294800
C	-1.27128100	-2.64217000	-2.94676000
C	0.03718300	-2.87130600	-3.34576600
C	0.14688900	-0.47437600	1.18730600
C	1.10832900	0.29831300	1.81742700
C	0.09163100	-1.81959600	1.60486200
C	1.97745700	-0.24666300	2.80394600
C	0.92047900	-2.37386300	2.59936500
C	1.87774200	-1.60578800	3.20408600
C	-2.40548700	0.04289000	0.45143300
C	-2.85470500	-0.40970900	1.69169600
C	-3.39356300	0.55752400	-0.40709200
C	-4.20250800	-0.37448400	2.05836000
C	-4.73485600	0.59276300	-0.05506700
C	-5.15492600	0.12396900	1.18829900
C	-0.44174700	1.59608000	-0.44462900
C	-0.71619500	2.64115800	0.44990500
C	0.16524800	1.96745900	-1.64831700
C	-0.38037700	3.96358900	0.17780500
C	0.49298700	3.29209900	-1.93608400

C	0.23150000	4.30302100	-1.02257200
B	-0.83662800	0.04965200	-0.03735100
H	-2.59473500	-1.59544400	-1.64562700
H	1.57815400	-0.75151600	-1.21191400
H	0.26018700	-3.57231000	-4.13561200
H	0.38236400	1.21466900	-2.39419900
H	-1.22260600	2.42263700	1.38340300
H	1.22229600	1.34009900	1.55023600
H	0.48390000	5.32894400	-1.24418000
H	2.53928900	-2.01531700	3.95281000
H	-0.62305100	-2.47726200	1.12272200
H	-3.10488400	0.94432900	-1.37612600
H	-2.15233400	-0.79511900	2.42044800
H	-6.19835000	0.15100500	1.46526400
C	-4.59074100	-0.89340800	3.41568300
C	-5.75000400	1.19401500	-0.99218900
C	-2.40988300	-3.38835300	-3.59253300
C	2.49265800	-2.41934900	-3.05961500
C	0.74633400	-3.83108900	2.96621300
C	2.94551500	0.55343900	3.37503200
C	-0.65149800	5.01277200	1.22152000
C	1.18476500	3.60733600	-3.23567500
F	1.15247500	4.91972500	-3.53478300
F	0.63994700	2.94612400	-4.27561000
F	2.49418400	3.25008900	-3.19746900
F	-5.36347800	1.12530100	-2.27989500
F	-5.96533200	2.50229600	-0.71857700
F	-6.94790600	0.57989500	-0.90107500
F	-4.31616200	-2.21309200	3.54501800
F	-5.89837100	-0.73558300	3.68245400

F	-3.90549300	-0.26888500	4.40402400
F	-0.50641900	6.26483200	0.75337100
F	-1.89436900	4.91598400	1.73291100
F	0.20273100	4.88953200	2.27419900
F	3.10048600	1.79415100	3.10322900
F	3.76607000	0.15143600	4.27694300
F	2.63998600	-3.07754500	-4.22130700
F	3.19490600	-1.26700900	-3.16098000
F	3.12423100	-3.15734500	-2.10874000
F	-2.00981100	-4.14161400	-4.63452200
F	-3.01737900	-4.22059000	-2.71517500
F	-3.36347800	-2.55057100	-4.04965300
F	0.93001300	-4.62313000	1.89596600
F	-0.49194700	-4.06469500	3.43254600
F	1.61949200	-4.21600100	3.91369100
Si	6.23701100	0.15954600	0.65507400
H	7.32114800	0.17579100	1.66903800
C	6.24668200	1.71689200	-0.37309700
C	6.21255200	3.03022000	0.42359000
H	5.40607500	1.67655600	-1.07372300
H	7.14804900	1.68207700	-0.99629700
H	6.24231000	3.89506600	-0.24116100
H	5.30299700	3.11195400	1.02188100
H	7.06283400	3.10882000	1.10439100
C	6.26573700	-1.41331400	-0.34665000
C	6.17697300	-2.71188200	0.46959100
H	7.19543800	-1.40124000	-0.92798400
H	5.45909200	-1.37467200	-1.08568000
H	6.22023900	-3.58767400	-0.17960200
H	6.99585800	-2.79211700	1.18789600

H	5.24071000	-2.76827400	1.02806100
F	4.85836500	0.16095100	1.55256300

Complex I_A

Si	-4.54055700	-1.11251900	1.39741500
H	-4.21084800	0.28915800	1.71004300
C	-6.28589200	-1.58214400	1.84346300
C	-3.22318900	-2.31970400	1.90389300
H	-3.48914200	-3.31646000	1.53916400
C	-3.01781100	-2.35006100	3.43167800
H	-2.28468200	-2.05082400	1.41209600
H	-6.48264500	-2.57418500	1.42316700
H	-6.27900300	-1.72539600	2.93088800
C	-7.39592800	-0.59494400	1.45388600
H	-7.45274400	-0.45315800	0.37464800
H	-7.23136300	0.38745500	1.89778100
H	-8.36671600	-0.95695000	1.79572100
H	-2.74586400	-1.36682600	3.82050200
H	-3.91415900	-2.68353600	3.95836400
H	-2.20997600	-3.03527700	3.68633800
C	1.13907400	1.42742100	0.58597300
C	-0.19829300	1.40568600	0.96722800
C	1.75350400	2.65950500	0.80870200
C	-0.89636200	2.48448400	1.47693800
C	1.09320200	3.77503400	1.31838900
C	-0.24624000	3.69337300	1.65207400
C	1.88630400	-0.87166700	1.48096500
C	1.07589300	-1.96229500	1.77554200
C	2.67295700	-0.45540200	2.55640600
C	1.07049800	-2.61780500	3.00063100

C	2.70077600	-1.07582500	3.79521600
C	1.89076900	-2.17724500	4.02220300
C	3.39151200	0.26393500	-0.58047000
C	4.55632000	-0.43097500	-0.26531300
C	3.53582900	1.14709300	-1.64995300
C	5.76808400	-0.24277500	-0.92161600
C	4.71989400	1.36722700	-2.33262500
C	5.85544700	0.66493200	-1.96154100
C	1.09997400	-0.73165100	-1.19042900
C	1.50512000	-2.01032200	-1.58122900
C	0.14055200	-0.17594500	-2.02850500
C	0.99080100	-2.70374200	-2.66672800
C	-0.40217500	-0.83367500	-3.12556200
C	0.01590800	-2.10908800	-3.45109200
B	1.88522200	0.02248400	0.07653600
F	4.77855400	2.24496800	-3.34354000
F	7.01170500	0.85596900	-2.60384100
F	0.25804200	-3.67005700	3.20609300
F	0.20103500	-2.45805500	0.86757600
F	1.89182400	-2.79315800	5.20855300
F	3.49360900	-0.62261400	4.77306100
F	3.46890100	0.62013200	2.42106200
F	2.46788600	1.85508400	-2.07826500
F	-0.92565100	0.25985600	0.83252900
F	-2.20503000	2.37191600	1.78997100
F	-0.90476000	4.75578900	2.13130400
F	1.74508200	4.92941900	1.49123400
F	3.05278400	2.85076500	0.54317200
F	-0.34718400	1.06950700	-1.82601700
F	-1.35448200	-0.23329000	-3.87823200

F	-0.51722600	-2.75482200	-4.49654000
F	1.41603800	-3.93705900	-2.96066300
F	2.45198200	-2.65139400	-0.87903900
F	4.57681700	-1.35832900	0.70953500
F	6.85165500	-0.94166500	-0.56011200
C	-4.17033500	-0.51924500	-1.37053000
O	-4.54001300	-1.20904500	-0.39106600
C	-3.51289900	0.80759400	-1.18992300
H	-2.95373600	1.08780100	-2.08055900
H	-2.80453600	0.76867300	-0.35902400
C	-4.54926500	1.89190400	-0.87152800
O	-5.70247000	1.65669900	-0.60598500
C	-4.44255300	-1.01618900	-2.73825700
H	-5.12982000	-0.26027800	-3.15306500
H	-3.52497500	-0.87643700	-3.32213400
C	-5.00934200	-2.42642500	-2.88488600
H	-5.85471600	-2.55373400	-2.20543700
H	-5.41375400	-2.50937400	-3.89553200
C	-3.97326700	-3.52913000	-2.66316300
H	-3.55160800	-3.49915000	-1.65715700
H	-4.42712000	-4.51118400	-2.79938900
H	-3.14905400	-3.44466000	-3.37385200
O	-3.98471900	3.08346700	-0.92541000
C	-4.81372800	4.23609200	-0.57651000
H	-5.22672100	4.06146100	0.41661900
H	-5.63727400	4.28328200	-1.28937200
C	-3.93296400	5.46007600	-0.62649800
H	-3.51377100	5.60337800	-1.62271300
H	-4.52820400	6.33978800	-0.37593000
H	-3.11554000	5.38502800	0.09050100

Complex I_C

C	-2.23362600	-0.63143800	1.42680100
C	-0.91175900	-0.97096100	1.75298800
C	-3.17192100	-0.78581700	2.45143000
C	-0.55023000	-1.44506500	3.00839600
C	-2.81926400	-1.26076300	3.71493900
C	-1.50589000	-1.59567900	4.00682100
C	-1.60340100	1.30562900	-0.18149400
C	-0.59757400	1.47775700	-1.11871100
C	-1.70322500	2.32673000	0.78474500
C	0.26403200	2.60957900	-1.09077300
C	-0.88251500	3.46980100	0.81425700
C	0.11332400	3.62604000	-0.11159700
C	-4.15718800	0.39661600	-0.23349400
C	-4.61457200	1.68830600	-0.49287500
C	-5.14502100	-0.60263500	-0.17316200
C	-5.97001700	1.98242400	-0.66270700
C	-6.49330000	-0.32101300	-0.33609200
C	-6.92159400	0.98217500	-0.58281100
C	-2.23681400	-1.08947900	-1.27932200
C	-2.46545200	-0.71730800	-2.61207800
C	-1.75334000	-2.38657300	-1.08243800
C	-2.19831400	-1.56726400	-3.68025300
C	-1.49895400	-3.25231000	-2.14584300
C	-1.70909000	-2.84835700	-3.45631500
B	-2.57839100	-0.02688600	-0.06964700
H	-4.20839000	-0.53209600	2.27172300
H	-0.13456900	-0.86433800	1.00496400
H	-1.23261700	-1.96359400	4.98401300

H	-1.57818600	-2.74675600	-0.07744800
H	-2.88280100	0.26017000	-2.82749800
H	-0.44561600	0.73413100	-1.88920600
H	-1.51103100	-3.51673700	-4.28046300
H	0.76947300	4.48331600	-0.09461900
H	-2.44838900	2.22279900	1.56560900
H	-4.85053900	-1.62961000	0.00199300
H	-3.91283600	2.50825200	-0.58453300
H	-7.97075900	1.20408200	-0.70984600
C	-6.36803300	3.40878600	-0.92555800
C	-7.50989300	-1.43251400	-0.30642000
C	-3.89376400	-1.38754200	4.76302300
C	0.89742300	-1.74191800	3.28591600
C	-1.10832600	4.50304800	1.89538100
C	1.27482700	2.72133700	-2.02463300
C	-2.41341100	-1.06302100	-5.08105700
C	-0.94547800	-4.62238600	-1.85585300
F	-1.04582200	-5.45453300	-2.91022300
F	-1.57270200	-5.21380900	-0.81936000
F	0.36925600	-4.56694100	-1.52800800
F	-7.09696400	-2.48663400	0.42196400
F	-7.77512300	-1.89744100	-1.55005000
F	-8.68818400	-1.02629700	0.21112200
F	-6.06828600	4.21373600	0.12183000
F	-7.68304900	3.54995100	-1.16539600
F	-5.71200200	3.92282900	-1.99354200
F	-2.32617000	-2.03499400	-6.00597100
F	-3.61555200	-0.47160600	-5.23222100
F	-1.48555600	-0.12443700	-5.41375100
F	1.48624900	1.86873300	-2.95432600

F	3.14497400	1.49152900	-0.67087300
F	2.07418100	3.72265700	-2.09641000
F	1.07958600	-2.45321700	4.41143700
F	1.47817300	-2.43081700	2.27846900
F	1.62273400	-0.59964500	3.42488100
F	-3.43195000	-1.90049500	5.91931900
F	-4.44434400	-0.18664400	5.05671500
F	-4.90455400	-2.18171600	4.35172800
F	-0.98737200	3.95755900	3.11798400
F	-2.34255500	5.02797200	1.80955100
F	-0.22747400	5.51599900	1.81176300
Si	4.52363900	0.73528000	-0.16144700
H	5.57622200	1.34656500	-1.00934300
C	4.31698200	-1.08470800	-0.50361800
C	4.08637400	-1.45389000	-1.97686200
H	3.49744700	-1.46410600	0.11579900
H	5.22482100	-1.56928000	-0.13011200
H	3.98869600	-2.53368100	-2.10202100
H	3.17403000	-0.99965200	-2.36922700
H	4.91555800	-1.12367400	-2.60643400
C	4.74623500	1.16512300	1.63998500
C	4.85207300	2.66643400	1.94627200
H	5.65357700	0.64776700	1.96770000
H	3.92286400	0.71904800	2.20732800
H	5.00091800	2.84331100	3.01330500
H	5.69065700	3.12537900	1.41763000
H	3.94662100	3.20164500	1.65202500
C	8.83174300	-1.03689900	0.79112400
O	7.64492000	-0.80465400	0.73291500
C	9.82512700	0.12576100	0.85605400

H	10.65840300	-0.12534800	1.51834200
H	9.31921800	1.00545700	1.24645500
C	10.42823400	0.43957400	-0.50384300
O	10.75713300	-0.38473100	-1.31923100
C	9.41045300	-2.43135500	0.77898800
H	10.04436100	-2.47322000	-0.11463700
H	10.10711200	-2.51272400	1.62240500
C	8.39160500	-3.56796500	0.79822700
H	7.63466700	-3.37956400	0.03374000
H	8.90363200	-4.48858700	0.50703900
C	7.71765200	-3.77026300	2.15508300
H	7.16808400	-2.88012600	2.46333000
H	7.01054500	-4.60070200	2.11966800
H	8.45489300	-3.99857300	2.92952400
O	10.56893300	1.76131200	-0.67123200
C	11.17602300	2.19522300	-1.91612600
H	10.56974500	1.82280700	-2.74253600
H	12.16430300	1.74058500	-1.99492000
C	11.24262400	3.70430300	-1.89172600
H	11.84708400	4.05738500	-1.05536200
H	11.69565100	4.06436500	-2.81708500
H	10.24620600	4.13958100	-1.80672400

9.3. DFT calculations of Mulliken charge on silicon atom

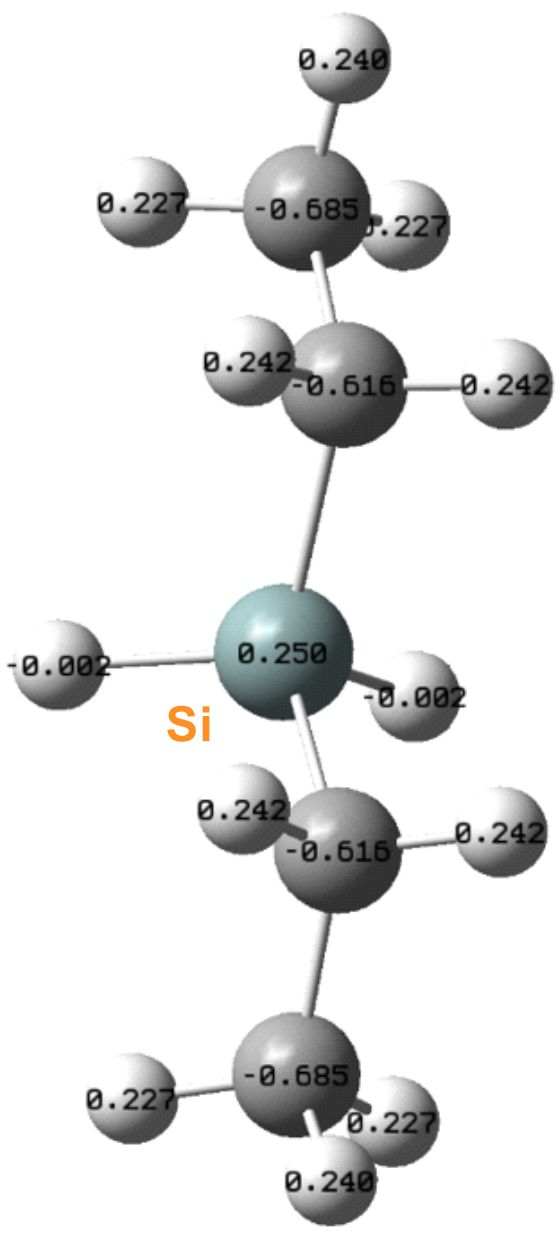

[Et_2SiH_2]
Mulliken charge (Si) = **0.250**

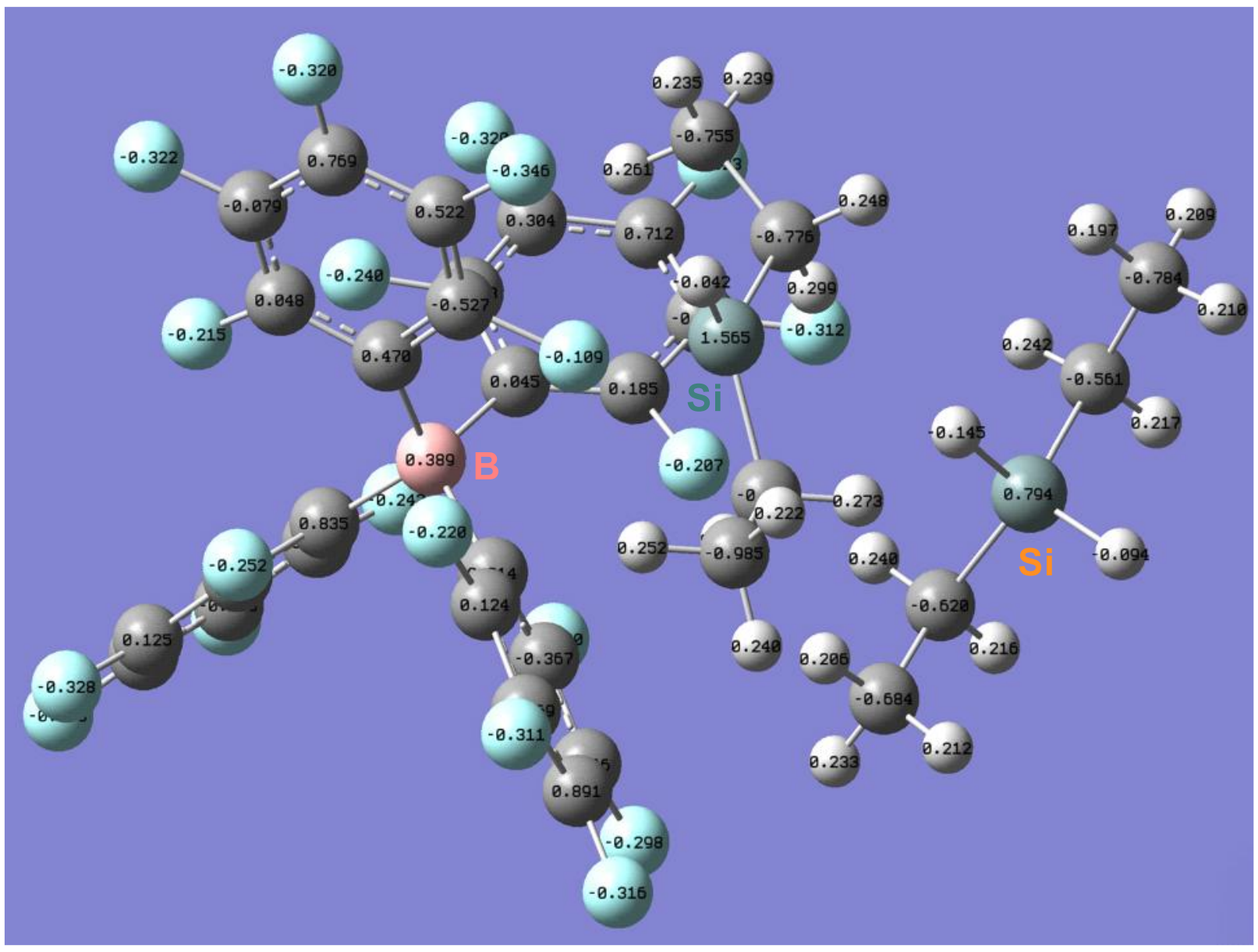

[$Et_2HSi]^+[B(C_6F_5)_4]^-$ with [Et_2SiH_2]
Mulliken charge (Si) = **0.794**

9.4. Computational study of ketimine-enamine type tautomerization

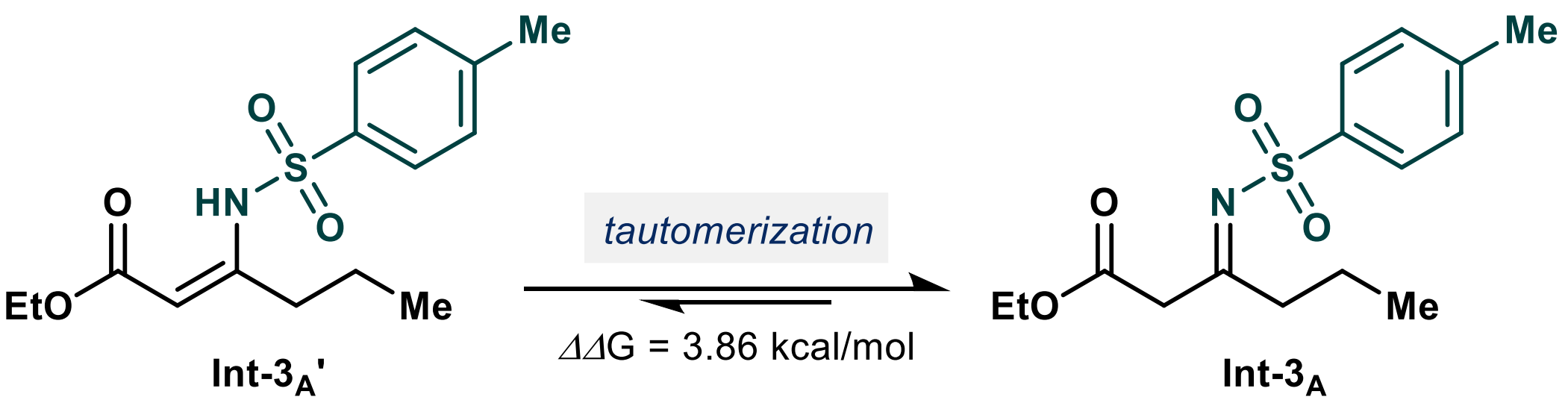

**Int-3$_A$'**	**Int-3$_A$**
**[enamine]**	**[ketimine]**
**DFT energy: -1337.216945 Ha**	**DFT energy: -1337.223094 Ha**

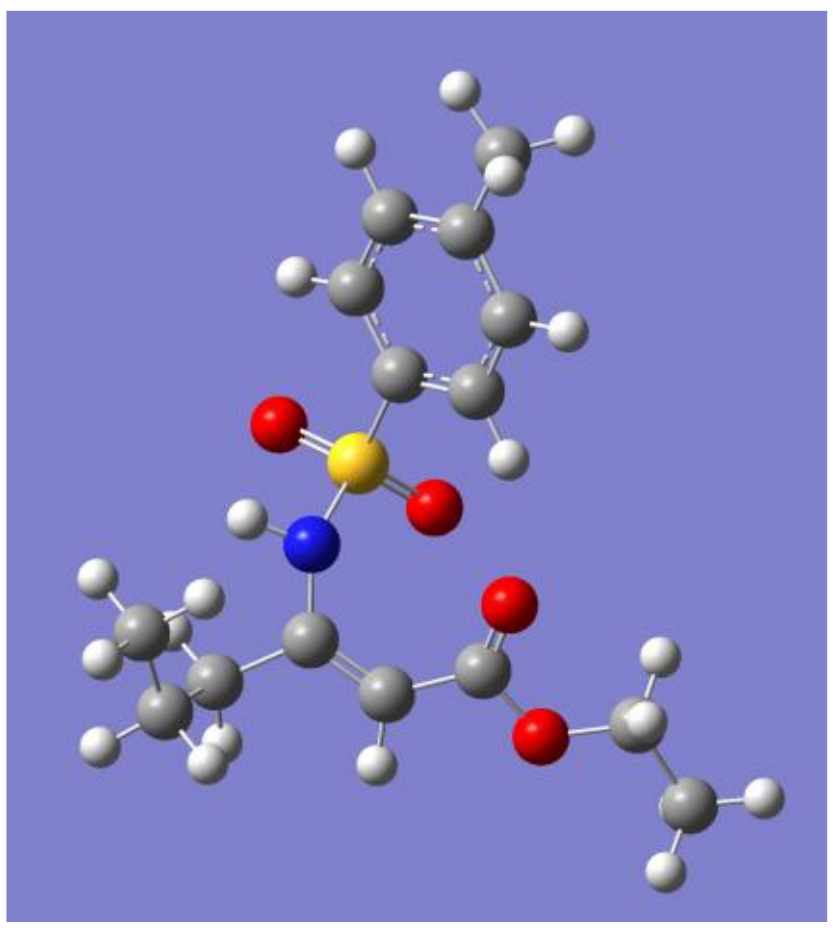

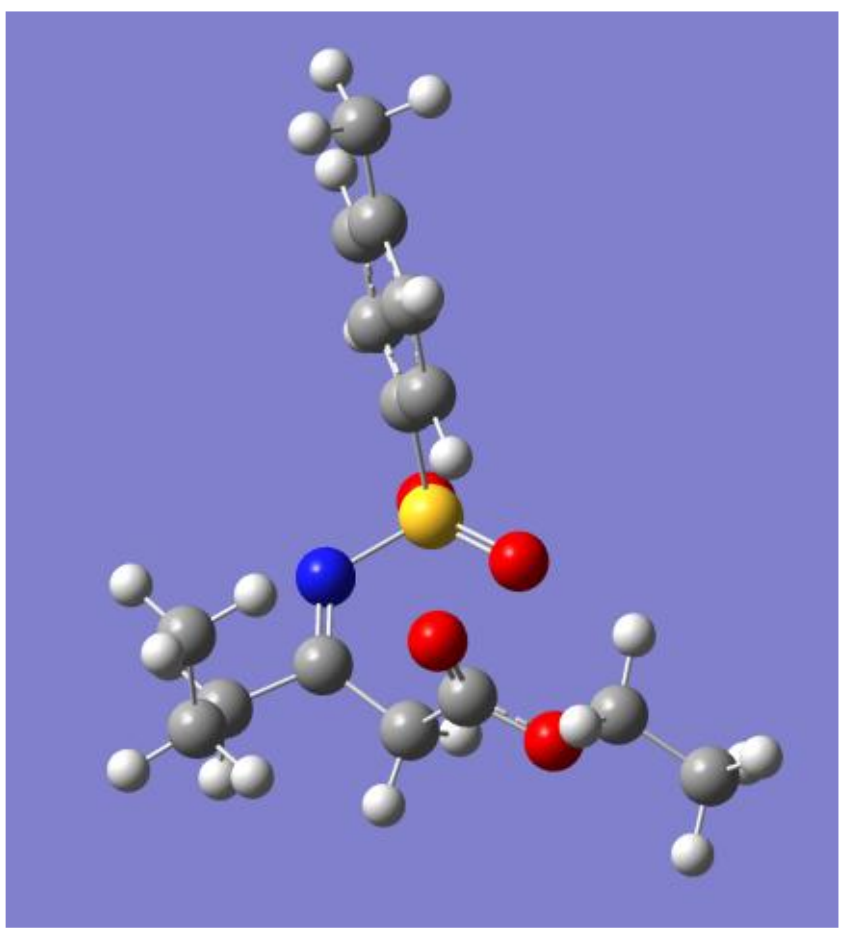

10. HR-MS analytical data for products

No.	Sample name	Chemical Formula	Exact Mass (M)	Calcd for [M+Na]+	found Mass [M+Na]+
1	a1	C15H23NO4S	313.1348	336.1240	336.1242

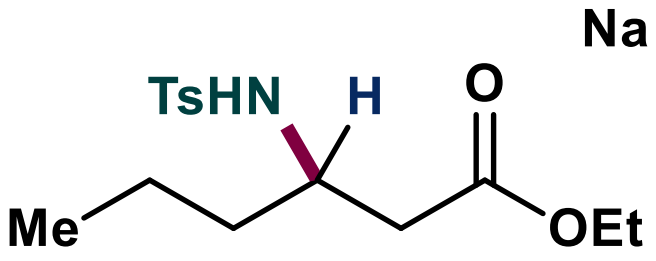

1

Exact Mass: 336.1245

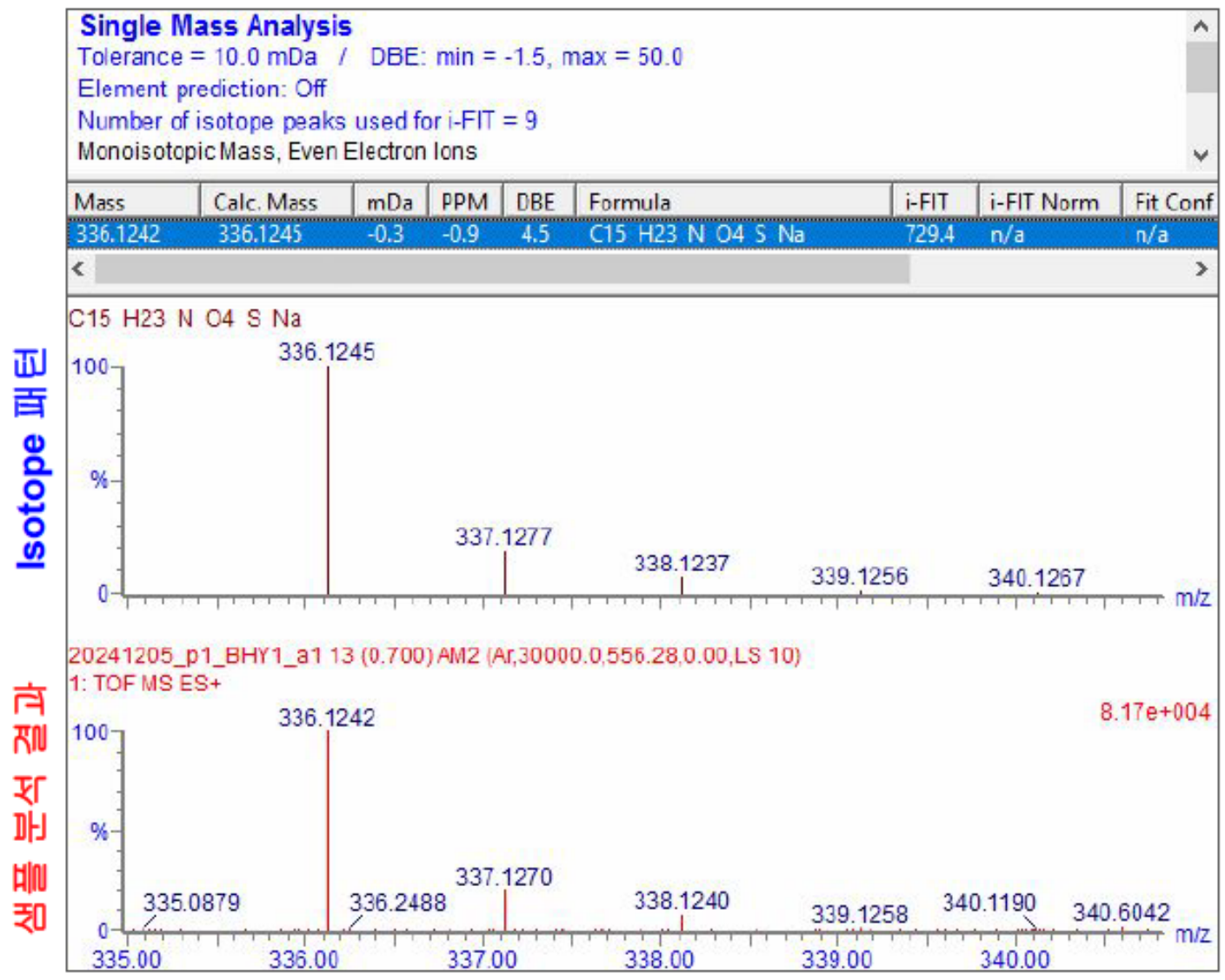

High-resolution mass spectra (**1**)

No.	Sample name	Chemical Formula	Exact Mass (M)	Calcd for [M+Na]+	found Mass [M+Na]+
5	a8	C14H21NO4S	299.1191	322.1083	322.1090

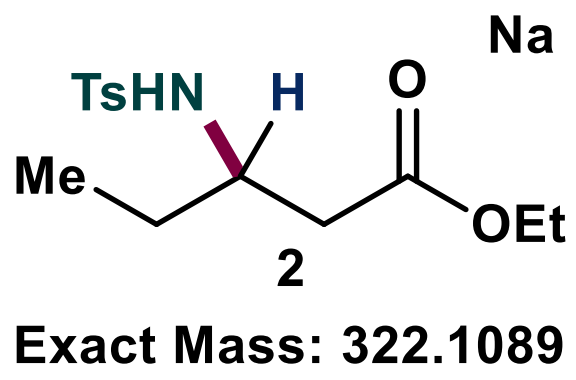

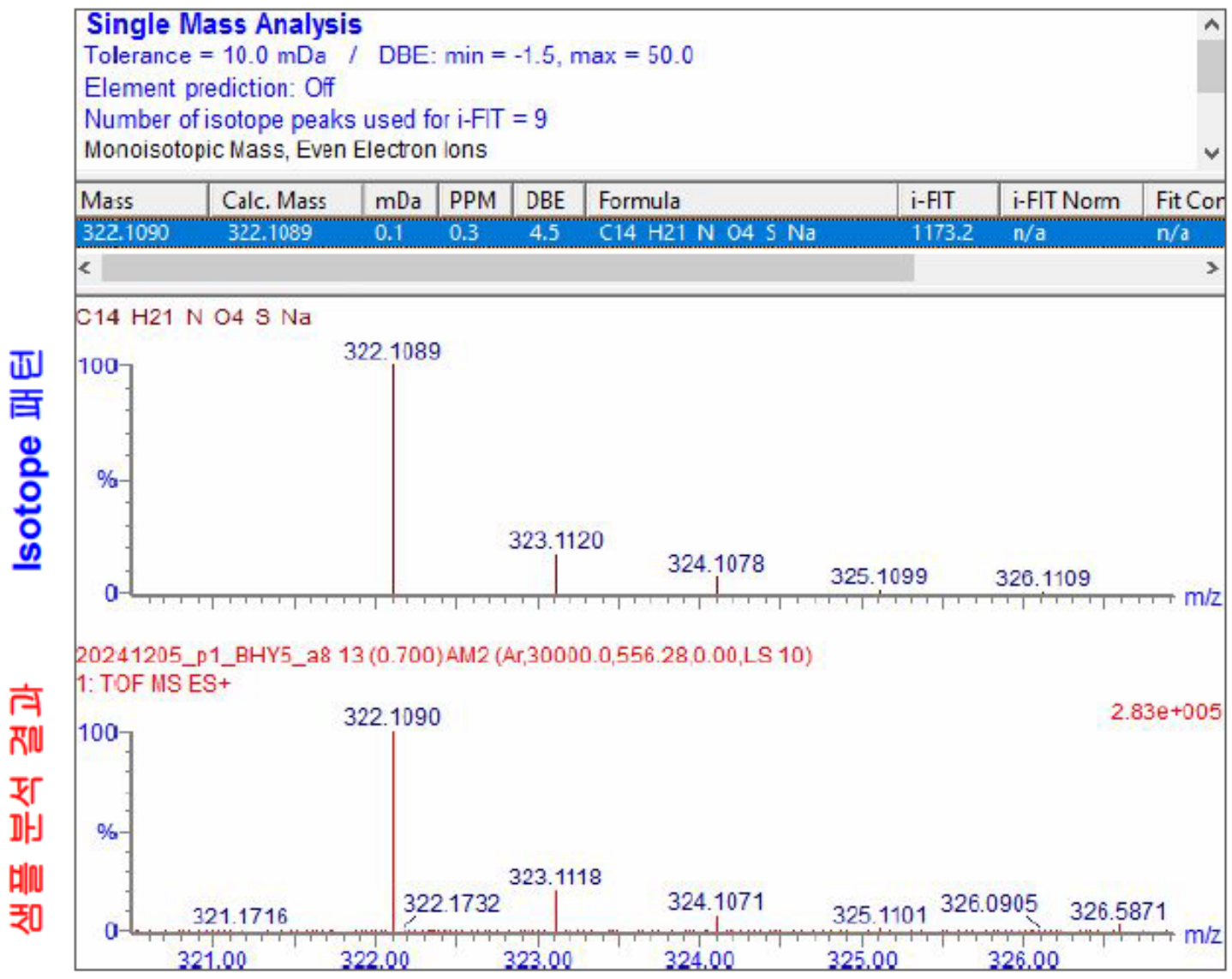

High-resolution mass spectra (**2**)

No.	Sample name	Chemical Formula	Exact Mass (M)	Calcd for [M+Na]+	found Mass [M+Na]+
6	a9	C15H23NO4S	313.1348	336.1240	336.1244

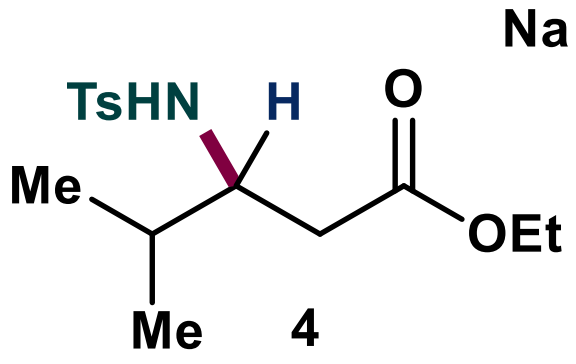

4

Exact Mass: 336.1245

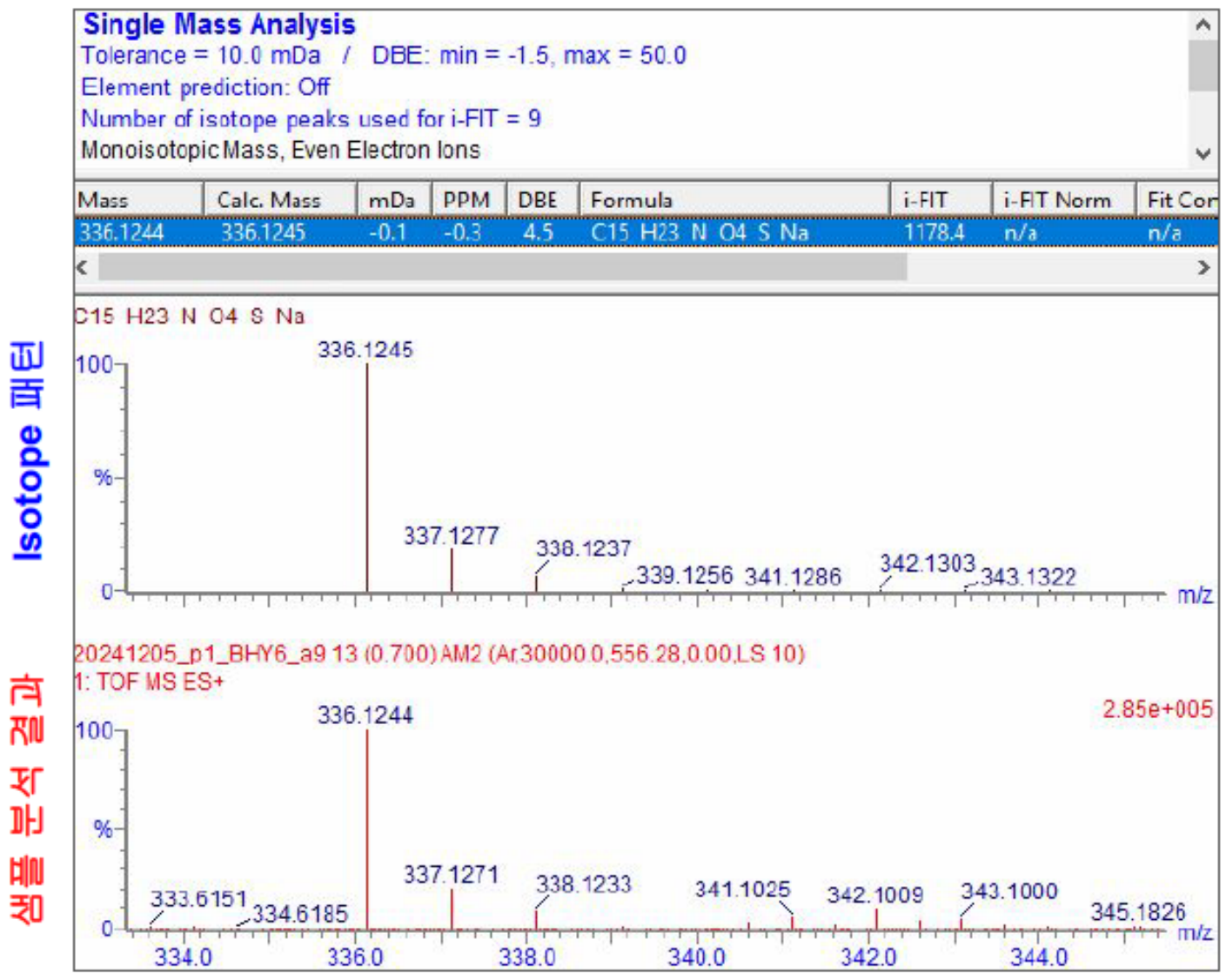

High-resolution mass spectra (**4**)

No.	Sample name	Chemical Formula	Exact Mass (M)	Calcd for [M+Na]+	found Mass [M+Na]+
3	a5	C15H23NO4S	313.1348	336.1240	336.1240

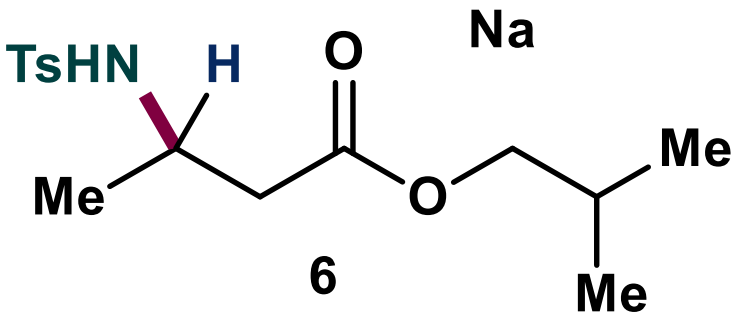

6

Exact Mass: 336.1245

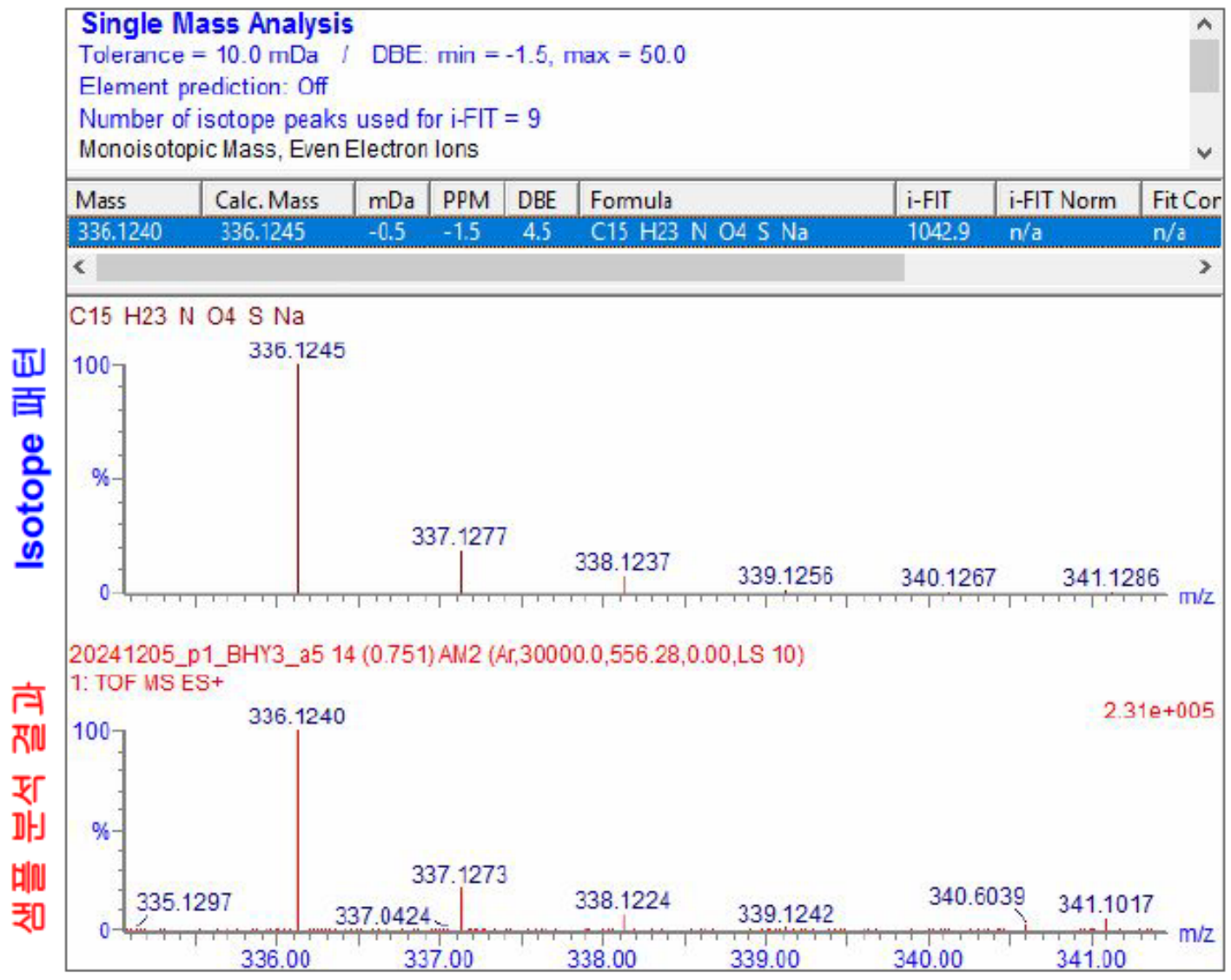

High-resolution mass spectra (**6**)

No.	Sample name	Chemical Formula	Exact Mass (M)	Calcd for [M+Na]+	found Mass [M+Na]+
8	a11	C18H21NO4S	347.1191	370.1083	370.1085

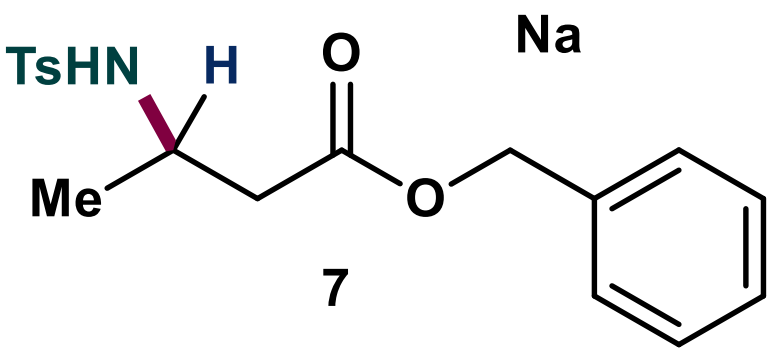

7

Exact Mass: 370.1089

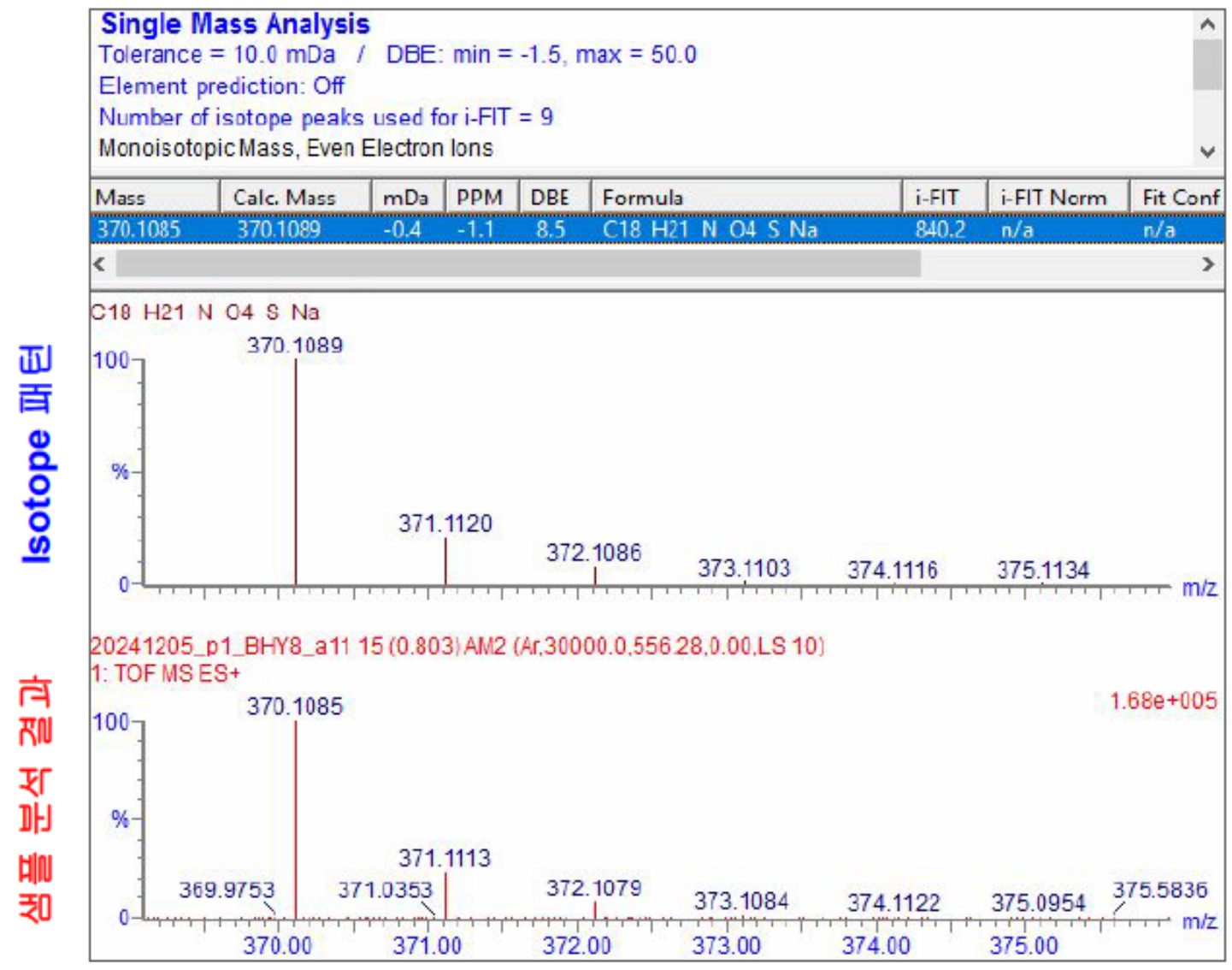

High-resolution mass spectra (**7**)

No.	Sample name	Chemical Formula	Exact Mass (M)	Calcd for [M+Na]+	found Mass [M+Na]+
2	a4	C12H16ClNO4S	305.0489	328.0381	328.0385

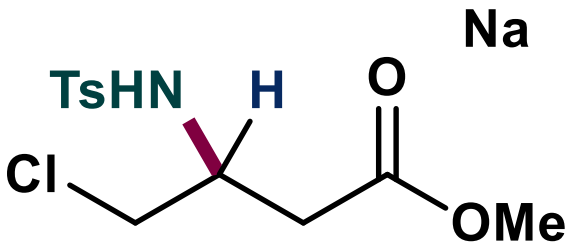

8

Exact Mass: 328.0386

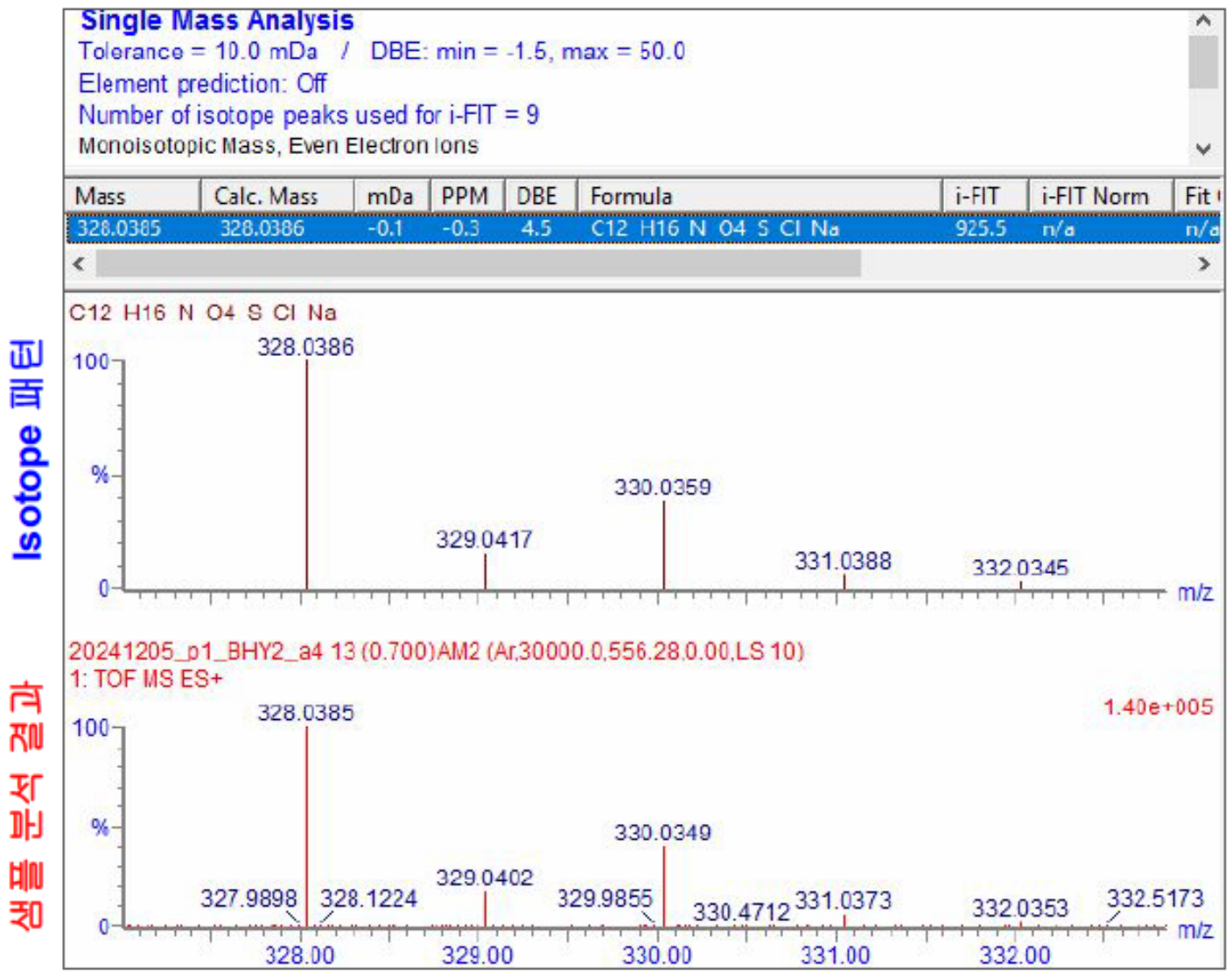

High-resolution mass spectra (**8**)

No.	Sample name	Chemical Formula	Exact Mass (M)	Calcd for [M+Na]+	found Mass [M+Na]+
7	a10	C12H16ClNO4S	305.0489	328.0381	328.0385

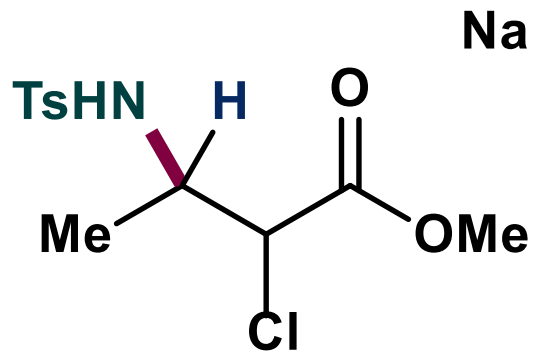

9

Exact Mass: 328.0386

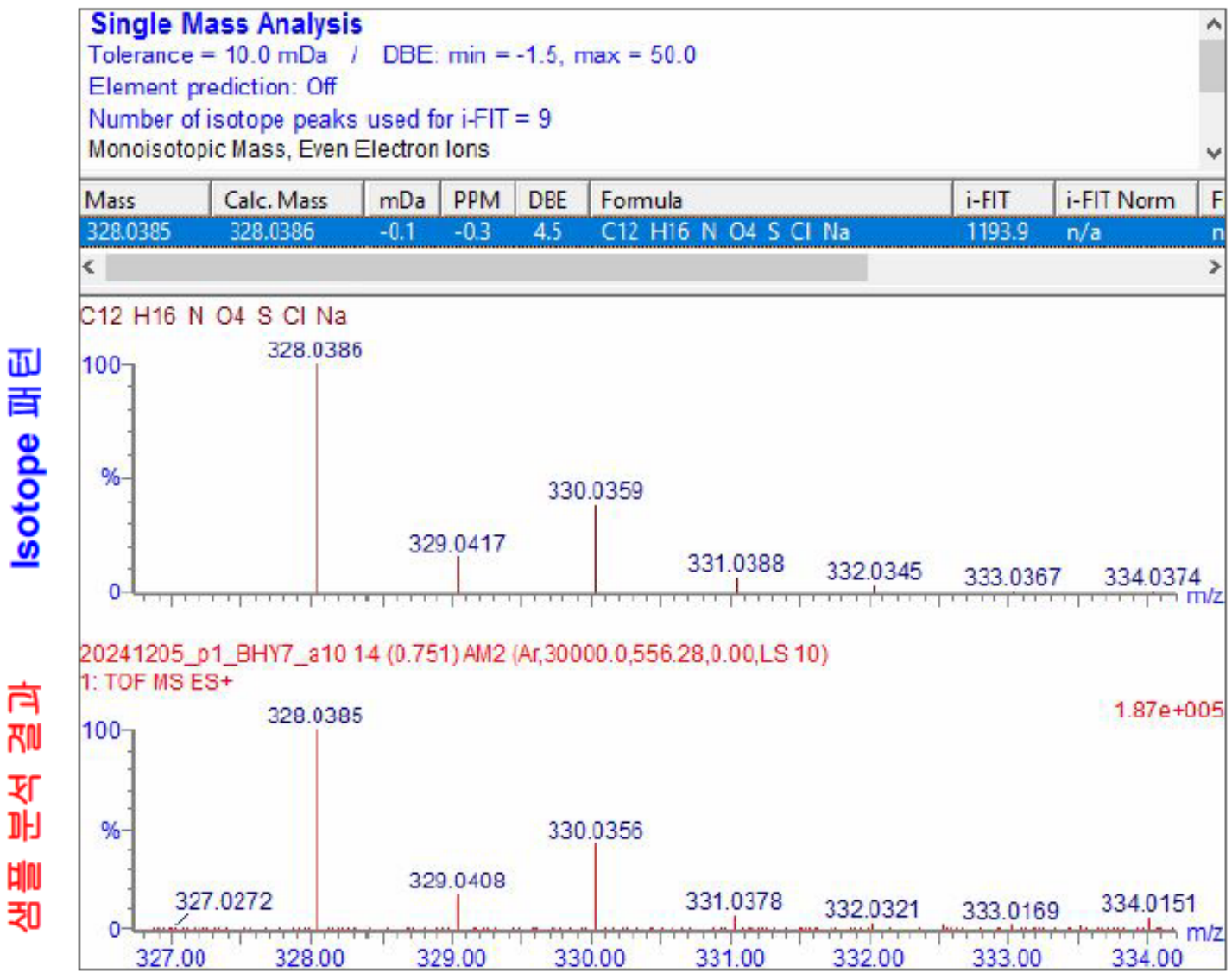

High-resolution mass spectra (**9**)

No.	Sample name	Chemical Formula	Exact Mass (M)	Calcd for [M+Na]+	found Mass [M+Na]+
4	a6	C16H23NO4S	325.1348	348.1240	348.1237

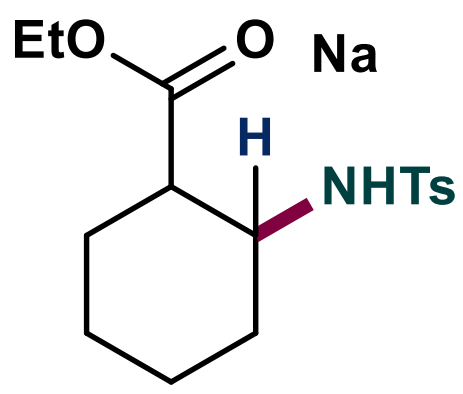

11

Exact Mass: 348.1245

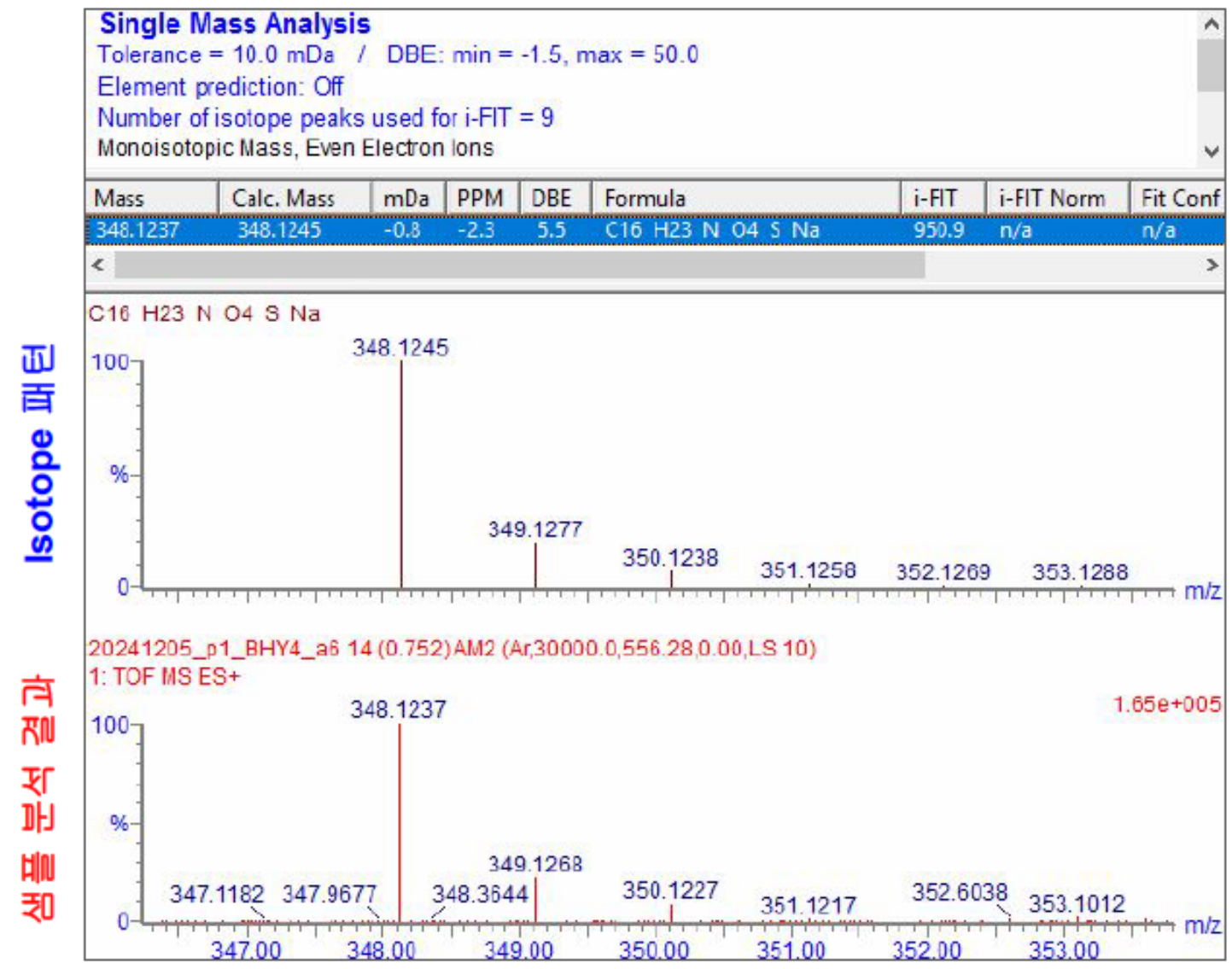

High-resolution mass spectra (**11**)

No.	Sample name	Chemical Formula	Exact Mass (M)	Calcd for [M+Na]+	found Mass [M+Na]+
9	a12	C18H18F3NO4S	401.0909	424.0801	424.0801

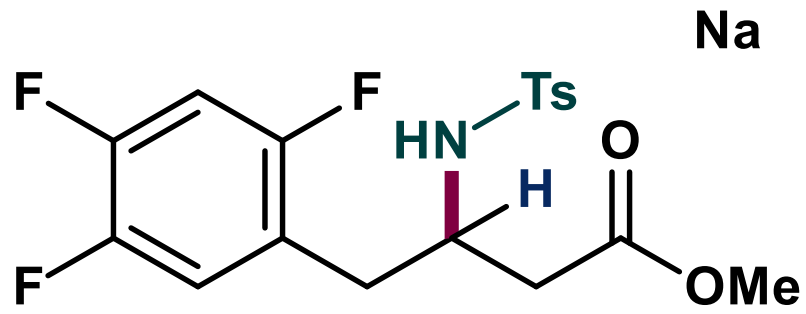

12

Exact Mass: 424.0806

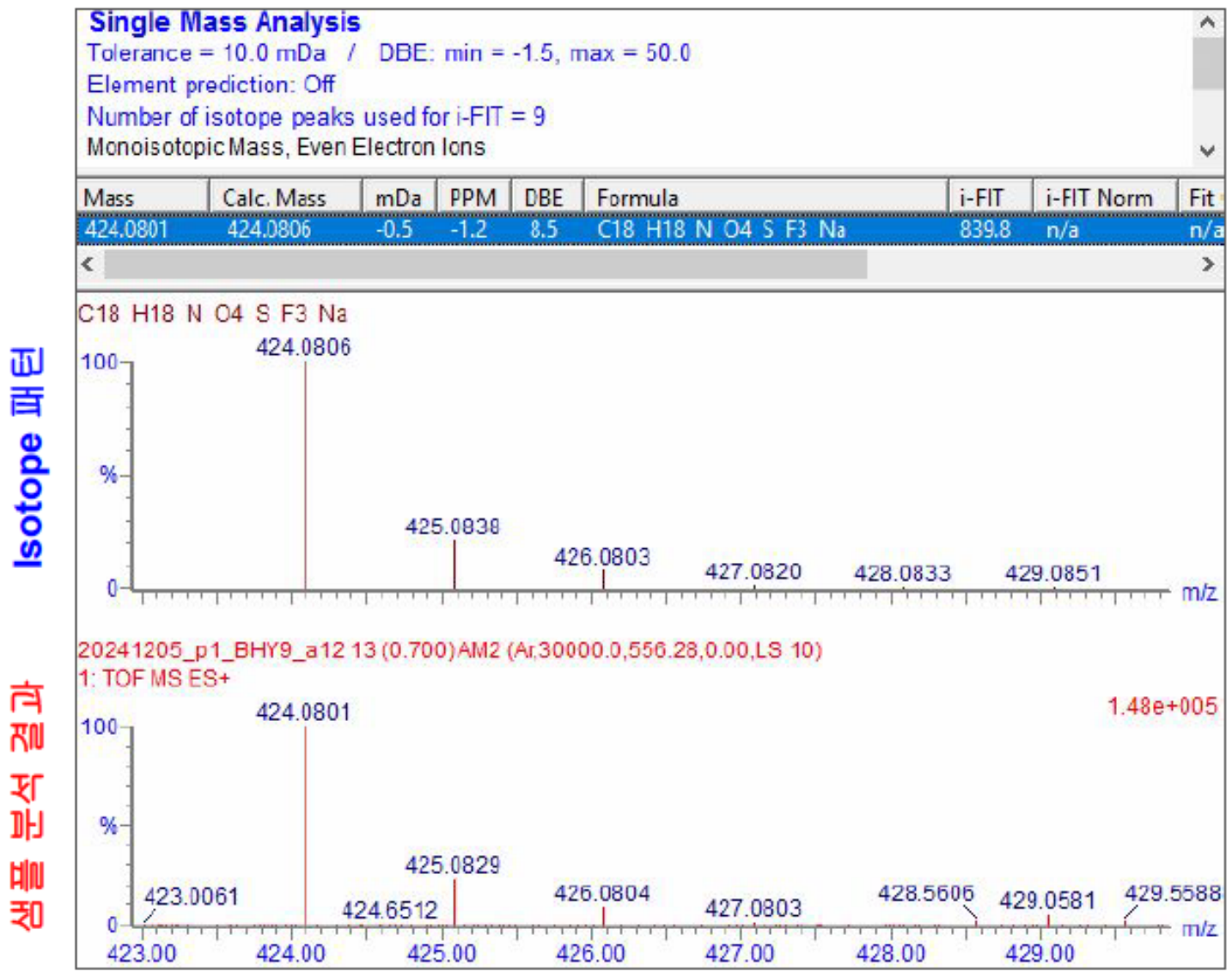

High-resolution mass spectra (**12**)

No.	Sample name	Chemical Formula	Exact Mass (M)	Calcd for [M+Na]+	found Mass [M+Na]+
10	a14	C17H18FNO4S	351.0941	374.0833	374.0835

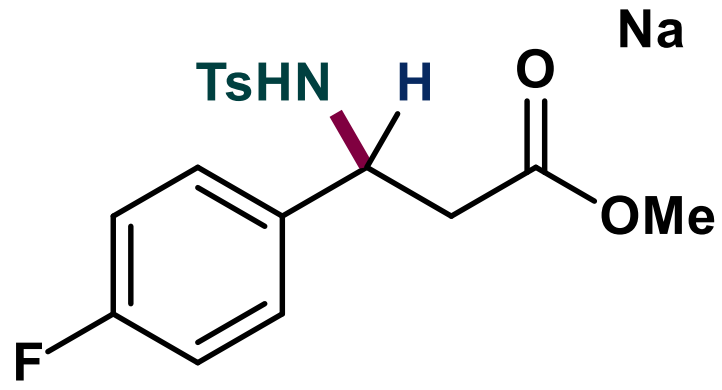

14

Exact Mass: 374.0838

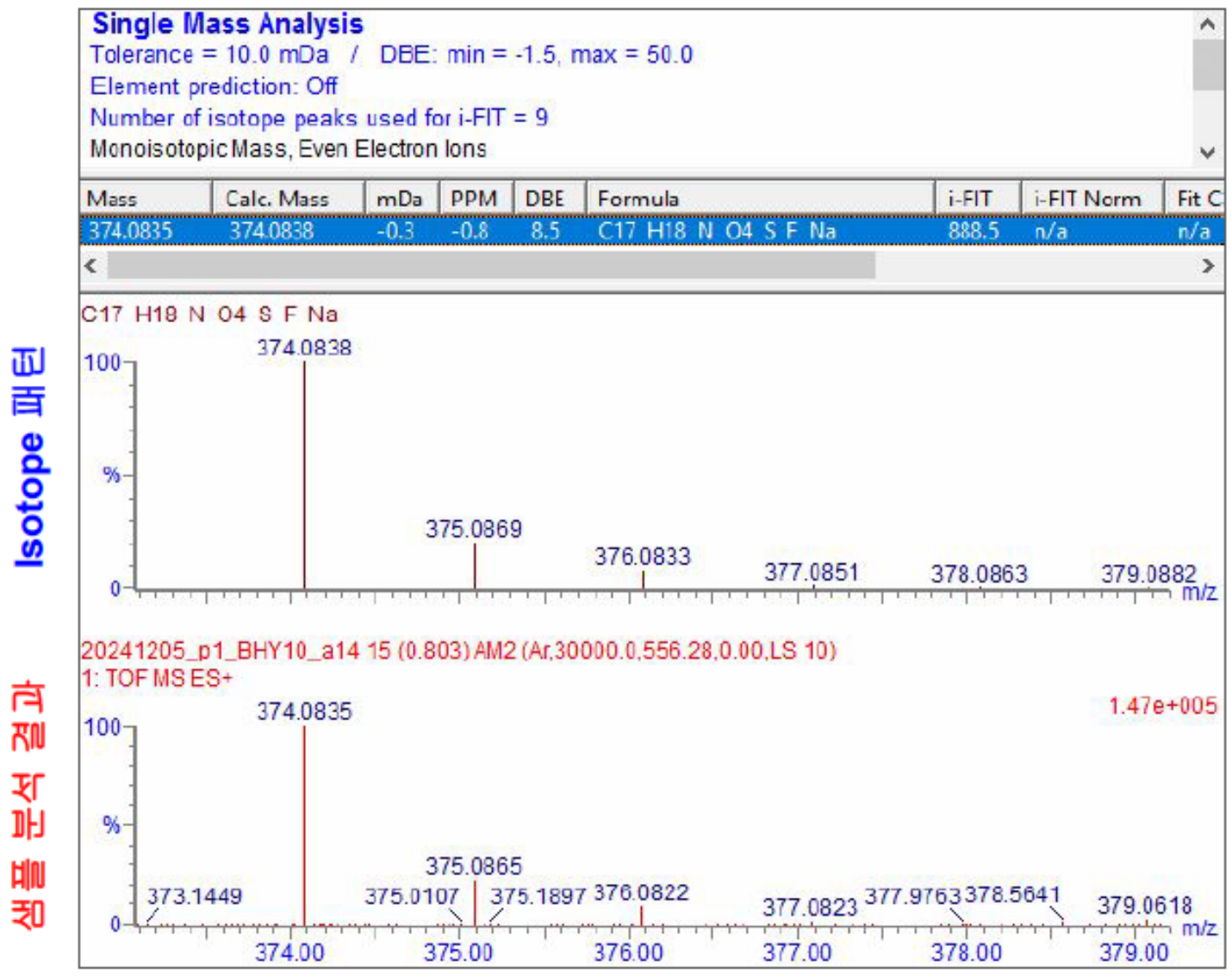

High-resolution mass spectra (**14**)

No.	Sample name	Chemical Formula	Exact Mass (M)	Calcd for [M+Na]+	found Mass [M+Na]+
11	a16	C16H24ClNO4S	361.1115	384.1007	384.1008

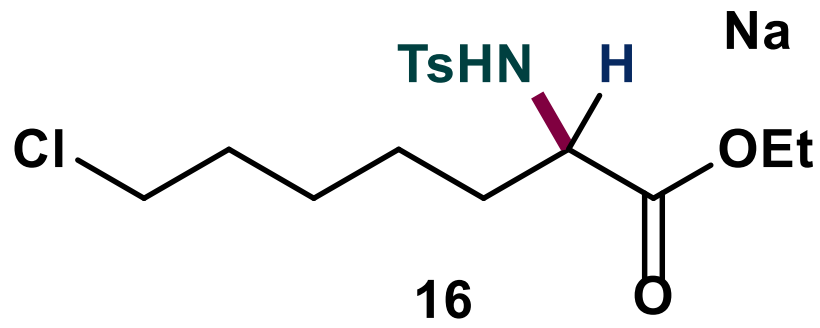

16

Exact Mass: 384.1012

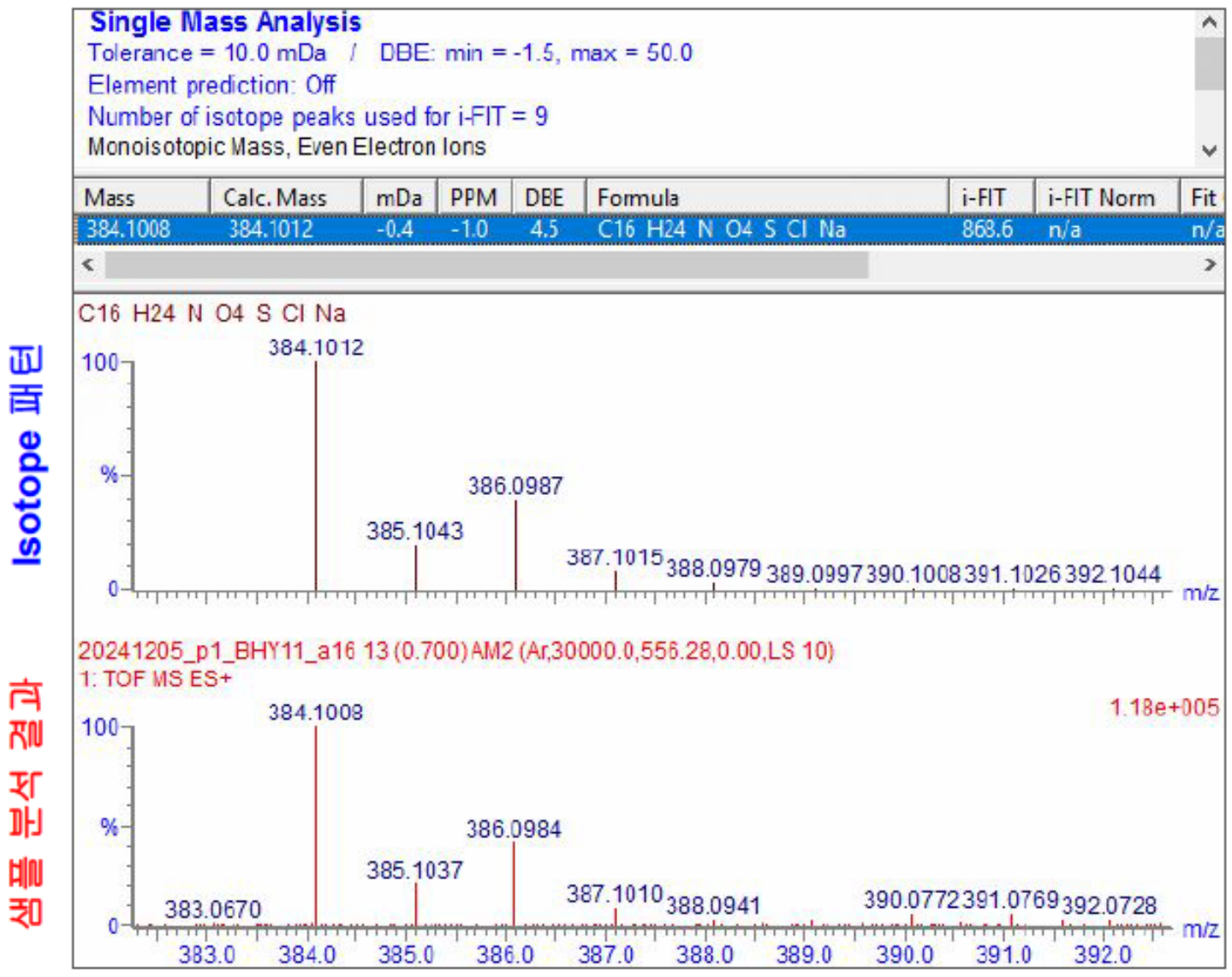

High-resolution mass spectra (**16**)

No.	Sample name	Chemical Formula	Exact Mass (M)	Calcd for [M+Na]+	found Mass [M+Na]+
14	b6	C17H29NO2S	311.1919	334.1811	334.1812

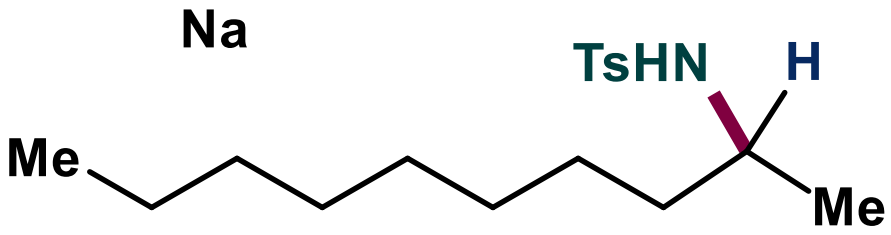

21

Exact Mass: 334.1817

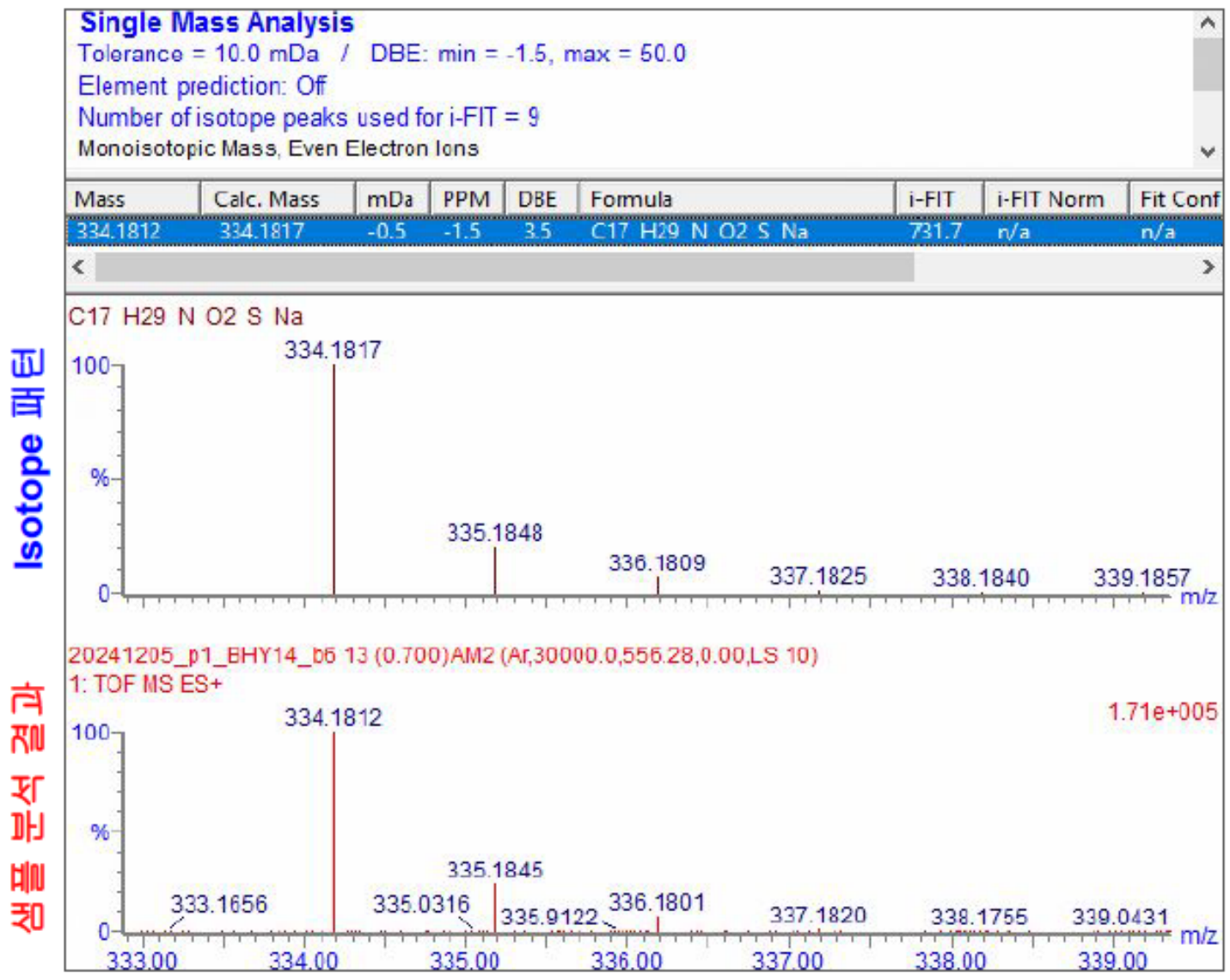

High-resolution mass spectra (**21**)

No.	Sample name	Chemical Formula	Exact Mass (M)	Calcd for [M+Na]+	found Mass [M+Na]+
12	b3	C14H23NO2S	269.1449	292.1341	292.1346

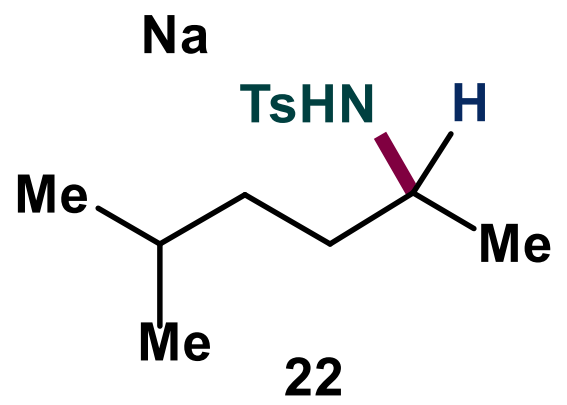

22

Exact Mass: 292.1347

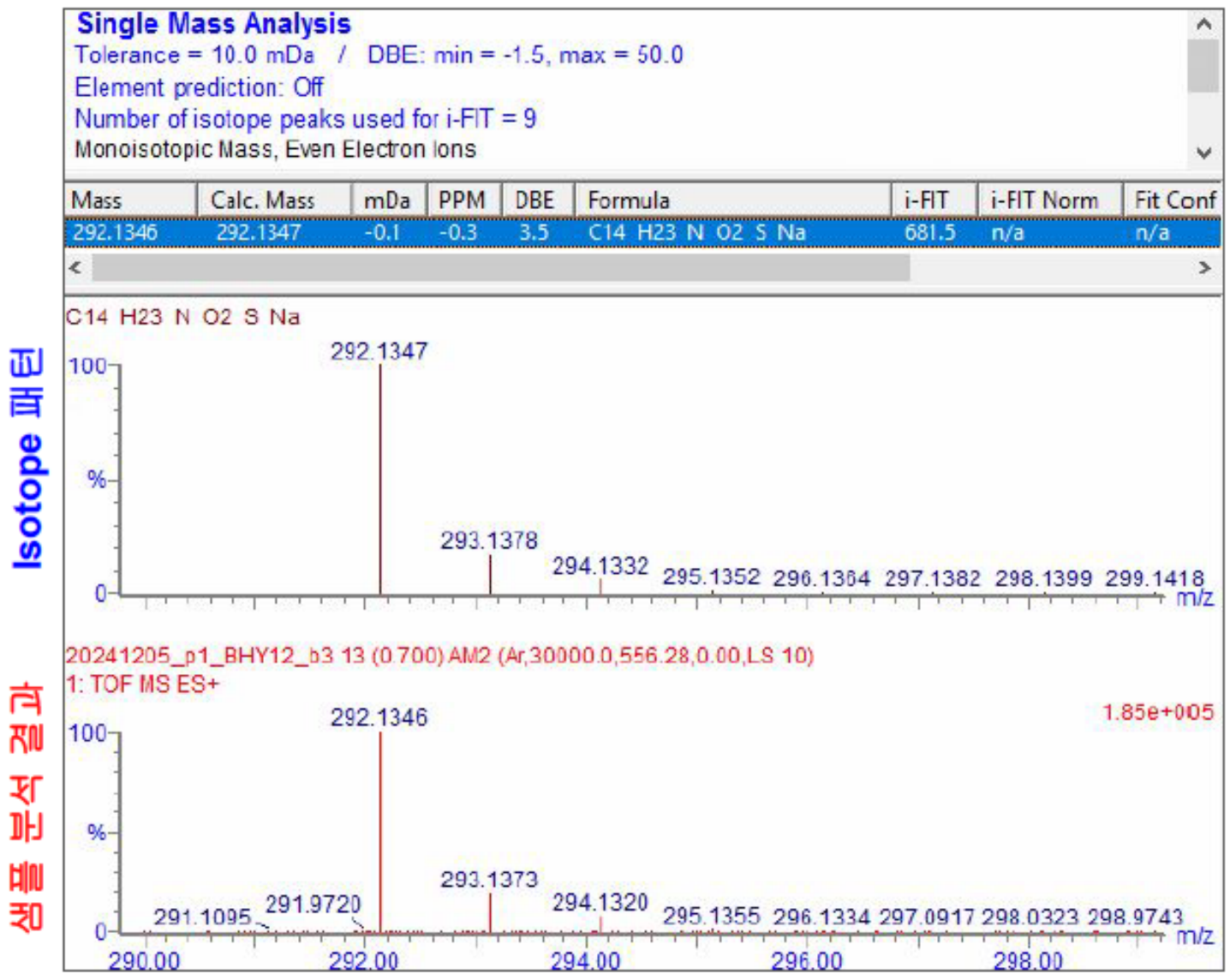

High-resolution mass spectra (**22**)

No.	Sample name	Chemical Formula	Exact Mass (M)	Calcd for [M+Na]+	found Mass [M+Na]+
13	b5	C12H18ClNO2S	275.0747	298.0639	298.0645

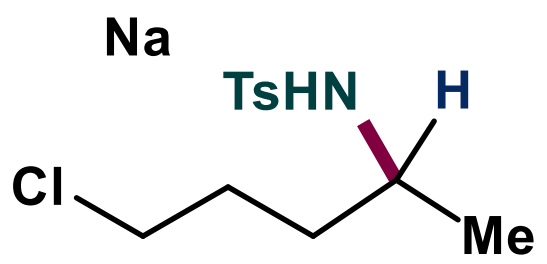

23

Exact Mass: 298.0644

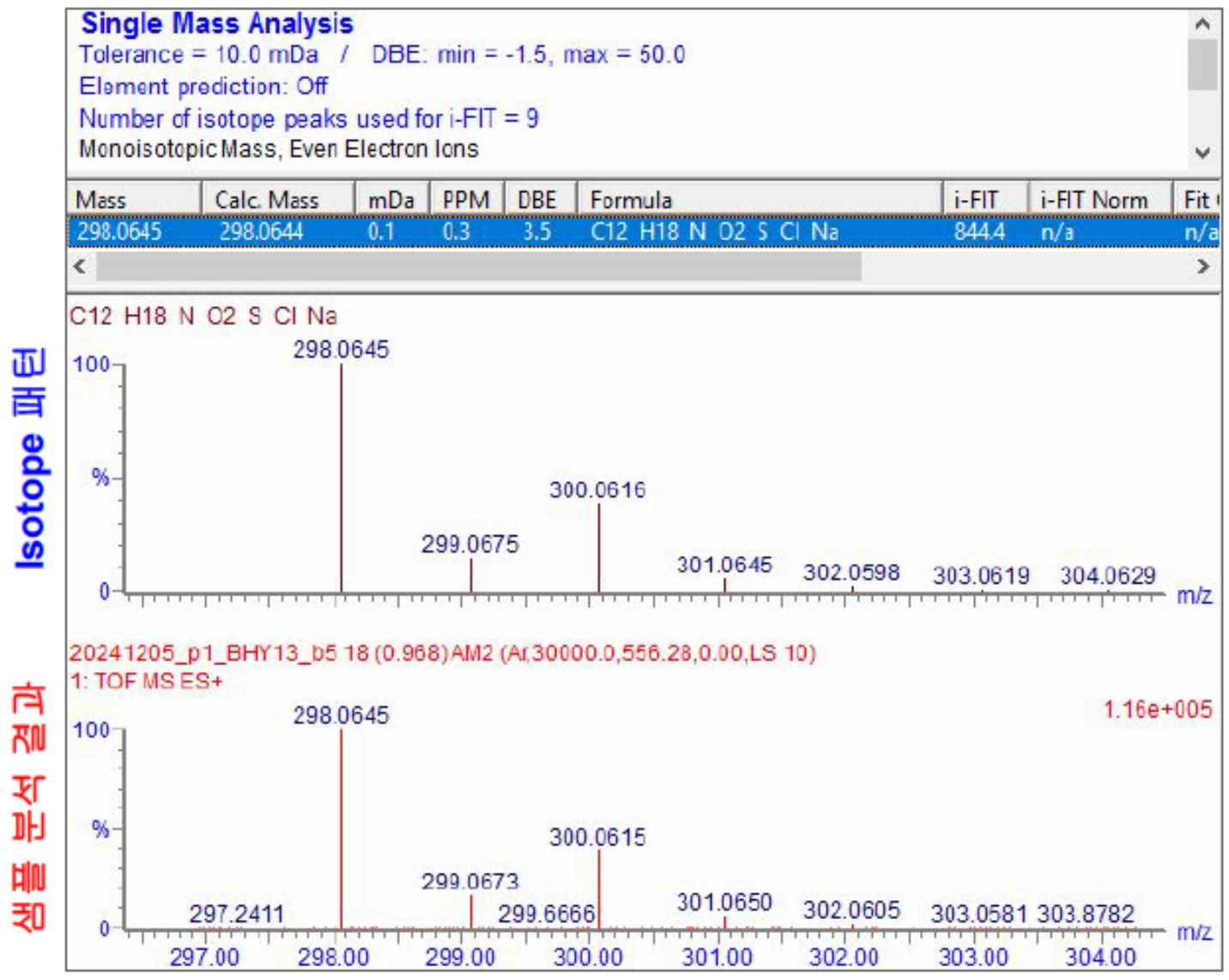

High-resolution mass spectra (**23**)

No.	Sample name	Chemical Formula	Exact Mass (M)	Calcd for [M+Na]+	found Mass [M+Na]+
15	b16	C15H16ClNO2S	309.0590	332.0482	332.0480

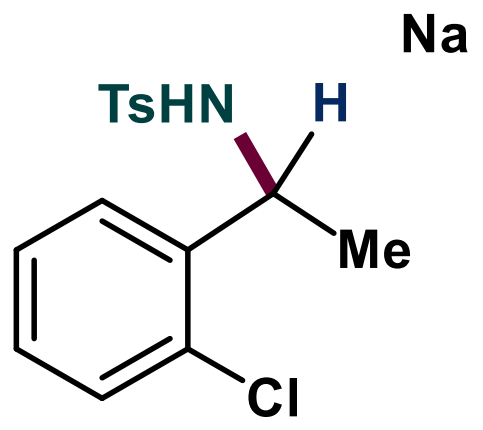

35

Exact Mass: 332.0488

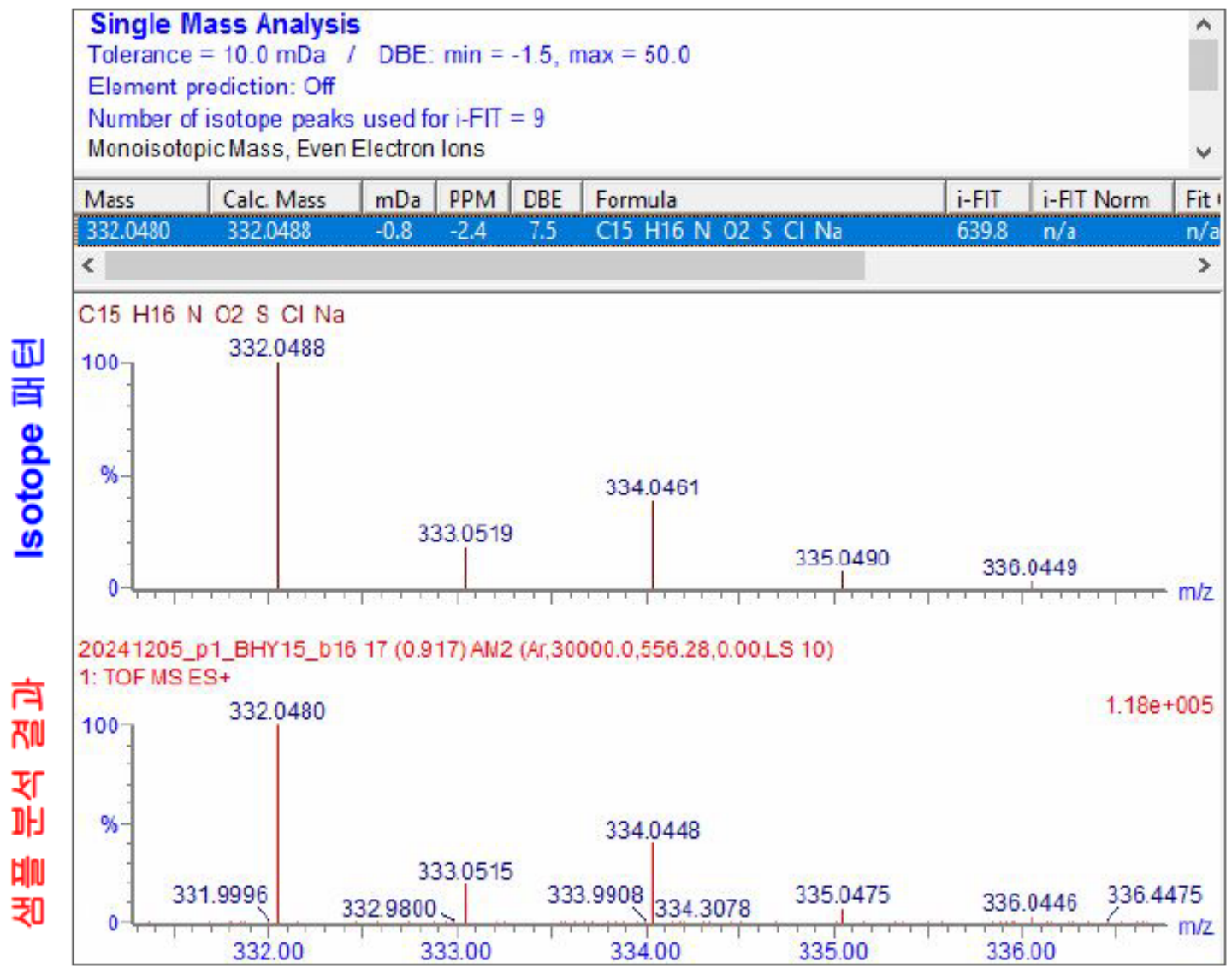

High-resolution mass spectra (**35**)

No.	Sample name	Chemical Formula	Exact Mass (M)	Calcd for [M+Na]+	found Mass [M+Na]+
16	b19	C15H15F2NO2S	311.0792	334.0684	334.0688

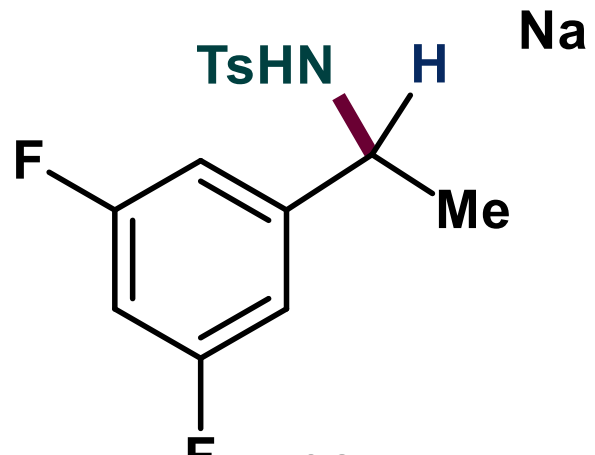

38

Exact Mass: 334.0689

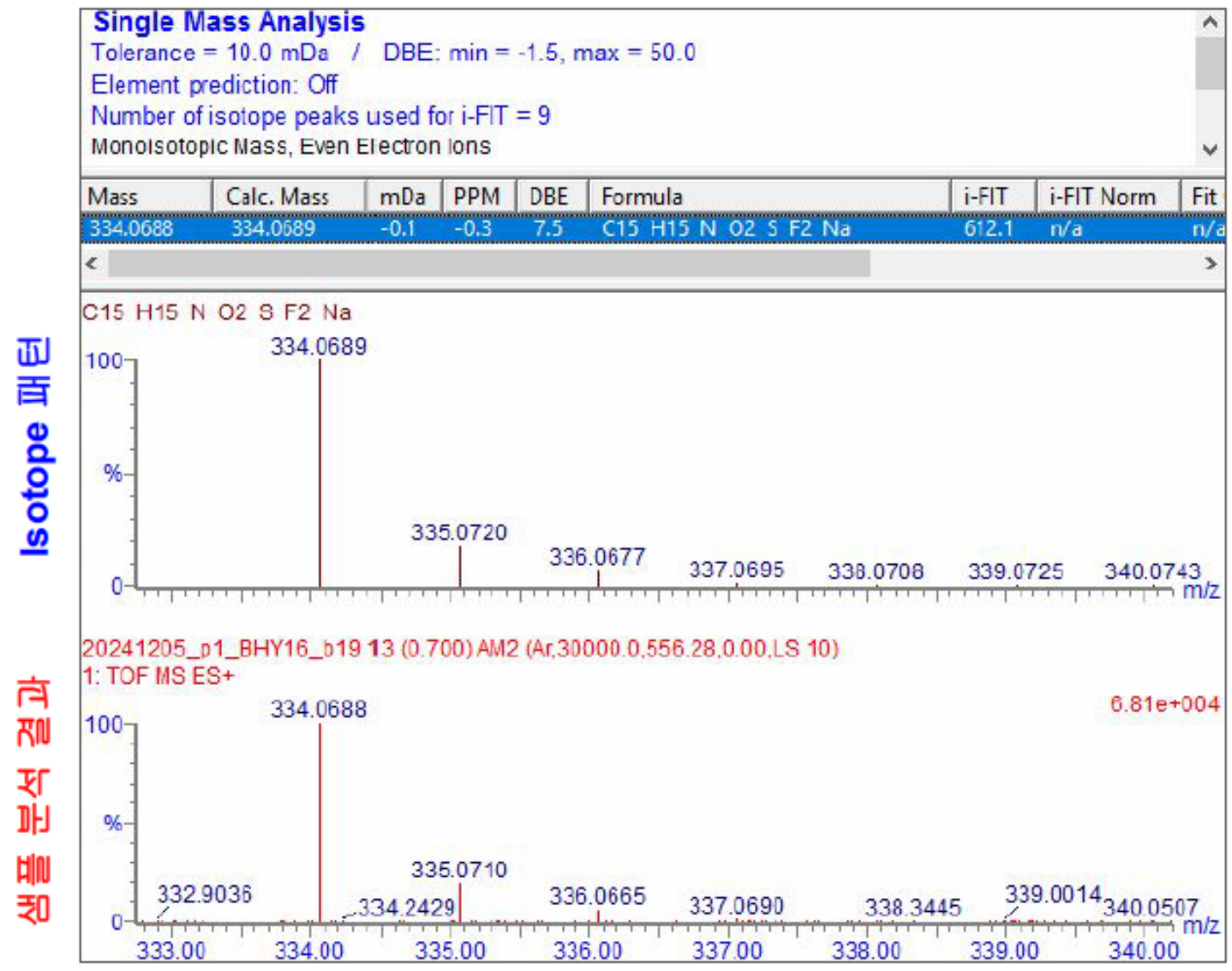

High-resolution mass spectra (**38**)

No.	Sample name	Chemical Formula	Exact Mass (M)	Calcd for [M+Na]+	found Mass [M+Na]+
17	b28	C16H16ClNO3S	337.0539	360.0431	360.0432

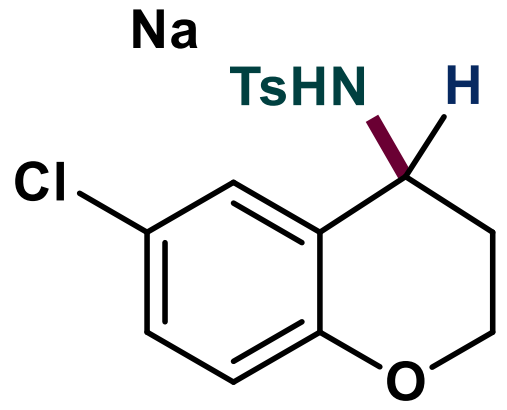

46

Exact Mass: 360.0437

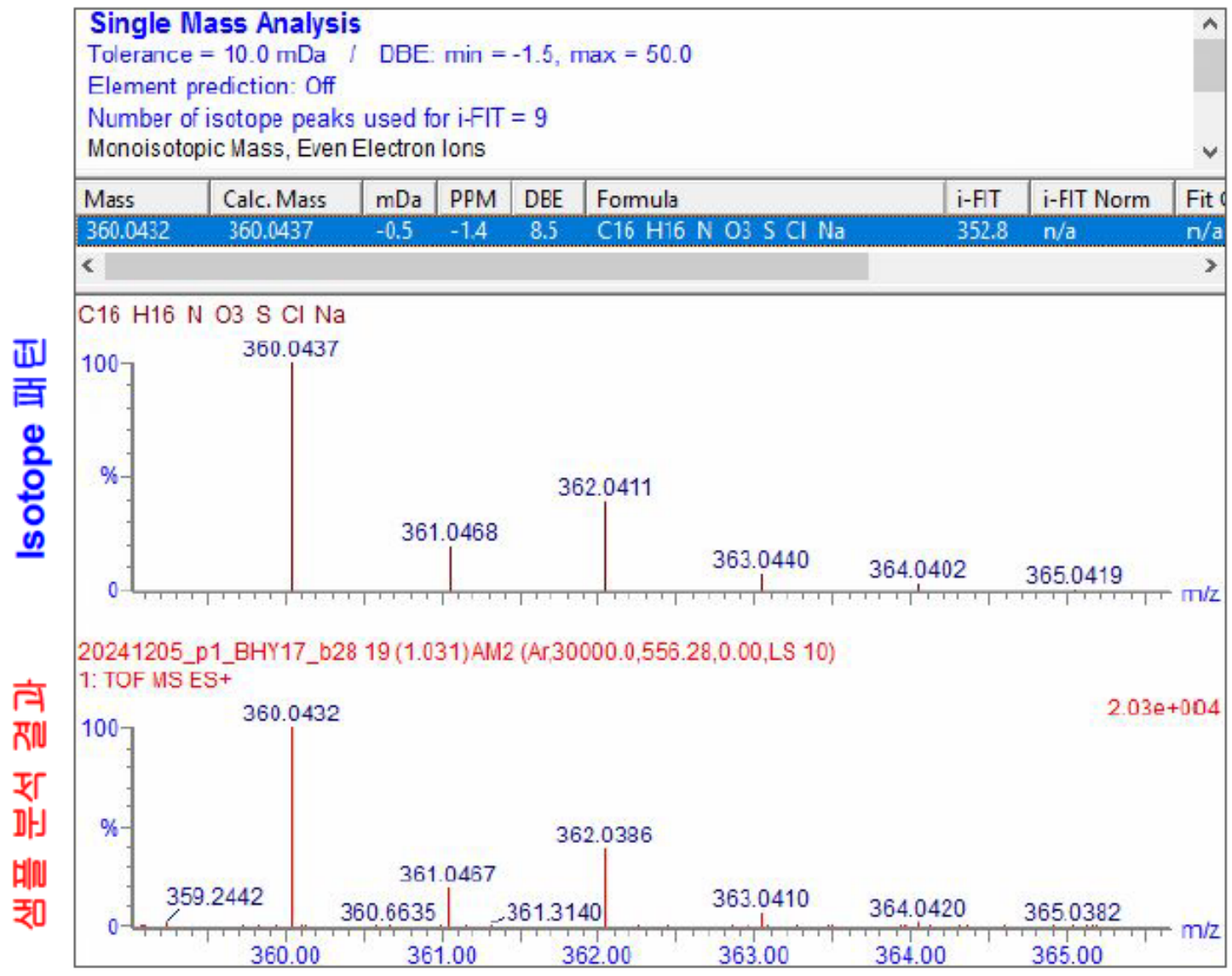

High-resolution mass spectra (**46**)

No.	Sample name	Chemical Formula	Exact Mass (M)	Calcd for [M+Na]+	found Mass [M+Na]+
32	G1	C22H25NO3S	383.1555	406.1447	406.1445

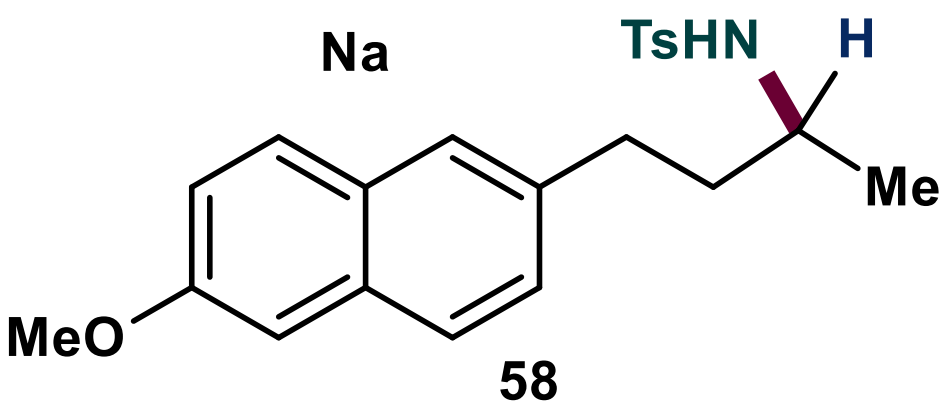

58

Exact Mass: 406.1453

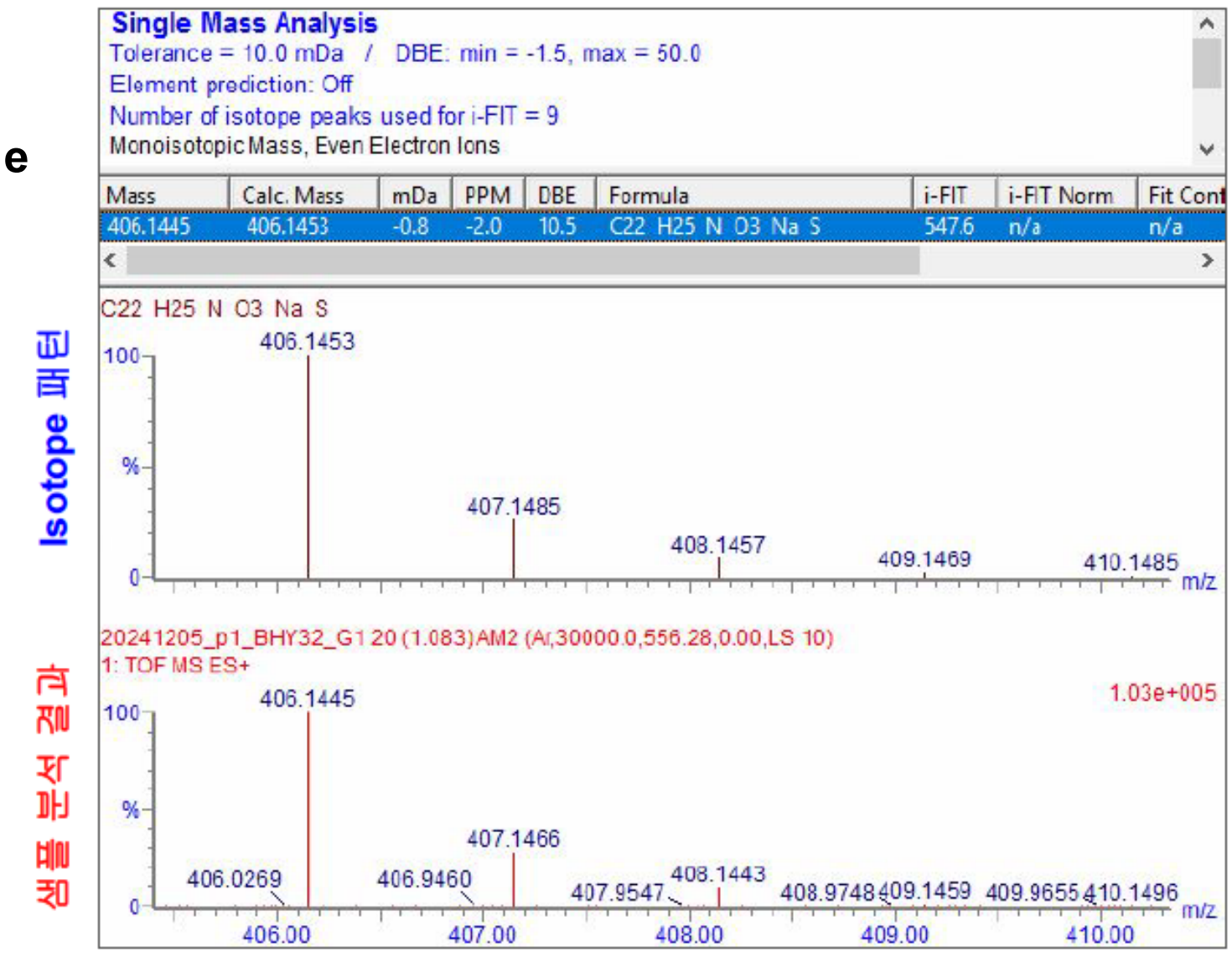

High-resolution mass spectra (**58**)

No.	Sample name	Chemical Formula	Exact Mass (M)	Calcd for [M+Na]+	found Mass [M+Na]+
33	G2	C20H31NO4S	381.1974	404.1866	404.1868

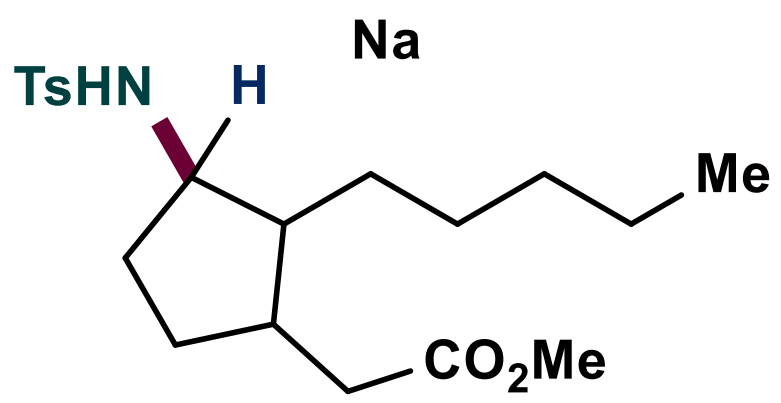

59

Exact Mass: 404.1871

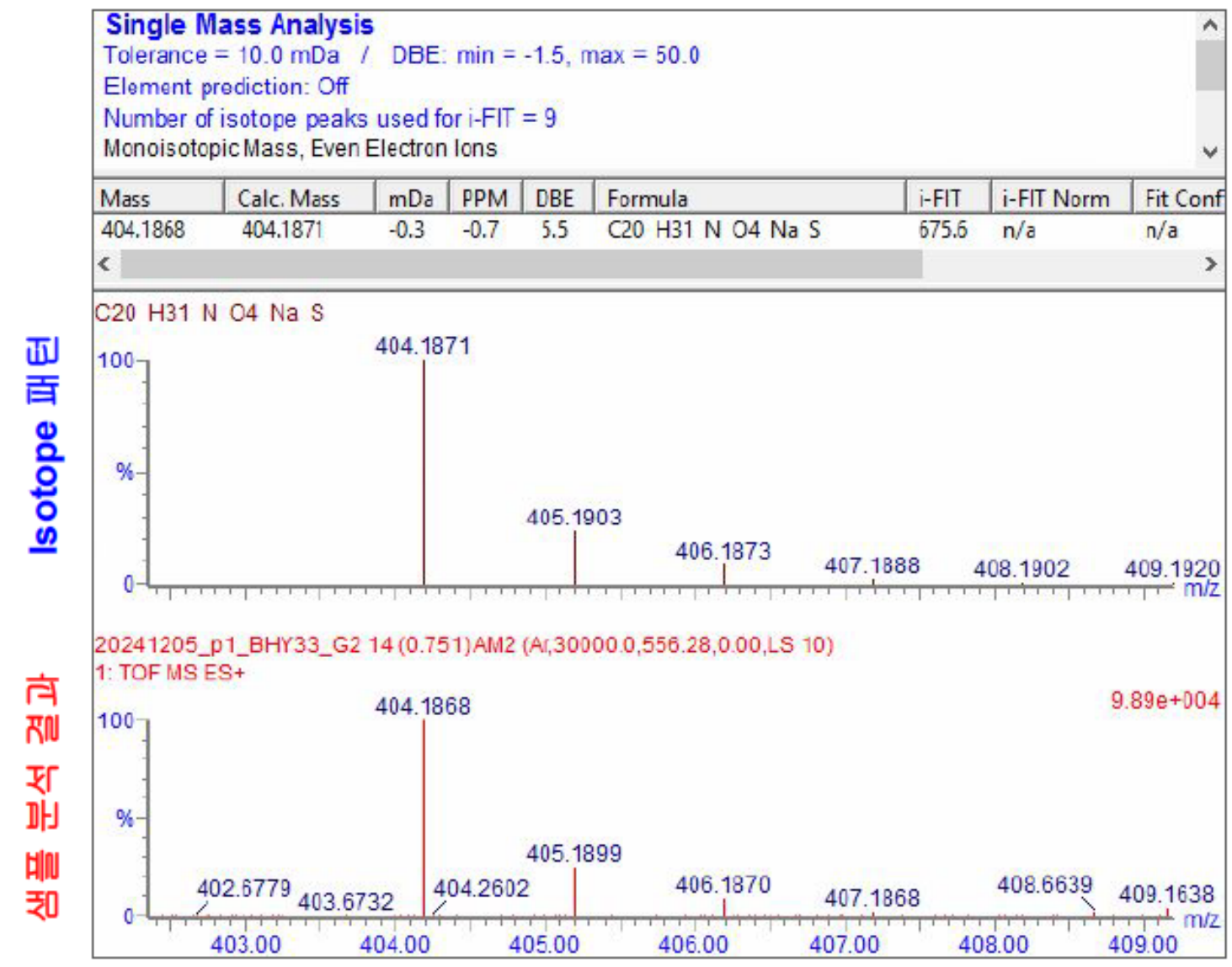

High-resolution mass spectra (**59**)

No.	Sample name	Chemical Formula	Exact Mass (M)	Calcd for [M+Na]+	found Mass [M+Na]+
22	E2	C16H25NO4S	327.1504	350.1396	350.1399

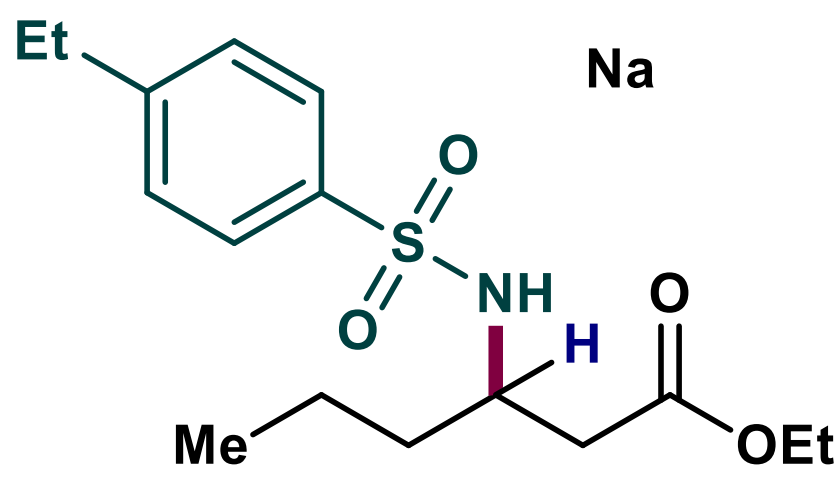

60

Exact Mass: 350.1402

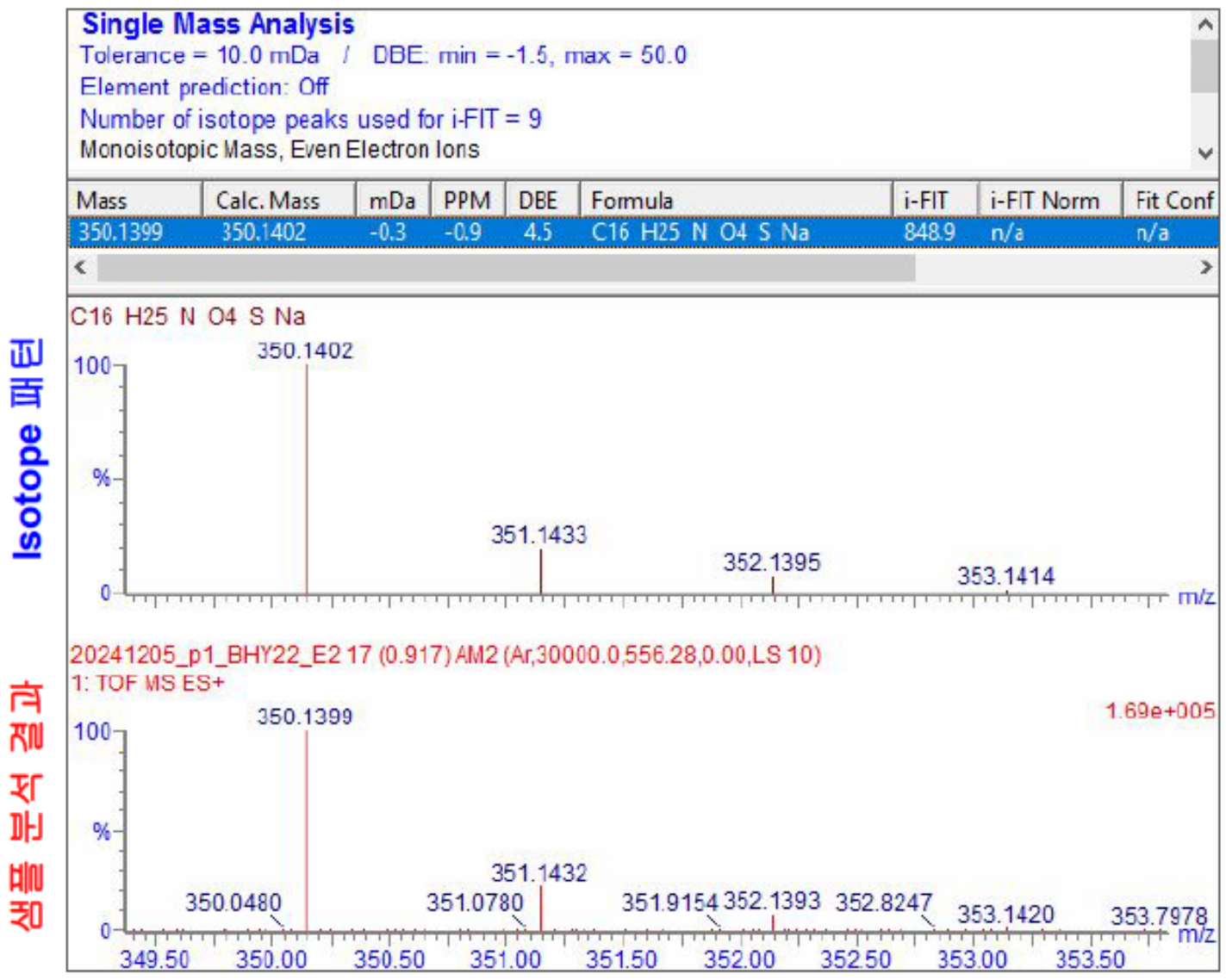

High-resolution mass spectra (**60**)

No.	Sample name	Chemical Formula	Exact Mass (M)	Calcd for [M+Na]+	found Mass [M+Na]+
21	E1	C14H21NO4S	299.1191	322.1083	322.1083

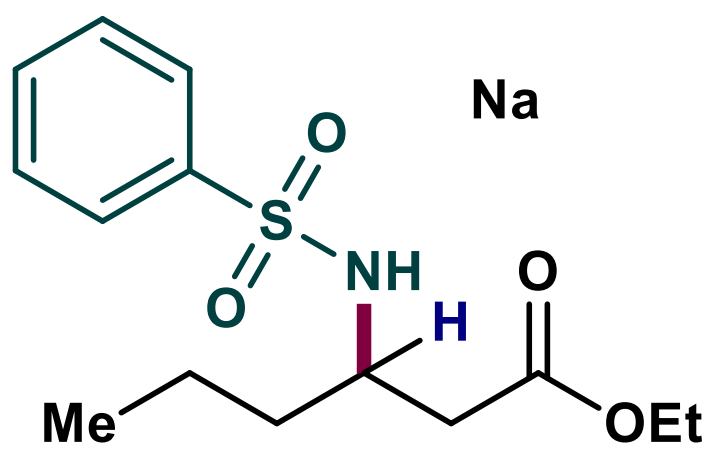

61

Exact Mass: 322.1089

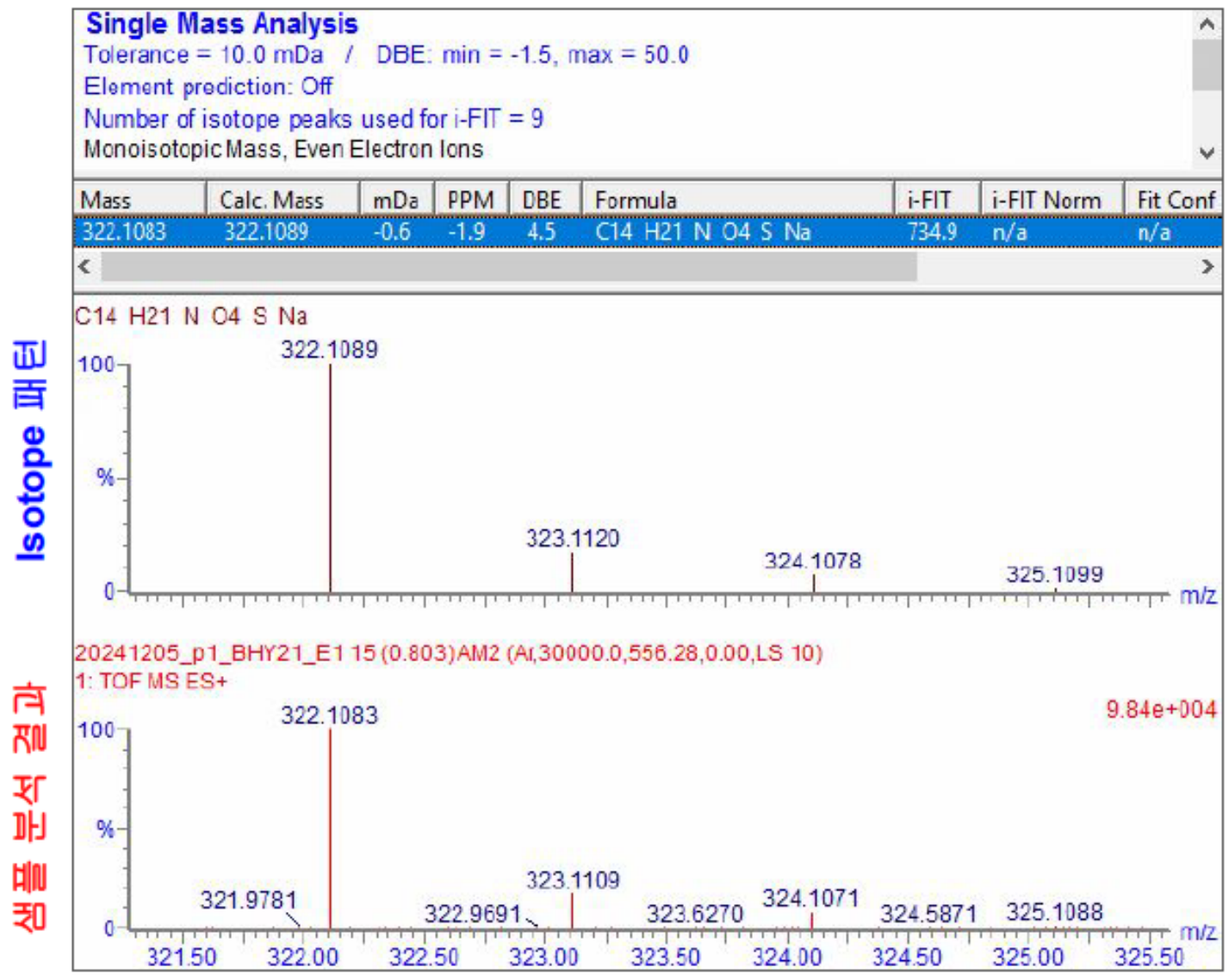

High-resolution mass spectra (**61**)

No.	Sample name	Chemical Formula	Exact Mass (M)	Calcd for [M+Na]+	found Mass [M+Na]+
24	E4	C14H20FNO4S	317.1097	340.0989	340.0991

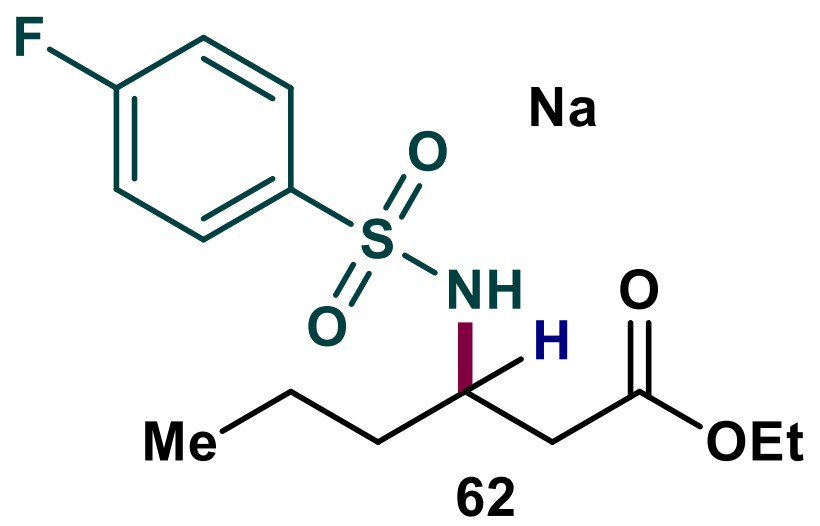

62

Exact Mass: 340.0995

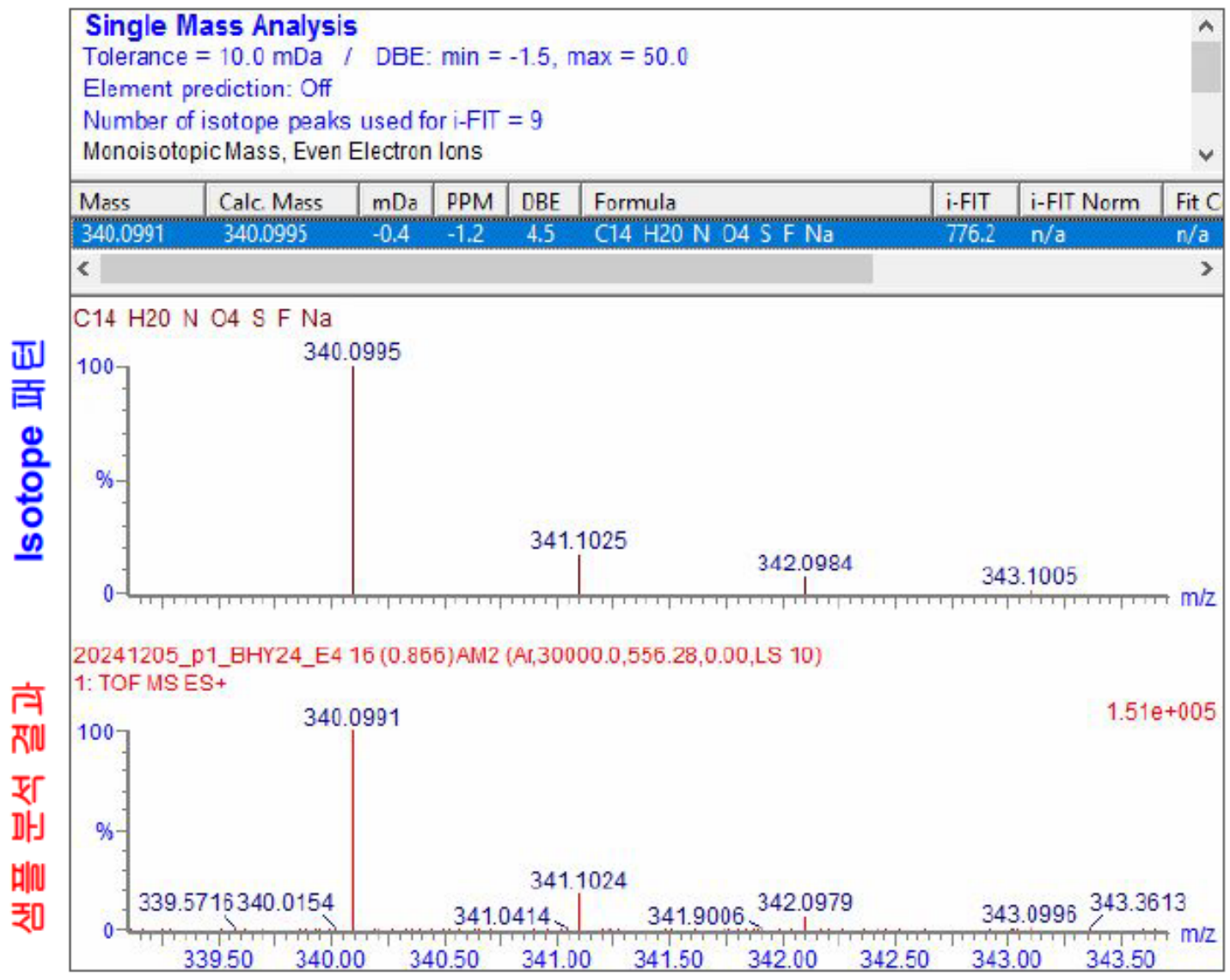

High-resolution mass spectra (**62**)

No.	Sample name	Chemical Formula	Exact Mass (M)	Calcd for [M+Na]+	found Mass [M+Na]+
23	E3	C14H20ClNO4S	333.0802	356.0694	356.0695

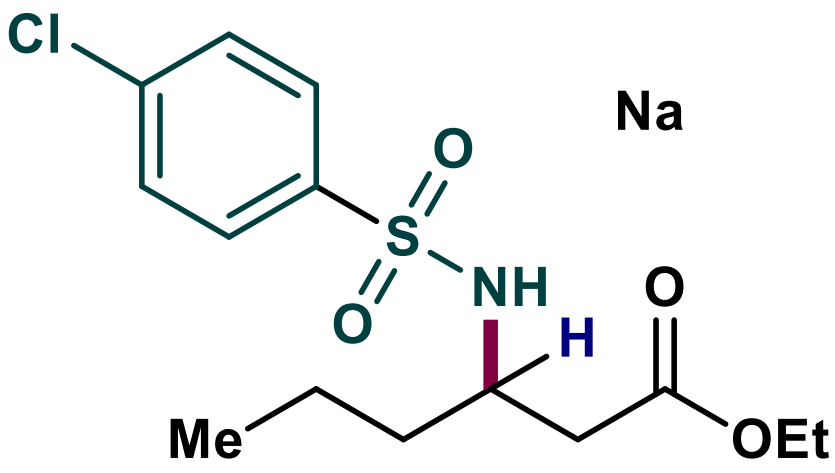

63

Exact Mass: 356.0699

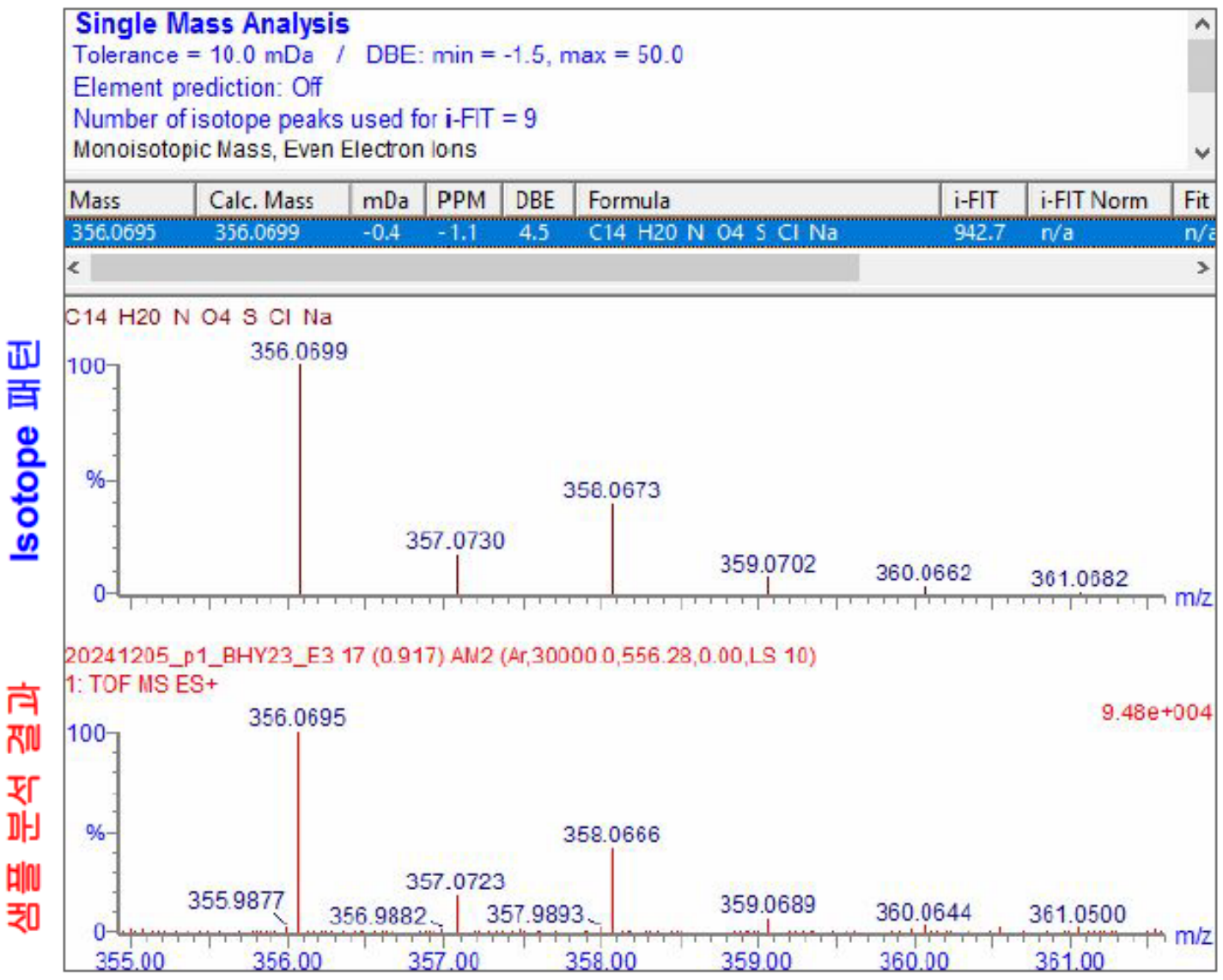

High-resolution mass spectra (**63**)

No.	Sample name	Chemical Formula	Exact Mass (M)	Calcd for [M+Na]+	found Mass [M+Na]+
27	E7	C12H19NO4S2	305.0755	328.0647	328.0645

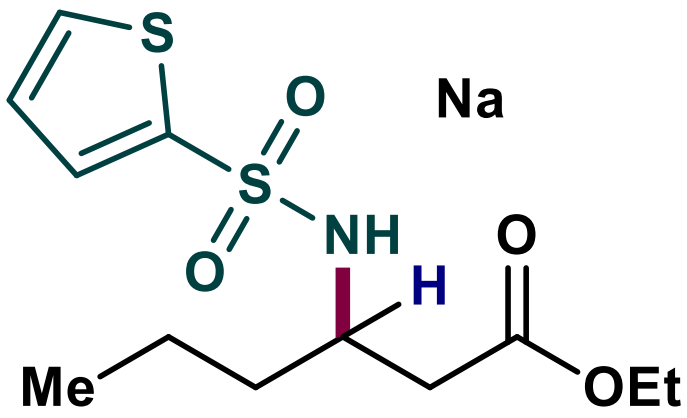

64

Exact Mass: 328.0653

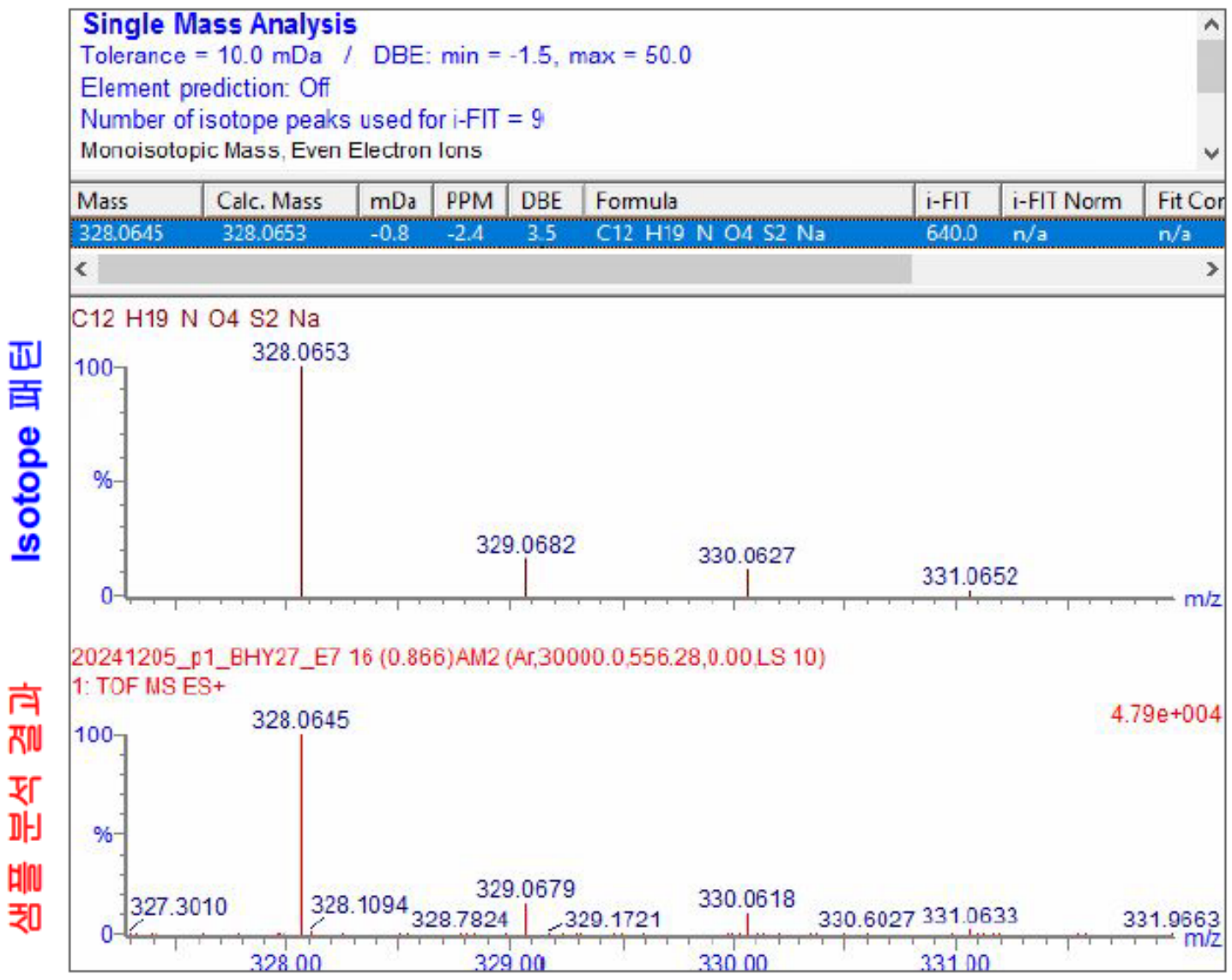

High-resolution mass spectra (**64**)

No.	Sample name	Chemical Formula	Exact Mass (M)	Calcd for [M+Na]+	found Mass [M+Na]+
26	E6	C18H23NO4S	349.1348	372.1240	372.1239

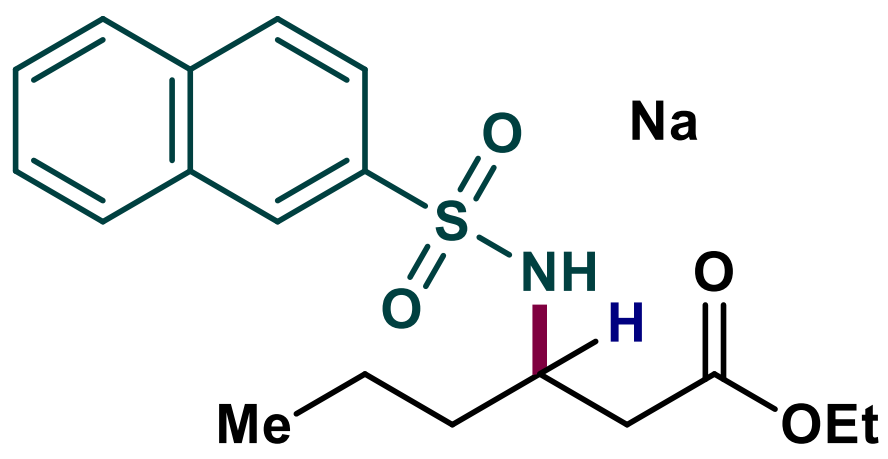

65

Exact Mass: 372.1245

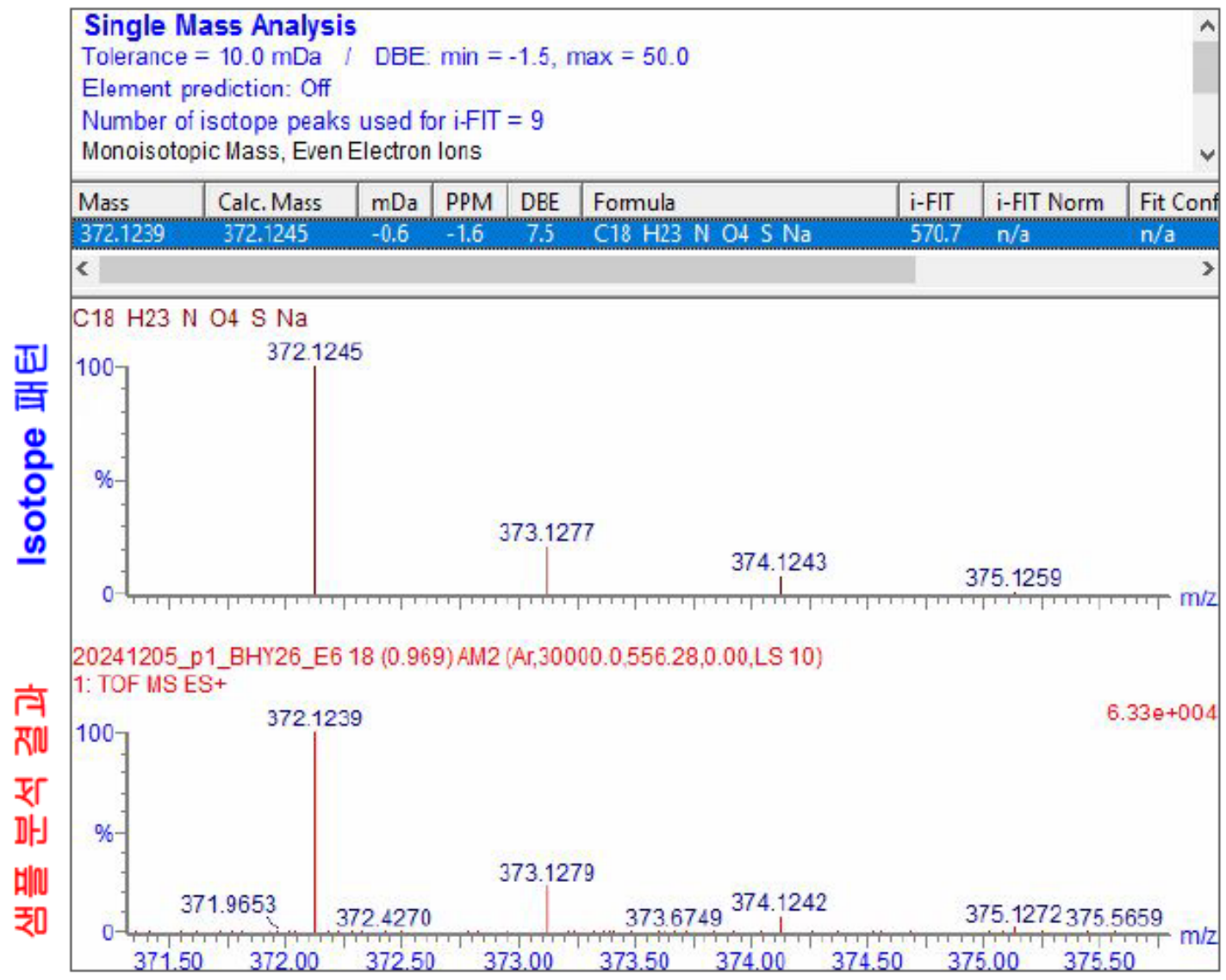

High-resolution mass spectra (**65**)

No.	Sample name	Chemical Formula	Exact Mass (M)	Calcd for [M+Na]+	found Mass [M+Na]+
25	E5	C15H20F3NO4S	367.1065	390.0957	390.0962

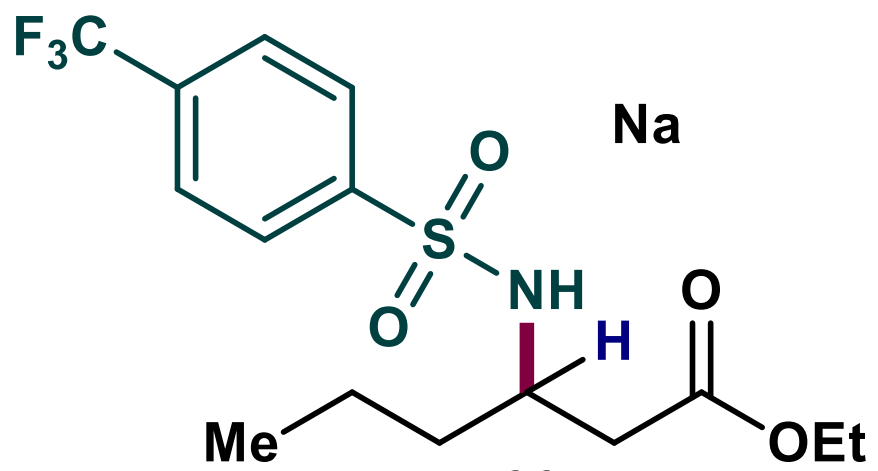

66

Exact Mass: 390.0963

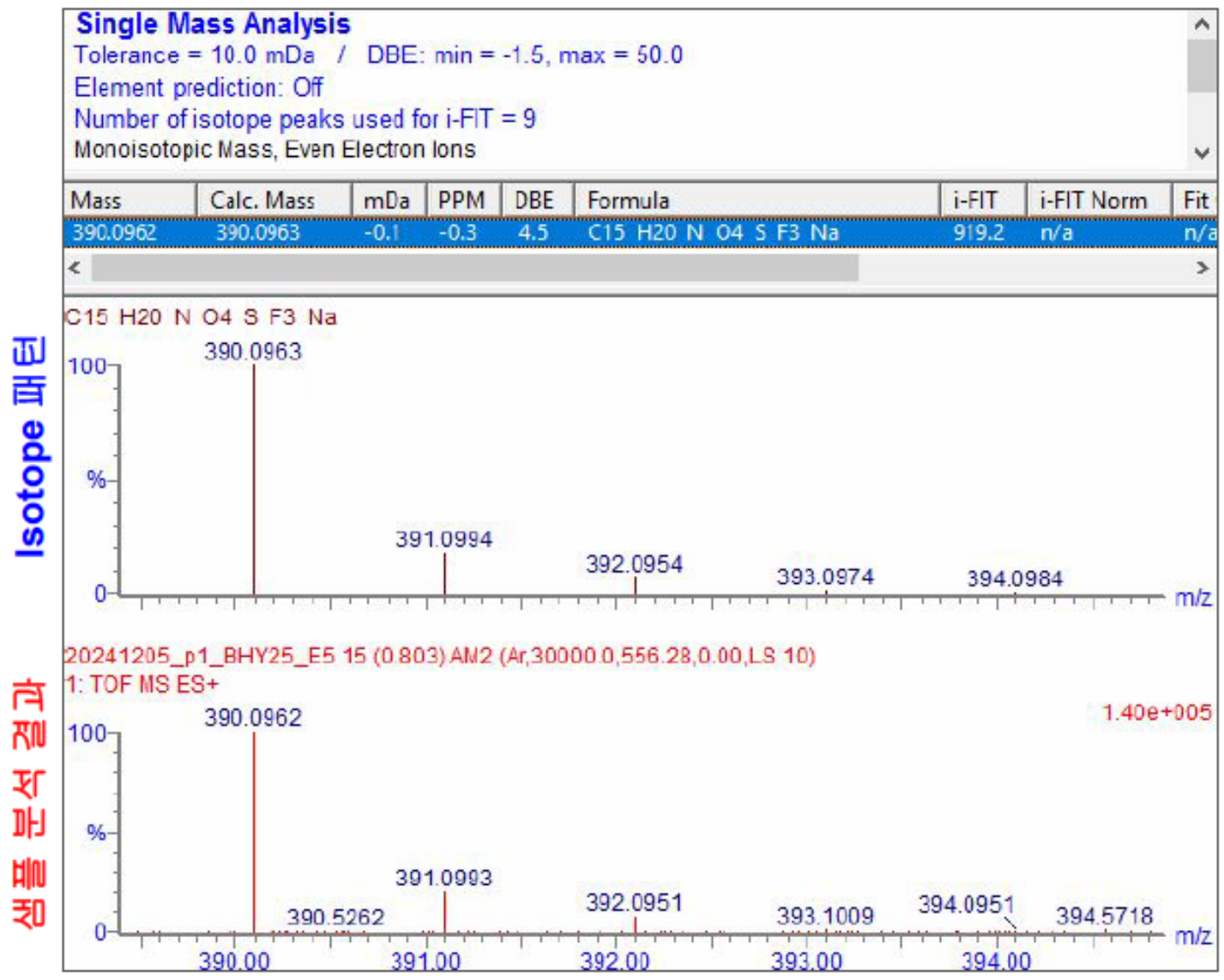

High-resolution mass spectra (**66**)

No.	Sample name	Chemical Formula	Exact Mass (M)	Calcd for [M+Na]+	found Mass [M+Na]+
29	E9	C12H12F3NO4S	323.0439	346.0331	346.0335

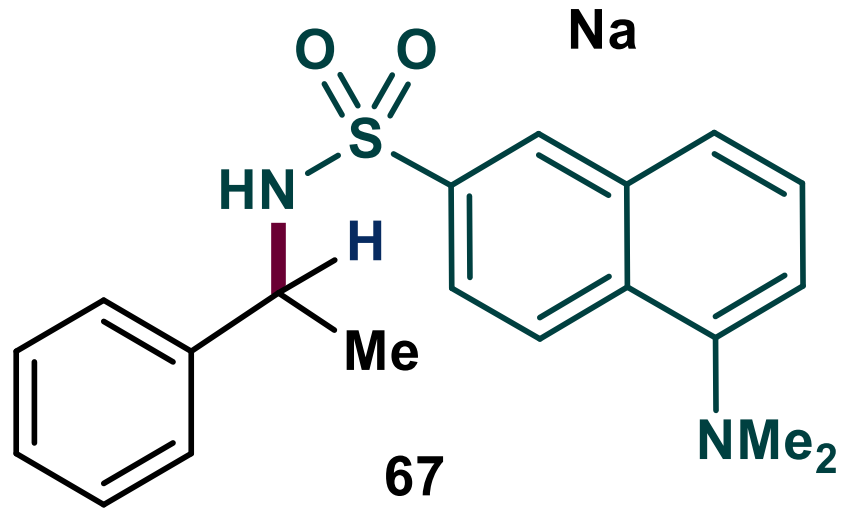

67

Exact Mass: 377.1300

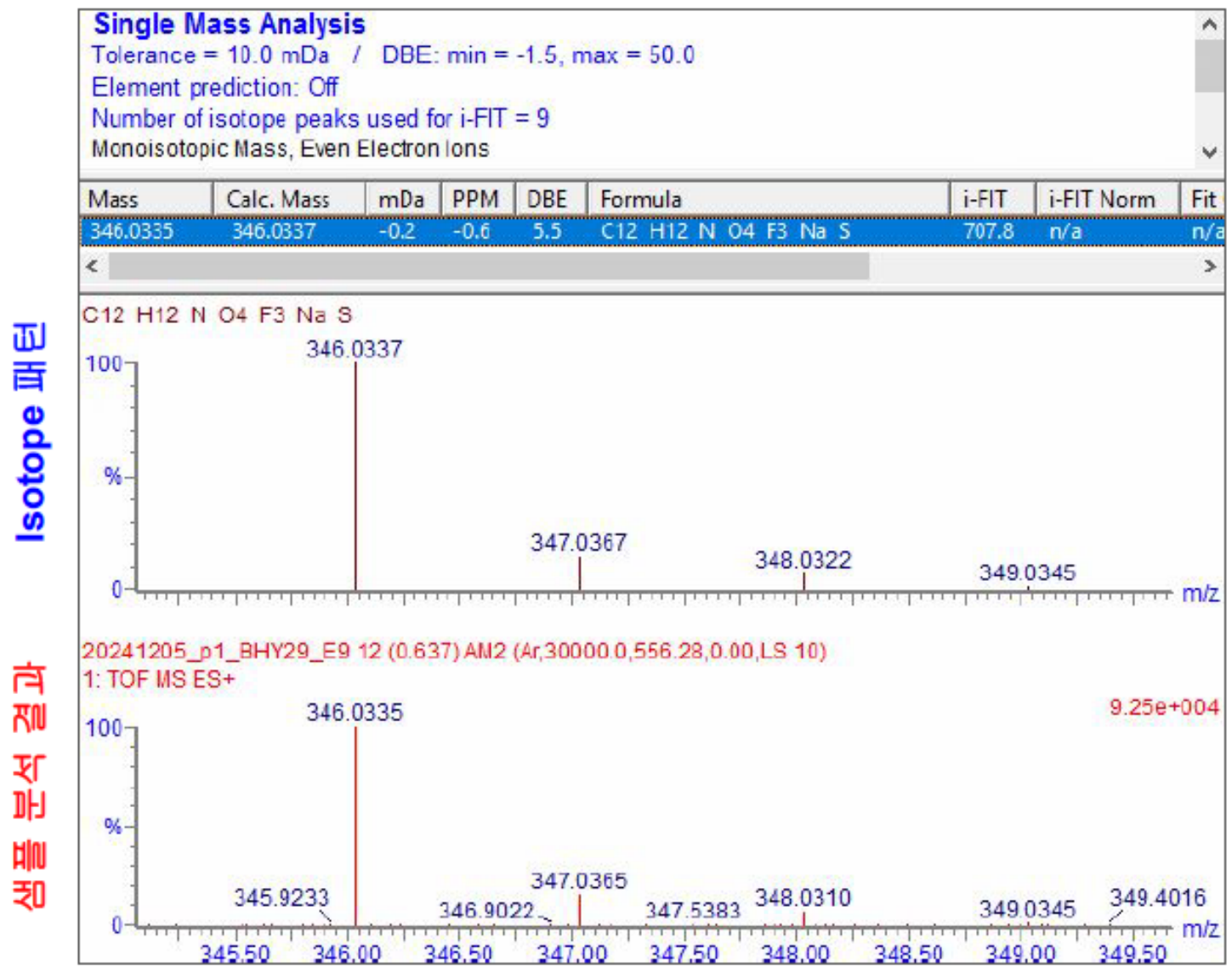

High-resolution mass spectra (**67**)

No.	Sample name	Chemical Formula	Exact Mass (M)	Calcd for [M+Na]+	found Mass [M+Na]+
18	D3	C16H23NO3S	309.1399	332.1291	332.1292

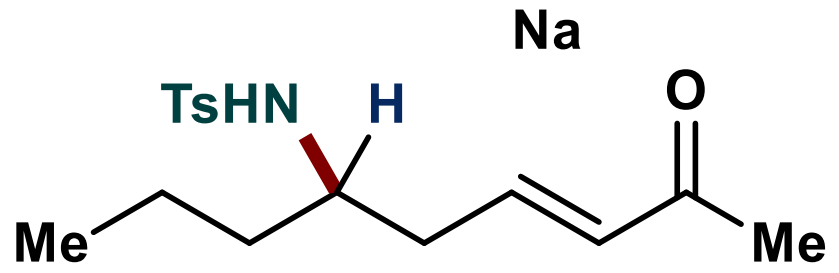

70

Exact Mass: 332.1296

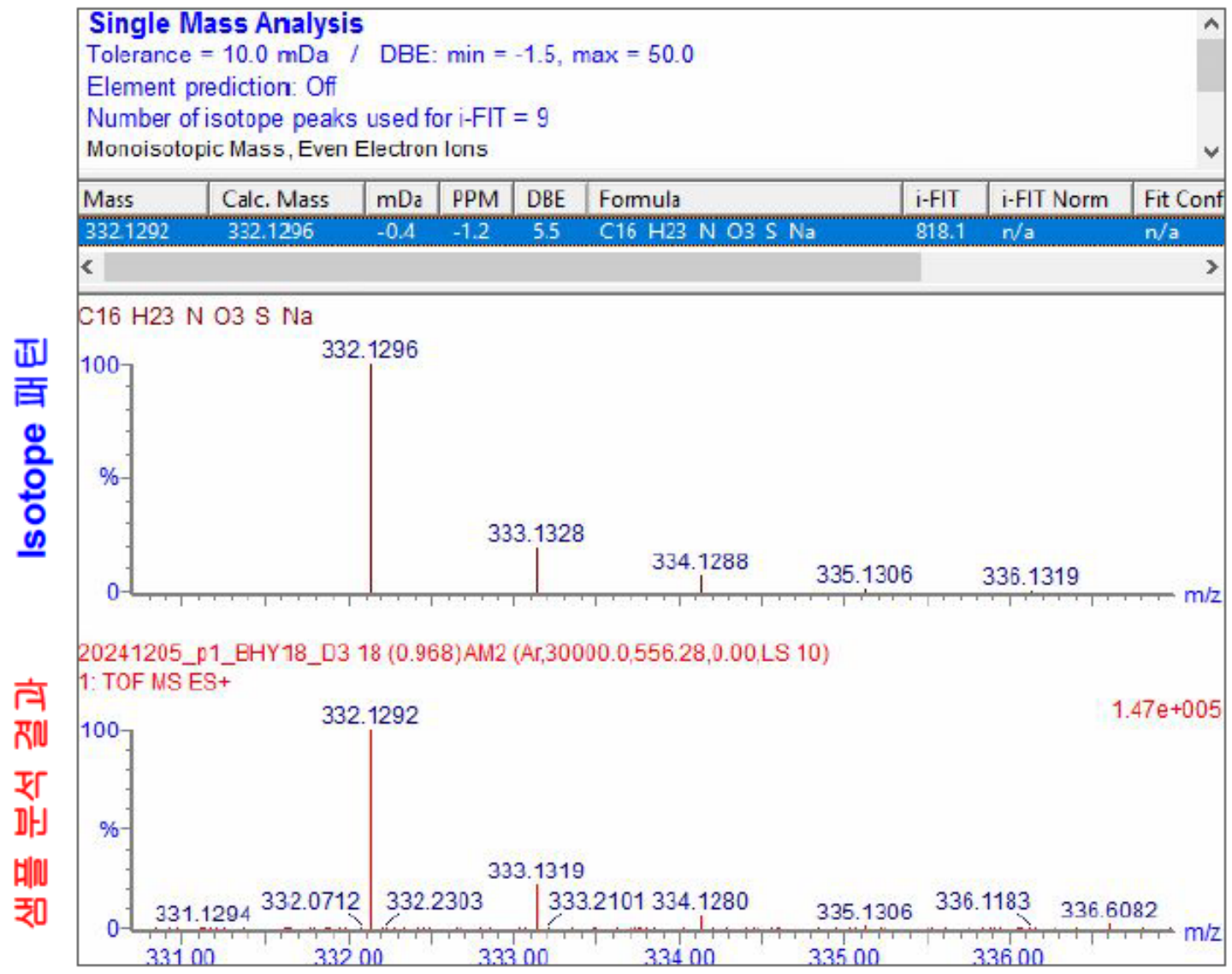

High-resolution mass spectra (**70**)

No.	Sample name	Chemical Formula	Exact Mass (M)	Calcd for [M+Na]+	found Mass [M+Na]+
19	D5	C13H19NO2S	253.1136	276.1028	276.1032

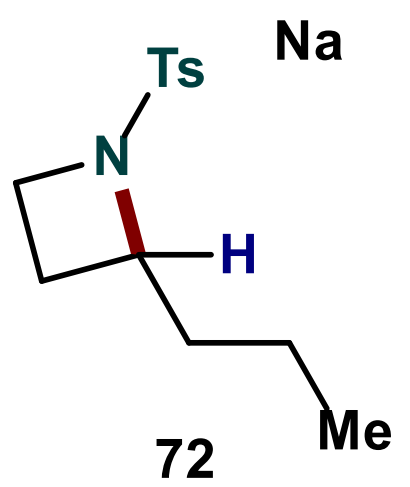

72

Exact Mass: 276.1034

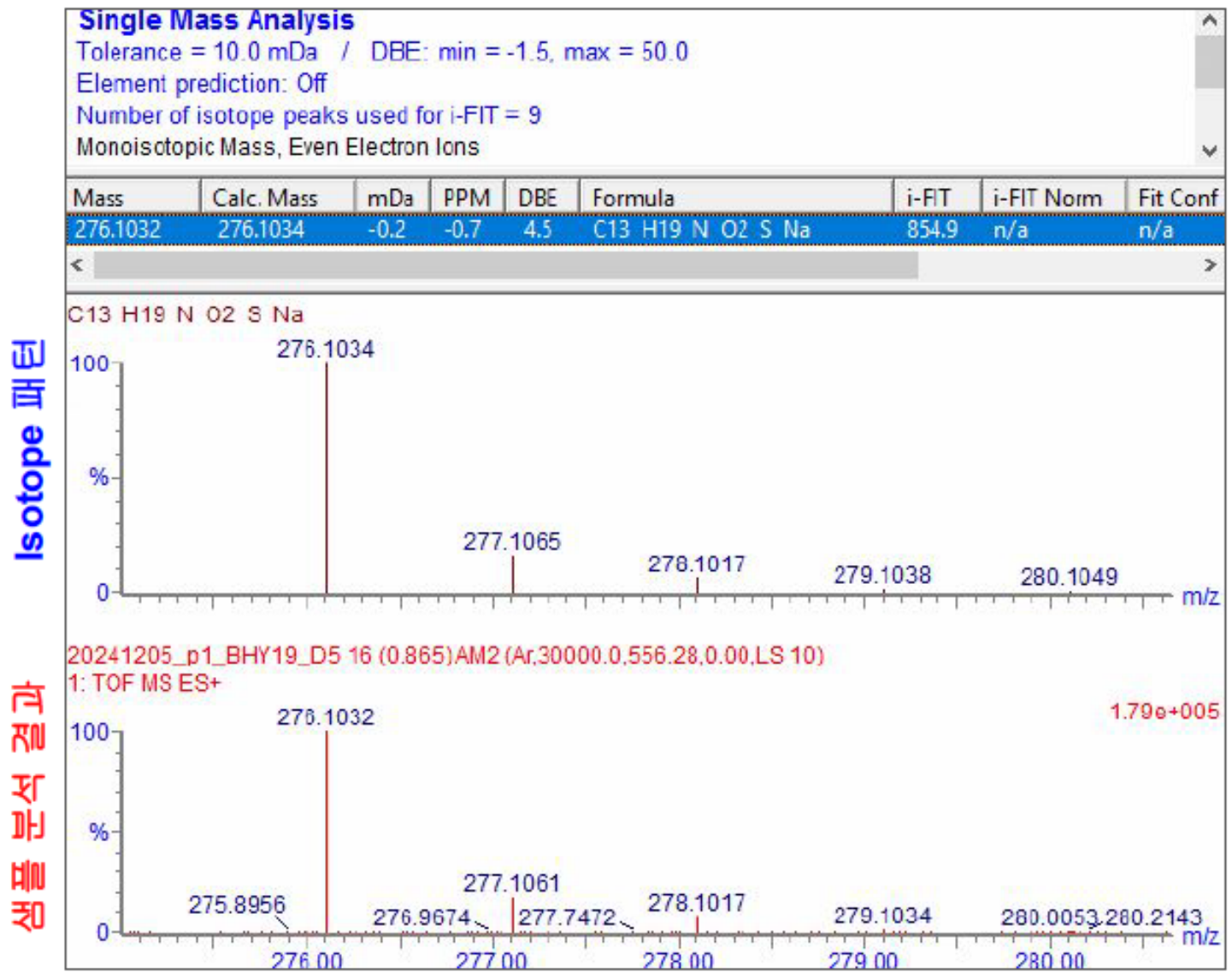

High-resolution mass spectra (**72**)

No.	Sample name	Chemical Formula	Exact Mass (M)	Calcd for [M+Na]+	found Mass [M+Na]+
20	D6	C13H19NO4S	285.1035	308.0927	308.0929

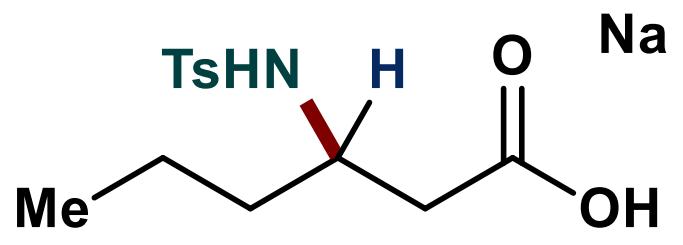

73

Exact Mass: 308.0932

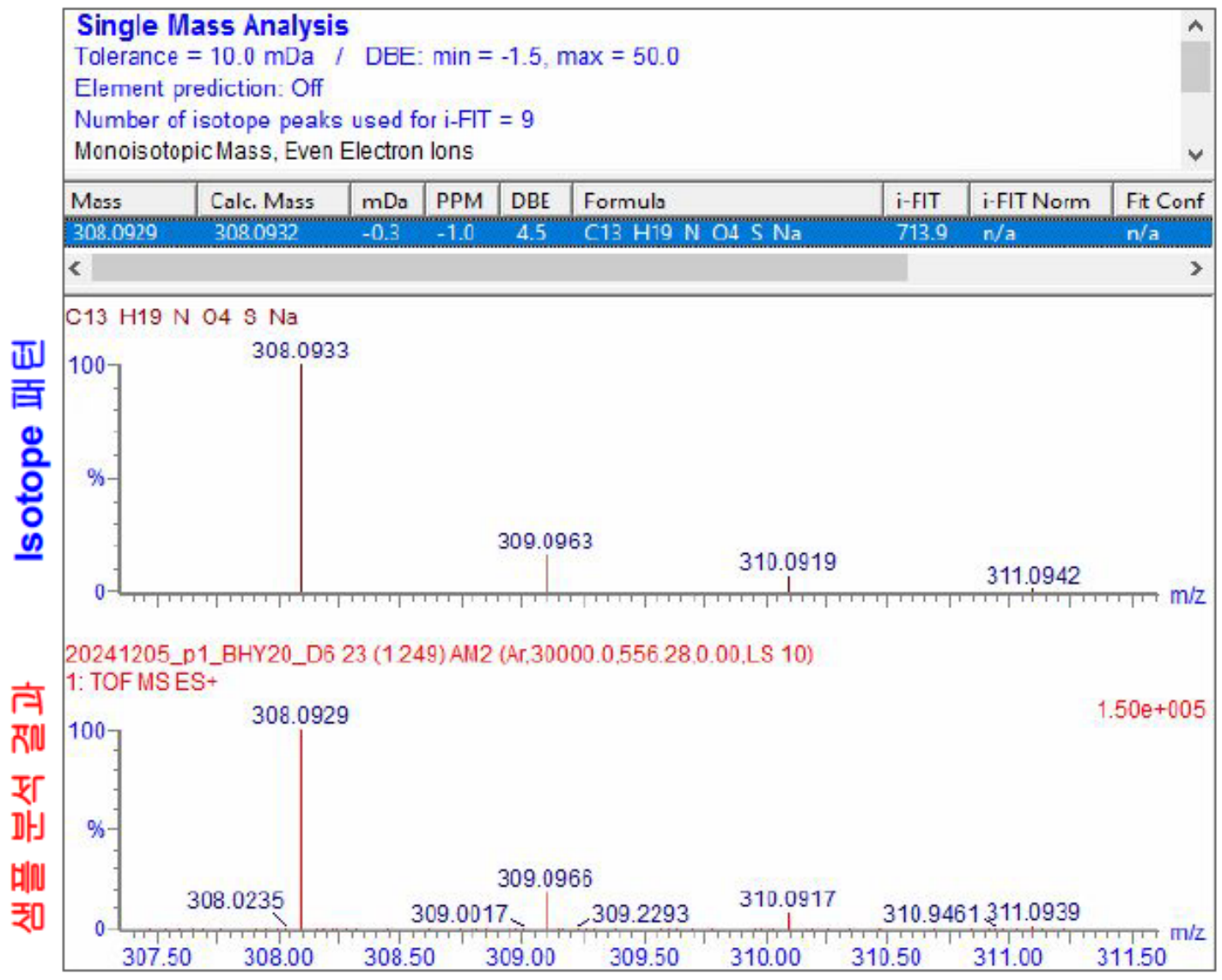

High-resolution mass spectra (**73**)

No.	Sample name	Chemical Formula	Exact Mass (M)	Calcd for [M+H]+	found Mass [M+H]+
2	N-Ts-ST	$C_{23}H_{21}F_6N_5O_3S$	561.1269	562.1342	562.1349

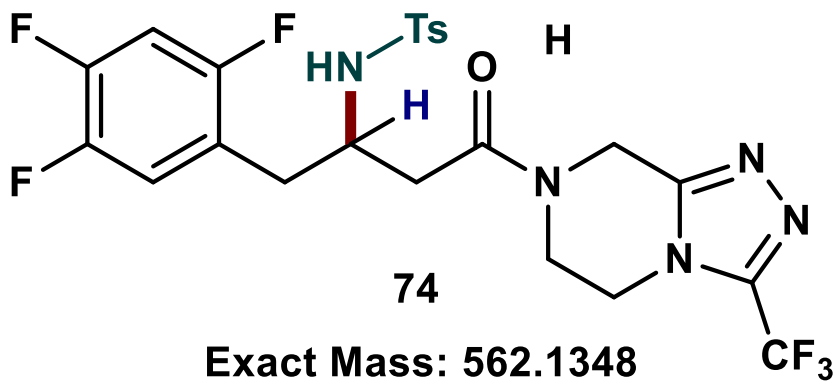

74

Exact Mass: 562.1348

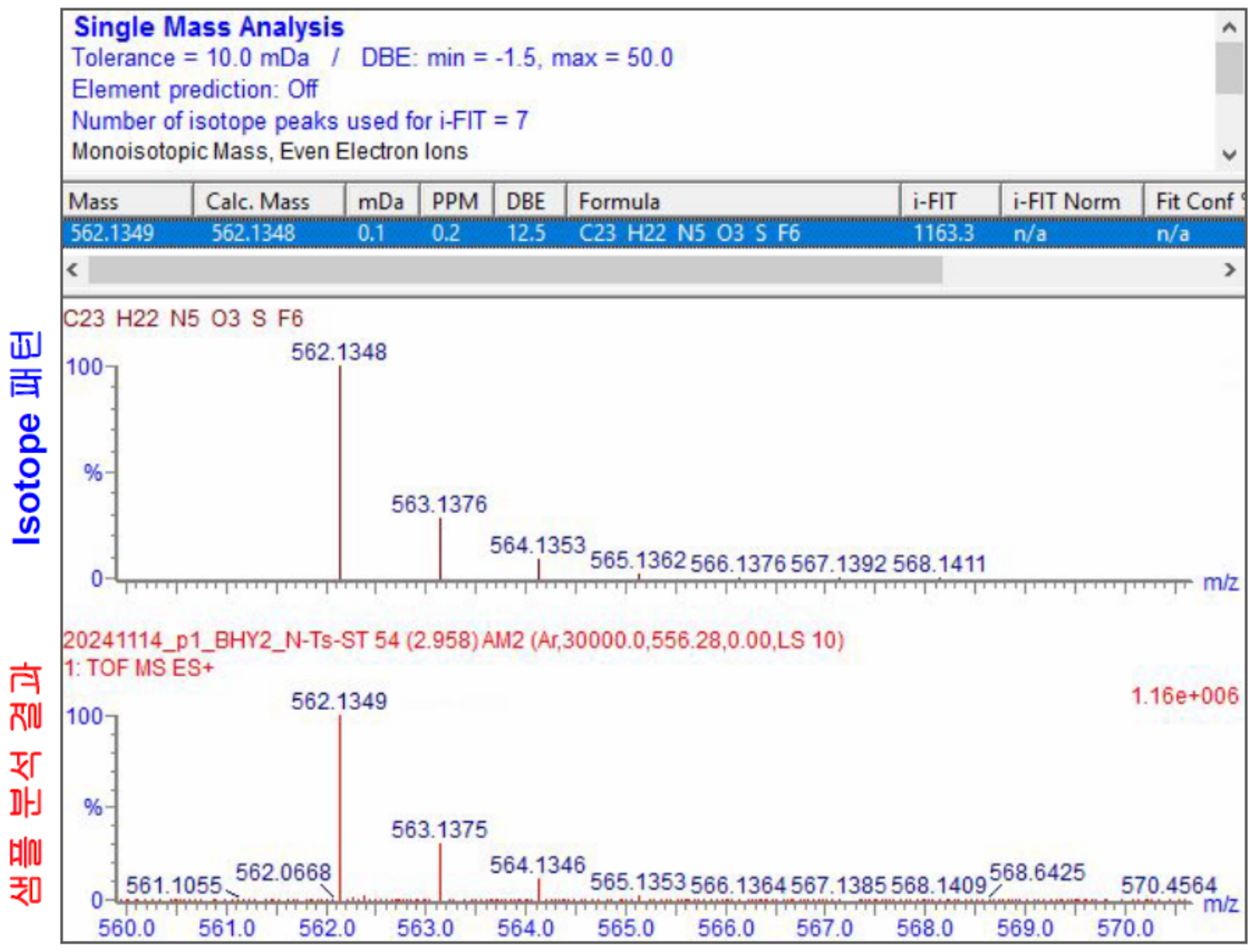

High-resolution mass spectra (**74**)

No.	Sample name	Chemical Formula	Exact Mass (M)	Calcd for [M+H]+	found Mass [M+H]+
3	ST	$C_{16}H_{15}F_6N_5O$	407.1181	408.1254	408.1257

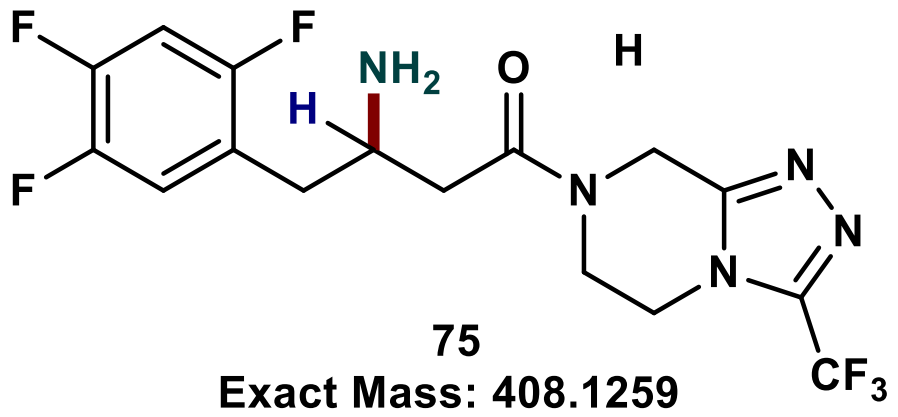

75
Exact Mass: 408.1259

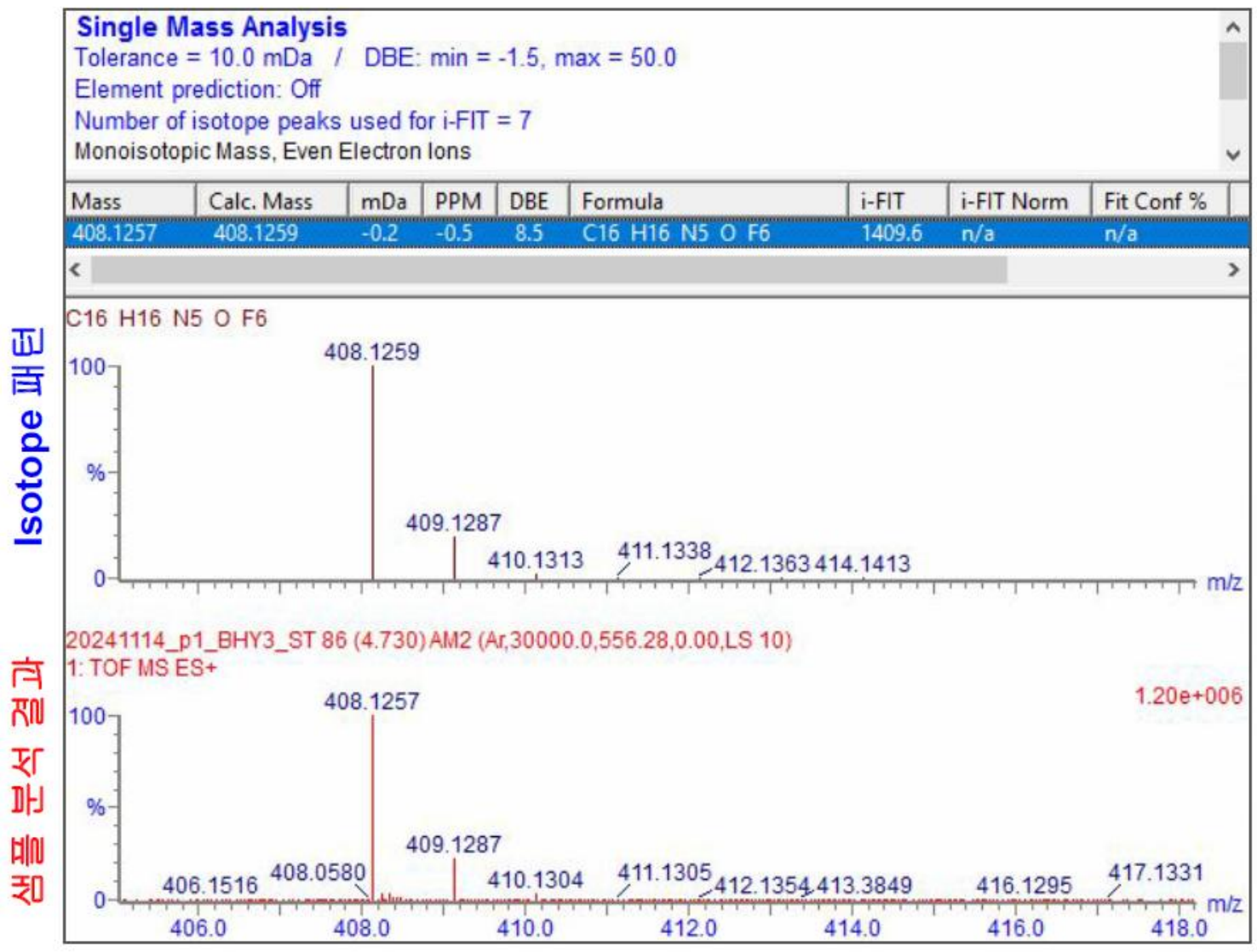

High-resolution mass spectra (**75**)

No.	Sample name	Chemical Formula	Exact Mass (M)	Calcd for [M+H]+	found Mass [M+H]+
30	E12	C16H19F6NO2	371.1320	372.1393	372.1398

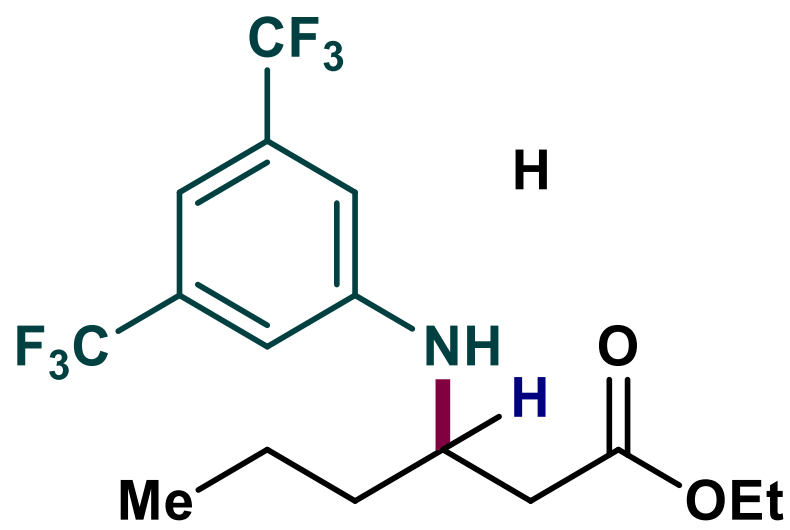

1a

Exact Mass: 372.1398

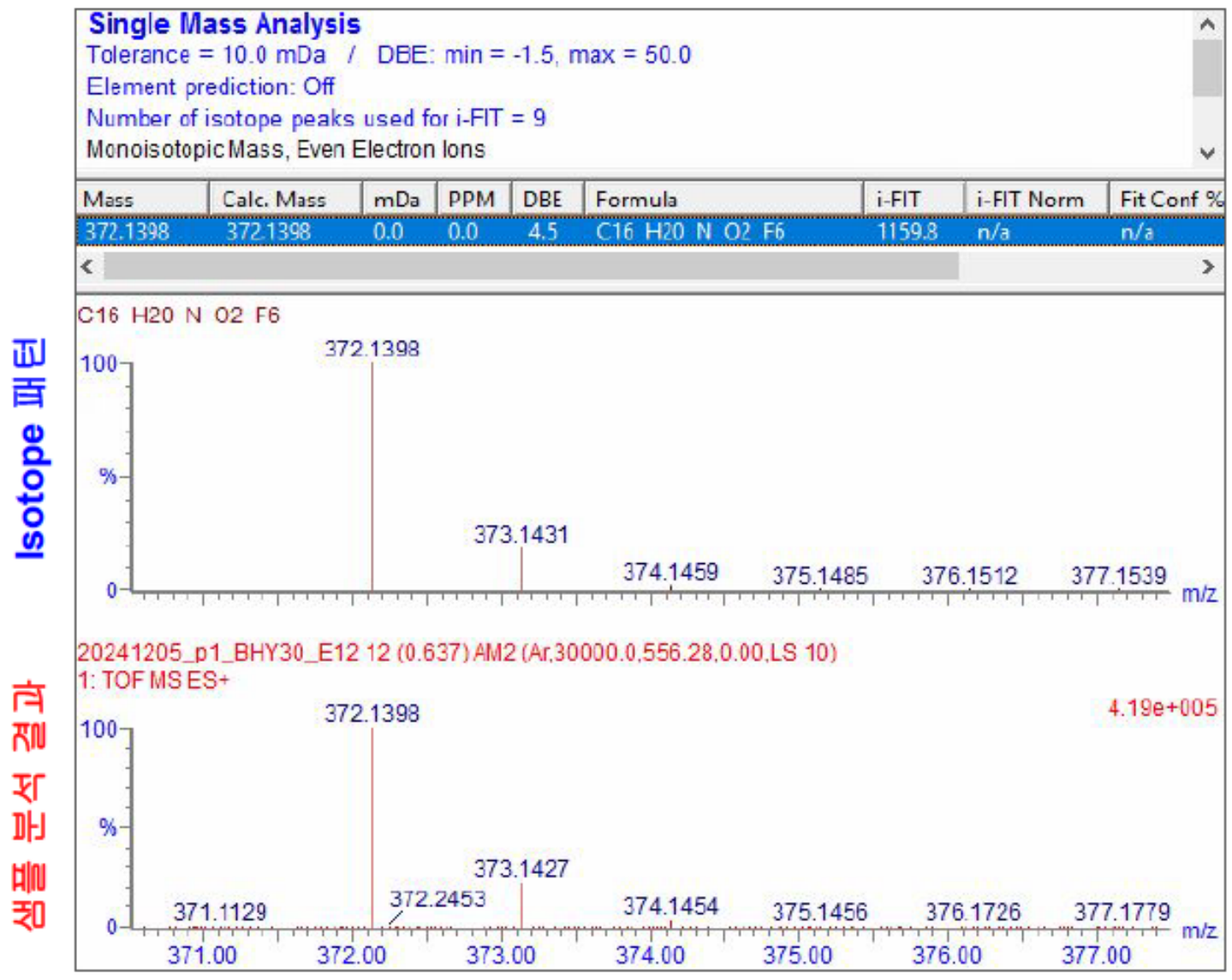

High-resolution mass spectra (**1a**)

No.	Sample name	Chemical Formula	Exact Mass (M)	Calcd for [M+Na]+	found Mass [M+Na]+
31	E13	C23H27NO4	381.1940	404.1832	404.1835

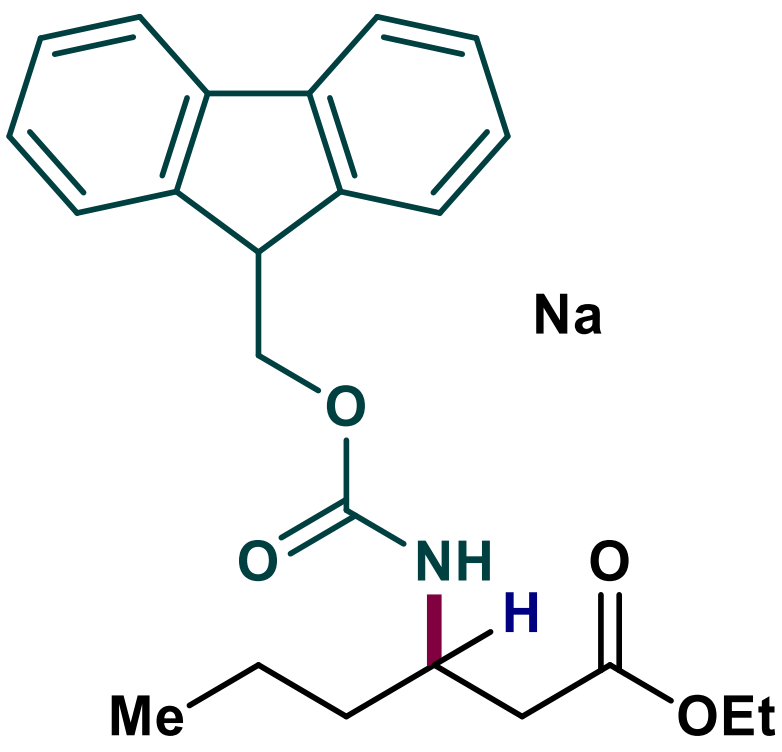

1d

Exact Mass: 404.1838

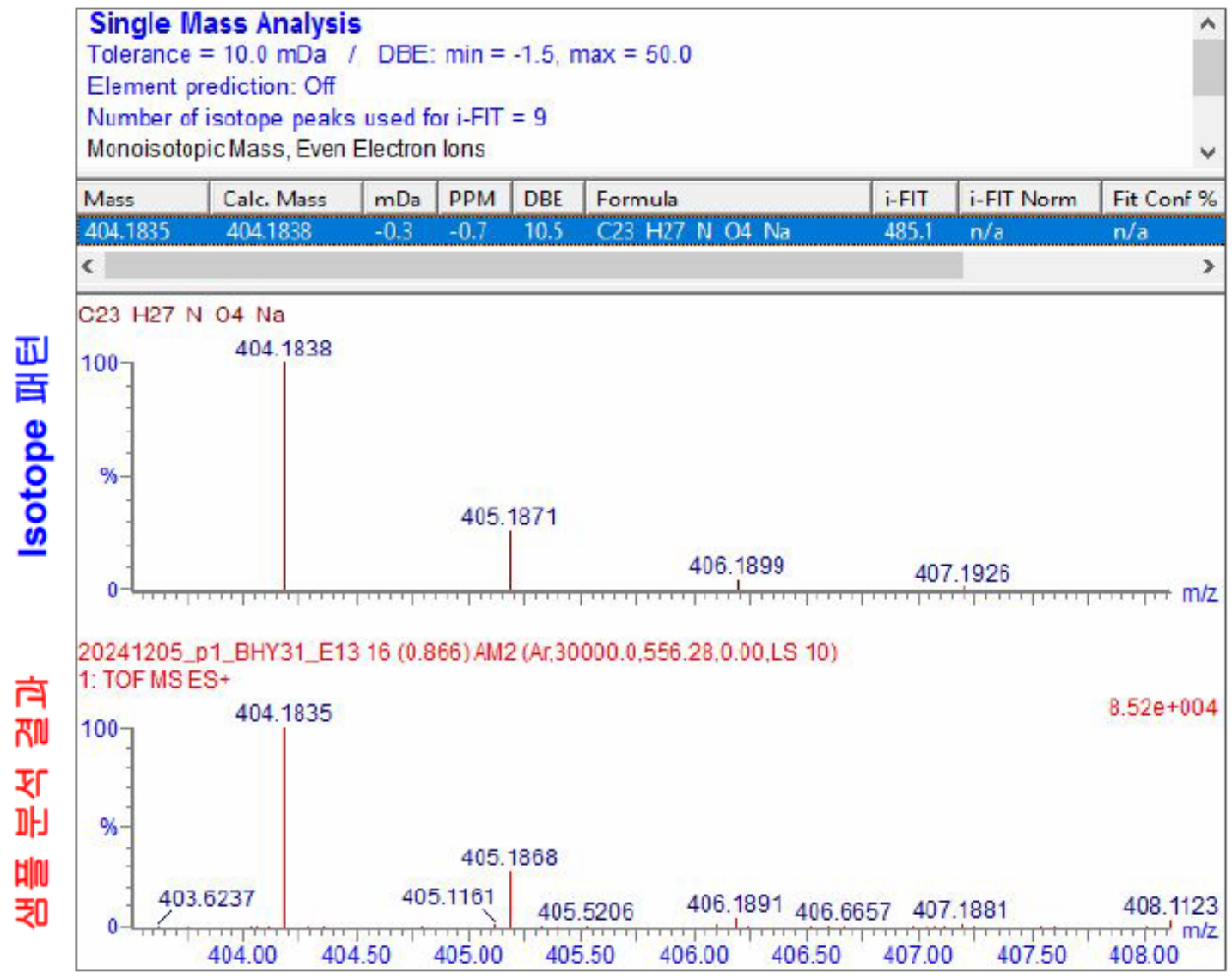

High-resolution mass spectra (**1d**)

No.	Sample name	Chemical Formula	Exact Mass (M)	Calcd for [M+Na]+	found Mass [M+Na]+
28	E8	C20H22N2O2S	354.1402	377.1294	377.1301

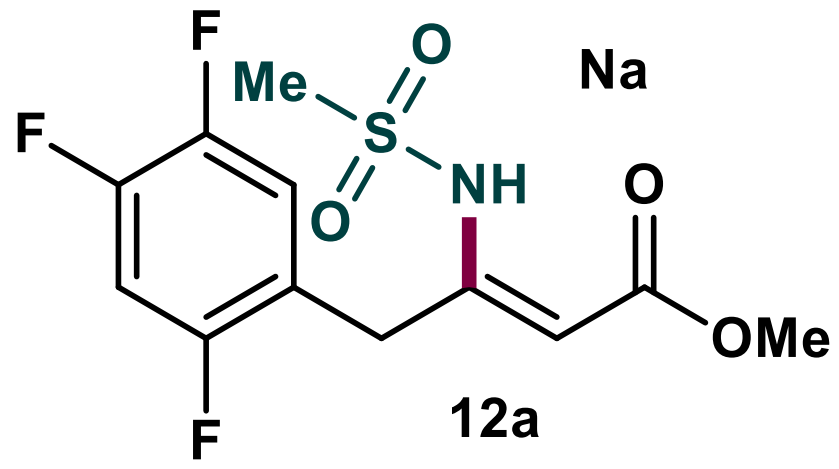

12a

Exact Mass: 346.0337

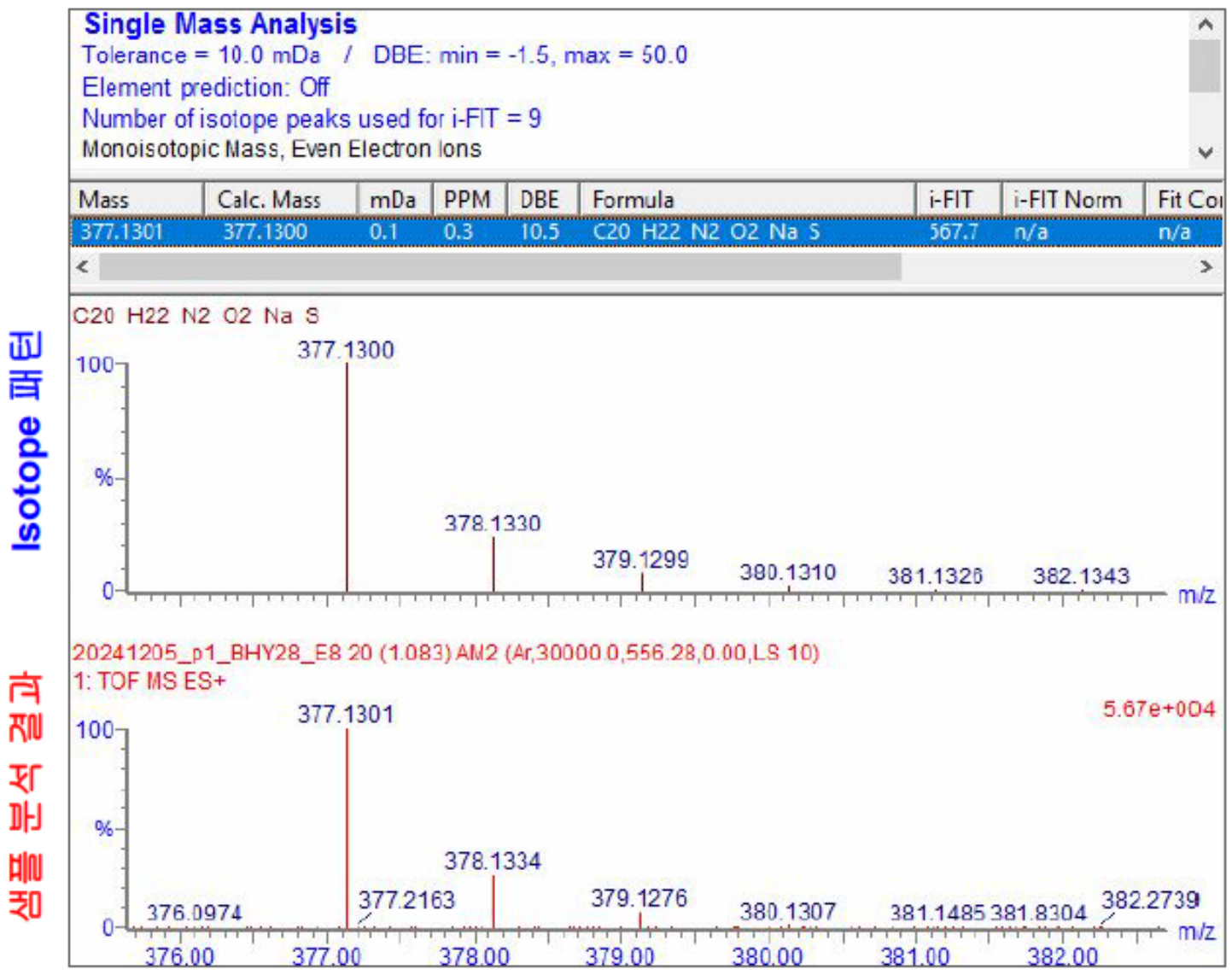

High-resolution mass spectra (**12a**)

No.	Sample name	Chemical Formula	Exact Mass (M)	[M+Na]
2		$C_{15}H_{21}NO_4S$	311.1191	334.1089

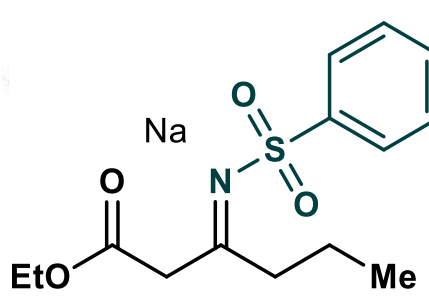

Exact Mass: 334.1089

HR-MS analysis (SFC-QTOF) $[C_{15}H_{21}NO_4S^+ + Na]^+$
m/z 334.1089 (expected), 334.1091 (detected)

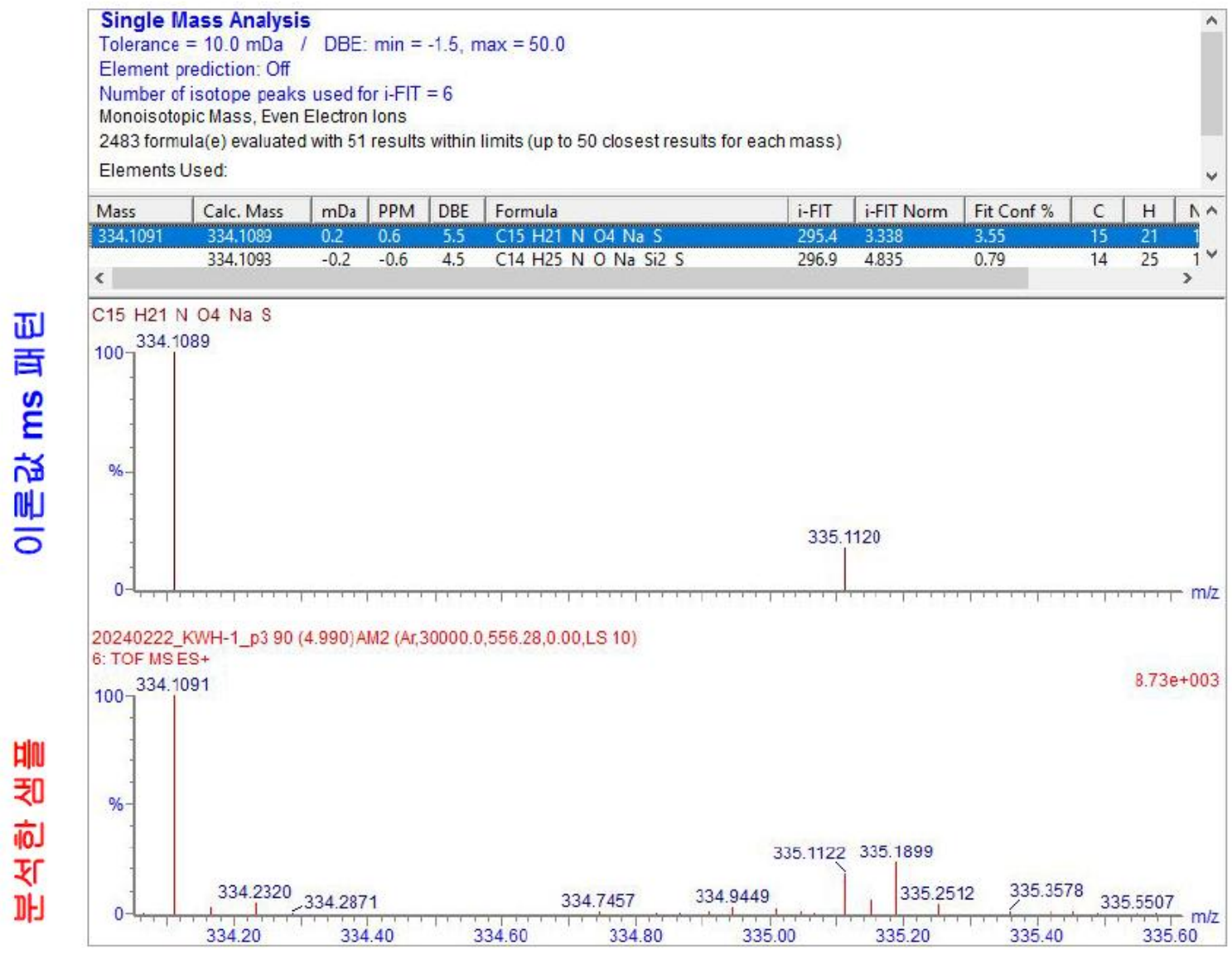

in-situ High-resolution mass spectra (**Int-3**$_{\mathbf{A}}$)

No.	Sample name	Chemical Formula	Exact Mass (M)	[M+H]
2		$C_{15}H_{21}NO_4S$	311.1191	312.1270

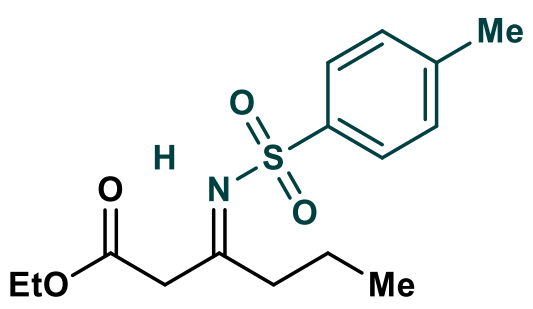

Exact Mass: 312.1270

HR-MS analysis (SFC-QTOF) $[C_{15}H_{21}NO_4S^{+} + H]^{+}$
m/z 312.1270 (expected), 312.1266 (detected)

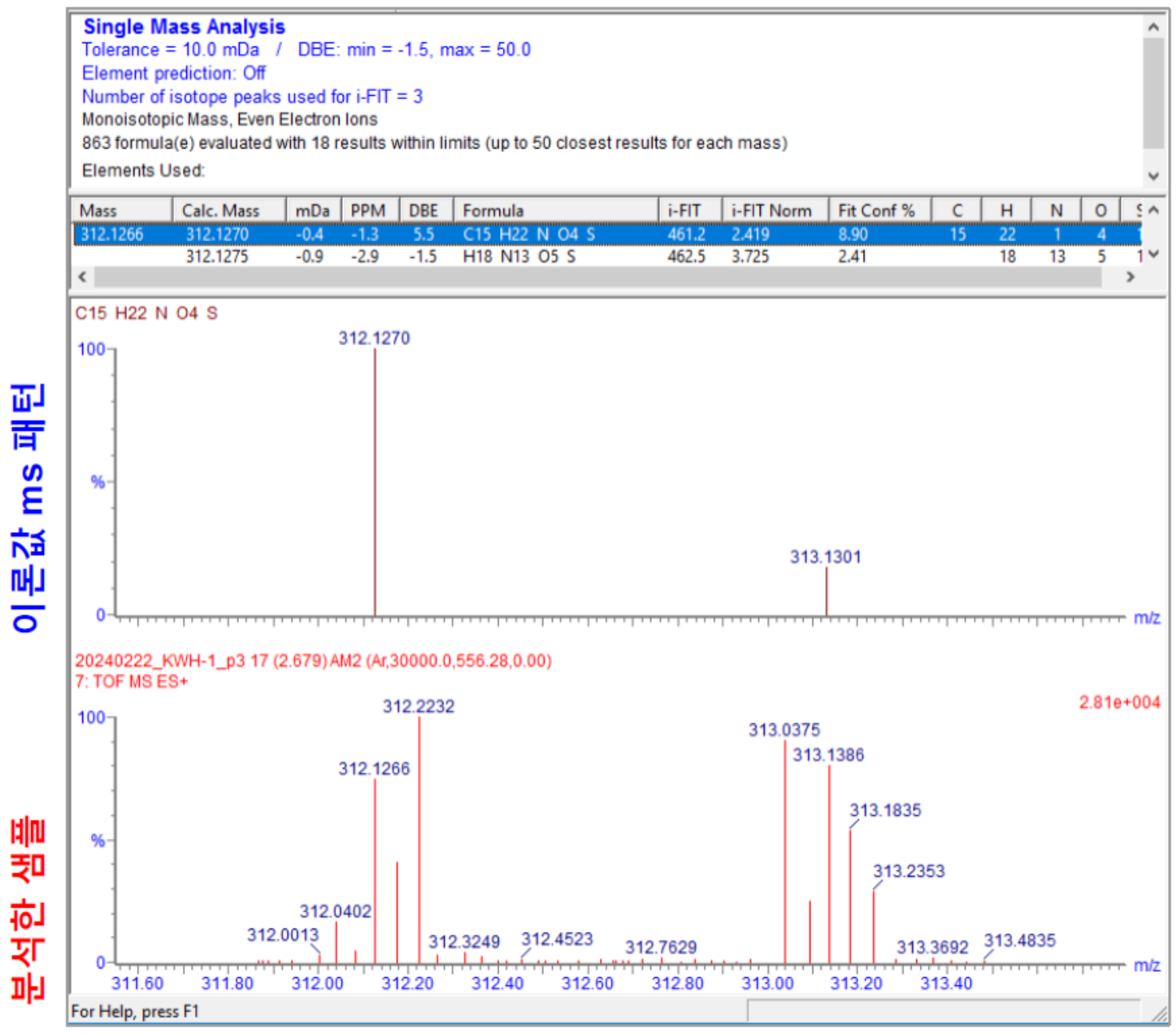

in-situ High-resolution mass spectra (**Int-3**$_{\mathbf{A}}$)

No.	Sample name	Chemical Formula	Exact Mass (M)	[M+Na]
4		$C_4H_{12}OSi$	104.0657	127.0555

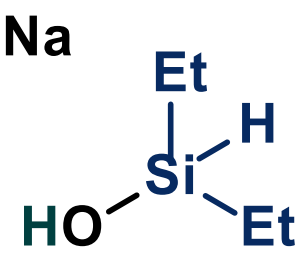

Exact Mass: 127.0555

HR-MS analysis (SFC-QTOF)
$[C_4H_{12}OSi + Na]^+$
m/z 127.0555 (expected),
127.0551 (detected)

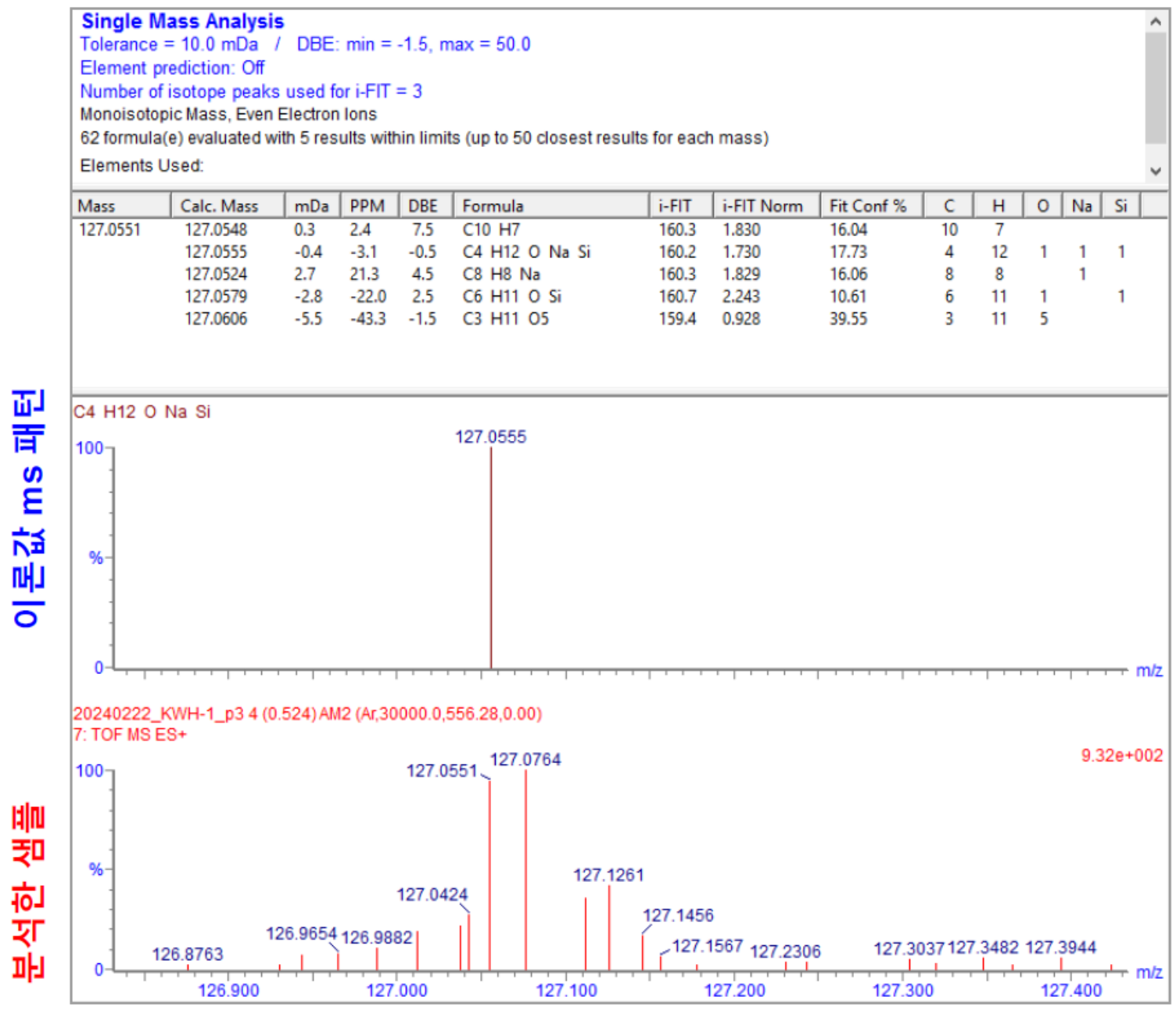

in-situ High-resolution mass spectra (**Et₂HSi-OH**)

No.	Sample name	Chemical Formula	Exact Mass (M)	[M+Na]
5		$C_8H_{22}OSi_2$	190.1209	213.1107

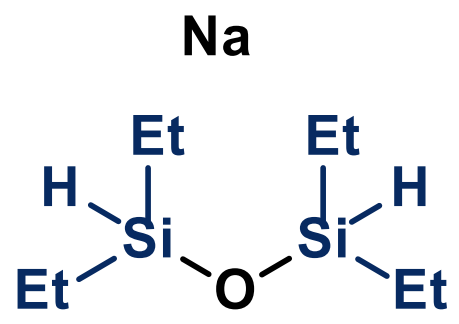

Exact Mass: 213.1107

HR-MS analysis (SFC-QTOF)
$[C_8H_{22}OSi_2 + Na]^+$
m/z 213.1107 (expected),
213.1100 (detected)

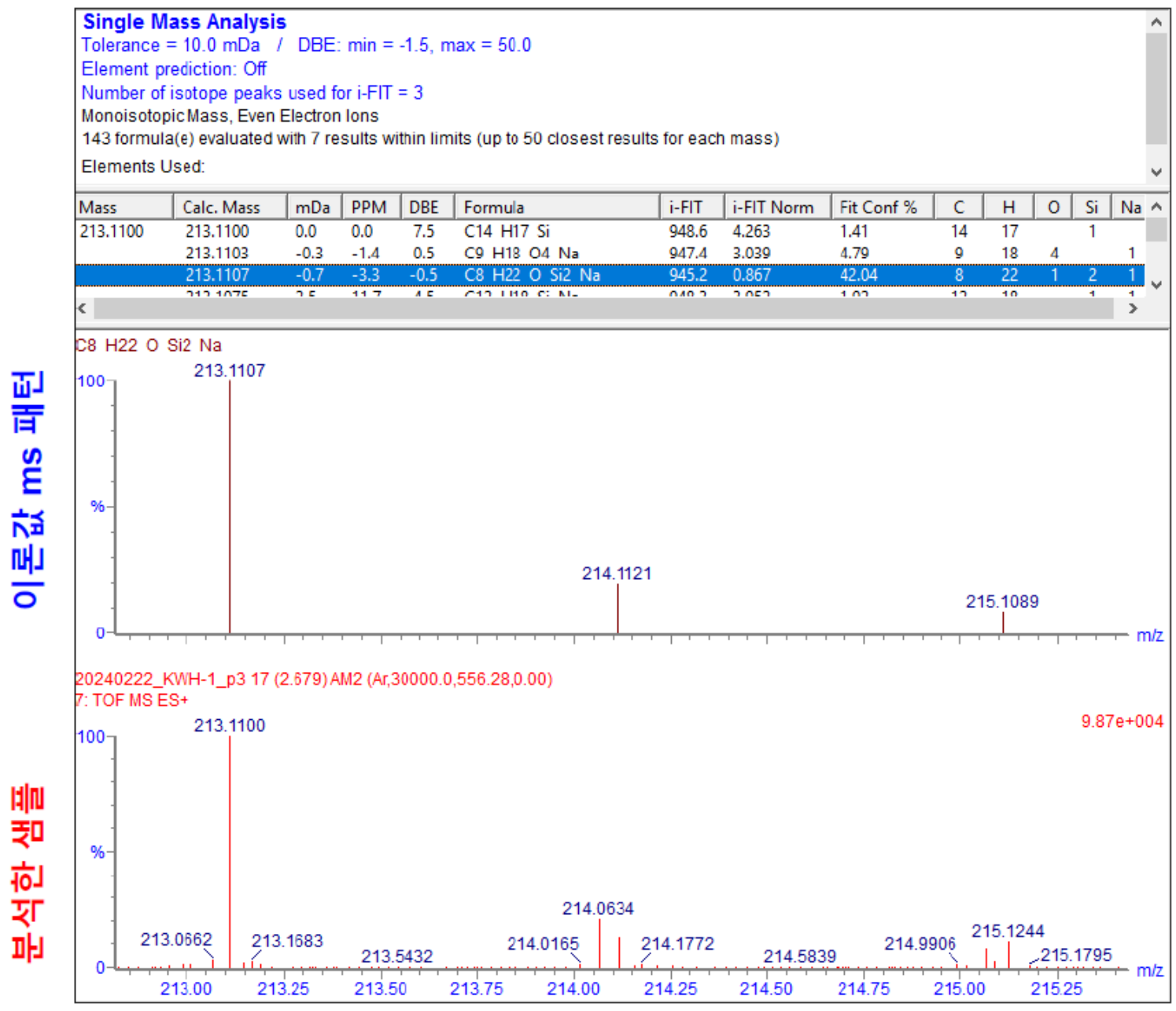

in-situ High-resolution mass spectra (**Et_2HSi-O-$SiHEt_2$**)

11. HPLC spectra of product

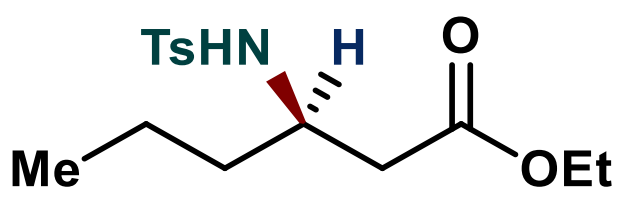

(*R*)-1

HPLC analysis (Chiralpak IA; *n*-heptane/isopropyl alcohol = 90:10; flow rate = 1 mL/min; 210 nm): t_R = 13.13 min (major), t_R = 15.13 min; 94% ee (*R*)

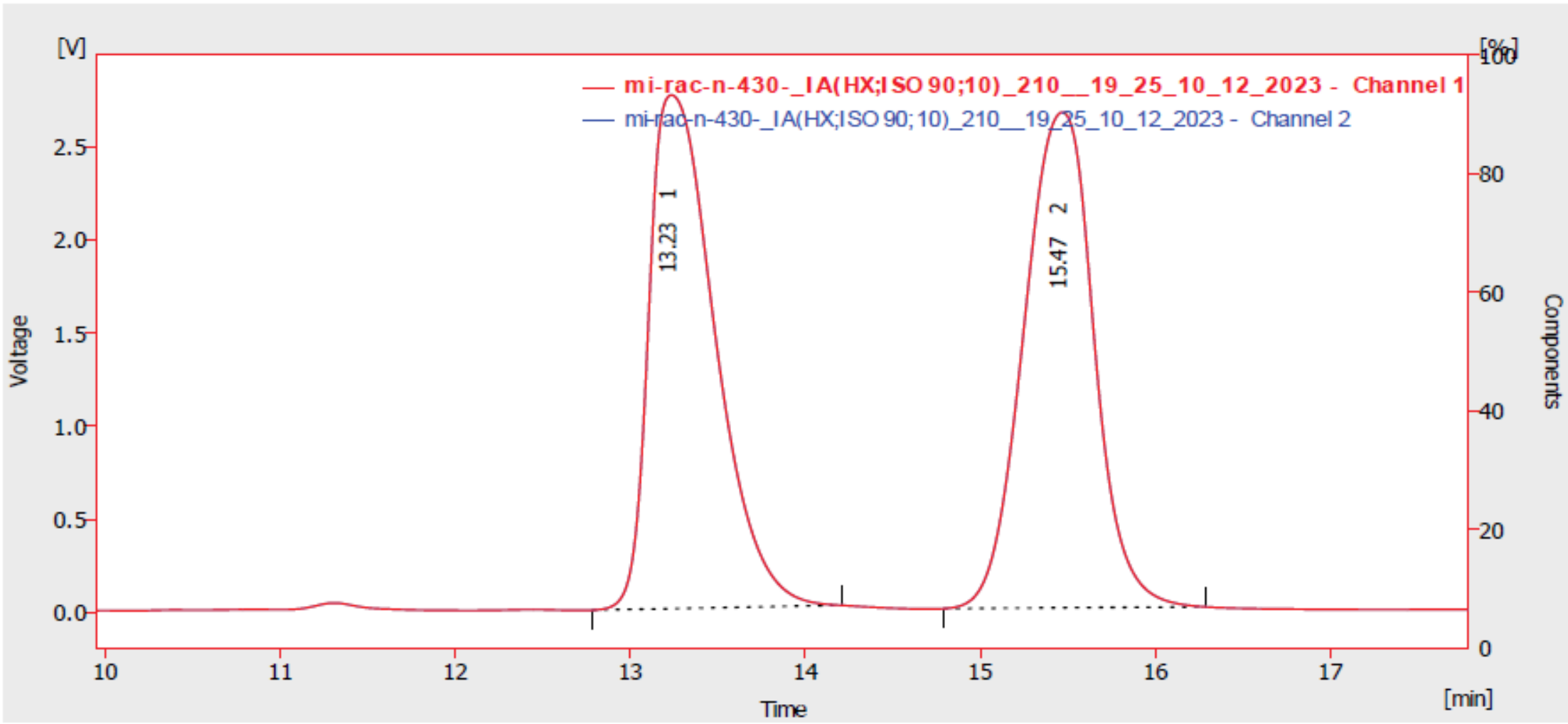

Result Table (Uncal - mi-rac-n-430-_IA(HX;ISO 90;10)_210__19_25_10_12_2023 - Channel 1)

	Compound Name	Reten. Time [min]	Area [mV.s]	Height [mV]	Area [%]	Height [%]	W05 [min]
1		13.235	72251.302	2757.643	49.4	50.9	0.41
2		15.468	74041.216	2660.515	50.6	49.1	0.43
		Total	146292.517	5418.158	100.0	100.0	

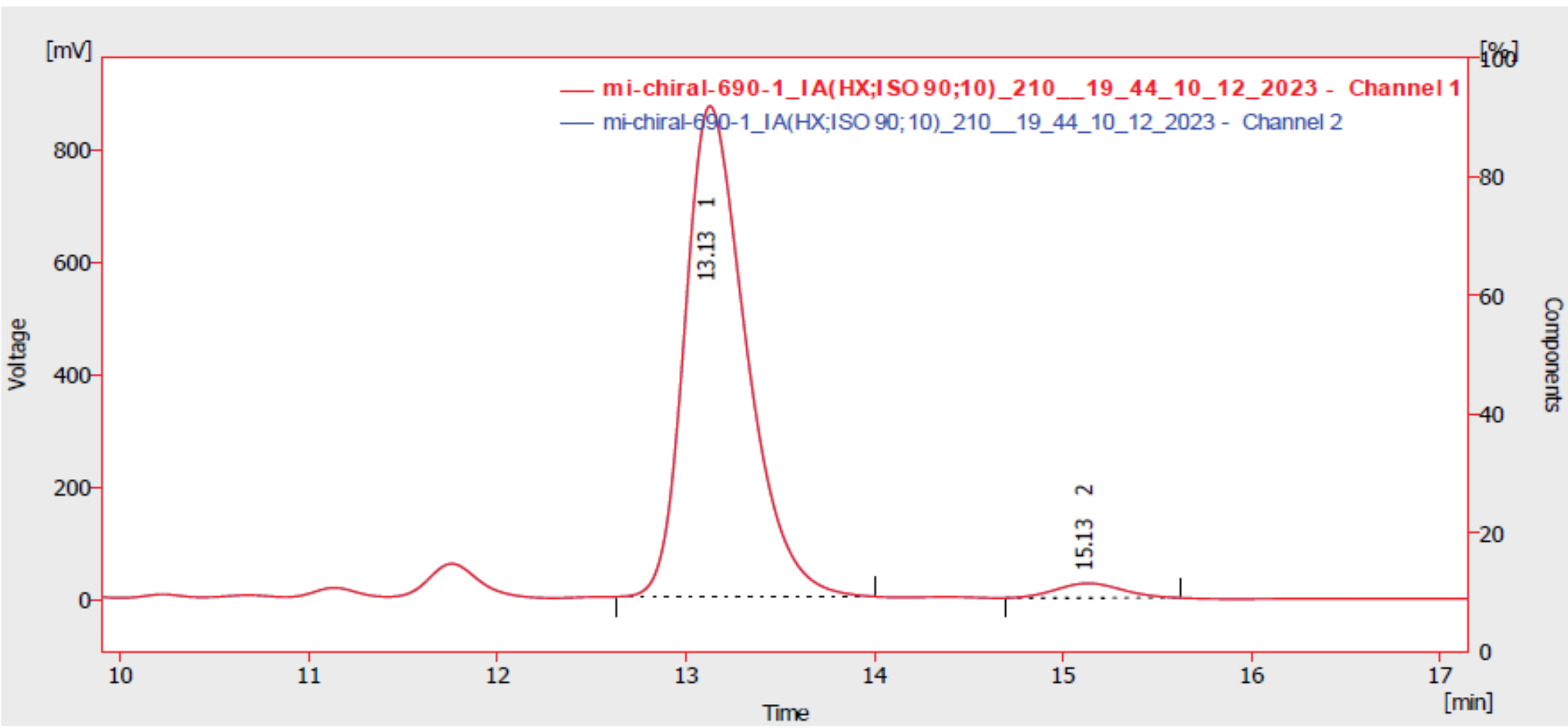

Result Table (Uncal - mi-chiral-690-1_IA(HX;ISO 90;10)_210__19_44_10_12_2023 - Channel 1)

	Compound Name	Reten. Time [min]	Area [mV.s]	Height [mV]	Area [%]	Height [%]	W05 [min]
1		13.128	19666.026	872.361	96.9	97.1	0.34
2		15.133	637.429	26.024	3.1	2.9	0.39
		Total	20303.455	898.385	100.0	100.0	

12. Calculation of E-factors

E-factors were evaluated for product **28** and similar compound, derived from Ru-catalyzed reductive amination reference ('Nat. Commun. 2018, 9, 4123.'). The E-factor of our silylium-catalyzed reductive sulfonamidation was calculated to be 2.44, whereas the Ru-catalyzed reductive amination showed and E-factor of 23.9 (10 mmol scale).

*E-factor

$$\mathbf{A} + \mathbf{B} \xrightarrow[\text{solvent}]{\text{catalyst, reagents}} \mathbf{C} + \mathbf{D} + \textbf{waste}$$

**Mass:**	m_1	+	m_2		m_3	+	m_4	+	m_5
**Moles:**	n_1	+	n_1		n_2	+	n_2	+	-

$$\mathbf{E-factor} = \frac{(\mathbf{m_1} + \mathbf{m_2} + \mathbf{m_4} + \mathbf{m_5}) - \mathbf{m_3}}{\mathbf{m_3}}$$

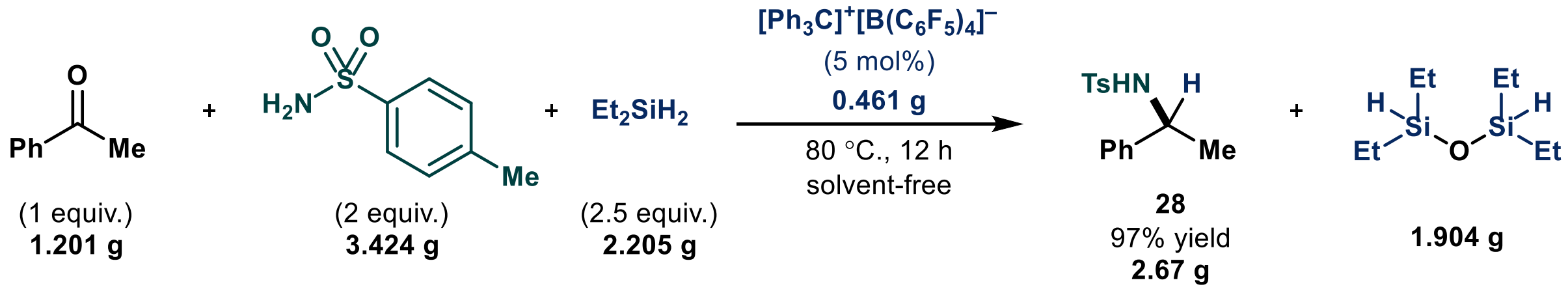

$$\mathbf{E-factor} = \frac{1.201\text{ g} + 3.424\text{g} + 2.205\text{ g} + 0.461\text{ g} + 1.904\text{ g} - 2.67\text{ g}}{2.67\text{ g}} = 2.44$$

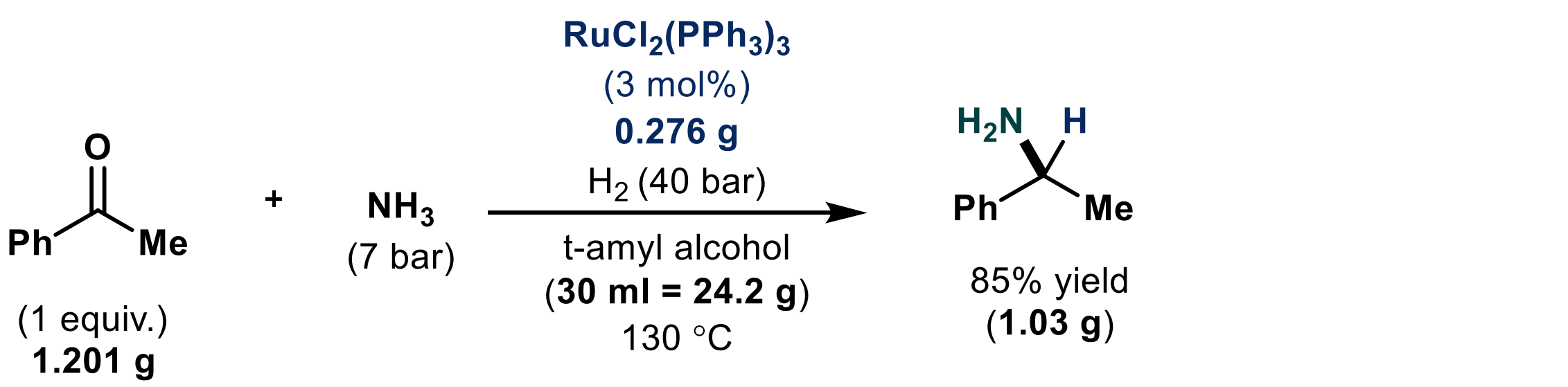

Nat. Commun. **2018**, *9*, 4123

$$\mathbf{E-factor} = \frac{1.201\ \mathrm{g} + 0.276\mathrm{g} + 24.2\ \mathrm{g} - 1.03\ \mathrm{g}}{1.03\ \mathrm{g}} = 23.9$$

13. Supplementary References

1. Ooi, T.; Uematsu, Y.; Maruoka, K. Asymmetric Strecker Reaction of Aldimines Using Aqueous Potassium Cyanide by Phase-Transfer Catalysis of Chiral Quaternary Ammonium Salts with a Tetranaphthyl Backbone. *J. Am. Chem. Soc.* **2006**, *128*, 2548-2549. DOI: 10.1021/ja058066n.
2. Lyu, X.; Seo, C.; Jung, H.; Faber, T.; Kim, D.; Seo, S.; Chang, S. Intramolecular hydroamidation of alkenes enabling asymmetric synthesis of β-lactams via transposed NiH catalysis. *Nat. Catal.* **2023**, *6*, 784-795. DOI: 10.1038/s41929-023-01014-2.
3. Liu, W.; Pan, H.; Tian, H.; Shi, Y. Enantioselective 6-exo-Bromoaminocyclization of Homoallylic *N*-Tosylcarbamates Catalyzed by a Novel Monophosphine-Sc(OTf)$_3$ Complex. *Org. Lett.* **2015**, *17*, 3956-3959. DOI: 10.1021/acs.orglett.5b01779.
4. Ghorai, M. K.; Das, K.; Kumar, A. A convenient synthetic route to enantiopure *N*-tosylazetidines from α-amino acids. *Tetrahedron Letters* **2007**, *48*, 2471-2475. DOI: https://doi.org/10.1016/j.tetlet.2007.02.033.
5. Zhao, L.-M.; Liu, K.; Li, D.-F. One-Pot Synthesis of Aza-Morita-Baylis-Hillman Adducts via Zinc-Mediated Allylation of 4-Bromocrotonates and Imines. *J. Org. Chem* **2019**, *84*, 15429-15436. DOI: 10.1021/acs.joc.9b02434.
6. Anderson, K. R.; Atkinson, S. L. G.; Fujiwara, T.; Giles, M. E.; Matsumoto, T.; Merifield, E.; Singleton, J. T.; Saito, T.; Sotoguchi, T.; Tornos, J. A.; et al. Routes for the Synthesis of (2*S*)-2-Methyltetrahydropyran-4-one from Simple Optically Pure Building Blocks. *Org. Process Res. Dev.* **2010**, *14*, 58-71. DOI: 10.1021/op900163a.
7. Gu, Z.; Comito, R. J. Binucleating Bis(pyrazolyl)alkane Ligands and Their Cationic Dizinc Complexes: Modular, Bimetallic Catalysts for Ring-Opening Polymerization. *Organometallics* **2022**, *41*, 1911-1916. DOI: 10.1021/acs.organomet.2c00167.
8. Zhou, S.; Lv, K.; Fu, R.; Zhu, C.; Bao, X. Nickel/Photoredox Dual Catalytic Cross-Coupling of Alkyl and Amidyl Radicals to Construct C(sp3)–N Bonds. *ACS Catal.* **2021**, *11*, 5026-5034. DOI: 10.1021/acscatal.1c00731.
9. Saha, D.; Taily, I. M.; Banerjee, N.; Banerjee, P. Electricity mediated [3+2]-cycloaddition of N-sulfonylcyclopropanes with olefins via N-centered radical intermediates: access to cyclopentane analogs. *Chem. Commun.* **2022**, *58*, 5459-5462, 10.1039/D2CC00761D. DOI: 10.1039/D2CC00761D.
10. Lu, Z.; Zhang, Y.; Wulff, W. D. Direct Access to N-H-Aziridines from Asymmetric Catalytic Aziridination with Borate Catalysts Derived from Vaulted Binaphthol and Vaulted Biphenanthrol Ligands. *J. Am. Chem. Soc.* **2007**, *129*, 7185-7194. DOI: 10.1021/ja069371r.
11. Muramatsu, W.; Hattori, T.; Yamamoto, H. Substrate-Directed Lewis-Acid Catalysis for Peptide Synthesis. *J. Am. Chem. Soc.* **2019**, *141*, 12288-12295. DOI: 10.1021/jacs.9b03850.
12. Singh, S.; Rath, S.; Bera, S.; Maiti, D.; Sen, S. Catalyst-free electro-photochemical insertion reactions of carbene anion radicals by convergent paired electrolysis. *Cell Rep. Phys. Sci.* **2024**, *5*, 101944. DOI: https://doi.org/10.1016/j.xcrp.2024.101944.
13. Beisel, T.; Diehl, A. M.; Manolikakes, G. Palladium-Catalyzed Enantioselective Three-Component Synthesis of α-Arylglycines. *Org. Lett.* **2016**, *18*, 4116-4119. DOI: 10.1021/acs.orglett.6b02045.
14. Lin, M.; Luo, J.; Xie, Y.; Du, G.; Cai, Z.; Dai, B.; He, L. SuFEx Reactions of Sulfonyl Fluorides, Fluorosulfates, and Sulfamoyl Fluorides Catalyzed by N-Heterocyclic Carbenes. *ACS Catal.* **2023**, *13*, 14503-14512. DOI: 10.1021/acscatal.3c03820.
15. Paz, N. R.; Rodríguez-Sosa, D.; Valdés, H.; Marticorena, R.; Melián, D.; Copano, M. B.; González, C. C.; Herrera, A. J. Chemoselective Intramolecular Functionalization of Methyl Groups in Nonconstrained Molecules Promoted by N-Iodosulfonamides. *Org. Lett.* **2015**, *17*, 2370-2373. DOI: 10.1021/acs.orglett.5b00866.
16. Wang, G.-W.; McCreanor, N. G.; Shaw, M. H.; Whittingham, W. G.; Bower, J. F. New Initiation Modes for Directed Carbonylative C–C Bond Activation: Rhodium-Catalyzed (3 + 1 + 2) Cycloadditions of Aminomethylcyclopropanes. *J. Am. Chem. Soc.* **2016**, *138*, 13501-13504. DOI: 10.1021/jacs.6b08608.
17. Lv, X.-Y.; Martin, R. Cu-Catalyzed C(sp3) Amination of Unactivated Secondary Alkyl Iodides Promoted by Diaryliodonium Salts. *Org. Lett.* **2023**, *25*, 3750-3754. DOI: 10.1021/acs.orglett.3c01216.
18. Nishikata, T.; Nagashima, H. *N* Alkylation of Tosylamides Using Esters as Primary and Tertiary Alkyl Sources: Mediated by Hydrosilanes Activated by a Ruthenium Catalyst. *Angew. Chem. Int. Ed.* **2012**, *51*, 5363-5366. DOI: https://doi.org/10.1002/anie.201201426.
19. Han, J.; Takeda, R.; Sato, T.; Moriwaki, H.; Abe, H.; Izawa, K.; Soloshonok, V. A. Optical Resolution of Rimantadine. *Molecules* **2019**, *24*, 1828.

20. Yang, P.; Zhang, L.; Fu, K.; Sun, Y.; Wang, X.; Yue, J.; Ma, Y.; Tang, B. Nickel-Catalyzed Asymmetric Transfer Hydrogenation and α-Selective Deuteration of *N*-Sulfonyl Imines with Alcohols: Access to α-Deuterated Chiral Amines. *Org. Lett.* **2020**, *22*, 8278-8284. DOI: 10.1021/acs.orglett.0c02921.
21. Wang, Z.; Zhang, Y.; Fu, H.; Jiang, Y.; Zhao, Y. Efficient Intermolecular Iron-Catalyzed Amidation of C−H Bonds in the Presence of N-Bromosuccinimide. *Org. Lett.* **2008**, *10*, 1863-1866. DOI: 10.1021/ol800593p.
22. Verdelet, T.; Ward, R. M.; Hall, D. G. Direct Sulfonamidation of Primary and Secondary Benzylic Alcohols Catalyzed by a Boronic Acid/Oxalic Acid System. *Eur. J. Org. Chem.* **2017**, *2017*, 5729-5738. DOI: https://doi.org/10.1002/ejoc.201700621.
23. Nishimura, T.; Yasuhara, Y.; Hayashi, T. Asymmetric Addition of Dimethylzinc to *N*-Tosylarylimines Catalyzed by a Rhodium−Diene Complex toward the Synthesis of Chiral 1-Arylethylamines. *Org. Lett.* **2006**, *8*, 979-981. DOI: 10.1021/ol060213e.
24. Soeta, T.; Ishizaka, T.; Tabatake, Y.; Ukaji, Y. Chiral N-Heterocyclic Carbene Ligands Bearing a Pyridine Moiety for the Copper-Catalyzed Alkylation of *N*-Sulfonylimines with Dialkylzinc Reagents. *Chem. Eur. J.* **2014**, *20*, 16773-16778. DOI: https://doi.org/10.1002/chem.201404241.
25. Cui, Z.; Yu, H.-J.; Yang, R.-F.; Gao, W.-Y.; Feng, C.-G.; Lin, G.-Q. Highly Enantioselective Arylation of *N*-Tosylalkylaldimines Catalyzed by Rhodium-Diene Complexes. *J. Am. Chem. Soc.* **2011**, *133*, 12394-12397. DOI: 10.1021/ja2046217.
26. Yamada, K.-i.; Yamamoto, Y.; Maekawa, M.; Akindele, T.; Umeki, H.; Tomioka, K. Tin-Free Intermolecular Addition of Primary Alkyls to Imines via the Dimethylzinc−Air Radical Process. *Org. Lett.* **2006**, *8*, 87-89. DOI: 10.1021/ol052563r.
27. Facchetti, G.; Gandolfi, R.; Fusè, M.; Zerla, D.; Cesarotti, E.; Pellizzoni, M.; Rimoldi, I. Simple 1,3-diamines and their application as ligands in ruthenium(ii) catalysts for asymmetric transfer hydrogenation of aryl ketones. *New J. Chem.* **2015**, *39*, 3792-3800, 10.1039/C5NJ00110B. DOI: 10.1039/C5NJ00110B.
28. Chen, Y.; Yang, B.; Li, Q.-Y.; Lin, Y.-M.; Gong, L. Selectfluor®-enabled photochemical selective C(sp3)–H(sulfonyl)amidation. *Chem. Commun.* **2023**, *59*, 118-121, 10.1039/D2CC05569D. DOI: 10.1039/D2CC05569D.
29. Yu, X.; Lu, X. Efficient Synthesis of 9-Tosylaminofluorene Derivatives by Boron Trifluoride Etherate-Catalyzed Aza-Friedel–Crafts Reaction of in situ Generated *N*-Tosylbenzaldimines. *Adv. Synth. Catal.* **2011**, *353*, 569-574. DOI: https://doi.org/10.1002/adsc.201000732.
30. Terrasson, V.; Marque, S.; Georgy, M.; Campagne, J.-M.; Prim, D. Lewis Acid-Catalyzed Direct Amination of Benzhydryl Alcohols. *Adv. Synth. Catal.* **2006**, *348*, 2063-2067. DOI: https://doi.org/10.1002/adsc.200600236.
31. Blay, G.; Cardona, L.; Climent, E.; Pedro, J. R. Highly Enantioselective Zinc/Binol-Catalyzed Alkynylation of *N*-Sulfonyl Aldimines. *Angew. Chem. Int. Ed.* **2008**, *47*, 5593-5596. DOI: https://doi.org/10.1002/anie.200801020.
32. Yan, X.-B.; Li, L.; Wu, W.-Q.; Xu, L.; Li, K.; Liu, Y.-C.; Shi, H. Ni-catalyzed hydroalkylation of olefins with *N*-sulfonyl amines. *Nat. Commun.* **2021**, *12*, 5881. DOI: 10.1038/s41467-021-26194-y.
33. Vargová, D.; Mudráková, B.; Némethová, I.; Šebesta, R. Reductions of Imines Using Zirconocene Chloride Hydride. *Eur. J. Org. Chem.* **2019**, *2019*, 7606-7612. DOI: https://doi.org/10.1002/ejoc.201901607.
34. He, Y.; Li, S.-G.; Mbaezue, I. I.; Reddy, A. C. S.; Tsantrizos, Y. S. Copper-boryl mediated transfer hydrogenation of *N*-sulfonyl imines using methanol as the hydrogen donor. *Tetrahedron* **2021**, *85*, 132063. DOI: https://doi.org/10.1016/j.tet.2021.132063.
35. Vesely, J.; Ibrahem, I.; Zhao, G.-L.; Rios, R.; Córdova, A. Organocatalytic Enantioselective Aziridination of α,β-Unsaturated Aldehydes. *Angew. Chem. Int. Ed.* **2007**, *46*, 778-781. DOI: https://doi.org/10.1002/anie.200603810.
36. Lu, H.; Li, C. General and Highly Efficient Synthesis of 2-Alkylideneazetidines and β-Lactams via Copper-Catalyzed Intramolecular *N*-Vinylation. *Org. Lett.* **2006**, *8*, 5365-5367. DOI: 10.1021/ol062274i.
37. Ravn, A. K.; Vilstrup, M. B. T.; Noerby, P.; Nielsen, D. U.; Daasbjerg, K.; Skrydstrup, T. Carbon Isotope Labeling Strategy for β-Amino Acid Derivatives via Carbonylation of Azanickellacycles. *J. Am. Chem. Soc.* **2019**, *141*, 11821-11826. DOI: 10.1021/jacs.9b05934.
38. Davies, J.; Janssen-Müller, D.; Zimin, D. P.; Day, C. S.; Yanagi, T.; Elfert, J.; Martin, R. Ni-Catalyzed Carboxylation of Aziridines en Route to β-Amino Acids. *J. Am. Chem. Soc.* **2021**, *143*, 4949-4954. DOI: 10.1021/jacs.1c01916.
39. Bae, H. Y.; Kim, M. J.; Sim, J. H.; Song, C. E. Direct Catalytic Asymmetric Mannich Reaction with Dithiomalonates as Excellent Mannich Donors: Organocatalytic Synthesis of (R)-Sitagliptin. *Angew. Chem. Int. Ed.* **2016**, *55*, 10825-10829. DOI: https://doi.org/10.1002/anie.201605167.
40. Tan, G.; Das, M.; Keum, H.; Bellotti, P.; Daniliuc, C.; Glorius, F. Photochemical single-step synthesis of β-

amino acid derivatives from alkenes and (hetero)arenes. *Nat. Chem.* **2022**, *14*, 1174-1184. DOI: 10.1038/s41557-022-01008-w.
41. Lyu, X.; Jung, H.; Kim, D.; Chang, S. Enantioselective Access to β-Amino Carbonyls via Ni-Catalyzed Formal Olefin Hydroamidation. *J. Am. Chem. Soc.* **2024**, *146*, 14745-14753. DOI: 10.1021/jacs.4c02497.
42. Deiana, L.; Dziedzic, P.; Zhao, G.-L.; Vesely, J.; Ibrahem, I.; Rios, R.; Sun, J.; Córdova, A. Catalytic Asymmetric Aziridination of α,β-Unsaturated Aldehydes. *Chem. Eur. J.* **2011**, *17*, 7904-7917. DOI: https://doi.org/10.1002/chem.201100042.
43. Becke, A.D., *Density-functional thermochemistry. III. The role of exact exchange.* The Journal of Chemical Physics, 1993. **98**(7): p. 5648-5652.
44. Weigend, F.; Ahlrichs, R. Balanced basis sets of split valence, triple zeta valence and quadruple zeta valence quality for H to Rn: Design and assessment of accuracy. *Phys. Chem. Chem. Phys.* **2005**, *7*, 3297-3305, 10.1039/B508541A. DOI: 10.1039/B508541A.
45. Frisch, M.J., et al., *Gaussian 16 Rev. C.01*. 2016: Wallingford, CT.
